# Activation of the p53-MDM4 regulatory axis defines the anti-tumour response to PRMT5 inhibition through its role in regulating cellular splicing

**DOI:** 10.1038/s41598-018-28002-y

**Published:** 2018-06-26

**Authors:** Sarah V. Gerhart, Wendy A. Kellner, Christine Thompson, Melissa B. Pappalardi, Xi-Ping Zhang, Rocio Montes de Oca, Elayne Penebre, Kenneth Duncan, Ann Boriack-Sjodin, BaoChau Le, Christina Majer, Michael T. McCabe, Chris Carpenter, Neil Johnson, Ryan G. Kruger, Olena Barbash

**Affiliations:** 1 Epigenetics Discovery Performance Unit, Oncology R&D, GlaxoSmithKline, Collegeville, PA USA; 2 Epizyme, Inc., Cambridge, MA USA; 3 Present Address: Rubius Therapeutics, Boston, MA USA

## Abstract

Evasion of the potent tumour suppressor activity of p53 is one of the hurdles that must be overcome for cancer cells to escape normal regulation of cellular proliferation and survival. In addition to frequent loss of function mutations, p53 wild-type activity can also be suppressed post-translationally through several mechanisms, including the activity of PRMT5. Here we describe broad anti-proliferative activity of potent, selective, reversible inhibitors of protein arginine methyltransferase 5 (PRMT5) including GSK3326595 in human cancer cell lines representing both hematologic and solid malignancies. Interestingly, PRMT5 inhibition activates the p53 pathway via the induction of alternative splicing of MDM4. The MDM4 isoform switch and subsequent p53 activation are critical determinants of the response to PRMT5 inhibition suggesting that the integrity of the p53-MDM4 regulatory axis defines a subset of patients that could benefit from treatment with GSK3326595.

## Introduction

Protein arginine methyltransferases (PRMTs) are enzymes that methylate arginine side chains to generate monomethylation (MMA), asymmetric (ADMA) and symmetric dimethylation (SDMA) on target proteins. PRMT5 activity is responsible for the vast majority of cellular SDMA^1,2^. PRMT5 methylation of the spliceosome is a key event in spliceosome assembly, and the attenuation of PRMT5 activity through knockdown or genetic knockout leads to the disruption of cellular splicing^3^. In addition, PRMT5 methylation of histone arginine residues (H3R8, H2AR3 and H4R3) is associated with transcriptional silencing, and symmetric dimethylation of H2AR3 has been further implicated in the repression of differentiation genes in embryonic stem cells^4^.

Increasing evidence suggests that PRMT5 is involved in tumourigenesis. PRMT5 protein is overexpressed in many cancer types, including lymphoma, glioma, breast and lung cancer. PRMT5 overexpression alone is sufficient to transform normal fibroblasts, while knockdown of PRMT5 leads to a decrease in cell growth and survival in cancer cell lines^5–9^. In breast cancer, high PRMT5 expression, together with high PDCD4 (programmed cell death 4) levels predict overall poor survival^7^. High expression of PRMT5 in glioma is associated with high tumour grade and overall poor survival and PRMT5 knockdown provides a survival benefit in an orthotopic glioblastoma model^8^. Increased PRMT5 expression and activity contribute to silencing of several tumour suppressor genes in glioma cell lines.

Recent studies highlighted PRMT5 as a key regulator of lymphomagenesis. The strongest mechanistic link currently described between PRMT5 and cancer is in mantle cell lymphoma (MCL). PRMT5 is frequently overexpressed in MCL and is highly expressed in the nuclear compartment where it increases the levels of histone methylation and silences a subset of tumour suppressor genes^5^. Recent studies uncovered the role of miRNAs in the upregulation of PRMT5 expression in MCL. It was reported that miR-92b and miR-96 levels inversely correlate with PRMT5 levels in MCL and that the downregulation of these miRNAs in MCL cells results in the upregulation PRMT5 protein levels^5^. Cyclin D1, the oncogene that is translocated in most MCL patients, associates with PRMT5 and increases its activity through a CDK4-dependent mechanism^10^. PRMT5 mediates the suppression of key genes that negatively regulate DNA replication allowing for cyclin D1-dependent neoplastic growth. PRMT5 knockdown inhibits cyclin D1-dependent cell transformation causing death of tumour cells. Additionally, PRMT5 has been implicated as a key regulator of p53 activity in lymphoma models^11^. Increased activity of PRMT5 leads to the methylation and inactivation of p53 in cyclin D1 driven lymphoma models, escaping the need of mutational inactivation of p53^11^. These data suggest that high PRMT5 activity leads to inactivation of p53 in certain genetic and phenotypic contexts, indicating that PRMT5 inhibition could lead to activation of p53 activity and its transcriptional programs in some p53 wild-type cancers.

Here we describe the cellular activity of two potent and selective inhibitors of PRMT5, GSK3203591 and GSK3326595. We demonstrate that PRMT5 inhibition attenuated growth and survival across solid and hematologic cancer cell lines. Lymphoma and breast cancer cell lines were among the most sensitive cell lines tested. Treatment of lymphoma cells with PRMT5 inhibitor induced G1 arrest and subsequent apoptosis in a subset of cell lines. Mechanistic studies demonstrated that PRMT5 inhibition alters gene expression and the splicing phenotype of cells. Alternative splicing events that occur in response to PRMT5 inhibition are enriched in genes that regulate cell cycle progression, suggesting that the splicing phenotype could potentially contribute to the anti-proliferative activity of PRMT5 inhibitors. Importantly, PRMT5 inhibition activated p53 activity in cancer cells through the induction of alternative splicing of the p53 regulator, MDM4. Genome-wide association studies suggest that p53 mutations are among the most highly correlated mutations with the anti-proliferative activity of PRMT5 inhibitors. These data suggest that PRMT5 inhibitors can potentially target many tumour types at least partially due to their ability to regulate cancer relevant pathways, such as p53 and cell cycle. Our data highlight the potential of PRMT5 inhibition as a treatment approach for human cancers and supports the progression of GSK3326595 to clinical trials in cancer patients with solid tumours and lymphoma (NCT02783300).

## Results

### The biochemical potency and mechanism of action of novel PRMT5 inhibitors, GSK3203591 and GSK3326595

**Figure 1:**
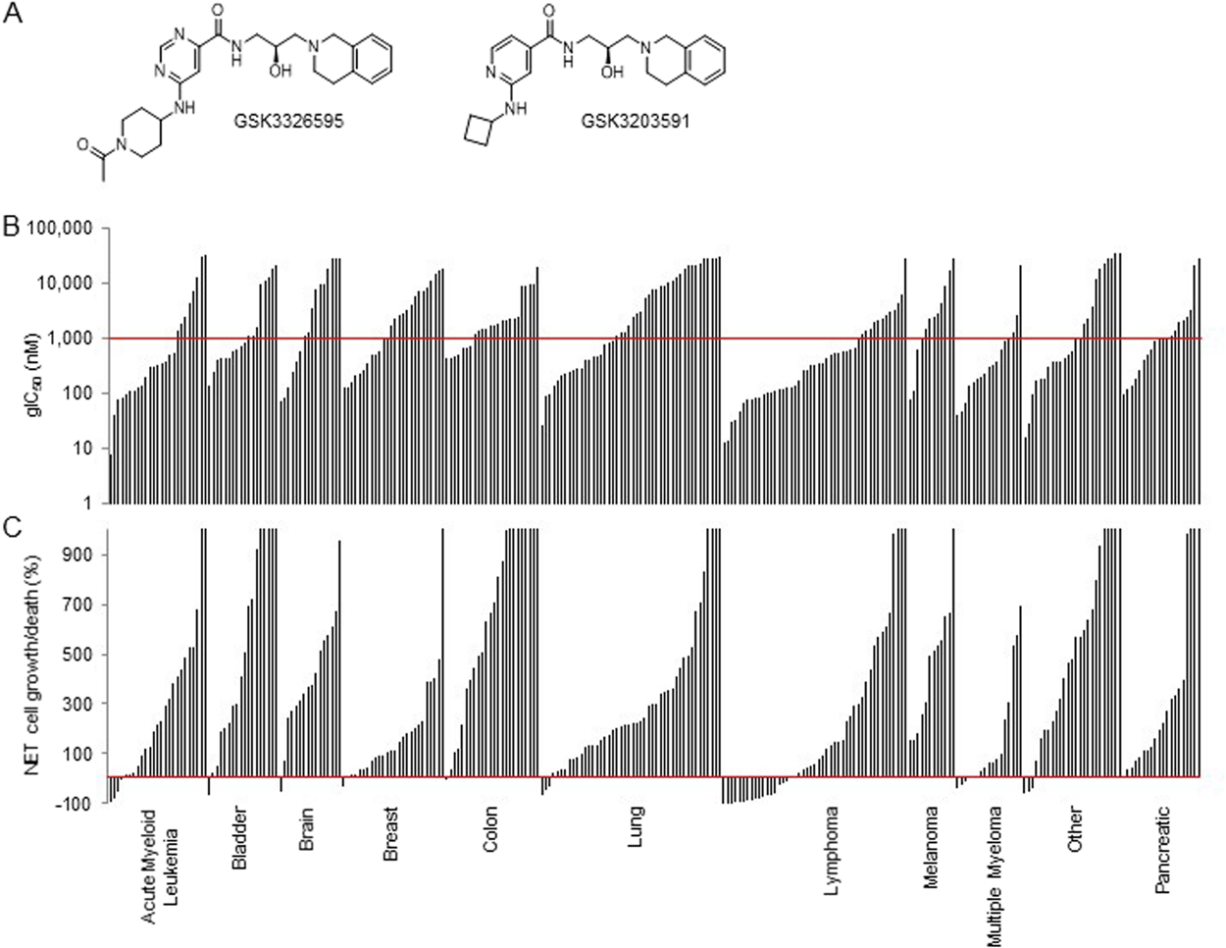
PRMT5 inhibition attenuates growth and survival in cancer cell lines. (**A**) Chemical structure of GSK3326595 (left) and GSK3203591 (right). (**B**) gIC_50_ (nM) and (**C**) net cell growth/death (%) values following a 6-day treatment with GSK3203591 in a panel of cell lines representing various tumour types. Bars represent average values for individual cell lines. Top dose of assay 29 uM. Cell lines with average gIC_50_ < 1 uM and net cell growth/death <0% represent the most sensitive and cytotoxic cell lines, respectively.

The discovery of a chemical probe that selectively targets PRMT5/MEP50 activity was described previously (GSK3235025 (EPZ015666))^12^. GSK3235025 was further optimized through the medicinal chemistry efforts to improve the cellular potency, physicochemical properties and pharmacokinetics parameters to yield more drug-like molecules. Here we describe the activity of two highly selective PRMT5 inhibitors, GSK3203591 (*in vitro* tool molecule) and GSK3326595 (currently in clinical development) (Fig. 1A).

Analysis of the mechanism of inhibition data for GSK3326595 resulted in uncompetitive behaviour with respect to S-adenosyl-L-methionine (SAM) yielding a *K*_i_^app^ value of 43 ± 1 nM (Supplementary Figure S1A, top panel), while the peptide data was indicative of competitive inhibition yielding a *K*_i_^app^ value of 26 ± 1 nM (Supplementary Figure S1A, bottom panel). To determine the binding pocket, GSK3326595 was co-crystallized with the PRMT5/MEP50 complex and sinefungin, a natural product SAM analogue (data not shown). The inhibitor binds in the cleft normally occupied by the substrate peptide, consistent with the binding of EPZ015666^12^. Combined, the enzymatic and crystallographic data supports an inhibitory mechanism that is uncompetitive with SAM and competitive with substrate peptide similar to that observed with GSK3235025.

The inhibitory potency of GSK3326595 increased (decreasing IC_50_ values) with extended preincubation time revealing slow binding inhibition (Supplementary Figure S1B, top panel). This phenomenon was only observed in the presence of SAM, consistent with the SAM uncompetitive inhibitory mechanism where generation of the SAM:PRMT5 complex is required to support binding of GSK3326595. IC_50_ values were determined under conditions that showed near maximal potency (60 minute preincubation) to provide an estimate of the true potency (*K*_i_*^app^). GSK3326595 was found to be a potent inhibitor of PRMT5/MEP50 with an IC_50_ of 6.2 ± 0.8 nM (n = 7, Supplementary Figure S1B, bottom panel), resulting in a calculated *K*_i_^*app^ of 3.1 ± 0.4 nM.

To assess the ability of GSK3326595 to inhibit PRMT5/MEP50-dependent methylation of substrates other than histone H4, a panel of peptides derived from PRMT5 substrates involved in splicing and transcriptional silencing including SmD3, HNRNPH1, H2A and FUBP1 were evaluated (identification of substrates described in Supplementary Figure S2A). Following a 60 minute SAM:Enzyme:Inhibitor preincubation, GSK3326595 inhibited PRMT5/MEP50-catalyzed methylation of all tested peptide substrates resulting in similar *K*_i_^*app^ values of 3.0 ± 0.3, 3.0 ± 0.8, 9.9 ± 0.8, and 9.5 ± 3.3 nM for H2A, SmD3, FUBP1 and HNRNPH1, respectively (n = 4, *K*_i_^*app^ values were calculated from IC_50_ values based on the Cheng-Prusoff equation assuming competitive behaviour Supplementary Figure S1C), suggesting that the identity of the methylated substrate doesn’t impact the ability of GSK3326595 to inhibit PRMT5. Since GSK3326595 is competitive with the protein substrate, the concentration of these protein substrates relative to their *K*_M_ values for PRMT5 could still allow for differential inhibition in cells.

GSK3326595 was >4,000-fold selective for PRMT5/MEP50 over any other enzyme when tested in a panel containing 20 methyltransferases including PRMT9, the other Type II PRMT (IC_50_ > 40 µM, tested with substrate concentrations held near *K*_M_^app^, Supplementary Figure S1D). Selectivity for PRMT5/MEP50 over the other methyltransferases was also observed for GSK3203591. Overall, these data demonstrate that GSK3326595 and GSK3203591 are potent, selective, SAM uncompetitive, peptide competitive, slow binding inhibitors of PRMT5 activity.

### PRMT5 inhibition attenuates growth and survival in human hematologic and solid cancer cell lines

**Figure 2:**
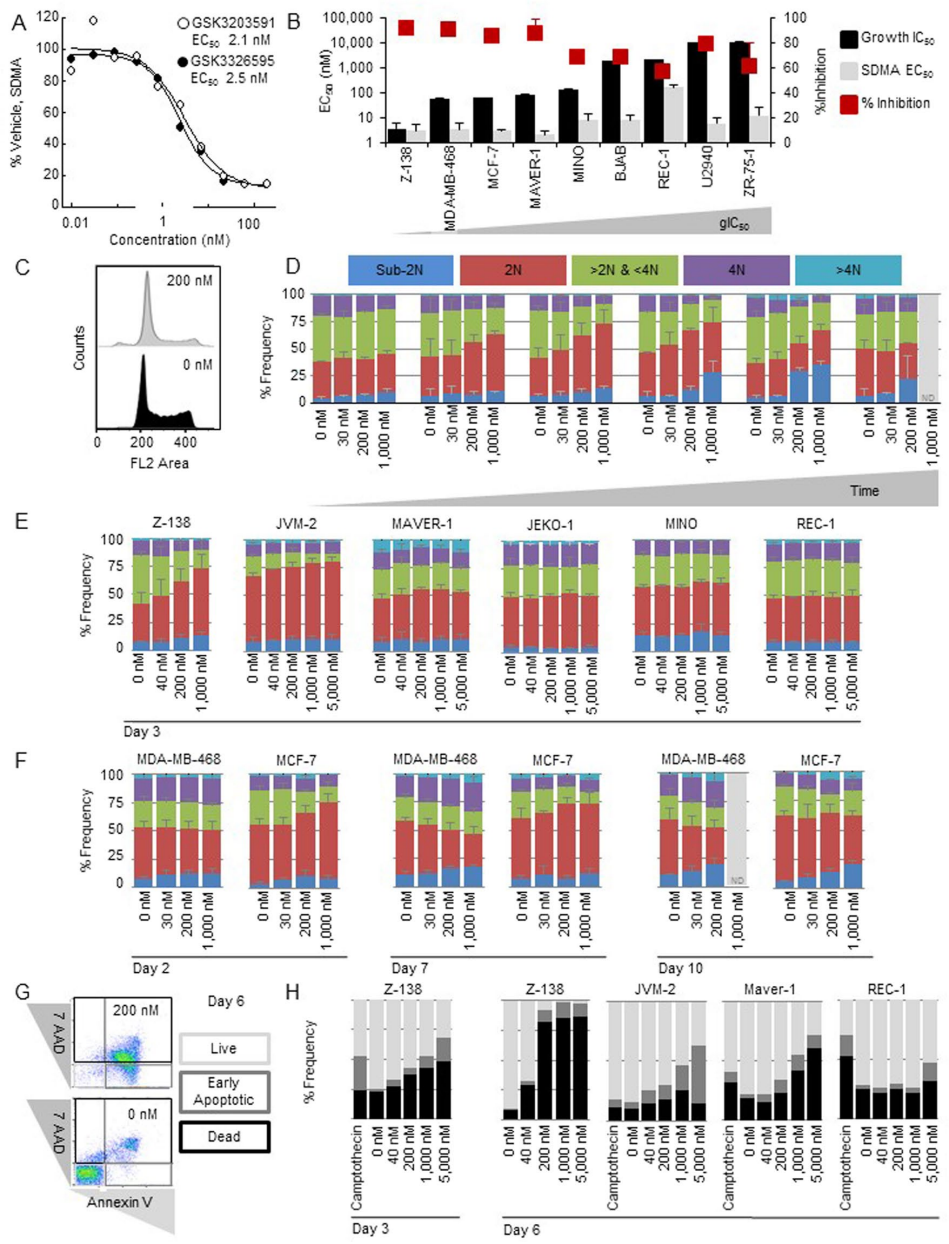
PRMT5 inhibition decreases global cellular SDMA methylation and attenuates cell cycle and viability of cancer cell lines. (**A**) Representative SDMA dose-response curve (total SDMA normalized to SMD3) following 3 days of compound treatment. (**B**) Growth IC_50_ (6 days treatment) in comparison to SDMA EC_50_ and percent decrease in SDMA (maximum minus minimum at concentrations evaluated) in a panel of MCL cell lines (3 days treatment with GSK3326595). (**C**) Representative cell cycle histogram of Z-138 cells treated with 0 or 200 nM GSK3326595 for 3 days. Propidium iodide stained nuclei were evaluated by flow cytometry. (**D**) Cell cycle distribution of Z-138 cells treated with 30, 200 and 1,000 nM GSK3326595 for 1, 2, 3, 5, 7, or 10 days. ND, not determined. (**E**) Cell cycle distribution of a panel of MCL cell lines treated with 40, 200, 1,000, and 5,000 nM GSK3326595 for 3 days. (**F**) Cell cycle distribution of breast cancer cell lines treated with 30, 200, and 1,000 nM GSK3326595 for 2, 7, or 10 days. (**G**) Representative plot of early apoptosis (Annexin V) and cell death (7AAD) markers comparing cells treated with DMSO and cells treated with 200 nM GSK3326595 by flow cytometry. (**H**) Annexin V and 7AAD staining of MCL cell lines treated with 40 nM, 200 nM, 1,000 nM or 5,000 nM GSK3326595 for 3 or 6 days.

To further develop these molecules and to guide clinical strategy, it was necessary to understand sensitivity in preclinical models by assessing the anti-tumour activity of the PRMT5 inhibitors in various cancers. GSK3203591 and GSK3326595 were profiled in a 6-day *in vitro* growth/death assay using a panel of hematologic and solid cancer lines (Fig. 1B,C, Supplementary Table 1). To compare the sensitivity to PRMT5 inhibition between cell lines, growth IC_50_ (gIC_50_) values and net cell growth/death values were calculated. gIC_50_ values for GSK3203591 ranged from 7.6 nM to over 30 µM, but the majority (147/276) of cell lines exhibited values below 1 µM. Lymphoma, including MCL and diffuse large B-cell lymphoma (DLBCL), breast, and multiple myeloma cell lines were among the most sensitive tumour types and had gIC_50_ values in the low nM range (Fig. 1B). PRMT5 inhibition induced net cell death (net cell growth/death < 0%) in a subset of cancer cell lines at concentrations above 100 nM (Fig. 1C). MCL and DLBCL cell lines exhibited the strongest net cell death response. The gIC_50_ values for GSK3326595 and GSK3203591 were similar across the panel of cell lines tested (Supplementary Figure S1E, Supplementary Table 2), and due to similar biochemical and cellular potency, these compounds were used interchangeably in subsequent experiments. Since the growth/survival effects of PRMT5 inhibitors are time-dependent (Fig. 2D,F, Supplementary Figure S5D,E), the anti-proliferative activity of GSK3203591 was further profiled in a panel of 240 cancer cell lines representing 40 different tumour types in a 10-day growth/death assay (Supplementary Figure S1F, Supplementary Table 3). gIC_50_ values ranged from 2.5 nM to over 10 µM, and breast, acute myeloid leukemia, and multiple myeloma were amongst the most sensitive tumour types. Since lymphoma and breast cancer cell lines were among the most sensitive in the proliferation screens, subsequent mechanistic studies were performed in these tumour types.

### PRMT5 is responsible for global cellular symmetric dimethyl arginine (SDMA) methylation of proteins involved in RNA splicing

PRMT5 is responsible for the vast majority of cellular symmetric arginine dimethylation^1,2^. To investigate the biological mechanisms linking PRMT5 inhibition to the anti-proliferative activity in cancer cell lines, methylated PRMT5 substrates were identified using a pan SDMA antibody recognizing a subset of cellular proteins that are symmetrically dimethylated at arginine residues. The identities of the proteins detected by the SDMA antibody were determined in Z-138 cellular lysates (the most sensitive mantle cell lymphoma cell line in a 6-day growth/death assay) from control- and PRMT5 inhibitor-treated cells by immunoprecipitation with the SDMA antibody followed by mass-spectrometric analysis (Methylscan^TM^). Most of the SDMA-containing proteins identified were factors that are involved in cellular splicing and RNA processing (SmB, Lsm4, hnRNPH1 and others), transcription (FUBP1), and translation, highlighting the role of PRMT5 as an important regulator of cellular RNA homeostasis (Supplementary Figure S2A, >2 fold GSK3203591/DMSO changes are shown). While SmB and Lsm4 have been previously reported to be methylated by PRMT5^13^, these data reveal novel PRMT5 substrates, such as hnRNPH1 and FUBP1.

To explore the potential utility of SDMA to be used as a pharmacodynamic biomarker for PRMT5 inhibition, GSK3326595- and GSK3203591-dependent inhibition of cellular SDMA were measured by western and ELISA. First, Z-138 MCL cells were treated with increasing concentrations of GSK3326595 and GSK3203591 to determine the cellular EC_50_ of SDMA inhibition following 3 days of treatment (Fig. 2A). An ELISA revealed changes in SDMA levels with EC_50_ values of 2.5 and 2.1 nM (GSK3326595 and GSK3203591, respectively). Nearly complete inhibition of SDMA was observed at concentrations similar to that required for complete inhibition of net cell growth in Z-138 cells (gIC_100_ = 80.7 nM in a 6-day growth/death assay). These data suggest that complete inhibition of net cell growth and the induction of net cell death in Z-138 cells require near-complete suppression of global SDMA levels. The cellular potency of GSK3326595 (SDMA EC_50_) was further assessed in a panel of breast and lymphoma cell lines representing a range of sensitivities to PRMT5 inhibition (Fig. 2B). SDMA levels decreased in all cell lines treated with GSK3326595 at EC_50_ values of 2 to 160 nM and with a range of maximal percent of inhibition (57–91%). Overall, GSK3326595 inhibited SDMA in all cancer cell lines tested. A trend of more robust inhibition of SDMA was observed in cell lines that are sensitive to PRMT5 inhibition in a proliferation assay.

### PRMT5 inhibition attenuates cell cycle and viability of cancer cell lines

To investigate the mechanism of the growth attenuation by PRMT5 inhibitors, we evaluated the effects of GSK3326595 on the distribution of Z-138 cells in the phases of cell cycle (Fig. 2C). Propidium iodide stained nuclei of Z-138 cells treated with a range of concentrations of GSK3326595 for 1, 2, 3, 5, 7, or 10 days (Fig. 2D) were evaluated by flow cytometry. A dose- and time-dependent G1 arrest (an increase in 2 N DNA) was evident in Z-138 cells treated for 2, 3, and 5 days with a reciprocal decrease in >2 N & <4 N, and 4 N cell populations. After 5 days of treatment, an increase in the sub-2N population (cell death) was observed. We further evaluated the cell cycle profiles of lymphoma and breast cancer cell lines of varying sensitivities to GSK3326595 (Fig. 2E, 3 days post treatment). GSK3326595 treatment led to an increase in the 2 N population in the most sensitive MCL cell lines, Z-138 and JVM-2 (gIC_50 _ < 100 nM) while having no effect on cell cycle distribution in the less sensitive MCL cell lines, JEKO-1, MINO, and REC-1 (gIC_50_ > 100 nM). When tested in breast cancer cell lines, GSK3326595 treatment resulted in an accumulation of MCF-7 cells in the 2 N population after 2 and 7 days of treatment, while in MDA-MB-468 cells the treatment resulted in a decrease in 2 N, and an increase in sub-2N, 4 N and >4 N with 7 and 10 days of treatment. In both MDA-MB-468 and MCF-7, an accumulation of the sub-2N population was observed after 7 and 10 days of treatment, respectively (Fig. 2F). The effects of GSK3326595 in lymphoma and breast cancer cell lines suggest that PRMT5 inhibition results in context-dependent effects on cell cycle (a 2 N population increase was observed only in a subset of sensitive models, while a decrease in 2 N and an increase in 4 N was observed in MDA-MB-468). It is interesting to note that the increase in the 2 N population in response to PRMT5 inhibition, corresponding to a G1 arrest, was observed only in p53 wild-type cell lines (Z-138, JVM-2 and MCF-7).

**Figure 3:**
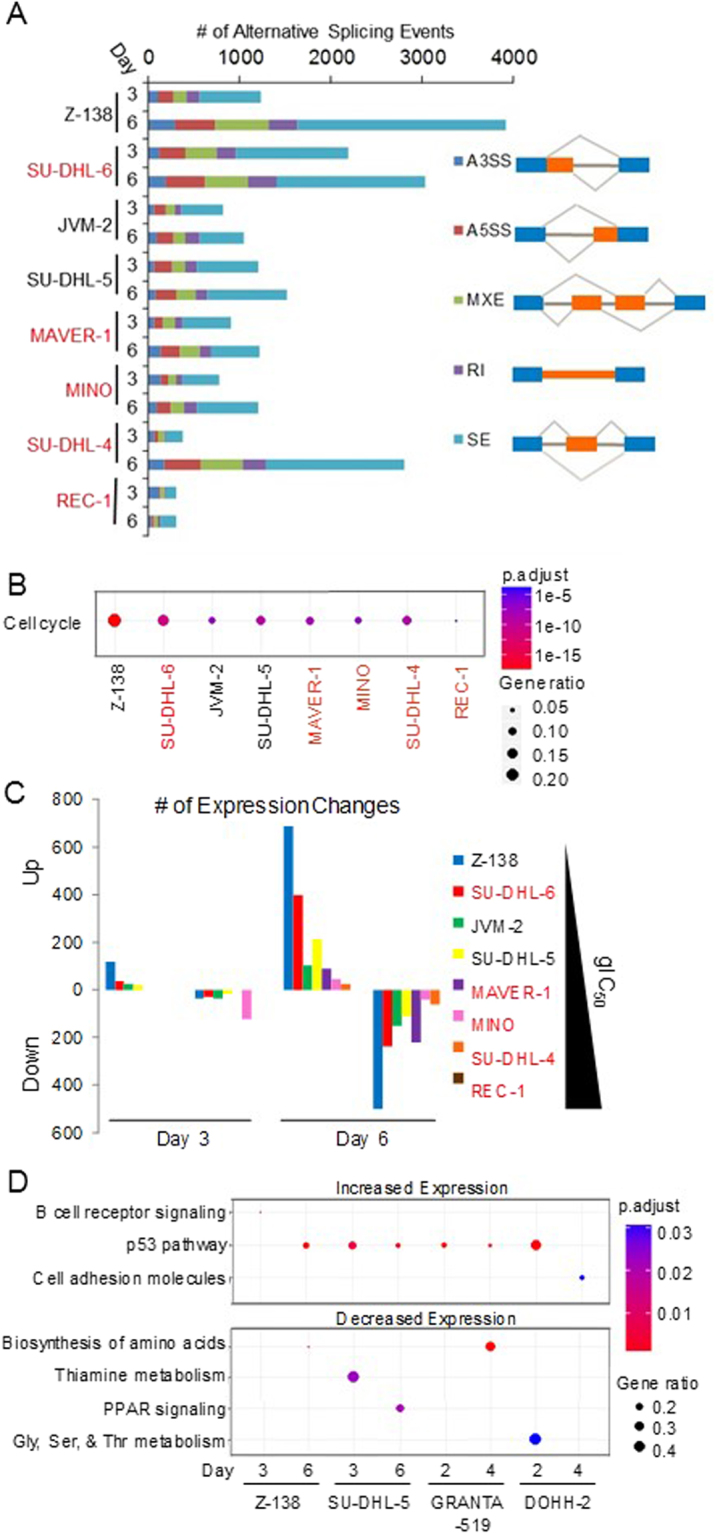
PRMT5 inhibition causes changes in gene expression and splicing. (**A**) The number of significant rMATS alternative splicing events (FDR < 0.01, SD < 0.2, junction coverage > 10, −0.2 < IncLevelDifference >0.2) in lymphoma cell lines treated with 200 nM GSK3326595 for 3 days. p53 mutant cell lines are highlighted in red. (**B**) Significantly alternatively spliced genes were submitted for enrichment of gene sets for each cell line using MsigDB where cell cycle was one of the top gene sets for all cell lines. This cell cycle gene set compiled from all cell lines was analyzed by ReactomePA. p53 mutant cell lines are highlighted in red. (**C**) The number of significant gene expression changes (FDR < 0.05) in lymphoma cell lines following treatment with 200 nM GSK3326595 for 3 or 6 days. Cell lines are sorted by decreasing sensitivity based on gIC_50_ values in a 6-day proliferation assay. p53 mutant cell lines are highlighted in red. (**D**) Significantly changed genes in p53 wild-type cell lines were submitted for MsigDB enrichment analysis (Broad) and the most significantly enriched gene set for each cell line is reported.

To further evaluate the effects of PRMT5 inhibition on cell death, we performed Annexin V and 7AAD staining (markers of early apoptosis and cell death) in a panel of MCL cell lines. Treatment of Z-138 cells led to a dose-dependent increase in early apoptotic and dead cells (Fig. 2G). A dose-dependent increase in early apoptotic and dead cells was also evident in JVM-2 and MAVER-1 after 6 days of treatment (Fig. 2H). In REC-1 cells, GSK3326595 treatment had minimal effect on cell death, consistent with our previous finding that this cell line is resistant to PRMT5 inhibition in a growth/death assay. These data confirm the results of our growth/death assay, which also suggested that PRMT5 inhibition induces cell death in a subset of MCL lines in a dose- and time-dependent manner.

### PRMT5 inhibition impacts cellular splicing and gene expression

Since in our SDMA target identification studies the majority of symmetrically methylated arginines were detected on proteins that regulate RNA splicing, we next sought out to explore the effect of PRMT5 inhibition on cellular splicing by RNA-sequencing (RNA-seq) analysis in a panel of lymphoma cell lines with a range of sensitivities to PRMT5 inhibition. In these datasets, PRMT5 inhibition induced alternative splicing events in thousands of genes, supporting the reported splicing defect^3^ (Fig. 3A). Of note, one of the most enriched gene sets with alternative splicing events across all cell lines tested was cell cycle regulation (Fig. 3B and Supplementary Tables 4–11), suggesting that splicing defects play a role in the cell cycle arrest and cell death phenotype observed upon treatment with PRMT5 inhibitor.

One particular alternative splicing event observed in a PRMT5 knockout mouse model, skipping of MDM4 exon 6, has been linked to p53 pathway activation^14^. The loss of exon 6 in MDM4 is proposed to be a sensing mechanism for splicing defects, whereby the skipping of this exon results in loss of the p53 interacting domain, releasing p53 from MDM4 inhibition^3,15^. MDM4 exon skipping was observed in our RNA-seq experiment of PRMT5 inhibitor-treated cells regardless of p53 status (Supplementary Figure S3A). This was a reproducible event in all cell lines (except for REC-1 cells), as detected by calculating the change in gapped alignment ratios between the splicing events as well as rMATS alternative splicing analysis reported FDR^16^.

Using the same RNA-seq dataset, we further evaluated gene expression changes triggered by PRMT5 inhibition. Cell lines treated with 200 nM GSK3326595 for 3 and 6 days showed a time-dependent induction of many gene expression changes which correlated with sensitivity in the growth/death assay (Fig. 3C and Supplementary Figure S3B). Pathway enrichment analysis revealed that in a subset of lymphoma cell lines of p53 wild-type genotype, GSK3326595 or GSK3203591 treatment lead to a significant upregulation of genes involved in the p53 pathway (Fig. 3D, except JVM-2). Although PRMT5 inhibition did upregulate the p53 pathway genes in JVM-2, the lack of statistical significance could potentially be explained by the high basal expression of p53 target genes in this cell line (Supplementary Fig. S3C and S3D). Gene ontology analysis of gene expression changes induced by PRMT5 inhibition in p53 mutant cell lines did not identify consistent changes in any pathways/gene sets in response to PRMT5 inhibition (Supplementary Figure S3E). This result, along with the observed alternative splicing in MDM4, suggests that the MDM4-p53 pathway is one potential mechanism of growth inhibition and/or cell death in response to PRMT5 inhibition.

### PRMT5 inhibition induces alternative splicing of MDM4 and p53 pathway activity

**Figure 4:**
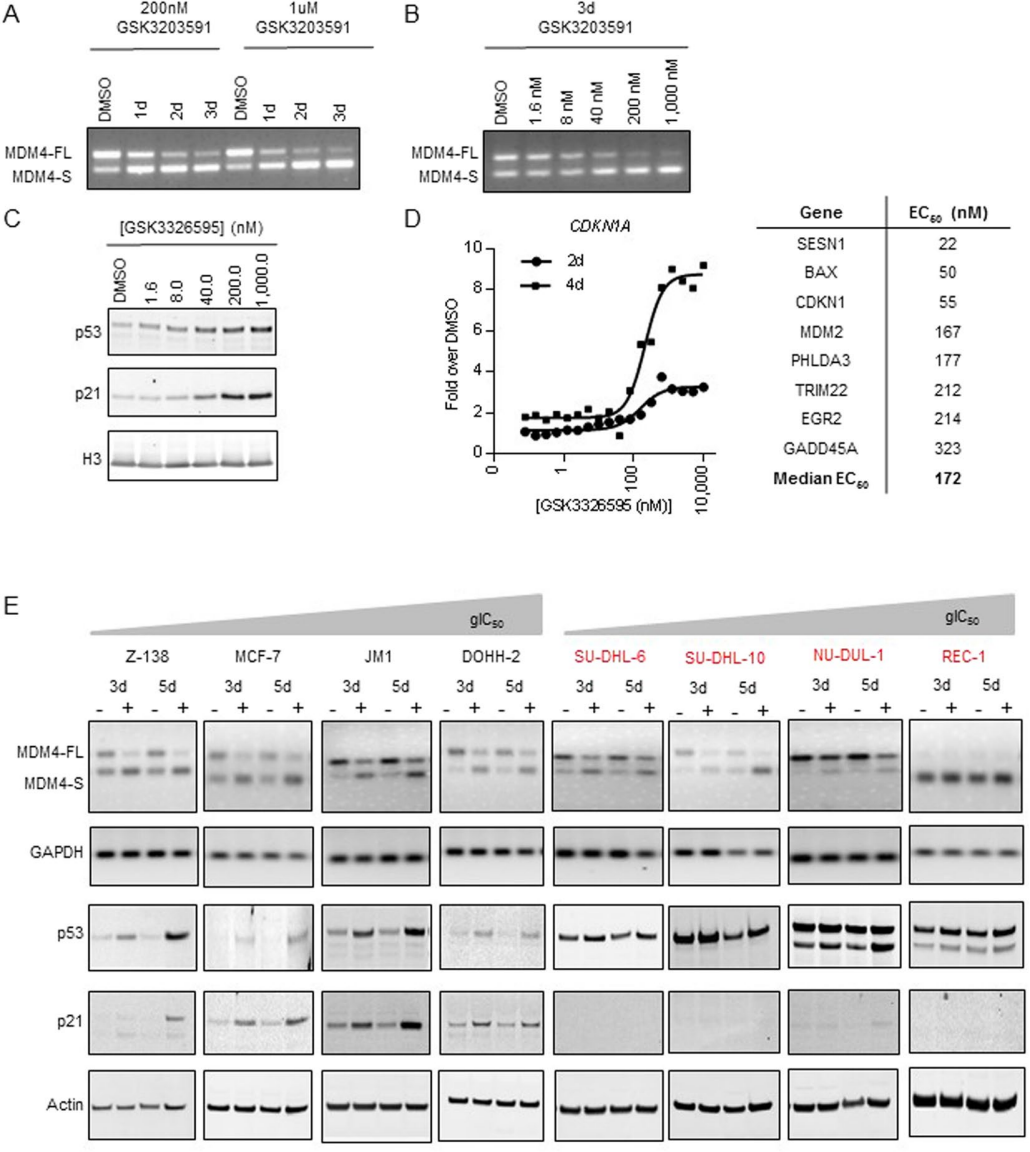
PRMT5 inhibition induces alternative splicing of MDM4 and p53 pathway activation. (**A**) MDM4 splicing time course evaluating relative abundances of MDM4-FL and MDM4-S isoforms in Z-138 following 1, 2, or 3 days of GSK3203591 treatment at concentrations of 200 nM or 1 μM using ethidium bromide gel electrophoresis. (**B**) MDM4 splicing dose response in Z-138 following 3 days of GSK3203591 treatment using ethidium bromide gel electrophoresis. (**C**) Western blot dose response for p53 and p21 protein levels in Z-138 following 3 days of GSK3326595 treatment. (**D**) Gene expression EC_50_ values in Z-138 cells treated with GSK3326595 for 2 or 4 days. Gene panel was selected based on results of RNA-sequencing. Representative dose-response curves for *CDKN1A* (days 2 and 4, left panel) and gene panel EC_50_ summary table (day 4, right panel) are shown. (**E**) MDM4 splicing analysis (via ethidium bromide gel electrophoresis) and p53/p21 induction analysis (via Western blot) in a panel of p53 wild-type (black text) and mutant (red text) breast and lymphoma cell lines treated with DMSO (−) or 200 nM GSK3326595 (+) for 3 or 5 days arranged in order of increasing gIC_50_ value in a 6-day proliferation assay with GSK3326595. MDM4-FL, full-length isoform of MDM4; MDM4-S, short isoform of MDM4 with skipped exon 6.

To further confirm the link between MDM4 splicing and p53 pathway activity, we assessed MDM4 splicing changes by RT-PCR on RNA isolated from Z-138 cells that were treated over a 3-day time course with 200 nM or 1 μM GSK3203591 (Fig. 4A). We confirmed PRMT5 inhibitor-induced alternative splicing of MDM4 from the full-length isoform (MDM4-FL) to the short isoform (MDM4-S) at both treatment concentrations, with the most robust changes after 3 days of compound exposure. Based on these results, a 3-day exposure time was selected to evaluate the dose response of GSK3203591 at concentrations ranging from 1.6 nM to 1 μM (Fig. 4B). MDM4 alternative splicing was evident at GSK3203591 concentrations of 8 nM and above.

Having confirmed that MDM4 undergoes alternative splicing in response to PRMT5 inhibition in Z-138, we next sought to analyze the subsequent p53 pathway activation. p53 and p53-target gene (p21) induction were evaluated in Z-138 (p53 wild-type) cells following a dose response of GSK3326595 treatment with concentrations ranging from 1.6 nM to 1 μM. p53 and p21 protein expression were induced in a dose-dependent manner in Z-138 and JVM-2 cells (Fig. 4C, Supplementary Figure S4A). In Z-138 cells, treatment with GSK3326595 induced p53 and p21 expression to a similar extent as the positive control for p53 activation, Nutlin-3 (an inhibitor of the p53 negative regulator, MDM2) (Supplementary Figure S4A). In contrast to the p53 wild-type cell lines, the p53 mutant cell lines, MAVER-1 and REC-1 (Supplementary Figure S4A), show no induction in p53 or p21 levels in response to PRMT5 inhibition. To further explore the activation of p53, the expression of a subset of p53 target genes was evaluated in Z-138 cells treated with increasing doses of GSK3326595 for 2 or 4 days (Fig. 4D). All the genes tested showed time- and dose-dependent expression changes, with a median gene expression EC_50_ of 172 nM. Importantly, this EC_50_ corresponds to the GSK3326595 concentration at which SDMA inhibition is maximal, suggesting that nearly complete inhibition of PRMT5 activity is required to establish changes in p53-dependent gene expression.

Next, we determined whether PRMT5 inhibition induces an MDM4 isoform switch and p53 activation in a larger panel of lymphoma and breast cell lines (Fig. 4E, Supplementary Figure S4B). An MDM4 isoform switch was observed in response to PRMT5 inhibition with 200 nM GSK3326595 at 3 and 5 days in every cell line tested, except for REC-1 (consistent with RNA-seq data, Supplementary Figure S3A), which has nearly undetectable basal expression of the MDM4-FL isoform (Fig. 4E). PRMT5 inhibition induced p53 and p21 protein levels in all p53 wild-type cell lines evaluated (to varying degree, likely due to the concentration and time point tested). PRMT5 inhibition did not activate the p53 pathway in p53 mutant cell lines, as indicated by the lack of induction of p21 protein levels. Overall, these data suggest that PRMT5 inhibition induces changes in MDM4 alternative splicing (MDM4-FL to MDM4-S isoform switch) and leads to p53 pathway activation in most p53 wild-type cell lines.

### MDM4 isoform switch and p53 activation play an important role in establishing the response to PRMT5 inhibition in p53 wild-type cell lines

**Figure 5:**
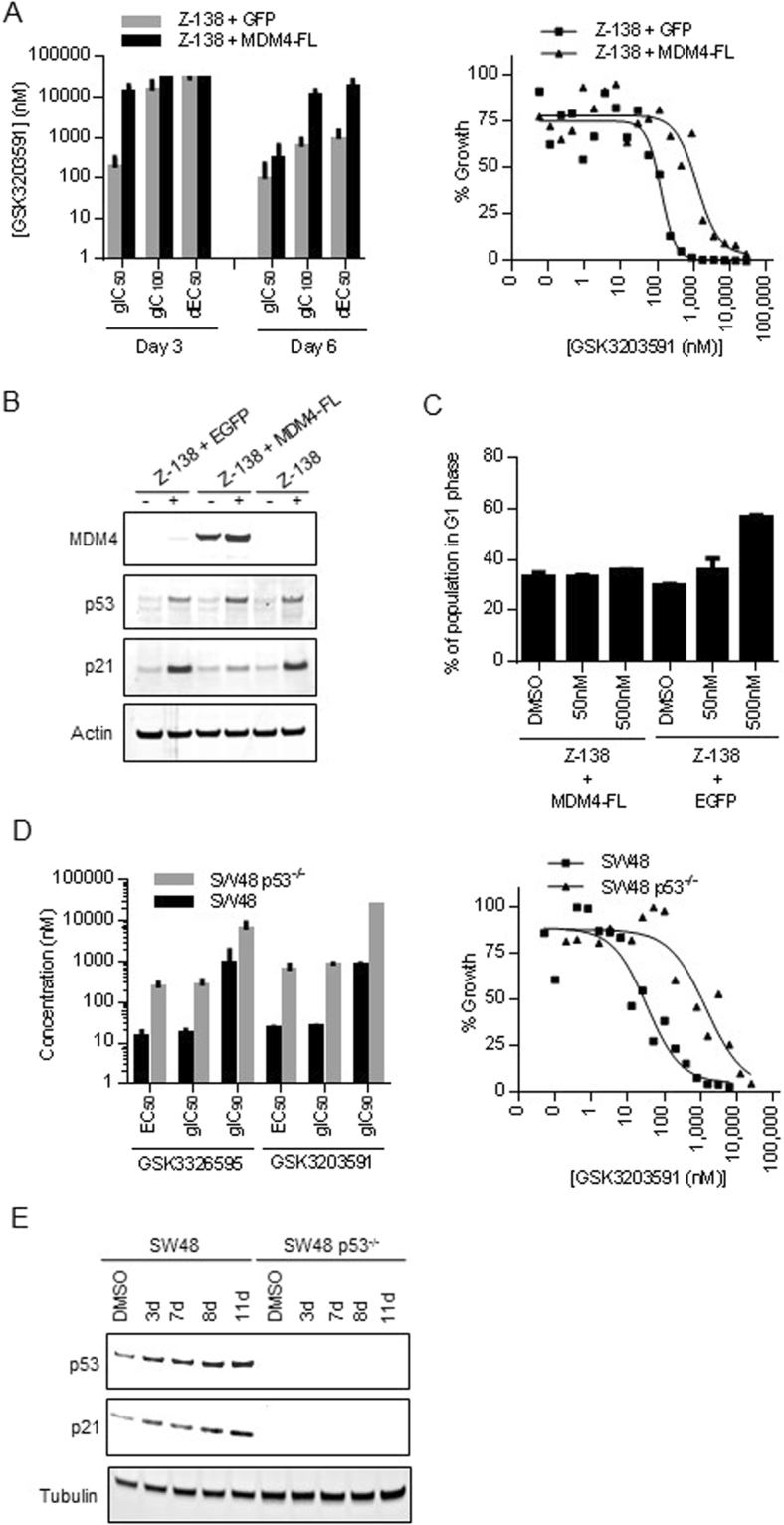
Sensitivity to PRMT5 inhibition is attenuated by overexpression of MDM4-FL or loss of p53. (**A**) (Left panel) Effect of MDM4-FL overexpression on proliferation of Z-138 cells following 3 or 6 days of GSK3203591 treatment with respect to gIC_50_, gIC_100_, and dEC_50_ parameters. Transduction of an EGFP construct was used as a negative control. (Right panel) Representative growth curves comparing % growth relative to Day 0 in Z-138 cells overexpressing MDM4-FL vs. EGFP treated with various doses of GSK3203591 for 6 days. (**B**) Western blot showing the effect of MDM4-FL overexpression on p53 and p21 protein levels in Z-138 cells following 4 days of 1 µM GSK3203591 (+) or DMSO (−) treatment. (**C**) Effect of MDM4-FL overexpression on G1 cell cycle phase in Z-138 cells following 4 days of treatment with DMSO, 50 nM, or 500 nM GSK3203591. (**D**) (Left panel) Effect of p53 knockout on proliferation in SW48 colon cancer cells following 10 days of GSK3203591 or GSK3326595 treatment with respect to EC_50_, gIC_50_, and gIC_90_ parameters. (Right panel) Representative growth curves comparing % growth relative to Day 0 in wild-type or p53 genetic knockout SW48 cells treated with various doses of GSK3203591 for 10 days. (E) Western blot showing the effect of p53 knockout on p53 and p21 protein levels in SW48 cells following a time course (3, 7, 8, or 11 days) of 500 nM GSK3203591 treatment.

To better understand the role of the MDM4 isoform switch and p53 pathway activation in the response of cell lines to PRMT5 inhibition, we evaluated the effects of MDM4-FL expression on the sensitivity of Z-138 cells to GSK3203591. Since PRMT5 inhibition has been shown to reduce cellular levels of MDM4-FL as a result of the isoform switch, we hypothesized that the introduction of exogenous MDM4-FL cDNA expression would prevent the activation of the p53 pathway and rescue the growth/death effects induced by PRMT5 inhibitors. Consistent with this hypothesis, Z-138 cells that express exogenous MDM4-FL showed diminished sensitivity to PRMT5 inhibition compared to control Z-138 cells that express EGFP (Fig. 5A). While MDM4-FL overexpression did not affect p53 protein levels upon PRMT5 inhibition, it did attenuate p21 induction (Fig. 5B, MDM4 antibody recognizes only exogenous MDM4), consistent with the role of MDM4 as a regulator of p53 transcriptional activity^17^. These data suggest that p53 protein levels are induced by PRMT5 inhibition independently of the MDM4 isoform switch. While it is unclear why p53 protein levels are induced, the observed phenotype could be a result of direct methylation of p53 by PRMT5^18^. Additionally, expression of MDM4-FL significantly attenuated G1 cell cycle arrest induced by GSK3203591, most notably at 500 nM of GSK3203591 treatment (Fig. 5C). These data suggest that alternative splicing of MDM4 regulates p53 transcriptional activity and plays a critical role in the growth/death response of Z-138 cells to PRMT5 inhibition.

Next, we tested whether the knockdown of p53 would attenuate the growth phenotype induced by PRMT5 inhibition. Indeed, p53 knockdown in Z-138 cells resulted in reduced sensitivity (Supplementary Figure S5A) as well as an attenuated G1 cell cycle arrest (Supplementary Figure S5B) in response to GSK3203591 treatment. However, the p53 knockdown was incomplete, thus the magnitude of its effect on sensitivity to GSK3203591 was moderate (Supplementary Figure S5C). To evaluate whether a complete loss of p53 would lead to greater effects, we compared the effects of PRMT5 inhibition on growth of the colon cancer cell line, SW48, and a genetic p53 knockout cell line, SW48 p53^−/−^ (Fig. 5D). The p53 knockout cell line had no p53 or p21 expression at any time point evaluated when treated with 500 nM GSK3203591 (Fig. 5E) and had substantially higher EC_50_, gIC_50_, and gIC_90_ values than its wild-type counterpart, suggesting the p53 knockout cell line is more resistant to PRMT5 inhibition. These data highlight the critical role of the MDM4/p53 pathway in defining the cell growth/death phenotype in response to PRMT5 inhibition. Consistently, most p53 wild-type lymphoma cell lines exhibit potent anti-proliferative effects and profound net cell death in response to PRMT5 inhibition, apart from a few, including RCK8 and U2940. In these two cell lines, SDMA inhibition was not complete at the early time points tested (SDMA levels were reduced by 43% in RCK8 and by 79% in U2940 after 3 days of PRMT5 inhibition, compared to 97% in one of the most sensitive cell lines, Z-138) but was improved by later time points (98% and 97% SDMA inhibition in RCK8 and U2940, respectively) (Supplementary Figures S5D, E). This further reduction in SDMA was paralleled by robust effects on sensitivity and net cell growth/death, suggesting that PRMT5 inhibitors are effective at inducing cytotoxicity in the majority of p53 wild-type lymphoma cell lines with various kinetics that mirror SDMA inhibition. These data highlight an important role of p53 activation in defining the cellular growth response to PRMT5 inhibition.

### p53 mutational status associates with sensitivity to PRMT5 inhibition

**Figure 6:**
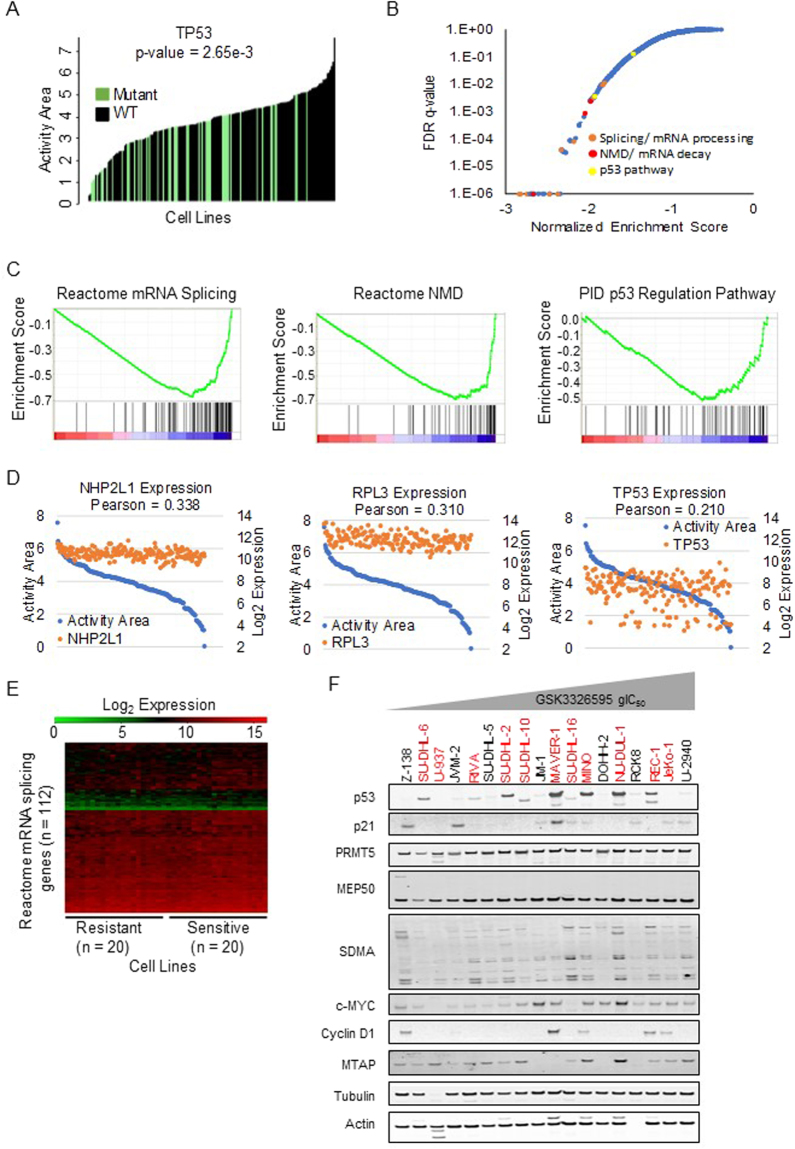
p53 mutational status correlates with sensitivity to PRMT5 inhibition. (**A**) Correlations with activity area calculated from a 10-point dose curve 10 days post treatment with GSK3203591. TP53 mutation is one of the top predictors of resistance. (**B**) A pre-ranked list of Pearson correlation of gene expression with sensitivity was submitted to GSEA and the gene sets plotted by increasing FDR. (**C**) The lowest FDR gene set from each of the three categories of interest: Splicing, Nonsense mediated decay (NMD), and p53 pathway gene sets, are represented by enrichment plots from the GSEA outputs (top panel). The highest correlated gene by Pearson from each of the three represented gene sets is plotted by decreasing sensitivity. (bottom panel). (**D**) A hierarchical clustering of expression values for genes in the “Reactome_mRNA_splicing” gene set from the 20 most sensitive and resistant cell lines. (**E**) Western blot showing basal expression of a variety of proteins in lymphoma cell lines ranging in sensitivity to PRMT5i (most sensitive far left, least sensitive far right) and of various p53 mutational statuses. p53 mutant cell lines in red.

To further explore the role of p53 mutation status as a predictor of sensitivity to PRMT5 inhibition and to identify additional predictive biomarkers, we analyzed the association of the anti-proliferative response to PRMT5 inhibitor across 240 cell lines (Fig. 6A, as defined by activity area or gIC_50_) and the mutation profiles of individual genes in 185 human cancer cell lines for which both proliferation and exome-seq data were available. Wilcoxon rank sum tests revealed TP53 mutations as having one of the most significantly different sensitivities between the wild type and mutant cell lines by either gIC_50_ (2^nd^ most significant) or activity area (10^th^ most significant) (Fig. 6A, Supplementary Tables 12 and 13).

Interestingly, a recent study of 18 cancer cell lines reported that mRNA splicing gene sets were enriched among genes whose basal expression correlated with sensitivity to PRMT5 inhibition, and indicated that expression of p53 pathway genes was not correlated with sensitivity^19^. In order to reproduce this experiment in an expanded cell line panel, the Pearson correlation was calculated for the expression of each gene relative to sensitivity for 178 cell lines, and gene set enrichment analysis (GSEA) was performed on this ranked gene list (Supplementary Tables 15 and 16). Indeed, the most significant gene set from this analysis is mRNA splicing (Fig. 6B). Other gene sets provide evidence that sensitive cell lines may have an increased number of unstable transcripts suggested by high expression of nonsense mediated decay, deadenylation dependent mRNA decay, and destabilization of mRNA by KSRP. However, investigation of the most significant gene in the mRNA splicing (NHP2L1) and nonsense mediated decay (RPL3) gene sets demonstrates that the variability within sensitive and resistant cell lines is larger than the difference between sensitive and resistant cell lines (Fig. 6C, left and middle panels). Hierarchical clustering of splicing gene expression data for the 20 most sensitive and resistant cell lines demonstrates that expression of individual splicing factors is not a robust predictive biomarker of sensitivity (Fig. 6D). The p53 pathway was also found to be significantly correlated with sensitivity (FDR = 0.003749); however, this was primarily due to a disproportionate number of insensitive cell lines lacking p53 expression (Fig. 6C, right panel). The observation that some p53 mutant cell lines also have profound anti-proliferative and net cell death responses to PRMT5 inhibition suggests that additional mechanisms contribute to sensitivity.

Indeed, other markers of sensitivity to the attenuation of PRMT5 activity are described in the literature. Most frequently, the expression of proteins in the PRMT5 complex or regulators of PRMT5 activity is associated with differential sensitivity to PRMT5 knockdown or inhibition (for example, CLNS1A and RIOK1 expression ratio^19^ and MTAP copy number^20–22^). In our lymphoma dataset, the levels of PRMT5 and SDMA did not correlate with anti-proliferative activity (Fig. 6E). Similarly, there was no correlation between CLNS1A/RIOK1 expression and sensitivity to PRMT5 inhibition in a broad GSEA analysis of 178 cancer cell lines (Supplementary Figure S6A). Finally, cell lines with MTAP homozygous deletion and those with at least one functional copy of MTAP were equally sensitive to PRMT5 inhibition (Supplementary Figure S6B). Overall, p53 mutational status was the most highly correlated biomarker for sensitivity to PRMT5 inhibition. It is likely that larger panels of p53 mutant cell lines are necessary to identify predictive biomarkers that are specific to p53 mutant cancers.

### PRMT5 inhibition has profound anti-tumour activity *in vivo*

**Figure 7:**
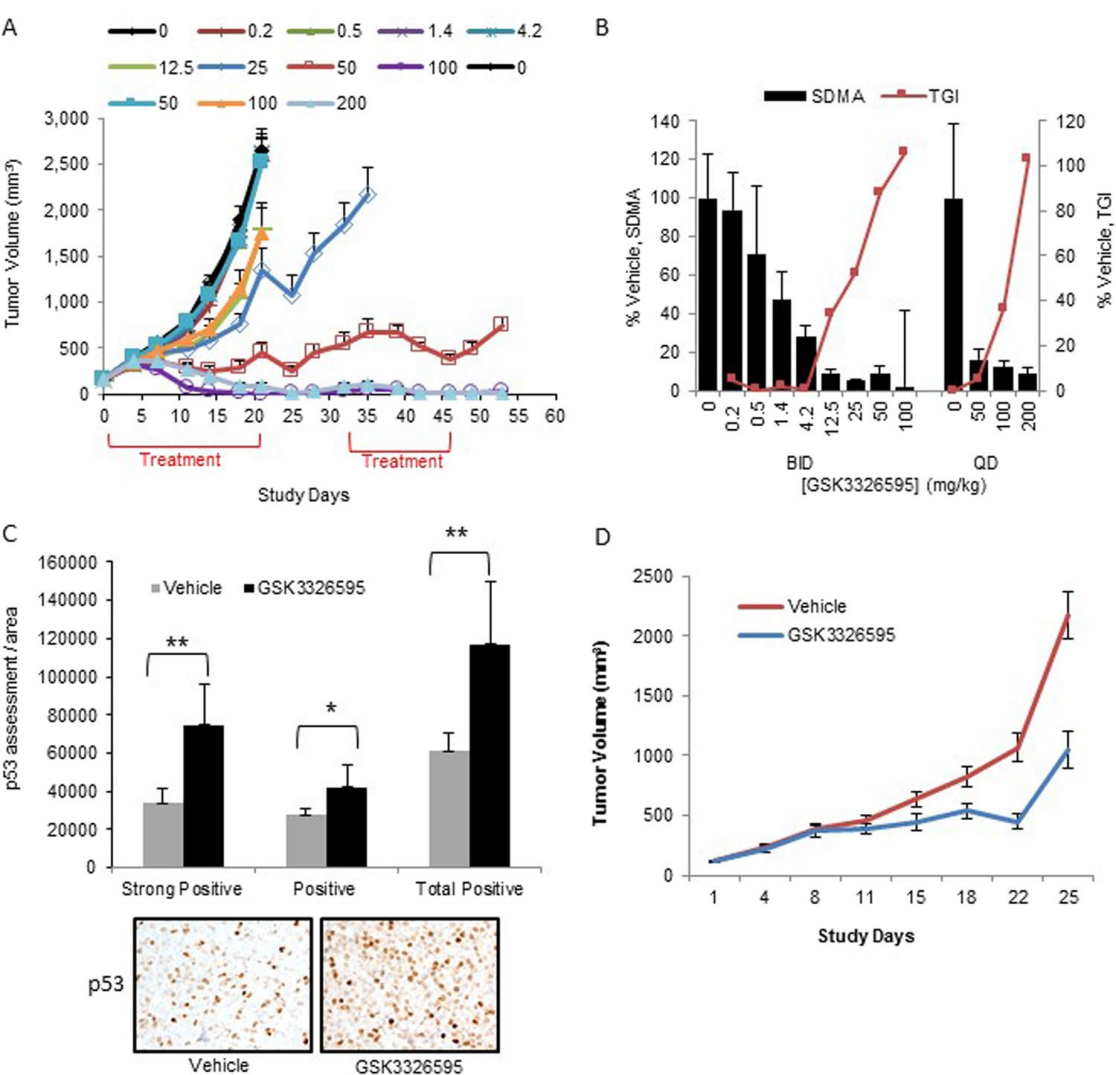
PRMT5 inhibition leads to tumour growth attenuation in lymphoma xenograft models. (**A**) Tumour volume in a Z-138 mouse tumour xenograft with 21 days of GSK3326595 treatment. Groups undergoing treatments that resulted in statistically significant decreases in tumour volume (i.e. 50 mg/kg BID, 100 mg/kg BID and 200 mg/kg QD) were placed on a treatment holiday for 10 days to assess the durability of the response. Treatment was re-instated on day 32 for 2 weeks followed by a 1 week holiday. The 25 mg/kg BID dose group was euthanized on day 35 due to tumour burden. BID doses evaluated include 0, 0.2, 0.5, 1.4, 4.2, 12.5, 25, 50, and 100 mg/kg. QD doses evaluated include 0, 50, 100, and 200 mg/kg. A statistically significant dose-dependent anti-tumour effect was observed for the 25 mg/kg BID, 50 mg/kg BID, 100 mg/kg BID, and 200 mg/kg QD treatment groups (corresponding percent tumor growth inhibition values, % TGI, of 52.1%, 88.03%, 106.05%, and 102.81%, respectively). (**B**) SDMA in Z-138 tumour xenograft model post 7 days treatment with GSK3326595 with twice daily oral dosing. Sampling 2 hours post last dose. Tumour growth inhibition is indicated from a parallel efficacy study. SDMA % vehicle: 94%, 71%, 47%, 28%, 9%, 5%, 8%, 2% in mice given 0.2 mg/kg, 0.5 mg/kg, 1.4 mg/kg, 4.2 mg/kg, 12.5 mg/kg, 25 mg/kg, 50 mg/kg, or 100 mg/kg GSK3326595 BID and SDMA % vehicle: 16%, 12%, and 9% in mice given 50 mg/kg, 100 mg/kg, 200 mg/kg GSK3326595 QD. (**C**) Effects of 100 mg/kg BID GSK3326595 treatment for 7 days on p53 protein levels in Z-138 tumours by immunohistochemistry. (**D**) Tumour volume in a REC-1 (p53 mutant) mouse tumour xenograft treated with 100 mg/kg BID GSK3326595 or vehicle.

To assess whether the anti-tumour activity of PRMT5 inhibition observed for p53 wild-type models *in vitro* translates *in vivo*, we evaluated the efficacy of GSK3326595 in a p53 wild-type Z-138 xenograft model. Since the elimination half-life of GSK3326595 is 1.8 hours in the mouse (data not shown), the efficacy was tested in once daily (QD) and twice daily (BID) schedules. Following 21 days of oral dosing (Fig. 7A), a statistically significant dose-dependent anti-tumour effect was observed in the 25 mg/kg BID, 50 mg/kg BID, 100 mg/kg BID, and 200 mg/kg QD treatment groups (tumour growth inhibition (TGI) of 106.05% for 100 mg/kg BID and 102.81% for 200 mg/kg QD). Comparing QD treatment to the corresponding BID treatments revealed that 25 mg/kg and 50 mg/kg BID treatment schedules were significantly more efficacious (p < 0.01) than the corresponding 50 mg/kg and 100 mg/kg QD treatments starting from day 14. Treatment was withdrawn for 10 days in treatment groups for which significant decreases in tumor volume were observed to assess the durability of the response. Tumour volume increases were observed during the dosing holiday, but resuming treatment with GSK3326595 decreased tumour volume to pre-dosing holiday levels. No significant body weight losses were observed in any of the treatment groups (Supplementary Figure S7A). These data show that the anti-proliferative activity of GSK3326595 observed *in vitro* translates into significant anti-tumor activity in a p53 wild-type *in vivo* model.

To assess the relationship between SDMA inhibition and efficacy, we analyzed the levels of SDMA in Z-138 tumors treated with various doses of GSK3326595 (Fig. 7B). After 7 days of treatment, a dose-dependent decrease in SDMA was evident for both BID and QD treatment schedules. Doses of GSK3326595 that resulted in significant TGI (25, 50, and 100 mg/kg BID and 200 mg/kg QD) also produced substantial decreases in SDMA (SDMA relative to vehicle: 95%, 92%, 98%, and 91%, respectively). Comparison of treatment schedules revealed a trend toward greater efficacy and lower levels of SDMA with BID treatment compared to QD treatment. Overall, the GSK3326595 efficacy/PD relationship in a Z-138 xenograft model suggests that to achieve TGI > 50%, SDMA levels need to be inhibited >80–90%, similar to the relationship noted in *in vitro* studies.

The studies described above confirm that the anti-proliferative effects and corresponding SDMA target engagement resulting from GSK3326595 treatment *in vivo* are consistent with what has been observed *in vitro*. To further confirm *in vitro* studies showing that PRMT5 inhibition induces p53 pathway activity in p53 wild-type cell lines, we next assessed the effects of GSK3326595 treatment on the p53 pathway in these p53 wild-type Z-138 xenograft tumours. We analyzed p53 protein levels from the 100 mg/kg BID treatment group by immunohistochemistry (IHC, post 7 days of treatment, Fig. 7C) and observed an increase in the number of p53 staining positive cells in tumours treated with GSK3326595, consistent with our cell line data. We also observed an increase in the expression of p53 target genes including p21/CDKN1 (Supplementary Figure S7B), confirming induction of p53 activity. These data highlight that the p53 pathway activation observed in *in vitro* experiments translates into *in vivo* models. Importantly, we tested GSK3326595 efficacy in a p53 mutant MCL xenograft model, REC-1, and observed partial (55%) TGI at the 100 mg/kg BID dosing schedule (Fig. 7D), confirming reduced GSK3326595 activity in p53 mutant models.

## Discussion

In this study, we present the first characterization of the activity of potent and selective SAM uncompetitive/peptide competitive PRMT5 inhibitors across a broad range of human cancer models. Protein arginine methylation is involved in the regulation of a variety of biological processes in cells, such as signaling, cell cycle, splicing, transcription, etc.^2^. Our work demonstrates that selective inhibitors of PRMT5 have anti-proliferative and pro-apoptotic activity in solid and hematological models of human cancers and highlights the importance of PRMT5-dependent regulation of cellular splicing as one of the potential mechanisms linked to this broad anti-growth activity.

Most cancer cell lines treated with PRMT5 inhibitors exhibit the attenuation of cell growth and survival. While most gIC_50_ values are in a submicromolar range, the extent of the growth inhibition varies by the tumour type in a 6-day growth/death assay. In our screen, a large fraction of lymphoma, breast and multiple myeloma cell lines exhibited close to complete growth inhibition or a net cell death phenotype, suggesting that these tumour types are the most vulnerable to the attenuation of PRMT5 activity *in vitro*. Cell cycle analysis revealed that in a subset of lymphoma and breast cancer cell lines, PRMT5 inhibition triggers G1 cell cycle arrest and subsequent induction of the sub-G1 cell population. Cell death assays further confirmed that PRMT5 inhibition triggers apoptosis in these cell lines. Our proliferation data highlight that PRMT5 inhibition attenuates proliferation of many cell lines/tumour types and induces apoptosis in a subset of models.

Emerging studies have shown that PRMT5 plays an important role in tumourigenesis through the regulation of epigenetic mechanisms (such as PRMT5-dependent histone arginine methylation^4,5,23^, signal transduction^24,25^, and splicing^3,26^). Our data suggest that cancer cells treated with PRMT5 inhibitors exhibit defects in cellular splicing early, followed up by significant changes in gene expression, highlighting the splicing phenotype as an early event in the response to PRMT5 inhibition. Our numerous attempts to detect reproducible global changes in histone arginine methylation (H4R3me2s, H2AR3me2s and H3R8me2s) were unsuccessful (data not shown), and therefore the contribution of histone methylation to the growth/death phenotype induced by PRMT5 inhibition remains untested.

Previous studies demonstrated that PRMT5 plays an important role in the regulation of splicing^3,26^ in normal tissues and tumour models. Importantly, PRMT5 knockout in the neural progenitor cell compartment in mice leads to defects in the constitutive and alternative splicing of many transcripts, including MDM4. The alternative splicing of MDM4 induced by attenuation of PRMT5 activity leads to the activation of p53 transcriptional activity and subsequent induction of apoptosis in neural progenitor cells. Consistent with these data, we observe that PRMT5 inhibition leads to MDM4 alternative splicing in many cancer cell lines and subsequent activation of p53.

To directly test the role of p53 and MDM4 in the response to PRMT5 inhibition in p53 wild-type cell lines, we compared proliferation and cell cycle effects following PRMT5 inhibition in cell lines with MDM4-FL rescue and p53 knockdown/knockout. We observed that sensitivity, net cell death, and G1 cell cycle arrest were significantly attenuated in the cell lines with p53 knockdown/knockout and MDM4-FL rescue, highlighting the critical role of the MDM4/p53 pathway in defining the cell growth/death phenotype in response to PRMT5 inhibition. An unbiased approach based on a Wilcoxon rank sum test identified TP53 mutation as one of the most significant predictors of resistance to PRMT5 inhibition and GSEA analysis found p53 pathway gene expression to be predictive of sensitivity. These findings demonstrate that activation of the p53 pathway plays an important role in the response to PRMT5 inhibition and cell lines with p53 mutations are overall less sensitive to PRMT5 inhibition.

However, some p53 mutant lines are sensitive to PRMT5 inhibition, suggesting that additional biomarkers of sensitivity may exist. Indeed, MYC expression^26^, CLNS1A and RIOK1 expression ratio^19^, and MTAP copy number^20–22^, have been described in the literature. However, in the panel of 178 cell lines, MTAP status, MYC expression or CLNS1A/RIOK1 expression ratio were not predictive of sensitivity to PRMT5 inhibitor. While the expression of individual splicing genes was not strongly correlated with PRMT5 inhibitor sensitivity, RNA splicing gene sets collectively exhibited a reproducible enrichment. This finding appears to be consistent with the observation that these inhibitors function in part through alternative splicing.

Overall, the data presented in this paper strongly suggest that PRMT5 inhibitors have therapeutic potential in numerous human cancers. While the broad activity offers an opportunity to test PRMT5 inhibitors in many tumour types, it also presents a challenge to identify patient populations that are most likely to benefit from PRMT5 inhibitor treatment. Importantly, we have demonstrated that PRMT5 inhibition activated the p53 pathway via the induction of alternative splicing of a key p53 regulator, MDM4, and that the MDM4 isoform switch together with the induction of p53 transcriptional activity in p53 wild-type models establishes the response to PRMT5 inhibition. Additionally, p53 mutation status and the expression of p53 pathway genes strongly correlate with the anti-proliferative response to PRMT5 inhibition. This suggests that the p53/MDM4 axis status defines one of the cancer patient populations that could potentially benefit from treatment with PRMT5 inhibitors. GSK3326595 is a selective and potent inhibitor of PRMT5 that is currently being tested in a Phase I trial in patients with refractory solid tumours and lymphoma (NCT02783300, clinicaltrials.gov). Future translational studies are needed to further understand biological mechanisms of action of PRMT5 inhibitors and refine additional patient populations that could benefit from this novel agent.

## Methods

Two PRMT5 inhibitors, GSK3203591 and GSK3326595, were obtained from GSK Medicinal Chemistry or Epizyme. Cell lines were obtained from ATCC or DSMZ and were grown in recommended medium, which was most frequently RPMI-1640 containing 10% FBS. Cell line proliferation assays, Western blotting, qRT-PCR, and cell cycle analyses were performed as described previously^27,28^. Antibody and gene expression reagents are as described in Supplementary Table 16. All studies were conducted in accordance with the GSK Policy on the Care, Welfare and Treatment of Laboratory Animals and were reviewed by the Institutional Animal Care and Use Committee either at GSK or by the ethical review process at the institution where the work was performed. Detailed descriptions of all methods can be found in the Supplemental Materials.

### MethylScan®

MethylScan technology (Cell Signaling Technology) was used to evaluate the cellular targets of PRMT5 using a symmetric dimethyl arginine antibody (Cell Signaling, # 13222 S clone D2C3D6).

### SDMA Analysis

SDMA ELISA was performed on homogenized cells and frozen tumour lysates. Fluorescence intensity generated by addition of Luminata Forte (Millipore #WBKUF0500) substrate was measured on a PerkinElmer Envision plate reader.

An SDMA Imaging Assay was used to evaluate the potency of GSK3326595 in breast cancer cell lines. Cells were seeded in optical bottom cell culture plates and targets were probed with fluorescent secondary antibodies and DAPI stain. Quantification of nuclear SDMA and SMD3 levels was performed with a Molecular Devices MetaExpress high-content fluorescence imager and image analysis was performed with ImageExpress software.

### RNA-Seq

RNA was extracted from cell pellets with Trizol using Qiagen’s RNeasy Mini Kit (74104). Libraries were prepared using Illumina TruSeq Stranded Total RNA with Ribo-Zero Gold Library Prep and sequenced on Illumina HiSeq.2500 to 60–80 million PE 2 × 101 bp.

### MDM4 splicing analysis

RNA was isolated from cell pellets as described above and was converted to cDNA using ABI High Capacity Reverse Transcription Reagents (4274966) per manufacturer’s instructions and PCR was performed as described elsewhere^3^. Reaction products were separated using agarose ethidium bromide gel electrophoresis and visualized using a Bio-Rad VersaDoc Model 5000 imaging system.

### Lentiviral Transduction

Lentiviral particles were added at a multiplicity of transduction (MOT) of 20 to Z-138 cells with 50 ug/mL polybrene and cells were centrifuged at 1,000×g for 90 minutes at 37 °C. The virus was washed off and cells were incubated at 37 °C and 5% CO_2_ for 48 hours. Puromycin selection was used to generate and maintain stable cell lines.

### Immunohistochemistry

IHC detection was performed using the HRP/DAB IHC detection kit (ABC kit, #ab64261) and an anti-p53 antibody (Thermo MA5-16387), following the manufacturer’s protocol. Images were taken with AxioVision software, and cell staining intensity was quantified using the Meta Imaging Series software from Molecular Devices.

### Data availability

All data generated or analyzed during this study are included in this published article (and its Supplementary Information files) or are available from the corresponding author on reasonable request. RNA-Seq data is publicly available under Geo accession #GSE108651.

## Electronic supplementary material

Supplementary Dataset 1
