# Supplementary material for: Activation of the p53-MDM4 regulatory axis defines the anti-tumour response to PRMT5 inhibition through its role in regulating cellular splicing
Source: Sci Rep. 2018 Jun 26;8:9711. doi: 10.1038/s41598-018-28002-y (PMC6018746; doi:10.1038/s41598-018-28002-y)
Supplement: Supplementary file 1 — Supplementary Dataset 1 [file 41598_2018_28002_MOESM1_ESM.pdf]

Activation of the p53-MDM4 regulatory axis defines the anti-tumour response to PRMT5 inhibition through its role in regulating cellular splicing.

Sarah Gerhart[1], Wendy Kellner[1], Christine Thompson[1], Melissa Pappalardi[1], Ping Zhang[1], Rocio Montes de Oca[1], Elayne Penebre[2], Kenneth Duncan[2], Ann Boriack-Sjodin[2], BaoChau Le[1], Christina Majer[2], Michael T. McCabe[1], Chris Carpenter[1,3], Neil Johnson[1], Ryan G. Kruger[1], Olena Barbash[1,*]

[1]Epigenetics Discovery Performance Unit, Oncology R&D, GlaxoSmithKline, Collegeville, PA, USA

[2]Epizyme, Inc., Cambridge, MA, USA

[3]Current Affiliation: Rubius Therapeutics, Boston, MA, USA

*Correspondence to olena.x.barbash@gsk.com

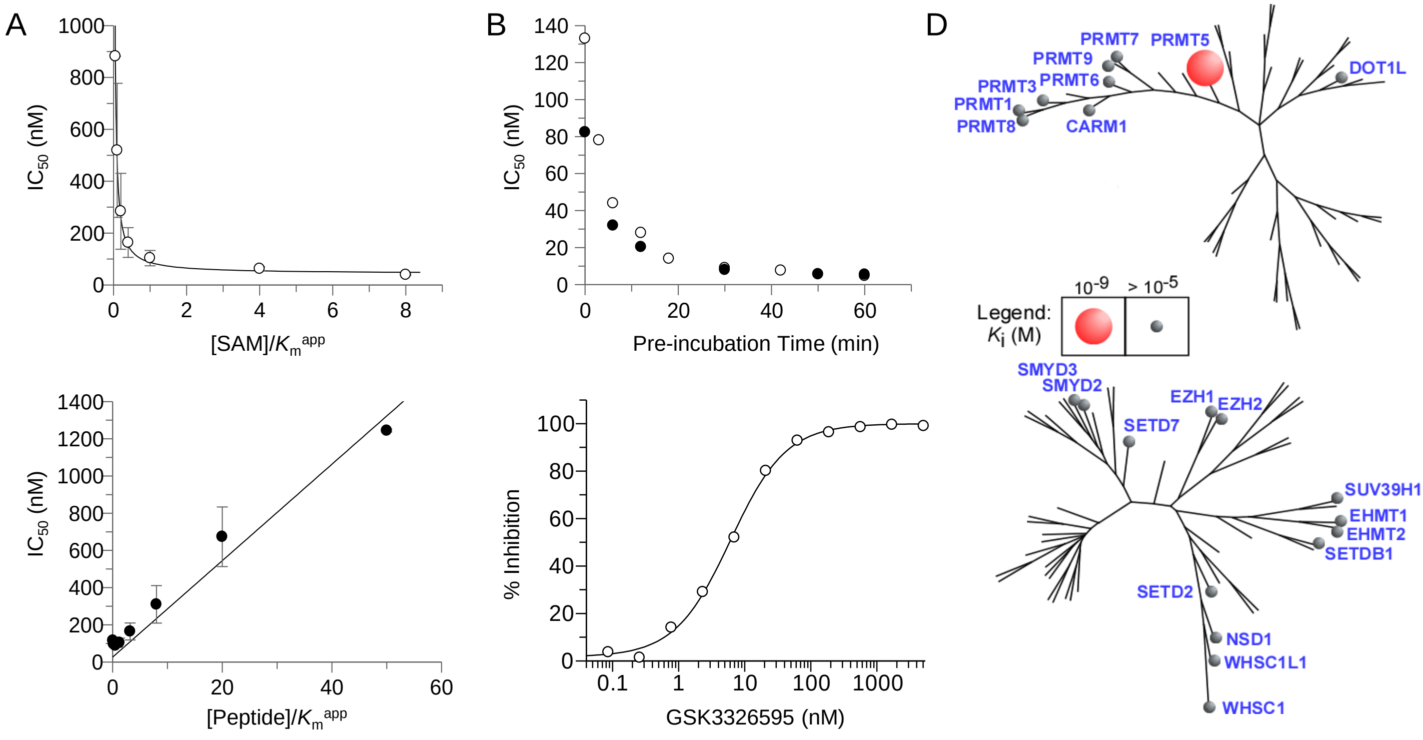

C

Substrate	Amino Acids	Inhibition of PRMT5/MEP50							
		GSK3326595				GSK3203591			
		K_i^{*app} (nM)	IC_{50} (nM)	StdDev	N	K_i^{*app} (nM)	IC_{50} (nM)	StdDev	N
H4	1-21	3.1	6.2	0.8	7	2.8	5.5	1.8	4
H2A	1-20	3.0	6	0.6	4	3.1	6.1	1.5	4
Sm3d	88-106	3.0	5.9	1.5	4	2.4	4.8	0.9	4
FUBP1	349-369	9.9	19.7	1.7	4	23.2	46.3	24.6	4
HNRNPH1	211-231	9.5	18.9	6.5	4	9.8	19.6	4.6	4

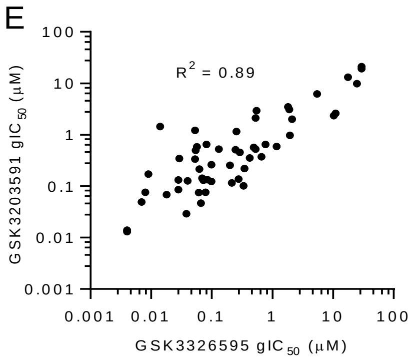

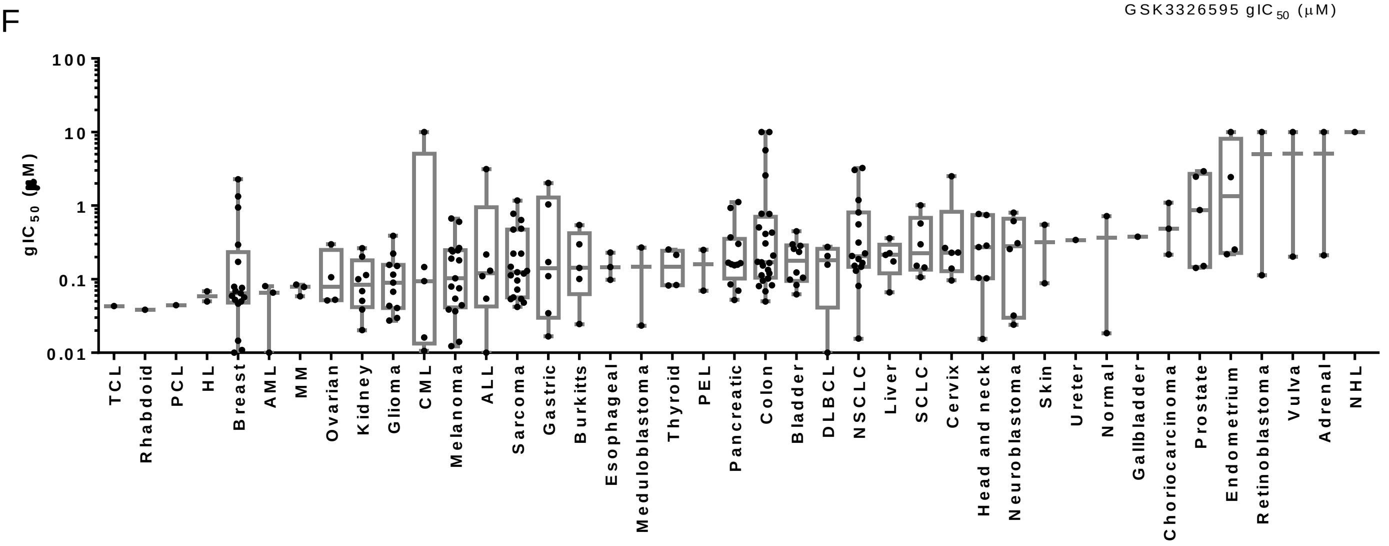

Supplementary Figure S1

Supplementary Figure S1. **(A)** Plots indicating the mode of inhibition against PRMT5/MEP50 by GSK3362595 according to Cheng-Prusoff relationship where IC_{50} values are graphed as a function of substrate concentration relative to K_m^{app} (top panel, SAM; bottom panel, H4 1-21 peptide). Data points (closed vs. open circles) represent two independent experiments. **(B)** IC_{50} values plotted as a function of Enz:SAM:GSK3326595 preincubation time. The open circles represent data generated using 0.8 nM PRMT5/MEP50 while closed circles represent data with 4 nM PRMT5/MEP50 (top panel). The bottom panel shows a representative IC_{50} curve for GSK3362595 inhibition of PRMT5/MEP50 activity following a 60 minute Enz:SAM:Inh preincubation fit to a 3-parameter dose-response equation. **(C)** Averaged IC_{50} values determined following a 60 minute Enz:SAM:Inh preincubation. IC_{50} values were determined by fitting inhibition data to a 3-parameter dose-response equation. K_i^{*app} values were calculated from IC50 values using the Cheng-Prusoff equation for a competitive inhibitor. **(D)** Phylogenetic tree highlighting the methyltransferases tested in the selectivity panel. GSK3326595 showed much greater potency for PRMT5 (10^{-8} M) than for any other tested enzyme (● , > 10^{-5} M). **(E)** Correlation plot comparing gIC_{50} of tool PRMT5 inhibitor, GSK3203591, and candidate PRMT5 inhibitor, GSK3326595, in a panel of cell lines of various tumour types in a 6-day proliferation assay. R^2 value was calculated using the linear regression fitted to these data. **(F)** Box and whisker plots comparing average gIC_{50} values across tumour types following 10 days of treatment with GSK3203591 (TCL- T-cell lymphoma, PCL- plasma cell leukemia, HL- Hodgkin's lymphoma, AML – acute myeloid leukemia, MM- multiple myeloma, CML- chronic myelogenous leukemia, ALL- acute lymphoblastic leukemia, PEL- primary effusion lymphoma, DLBCL- diffuse large B-cell lymphoma, NSCLC- non-small cell lung cancer, SCLC – small cell lung cancer, NHL- non-Hodgkin's lymphoma). Dots represent an average of values for individual cell lines.

A

GSK3203591/DMSO Fold Change	Protein Name	Function
-141.0	LSM4	Splicing
-22.7	ZNF193	Unknown
-16.8	snRNP B1	Splicing
-14.5	snRNP B1	Splicing
-8.3	N-CoR1	Transcription
-8.2	snRNP B1	Splicing
-7.8	KHSRP	Splicing, RNA stability
-6.8	FUBP1	Splicing, RNA stability
-6.6	FUBP1	Splicing, RNA stability
-6.3	KHSRP	Splicing, RNA stability
-5.7	eEF1A1	Translation
-5.3	hnRNP H1; hnRNP H2	Splicing
-5.2	NEURL4	Centriole organization
-5.1	SACM1L	Unknown
-5.0	hnRNP H1; hnRNP H2	Splicing
-4.4	hnRNP H1; hnRNP H2	Splicing
-4.0	FUBP1	Splicing, transcription
-3.8	WDR33	mRNA processing
-3.8	G3BP-2	RNA binding protein
-3.4	hnRNP H1; hnRNP H2	Splicing
-2.8	KHSRP	Splicing, RNA stability
-2.8	TOX	T-cell development

Supplementary Figure S2

Supplementary Figure S2. (A) PRMT5 target proteins were identified using MethylScan^{TM} technology in Z-138. More negative fold change values correspond to the greatest effect.

A

MDM4 splicing	Z-138	SU-DHL-6	JVM-2	SU-DHL-5	MAVER-1	MINO	SU-DHL-4	REC-1
Day 3 FDR	1.0E-11	7.3E-03	3.8E-04	2.4E-03	1.5E-03	9.6E-02	6.9E-02	4.5E-01
Day 6 FDR	1.1E-10	1.6E-01	2.4E-07	1.3E-10	4.6E-02	1.2E-05	0.0E+00	8.5E-01

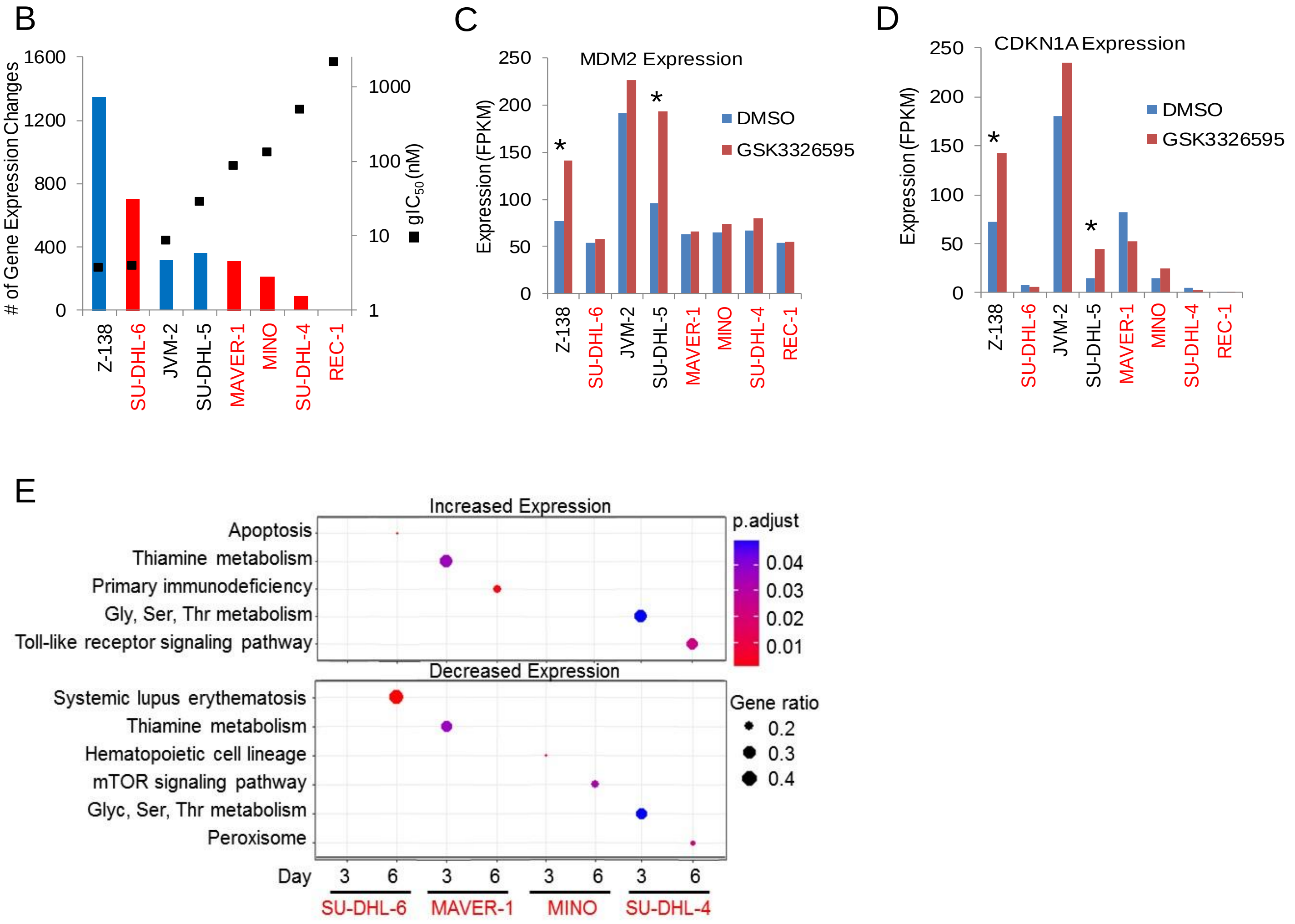

Supplementary Figure S3

Supplementary Figure S3. **(A)** FDR values from rMATS alternative splicing output of MDM4 exon 6 skipping event upon treatment with GSK3326595 for 3 or 6 days. p53 mutant cell lines are highlighted in red. **(B)** The total number of significantly changed genes from days 3 and 6 are plotted against the gIC_{50} for that cell line. The Spearman and Pearson correlations demonstrate a correlation between number of gene expression changes and sensitivity to PRMT5 inhibition. p53 mutant cell lines are highlighted in red. The FPKM values reported from CuffDiff outputs for **(C)** MDM2 and **(D)** CDKN1A. p53 mutant cell lines are highlighted in red. **(E)** Significantly changed genes in p53 mutant cell lines were submitted for MsigDB enrichment analysis (Broad) and the most significantly changed gene set for each is reported.

A

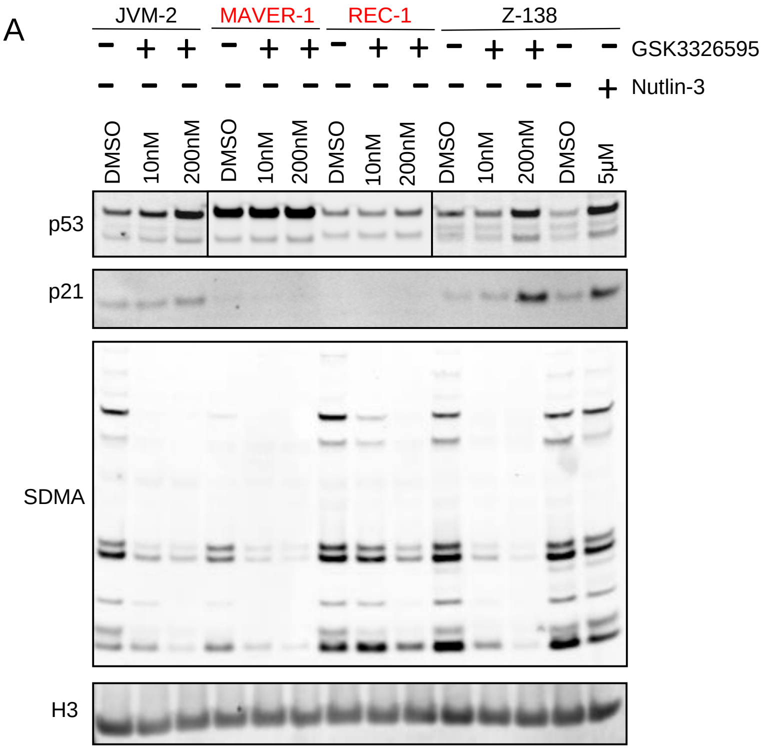
JVM-2
MAVER-1
REC-1
Z-138
GSK3326595
Nutlin-3
DMSO
10nM
200nM
5µM
p53
p21
SDMA
H3

B

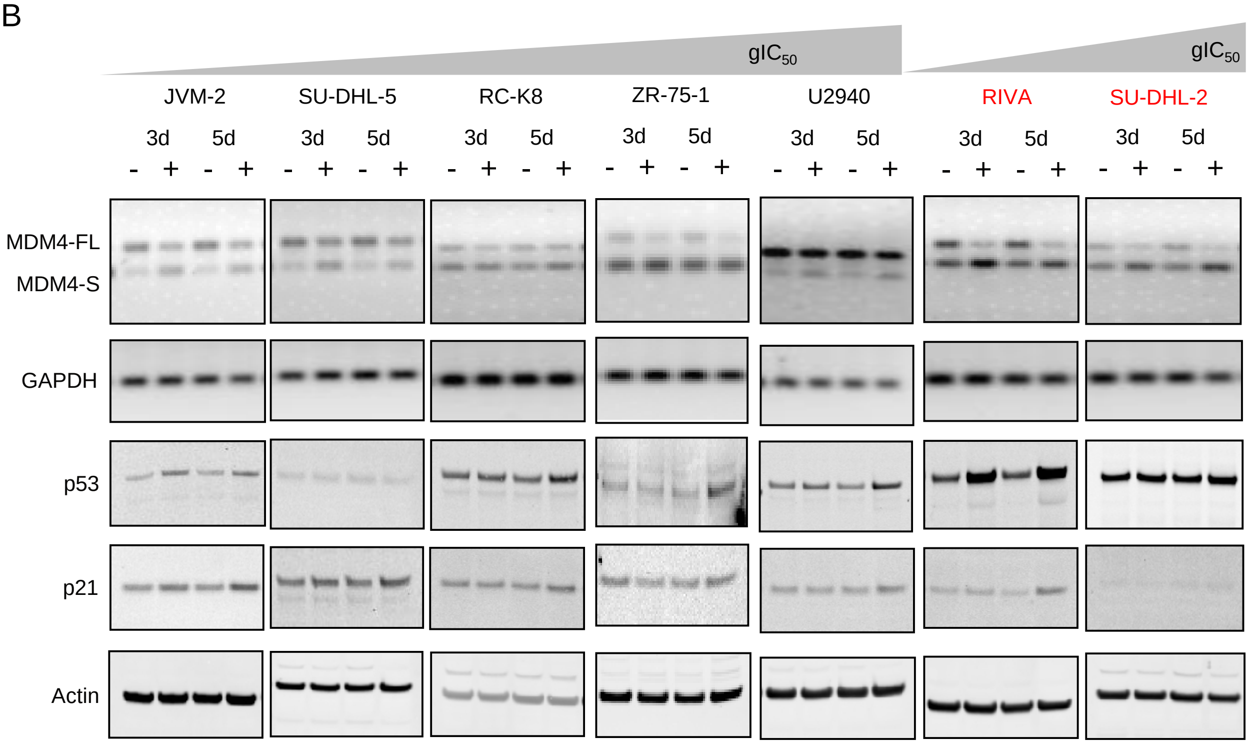
gIC50
JVM-2
SU-DHL-5
RC-K8
ZR-75-1
U2940
RIVA
SU-DHL-2
3d
5d
MDM4-FL
MDM4-S
GAPDH
p53
p21
Actin

Supplementary Figure S4. (A) Western blot showing a p53 and p21 dose response in a panel of MCL cell lines following 3 days of treatment with 10 nM or 200 nM GSK3326595 in a panel of mantle cell lymphoma cell lines. A 5 µM Nutlin-3 treatment condition was also included as a positive control for p53 activation. **(B)** MDM4 splicing and p53/p21 induction in an additional panel of p53 wild-type (black text) and mutant (red text) lymphoma cell lines treated with DMSO (-) or 200nM GSK3326595 (+) for 3 or 5 days arranged in order of increasing gIC_{50} value in a 6-day proliferation assay with GSK3326595.

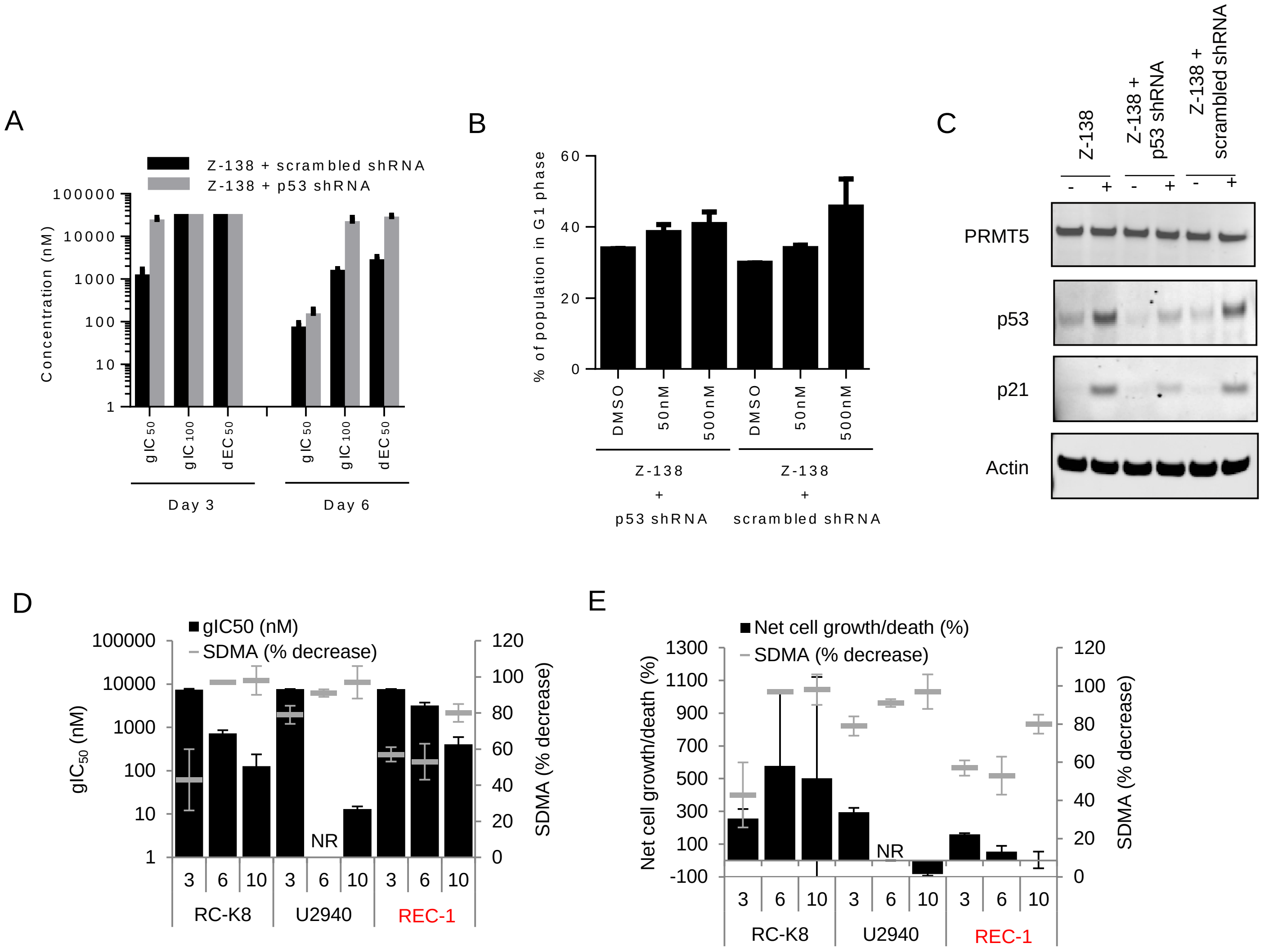

Supplementary Figure S5

Supplementary Figure S5. (A) Effect of p53 knockdown via lentiviral transduction of p53 shRNA on proliferation in Z-138 cells following 3 or 6 days of GSK3203591 treatment with respect to gIC_{50}, gIC_{100}, and dEC_{50} parameters. Transduction of a scrambled, non-targeting shRNA was used as a negative control. **(B)** Effect of p53 knockdown via lentiviral transduction of p53 shRNA on G1 cell cycle phase in Z-138 cells following 4 days of treatment with DMSO, 50 nM, or 500 nM GSK3203591. **(C)** Western blot showing the effect of p53 knockdown via lentiviral transduction of p53 shRNA on p53 and p21 protein levels in Z-138 cells following 4 days of 1 µM GSK3203591 (+) or DMSO (-) treatment. **(D)** gIC_{50} and **(E)** net cell growth/death values from a 6-day growth/death assay with GSK3326595 (black bars) are plotted with % decrease in SDMA (gray rectangles) for 3-, 6-, and 10-day treatment time points. p53 mutant cell lines in red.

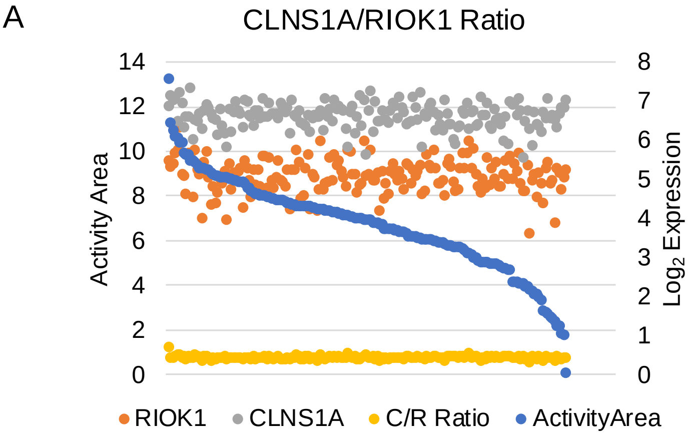

Gene Expression	Pearson r
RIOK1	0.10937
CLNS1A	0.111842
CLNS1A/RIOK1	0.145908

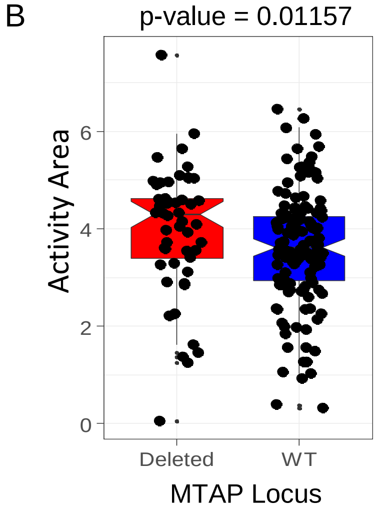

Supplementary Figure S6

Supplementary Figure S6. (A) Gene expression of CLNS1A, RIOK1, and the ratio of the two in 184 cell lines plotted by decreasing sensitivity. Pearson correlations with sensitivity are also shown for each parameter. **(B)** Activity area from proliferation assays are compared between MTAP homozygous deleted cell lines and those with at least one functional copy of MTAP. The result of a Wilcoxon rank sum test is shown.

A

B

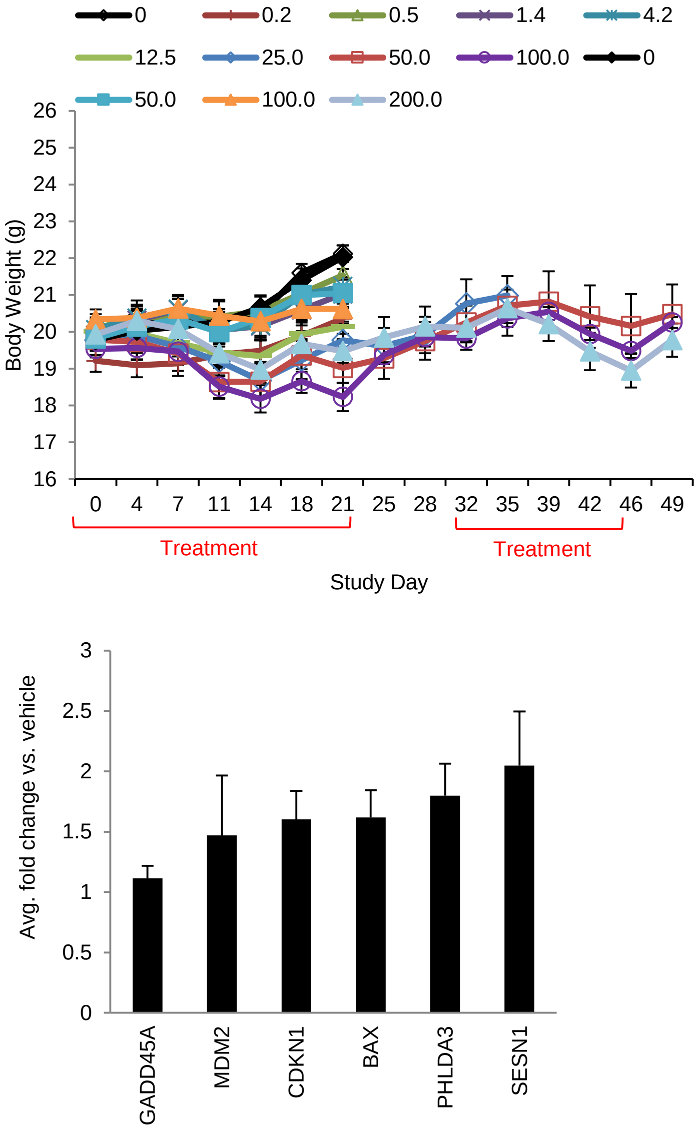

Supplementary Figure S7

Supplementary Figure S7. (A) Body weight changes in S.C. Z-138 tumour bearing mice treated with GSK3326595. Treatments that resulted in significant decreases in tumour volume were placed on a treatment holiday for 10 days. Treatments were reinstated on day 32 for 2 weeks followed by a one week holiday. The 25 mg/kg dose group were euthanized on day 35 due to tumour burden. Day 53 data not available for this data set. **(B)** p53 target gene expression change in Z-138 tumor xenografts after 7 days of 100 mg/kg BID GSK3326595 treatment compared to vehicle control.

Full scans to accompany Figure 4A

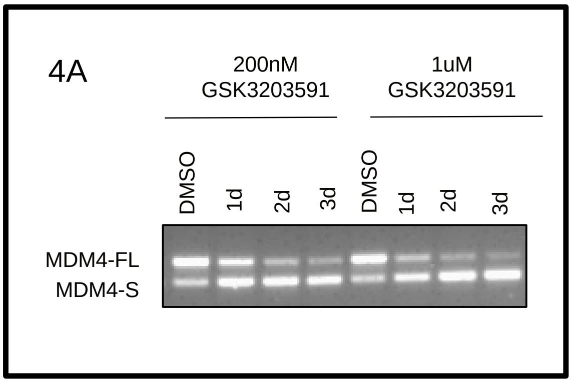

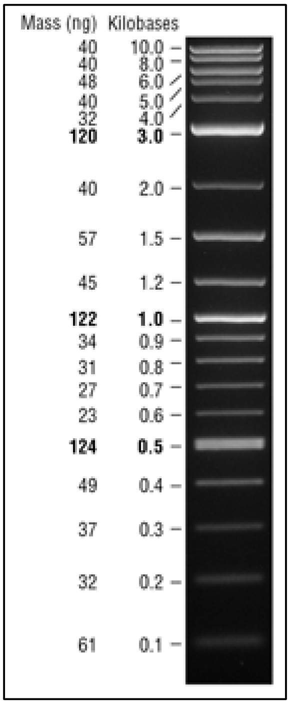

Quick-Load 2-Log DNA Ladder
(New England BioLabs #N0469S)

MDM4

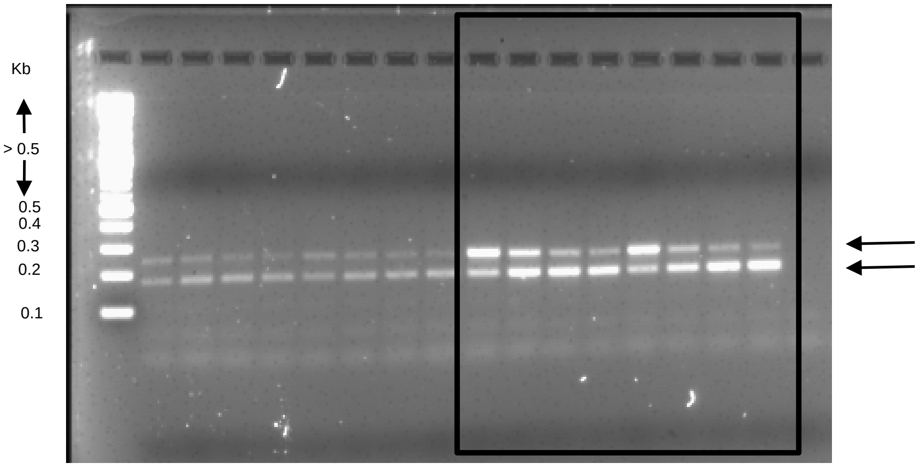

Full scans to accompany Figure 4B

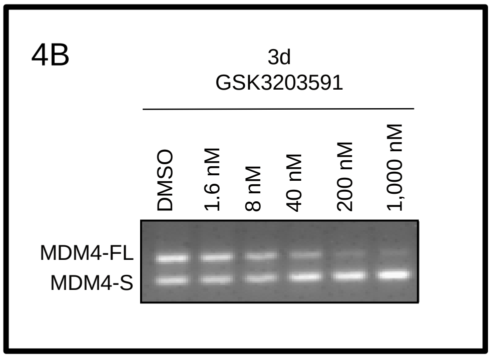

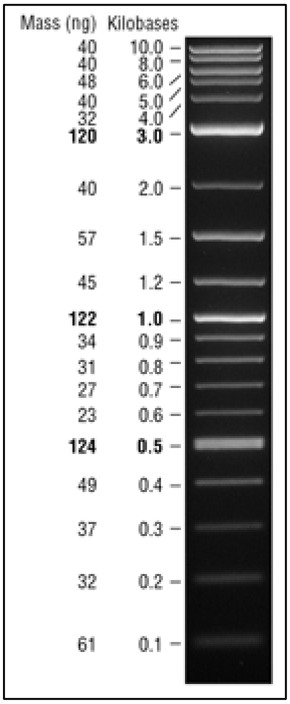

Quick-Load 2-Log DNA Ladder
(New England BioLabs #N0469S)

MDM4

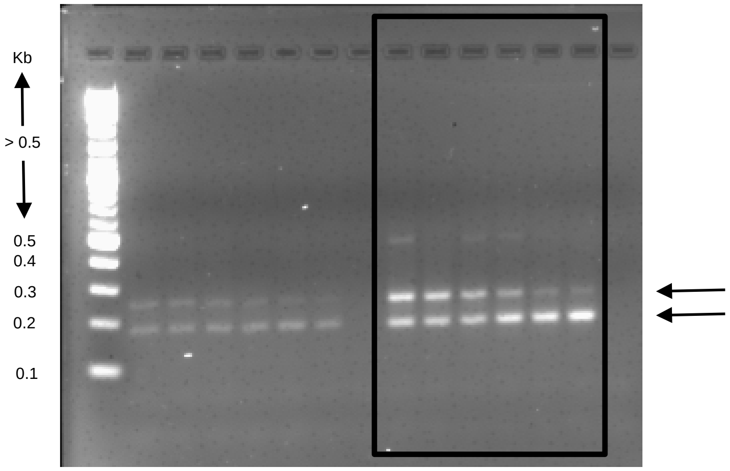

Full scans to accompany Figure 4C

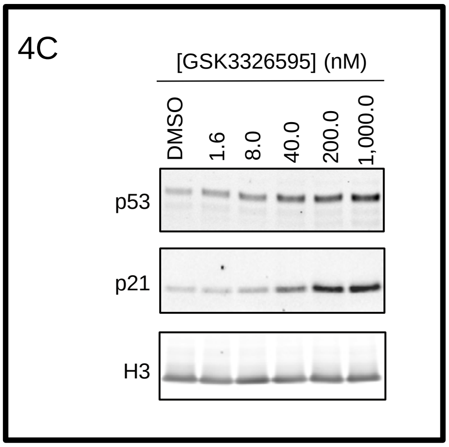

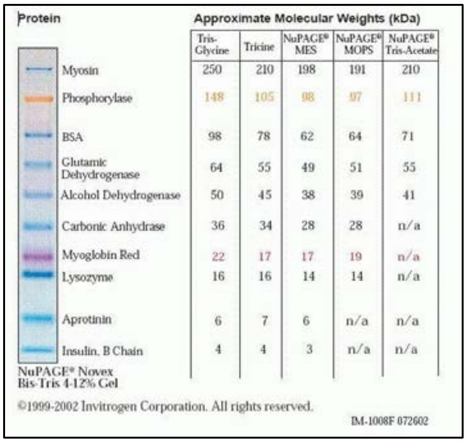

Protein	Tris-Glycine	Tricine	NuPAGE® MES	NuPAGE® MOPS	NuPAGE® Tris-Acetate
Myosin	250	210	198	191	210
Phosphorylase	148	105	98	97	111
BSA	98	78	62	64	71
Glutamic Dehydrogenase	64	55	49	51	55
Alcohol Dehydrogenase	50	45	38	39	41
Carbonic Anhydrase	36	34	28	28	n/a
Myoglobin Red	22	17	17	19	n/a
Lysozyme	16	16	14	14	n/a
Aprotinin	6	7	6	n/a	n/a
Insulin, B Chain	4	4	3	n/a	n/a

Approximate Molecular Weights (kDa)

NuPAGE® Novex Bis-Tris 4-12% Gel

©1999-2002 Invitrogen Corporation. All rights reserved.

IM-1008F 072602

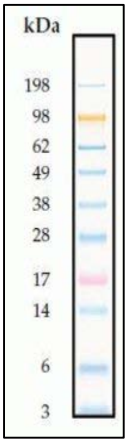

SeeBlue Plus2 Pre-stained Protein Standard
(Invitrogen #LC5925)

p53

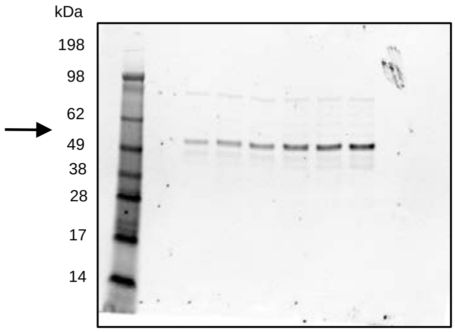

p21

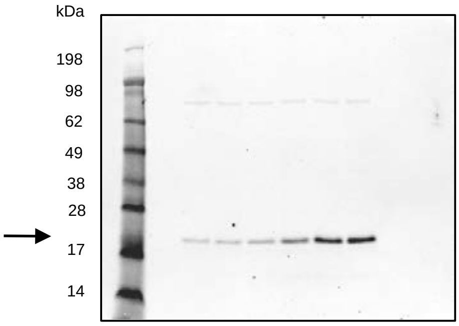

H3

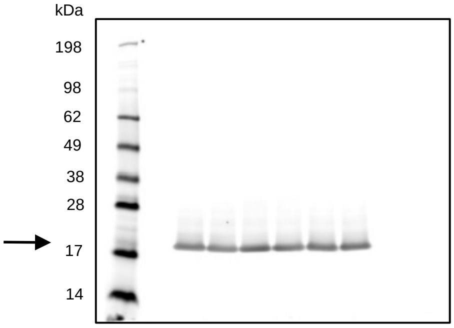

Full Scans to accompany Supplementary Figure S4A

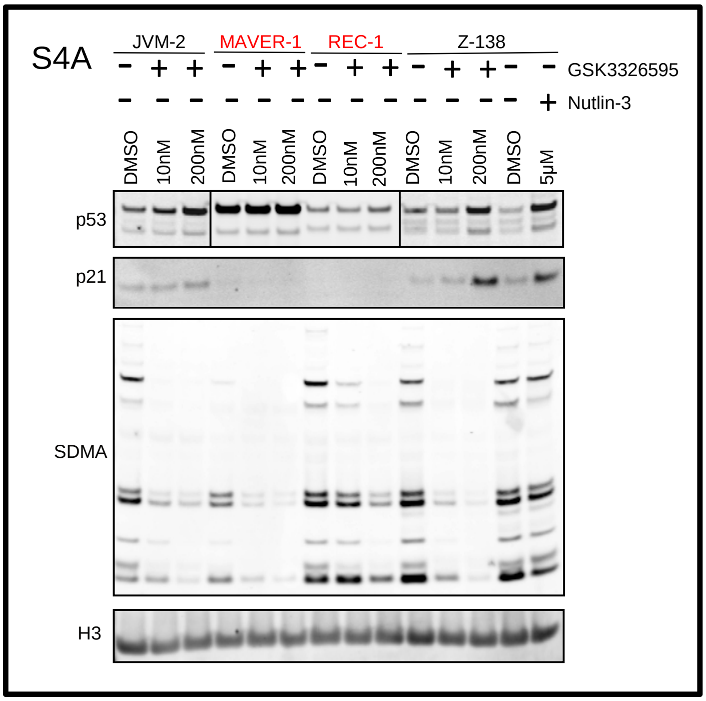

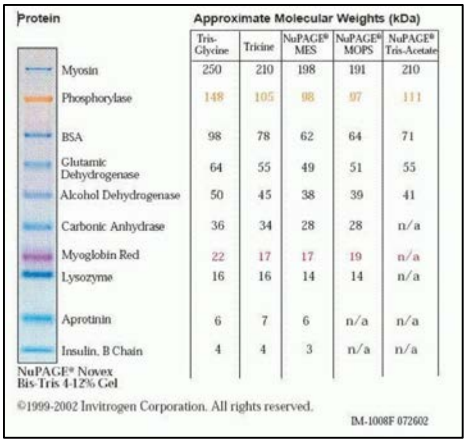

Protein	Approximate Molecular Weights (kDa) Tris-Glycine	Tricine	NuPAGE® MES	NuPAGE® MOPS	NuPAGE® Tris-Acetate
Myosin	250	210	198	191	210
Phosphorylase	148	105	98	97	111
BSA	98	78	62	64	71
Glutamic Dehydrogenase	64	55	49	51	55
Alcohol Dehydrogenase	50	45	38	39	41
Carbonic Anhydrase	36	34	28	28	n/a
Myoglobin Red	22	17	17	19	n/a
Lysozyme	16	16	14	14	n/a
Aprotinin	6	7	6	n/a	n/a
Insulin, B Chain	4	4	3	n/a	n/a

NuPAGE® Novex Bis-Tris 4-12% Gel

©1999-2002 Invitrogen Corporation. All rights reserved.

IM-1008F 072602

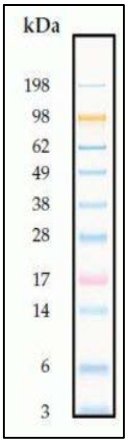

SeeBlue Plus2 Pre-stained Protein Standard (Invitrogen #LC5925)

p53 (low exposure)

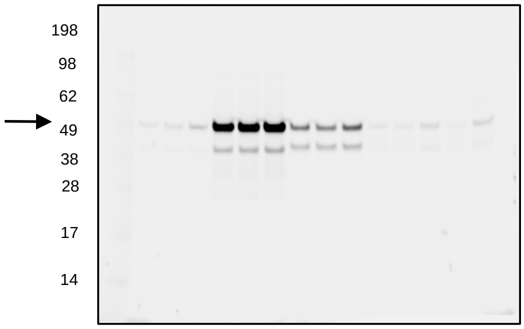

p53 (high exposure)

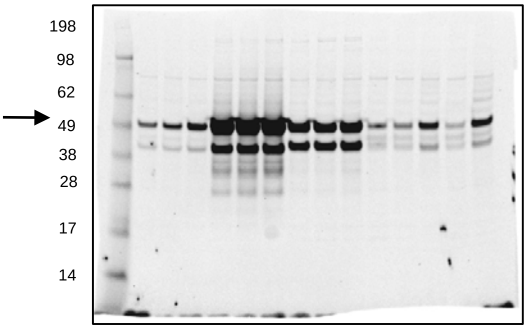

p21

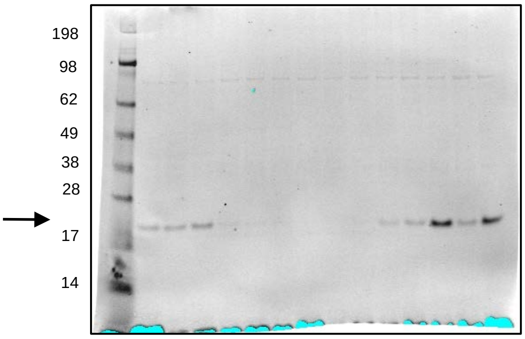

SDMA

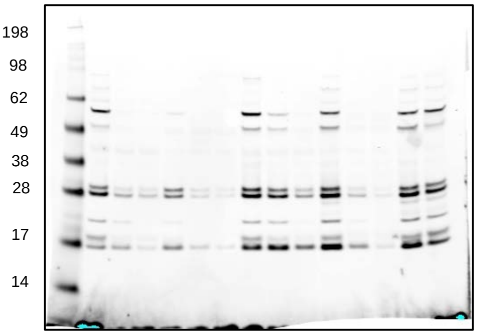

H3

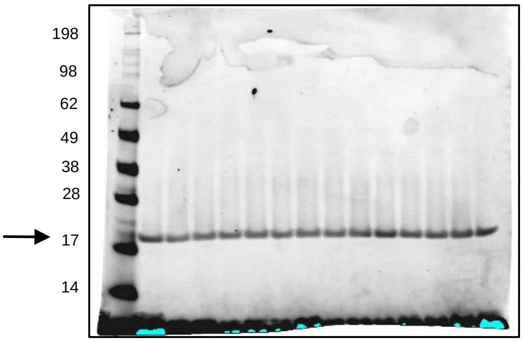

Full scans to accompany Figure 5B

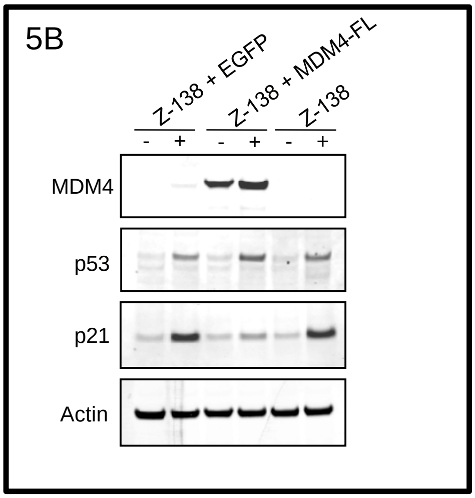

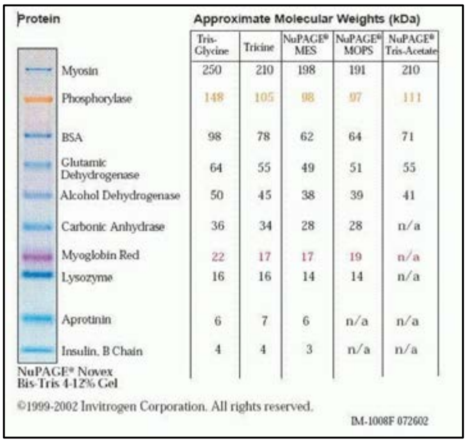

Protein	Tris-Glycine	Tricine	NuPAGE® MES	NuPAGE® MOPS	NuPAGE® Tris-Acetate
Myosin	250	210	198	191	210
Phosphorylase	148	105	98	97	111
BSA	98	78	62	64	71
Glutamic Dehydrogenase	64	55	49	51	55
Alcohol Dehydrogenase	50	45	38	39	41
Carbonic Anhydrase	36	34	28	28	n/a
Myoglobin Red	22	17	17	19	n/a
Lysozyme	16	16	14	14	n/a
Aprotinin	6	7	6	n/a	n/a
Insulin, B Chain	4	4	3	n/a	n/a

Approximate Molecular Weights (kDa)

NuPAGE® Novex Bis-Tris 4-12% Gel

©1999-2002 Invitrogen Corporation. All rights reserved.

IM-1008F 072602

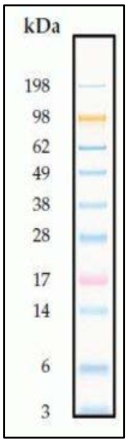

SeeBlue Plus2 Pre-stained Protein Standard
(Invitrogen #LC5925)

MDM4

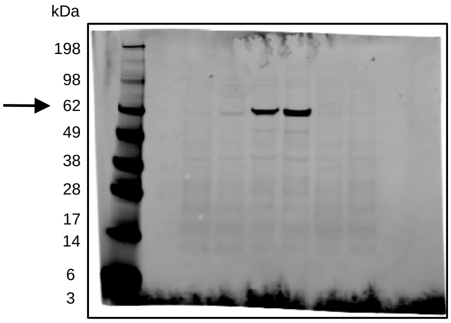

p53

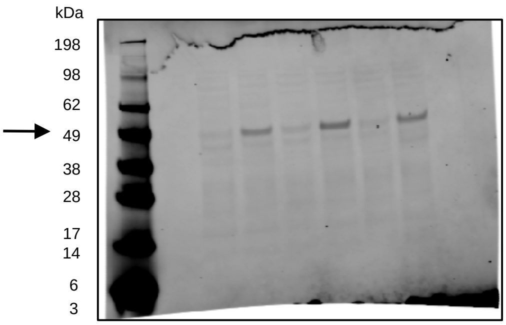

p21

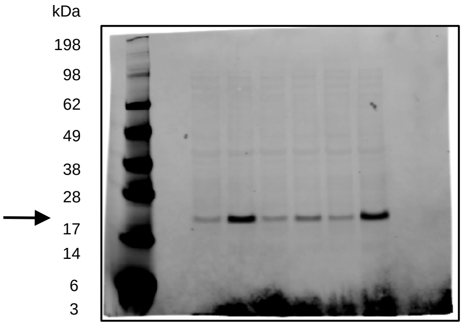

Actin

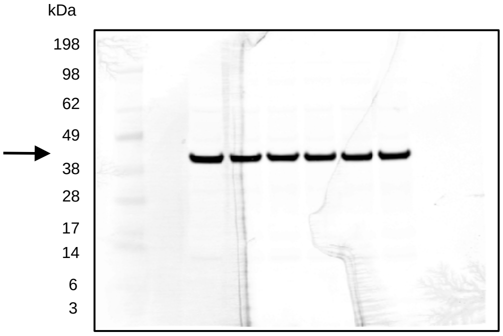

Full scans to accompany Figure 5E

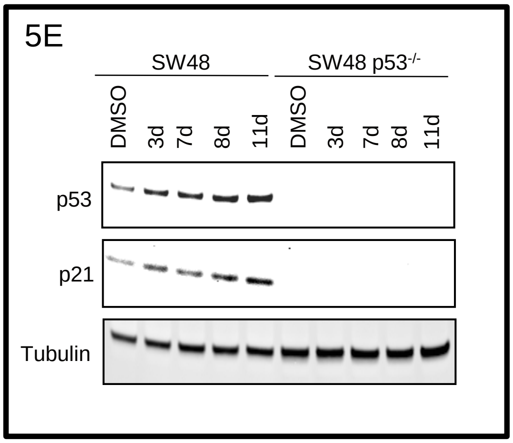

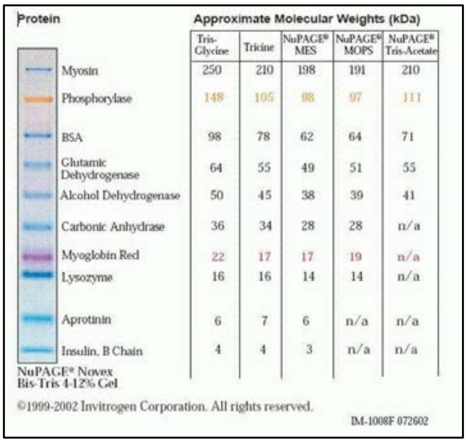

Protein	Approximate Molecular Weights (kDa)				
	Tris-Glycine	Tricine	NuPAGE® MES	NuPAGE® MOPS	NuPAGE® Tris-Acetate
Myosin	250	210	198	191	210
Phosphorylase	148	105	98	97	111
BSA	98	78	62	64	71
Glutamic Dehydrogenase	64	55	49	51	55
Alcohol Dehydrogenase	50	45	38	39	41
Carbonic Anhydrase	36	34	28	28	n/a
Myoglobin Red	22	17	17	19	n/a
Lysozyme	16	16	14	14	n/a
Aprotinin	6	7	6	n/a	n/a
Insulin, B Chain	4	4	3	n/a	n/a

NuPAGE® Novex Bis-Tris 4-12% Gel

©1999-2002 Invitrogen Corporation. All rights reserved.

IM-1008F 072602

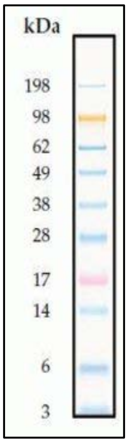

SeeBlue Plus2 Pre-stained Protein Standard (Invitrogen #LC5925)

p53

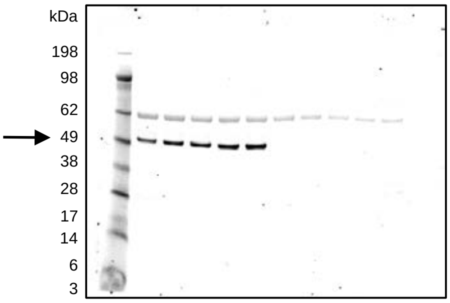

p21

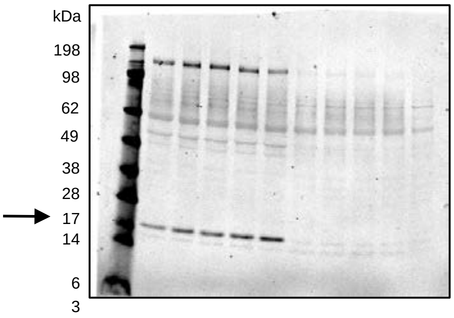

Tubulin

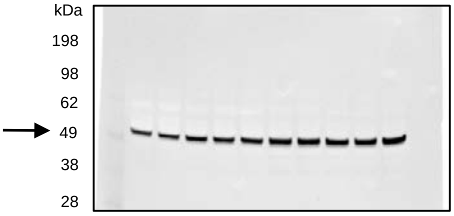

Full Scans to accompany Supplementary Figure S5C

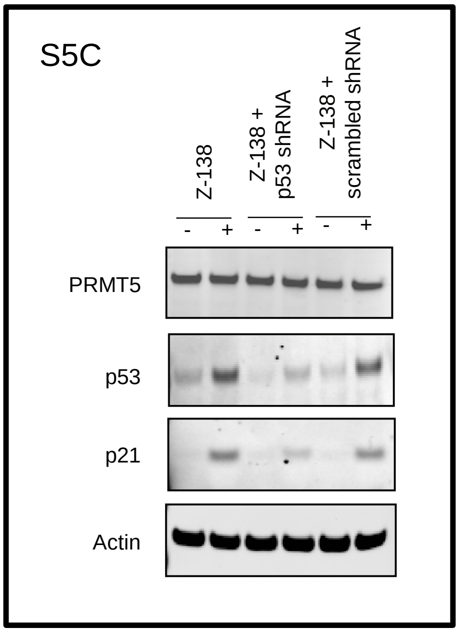

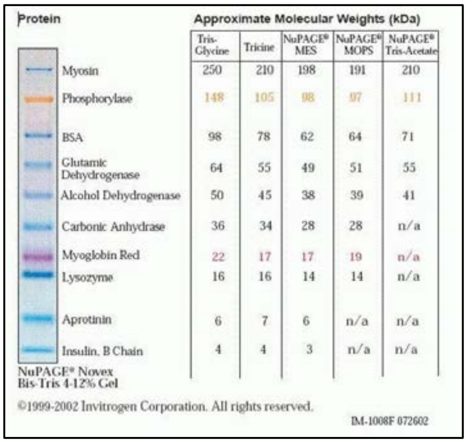

Protein	Tris-Glycine	Tricine	NuPAGE® MES	NuPAGE® MOPS	NuPAGE® Tris-Acetate
	Approximate Molecular Weights (kDa)				
Myosin	250	210	198	191	210
Phosphorylase	148	105	98	97	111
BSA	98	78	62	64	71
Glutamic Dehydrogenase	64	55	49	51	55
Alcohol Dehydrogenase	50	45	38	39	41
Carbonic Anhydrase	36	34	28	28	n/a
Myoglobin Red	22	17	17	19	n/a
Lysozyme	16	16	14	14	n/a
Aprotinin	6	7	6	n/a	n/a
Insulin, B Chain	4	4	3	n/a	n/a

NuPAGE® Novex Bis-Tris 4-12% Gel

©1999-2002 Invitrogen Corporation. All rights reserved.

IM-1008F 072602

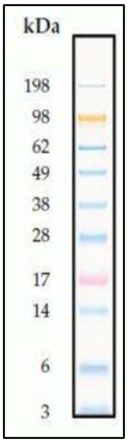

SeeBlue Plus2 Pre-stained Protein Standard
(Invitrogen #LC5925)

PRMT5

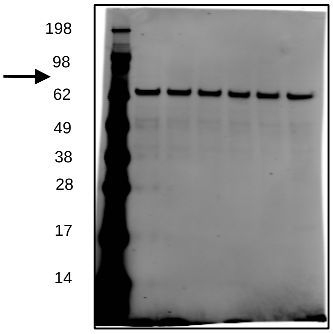

p53

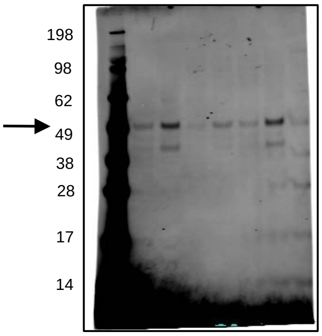

p21

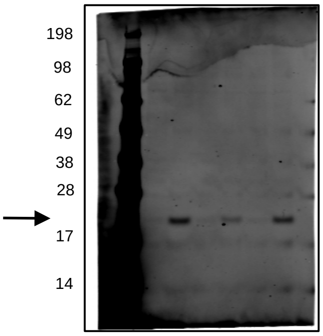

Actin

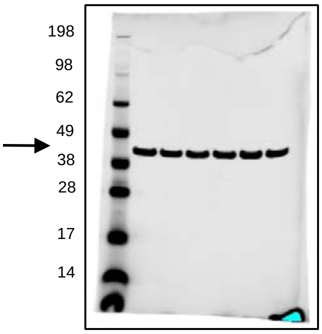

Full scans to accompany Figure 6F

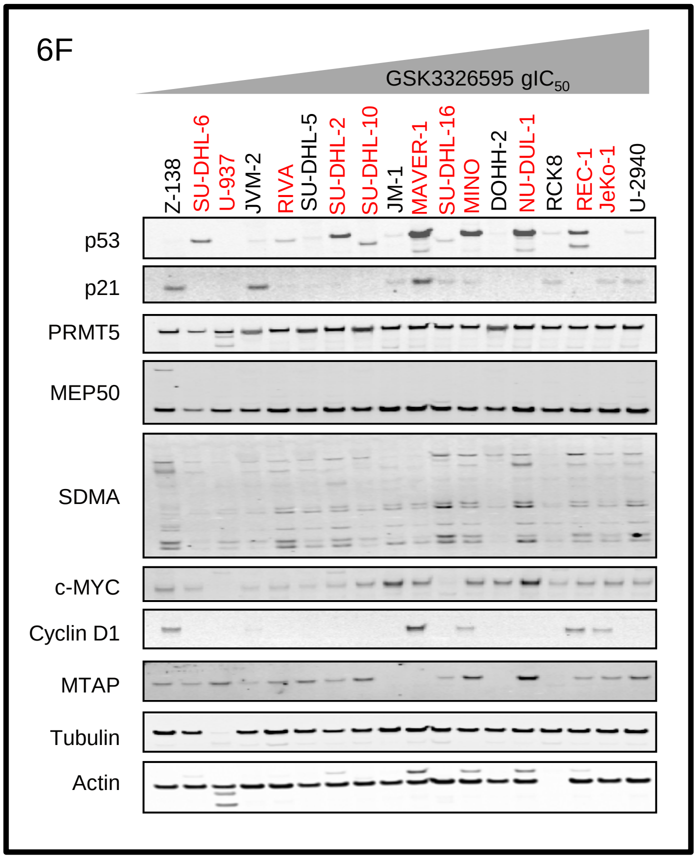

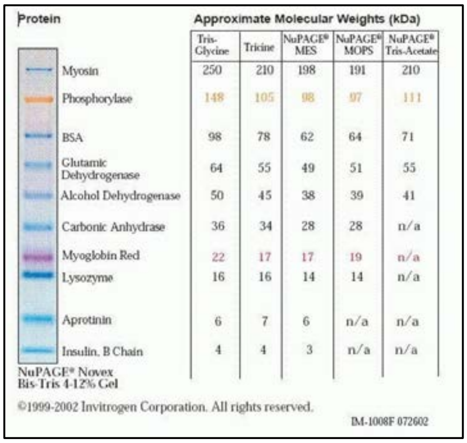

Protein	Approximate Molecular Weights (kDa)				
	Tris-Glycine	Tricine	NuPAGE® MES	NuPAGE® MOPS	NuPAGE® Tris-Acetate
Myosin	250	210	198	191	210
Phosphorylase	148	105	98	97	111
BSA	98	78	62	64	71
Glutamic Dehydrogenase	64	55	49	51	55
Alcohol Dehydrogenase	50	45	38	39	41
Carbonic Anhydrase	36	34	28	28	n/a
Myoglobin Red	22	17	17	19	n/a
Lysozyme	16	16	14	14	n/a
Aprotinin	6	7	6	n/a	n/a
Insulin, B Chain	4	4	3	n/a	n/a

NuPAGE® Novex Bis-Tris 4-12% Gel

©1999-2002 Invitrogen Corporation. All rights reserved.

IM-1008F 072602

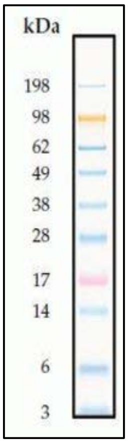

SeeBlue Plus2 Pre-stained Protein Standard (Invitrogen #LC5925)

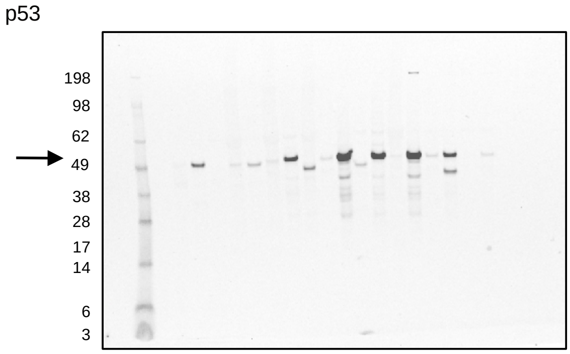

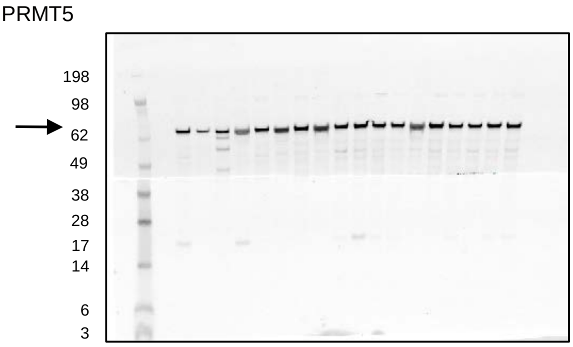

Blot was cut between molecular weight markers 38kDa and 49kDa. Top portion was probed with PRMT5 antibody and bottom portion was probed with p21 antibody.

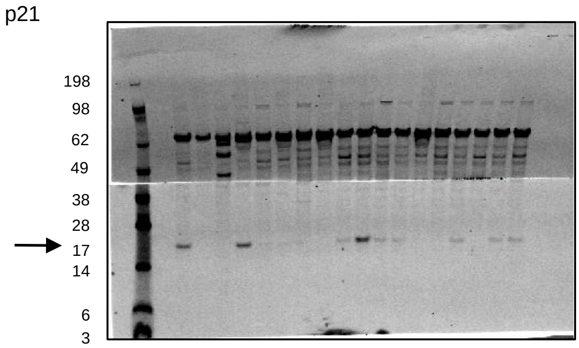

Blot was cut between molecular weight markers 38kDa and 49kDa. Top portion was probed with PRMT5 antibody and bottom portion was probed with p21 antibody.

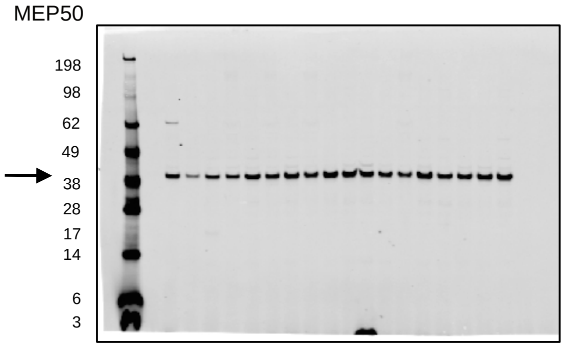

Full scans to accompany Figure 6F (continued)

SDMA

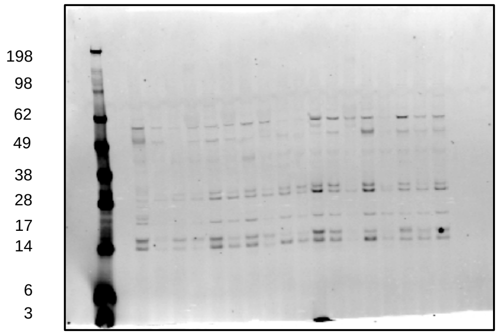

MTAP

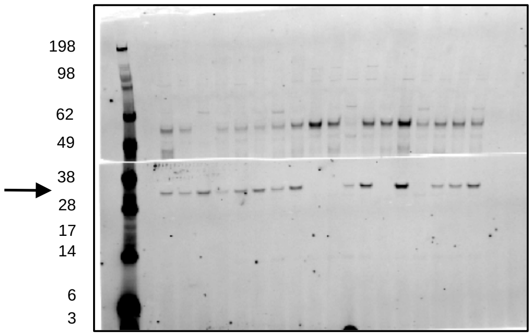

Blot was cut between molecular weight markers 38kDa and 49kDa. Top portion was probed with c-MYC antibody and bottom portion was probed with MTAP antibody.

c-MYC

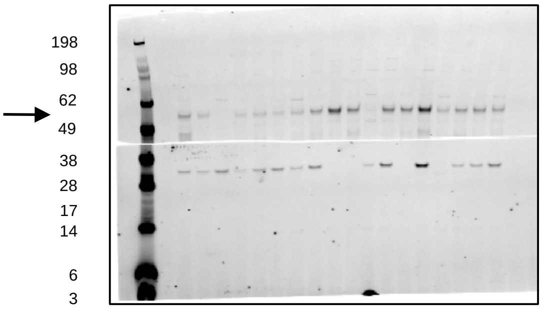

Blot was cut between molecular weight markers 38kDa and 49kDa. Top portion was probed with c-MYC antibody and bottom portion was probed with MTAP antibody.

Tubulin

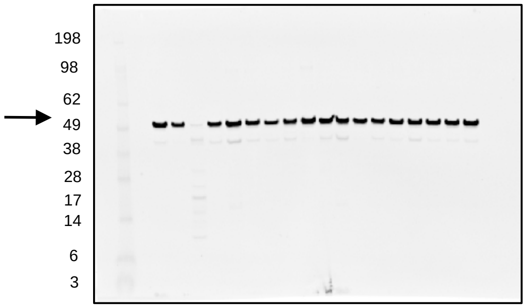

Cyclin D1

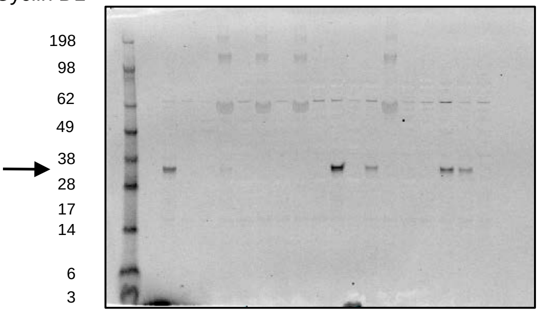

Actin

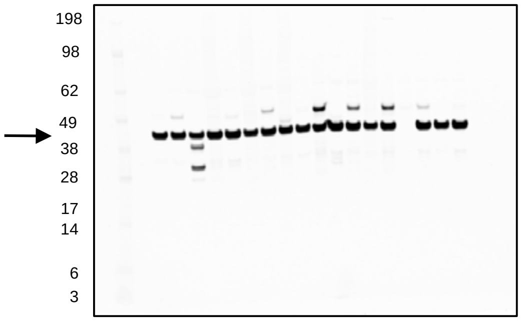

Supplementary Table 1: 6-day Proliferation Assay Data for GSK3203591 and GSK3326595

GSK3203591 6d Proliferation Data

Cell Line	Average of Pop Doubling	Average of gIC50 (nM)	Average of gIC100 (nM)	Average of Ymin-T0 (%)	Average of dEC50 (nM)	Sum of n=	StdDev of gIC50 (nM)	StdDev of gIC100 (nM)	StdDev of Ymin-T0 (%)	StdDev of dEC50 (nM)	Tumor Subtype	General tumor type
NKM-1	2.2	7.6	88.5	-78.6	351.1	3.0	3.2	56.3	5.4	252.6	AML	AML
GDM-1	3.9	42.3	30791.8	219.2	30791.8	5.0	13.1	3278.7	83.8	3278.7	AML	AML
NB-4	5.0	80.9	998.7	-94.5	1591.2	3.0	34.0	259.8	7.8	448.3	AML	AML
MOLM-13	6.7	86.2	25325.8	92.0	30843.8	13.0	33.8	11722.4	168.8	8493.3	AML	AML
MV-4-11	5.7	94.7	14938.6	25.3	25302.0	11.0	43.4	13156.4	198.6	10993.6	AML	AML
SKM-1	4.2	109.8	914.7	-56.0	10061.4	4.0	62.4	461.2	8.3	12887.8	AML	AML
ML-2	5.1	115.4	36656.9	124.1	36656.9	6.0	69.7	0.0	97.2	0.0	AML	AML
OCI-M1	7.1	130.1	29325.5	295.1	29325.5	3.0	100.3	0.0	382.4	0.0	AML	AML
TF-1	4.7	137.5	29325.5	529.6	29325.5	2.0	70.1	0.0	196.8	0.0	AML	AML
MonoMac6	3.6	196.3	22211.6	54.0	22498.7	4.0	180.7	14227.9	92.5	13653.6	AML	AML
OCI-AML5	6.4	297.4	29325.5	410.5	29325.5	6.0	263.8	0.0	169.8	0.0	AML	AML
SIG-M5	6.8	315.7	15150.6	17.8	22126.4	14.0	393.0	15175.8	107.9	14883.4	AML	AML
PL-21	5.6	329.2	36656.9	1042.6	36656.9	2.0	163.1	0.0	170.8	0.0	AML	AML
SHI-1	6.1	358.3	36656.9	382.7	36656.9	4.0	110.0	0.0	195.1	0.0	AML	AML
OCI-AML3	5.6	372.7	36656.9	683.3	36656.9	4.0	233.1	0.0	109.1	0.0	AML	AML
KO52	2.8	512.9	29325.5	117.1	29325.5	3.0	179.1	0.0	68.5	0.0	AML	AML
SKNO-1	3.1	563.2	36656.9	189.9	36656.9	2.0	241.1	0.0	1.6	0.0	AML	AML
AML-193	2.0	1397.2	24595.6	17.3	29325.5	4.0	787.1	9481.3	42.7	0.0	AML	AML
F-36P	4.2	1879.9	29325.5	529.3	29325.5	3.0	2600.9	0.0	143.2	0.0	AML	AML
ME1	1.8	2450.6	18761.8	-6.7	29325.5	2.0	2415.5	14939.4	44.0	0.0	AML	AML
HL60	5.6	4267.9	31769.3	1529.5	31769.3	6.0	5507.1	3785.9	840.8	3785.9	AML	AML
NOMO-1	3.7	7322.5	34824.1	435.2	34824.1	8.0	12678.0	3393.8	239.9	3393.8	AML	AML
KASUMI-1	2.7	13217.1	32074.8	227.3	32074.8	8.0	15552.4	7776.1	123.1	7776.1	AML	AML
MOLM-16	1.6	31016.6	35190.6	324.0	35190.6	5.0	8405.8	3278.7	93.7	3278.7	AML	AML
GF-D8	3.2	33199.1	36656.9	484.7	36656.9	6.0	8469.8	0.0	190.1	0.0	AML	AML
5637	4.3	143.1	10971.7	20.0	17043.8	8.0	73.8	8676.8	161.7	7823.4	Bladder	Bladder
SCaBER	5.0	255.1	5238.0	-65.7	10720.5	8.0	134.0	3995.2	26.2	8765.0	Bladder	Bladder
BC-3C	5.0	407.5	17601.8	410.5	17601.8	10.0	266.3	6178.9	155.9	6178.9	Bladder	Bladder
647-V	3.8	426.3	14669.7	290.5	14669.7	7.0	120.3	8.6	107.2	8.6	Bladder	Bladder
BFTC-905	4.9	439.6	14670.9	190.3	14670.9	8.0	61.1	8.6	120.7	8.6	Bladder	Bladder
SW-780	5.0	450.5	17601.8	222.8	17601.8	10.0	255.9	6178.9	130.9	6178.9	Bladder	Bladder
JMSU-1	5.3	583.2	17601.8	297.1	17601.8	10.0	263.8	6178.9	112.4	6178.9	Bladder	Bladder
T24	5.3	649.6	17601.8	691.4	17601.8	10.0	376.7	6178.9	96.7	6178.9	Bladder	Bladder
639-V	5.7	731.1	14670.9	926.0	14670.9	8.0	219.3	8.6	213.5	8.6	Bladder	Bladder
CAL-29	4.5	817.3	14572.3	54.0	14672.0	7.0	239.7	260.9	54.3	8.6	Bladder	Bladder
RT-112	5.7	1105.6	20527.9	1020.7	20527.9	5.0	610.9	8031.1	600.6	8031.1	Bladder	Bladder
KU-19-19	6.2	1114.4	14662.8	1485.8	14662.8	4.0	40.1	0.0	221.8	0.0	Bladder	Bladder
VM-CUB1	6.5	1579.1	19550.4	1575.5	19550.4	6.0	373.1	7571.8	420.0	7571.8	Bladder	Bladder
SW-1710	3.9	10024.0	19550.4	720.0	19550.4	6.0	7186.5	7571.8	52.3	7571.8	Bladder	Bladder
TCCSUP	3.5	11249.1	14662.8	504.8	14662.8	2.0	4827.7	0.0	149.3	0.0	Bladder	Bladder
HT-1376	2.3	13130.0	17595.3	200.7	17595.3	5.0	10636.9	6557.4	38.3	6557.4	Bladder	Bladder
UM-UC-3	4.8	19168.6	20946.8	1745.4	20946.8	7.0	8652.7	7837.5	1432.8	7837.5	Bladder	Bladder
J82	0.5	20946.8	20946.8	1482.0	20946.8	7.0	7837.5	7837.5	1346.4	7837.5	Bladder	Bladder
SF539	3.5	75.0	1482.7	-53.8	16948.8	2.0	15.4	692.7	9.7	17503.4	GBM	Brain
KS-1	3.7	85.5	29325.5	73.5	29325.5	2.0	14.2	0.0	69.7	0.0	GBM	Brain
U-87MG	3.8	127.5	29325.5	270.3	29325.5	4.0	124.3	0.0	122.4	0.0	GBM	Brain
YKG1	5.7	254.5	29325.5	959.3	29325.5	2.0	64.8	0.0	72.3	0.0	GBM	Brain
SF-126	3.5	385.8	29325.5	291.0	29325.5	2.0	127.3	0.0	61.2	0.0	GBM	Brain
A172	3.8	590.4	29325.5	555.1	29325.5	4.0	432.5	0.0	245.4	0.0	GBM	Brain
LN-229	4.1	1149.7	29325.5	578.8	29325.5	4.0	1565.1	0.0	160.4	0.0	GBM	Brain
T98MG	5.1	1296.2	29325.5	671.2	29325.5	2.0	634.0	0.0	25.2	0.0	GBM	Brain
U251MG	4.5	3490.9	29325.5	515.1	29325.5	5.0	2298.6	0.0	248.6	0.0	GBM	Brain
M059K	3.2	7887.2	29325.5	247.3	29325.5	2.0	1516.8	0.0	24.2	0.0	GBM	Brain
LN-18	3.5	9683.6	19615.1	314.6	29325.5	4.0	13132.8	12099.9	405.8	0.0	GBM	Brain
SF295	3.3	9842.8	29325.5	341.4	29325.5	2.0	10839.2	0.0	88.9	0.0	GBM	Brain
M059J	3.3	18934.3	29325.5	426.9	29325.5	2.0	14695.4	0.0	217.4	0.0	GBM	Brain
DBTRG05MG	3.6	29325.5	29325.5	610.3	29325.5	2.0	0.0	0.0	115.3	0.0	GBM	Brain
U138MG	2.7	29325.5	29325.5	369.0	29325.5	2.0	0.0	0.0	34.7	0.0	GBM	Brain
YH13	2.5	29325.5	29325.5	377.1	29325.5	2.0	0.0	0.0	20.9	0.0	GBM	Brain
HCC38	2.6	127.1	22842.0	17.5	34213.1	3.0	48.0	17957.1	25.3	4232.8	Breast	Breast
SK-BR-3	3.2	131.5	19074.3	2.1	30188.0	17.0	122.0	13143.1	36.9	2434.8	Breast	Breast
EFM-19	2.5	162.3	24437.9	44.0	24437.9	9.0	58.9	9698.5	28.8	9698.5	Breast	Breast
MCF-7	3.4	214.9	21375.0	82.4	28103.6	18.0	329.1	12888.3	120.4	8050.8	Breast	Breast
T-47D	3.0	225.0	21994.2	164.9	21994.2	4.0	118.3	16931.1	61.2	16931.1	Breast	Breast
HCC1806	4.7	260.9	11389.3	-32.8	26464.5	5.0	136.5	6412.9	31.7	10965.3	Breast	Breast
MDA-MB-453	3.2	343.0	28826.6	105.2	30954.7	9.0	387.5	7681.9	68.1	3232.8	Breast	Breast
HCC1143	2.8	509.3	30372.8	145.1	30372.8	7.0	260.1	2771.0	54.5	2771.0	Breast	Breast
HCC70	2.3	522.1	14489.6	13.6	27995.5	11.0	498.3	12679.8	43.4	7190.9	Breast	Breast
MDA-MB-468	2.9	585.0	12342.5	37.7	18606.7	16.0	705.6	11549.1	96.4	10895.1	Breast	Breast
HCC1937	1.8	956.5	14670.9	72.8	14678.9	6.0	521.0	8.8	32.9	0.0	Breast	Breast
DU-4475	3.9	1014.4	19561.1	187.0	19561.1	6.0	510.4	7563.5	111.1	7563.5	Breast	Breast
CAL-85-1	2.2	1741.7	14662.8	111.5	14678.9	3.0	854.6	0.0	45.1	0.0	Breast	Breast
CAL-51	5.9	2303.8	25943.0	1432.3	25945.5	13.0	4139.9	6427.7	665.3	6423.0	Breast	Breast
ZR-75-1	2.2	2615.1	30241.9	93.4	30241.9	8.0	4669.4	2592.0	57.3	2592.0	Breast	Breast
MDA-MB-231	3.9	2928.2	30453.4	404.1	30453.4	13.0	1894.0	2753.2	93.3	2753.2	Breast	Breast
MDA-MB-157	1.8	3250.0	33001.3	35.5	32991.2	4.0	2175.4	4244.5	12.3	4232.8	Breast	Breast
KPL-1	3.0	4113.1	29325.5	227.7	29325.5	5.0	3428.0	0.0	28.0	0.0	Breast	Breast
BT-474	1.7	5741.0	7331.4	110.8	7331.4	2.0	2249.2	0.0	32.7	0.0	Breast	Breast
MDA-MB-436	3.3	7132.0	20527.9	387.5	20537.5	5.0	4518.7	8031.1	59.3	8022.3	Breast	Breast
CAMA-1	2.0	7331.4	7331.4	202.0	7331.4	2.0	0.0	0.0	97.4	0.0	Breast	Breast
BT-20	2.2	8162.9	14669.2	178.8	14678.9	5.0	6314.0	8.8	84.6	0.0	Breast	Breast
HCC1569	2.5	11341.2	29325.5	218.5	29325.5	8.0	14894.6	0.0	83.2	0.0	Breast	Breast
HCC1395	1.4	14678.9	14662.8	95.0	14678.9	3.0	0.0	0.0	31.2	0.0	Breast	Breast
BT549	3.0	17803.2	24095.7	477.6	24095.7	7.0	8050.5	9182.1	207.3	9182.1	Breast	Breast
Hs578t	2.9	18240.7	26188.1	393.1	26188.1	7.0	11467.1	8305.5	111.8	8305.5	Breast	Breast
WiDr	5.8	438.3	29325.5	509.1	29325.5	2.0	18.0	0.0	33.6	0.0	Colon	Colon
Ls411N	5.3	442.3	29325.5	493.0	29325.5	2.0	91.1	0.0	91.4	0.0	Colon	Colon
SK-CO-1	2.8	473.1	29325.5	34.9	29325.5	3.0	102.2	0.0	35.1	0.0	Colon	Colon
LoVo	5.0	494.3	29325.5	669.1	29325.5	3.0	182.2	0.0	138.1	0.0	Colon	Colon
COLO 201	4.3	661.3	11107.7	360.1	14662.8	7.0	659.1	6094.4	469.9	0.0	Colon	Colon
COLO 205	5.5	693.7	24437.9	633.0	24437.9	3.0	210.9	8465.5	467.0	8465.5	Colon	Colon
CaCo-2	4.2	739.8	29325.5	214.6	29325.5	2.0	37.3	0.0	96.2	0.0	Colon	Colon
HCT-15	6.5	1248.3	29325.5	1571.1	29325.5	2.0	237.0	0.0	740.8	0.0	Colon	Colon
SW620	5.8	1352.1	29325.5	1640.5	29325.5	2.0	671.8	0.0	250.9	0.0	Colon	Colon
NCIH747	2.4	1499.1	22002.2	122.6	22002.2	4.0	406.2	8456.2	26.7	8456.2	Colon	Colon
SNUC1	4.1	1517.6	16766.7	999.4	16766.7	7.0	1175.0	5537.9	1544.3	5537.9	Colon	Colon
T84	2.5	1682.9	23253.9	-8.1	29325.5	3.0	792.6	7112.5	26.7	0.0	Colon	Colon
HT115	5.2	1742.1	29325.5	875.3	29325.5	2.0	737.7	0.0	326.0	0.0	Colon	Colon
HT-29	5.0	1812.3	16495.6	398.3	16495.6	8.0	1937.7	5184.0	131.6	5184.0	Colon	Colon
RKO	5.4	2208.3	14678.9	1047.3	14678.9	4.0	1600.6	0.0	489.6	0.0	Colon	Colon
COLO 320 HSR	5.4	2222.9	8842.4	1872.5	12999.3	4.0	2511.9	6784.6	2309.2	3326.9	Colon	Colon
SW480	4.8	2260.6	22002.2	814.3	22002.2	8.0	2687.8	7828.9	442.7	7828.9	Colon	Colon
COLO-320DM	6.4	2347.1	29325.5	2248.6	29325.5	2.0	317.9	0.0	31.1	0.0	Colon	Colon
DLD-1	6.3	2434.1	29325.5	1933.3	29325.5	5.0	1934.0	0.0	441.0	0.0	Colon	Colon
SW837	3.6	9145.2	14678.9	707.3	14678.9	6.0	4708.6	0.0	593.6	0.0	Colon	Colon
SW48	3.2	9337.4	14678.9	444.1	14678.9	3.0	7132.0	0.0	271.6	0.0	Colon	Colon
SW403	4.0	9764.1	14678.9	2368.4	14678.9	5.0	4650.1	0.0	2998.6	0.0	Colon	Colon
HCT-116	5.8	10047.2	26393.0	2309.7	26393.0	5.0	12130.0	6557.4	1298.2	6557.4	Colon	Colon
SW-1417	1.7	19615.4	29325.5	102.6	29325.5	2.0	8158.9	0.0	3.1	0.0	Colon	Colon
NCI-H520	4.4	26.1	29325.5	212.9	29325.5	4.0	17.4	0.0	27.5	0.0	Lung	Lung
LK2	6.0	89.9	29325.5	129.4	29325.5	2.0	8.6	0.0	21.2	0.0	Lung	Lung
PC9	4.5	95.0	22681.0	36.6	29325.5	3.0	44.6	11508.6	45.9	0.0	Lung	Lung
NCI-H526	3.2	138.2	7421.3	-35.0	28222.5	5.0	75.5	3759.8	13.2	2466.3	Lung	Lung
HCC4006	3.0	170.4	29325.5	29.4	29325.5	2.0	77.3	0.0	10.8	0.0	Lung	Lung
NCI-H526-R	3.6	218.8	4224.4	-64.6	14595.0	4.0	75.1	1248.0	14.3	5287.7	Lung	Lung
EBC-1	4.5	238.6	29325.5	297.5	29325.5	2.0	113.9	0.0	48.6	0.0	Lung	Lung
NCI-H1048	2.4	252.4	3534.1	-43.4	10888.9	2.0	190.4	2753.3	27.6	5337.1	Lung	Lung
DMS273	6.1	261.5	29325.5	2159.0	29325.5	2.0	36.2	0.0	1005.1	0.0	Lung	Lung
A427	2.5	276.1	22215.4	25.6	26393.0	5.0	334.1	9981.6	31.6	6557.4	Lung	Lung
NCI-H1975	5.2	289.1	29325.5	526.2	29325.5	2.0	67.5	0.0	42.4	0.0	Lung	Lung
NCI-H146	2.7	411.2	14662.8	136.5	14662.8	2.0	353.1	0.0	72.3	0.0	Lung	Lung
NCI-H1299	5.4	418.9	29325.5	486.2	29325.5	2.0	125.7	0.0	56.1	0.0	Lung	Lung
LU65	3.2	463.3	29325.5	355.5	29325.5	2.0	475.6	0.0	472.1	0.0	Lung	Lung
NCI-H1437	4.7	467.0	29325.5	364.3	29325.5	4.0	435.4	0.0	94.2	0.0	Lung	Lung
NCI-H2087	2.8	509.5	29325.5	75.7	29325.5	5.0	466.5	0.0	46.9	0.0	Lung	Lung
COR-L23	4.4	786.2	29325.5	167.0	29325.5	4.0	394.1	0.0	90.2	0.0	Lung	Lung
A549	5.6	837.1	29325.5	1720.2	29325.5	11.0	724.8	0.0	1007.2	0.0	Lung	Lung
SK-LU-1	2.9	881.1	29325.5	215.1	29325.5	2.0	119.5	0.0	69.2	0.0	Lung	Lung
NCI-H1915	5.3	1132.6	29325.5	1397.0	29325.5	2.0	347.9	0.0	903.1	0.0	Lung	Lung
Calu6	2.6	1275.9	22655.5	37.7	29325.5	3.0	713.3	11552.8	63.1	0.0	Lung	Lung
NCI-H292	5.9	1343.1	29325.5	1798.7	29325.5	2.0	1080.2	0.0	73.0	0.0	Lung	Lung
NCI-H322	3.9	1700.0	29325.5	447.4	29325.5	3.0	156.5	0.0	85.4	0.0	Lung	Lung
NCI-H647	2.7	2529.3	29325.5	201.2	29325.5	3.0	1290.4	0.0	45.4	0.0	Lung	Lung
Cha-Go-K1	2.6	2823.9	29325.5	86.8	29325.5	2.0	1061.7	0.0	107.9	0.0	Lung	Lung
SW1271	3.7	3180.2	29325.5	350.4	29325.5	5.0	2431.2	0.0	137.5	0.0	Lung	Lung
LU-99	3.5	5303.8	29325.5	409.1	29325.5	6.0	11775.3	0.0	270.7	0.0	Lung	Lung
NCI-H2170	2.3	6386.6	29325.5	136.0	29325.5	3.0	1665.2	0.0	72.5	0.0	Lung	Lung
LC-1sq	2.1	7594.2	29325.5	79.1	29325.5	5.0	9224.2	0.0	14.4	0.0	Lung	Lung
SW900	2.7	7967.8	29325.5	293.7	29325.5	5.0	12560.9	0.0	227.9	0.0	Lung	Lung
NCI-H1650	2.7	8766.6	29325.5	232.3	29325.5	5.0	11701.7	0.0	70.5	0.0	Lung	Lung
DMS114	3.5	9165.2	32991.2	340.2	32991.2	2.0	9115.4	5184.1	83.3	5184.1	Lung	Lung
UMC11	2.1	10650.8	29325.5	178.6	29325.5	3.0	16178.0	0.0	52.9	0.0	Lung	Lung
SW1573	4.3	11378.8	29325.5	711.5	29325.5	5.0	12758.3	0.0	101.4	0.0	Lung	Lung
NCI-H1581	2.7	12818.6	29325.5	222.4	29325.5	2.0	4049.2	0.0	1.7	0.0	Lung	Lung
NCI-H1651	2.6	15260.6	29325.5	199.4	29325.5	2.0	19890.8	0.0	42.2	0.0	Lung	Lung
SK-MES-1	2.5	18549.1	29325.5	301.2	29325.5	2.0	15240.1	0.0	320.4	0.0	Lung	Lung
NCI-H1648	1.6	20744.0	29325.5	98.5	29325.5	2.0	12136.1	0.0	34.6	0.0	Lung	Lung
HCC827	3.5	20985.3	29325.5	493.7	29325.5	5.0	11420.3	0.0	280.3	0.0	Lung	Lung
HCC2935	1.9	21588.3	29325.5	133.2	29325.5	2.0	10942.1	0.0	91.2	0.0	Lung	Lung
NCI-H441	1.9	22361.0	26881.7	154.5	26881.7	3.0	18773.5	11198.8	15.9	11198.8	Lung	Lung
Calu1	3.1	29325.5	29325.5	673.6	29325.5	2.0	0.0	0.0	563.8	0.0	Lung	Lung
NCI-H1563	2.1	29325.5	29325.5	241.8	29325.5	2.0	0.0	0.0	30.8	0.0	Lung	Lung
NCI-H522	0.0	29325.5	29325.5	225.8	29325.5	2.0	0.0	0.0	79.4	0.0	Lung	Lung
COR-L105	2.1	29325.5	29325.5	213.4	29325.5	5.0	0.0	0.0	20.1	0.0	Lung	Lung
NCI-H661	2.0	30791.8	30791.8	832.5	30791.8	5.0	3278.7	3278.7	441.5	3278.7	Lung	Lung
SU-DHL-6	4.7	12.9	109.7	-99.0	201.8	3.0	15.6	76.9	1.7	145.2	DLBCL	Lymphoma
Z-138	4.4	13.6	161.1	-95.5	277.1	15.0	13.2	154.1	5.4	266.8	MCL	Lymphoma
OCI-LY-18	4.0	30.8	8089.4	-66.8	13257.8	3.0	9.2	8987.0	57.4	13915.7	DLBCL	Lymphoma
SR-786	5.1	32.8	29325.5	46.2	29325.5	2.0	7.1	0.0	24.3	0.0	TCL	Lymphoma
U937	7.5	48.6	17747.9	39.0	21782.8	8.0	42.7	12767.2	139.0	11434.1	DLBCL	Lymphoma
RIVA	3.3	68.4	1756.5	-67.8	7150.6	3.0	50.4	1289.0	12.7	4754.5	DLBCL	Lymphoma
WSU-NHL	5.3	75.7	1782.5	-100.0	2913.3	2.0	57.6	906.1	0.0	1331.6	DLBCL	Lymphoma
JM1	4.2	75.8	1776.3	-79.1	7039.4	2.0	29.6	1441.4	29.6	8116.5	DLBCL	Lymphoma
MC116	4.4	82.0	380.4	-93.7	561.9	7.0	27.0	176.3	7.3	307.5	BCL	Lymphoma
SU-DHL-2	5.4	86.0	12374.3	60.6	12784.7	5.0	95.6	15475.1	238.0	15101.7	DLBCL	Lymphoma
NU-DHL-1	5.3	100.6	871.1	-100.0	1186.3	2.0	62.4	412.5	0.0	552.3	DLBCL	Lymphoma
OCI-LY-19	5.3	101.0	3773.1	-75.5	8887.1	5.0	33.0	4042.6	26.5	11552.4	DLBCL	Lymphoma
HuT78	4.3	103.2	1461.2	-93.1	2779.8	2.0	27.5	626.4	2.1	966.8	TCL	Lymphoma
DOHH-2	4.8	115.6	2218.1	-90.4	4204.5	7.0	64.1	1676.1	10.4	3720.4	DLBCL	Lymphoma
SC-1	6.8	117.4	24437.9	566.8	24437.9	3.0	70.0	8465.5	296.9	8465.5	DLBCL	Lymphoma
SU-DHL-16	4.5	123.8	29325.5	327.3	29325.5	3.0	35.4	0.0	145.8	0.0	DLBCL	Lymphoma
SU-DHL-5	5.9	132.2	6260.5	-87.3	6724.0	8.0	160.8	9449.8	35.8	9299.5	DLBCL	Lymphoma
MAVER-1	4.1	132.6	9259.9	11.0	16862.2	8.0	30.5	9421.7	31.6	13369.9	MCL	Lymphoma
HD-MY-Z	5.8	142.8	29325.5	390.9	29325.5	2.0	84.8	0.0	254.8	0.0	Hodgkin's lymphoma	Lymphoma
JVM-2	3.4	171.9	9761.8	53.6	13232.0	10.0	136.8	11217.1	88.2	11744.6	MCL	Lymphoma
WSU-FSCCL	4.7	268.1	21156.2	77.3	24437.9	6.0	175.8	9135.3	147.1	7571.8	DLBCL	Lymphoma
HT	5.0	272.4	14675.4	9.6	17420.3	13.0	158.5	10519.0	68.0	11251.4	DLBCL	Lymphoma
1A2	5.4	332.6	29325.5	145.3	29325.5	4.0	133.9	0.0	127.4	0.0	BCL	Lymphoma
U2932	3.9	336.4	2566.6	-77.7	8122.4	10.0	253.1	1562.2	24.9	8492.3	DLBCL	Lymphoma
DB	5.3	346.5	21999.5	152.0	21999.5	12.0	291.8	7651.7	153.6	7651.7	DLBCL	Lymphoma
Pfeiffer	2.3	352.1	4764.9	-62.8	12475.4	6.0	269.3	6802.3	30.1	13100.6	DLBCL	Lymphoma
Toledo	4.5	443.4	23281.4	94.5	23460.4	5.0	270.6	8282.2	68.4	8031.1	DLBCL	Lymphoma
SU-DHL-10	8.3	494.9	21219.7	2378.0	21219.7	12.0	439.7	8621.0	3398.7	8621.0	DLBCL	Lymphoma
MINO	4.5	536.1	12182.5	-16.9	24826.9	5.0	410.0	10099.8	72.7	6932.0	MCL	Lymphoma
NU-DUL-1	3.6	548.1	11772.7	-23.9	29325.5	2.0	348.7	6813.8	4.6	0.0	DLBCL	Lymphoma
SU-DHL-4	6.3	571.4	24437.9	984.4	24437.9	6.0	238.8	7571.8	506.9	7571.8	DLBCL	Lymphoma
CA-46	6.3	580.1	29325.5	1212.6	29325.5	2.0	12.7	0.0	653.3	0.0	Burkitt's lymphoma	Lymphoma
WSU-DLCL2	6.7	651.5	18332.5	1362.5	18332.5	4.0	751.4	7328.7	1394.2	7328.7	DLBCL	Lymphoma
L-428	4.4	665.1	29325.5	303.1	29325.5	4.0	291.0	0.0	145.6	0.0	Hodgkin's lymphoma	Lymphoma
Hs445	2.6	976.1	15892.2	-10.7	29325.5	2.0	392.6	18997.6	23.3	0.0	Hodgkin's lymphoma	Lymphoma
SU-DHL-8	5.9	1220.3	19159.6	24.8	20651.6	9.0	2189.5	13341.2	99.0	13025.3	DLBCL	Lymphoma
RL	4.3	1434.2	18775.9	253.7	18775.9	12.0	4181.1	7979.8	183.1	7979.8	DLBCL	Lymphoma
OCI-LY3	5.0	1512.9	12613.8	292.5	13124.5	3.0	1013.4	3549.0	359.8	2664.4	DLBCL	Lymphoma
REC-1	3.8	1994.2	15596.0	229.3	18399.8	5.0	1636.1	11540.7	351.4	12970.4	MCL	Lymphoma
RCK8	4.3	2110.5	22666.5	609.3	22666.5	11.0	1599.4	7650.6	126.6	7650.6	DLBCL	Lymphoma

GSK3326595 6d Proliferation Data

Cell Line	Average of Pop Doubling	Average of gIC50 (nM)	Average of gIC100 (nM)	Average of Ymin-T0 (%)	Average of dEC50 (nM)	Sum of n=	StdDev of gIC50 (nM)	StdDev of gIC100 (nM)	StdDev of Ymin-T0 (%)	StdDev of dEC50 (nM)	Tumor subtype	General tumor type
Z-138	5.1	3.6	80.7	-98.3	131.3	3.0	1.6	86.9	2.9	151.5	MCL	Lymphoma
SU-DHL-6	6.1	3.9	202.0	-99.0	299.9	2.0	0.1	130.5	1.4	142.3	DLBCL	Lymphoma
U937	8.1	6.5	11501.5	-57.6	12048.6	3.0	2.5	15440.0	73.5	14980.9	DLBCL	Lymphoma
WSU-NHL	3.4	8.4	1422.5	-89.7	3167.6	3.0	6.8	2225.5	9.9	4510.1	DLBCL	Lymphoma
JVM-2	2.5	8.6	798.6	-39.4	29325.5	2.0	1.1	694.0	7.1	0.0	MCL	Lymphoma
RL	6.4	11.8	29325.5	435.9	29325.5	2.0	5.9	0.0	242.9	0.0	DLBCL	Lymphoma
RIVA	4.2	18.2	1833.2	-35.3	23273.9	2.0	1.9	247.7	25.3	8558.3	DLBCL	Lymphoma
SU-DHL-5	6.4	28.3	799.8	-100.0	1061.9	4.0	5.7	502.4	0.0	660.6	DLBCL	Lymphoma
SU-DHL-2	5.8	34.1	15070.4	194.7	22069.9	4.0	22.6	16468.6	410.8	14511.2	DLBCL	Lymphoma
SU-DHL-10	9.8	36.7	29325.5	6479.7	29325.5	3.0	48.5	0.0	8321.9	0.0	DLBCL	Lymphoma
SU-DHL-8	7.8	52.5	5520.3	-100.0	7178.1	2.0	65.2	4510.2	0.0	5297.4	DLBCL	Lymphoma
U2932	4.8	52.5	3475.0	-63.9	14846.3	3.0	20.0	1019.5	37.7	12539.6	DLBCL	Lymphoma
JM1	3.9	79.0	1292.1	-94.6	2655.4	3.0	58.9	898.6	9.3	1949.3	DLBCL	Lymphoma
WSU-DLCL2	7.1	81.7	29325.5	1537.9	29325.5	2.0	87.8	0.0	1557.8	0.0	DLBCL	Lymphoma
MAVER-1	3.6	84.9	18658.4	-11.4	29325.5	2.0	33.4	15085.6	31.2	0.0	MCL	Lymphoma
SU-DHL-16	4.6	98.9	29325.5	244.6	29325.5	2.0	29.6	0.0	141.7	0.0	DLBCL	Lymphoma
MINO	5.4	130.2	7862.5	-27.1	29325.5	2.0	3.7	3927.8	8.4	0.0	MCL	Lymphoma
DOHH-2	4.8	213.2	20852.1	43.4	24546.7	3.0	154.5	14676.4	96.8	8277.1	DLBCL	Lymphoma
NU-DUL-1	3.6	333.1	17723.8	34.5	29325.5	2.0	198.4	16407.4	86.1	0.0	DLBCL	Lymphoma
Pfeiffer	2.8	421.0	29325.5	55.7	29325.5	3.0	44.6	0.0	42.4	0.0	DLBCL	Lymphoma
SU-DHL-4	5.7	492.3	29325.5	926.3	29325.5	2.0	110.5	0.0	97.7	0.0	DLBCL	Lymphoma
RCK8	5.3	528.9	29325.5	1154.6	29325.5	4.0	299.4	0.0	246.8	0.0	DLBCL	Lymphoma
BJAB	5.0	1900.0	29325.5	326.9	29325.5	3.0	1672.9	0.0	165.3	0.0	DLBCL	Lymphoma
REC-1	3.7	2108.0	29325.5	148.7	29325.5	4.0	2757.0	0.0	51.9	0.0	MCL	Lymphoma
JeKo-1	5.5	7174.5	19712.3	400.2	19751.7	6.0	11348.7	14893.8	456.0	14833.4	MCL	Lymphoma
U2940	4.4	14789.3	29325.5	1050.7	29325.5	2.0	20557.4	0.0	950.6	0.0	DLBCL	Lymphoma
MDA-MB-453	3.3	28.7	29325.5	17.8	29325.5	2.0	16.5	0.0	7.1	0.0	Breast	Breast
MDA-MB-468	3.3	56.5	19652.6	11.9	24693.5	6.0	43.4	14986.0	60.0	11346.0	Breast	Breast
MCF-7	2.8	61.7	23633.4	27.2	29325.5	9.0	38.0	11434.7	32.6	0.0	Breast	Breast
SK-BR-3	3.3	73.1	25366.2	42.9	29325.5	7.0	91.4	10475.4	43.6	0.0	Breast	Breast
HCC1954	3.5	79.2	1126.8	-65.6	4173.4	2.0	27.2	185.5	3.7	287.9	Breast	Breast
HCC1806	4.2	97.6	11957.5	-24.4	19622.1	3.0	76.7	15367.5	54.3	16806.8	Breast	Breast
HCC70	2.4	129.8	2217.1	-23.1	29325.5	2.0	24.2	339.3	9.8	0.0	Breast	Breast
HCC1143	2.8	245.8	29325.5	144.7	29325.5	2.0	157.4	0.0	9.3	0.0	Breast	Breast
MDA-MB-231	4.1	543.7	29325.5	368.8	29325.5	3.0	447.6	0.0	105.1	0.0	Breast	Breast
ZR-75-1	1.8	10974.0	29325.5	114.7	29325.5	2.0	14488.3	0.0	2.8	0.0	Breast	Breast
5637	4.5	69.3	20933.4	197.9	24223.2	3.0	47.3	14535.5	394.3	8837.4	Bladder	Bladder
ScaBER	5.6	197.7	14758.3	-42.6	23434.9	3.0	34.5	12841.8	60.3	10202.8	Bladder	Bladder
SW-780	5.9	290.0	29325.5	1377.6	29325.5	2.0	205.8	0.0	1605.7	0.0	Bladder	Bladder
T24	5.8	764.6	29325.5	1177.3	29325.5	3.0	321.6	0.0	1109.1	0.0	Bladder	Bladder
HT-1376	3.3	17599.0	29325.5	556.7	29325.5	2.0	16583.8	0.0	592.8	0.0	Bladder	Bladder
J82	0.0	29325.5	29325.5	2053.5	29325.5	3.0	0.0	0.0	2095.9	0.0	Bladder	Bladder
UM-UC-3	4.9	29325.5	29325.5	2302.4	29325.5	3.0	0.0	0.0	2381.6	0.0	Bladder	Bladder
U-87MG	3.7	39.6	29325.5	176.6	29325.5	2.0	11.2	0.0	55.4	0.0	GBM	GBM
SF539	3.5	60.9	1548.9	-67.2	16184.1	2.0	11.3	411.7	38.5	18584.7	GBM	GBM
LN-229	3.8	254.2	29325.5	444.3	29325.5	2.0	55.7	0.0	79.3	0.0	GBM	GBM
A172	3.2	1167.3	29325.5	380.0	29325.5	2.0	354.3	0.0	33.6	0.0	GBM	GBM
U-251	4.5	1798.3	29325.5	507.4	29325.5	3.0	557.2	0.0	259.8	0.0	GBM	GBM
SF295	2.2	24722.2	29325.5	755.6	29325.5	3.0	7973.1	0.0	420.6	0.0	GBM	GBM
RPMI-8226	3.2	21.7	10826.1	-12.2	29325.5	3.0	6.5	16046.6	11.9	0.0	MM	MM
KMS-11	4.3	66.5	351.2	-36.5	25029.2	5.0	54.0	171.6	14.9	6253.4	MM	MM
RPMI-8226-LR5	3.1	285.7	18313.3	106.6	29325.5	2.0	112.9	15573.6	174.4	0.0	MM	MM
LP1	4.9	1944.9	29325.5	1350.7	29325.5	5.0	3939.5	0.0	758.0	0.0	MM	MM
RPMI-8226-DOX	2.7	29325.5	29325.5	502.9	29325.5	2.0	0.0	0.0	538.7	0.0	MM	MM
PER-403	4.8	38.3	1142.4	-52.3	21100.4	3.0	15.3	617.6	24.3	14246.3	NMC	NMC
PER-624	7.5	523.8	29325.5	822.9	29325.5	2.0	80.3	0.0	25.2	0.0	NMC	NMC
PER-704	4.8	657.8	29325.5	566.3	29325.5	2.0	572.7	0.0	328.1	0.0	NMC	NMC
NCI-H526	3.4	275.1	7420.2	-34.2	27502.7	2.0	70.8	2392.5	25.0	2577.8	Lung	Lung
NCI-H526-R	3.9	343.7	9759.0	-57.8	20960.4	3.0	90.5	3569.6	22.2	7269.5	Lung	Lung

U2940	4.6	2348.5	20408.3	593.4	20439.9	6.0	3107.7	9859.0	588.9	9881.5	DLBCL	Lymphoma
RPMI-6666	2.9	2712.2	29325.5	144.6	29325.5	2.0	2486.4	0.0	76.2	0.0	Hodgkin's lymphoma	Lymphoma
BJAB	5.0	3100.3	20460.7	132.7	20953.7	7.0	2509.8	7307.3	125.5	7831.1	DLBCL	Lymphoma
MHH-PREB-1	5.9	3299.1	30791.8	664.9	30791.8	5.0	6921.3	3278.7	200.7	3278.7	BCL	Lymphoma
OCILY-10	3.7	4467.6	8640.2	120.9	11813.5	7.0	10972.1	10477.4	295.8	11108.1	DLBCL	Lymphoma
JeKo-1	5.3	6191.2	12532.2	436.2	12742.5	20.0	8254.0	12830.9	556.4	12664.1	MCL	Lymphoma
OCILY-1	3.1	29325.5	29325.5	532.9	29325.5	2.0	0.0	0.0	132.1	0.0	DLBCL	Lymphoma
MEL-JUSO	4.7	79.3	29325.5	511.8	29325.5	3.0	28.8	0.0	71.3	0.0	Melanoma	Melanoma
SK-MEL-5	3.7	111.1	29325.5	183.3	29325.5	5.0	76.0	0.0	122.7	0.0	Melanoma	Melanoma
A-375	6.9	647.4	29325.5	2727.5	29325.5	6.0	362.9	0.0	1171.2	0.0	Melanoma	Melanoma
HT-144	3.6	1051.2	29325.5	306.2	29325.5	4.0	125.0	0.0	79.6	0.0	Melanoma	Melanoma
MEL-HO	5.0	1551.3	29325.5	655.9	29325.5	2.0	1040.6	0.0	374.6	0.0	Melanoma	Melanoma
MDA-MB-435S	2.4	2224.3	29325.5	151.5	29325.5	3.0	1924.5	0.0	126.0	0.0	Melanoma	Melanoma
SH-4	3.4	2561.9	29325.5	261.5	29325.5	5.0	1769.0	0.0	103.7	0.0	Melanoma	Melanoma
COLO-741	2.4	2907.2	29325.5	154.8	29325.5	5.0	2642.1	0.0	49.0	0.0	Melanoma	Melanoma
G-361	4.1	4355.9	29325.5	535.5	29325.5	3.0	2975.8	0.0	166.2	0.0	Melanoma	Melanoma
IGR-1	4.3	8755.3	29325.5	670.0	29325.5	4.0	5772.3	0.0	251.0	0.0	Melanoma	Melanoma
WM-266-4	3.4	17213.5	29325.5	493.5	29325.5	3.0	11981.0	0.0	51.9	0.0	Melanoma	Melanoma
RVH-421	3.5	29325.5	29325.5	553.4	29325.5	2.0	0.0	0.0	194.7	0.0	Melanoma	Melanoma
OPM-2	2.1	40.2	825.0	-27.7	36656.9	2.0	8.1	154.9	21.0	0.0	MM	MM
KMS-11	4.5	46.8	3973.6	-38.8	23451.4	8.0	33.7	10244.3	17.2	10988.5	MM	MM
L363	6.3	65.7	17303.9	61.8	22727.3	4.0	22.0	14013.3	88.0	13196.5	MM	MM
RPMI-8226	3.5	134.7	18263.5	97.9	30372.8	7.0	183.6	16296.3	156.9	2771.0	MM	MM
JJN3	6.2	163.6	31158.4	304.5	31158.4	4.0	68.6	10997.1	270.8	10997.1	MM	MM
KMS-12-BM	6.0	190.5	21176.9	534.8	21831.2	9.0	121.8	12338.6	319.1	11740.4	MM	MM
RPMI-8226-DOX	4.3	204.0	10083.2	6.7	15936.3	4.0	188.2	13061.1	111.6	15463.7	MM	MM
ARH-77	5.3	237.6	16220.7	64.1	22755.9	9.0	227.1	11327.8	172.6	10615.8	MM	MM
RPMI-8226-LR5	4.0	303.3	8744.3	6.3	20527.9	6.0	338.5	10474.0	65.6	13629.3	MM	MM
AMO-1	5.4	336.9	29325.5	240.4	29325.5	3.0	331.9	12698.3	132.1	12698.3	MM	MM
RPMI-8226-MR20	3.9	383.7	10706.9	44.3	11391.5	3.0	223.7	16125.0	149.0	15539.6	MM	MM
U266B1	2.7	652.2	17262.6	32.7	19692.3	7.0	611.5	12323.1	61.1	12636.6	MM	MM
Huns1	6.5	887.2	14208.0	7.3	15010.3	6.0	937.5	12050.5	174.7	11589.5	MM	MM
LP1	4.7	975.0	30547.4	693.5	30547.4	12.0	2301.8	2853.7	359.7	2853.7	MM	MM
SK-MM-2	1.8	1257.4	24892.3	-14.7	36656.9	2.0	1083.7	16637.7	21.4	0.0	MM	MM
MC/CAR	5.0	2741.8	26602.4	578.3	26602.4	7.0	3079.0	10789.1	322.9	10789.1	MM	MM
MOLP-2	1.7	22073.1	36656.9	77.1	36656.9	2.0	7823.8	0.0	12.4	0.0	MM	MM
CHP-212	3.9	16.3	29325.5	73.2	29325.5	2.0	12.7	0.0	19.0	0.0	Neuroblastoma	Other
PER-403	4.8	29.2	2120.5	-50.2	20430.7	3.0	10.6	1025.1	43.1	15406.3	NMC	Other
JY	3.7	95.1	346.6	-62.3	10270.4	3.0	49.1	241.7	21.9	16505.1	3V transformed lymphoblasto	Other
SNU16	5.1	174.9	7766.6	-38.6	15702.4	6.0	133.1	7878.5	24.3	11683.9	Stomach	Other
IGROV-1	3.5	188.9	19550.4	198.7	19561.1	3.0	86.0	8465.5	128.1	8456.2	Ovarian	Other
G-401	7.8	189.0	29325.5	1621.4	29325.5	2.0	9.2	0.0	1096.5	0.0	Kidney	Other
K562	8.1	310.3	29325.5	1841.4	29325.5	2.0	116.7	0.0	229.9	0.0	CML	Other
SW982	3.3	368.9	29325.5	467.1	29325.5	2.0	45.0	0.0	519.0	0.0	Synovial sarcoma	Other
PER-704	4.8	374.5	29325.5	678.2	29325.5	2.0	159.2	0.0	259.4	0.0	NMC	Other
SK-UT-1	5.0	392.7	36656.9	401.0	36656.9	3.0	18.7	0.0	63.1	0.0	Sarcoma	Other
A498	4.8	435.1	29325.5	599.3	29325.5	2.0	257.8	0.0	125.4	0.0	Kidney	Other
PER-624	7.5	526.4	29325.5	1032.7	29325.5	2.0	235.5	0.0	263.5	0.0	NMC	Other
CAKI-1	4.4	577.9	29325.5	568.8	29325.5	2.0	304.1	0.0	13.3	0.0	Kidney	Other
THP-1	3.9	950.7	34824.1	229.8	34824.1	4.0	853.7	3665.7	77.6	3665.7	Leukemia	Other
NALM-6	4.5	1058.6	14662.8	193.3	14662.8	2.0	403.8	0.0	198.8	0.0	ALL	Other
PC-3	4.2	1893.2	26881.7	480.2	26881.7	12.0	2263.3	5707.4	185.1	5707.4	Prostate	Other
SK-NEP-1	4.2	2241.0	34213.1	272.3	34213.1	3.0	776.7	4232.8	229.8	4232.8	Kidney	Other
DU145	4.1	3716.5	24926.7	637.1	24926.7	5.0	1784.1	9836.1	220.0	9836.1	Prostate	Other
HEL 92.1.7	4.6	12416.1	17106.6	572.4	17106.6	3.0	20993.5	16931.1	308.9	16931.1	Leukemia	Other
LNCaP	4.1	18932.7	23827.0	938.5	23827.0	8.0	9781.0	7588.7	254.9	7588.7	Prostate	Other
22Rv1	2.7	23460.4	23460.4	797.4	23460.4	5.0	8031.1	8031.1	236.9	8031.1	Prostate	Other
769-P	2.7	29325.5	29325.5	3006.1	29325.5	2.0	0.0	0.0	32.0	0.0	Kidney	Other
VCaP	0.0	29325.5	29325.5	163.1	29325.5	2.0	0.0	0.0	54.9	0.0	Prostate	Other
A204	2.6	36656.9	36656.9	324.0	36656.9	2.0	0.0	0.0	118.2	0.0	Sarcoma	Other
HOS	6.0	36656.9	36656.9	3356.6	36656.9	3.0	0.0	0.0	385.3	0.0	Sarcoma	Other
KP-3	3.6	99.9	17954.6	82.3	29325.5	5.0	39.0	15571.0	161.2	0.0	Pancreatic	Pancreatic
SW-1990	3.0	124.7	29325.5	110.7	29325.5	2.0	32.6	0.0	109.6	0.0	Pancreatic	Pancreatic
PANC-08.13	3.8	135.5	22889.8	36.3	29325.5	3.0	74.3	11147.0	54.9	0.0	Pancreatic	Pancreatic
HuP-T4	4.0	189.5	29325.5	196.4	29325.5	3.0	151.8	0.0	60.2	0.0	Pancreatic	Pancreatic
Miapaca-2	5.3	263.3	29325.5	1126.4	29325.5	3.0	242.7	0.0	279.6	0.0	Pancreatic	Pancreatic
ASPC1	3.5	423.8	29325.5	319.9	29325.5	3.0	497.7	0.0	69.9	0.0	Pancreatic	Pancreatic
PSN-1	4.1	503.9	29325.5	158.3	29325.5	4.0	339.0	0.0	28.3	0.0	Pancreatic	Pancreatic
PANC-04.03	3.5	622.3	29325.5	275.1	29325.5	4.0	175.3	0.0	113.0	0.0	Pancreatic	Pancreatic
HPAC	5.3	901.2	29325.5	986.3	29325.5	5.0	860.5	0.0	517.8	0.0	Pancreatic	Pancreatic
PANC-1	3.8	939.3	29325.5	394.0	29325.5	2.0	185.9	0.0	4.8	0.0	Pancreatic	Pancreatic
HPAFII	1.8	957.1	23993.9	9.9	29325.5	3.0	210.6	9234.5	25.7	0.0	Pancreatic	Pancreatic
CAPAN2	2.4	1058.0	21798.3	41.6	29325.5	2.0	153.7	10645.1	88.7	0.0	Pancreatic	Pancreatic
CAPAN1	2.8	1109.2	29325.5	125.4	29325.5	2.0	512.2	0.0	40.3	0.0	Pancreatic	Pancreatic
PANC-02.03	3.8	1374.5	29325.5	364.1	29325.5	2.0	921.3	0.0	108.6	0.0	Pancreatic	Pancreatic
Hs766T	3.4	1974.5	29325.5	333.1	29325.5	2.0	963.6	0.0	76.5	0.0	Pancreatic	Pancreatic
PANC-10.05	2.9	2079.0	29325.5	225.6	29325.5	2.0	407.9	0.0	30.8	0.0	Pancreatic	Pancreatic
PANC-03.27	2.8	2483.2	29325.5	114.9	29325.5	3.0	2682.6	0.0	74.6	0.0	Pancreatic	Pancreatic
HuP-T3	1.7	3386.4	29325.5	73.7	29325.5	2.0	1209.2	0.0	21.6	0.0	Pancreatic	Pancreatic
KP-4	5.7	20836.0	29325.5	2372.2	29325.5	3.0	14704.2	0.0	390.1	0.0	Pancreatic	Pancreatic
PA-TU-8988T	2.2	29325.5	29325.5	1785.7	29325.5	2.0	0.0	0.0	150.3	0.0	Pancreatic	Pancreatic

Supplementary Table 2: Correlation Between GSK3203591 and GSK3326595 6-day Proliferation Assay Data

Cell Line	gIC50 (uM)	
	**GSK3326595**	**GSK3203591**
5637	0.069	0.143
A172	1.167	0.590
BJAB	1.900	3.100
DOHH-2	0.213	0.116
HCC1143	0.246	0.509
HCC1806	0.098	0.261
HCC70	0.130	0.522
HT-1376	17.599	13.130
J82	29.326	20.947
JeKo-1	5.402	6.191
JM1	0.079	0.076
JVM-2	0.009	0.172
KMS-11	0.066	0.047
LN-229	0.254	1.150
LP1	1.945	0.975
MAVER-1	0.085	0.133
MCF-7	0.062	0.215
MDA-MB-231	0.544	2.928
MDA-MB-453	0.029	0.343
MDA-MB-468	0.057	0.585
MINO	0.130	0.536
NCI-H526	0.275	0.138
NCI-H526-R	0.344	0.219
NU-DHL-1	0.333	0.101
PER-403	0.038	0.029
PER-624	0.524	0.526
PER-704	0.658	0.375
Pfeiffer	0.421	0.352
RCK8	0.529	2.110
REC-1	2.108	1.994
RIVA	0.018	0.068
RL	0.014	1.434
ScaBER	0.198	0.255
SF295	24.722	9.843
SF539	0.061	0.075
SK-BR-3	0.073	0.131
SU-DHL-10	0.054	0.495
SU-DHL-16	0.099	0.124
SU-DHL-2	0.028	0.086
SU-DHL-4	0.492	0.571

SU-DHL-5	0.028	0.132
SU-DHL-6	0.004	0.013
SU-DHL-8	0.053	1.220
SW-780	0.290	0.451
T24	0.765	0.650
U251MG	1.798	3.491
U2932	0.053	0.336
U2940	10.176	2.349
U-87MG	0.040	0.127
U937	0.007	0.049
UM-UC-3	29.326	19.169
WSU-DLCL2	0.082	0.651
WSU-NHL	0.008	0.076
Z-138	0.004	0.014
ZR-75-1	10.974	2.615

Supplementary Table 3: gIC50 and Activity Area values for 10-day Proliferation Assay (Eurofins panel)

CellLine	gIC50 (nM)	ActivityArea	TumorType
SW-13	211	3.37	Adrenal
NCI-H295R	10000	1.23	Adrenal
MOLT-3	217	3.72	ALL
NALM-6	110	4.32	ALL
MOLT-16	54.2	4.95	ALL
CEM-C1	7.92	6.64	ALL
Jurkat	130	4.17	ALL
CCRFCEM	3140	1.25	ALL
MV-4-11	65.8	4.8	AML
HEL-92-1-7	9.02	6.46	AML
Thp1	80.5	4.51	AML
UM-UC-3	105	4.49	Bladder
TCCSUP	235	2.85	Bladder
T24	449	2.76	Bladder
BFTC-905	259	3.46	Bladder
SCaBER	123	4.22	Bladder
HT-1197	98.9	4.47	Bladder
J82	83.1	4.4	Bladder
647-V	62	4.91	Bladder
5637.000	285	3.5	Bladder
HT1376	297	3.48	Bladder
EFM-19	69.5	4.42	Breast
T47D	52.4	4.07	Breast
MDA MB 468	4.83	5.05	Breast
KPL-1	1330	1.92	Breast
MT-3	46.4	4.72	Breast
CAMA-1	78.9	4.27	Breast
MX1	294	3.29	Breast
BT474	172	3.41	Breast
SK-BR-3	76.4	4.28	Breast
AU565	14.6	5.44	Breast
MDA MB 453	64.5	4.23	Breast
MCF7	10.9	5.15	Breast
MDA-MB-436	49.7	5.04	Breast
MDA MB 231	56.7	5	Breast
BT-549	59.4	2.74	Breast
Hs 578T	947	2.06	Breast
BT20	2280	1.46	Breast
ST486	101	4.41	Burkitts
Daudi	142	4.05	Burkitts
Raji	24.5	5.64	Burkitts

EB-3	299	3.45	Burkitts
Ramos (RA 1)	546	2.79	Burkitts
DoTc2 4510	95.8	4.42	Cervix
C-4 I	139	4.13	Cervix
C-4 II	266	3.54	Cervix
HeLa	230	3.51	Cervix
C-33A	228	3.45	Cervix
SiHa	2510	1.55	Cervix
JEG-3	216	3.81	Choriocarcinoma
JAR	485	2.95	Choriocarcinoma
BeWo	1090	2.24	Choriocarcinoma
BV-173	10.6	6.26	CML
EM-2	94	4.54	CML
CML-T1	16.1	5.95	CML
MEG01	10000	0.05	CML
K562	146	3.97	CML
Colo 201	49.6	4.93	Colon
LS-174T	170	3.99	Colon
SW480	413	3.2	Colon
RKO-AS45-1	113	4.31	Colon
RKO	772	2.71	Colon
SW48	10000	1.59	Colon
HCT-116	166	3.91	Colon
NCI-H747	2580	1.56	Colon
SW620	5640	1.26	Colon
SW948	120	4.1	Colon
HT-29	95.2	4.32	Colon
Colo 205	10000	1.03	Colon
WiDr	505	2.38	Colon
SW837	102	4.23	Colon
Colo 320 HSR	80.3	4.49	Colon
DLD-1	134	4.13	Colon
RKOE6	210	3.66	Colon
SW403	153	3.67	Colon
HCT-15	68.4	4.77	Colon
Colo 320DM	82.5	4.65	Colon
LS1034	172	3.85	Colon
NCI-H508	307	3.48	Colon
HCT-8	430	3.18	Colon
SW1417	773	2.59	Colon
DOHH-2	157	3.95	DLBCL
DB	2.51	7.57	DLBCL
SR	275	3.44	DLBCL
HT	206	3.58	DLBCL
AN3 CA	254	3.37	Endometrium
HEC-1-A	2440	1.98	Endometrium
KLE	10000	1.05	Endometrium

RL95-2	218	3.47	Endometrium
OE21	146	3.99	Esophageal
OE19	98	4.45	Esophageal
OE33	231	3.7	Esophageal
OCUG-1	379	3.12	Gallbladder
SNU-16	34.4	5.36	Gastric
AGS	2030	1.49	Gastric
SNU-1	1040	2.25	Gastric
SNU-5	110	4.34	Gastric
KATO III	16.7	6.09	Gastric
HS 746T	171	3.86	Gastric
SW1088	40.5	5.09	Glioma
DBTRG-05MG	156	2.13	Glioma
A172	29.6	5.28	Glioma
U-87 MG	67.6	3.26	Glioma
DK-MG	89.5	4.52	Glioma
H4	151	3.91	Glioma
T98G	27.3	5.47	Glioma
SNB-19	223	3.27	Glioma
CCF-STTG1	115	4.32	Glioma
SW1783	389	2.92	Glioma
U-138MG	43.3	5.04	Glioma
L-428	330	2.98	HL
RPMI 6666	748	2.6	HL
Cal 27	103	4.46	HN
Detroit 562	104	4.36	HN
SCC-9	747	2.69	HN
SCC-4	286	2.85	HN
SCC-25	273	3.32	HN
SW1463	770	2.36	HN
FaDu	15.4	6.08	HN sq
Caki-1	68.8	4.79	Kidney
G-402	99.7	4.3	Kidney
Caki-2	114	4.32	Kidney
786-O	20.3	5.64	Kidney
A498	50.7	5.01	Kidney
ACHN	38.6	5.27	Kidney
769-P	203	3.75	Kidney
SK-NEP-1	264	2.87	Kidney
HuCCT1	225	3.52	Liver
HUH-6 Clone 5	361	3.09	Liver
SNU-423	66	4.77	Liver
HepG2	214	3.55	Liver
HLE	174	3.77	Liver
D283 Med	23.5	5.48	Meduloblastoma
Daoy	270	3.56	Meduloblastoma
COLO 829	54.4	4.09	Melanoma

Hs 695T	602	2.82	Melanoma
Hs 294T	12.3	5.68	Melanoma
SH-4	75.2	4.66	Melanoma
A7	36.7	5.29	Melanoma
C32	14.1	5.08	Melanoma
HMCB	666	2.33	Melanoma
C32TG	103	4.15	Melanoma
MeWo	249	3.7	Melanoma
SK-MEL-1	38.7	5.04	Melanoma
SK-MEL-3	181	3.38	Melanoma
SK-MEL-28	239	3.48	Melanoma
A101D	190	3.47	Melanoma
RPMI-7951	44.2	5.08	Melanoma
A375	79.6	4.05	Melanoma
CHL-1	247	3.6	Melanoma
MALME3M	265	3.55	Melanoma
RPMI 8226	58.4	4.58	MM
U266B1	78.5	4.18	MM
SKO-007	84.4	4.01	MM
CHP-212	24	5.24	Neuroblastoma
SK-N-FI	308	3.32	Neuroblastoma
MC-IXC	615	2.67	Neuroblastoma
SK-N-AS	31.9	3.45	Neuroblastoma
SK-N-DZ	257	3.65	Neuroblastoma
BE(2)C	803	2.42	Neuroblastoma
MHH-PREB-1	10000	0.38	NHL
Wi38	18.5	3.96	Normal
BPH1	718	2.74	Normal
ChaGoK1	1190	2.06	NSCLC
NCI-H460	224	3.53	NSCLC
A427	130	4.02	NSCLC
NCI-H596	188	2.89	NSCLC
COR-L105	314	3.49	NSCLC
NCIH441	556	2.88	NSCLC
Calu6	206	3.34	NSCLC
COR-L23	147	4.15	NSCLC
NCI-H292	166	3.25	NSCLC
SW900	3250	1.26	NSCLC
Calu1	3050	1.37	NSCLC
SKMES1	807	2.26	NSCLC
NCI-H661	152	3.94	NSCLC
A549	80.9	4.57	NSCLC
NCI-H520	15.6	5.93	NSCLC sq
ES-2	106	4.24	Ovarian
OVCAR3	51.4	5.03	Ovarian
CaOV3	298	3.4	Ovarian
SKOV3	52.6	4.21	Ovarian

Mia PaCa-2	158	3.75	Pancreatic
HPAF-II	154	4.05	Pancreatic
Hs 766T	1120	1.62	Pancreatic
BxPC-3	160	3.87	Pancreatic
AsPC-1	302	3.26	Pancreatic
Capan-2	69.7	4.63	Pancreatic
HuP-T4	84.9	4.21	Pancreatic
PANC-1	165	3.97	Pancreatic
CFPAC-1	52.3	4.98	Pancreatic
YAPC	168	3.88	Pancreatic
SU.86.86	934	2.2	Pancreatic
Capan-1	370	2.9	Pancreatic
BC-1	69.6	4.67	PEL
CRO-AP2	250	3.58	PEL
ARH-77	44.4	4.72	Plasma cell leukimia
PC-3	873	2.36	Prostate
BM-1604	142	4.01	Prostate
DU145	151	4.02	Prostate
22Rv1	2480	1.97	Prostate
LNCaP	2930	1.61	Prostate
Y79	113	3.36	Retinoblastoma
HT-3	10000	0.92	Retinoblastoma
G-401	38.4	5.26	Rhabdoid
RD	639	2.85	Sarcoma
SJRH30	472	3.11	Sarcoma
SW872	54	4.97	Sarcoma
MES-SA	72	4.58	Sarcoma
SK-UT-1	42.1	5.16	Sarcoma
MG-63	96	4.31	Sarcoma
SaOS2	48.2	5.07	Sarcoma
TE 381.T	147	3.97	Sarcoma
A204	223	3.73	Sarcoma
SW982	56.7	4.95	Sarcoma
SW1353	129	4.23	Sarcoma
SJSA1	773	2.34	Sarcoma
SK-LMS-1	113	4.24	Sarcoma
A-673	119	4.12	Sarcoma
HT-1080	53.5	4.05	Sarcoma
KHOS-240S	124	3.92	Sarcoma
HOS	223	3.72	Sarcoma
U2OS	485	3.08	Sarcoma
SW684	1170	1.84	Sarcoma
DMS273	298	3.1	SCLC
DMS53	106	4.19	SCLC
NCI-H69	144	3.22	SCLC
DMS114	1010	2.35	SCLC
SHP-77	152	2.98	SCLC

NCIH446	570	2.88	SCLC
A431	549	2.85	Skin
HLF	87.7	4.6	Skin
J-RT3-T3-5	43.2	5.09	TCL
CAL-62	253	3.65	Thyroid
CGTH-W-1	82.2	4.62	Thyroid
SW579	83.3	4.6	Thyroid
BHT-101	214	3.72	Thyroid
639-V	342	3.41	Ureter
SW962	203	3.58	Vulva
SW954	10000	0.31	Vulva

Supplementary Table 4: Z-138 Alternative Splicing MsigDB Analysis

Overlap Results						
Collection(s):	CP, CP:BIOCARTA, CP:KEGG, CP:REACTOME, H					
# overlaps shown:	10					
# genesets in collections:	1379					
# genes in comparison (n):	2093					
# genes in universe (N):	45956					
Gene Set Name	# Genes in Gene Set (K)	Description	# Genes in Overlap (k)	k/K	p-value	FDR q-value
REACTOME_CELL_CYCLE	421	Genes involved in Cell Cycle	93	0.2209	1.96E-37	2.70E-34
REACTOME_CELL_CYCLE_MITOTIC	325	Genes involved in Cell Cycle, Mitotic	79	0.2431	4.43E-35	3.05E-32
REACTOME_IMMUNE_SYSTEM	933	Genes involved in Immune System	126	0.135	9.78E-28	4.49E-25
REACTOME_DNA_REPLICATION	192	Genes involved in DNA Replication	48	0.25	2.00E-22	6.89E-20
REACTOME_MITOTIC_M_M_G1_PHASES	172	Genes involved in Mitotic M-M/G1 phases	43	0.25	3.05E-20	8.42E-18
REACTOME_METABOLISM_OF_PROTEINS	518	Genes involved in Metabolism of proteins	77	0.1486	7.11E-20	1.64E-17
REACTOME_METABOLISM_OF_RNA	330	Genes involved in Metabolism of RNA	58	0.1758	9.51E-19	1.87E-16
REACTOME_ADAPTIVE_IMMUNE_SYSTEM	539	Genes involved in Adaptive Immune System	75	0.1391	9.69E-18	1.67E-15
HALLMARK_E2F_TARGETS	200	Genes encoding cell cycle related targets of E2F transcription factors.	43	0.215	1.37E-17	1.89E-15
HALLMARK_G2M_CHECKPOINT	200	Genes involved in the G2/M checkpoint, as in progression through the cell division cycle.	43	0.215	1.37E-17	1.89E-15

Gene/Gene Set Overlap Matrix												
Entrez Gene Id	Gene Symbol	Gene Description	REACTOME_CELL_CYCLE	REACTOME_CELL_CYCLE_MITOTIC	REACTOME_IMMUNE_SYSTEM	REACTOME_DNA_REPLICATION	REACTOME_MITOTIC_M_M_G1_PHASES	REACTOME_METABOLISM_OF_PROTEINS	REACTOME_METABOLISM_OF_RNA	REACTOME_ADAPTIVE_IMMUNE_SYSTEM	HALLMARK_E2F_TARGETS	HALLMARK_G2M_CHECKPOINT
6233	RPS27A	ribosomal protein S27a	REACTOME_CELL_CYCLE	REACTOME_CELL_CYCLE_MITOTIC	REACTOME_IMMUNE_SYSTEM	REACTOME_DNA_REPLICATION	REACTOME_MITOTIC_M_M_G1_PHASES	REACTOME_METABOLISM_OF_PROTEINS	REACTOME_METABOLISM_OF_RNA	REACTOME_ADAPTIVE_IMMUNE_SYSTEM		
7311	UBA52	ubiquitin A-52 residue ribosomal protein fusion product 1	REACTOME_CELL_CYCLE	REACTOME_CELL_CYCLE_MITOTIC	REACTOME_IMMUNE_SYSTEM	REACTOME_DNA_REPLICATION	REACTOME_MITOTIC_M_M_G1_PHASES	REACTOME_METABOLISM_OF_PROTEINS	REACTOME_METABOLISM_OF_RNA	REACTOME_ADAPTIVE_IMMUNE_SYSTEM		
6396	SEC13	SEC13 homolog (S. cerevisiae)	REACTOME_CELL_CYCLE	REACTOME_CELL_CYCLE_MITOTIC	REACTOME_IMMUNE_SYSTEM	REACTOME_DNA_REPLICATION	REACTOME_MITOTIC_M_M_G1_PHASES	REACTOME_METABOLISM_OF_PROTEINS		REACTOME_ADAPTIVE_IMMUNE_SYSTEM		
10213	PSMD14	proteasome (prosome, macropain) 26S subunit, non-ATPase, 14	REACTOME_CELL_CYCLE	REACTOME_CELL_CYCLE_MITOTIC	REACTOME_IMMUNE_SYSTEM	REACTOME_DNA_REPLICATION	REACTOME_MITOTIC_M_M_G1_PHASES		REACTOME_METABOLISM_OF_RNA	REACTOME_ADAPTIVE_IMMUNE_SYSTEM		
5682	PSMA1	proteasome (prosome, macropain) subunit, alpha type, 1	REACTOME_CELL_CYCLE	REACTOME_CELL_CYCLE_MITOTIC	REACTOME_IMMUNE_SYSTEM	REACTOME_DNA_REPLICATION	REACTOME_MITOTIC_M_M_G1_PHASES		REACTOME_METABOLISM_OF_RNA	REACTOME_ADAPTIVE_IMMUNE_SYSTEM		
5687	PSMA6	proteasome (prosome, macropain) subunit, alpha type, 6	REACTOME_CELL_CYCLE	REACTOME_CELL_CYCLE_MITOTIC	REACTOME_IMMUNE_SYSTEM	REACTOME_DNA_REPLICATION	REACTOME_MITOTIC_M_M_G1_PHASES		REACTOME_METABOLISM_OF_RNA	REACTOME_ADAPTIVE_IMMUNE_SYSTEM		
5684	PSMA3	proteasome (prosome, macropain) subunit, alpha type, 3	REACTOME_CELL_CYCLE	REACTOME_CELL_CYCLE_MITOTIC	REACTOME_IMMUNE_SYSTEM	REACTOME_DNA_REPLICATION	REACTOME_MITOTIC_M_M_G1_PHASES		REACTOME_METABOLISM_OF_RNA	REACTOME_ADAPTIVE_IMMUNE_SYSTEM		
5720	PSME1	proteasome (prosome, macropain) activator subunit 1 (PA28 alpha)	REACTOME_CELL_CYCLE	REACTOME_CELL_CYCLE_MITOTIC	REACTOME_IMMUNE_SYSTEM	REACTOME_DNA_REPLICATION	REACTOME_MITOTIC_M_M_G1_PHASES		REACTOME_METABOLISM_OF_RNA	REACTOME_ADAPTIVE_IMMUNE_SYSTEM		
5686	PSMA5	proteasome (prosome, macropain) subunit, alpha type, 5	REACTOME_CELL_CYCLE	REACTOME_CELL_CYCLE_MITOTIC	REACTOME_IMMUNE_SYSTEM	REACTOME_DNA_REPLICATION	REACTOME_MITOTIC_M_M_G1_PHASES		REACTOME_METABOLISM_OF_RNA	REACTOME_ADAPTIVE_IMMUNE_SYSTEM		
5721	PSME2	proteasome (prosome, macropain) activator subunit 2 (PA28 beta)	REACTOME_CELL_CYCLE	REACTOME_CELL_CYCLE_MITOTIC	REACTOME_IMMUNE_SYSTEM	REACTOME_DNA_REPLICATION	REACTOME_MITOTIC_M_M_G1_PHASES		REACTOME_METABOLISM_OF_RNA	REACTOME_ADAPTIVE_IMMUNE_SYSTEM		
5705	PSMC5	proteasome (prosome, macropain) 26S subunit, ATPase, 5	REACTOME_CELL_CYCLE	REACTOME_CELL_CYCLE_MITOTIC	REACTOME_IMMUNE_SYSTEM	REACTOME_DNA_REPLICATION	REACTOME_MITOTIC_M_M_G1_PHASES		REACTOME_METABOLISM_OF_RNA	REACTOME_ADAPTIVE_IMMUNE_SYSTEM		
79902	NUP85	nucleoporin 85kDa	REACTOME_CELL_CYCLE	REACTOME_CELL_CYCLE_MITOTIC	REACTOME_IMMUNE_SYSTEM	REACTOME_DNA_REPLICATION	REACTOME_MITOTIC_M_M_G1_PHASES		REACTOME_METABOLISM_OF_RNA			
55746	NUP133	nucleoporin 133kDa	REACTOME_CELL_CYCLE	REACTOME_CELL_CYCLE_MITOTIC	REACTOME_IMMUNE_SYSTEM	REACTOME_DNA_REPLICATION	REACTOME_MITOTIC_M_M_G1_PHASES		REACTOME_METABOLISM_OF_RNA			
9493	KIF23	kinesin family member 23	REACTOME_CELL_CYCLE	REACTOME_CELL_CYCLE_MITOTIC	REACTOME_IMMUNE_SYSTEM	REACTOME_DNA_REPLICATION	REACTOME_MITOTIC_M_M_G1_PHASES			REACTOME_ADAPTIVE_IMMUNE_SYSTEM		HALLMARK_G2M_CHECKPOINT
3796	KIF2A	kinesin heavy chain member 2A	REACTOME_CELL_CYCLE	REACTOME_CELL_CYCLE_MITOTIC	REACTOME_IMMUNE_SYSTEM	REACTOME_DNA_REPLICATION	REACTOME_MITOTIC_M_M_G1_PHASES			REACTOME_ADAPTIVE_IMMUNE_SYSTEM		
5525	PPP2R5A	protein phosphatase 2, regulatory subunit B', alpha	REACTOME_CELL_CYCLE	REACTOME_CELL_CYCLE_MITOTIC	REACTOME_IMMUNE_SYSTEM	REACTOME_DNA_REPLICATION	REACTOME_MITOTIC_M_M_G1_PHASES			REACTOME_ADAPTIVE_IMMUNE_SYSTEM		
5527	PPP2R5C	protein phosphatase 2, regulatory subunit B', gamma	REACTOME_CELL_CYCLE	REACTOME_CELL_CYCLE_MITOTIC	REACTOME_IMMUNE_SYSTEM	REACTOME_DNA_REPLICATION	REACTOME_MITOTIC_M_M_G1_PHASES			REACTOME_ADAPTIVE_IMMUNE_SYSTEM		
11065	UBE2C	ubiquitin-conjugating enzyme E2C	REACTOME_CELL_CYCLE	REACTOME_CELL_CYCLE_MITOTIC	REACTOME_IMMUNE_SYSTEM					REACTOME_ADAPTIVE_IMMUNE_SYSTEM		HALLMARK_G2M_CHECKPOINT
10393	ANAPC10	anaphase promoting complex subunit 10	REACTOME_CELL_CYCLE	REACTOME_CELL_CYCLE_MITOTIC	REACTOME_IMMUNE_SYSTEM					REACTOME_ADAPTIVE_IMMUNE_SYSTEM		
51529	ANAPC11	anaphase promoting complex subunit 11	REACTOME_CELL_CYCLE	REACTOME_CELL_CYCLE_MITOTIC	REACTOME_IMMUNE_SYSTEM					REACTOME_ADAPTIVE_IMMUNE_SYSTEM		
8881	CDC16	cell division cycle 16 homolog (S. cerevisiae)	REACTOME_CELL_CYCLE	REACTOME_CELL_CYCLE_MITOTIC	REACTOME_IMMUNE_SYSTEM					REACTOME_ADAPTIVE_IMMUNE_SYSTEM		
6502	SKP2	S-phase kinase-associated protein 2 (p45)	REACTOME_CELL_CYCLE	REACTOME_CELL_CYCLE_MITOTIC	REACTOME_IMMUNE_SYSTEM					REACTOME_ADAPTIVE_IMMUNE_SYSTEM		
6232	RPS27	ribosomal protein S27	REACTOME_CELL_CYCLE	REACTOME_CELL_CYCLE_MITOTIC		REACTOME_DNA_REPLICATION	REACTOME_MITOTIC_M_M_G1_PHASES	REACTOME_METABOLISM_OF_PROTEINS	REACTOME_METABOLISM_OF_RNA			
7514	XPO1	exportin 1 (CRM1 homolog, yeast)	REACTOME_CELL_CYCLE	REACTOME_CELL_CYCLE_MITOTIC		REACTOME_DNA_REPLICATION	REACTOME_MITOTIC_M_M_G1_PHASES		REACTOME_METABOLISM_OF_RNA		HALLMARK_E2F_TARGETS	HALLMARK_G2M_CHECKPOINT
332	BIRC5	baculoviral IAP repeat containing 5	REACTOME_CELL_CYCLE	REACTOME_CELL_CYCLE_MITOTIC		REACTOME_DNA_REPLICATION	REACTOME_MITOTIC_M_M_G1_PHASES				HALLMARK_E2F_TARGETS	HALLMARK_G2M_CHECKPOINT
9212	AURKB	aurora kinase B	REACTOME_CELL_CYCLE	REACTOME_CELL_CYCLE_MITOTIC		REACTOME_DNA_REPLICATION	REACTOME_MITOTIC_M_M_G1_PHASES				HALLMARK_E2F_TARGETS	HALLMARK_G2M_CHECKPOINT
10274	STAG1	stromal antigen 1	REACTOME_CELL_CYCLE	REACTOME_CELL_CYCLE_MITOTIC		REACTOME_DNA_REPLICATION	REACTOME_MITOTIC_M_M_G1_PHASES				HALLMARK_E2F_TARGETS	HALLMARK_G2M_CHECKPOINT
701	BUB1B	budding uninhibited by benzimidazoles 1 homolog beta (yeast)	REACTOME_CELL_CYCLE	REACTOME_CELL_CYCLE_MITOTIC		REACTOME_DNA_REPLICATION	REACTOME_MITOTIC_M_M_G1_PHASES				HALLMARK_E2F_TARGETS	
147841	SPC24	SPC24, NDC80 kinetochore complex component, homolog (S. cerevisiae)	REACTOME_CELL_CYCLE	REACTOME_CELL_CYCLE_MITOTIC		REACTOME_DNA_REPLICATION	REACTOME_MITOTIC_M_M_G1_PHASES				HALLMARK_E2F_TARGETS	
699	BUB1	budding uninhibited by benzimidazoles 1 homolog (yeast)	REACTOME_CELL_CYCLE	REACTOME_CELL_CYCLE_MITOTIC		REACTOME_DNA_REPLICATION	REACTOME_MITOTIC_M_M_G1_PHASES					HALLMARK_G2M_CHECKPOINT
1869	E2F1	E2F transcription factor 1	REACTOME_CELL_CYCLE	REACTOME_CELL_CYCLE_MITOTIC		REACTOME_DNA_REPLICATION	REACTOME_MITOTIC_M_M_G1_PHASES					HALLMARK_G2M_CHECKPOINT
5001	ORC5	origin recognition complex, subunit 5	REACTOME_CELL_CYCLE	REACTOME_CELL_CYCLE_MITOTIC		REACTOME_DNA_REPLICATION	REACTOME_MITOTIC_M_M_G1_PHASES					HALLMARK_G2M_CHECKPOINT
9735	KNTC1	kinetochore associated 1	REACTOME_CELL_CYCLE	REACTOME_CELL_CYCLE_MITOTIC		REACTOME_DNA_REPLICATION	REACTOME_MITOTIC_M_M_G1_PHASES					
11130	ZWINT	ZW10 interactor	REACTOME_CELL_CYCLE	REACTOME_CELL_CYCLE_MITOTIC		REACTOME_DNA_REPLICATION	REACTOME_MITOTIC_M_M_G1_PHASES					
151648	SGOL1	shugoshin-like 1 (S. pombe)	REACTOME_CELL_CYCLE	REACTOME_CELL_CYCLE_MITOTIC		REACTOME_DNA_REPLICATION	REACTOME_MITOTIC_M_M_G1_PHASES					
23421	ITGB3BP	integrin beta 3 binding protein (beta3-endonexin)	REACTOME_CELL_CYCLE	REACTOME_CELL_CYCLE_MITOTIC		REACTOME_DNA_REPLICATION	REACTOME_MITOTIC_M_M_G1_PHASES					
55839	CENPN	centromere protein N	REACTOME_CELL_CYCLE	REACTOME_CELL_CYCLE_MITOTIC		REACTOME_DNA_REPLICATION	REACTOME_MITOTIC_M_M_G1_PHASES					
2491	CENPI	centromere protein I	REACTOME_CELL_CYCLE	REACTOME_CELL_CYCLE_MITOTIC		REACTOME_DNA_REPLICATION	REACTOME_MITOTIC_M_M_G1_PHASES					
79172	CENPO	centromere protein O	REACTOME_CELL_CYCLE	REACTOME_CELL_CYCLE_MITOTIC		REACTOME_DNA_REPLICATION	REACTOME_MITOTIC_M_M_G1_PHASES					
348235	SKA2	spindle and kinetochore associated complex subunit 2	REACTOME_CELL_CYCLE	REACTOME_CELL_CYCLE_MITOTIC		REACTOME_DNA_REPLICATION	REACTOME_MITOTIC_M_M_G1_PHASES					
55055	ZWILCH	Zwilch, kinetochore associated, homolog (Drosophila)	REACTOME_CELL_CYCLE	REACTOME_CELL_CYCLE_MITOTIC		REACTOME_DNA_REPLICATION	REACTOME_MITOTIC_M_M_G1_PHASES					
79980	DSN1	DSN1, MIND kinetochore complex component, homolog (S. cerevisiae)	REACTOME_CELL_CYCLE	REACTOME_CELL_CYCLE_MITOTIC		REACTOME_DNA_REPLICATION	REACTOME_MITOTIC_M_M_G1_PHASES					
80152	CENPT	centromere protein T	REACTOME_CELL_CYCLE	REACTOME_CELL_CYCLE_MITOTIC		REACTOME_DNA_REPLICATION	REACTOME_MITOTIC_M_M_G1_PHASES					
91687	CENPL	centromere protein L	REACTOME_CELL_CYCLE	REACTOME_CELL_CYCLE_MITOTIC		REACTOME_DNA_REPLICATION	REACTOME_MITOTIC_M_M_G1_PHASES					
5422	POLA1	polymerase (DNA directed), alpha 1, catalytic subunit	REACTOME_CELL_CYCLE	REACTOME_CELL_CYCLE_MITOTIC		REACTOME_DNA_REPLICATION	REACTOME_MITOTIC_M_M_G1_PHASES					
84515	MCM8	minichromosome maintenance complex component 8	REACTOME_CELL_CYCLE	REACTOME_CELL_CYCLE_MITOTIC		REACTOME_DNA_REPLICATION	REACTOME_MITOTIC_M_M_G1_PHASES					
64689	GORASP1	golgi reassembly stacking protein 1, 65kDa	REACTOME_CELL_CYCLE	REACTOME_CELL_CYCLE_MITOTIC		REACTOME_DNA_REPLICATION	REACTOME_MITOTIC_M_M_G1_PHASES					
5427	POLE2	polymerase (DNA directed), epsilon 2 (p59 subunit)	REACTOME_CELL_CYCLE	REACTOME_CELL_CYCLE_MITOTIC		REACTOME_DNA_REPLICATION	REACTOME_MITOTIC_M_M_G1_PHASES					
10714	POLD3	polymerase (DNA-directed), delta 3, accessory subunit	REACTOME_CELL_CYCLE	REACTOME_CELL_CYCLE_MITOTIC		REACTOME_DNA_REPLICATION					HALLMARK_E2F_TARGETS	
5424	POLD1	polymerase (DNA directed), delta 1, catalytic subunit 125kDa	REACTOME_CELL_CYCLE	REACTOME_CELL_CYCLE_MITOTIC		REACTOME_DNA_REPLICATION					HALLMARK_E2F_TARGETS	
9837	GINS1	GINS complex subunit 1 (Psf1 homolog)	REACTOME_CELL_CYCLE	REACTOME_CELL_CYCLE_MITOTIC		REACTOME_DNA_REPLICATION					HALLMARK_E2F_TARGETS	
5985	RFC5	replication factor C (activator 1) 5, 36.5kDa	REACTOME_CELL_CYCLE	REACTOME_CELL_CYCLE_MITOTIC		REACTOME_DNA_REPLICATION						
1763	DNA2	DNA replication helicase 2 homolog (yeast)	REACTOME_CELL_CYCLE	REACTOME_CELL_CYCLE_MITOTIC		REACTOME_DNA_REPLICATION						
6790	AURKA	aurora kinase A	REACTOME_CELL_CYCLE	REACTOME_CELL_CYCLE_MITOTIC							HALLMARK_E2F_TARGETS	HALLMARK_G2M_CHECKPOINT
6241	RRM2	ribonucleotide reductase M2	REACTOME_CELL_CYCLE	REACTOME_CELL_CYCLE_MITOTIC							HALLMARK_E2F_TARGETS	
7027	TFDP1	transcription factor Dp-1	REACTOME_CELL_CYCLE	REACTOME_CELL_CYCLE_MITOTIC								HALLMARK_G2M_CHECKPOINT
4751	NEK2	NIMA (never in mitosis gene a)-related kinase 2	REACTOME_CELL_CYCLE	REACTOME_CELL_CYCLE_MITOTIC								HALLMARK_G2M_CHECKPOINT
4926	NUMA1	nuclear mitotic apparatus protein 1	REACTOME_CELL_CYCLE	REACTOME_CELL_CYCLE_MITOTIC								HALLMARK_G2M_CHECKPOINT
1453	CSNK1D	casein kinase 1, delta	REACTOME_CELL_CYCLE	REACTOME_CELL_CYCLE_MITOTIC								
11064	CNTRL	centriolin	REACTOME_CELL_CYCLE	REACTOME_CELL_CYCLE_MITOTIC								
5116	PCNT	pericentrin	REACTOME_CELL_CYCLE	REACTOME_CELL_CYCLE_MITOTIC								
55125	CEP192	centrosomal protein 192kDa	REACTOME_CELL_CYCLE	REACTOME_CELL_CYCLE_MITOTIC								
55755	CDK5RAP2	CDK5 regulatory subunit associated protein 2	REACTOME_CELL_CYCLE	REACTOME_CELL_CYCLE_MITOTIC								
55835	CENPJ	centromere protein J	REACTOME_CELL_CYCLE	REACTOME_CELL_CYCLE_MITOTIC								
85378	TUBGCP6	tubulin, gamma complex associated protein 6	REACTOME_CELL_CYCLE	REACTOME_CELL_CYCLE_MITOTIC								
3320	HSP90AA1	heat shock protein 90kDa alpha (cytosolic), class A member 1	REACTOME_CELL_CYCLE	REACTOME_CELL_CYCLE_MITOTIC								
891	CCNB1	cyclin B1	REACTOME_CELL_CYCLE	REACTOME_CELL_CYCLE_MITOTIC								
995	CDC25C	cell division cycle 25 homolog C (S. pombe)	REACTOME_CELL_CYCLE	REACTOME_CELL_CYCLE_MITOTIC								
22897	CEP164	centrosomal protein 164kDa	REACTOME_CELL_CYCLE	REACTOME_CELL_CYCLE_MITOTIC								
121441	NEDD1	neural precursor cell expressed, developmentally down-regulated 1	REACTOME_CELL_CYCLE	REACTOME_CELL_CYCLE_MITOTIC								
10142	AKAP9	A kinase (PRKA) anchor protein (yotiao) 9	REACTOME_CELL_CYCLE	REACTOME_CELL_CYCLE_MITOTIC								
10806	SDCCAG8	serologically defined colon cancer antigen 8	REACTOME_CELL_CYCLE	REACTOME_CELL_CYCLE_MITOTIC								
80321	CEP70	centrosomal protein 70kDa	REACTOME_CELL_CYCLE	REACTOME_CELL_CYCLE_MITOTIC								
8481	OFD1	oral-facial-digital syndrome 1	REACTOME_CELL_CYCLE	REACTOME_CELL_CYCLE_MITOTIC								
8636	SSNA1	Sjogren syndrome nuclear autoantigen 1	REACTOME_CELL_CYCLE	REACTOME_CELL_CYCLE_MITOTIC								
9088	PKMYT1	protein kinase, membrane associated tyrosine/threonine 1	REACTOME_CELL_CYCLE	REACTOME_CELL_CYCLE_MITOTIC								
5928	RBBP4	retinoblastoma binding protein 4	REACTOME_CELL_CYCLE	REACTOME_CELL_CYCLE_MITOTIC								
1859	DYRK1A	dual-specificity tyrosine-(Y)-phosphorylation regulated kinase 1A	REACTOME_CELL_CYCLE	REACTOME_CELL_CYCLE_MITOTIC								
132660	LIN54	lin-54 homolog (C. elegans)	REACTOME_CELL_CYCLE	REACTOME_CELL_CYCLE_MITOTIC								
4193	MDM2	Mdm2 p53 binding protein homolog (mouse)	REACTOME_CELL_CYCLE		REACTOME_IMMUNE_SYSTEM					REACTOME_ADAPTIVE_IMMUNE_SYSTEM		
3364	HUS1	HUS1 checkpoint homolog (S. pombe)	REACTOME_CELL_CYCLE								HALLMARK_E2F_TARGETS	HALLMARK_G2M_CHECKPOINT
5810	RAD1	RAD1 homolog (S. pombe)	REACTOME_CELL_CYCLE								HALLMARK_E2F_TARGETS	
25777	SUN2	Sad1 and UNC84 domain containing 2	REACTOME_CELL_CYCLE									
4869	NPM1	nucleophosmin (nucleolar phosphoprotein B23, numatrin)	REACTOME_CELL_CYCLE									
10856	RUVBL2	RuvB-like 2 (E. coli)	REACTOME_CELL_CYCLE									
64326	RFWD2	ring finger and WD repeat domain 2	REACTOME_CELL_CYCLE									
472	ATM	ataxia telangiectasia mutated	REACTOME_CELL_CYCLE									
5884	RAD17	RAD17 homolog (S. pombe)	REACTOME_CELL_CYCLE									
7014	TERF2	telomeric repeat binding factor 2	REACTOME_CELL_CYCLE									
7013	TERF1	telomeric repeat binding factor (NIMA-interacting) 1	REACTOME_CELL_CYCLE									
25913	POT1	protection of telomeres 1 homolog (S. pombe)	REACTOME_CELL_CYCLE									
55320	MIS18BP1	MIS18 binding protein 1	REACTOME_CELL_CYCLE									
23224	SYNE2	spectrin repeat containing, nuclear envelope 2	REACTOME_CELL_CYCLE									
1977	EIF4E	eukaryotic translation initiation factor 4E			REACTOME_IMMUNE_SYSTEM			REACTOME_METABOLISM_OF_PROTEINS	REACTOME_METABOLISM_OF_RNA			
1981	EIF4G1	eukaryotic translation initiation factor 4 gamma, 1			REACTOME_IMMUNE_SYSTEM			REACTOME_METABOLISM_OF_PROTEINS	REACTOME_METABOLISM_OF_RNA			
1974	EIF4A2	eukaryotic translation initiation factor 4A2			REACTOME_IMMUNE_SYSTEM			REACTOME_METABOLISM_OF_PROTEINS	REACTOME_METABOLISM_OF_RNA			
55294	FBXW7	F-box and WD repeat domain containing 7			REACTOME_IMMUNE_SYSTEM			REACTOME_METABOLISM_OF_PROTEINS		REACTOME_ADAPTIVE_IMMUNE_SYSTEM		
10427	SEC24B	SEC24 family, member B (S. cerevisiae)			REACTOME_IMMUNE_SYSTEM			REACTOME_METABOLISM_OF_PROTEINS		REACTOME_ADAPTIVE_IMMUNE_SYSTEM		
5589	PRKCSH	protein kinase C substrate 80K-H			REACTOME_IMMUNE_SYSTEM			REACTOME_METABOLISM_OF_PROTEINS				
9972	NUP153	nucleoporin 153kDa			REACTOME_IMMUNE_SYSTEM				REACTOME_METABOLISM_OF_RNA		HALLMARK_E2F_TARGETS	
5600	MAPK11	mitogen-activated protein kinase 11			REACTOME_IMMUNE_SYSTEM				REACTOME_METABOLISM_OF_RNA			
9261	MAPKAPK2	mitogen-activated protein kinase-activated protein kinase 2			REACTOME_IMMUNE_SYSTEM				REACTOME_METABOLISM_OF_RNA			
9883	POM121	POM121 membrane glycoprotein			REACTOME_IMMUNE_SYSTEM				REACTOME_METABOLISM_OF_RNA			
8086	AAAS	achalasia, adrenocortical insufficiency, alacrimia			REACTOME_IMMUNE_SYSTEM				REACTOME_METABOLISM_OF_RNA			
9818	NUPL1	nucleoporin like 1			REACTOME_IMMUNE_SYSTEM				REACTOME_METABOLISM_OF_RNA			
23225	NUP210	nucleoporin 210kDa			REACTOME_IMMUNE_SYSTEM				REACTOME_METABOLISM_OF_RNA			
23636	NUP62	nucleoporin 62kDa			REACTOME_IMMUNE_SYSTEM				REACTOME_METABOLISM_OF_RNA			
7323	UBE2D3	ubiquitin-conjugating enzyme E2D 3			REACTOME_IMMUNE_SYSTEM					REACTOME_ADAPTIVE_IMMUNE_SYSTEM		
3831	KLC1	kinesin light chain 1			REACTOME_IMMUNE_SYSTEM					REACTOME_ADAPTIVE_IMMUNE_SYSTEM		
10075	HUWE1	HECT, UBA and WWE domain containing 1			REACTOME_IMMUNE_SYSTEM					REACTOME_ADAPTIVE_IMMUNE_SYSTEM		
6921	TCEB1	transcription elongation factor B (SIII), polypeptide 1 (15kDa, elongin C)			REACTOME_IMMUNE_SYSTEM					REACTOME_ADAPTIVE_IMMUNE_SYSTEM		
23291	FBXW11	F-box and WD repeat domain containing 11			REACTOME_IMMUNE_SYSTEM					REACTOME_ADAPTIVE_IMMUNE_SYSTEM		
7337	UBE3A	ubiquitin protein ligase E3A			REACTOME_IMMUNE_SYSTEM					REACTOME_ADAPTIVE_IMMUNE_SYSTEM		
9820	CUL7	cullin 7			REACTOME_IMMUNE_SYSTEM					REACTOME_ADAPTIVE_IMMUNE_SYSTEM		
10273	STUB1	STIP1 homology and U-box containing protein 1, E3 ubiquitin protein ligase			REACTOME_IMMUNE_SYSTEM					REACTOME_ADAPTIVE_IMMUNE_SYSTEM		
7317	UBA1	ubiquitin-like modifier activating enzyme 1			REACTOME_IMMUNE_SYSTEM					REACTOME_ADAPTIVE_IMMUNE_SYSTEM		
8453	CUL2	cullin 2			REACTOME_IMMUNE_SYSTEM					REACTOME_ADAPTIVE_IMMUNE_SYSTEM		
10055	SAE1	SUMO1 activating enzyme subunit 1			REACTOME_IMMUNE_SYSTEM					REACTOME_ADAPTIVE_IMMUNE_SYSTEM		
26259	FBXW8	F-box and WD repeat domain containing 8			REACTOME_IMMUNE_SYSTEM					REACTOME_ADAPTIVE_IMMUNE_SYSTEM		
55070	DET1	de-etiolated homolog 1 (Arabidopsis)			REACTOME_IMMUNE_SYSTEM					REACTOME_ADAPTIVE_IMMUNE_SYSTEM		
63893	UBE2O	ubiquitin-conjugating enzyme E2O			REACTOME_IMMUNE_SYSTEM					REACTOME_ADAPTIVE_IMMUNE_SYSTEM		
7320	UBE2B	ubiquitin-conjugating enzyme E2B			REACTOME_IMMUNE_SYSTEM					REACTOME_ADAPTIVE_IMMUNE_SYSTEM		
9320	TRIP12	thyroid hormone receptor interactor 12			REACTOME_IMMUNE_SYSTEM					REACTOME_ADAPTIVE_IMMUNE_SYSTEM		
9690	UBE3C	ubiquitin protein ligase E3C			REACTOME_IMMUNE_SYSTEM					REACTOME_ADAPTIVE_IMMUNE_SYSTEM		
10616	RBCK1	RanBP-type and C3HC4-type zinc finger containing 1			REACTOME_IMMUNE_SYSTEM					REACTOME_ADAPTIVE_IMMUNE_SYSTEM		
63891	RNF123	ring finger protein 123			REACTOME_IMMUNE_SYSTEM					REACTOME_ADAPTIVE_IMMUNE_SYSTEM		
26118	WSB1	WD repeat and SOCS box containing 1			REACTOME_IMMUNE_SYSTEM					REACTOME_ADAPTIVE_IMMUNE_SYSTEM		
27252	KLHL20	kelch-like 20 (Drosophila)			REACTOME_IMMUNE_SYSTEM					REACTOME_ADAPTIVE_IMMUNE_SYSTEM		
10425	ARIH2	ariadne homolog 2 (Drosophila)			REACTOME_IMMUNE_SYSTEM					REACTOME_ADAPTIVE_IMMUNE_SYSTEM		
122769	LRR1	leucine rich repeat protein 1			REACTOME_IMMUNE_SYSTEM					REACTOME_ADAPTIVE_IMMUNE_SYSTEM		
55182	RNF220	ring finger protein 220			REACTOME_IMMUNE_SYSTEM					REACTOME_ADAPTIVE_IMMUNE_SYSTEM		
90678	LRSAM1	leucine rich repeat and sterile alpha motif containing 1			REACTOME_IMMUNE_SYSTEM					REACTOME_ADAPTIVE_IMMUNE_SYSTEM		
93611	FBXO44	F-box protein 44			REACTOME_IMMUNE_SYSTEM					REACTOME_ADAPTIVE_IMMUNE_SYSTEM		
9730	VPRBP	Vpr (HIV-1) binding protein			REACTOME_IMMUNE_SYSTEM					REACTOME_ADAPTIVE_IMMUNE_SYSTEM		
164	AP1G1	adaptor-related protein complex 1, gamma 1 subunit			REACTOME_IMMUNE_SYSTEM					REACTOME_ADAPTIVE_IMMUNE_SYSTEM		
8905	AP1S2	adaptor-related protein complex 1, sigma 2 subunit			REACTOME_IMMUNE_SYSTEM					REACTOME_ADAPTIVE_IMMUNE_SYSTEM		
3932	LCK	lymphocyte-specific protein tyrosine kinase			REACTOME_IMMUNE_SYSTEM					REACTOME_ADAPTIVE_IMMUNE_SYSTEM		
5476	CTSA	cathepsin A			REACTOME_IMMUNE_SYSTEM					REACTOME_ADAPTIVE_IMMUNE_SYSTEM		
6885	MAP3K7	mitogen-activated protein kinase kinase kinase 7			REACTOME_IMMUNE_SYSTEM					REACTOME_ADAPTIVE_IMMUNE_SYSTEM		
3265	HRAS	v-Ha-ras Harvey rat sarcoma viral oncogene homolog			REACTOME_IMMUNE_SYSTEM					REACTOME_ADAPTIVE_IMMUNE_SYSTEM		
5894	RAF1	v-raf-1 murine leukemia viral oncogene homolog 1			REACTOME_IMMUNE_SYSTEM					REACTOME_ADAPTIVE_IMMUNE_SYSTEM		
6199	RPS6KB2	ribosomal protein S6 kinase, 70kDa, polypeptide 2			REACTOME_IMMUNE_SYSTEM					REACTOME_ADAPTIVE_IMMUNE_SYSTEM		
5336	PLCG2	phospholipase C, gamma 2 (phosphatidylinositol-specific)			REACTOME_IMMUNE_SYSTEM					REACTOME_ADAPTIVE_IMMUNE_SYSTEM		
1785	DNM2	dynamin 2			REACTOME_IMMUNE_SYSTEM					REACTOME_ADAPTIVE_IMMUNE_SYSTEM		
79109	MAPKAP1	mitogen-activated protein kinase associated protein 1			REACTOME_IMMUNE_SYSTEM					REACTOME_ADAPTIVE_IMMUNE_SYSTEM		
3709	ITPR2	inositol 1,4,5-trisphosphate receptor, type 2			REACTOME_IMMUNE_SYSTEM					REACTOME_ADAPTIVE_IMMUNE_SYSTEM		
10000	AKT3	v-akt murine thymoma viral oncogene homolog 3 (protein kinase B, gamma)			REACTOME_IMMUNE_SYSTEM					REACTOME_ADAPTIVE_IMMUNE_SYSTEM		
64223	MLST8	MTOR associated protein, LST8 homolog (S. cerevisiae)			REACTOME_IMMUNE_SYSTEM					REACTOME_ADAPTIVE_IMMUNE_SYSTEM		
973	CD79A	CD79a molecule, immunoglobulin-associated alpha			REACTOME_IMMUNE_SYSTEM					REACTOME_ADAPTIVE_IMMUNE_SYSTEM		
5788	PTPRC	protein tyrosine phosphatase, receptor type, C			REACTOME_IMMUNE_SYSTEM					REACTOME_ADAPTIVE_IMMUNE_SYSTEM		
5908	RAP1B	RAP1B, member of RAS oncogene family			REACTOME_IMMUNE_SYSTEM					REACTOME_ADAPTIVE_IMMUNE_SYSTEM		
120425	AMICA1	adhesion molecule, interacts with CXADR antigen 1			REACTOME_IMMUNE_SYSTEM					REACTOME_ADAPTIVE_IMMUNE_SYSTEM		
972	CD74	CD74 molecule, major histocompatibility complex, class II invariant chain			REACTOME_IMMUNE_SYSTEM					REACTOME_ADAPTIVE_IMMUNE_SYSTEM		
3108	HLA-DMA	major histocompatibility complex, class II, DM alpha			REACTOME_IMMUNE_SYSTEM					REACTOME_ADAPTIVE_IMMUNE_SYSTEM		
3695	ITGB7	integrin, beta 7			REACTOME_IMMUNE_SYSTEM					REACTOME_ADAPTIVE_IMMUNE_SYSTEM		
3111	HLA-DOA	major histocompatibility complex, class II, DO alpha			REACTOME_IMMUNE_SYSTEM					REACTOME_ADAPTIVE_IMMUNE_SYSTEM		
1783	DYNC1LI2	dynein, cytoplasmic 1, light intermediate chain 2			REACTOME_IMMUNE_SYSTEM					REACTOME_ADAPTIVE_IMMUNE_SYSTEM		
10120	ACTR1B	ARP1 actin-related protein 1 homolog B, centractin beta (yeast)			REACTOME_IMMUNE_SYSTEM					REACTOME_ADAPTIVE_IMMUNE_SYSTEM		
3385	ICAM3	intercellular adhesion molecule 3			REACTOME_IMMUNE_SYSTEM					REACTOME_ADAPTIVE_IMMUNE_SYSTEM		
2317	FLNB	filamin B, beta			REACTOME_IMMUNE_SYSTEM							
3636	INPPL1	inositol polyphosphate phosphatase-like 1			REACTOME_IMMUNE_SYSTEM							
5595	MAPK3	mitogen-activated protein kinase 3			REACTOME_IMMUNE_SYSTEM							
5606	MAP2K3	mitogen-activated protein kinase kinase 3			REACTOME_IMMUNE_SYSTEM							
5599	MAPK8	mitogen-activated protein kinase 8			REACTOME_IMMUNE_SYSTEM							
2002	ELK1	ELK1, member of ETS oncogene family			REACTOME_IMMUNE_SYSTEM							
4205	MEF2A	myocyte enhancer factor 2A			REACTOME_IMMUNE_SYSTEM							
1386	ATF2	activating transcription factor 2			REACTOME_IMMUNE_SYSTEM							
6300	MAPK12	mitogen-activated protein kinase 12			REACTOME_IMMUNE_SYSTEM							
10392	NOD1	nucleotide-binding oligomerization domain containing 1			REACTOME_IMMUNE_SYSTEM							
51135	IRAK4	interleukin-1 receptor-associated kinase 4			REACTOME_IMMUNE_SYSTEM							
351	APP	amyloid beta (A4) precursor protein			REACTOME_IMMUNE_SYSTEM							
51295	ECSIT	ECSIT homolog (Drosophila)			REACTOME_IMMUNE_SYSTEM							
3146	HMGB1	high mobility group box 1			REACTOME_IMMUNE_SYSTEM							
177	AGER	advanced glycosylation end product-specific receptor			REACTOME_IMMUNE_SYSTEM							
23643	LY96	lymphocyte antigen 96			REACTOME_IMMUNE_SYSTEM							
3665	IRF7	interferon regulatory factor 7			REACTOME_IMMUNE_SYSTEM							
10695	CNPY3	canopy 3 homolog (zebrafish)			REACTOME_IMMUNE_SYSTEM							
8672	EIF4G3	eukaryotic translation initiation factor 4 gamma, 3			REACTOME_IMMUNE_SYSTEM							
6868	ADAM17	ADAM metallopeptidase domain 17			REACTOME_IMMUNE_SYSTEM							
51447	IP6K2	inositol hexakisphosphate kinase 2			REACTOME_IMMUNE_SYSTEM							
6773	STAT2	signal transducer and activator of transcription 2, 113kDa			REACTOME_IMMUNE_SYSTEM							
5771	PTPN2	protein tyrosine phosphatase, non-receptor type 2			REACTOME_IMMUNE_SYSTEM							
7297	TYK2	tyrosine kinase 2			REACTOME_IMMUNE_SYSTEM							
4261	CIITA	class II, major histocompatibility complex, transactivator			REACTOME_IMMUNE_SYSTEM							
3454	IFNAR1	interferon (alpha, beta and omega) receptor 1			REACTOME_IMMUNE_SYSTEM							
25970	SH2B1	SH2B adaptor protein 1			REACTOME_IMMUNE_SYSTEM							
3718	JAK3	Janus kinase 3			REACTOME_IMMUNE_SYSTEM							
1439	CSF2RB	colony stimulating factor 2 receptor, beta, low-affinity (granulocyte-macrophage)			REACTOME_IMMUNE_SYSTEM							
3556	IL1RAP	interleukin 1 receptor accessory protein			REACTOME_IMMUNE_SYSTEM							
841	CASP8	caspase 8, apoptosis-related cysteine peptidase			REACTOME_IMMUNE_SYSTEM							
1540	CYLD	cylindromatosis (turban tumor syndrome)			REACTOME_IMMUNE_SYSTEM							
79132	DHX58	DEXH (Asp-Glu-X-His) box polypeptide 58			REACTOME_IMMUNE_SYSTEM							
57506	MAVS	mitochondrial antiviral signaling protein			REACTOME_IMMUNE_SYSTEM							
55593	OTUD5	OTU domain containing 5			REACTOME_IMMUNE_SYSTEM							
22861	NLRP1	NLR family, pyrin domain containing 1			REACTOME_IMMUNE_SYSTEM							
4179	CD46	CD46 molecule, complement regulatory protein			REACTOME_IMMUNE_SYSTEM							
6188	RPS3	ribosomal protein S3						REACTOME_METABOLISM_OF_PROTEINS	REACTOME_METABOLISM_OF_RNA			
6146	RPL22	ribosomal protein L22						REACTOME_METABOLISM_OF_PROTEINS	REACTOME_METABOLISM_OF_RNA			
6164	RPL34	ribosomal protein L34						REACTOME_METABOLISM_OF_PROTEINS	REACTOME_METABOLISM_OF_RNA			
6175	RPLP0	ribosomal protein, large, P0						REACTOME_METABOLISM_OF_PROTEINS	REACTOME_METABOLISM_OF_RNA			
6135	RPL11	ribosomal protein L11						REACTOME_METABOLISM_OF_PROTEINS	REACTOME_METABOLISM_OF_RNA			
6203	RPS9	ribosomal protein S9						REACTOME_METABOLISM_OF_PROTEINS	REACTOME_METABOLISM_OF_RNA			
6223	RPS19	ribosomal protein S19						REACTOME_METABOLISM_OF_PROTEINS	REACTOME_METABOLISM_OF_RNA			
3921	RPSA	ribosomal protein SA						REACTOME_METABOLISM_OF_PROTEINS	REACTOME_METABOLISM_OF_RNA			
6205	RPS11	ribosomal protein S11						REACTOME_METABOLISM_OF_PROTEINS	REACTOME_METABOLISM_OF_RNA			
6209	RPS15	ribosomal protein S15						REACTOME_METABOLISM_OF_PROTEINS	REACTOME_METABOLISM_OF_RNA			
6222	RPS18	ribosomal protein S18						REACTOME_METABOLISM_OF_PROTEINS	REACTOME_METABOLISM_OF_RNA			
6229	RPS24	ribosomal protein S24						REACTOME_METABOLISM_OF_PROTEINS	REACTOME_METABOLISM_OF_RNA			
6234	RPS28	ribosomal protein S28						REACTOME_METABOLISM_OF_PROTEINS	REACTOME_METABOLISM_OF_RNA			
6124	RPL4	ribosomal protein L4						REACTOME_METABOLISM_OF_PROTEINS	REACTOME_METABOLISM_OF_RNA			
6134	RPL10	ribosomal protein L10						REACTOME_METABOLISM_OF_PROTEINS	REACTOME_METABOLISM_OF_RNA			
6136	RPL12	ribosomal protein L12						REACTOME_METABOLISM_OF_PROTEINS	REACTOME_METABOLISM_OF_RNA			
6139	RPL17	ribosomal protein L17						REACTOME_METABOLISM_OF_PROTEINS	REACTOME_METABOLISM_OF_RNA			
6142	RPL18A	ribosomal protein L18a						REACTOME_METABOLISM_OF_PROTEINS	REACTOME_METABOLISM_OF_RNA			
6157	RPL27A	ribosomal protein L27a						REACTOME_METABOLISM_OF_PROTEINS	REACTOME_METABOLISM_OF_RNA			
6169	RPL38	ribosomal protein L38						REACTOME_METABOLISM_OF_PROTEINS	REACTOME_METABOLISM_OF_RNA			
6171	RPL41	ribosomal protein L41						REACTOME_METABOLISM_OF_PROTEINS	REACTOME_METABOLISM_OF_RNA			
10528	NOP56	NOP56 ribonucleoprotein homolog (yeast)						REACTOME_METABOLISM_OF_PROTEINS			HALLMARK_E2F_TARGETS	
6904	TBCD	tubulin folding cofactor D						REACTOME_METABOLISM_OF_PROTEINS				
8662	EIF3B	eukaryotic translation initiation factor 3, subunit B						REACTOME_METABOLISM_OF_PROTEINS				
10575	CCT4	chaperonin containing TCP1, subunit 4 (delta)						REACTOME_METABOLISM_OF_PROTEINS				
6950	TCP1	t-complex 1						REACTOME_METABOLISM_OF_PROTEINS				
3646	EIF3E	eukaryotic translation initiation factor 3, subunit E						REACTOME_METABOLISM_OF_PROTEINS				
27335	EIF3K	eukaryotic translation initiation factor 3, subunit K						REACTOME_METABOLISM_OF_PROTEINS				
8666	EIF3G	eukaryotic translation initiation factor 3, subunit G						REACTOME_METABOLISM_OF_PROTEINS				
8668	EIF3I	eukaryotic translation initiation factor 3, subunit I						REACTOME_METABOLISM_OF_PROTEINS				
6185	RPN2	ribophorin II						REACTOME_METABOLISM_OF_PROTEINS				
90701	SEC11C	SEC11 homolog C (S. cerevisiae)						REACTOME_METABOLISM_OF_PROTEINS				
6747	SSR3	signal sequence receptor, gamma (translocon-associated protein gamma)						REACTOME_METABOLISM_OF_PROTEINS				
6726	SRP9	signal recognition particle 9kDa						REACTOME_METABOLISM_OF_PROTEINS				
1936	EEF1D	eukaryotic translation elongation factor 1 delta (guanine nucleotide exchange protein)						REACTOME_METABOLISM_OF_PROTEINS				
10063	COX17	COX17 cytochrome c oxidase assembly homolog (S. cerevisiae)						REACTOME_METABOLISM_OF_PROTEINS				
410	ARSA	arylsulfatase A						REACTOME_METABOLISM_OF_PROTEINS				
285362	SUMF1	sulfatase modifying factor 1						REACTOME_METABOLISM_OF_PROTEINS				
25870	SUMF2	sulfatase modifying factor 2						REACTOME_METABOLISM_OF_PROTEINS				
22803	XRN2	5'-3' exoribonuclease 2						REACTOME_METABOLISM_OF_PROTEINS				
10113	PREB	prolactin regulatory element binding						REACTOME_METABOLISM_OF_PROTEINS				
4351	MPI	mannose phosphate isomerase						REACTOME_METABOLISM_OF_PROTEINS				
29925	GMPPB	GDP-mannose pyrophosphorylase B						REACTOME_METABOLISM_OF_PROTEINS				
8703	B4GALT3	UDP-Gal:betaGlcNAc beta 1,4- galactosyltransferase, polypeptide 3						REACTOME_METABOLISM_OF_PROTEINS				
6482	ST3GAL1	ST3 beta-galactoside alpha-2,3-sialyltransferase 1						REACTOME_METABOLISM_OF_PROTEINS				
29880	ALG5	asparagine-linked glycosylation 5, dolichyl-phosphate beta-glucosyltransferase homolog (S. cerevisiae)						REACTOME_METABOLISM_OF_PROTEINS				
10195	ALG3	asparagine-linked glycosylation 3, alpha-1,3- mannosyltransferase homolog (S. cerevisiae)						REACTOME_METABOLISM_OF_PROTEINS				
79868	ALG13	asparagine-linked glycosylation 13 homolog (S. cerevisiae)						REACTOME_METABOLISM_OF_PROTEINS				
84920	ALG10	asparagine-linked glycosylation 10, alpha-1,2-glucosyltransferase homolog (S. pombe)						REACTOME_METABOLISM_OF_PROTEINS				
6480	ST6GAL1	ST6 beta-galactosamide alpha-2,6-sialyltranferase 1						REACTOME_METABOLISM_OF_PROTEINS				
55741	EDEM2	ER degradation enhancer, mannosidase alpha-like 2						REACTOME_METABOLISM_OF_PROTEINS				
56886	UGGT1	UDP-glucose glycoprotein glucosyltransferase 1						REACTOME_METABOLISM_OF_PROTEINS				
23556	PIGN	phosphatidylinositol glycan anchor biosynthesis, class N						REACTOME_METABOLISM_OF_PROTEINS				
51227	PIGP	phosphatidylinositol glycan anchor biosynthesis, class P						REACTOME_METABOLISM_OF_PROTEINS				
5277	PIGA	phosphatidylinositol glycan anchor biosynthesis, class A						REACTOME_METABOLISM_OF_PROTEINS				
5281	PIGF	phosphatidylinositol glycan anchor biosynthesis, class F						REACTOME_METABOLISM_OF_PROTEINS				
9487	PIGL	phosphatidylinositol glycan anchor biosynthesis, class L						REACTOME_METABOLISM_OF_PROTEINS				
51809	GALNT7	UDP-N-acetyl-alpha-D-galactosamine:polypeptide N-acetylgalactosaminyltransferase 7 (GalNAc-T7)						REACTOME_METABOLISM_OF_PROTEINS				
79695	GALNT12	UDP-N-acetyl-alpha-D-galactosamine:polypeptide N-acetylgalactosaminyltransferase 12 (GalNAc-T12)						REACTOME_METABOLISM_OF_PROTEINS				
146712	B3GNTL1	UDP-GlcNAc:betaGal beta-1,3-N-acetylglucosaminyltransferase-like 1						REACTOME_METABOLISM_OF_PROTEINS				
1431	CS	citrate synthase						REACTOME_METABOLISM_OF_PROTEINS				
10564	ARFGEF2	ADP-ribosylation factor guanine nucleotide-exchange factor 2 (brefeldin A-inhibited)						REACTOME_METABOLISM_OF_PROTEINS				
26224	FBXL3	F-box and leucine-rich repeat protein 3						REACTOME_METABOLISM_OF_PROTEINS				
10694	CCT8	chaperonin containing TCP1, subunit 8 (theta)						REACTOME_METABOLISM_OF_PROTEINS				
5204	PFDN5	prefoldin subunit 5						REACTOME_METABOLISM_OF_PROTEINS				
51025	PAM16	presequence translocase-associated motor 16 homolog (S. cerevisiae)						REACTOME_METABOLISM_OF_PROTEINS				
617	BCS1L	BCS1-like (S. cerevisiae)						REACTOME_METABOLISM_OF_PROTEINS				
3184	HNRNPD	heterogeneous nuclear ribonucleoprotein D (AU-rich element RNA binding protein 1, 37kDa)							REACTOME_METABOLISM_OF_RNA		HALLMARK_E2F_TARGETS	HALLMARK_G2M_CHECKPOINT
6634	SNRPD3	small nuclear ribonucleoprotein D3 polypeptide 18kDa							REACTOME_METABOLISM_OF_RNA			
23658	LSM5	LSM5 homolog, U6 small nuclear RNA associated (S. cerevisiae)							REACTOME_METABOLISM_OF_RNA			
65110	UPF3A	UPF3 regulator of nonsense transcripts homolog A (yeast)							REACTOME_METABOLISM_OF_RNA			
9887	SMG7	smg-7 homolog, nonsense mediated mRNA decay factor (C. elegans)							REACTOME_METABOLISM_OF_RNA			
167227	DCP2	DCP2 decapping enzyme homolog (S. cerevisiae)							REACTOME_METABOLISM_OF_RNA			
22894	DIS3	DIS3 mitotic control homolog (S. cerevisiae)							REACTOME_METABOLISM_OF_RNA			
51010	EXOSC3	exosome component 3							REACTOME_METABOLISM_OF_RNA			
57472	CNOT6	CCR4-NOT transcription complex, subunit 6							REACTOME_METABOLISM_OF_RNA			
25904	CNOT10	CCR4-NOT transcription complex, subunit 10							REACTOME_METABOLISM_OF_RNA			
219988	PATL1	protein associated with topoisomerase II homolog 1 (yeast)							REACTOME_METABOLISM_OF_RNA			
80153	EDC3	enhancer of mRNA decapping 3 homolog (S. cerevisiae)							REACTOME_METABOLISM_OF_RNA			
1062	CENPE	centromere protein E, 312kDa									HALLMARK_E2F_TARGETS	HALLMARK_G2M_CHECKPOINT
6427	SRSF2	serine/arginine-rich splicing factor 2									HALLMARK_E2F_TARGETS	HALLMARK_G2M_CHECKPOINT
6434	TRA2B	transformer 2 beta homolog (Drosophila)									HALLMARK_E2F_TARGETS	HALLMARK_G2M_CHECKPOINT
3161	HMMR	hyaluronan-mediated motility receptor (RHAMM)									HALLMARK_E2F_TARGETS	HALLMARK_G2M_CHECKPOINT
4678	NASP	nuclear autoantigenic sperm protein (histone-binding)									HALLMARK_E2F_TARGETS	HALLMARK_G2M_CHECKPOINT
3609	ILF3	interleukin enhancer binding factor 3, 90kDa									HALLMARK_E2F_TARGETS	HALLMARK_G2M_CHECKPOINT
7112	TMPO	thymopoietin									HALLMARK_E2F_TARGETS	HALLMARK_G2M_CHECKPOINT
9787	DLGAP5	discs, large (Drosophila) homolog-associated protein 5									HALLMARK_E2F_TARGETS	
2547	XRCC6	X-ray repair complementing defective repair in Chinese hamster cells 6									HALLMARK_E2F_TARGETS	
4673	NAP1L1	nucleosome assembly protein 1-like 1									HALLMARK_E2F_TARGETS	
7037	TFRC	transferrin receptor (p90, CD71)									HALLMARK_E2F_TARGETS	
5591	PRKDC	protein kinase, DNA-activated, catalytic polypeptide									HALLMARK_E2F_TARGETS	
9738	CCP110	centriolar coiled coil protein 110kDa									HALLMARK_E2F_TARGETS	
9918	NCAPD2	non-SMC condensin I complex, subunit D2									HALLMARK_E2F_TARGETS	

3148	HMGB2	high mobility group box 2									HALLMARK_E2F_TARGETS	
8914	TIMELESS	timeless homolog (Drosophila)									HALLMARK_E2F_TARGETS	
9238	TBRG4	transforming growth factor beta regulator 4									HALLMARK_E2F_TARGETS	
146909	KIF18B	kinesin family member 18B									HALLMARK_E2F_TARGETS	
197335	WDR90	WD repeat domain 90									HALLMARK_E2F_TARGETS	
29893	PSMC3IP	PSMC3 interacting protein									HALLMARK_E2F_TARGETS	
29980	DONSON	downstream neighbor of SON									HALLMARK_E2F_TARGETS	
64858	DCLRE1B	DNA cross-link repair 1B									HALLMARK_E2F_TARGETS	
6941	TCF19	transcription factor 19									HALLMARK_E2F_TARGETS	
83461	CDCA3	cell division cycle associated 3									HALLMARK_E2F_TARGETS	
84312	BRMS1L	breast cancer metastasis-suppressor 1-like									HALLMARK_E2F_TARGETS	
9833	MELK	maternal embryonic leucine zipper kinase									HALLMARK_E2F_TARGETS	
9924	PAN2	PAN2 poly(A) specific ribonuclease subunit homolog (S. cerevisiae)									HALLMARK_E2F_TARGETS	
6599	SMARCC1	SWI/SNF related, matrix associated, actin dependent regulator of chromatin, subfamily c, member 1										HALLMARK_G2M_CHECKPOINT
4957	ODF2	outer dense fiber of sperm tails 2										HALLMARK_G2M_CHECKPOINT
7040	TGFB1	transforming growth factor, beta 1										HALLMARK_G2M_CHECKPOINT
10772	SRSF10	serine/arginine-rich splicing factor 10										HALLMARK_G2M_CHECKPOINT
2130	EWSR1	Ewing sarcoma breakpoint region 1										HALLMARK_G2M_CHECKPOINT
7090	TLE3	transducin-like enhancer of split 3 (E(sp1) homolog, Drosophila)										HALLMARK_G2M_CHECKPOINT
387893	SETD8	SET domain containing (lysine methyltransferase) 8										HALLMARK_G2M_CHECKPOINT
6558	SLC12A2	solute carrier family 12 (sodium/potassium/chloride transporters), member 2										HALLMARK_G2M_CHECKPOINT
81539	SLC38A1	solute carrier family 38, member 1										HALLMARK_G2M_CHECKPOINT
1756	DMD	dystrophin										HALLMARK_G2M_CHECKPOINT
9156	EXO1	exonuclease 1										HALLMARK_G2M_CHECKPOINT
9994	CASP8AP2	caspase 8 associated protein 2										HALLMARK_G2M_CHECKPOINT
546	ATRX	alpha thalassemia/mental retardation syndrome X-linked										HALLMARK_G2M_CHECKPOINT
6491	STIL	SCL/TAL1 interrupting locus										HALLMARK_G2M_CHECKPOINT
94239	H2AFV	H2A histone family, member V										HALLMARK_G2M_CHECKPOINT
10721	POLQ	polymerase (DNA directed), theta										HALLMARK_G2M_CHECKPOINT
22823	MTF2	metal response element binding transcription factor 2										HALLMARK_G2M_CHECKPOINT
30000	TNPO2	transportin 2										HALLMARK_G2M_CHECKPOINT
4928	NUP98	nucleoporin 98kDa										HALLMARK_G2M_CHECKPOINT
6760	SS18	synovial sarcoma translocation, chromosome 18										HALLMARK_G2M_CHECKPOINT
8899	PRPF4B	PRP4 pre-mRNA processing factor 4 homolog B (yeast)										HALLMARK_G2M_CHECKPOINT
2648	KAT2A	K(lysine) acetyltransferase 2A										
905	CCNT2	cyclin T2										
1024	CDK8	cyclin-dependent kinase 8										
9612	NCOR2	nuclear receptor corepressor 2										
10025	MED16	mediator complex subunit 16										
9282	MED14	mediator complex subunit 14										
9439	MED23	mediator complex subunit 23										
4087	SMAD2	SMAD family member 2										
7050	TGIF1	TGFB-induced factor homeobox 1										
5914	RARA	retinoic acid receptor, alpha										
10782	ZNF274	zinc finger protein 274										
126382	NR2C2AP	nuclear receptor 2C2-associated protein										
10308	ZNF267	zinc finger protein 267										
10781	ZNF266	zinc finger protein 266										
121274	ZNF641	zinc finger protein 641										
146198	ZFP90	zinc finger protein 90 homolog (mouse)										
147657	ZNF480	zinc finger protein 480										
147686	ZNF418	zinc finger protein 418										
147949	ZNF583	zinc finger protein 583										
148156	ZNF558	zinc finger protein 558										
162655	ZNF519	zinc finger protein 519										
162963	ZNF610	zinc finger protein 610										
162967	ZNF320	zinc finger protein 320										
163087	ZNF383	zinc finger protein 383										
170959	ZNF431	zinc finger protein 431										
201514	ZNF584	zinc finger protein 584										
22869	ZNF510	zinc finger protein 510										
23660	ZKSCAN5	zinc finger with KRAB and SCAN domains 5										
27300	ZNF544	zinc finger protein 544										
284307	ZIK1	zinc finger protein interacting with K protein 1 homolog (mouse)										
284443	ZNF493	zinc finger protein 493										
339327	ZNF546	zinc finger protein 546										
348327	ZNF530	zinc finger protein 530										
51276	ZNF571	zinc finger protein 571										
55786	ZNF415	zinc finger protein 415										
57693	ZNF317	zinc finger protein 317										
57711	ZNF529	zinc finger protein 529										
7551	ZNF3	zinc finger protein 3										
7562	ZNF708	zinc finger protein 708										
7571	ZNF23	zinc finger protein 23 (KOX 16)										
7574	ZNF26	zinc finger protein 26										
7576	ZNF28	zinc finger protein 28										
7581	ZNF33A	zinc finger protein 33A										
7582	ZNF33B	zinc finger protein 33B										
7587	ZNF37A	zinc finger protein 37A										
7697	ZNF138	zinc finger protein 138										
7733	ZNF180	zinc finger protein 180										
7753	ZNF202	zinc finger protein 202										
7769	ZNF226	zinc finger protein 226										
7770	ZNF227	zinc finger protein 227										
79175	ZNF343	zinc finger protein 343										
79818	ZNF552	zinc finger protein 552										
92285	ZNF585B	zinc finger protein 585B										
9534	ZNF254	zinc finger protein 254										
10128	LRPPRC	leucine-rich PPR-motif containing										
23365	ARHGEF12	Rho guanine nucleotide exchange factor (GEF) 12										
9181	ARHGEF2	Rho/Rac guanine nucleotide exchange factor (GEF) 2										
393	ARHGAP4	Rho GTPase activating protein 4										
64857	PLEKHG2	pleckstrin homology domain containing, family G (with RhoGef domain) member 2										
6709	SPTAN1	spectrin, alpha, non-erythrocytic 1 (alpha-fodrin)										
54509	RHOF	ras homolog gene family, member F (in filopodia)										
9344	TAOK2	TAO kinase 2										
2037	EPB41L2	erythrocyte membrane protein band 4.1-like 2										
5829	PXN	paxillin										
2035	EPB41	erythrocyte membrane protein band 4.1 (elliptocytosis 1, RH-linked)										
23095	KIF1B	kinesin family member 1B										
8409	UXT	ubiquitously-expressed transcript										
3996	LLGL1	lethal giant larvae homolog 1 (Drosophila)										
85464	SSH2	slingshot homolog 2 (Drosophila)										
51199	NIN	ninein (GSK3B interacting protein)										
23607	CD2AP	CD2-associated protein										
116840	CNTROB	centrobin, centrosomal BRCA2 interacting protein										
163786	SASS6	spindle assembly 6 homolog (C. elegans)										
5311	PKD2	polycystic kidney disease 2 (autosomal dominant)										
55704	CCDC88A	coiled-coil domain containing 88A										
790	CAD	carbamoyl-phosphate synthetase 2, aspartate transcarbamylase, and dihydroorotase										
8886	DDX18	DEAD (Asp-Glu-Ala-Asp) box polypeptide 18										
5478	PPIA	peptidylprolyl isomerase A (cyclophilin A)										
3183	HNRNPC	heterogeneous nuclear ribonucleoprotein C (C1/C2)										
6432	SRSF7	serine/arginine-rich splicing factor 7										
10907	TXNL4A	thioredoxin-like 4A										
3181	HNRNPA2B1	heterogeneous nuclear ribonucleoprotein A2/B1										
3615	IMPDH2	IMP (inosine 5'-monophosphate) dehydrogenase 2										
2058	EPRS	glutamyl-prolyl-tRNA synthetase										
10987	COPS5	COP9 constitutive photomorphogenic homolog subunit 5 (Arabidopsis)										
10399	GNB2L1	guanine nucleotide binding protein (G protein), beta polypeptide 2-like 1										
11331	PHB2	prohibitin 2										
11260	XPOT	exportin, tRNA (nuclear export receptor for tRNAs)										
7555	CNBP	CCHC-type zinc finger, nucleic acid binding protein										
7965	AIMP2	aminoacyl tRNA synthetase complex-interacting multifunctional protein 2										
708	C1QBP	complement component 1, q subcomponent binding protein										
6742	SSBP1	single-stranded DNA binding protein 1										
8761	PABPC4	poly(A) binding protein, cytoplasmic 4 (inducible form)										
6880	TAF9	TAF9 RNA polymerase II, TATA box binding protein (TBP)-associated factor, 32kDa										
1104	RCC1	regulator of chromosome condensation 1										
6875	TAF4B	TAF4b RNA polymerase II, TATA box binding protein (TBP)-associated factor, 105kDa										
581	BAX	BCL2-associated X protein										
5213	PFKM	phosphofructokinase, muscle										
8295	TRRAP	transformation/transcription domain-associated protein										
9913	SUPT7L	suppressor of Ty 7 (S. cerevisiae)-like										
5757	PTMA	prothymosin, alpha										
83879	CDCA7	cell division cycle associated 7										
84864	MINA	MYC induced nuclear antigen										
92140	MTDH	metadherin										
200916	RPL22L1	ribosomal protein L22-like 1										
1643	DDB2	damage-specific DNA binding protein 2, 48kDa										
84447	SYVN1	synovial apoptosis inhibitor 1, synoviolin										
8916	HERC3	hect domain and RLD 3										
11060	WWP2	WW domain containing E3 ubiquitin protein ligase 2										
26091	HERC4	hect domain and RLD 4										
51366	UBR5	ubiquitin protein ligase E3 component n-recognin 5										
57448	BIRC6	baculoviral IAP repeat containing 6										
2963	GTF2F2	general transcription factor IIF, polypeptide 2, 30kDa										
2071	ERCC3	excision repair cross-complementing rodent repair deficiency, complementation group 3 (xeroderma pigmentosum group B complementing)										
8731	RNMT	RNA (guanine-7-) methyltransferase										
2968	GTF2H4	general transcription factor IIH, polypeptide 4, 52kDa										
6878	TAF6	TAF6 RNA polymerase II, TATA box binding protein (TBP)-associated factor, 80kDa										
51160	VPS28	vacuolar protein sorting 28 homolog (S. cerevisiae)										
6872	TAF1	TAF1 RNA polymerase II, TATA box binding protein (TBP)-associated factor, 250kDa										
7520	XRCC5	X-ray repair complementing defective repair in Chinese hamster cells 5 (double-strand-break rejoining)										
6431	SRSF6	serine/arginine-rich splicing factor 6										
6430	SRSF5	serine/arginine-rich splicing factor 5										
9785	DHX38	DEAH (Asp-Glu-Ala-His) box polypeptide 38										
29894	CPSF1	cleavage and polyadenylation specific factor 1, 160kDa										
10594	PRPF8	PRP8 pre-mRNA processing factor 8 homolog (S. cerevisiae)										
4670	HNRNPM	heterogeneous nuclear ribonucleoprotein M										
1477	CSTF1	cleavage stimulation factor, 3' pre-RNA, subunit 1, 50kDa										
2521	FUS	fused in sarcoma										
79869	CPSF7	cleavage and polyadenylation specific factor 7, 59kDa										
3187	HNRNPH1	heterogeneous nuclear ribonucleoprotein H1 (H)										
55749	CCAR1	cell division cycle and apoptosis regulator 1										
56339	METTL3	methyltransferase like 3										
104	ADARB1	adenosine deaminase, RNA-specific, B1										
5611	DNAJC3	DnaJ (Hsp40) homolog, subfamily C, member 3										
5888	RAD51	RAD51 homolog (S. cerevisiae)										
5893	RAD52	RAD52 homolog (S. cerevisiae)										
27343	POLL	polymerase (DNA directed), lambda										
30833	NT5C	5', 3'-nucleotidase, cytosolic										
4833	NME4	non-metastatic cells 4, protein expressed in										
1716	DGUOK	deoxyguanosine kinase										
51292	GMPR2	guanosine monophosphate reductase 2										
2987	GUK1	guanylate kinase 1										
124583	CANT1	calcium activated nucleotidase 1										
30834	ZNRD1	zinc ribbon domain containing 1										
9013	TAF1C	TATA box binding protein (TBP)-associated factor, RNA polymerase I, C, 110kDa										
10302	SNAPC5	small nuclear RNA activating complex, polypeptide 5, 19kDa										
6895	TARBP2	TAR (HIV-1) RNA binding protein 2										
23309	SIN3B	SIN3 homolog B, transcription regulator (yeast)										
7296	TXNRD1	thioredoxin reductase 1										
79071	ELOVL6	ELOVL fatty acid elongase 6										
3156	HMGCR	3-hydroxy-3-methylglutaryl-CoA reductase										
5660	PSAP	prosaposin										
2629	GBA	glucosidase, beta, acid										
200576	PIKFYVE	phosphoinositide kinase, FYVE finger containing										
81579	PLA2G12A	phospholipase A2, group XIIA										
37	ACADVL	acyl-CoA dehydrogenase, very long chain										
30	ACAA1	acetyl-CoA acyltransferase 1										
1666	DECR1	2,4-dienoyl CoA reductase 1, mitochondrial										
51703	ACSL5	acyl-CoA synthetase long-chain family member 5										
3295	HSD17B4	hydroxysteroid (17-beta) dehydrogenase 4										
8443	GNPAT	glyceronephosphate O-acyltransferase										
2224	FDPS	farnesyl diphosphate synthase										
4597	MVD	mevalonate (diphospho) decarboxylase										
6576	SLC25A1	solute carrier family 25 (mitochondrial carrier; citrate transporter), member 1										
23175	LPIN1	lipin 1										
8694	DGAT1	diacylglycerol O-acyltransferase 1										
253558	LCLAT1	lysocardiolipin acyltransferase 1										
23389	MED13L	mediator complex subunit 13-like										
6837	MED22	mediator complex subunit 22										
2222	FDFT1	farnesyl-diphosphate farnesyltransferase 1										
9524	TECR	trans-2,3-enoyl-CoA reductase										
1375	CPT1B	carnitine palmitoyltransferase 1B (muscle)										
133522	PPARGC1B	peroxisome proliferator-activated receptor gamma, coactivator 1 beta										
622	BDH1	3-hydroxybutyrate dehydrogenase, type 1										
3339	HSPG2	heparan sulfate proteoglycan 2										
23396	PIP5K1C	phosphatidylinositol-4-phosphate 5-kinase, type I, gamma										
259230	SGMS1	sphingomyelin synthase 1										
10162	LPCAT3	lysophosphatidylcholine acyltransferase 3										
55627	SMPD4	sphingomyelin phosphodiesterase 4, neutral membrane (neutral sphingomyelinase-3)										
57704	GBA2	glucosidase, beta (bile acid) 2										
5298	PI4KB	phosphatidylinositol 4-kinase, catalytic, beta										
1120	CHKB	choline kinase beta										
55500	ETNK1	ethanolamine kinase 1										
122618	PLD4	phospholipase D family, member 4										
64419	MTMR14	myotubularin related protein 14										
26061	HACL1	2-hydroxyacyl-CoA lyase 1										
4598	MVK	mevalonate kinase										
3991	LIPE	lipase, hormone-sensitive										
4047	LSS	lanosterol synthase (2,3-oxidosqualene-lanosterol cyclase)										
120227	CYP2R1	cytochrome P450, family 2, subfamily R, polypeptide 1										
5360	PLTP	phospholipid transfer protein										
80765	STARD5	StAR-related lipid transfer (START) domain containing 5										
4659	PPP1R12A	protein phosphatase 1, regulatory subunit 12A										
2801	GOLGA2	golgin A2										
7178	TPT1	tumor protein, translationally-controlled 1										
2189	FANCG	Fanconi anemia, complementation group G										
2175	FANCA	Fanconi anemia, complementation group A										
4913	NTHL1	nth endonuclease III-like 1 (E. coli)										
57697	FANCM	Fanconi anemia, complementation group M										
9656	MDC1	mediator of DNA-damage checkpoint 1										
4595	MUTYH	mutY homolog (E. coli)										
121642	ALKBH2	alkB, alkylation repair homolog 2 (E. coli)										
51455	REV1	REV1 homolog (S. cerevisiae)										
6198	RPS6KB1	ribosomal protein S6 kinase, 70kDa, polypeptide 1										
2232	FDXR	ferredoxin reductase										
440	ASNS	asparagine synthetase (glutamine-hydrolyzing)										
27230	SERP1	stress-associated endoplasmic reticulum protein 1										
226	ALDOA	aldolase A, fructose-bisphosphate										
79602	ADIPOR2	adiponectin receptor 2										
7167	TPI1	triosephosphate isomerase 1										
5033	P4HA1	prolyl 4-hydroxylase, alpha polypeptide I										
5211	PFKL	phosphofructokinase, liver										
3145	HMBS	hydroxymethylbilane synthase										
6533	SLC6A6	solute carrier family 6 (neurotransmitter transporter, taurine), member 6										
5464	PPA1	pyrophosphatase (inorganic) 1										
5723	PSPH	phosphoserine phosphatase										
8553	BHLHE40	basic helix-loop-helix family, member e40										
441024	MTHFD2L	methylenetetrahydrofolate dehydrogenase (NADP+ dependent) 2-like										
120	ADD3	adducin 3 (gamma)										
6472	SHMT2	serine hydroxymethyltransferase 2 (mitochondrial)										
5871	MAP4K2	mitogen-activated protein kinase kinase kinase kinase 2										
4294	MAP3K10	mitogen-activated protein kinase kinase kinase 10										
9448	MAP4K4	mitogen-activated protein kinase kinase kinase kinase 4										
9693	RAPGEF2	Rap guanine nucleotide exchange factor (GEF) 2										
22985	ACIN1	apoptotic chromatin condensation inducer 1										
1655	DDX5	DEAD (Asp-Glu-Ala-Asp) box polypeptide 5										
9129	PRPF3	PRP3 pre-mRNA processing factor 3 homolog (S. cerevisiae)										
10450	PPIE	peptidylprolyl isomerase E (cyclophilin E)										
10523	CHERP	calcium homeostasis endoplasmic reticulum protein										
10713	USP39	ubiquitin specific peptidase 39										
11325	DDX42	DEAD (Asp-Glu-Ala-Asp) box polypeptide 42										
22827	PUF60	poly-U binding splicing factor 60KDa										
29896	TRA2A	transformer 2 alpha homolog (Drosophila)										
55696	RBM22	RNA binding motif protein 22										
9583	ENTPD4	ectonucleoside triphosphate diphosphohydrolase 4										
23457	ABCB9	ATP-binding cassette, sub-family B (MDR/TAP), member 9										
2990	GUSB	glucuronidase, beta										
3423	IDS	iduronate 2-sulfatase										
3920	LAMP2	lysosomal-associated membrane protein 2										
535	ATP6V0A1	ATPase, H+ transporting, lysosomal V0 subunit a1										
1497	CTNS	cystinosin, lysosomal cystine transporter										
10717	AP4B1	adaptor-related protein complex 4, beta 1 subunit										
4126	MANBA	mannosidase, beta A, lysosomal										
23163	GGA3	golgi-associated, gamma adaptin ear containing, ARF binding protein 3										
4668	NAGA	N-acetylgalactosaminidase, alpha-										
1201	CLN3	ceroid-lipofuscinosis, neuronal 3										
256471	MFSD8	major facilitator superfamily domain containing 8										
26088	GGA1	golgi-associated, gamma adaptin ear containing, ARF binding protein 1										
84572	GNPTG	N-acetylglucosamine-1-phosphate transferase, gamma subunit										
23385	NCSTN	nicastrin										
51564	HDAC7	histone deacetylase 7										
9734	HDAC9	histone deacetylase 9										
83933	HDAC10	histone deacetylase 10										
7091	TLE4	transducin-like enhancer of split 4 (E(sp1) homolog, Drosophila)										
8650	NUMB	numb homolog (Drosophila)										
142678	MIB2	mindbomb homolog 2 (Drosophila)										
27327	TNRC6A	trinucleotide repeat containing 6A										
57690	TNRC6C	trinucleotide repeat containing 6C										
56983	POGLUT1	protein O-glucosyltransferase 1										
54884	RETSAT	retinol saturase (all-trans-retinol 13,14-reductase)										
23479	ISCU	iron-sulfur cluster scaffold homolog (E. coli)										
6520	SLC3A2	solute carrier family 3 (activators of dibasic and neutral amino acid transport), member 2										
6303	SAT1	spermidine/spermine N1-acetyltransferase 1										
1606	DGKA	diacylglycerol kinase, alpha 80kDa										
22824	HSPA4L	heat shock 70kDa protein 4-like										
9538	EI24	etoposide induced 2.4 mRNA										
23654	PLXNB2	plexin B2										
51181	DCXR	dicarbonyl/L-xylulose reductase										
10608	MXD4	MAX dimerization protein 4										
30836	DNTTIP2	deoxynucleotidyltransferase, terminal, interacting protein 2										
3094	HINT1	histidine triad nucleotide binding protein 1										
3275	PRMT2	protein arginine methyltransferase 2										
4033	LRMP	lymphoid-restricted membrane protein										
49856	WRAP73	WD repeat containing, antisense to TP73										
51768	TM7SF3	transmembrane 7 superfamily member 3										
5820	PVT1	Pvt1 oncogene (non-protein coding)										
23517	SKIV2L2	superkiller viralicidic activity 2-like 2 (S. cerevisiae)										
5394	EXOSC10	exosome component 10										
23019	CNOT1	CCR4-NOT transcription complex, subunit 1										
64895	PAPOLG	poly(A) polymerase gamma										
6499	SKIV2L	superkiller viralicidic activity 2-like (S. cerevisiae)										
84186	ZCCHC7	zinc finger, CCHC domain containing 7										

814	CAMK4	calcium/calmodulin-dependent protein kinase IV
5781	PTPN11	protein tyrosine phosphatase, non-receptor type 11
6867	TACC1	transforming, acidic coiled-coil containing protein 1
7813	EVI5	ecotropic viral integration site 5
54888	NSUN2	NOP2/Sun domain family, member 2
10449	ACAA2	acetyl-CoA acyltransferase 2
3420	IDH3B	isocitrate dehydrogenase 3 (NAD+) beta
8402	SLC25A11	solute carrier family 25 (mitochondrial carrier; oxoglutarate carrier), member 11
27429	HTRA2	HtrA serine peptidase 2
55288	RHOT1	ras homolog gene family, member T1
10975	UQCR11	ubiquinol-cytochrome c reductase, complex III subunit XI
7388	UQCRH	ubiquinol-cytochrome c reductase hinge protein
4696	NDUFA3	NADH dehydrogenase (ubiquinone) 1 alpha subcomplex, 3, 9kDa
4717	NDUFC1	NADH dehydrogenase (ubiquinone) 1, subcomplex unknown, 1, 6kDa
4729	NDUFV2	NADH dehydrogenase (ubiquinone) flavoprotein 2, 24kDa
522	ATP5J	ATP synthase, H+ transporting, mitochondrial Fo complex, subunit F6
1347	COX7A2	cytochrome c oxidase subunit VIIa polypeptide 2 (liver)
9551	ATP5J2	ATP synthase, H+ transporting, mitochondrial Fo complex, subunit F2
65003	MRPL11	mitochondrial ribosomal protein L11
4552	MTRR	5-methyltetrahydrofolate-homocysteine methyltransferase reductase
4835	NQO2	NAD(P)H dehydrogenase, quinone 2
4976	OPA1	optic atrophy 1 (autosomal dominant)
9617	MTRF1	mitochondrial translational release factor 1
27434	POLM	polymerase (DNA directed), mu
11284	PNKP	polynucleotide kinase 3'-phosphatase
200558	APLF	aprataxin and PNKP like factor
54840	APTX	aprataxin
55738	ARFGAP1	ADP-ribosylation factor GTPase activating protein 1
10897	YIF1A	Yip1 interacting factor homolog A (S. cerevisiae)
55062	WIPI1	WD repeat domain, phosphoinositide interacting 1
2081	ERN1	endoplasmic reticulum to nucleus signaling 1
9709	HERPUD1	homocysteine-inducible, endoplasmic reticulum stress-inducible, ubiquitin-like domain member 1
1452	CSNK1A1	casein kinase 1, alpha 1
1456	CSNK1G3	casein kinase 1, gamma 3
4194	MDM4	Mdm4 p53 binding protein homolog (mouse)
1877	E4F1	E4F transcription factor 1
56950	SMYD2	SET and MYND domain containing 2
1663	DDX11	DEAD/H (Asp-Glu-Ala-Asp/His) box polypeptide 11
158	ADSL	adenylosuccinate lyase
56953	NT5M	5',3'-nucleotidase, mitochondrial
132	ADK	adenosine kinase
271	AMPD2	adenosine monophosphate deaminase 2
122622	ADSSL1	adenylosuccinate synthase like 1
1890	TYMP	thymidine phosphorylase
84033	OBSCN	obscurin, cytoskeletal calmodulin and titin-interacting RhoGEF
221472	FGD2	FYVE, RhoGEF and PH domain containing 2
2639	GCDH	glutaryl-CoA dehydrogenase
26275	HIBCH	3-hydroxyisobutyryl-CoA hydrolase
549	AUH	AU RNA binding protein/enoyl-CoA hydratase
728294	D2HGDH	D-2-hydroxyglutarate dehydrogenase
670	BPHL	biphenyl hydrolase-like (serine hydrolase)
5910	RAP1GDS1	RAP1, GTP-GDP dissociation stimulator 1
131076	CCDC58	coiled-coil domain containing 58
23173	METAP1	methionyl aminopeptidase 1
10201	NME6	non-metastatic cells 6, protein expressed in (nucleoside-diphosphate kinase)
5158	PDE6B	phosphodiesterase 6B, cGMP-specific, rod, beta
11164	NUDT5	nudix (nucleoside diphosphate linked moiety X)-type motif 5
58497	PRUNE	prune homolog (Drosophila)
6648	SOD2	superoxide dismutase 2, mitochondrial
706	TSPO	translocator protein (18kDa)
1487	CTBP1	C-terminal binding protein 1
225	ABCD2	ATP-binding cassette, sub-family D (ALD), member 2
25824	PRDX5	peroxiredoxin 5
6310	ATXN1	ataxin 1
9270	ITGB1BP1	integrin beta 1 binding protein 1
10059	DNM1L	dynamin 1-like
1676	DFFA	DNA fragmentation factor, 45kDa, alpha polypeptide
5339	PLEC	plectin
2771	GNAI2	guanine nucleotide binding protein (G protein), alpha inhibiting activity polypeptide 2
2782	GNB1	guanine nucleotide binding protein (G protein), beta polypeptide 1
8100	IFT88	intraflagellar transport 88 homolog (Chlamydomonas)
27148	STK36	serine/threonine kinase 36
11127	KIF3A	kinesin family member 3A
7029	TFDP2	transcription factor Dp-2 (E2F dimerization partner 2)
1028	CDKN1C	cyclin-dependent kinase inhibitor 1C (p57, Kip2)
3675	ITGA3	integrin, alpha 3 (antigen CD49C, alpha 3 subunit of VLA-3 receptor)
4771	NF2	neurofibromin 2 (merlin)
6237	RRAS	related RAS viral (r-ras) oncogene homolog
1500	CTNND1	catenin (cadherin-associated protein), delta 1
7059	THBS3	thrombospondin 3
64398	MPP5	membrane protein, palmitoylated 5 (MAGUK p55 subfamily member 5)
6251	RSU1	Ras suppressor protein 1
8754	ADAM9	ADAM metallopeptidase domain 9
8751	ADAM15	ADAM metallopeptidase domain 15
9019	MPZL1	myelin protein zero-like 1
5310	PKD1	polycystic kidney disease 1 (autosomal dominant)
7073	TIAL1	TIA1 cytotoxic granule-associated RNA binding protein-like 1
7106	TSPAN4	tetraspanin 4
8910	SGCE	sarcoglycan, epsilon
6888	TALDO1	transaldolase 1
5256	PHKA2	phosphorylase kinase, alpha 2 (liver)
55501	CHST12	carbohydrate (chondroitin 4) sulfotransferase 12
54480	CHPF2	chondroitin polymerizing factor 2
23443	SLC35A3	solute carrier family 35 (UDP-N-acetylglucosamine (UDP-GlcNAc) transporter), member A3
10461	MERTK	c-mer proto-oncogene tyrosine kinase
5066	PAM	peptidylglycine alpha-amidating monooxygenase
8497	PPFIA4	protein tyrosine phosphatase, receptor type, f polypeptide (PTPRF), interacting protein (liprin), alpha 4
4731	NDUFV3	NADH dehydrogenase (ubiquinone) flavoprotein 3, 10kDa
55139	ANKZF1	ankyrin repeat and zinc finger domain containing 1
10327	AKR1A1	aldo-keto reductase family 1, member A1 (aldehyde reductase)
10380	BPNT1	3'(2'), 5'-bisphosphate nucleotidase 1
64132	XYLT2	xylosyltransferase II
6676	SPAG4	sperm associated antigen 4
5261	PHKG2	phosphorylase kinase, gamma 2 (testis)
2592	GALT	galactose-1-phosphate uridylyltransferase
5106	PCK2	phosphoenolpyruvate carboxykinase 2 (mitochondrial)
5257	PHKB	phosphorylase kinase, beta
221914	GPC2	glypican 2
10919	EHMT2	euchromatic histone-lysine N-methyltransferase 2
8932	MBD2	methyl-CpG binding domain protein 2
9015	TAF1A	TATA box binding protein (TBP)-associated factor, RNA polymerase I, A, 48kDa
7019	TFAM	transcription factor A, mitochondrial
9815	GIT2	G protein-coupled receptor kinase interacting ArfGAP 2
3987	LIMS1	LIM and senescent cell antigen-like domains 1
6935	ZEB1	zinc finger E-box binding homeobox 1
63916	ELMO2	engulfment and cell motility 2
80895	ILKAP	integrin-linked kinase-associated serine/threonine phosphatase
3985	LIMK2	LIM domain kinase 2
8394	PIP5K1A	phosphatidylinositol-4-phosphate 5-kinase, type I, alpha
286205	SCAI	suppressor of cancer cell invasion
5586	PKN2	protein kinase N2
57591	MKL1	megakaryoblastic leukemia (translocation) 1
7048	TGFBR2	transforming growth factor, beta receptor II (70/80kDa)
6536	SLC6A9	solute carrier family 6 (neurotransmitter transporter, glycine), member 9
3029	HAGH	hydroxyacylglutathione hydrolase
8301	PICALM	phosphatidylinositol binding clathrin assembly protein
140609	NEK7	NIMA (never in mitosis gene a)-related kinase 7
9958	USP15	ubiquitin specific peptidase 15
5498	PPOX	protoporphyrinogen oxidase
2145	EZH1	enhancer of zeste homolog 1 (Drosophila)
51646	YPEL5	yippee-like 5 (Drosophila)
55589	BMP2K	BMP2 inducible kinase
571	BACH1	BTB and CNC homology 1, basic leucine zipper transcription factor 1
6397	SEC14L1	SEC14-like 1 (S. cerevisiae)
79269	DCAF10	DDB1 and CUL4 associated factor 10
80148	PQLC1	PQ loop repeat containing 1
6574	SLC20A1	solute carrier family 20 (phosphate transporter), member 1
2030	SLC29A1	solute carrier family 29 (nucleoside transporters), member 1
121260	SLC15A4	solute carrier family 15, member 4
6522	SLC4A2	solute carrier family 4, anion exchanger, member 2 (erythrocyte membrane protein band 3-like 1)
6575	SLC20A2	solute carrier family 20 (phosphate transporter), member 2
9497	SLC4A7	solute carrier family 4, sodium bicarbonate cotransporter, member 7
9498	SLC4A8	solute carrier family 4, sodium bicarbonate cotransporter, member 8
9990	SLC12A6	solute carrier family 12 (potassium/chloride transporters), member 6
9197	SLC33A1	solute carrier family 33 (acetyl-CoA transporter), member 1
55315	SLC29A3	solute carrier family 29 (nucleoside transporters), member 3
10559	SLC35A1	solute carrier family 35 (CMP-sialic acid transporter), member A1
29988	SLC2A8	solute carrier family 2 (facilitated glucose transporter), member 8
11182	SLC2A6	solute carrier family 2 (facilitated glucose transporter), member 6
55630	SLC39A4	solute carrier family 39 (zinc transporter), member 4
22909	FAN1	FANCD2/FANCI-associated nuclease 1
80010	RMI1	RMI1, RecQ mediated genome instability 1, homolog (S. cerevisiae)
54963	UCKL1	uridine-cytidine kinase 1-like 1
4839	NOP2	NOP2 nucleolar protein homolog (yeast)
80324	PUS1	pseudouridylate synthase 1
23223	RRP12	ribosomal RNA processing 12 homolog (S. cerevisiae)
10244	RABEPK	Rab9 effector protein with kelch motifs
54663	WDR74	WD repeat domain 74
6949	TCOF1	Treacher Collins-Franceschetti syndrome 1
81887	LAS1L	LAS1-like (S. cerevisiae)
4644	MYO5A	myosin VA (heavy chain 12, myoxin)
10643	IGF2BP3	insulin-like growth factor 2 mRNA binding protein 3
23495	TNFRSF13B	tumor necrosis factor receptor superfamily, member 13B
8573	CASK	calcium/calmodulin-dependent serine protein kinase (MAGUK family)
5562	PRKAA1	protein kinase, AMP-activated, alpha 1 catalytic subunit
10645	CAMKK2	calcium/calmodulin-dependent protein kinase kinase 2, beta
23433	RHOQ	ras homolog gene family, member Q
116985	ARAP1	ArfGAP with RhoGAP domain, ankyrin repeat and PH domain 1
116984	ARAP2	ArfGAP with RhoGAP domain, ankyrin repeat and PH domain 2
399	RHOH	ras homolog gene family, member H
2665	GDI2	GDP dissociation inhibitor 2
257106	ARHGAP30	Rho GTPase activating protein 30
51306	FAM13B	family with sequence similarity 13, member B
84986	ARHGAP19	Rho GTPase activating protein 19
93663	ARHGAP18	Rho GTPase activating protein 18
5859	QARS	glutaminyl-tRNA synthetase
10352	WARS2	tryptophanyl tRNA synthetase 2, mitochondrial
23438	HARS2	histidyl-tRNA synthetase 2, mitochondrial (putative)
57505	AARS2	alanyl-tRNA synthetase 2, mitochondrial (putative)
79587	CARS2	cysteinyl-tRNA synthetase 2, mitochondrial (putative)
80222	TARS2	threonyl-tRNA synthetase 2, mitochondrial (putative)
51520	LARS	leucyl-tRNA synthetase
8567	MADD	MAP-kinase activating death domain
952	CD38	CD38 molecule
51176	LEF1	lymphoid enhancer-binding factor 1
2944	GSTM1	glutathione S-transferase mu 1
7818	DAP3	death associated protein 3
51720	UIMC1	ubiquitin interaction motif containing 1
55247	NEIL3	nei endonuclease VIII-like 3 (E. coli)
79661	NEIL1	nei endonuclease VIII-like 1 (E. coli)
55870	ASH1L	ash1 (absent, small, or homeotic)-like (Drosophila)
8985	PLOD3	procollagen-lysine, 2-oxoglutarate 5-dioxygenase 3
132949	AASDH	aminoadipate-semialdehyde dehydrogenase
6419	SETMAR	SET domain and mariner transposase fusion gene
64324	NSD1	nuclear receptor binding SET domain protein 1
79813	EHMT1	euchromatic histone-lysine N-methyltransferase 1
83852	SETDB2	SET domain, bifurcated 2
1020	CDK5	cyclin-dependent kinase 5
961	CD47	CD47 molecule
1609	DGKQ	diacylglycerol kinase, theta 110kDa
8527	DGKD	diacylglycerol kinase, delta 130kDa
23028	KDM1A	lysine (K)-specific demethylase 1A
221955	DAGLB	diacylglycerol lipase, beta
27154	BRPF3	bromodomain and PHD finger containing, 3
7804	LRP8	low density lipoprotein receptor-related protein 8, apolipoprotein e receptor
91768	CABLES1	Cdk5 and Abl enzyme substrate 1
64147	KIF9	kinesin family member 9
23348	DOCK9	dedicator of cytokinesis 9
221037	JMJD1C	jumonji domain containing 1C
146956	EME1	essential meiotic endonuclease 1 homolog 1 (S. pombe)
7516	XRCC2	X-ray repair complementing defective repair in Chinese hamster cells 2
8940	TOP3B	topoisomerase (DNA) III beta
538	ATP7A	ATPase, Cu++ transporting, alpha polypeptide
8773	SNAP23	synaptosomal-associated protein, 23kDa
1315	COPB1	coatomer protein complex, subunit beta 1
8675	STX16	syntaxin 16
10282	BET1	blocked early in transport 1 homolog (S. cerevisiae)
23033	DOPEY1	dopey family member 1
27236	ARFIP1	ADP-ribosylation factor interacting protein 1
2803	GOLGA4	golgin A4
9586	CREB5	cAMP responsive element binding protein 5
1997	ELF1	E74-like factor 1 (ets domain transcription factor)
6598	SMARCB1	SWI/SNF related, matrix associated, actin dependent regulator of chromatin, subfamily b, member 1
117584	RFFL	ring finger and FYVE-like domain containing 1
51547	SIRT7	sirtuin 7
1107	CHD3	chromodomain helicase DNA binding protein 3
1108	CHD4	chromodomain helicase DNA binding protein 4
54815	GATAD2A	GATA zinc finger domain containing 2A
11144	DMC1	DMC1 dosage suppressor of mck1 homolog, meiosis-specific homologous recombination (yeast)
51533	PHF7	PHD finger protein 7
55342	STRBP	spermatid perinuclear RNA binding protein
55743	CHFR	checkpoint with forkhead and ring finger domains
79882	ZC3H14	zinc finger CCCH-type containing 14
219285	SAMD9L	sterile alpha motif domain containing 9-like
55072	RNF31	ring finger protein 31
64761	PARP12	poly (ADP-ribose) polymerase family, member 12
8869	ST3GAL5	ST3 beta-galactoside alpha-2,3-sialyltransferase 5
55024	BANK1	B-cell scaffold protein with ankyrin repeats 1
57674	RNF213	ring finger protein 213
55748	CNDP2	CNDP dipeptidase 2 (metallopeptidase M20 family)
125061	AFMID	arylformamidase
587	BCAT2	branched chain amino-acid transaminase 2, mitochondrial
4548	MTR	5-methyltetrahydrofolate-homocysteine methyltransferase
5831	PYCR1	pyrroline-5-carboxylate reductase 1
883	CCBL1	cysteine conjugate-beta lyase, cytoplasmic
51380	CSAD	cysteine sulfinic acid decarboxylase
435	ASL	argininosuccinate lyase
6821	SUOX	sulfite oxidase
5189	PEX1	peroxisomal biogenesis factor 1
26063	DECR2	2,4-dienoyl CoA reductase 2, peroxisomal
4358	MPV17	MpV17 mitochondrial inner membrane protein
5192	PEX10	peroxisomal biogenesis factor 10
971	CD72	CD72 molecule
5364	PLXNB1	plexin B1
10507	SEMA4D	sema domain, immunoglobulin domain (Ig), transmembrane domain (TM) and short cytoplasmic domain, (semaphorin) 4D
4637	MYL6	myosin, light chain 6, alkali, smooth muscle and non-muscle
55558	PLXNA3	plexin A3
9101	USP8	ubiquitin specific peptidase 8
7161	TP73	tumor protein p73
51512	GTSE1	G-2 and S-phase expressed 1
3708	ITPR1	inositol 1,4,5-trisphosphate receptor, type 1
3150	HMGN1	high mobility group nucleosome binding domain 1
10095	ARPC1B	actin related protein 2/3 complex, subunit 1B, 41kDa
23265	EXOC7	exocyst complex component 7
128239	IQGAP3	IQ motif containing GTPase activating protein 3
10188	TNK2	tyrosine kinase, non-receptor, 2
5217	PFN2	profilin 2
1298	COL9A2	collagen, type IX, alpha 2
6929	TCF3	transcription factor 3 (E2A immunoglobulin enhancer binding factors E12/E47)
8506	CNTNAP1	contactin associated protein 1
6938	TCF12	transcription factor 12
340061	TMEM173	transmembrane protein 173
8653	DDX3Y	DEAD (Asp-Glu-Ala-Asp) box polypeptide 3, Y-linked
2956	MSH6	mutS homolog 6 (E. coli)
22900	CARD8	caspase recruitment domain family, member 8
55914	ERBB2IP	erbb2 interacting protein
54583	EGLN1	egl nine homolog 1 (C. elegans)
197258	FUK	fucokinase
29085	PHPT1	phosphohistidine phosphatase 1
6595	SMARCA2	SWI/SNF related, matrix associated, actin dependent regulator of chromatin, subfamily a, member 2
2678	GGT1	gamma-glutamyltransferase 1
2686	GGT7	gamma-glutamyltransferase 7
114049	WBSCR22	Williams Beuren syndrome chromosome region 22
51409	HEMK1	HemK methyltransferase family member 1
51451	LCMT1	leucine carboxyl methyltransferase 1
10768	AHCYL1	adenosylhomocysteinase-like 1
51540	SCLY	selenocysteine lyase
135112	NCOA7	nuclear receptor coactivator 7
58513	EPS15L1	epidermal growth factor receptor pathway substrate 15-like 1
29979	UBQLN1	ubiquilin 1
10313	RTN3	reticulon 3
128338	DRAM2	DNA-damage regulated autophagy modulator 2
334	APLP2	amyloid beta (A4) precursor-like protein 2
84173	ELMOD3	ELMO/CED-12 domain containing 3
84895	FAM73B	family with sequence similarity 73, member B
9260	PDLIM7	PDZ and LIM domain 7 (enigma)
9144	SYNGR2	synaptogyrin 2
140465	MYL6B	myosin, light chain 6B, alkali, smooth muscle and non-muscle
165	AEBP1	AE binding protein 1
2314	FLII	flightless I homolog (Drosophila)
4608	MYBPH	myosin binding protein H
6525	SMTN	smoothelin
9172	MYOM2	myomesin (M-protein) 2, 165kDa
55577	NAGK	N-acetylglucosamine kinase
132789	GNPDA2	glucosamine-6-phosphate deaminase 2
51005	AMDHD2	amidohydrolase domain containing 2
5973	RENBP	renin binding protein
80896	NPL	N-acetylneuraminate pyruvate lyase (dihydrodipicolinate synthase)
1796	DOK1	docking protein 1, 62kDa (downstream of tyrosine kinase 1)
4776	NFATC4	nuclear factor of activated T-cells, cytoplasmic, calcineurin-dependent 4
10725	NFAT5	nuclear factor of activated T-cells 5, tonicity-responsive
3064	HTT	huntingtin
133746	JMY	junction mediating and regulatory protein, p53 cofactor
1820	ARID3A	AT rich interactive domain 3A (BRIGHT-like)
10347	ABCA7	ATP-binding cassette, sub-family A (ABC1), member 7
286410	ATP11C	ATPase, class VI, type 11C

374868	ATP9B	ATPase, class II, type 9B
28982	FLVCR1	feline leukemia virus subgroup C cellular receptor 1
9963	SLC23A1	solute carrier family 23 (nucleobase transporters), member 1
79621	RNASEH2B	ribonuclease H2, subunit B
9191	DEDD	death effector domain containing
27131	SNX5	sorting nexin 5
201163	FLCN	folliculin
57107	PDSS2	prenyl (decaprenyl) diphosphate synthase, subunit 2
6778	STAT6	signal transducer and activator of transcription 6, interleukin-4 induced
2241	FER	fer (fps/fes related) tyrosine kinase
5800	PTPRO	protein tyrosine phosphatase, receptor type, O
51701	NLK	nemo-like kinase
83637	ZMIZ2	zinc finger, MIZ-type containing 2
9744	ACAP1	ArfGAP with coiled-coil, ankyrin repeat and PH domains 1
10617	STAMBP	STAM binding protein
116986	AGAP2	ArfGAP with GTPase domain, ankyrin repeat and PH domain 2
5869	RAB5B	RAB5B, member RAS oncogene family
56904	SH3GLB2	SH3-domain GRB2-like endophilin B2
23013	SPEN	spen homolog, transcriptional regulator (Drosophila)
5993	RFX5	regulatory factor X, 5 (influences HLA class II expression)
4122	MAN2A2	mannosidase, alpha, class 2A, member 2
79412	KREMEN2	kringle containing transmembrane protein 2
1605	DAG1	dystroglycan 1 (dystrophin-associated glycoprotein 1)
7402	UTRN	utrophin
84271	POLDIP3	polymerase (DNA-directed), delta interacting protein 3
2011	MARK2	MAP/microtubule affinity-regulating kinase 2
984	CDK11B	cyclin-dependent kinase 11B
23081	KDM4C	lysine (K)-specific demethylase 4C
1998	ELF2	E74-like factor 2 (ets domain transcription factor)
7456	WIPF1	WAS/WASL interacting protein family, member 1
1523	CUX1	cut-like homeobox 1
4240	MFGE8	milk fat globule-EGF factor 8 protein
9910	RABGAP1L	RAB GTPase activating protein 1-like
8784	TNFRSF18	tumor necrosis factor receptor superfamily, member 18
5025	P2RX4	purinergic receptor P2X, ligand-gated ion channel, 4
6484	ST3GAL4	ST3 beta-galactoside alpha-2,3-sialyltransferase 4
22807	IKZF2	IKAROS family zinc finger 2 (Helios)
5325	PLAGL1	pleiomorphic adenoma gene-like 1
57231	SNX14	sorting nexin 14
25989	ULK3	unc-51-like kinase 3 (C. elegans)
51477	ISYNA1	inositol-3-phosphate synthase 1
10152	ABI2	abl-interactor 2
133	ADM	adrenomedullin
79017	GGCT	gamma-glutamylcyclotransferase
6601	SMARCC2	SWI/SNF related, matrix associated, actin dependent regulator of chromatin, subfamily c, member 2
11146	GLMN	glomulin, FKBP associated protein
4528	MTIF2	mitochondrial translational initiation factor 2
8444	DYRK3	dual-specificity tyrosine-(Y)-phosphorylation regulated kinase 3
10994	ILVBL	ilvB (bacterial acetolactate synthase)-like
10043	TOM1	target of myb1 (chicken)
22797	TFEC	transcription factor EC
27044	SND1	staphylococcal nuclease and tudor domain containing 1
25981	DNAH1	dynein, axonemal, heavy chain 1
132158	GLYCTK	glycerate kinase
23324	MAN2B2	mannosidase, alpha, class 2B, member 2
4123	MAN2C1	mannosidase, alpha, class 2C, member 1
79717	PPCS	phosphopantothenoylcysteine synthetase
80025	PANK2	pantothenate kinase 2
8875	VNN2	vanin 2
196403	DTX3	deltex homolog 3 (Drosophila)
22884	WDR37	WD repeat domain 37
23469	PHF3	PHD finger protein 3
27109	ATP5S	ATP synthase, H+ transporting, mitochondrial Fo complex, subunit s (factor B)
51460	SFMBT1	Scm-like with four mbt domains 1
7799	PRDM2	PR domain containing 2, with ZNF domain
8455	ATRN	attractin
275	AMT	aminomethyltransferase
55902	ACSS2	acyl-CoA synthetase short-chain family member 2
6311	ATXN2	ataxin 2
4008	LMO7	LIM domain 7
7391	USF1	upstream transcription factor 1
65220	NADK	NAD kinase
80308	FLAD1	FAD1 flavin adenine dinucleotide synthetase homolog (S. cerevisiae)
2356	FPGS	folylpolyglutamate synthase
79178	THTPA	thiamine triphosphatase
55844	PPP2R2D	protein phosphatase 2, regulatory subunit B, delta
9223	MAGI1	membrane associated guanylate kinase, WW and PDZ domain containing 1
2305	FOXM1	forkhead box M1
56938	ARNTL2	aryl hydrocarbon receptor nuclear translocator-like 2
26155	NOC2L	nucleolar complex associated 2 homolog (S. cerevisiae)
23464	GCAT	glycine C-acetyltransferase
55768	NGLY1	N-glycanase 1
64333	ARHGAP9	Rho GTPase activating protein 9
9941	EXOG	endo/exonuclease (5'-3'), endonuclease G-like
57680	CHD8	chromodomain helicase DNA binding protein 8
6801	STRN	striatin, calmodulin binding protein
10320	IKZF1	IKAROS family zinc finger 1 (Ikaros)
3783	KCNN4	potassium intermediate/small conductance calcium-activated channel, subfamily N, member 4
23268	DNMBP	dynamin binding protein
163590	TOR1AIP2	torsin A interacting protein 2
58155	PTBP2	polypyrimidine tract binding protein 2
83734	ATG10	ATG10 autophagy related 10 homolog (S. cerevisiae)
84869	CBR4	carbonyl reductase 4
59339	PLEKHA2	pleckstrin homology domain containing, family A (phosphoinositide binding specific) member 2
2054	STX2	syntaxin 2
203062	TSNARE1	t-SNARE domain containing 1
2595	GANC	glucosidase, alpha; neutral C
2907	GRINA	glutamate receptor, ionotropic, N-methyl D-aspartate-associated protein 1 (glutamate binding)
55937	APOM	apolipoprotein M
6415	SEPW1	selenoprotein W, 1
4841	NONO	non-POU domain containing, octamer-binding
84057	MND1	meiotic nuclear divisions 1 homolog (S. cerevisiae)
6284	S100A13	S100 calcium binding protein A13
10600	USP16	ubiquitin specific peptidase 16
120892	LRRK2	leucine-rich repeat kinase 2
8437	RASAL1	RAS protein activator like 1 (GAP1 like)
27342	RABGEF1	RAB guanine nucleotide exchange factor (GEF) 1
9555	H2AFY	H2A histone family, member Y
59349	KLHL12	kelch-like 12 (Drosophila)
8498	RANBP3	RAN binding protein 3
340481	ZDHHC21	zinc finger, DHHC-type containing 21
23387	SIK3	SIK family kinase 3
6642	SNX1	sorting nexin 1
4299	AFF1	AF4/FMR2 family, member 1
29887	SNX10	sorting nexin 10
4665	NAB2	NGFI-A binding protein 2 (EGR1 binding protein 2)
10237	SLC35B1	solute carrier family 35, member B1
7273	TTN	titin
196294	IMMP1L	IMP1 inner mitochondrial membrane peptidase-like (S. cerevisiae)
112483	SAT2	spermidine/spermine N1-acetyltransferase family member 2
98	ACYP2	acylphosphatase 2, muscle type
22876	INPP5F	inositol polyphosphate-5-phosphatase F
9094	UNC119	unc-119 homolog (C. elegans)
10863	ADAM28	ADAM metallopeptidase domain 28
7434	VIPR2	vasoactive intestinal peptide receptor 2
2696	GIPR	gastric inhibitory polypeptide receptor
1787	TRDMT1	tRNA aspartic acid methyltransferase 1
84938	ATG4C	ATG4 autophagy related 4 homolog C (S. cerevisiae)
9988	DMTF1	cyclin D binding myb-like transcription factor 1
50615	IL21R	interleukin 21 receptor
80331	DNAJC5	DnaJ (Hsp40) homolog, subfamily C, member 5
23415	KCNH4	potassium voltage-gated channel, subfamily H (eag-related), member 4
9196	KCNAB3	potassium voltage-gated channel, shaker-related subfamily, beta member 3
4026	LPP	LIM domain containing preferred translocation partner in lipoma
970	CD70	CD70 molecule
10630	PDPN	podoplanin
4481	MSR1	macrophage scavenger receptor 1
9465	AKAP7	A kinase (PRKA) anchor protein 7
27334	P2RY10	purinergic receptor P2Y, G-protein coupled, 10
2971	GTF3A	general transcription factor IIIA
7392	USF2	upstream transcription factor 2, c-fos interacting
79676	OGFOD2	2-oxoglutarate and iron-dependent oxygenase domain containing 2
9719	ADAMTSL2	ADAMTS-like 2
4780	NFE2L2	nuclear factor (erythroid-derived 2)-like 2
9120	SLC16A6	solute carrier family 16, member 6 (monocarboxylic acid transporter 7)
317749	DHRS4L2	dehydrogenase/reductase (SDR family) member 4 like 2
931	MS4A1	membrane-spanning 4-domains, subfamily A, member 1
6738	TROVE2	TROVE domain family, member 2
60401	EDA2R	ectodysplasin A2 receptor
203522	DDX26B	DEAD/H (Asp-Glu-Ala-Asp/His) box polypeptide 26B
22932	POMZP3	POM121 and ZP3 fusion
25992	SNED1	sushi, nidogen and EGF-like domains 1
78987	CRELD1	cysteine-rich with EGF-like domains 1
29121	CLEC2D	C-type lectin domain family 2, member D
5324	PLAG1	pleiomorphic adenoma gene 1
54901	CDKAL1	CDK5 regulatory subunit associated protein 1-like 1
9746	CLSTN3	calsyntenin 3
1954	MEGF8	multiple EGF-like-domains 8
440275	EIF2AK4	eukaryotic translation initiation factor 2 alpha kinase 4
4750	NEK1	NIMA (never in mitosis gene a)-related kinase 1
100048912	CDKN2B-AS	CDKN2B antisense RNA (non-protein coding)
100131187	TSTD1	thiosulfate sulfurtransferase (rhodanese)-like domain containing 1
100302692	FTX	FTX transcript, XIST regulator (non-protein coding)
10042	HMGXB4	HMG box domain containing 4
10045	SH2D3A	SH2 domain containing 3A
10069	RWDD2B	RWD domain containing 2B
10114	HIPK3	homeodomain interacting protein kinase 3
10138	YAF2	YY1 associated factor 2
10147	SUGP2	SURP and G patch domain containing 2
10150	MBNL2	muscleblind-like 2 (Drosophila)
10180	RBM6	RNA binding motif protein 6
10198	MPHOSPH9	M-phase phosphoprotein 9
10208	USPL1	ubiquitin specific peptidase like 1
10227	MFSD10	major facilitator superfamily domain containing 10
10260	DENND4A	DENN/MADD domain containing 4A
10269	ZMPSTE24	zinc metallopeptidase (STE24 homolog, S. cerevisiae)
10301	DLEU1	deleted in lymphocytic leukemia 1 (non-protein coding)
10307	APBB3	amyloid beta (A4) precursor protein-binding, family B, member 3
10322	SMYD5	SMYD family member 5
10329	TMEM5	transmembrane protein 5
10336	PCGF3	polycomb group ring finger 3
10443	N4BP2L2	NEDD4 binding protein 2-like 2
10447	FAM3C	family with sequence similarity 3, member C
10464	PIBF1	progesterone immunomodulatory binding factor 1
10467	ZNHIT1	zinc finger, HIT-type containing 1
10480	EIF3M	eukaryotic translation initiation factor 3, subunit M
10483	SEC23B	Sec23 homolog B (S. cerevisiae)
10531	PITRM1	pitrilysin metallopeptidase 1
10585	POMT1	protein-O-mannosyltransferase 1
10613	ERLIN1	ER lipid raft associated 1
10625	IVNS1ABP	influenza virus NS1A binding protein
10641	NPRL2	nitrogen permease regulator-like 2 (S. cerevisiae)
10658	CELF1	CUGBP, Elav-like family member 1
10691	GMEB1	glucocorticoid modulatory element binding protein 1
10741	RBBP9	retinoblastoma binding protein 9
10827	FAM114A2	family with sequence similarity 114, member A2
10865	ARID5A	AT rich interactive domain 5A (MRF1-like)
10922	FASTK	Fas-activated serine/threonine kinase
10923	SUB1	SUB1 homolog (S. cerevisiae)
10927	SPIN1	spindlin 1
10980	COPS6	COP9 constitutive photomorphogenic homolog subunit 6 (Arabidopsis)
11011	TLK2	tousled-like kinase 2
1102	RCBTB2	regulator of chromosome condensation (RCC1) and BTB (POZ) domain containing protein 2
11020	IFT27	intraflagellar transport 27 homolog (Chlamydomonas)
11101	ATE1	arginyltransferase 1
11118	BTN3A2	butyrophilin, subfamily 3, member A2
11129	CLASRP	CLK4-associating serine/arginine rich protein
11165	NUDT3	nudix (nucleoside diphosphate linked moiety X)-type motif 3
11169	WDHD1	WD repeat and HMG-box DNA binding protein 1
11177	BAZ1A	bromodomain adjacent to zinc finger domain, 1A
11188	NISCH	nischarin
11201	POLI	polymerase (DNA directed) iota
11212	PROSC	proline synthetase co-transcribed homolog (bacterial)
11234	HPS5	Hermansky-Pudlak syndrome 5
11257	TP53TG1	TP53 target 1 (non-protein coding)
11272	PRR4	proline rich 4 (lacrimal)
11276	SYNRG	synergin, gamma
112936	VPS26B	vacuolar protein sorting 26 homolog B (S. pombe)
1130	LYST	lysosomal trafficking regulator
113000	RPUSD1	RNA pseudouridylate synthase domain containing 1
113201	CASC4	cancer susceptibility candidate 4
113246	C12orf57	chromosome 12 open reading frame 57
113251	LARP4	La ribonucleoprotein domain family, member 4
113655	MFSD3	major facilitator superfamily domain containing 3
114785	MBD6	methyl-CpG binding domain protein 6
114793	FMNL2	formin-like 2
114803	MYSM1	Myb-like, SWIRM and MPN domains 1
114814	GNRHR2	gonadotropin-releasing hormone (type 2) receptor 2
114882	OSBPL8	oxysterol binding protein-like 8
114883	OSBPL9	oxysterol binding protein-like 9
114971	PTPMT1	protein tyrosine phosphatase, mitochondrial 1
115209	OMA1	OMA1 homolog, zinc metallopeptidase (S. cerevisiae)
1153	CIRBP	cold inducible RNA binding protein
115353	LRRC42	leucine rich repeat containing 42
115426	UHRF2	ubiquitin-like with PHD and ring finger domains 2
115950	ZNF653	zinc finger protein 653
116447	TOP1MT	topoisomerase (DNA) I, mitochondrial
118472	ZNF511	zinc finger protein 511
1185	CLCN6	chloride channel 6
118813	ZFYVE27	zinc finger, FYVE domain containing 27
1195	CLK1	CDC-like kinase 1
119504	ANAPC16	anaphase promoting complex subunit 16
1196	CLK2	CDC-like kinase 2
1198	CLK3	CDC-like kinase 3
120103	SLC36A4	solute carrier family 36 (proton/amino acid symporter), member 4
122509	IFI27L1	interferon, alpha-inducible protein 27-like 1
122553	TRAPPC6B	trafficking protein particle complex 6B
122704	MRPL52	mitochondrial ribosomal protein L52
123016	TTC8	tetratricopeptide repeat domain 8
123096	SLC25A29	solute carrier family 25, member 29
123606	NIPA1	non imprinted in Prader-Willi/Angelman syndrome 1
123811	FOPNL	FGFR1OP N-terminal like
124093	CCDC78	coiled-coil domain containing 78
124401	ANKS3	ankyrin repeat and sterile alpha motif domain containing 3
124411	ZNF720	zinc finger protein 720
124923	SGK494	uncharacterized serine/threonine-protein kinase SgK494
124944	C17orf49	chromosome 17 open reading frame 49
125058	TBC1D16	TBC1 domain family, member 16
126075	CCDC159	coiled-coil domain containing 159
126231	ZNF573	zinc finger protein 573
126526	C19orf47	chromosome 19 open reading frame 47
126626	GABPB2	GA binding protein transcription factor, beta subunit 2
127002	ATXN7L2	ataxin 7-like 2
127003	C1orf194	chromosome 1 open reading frame 194
127700	OSCP1	organic solute carrier partner 1
128240	APOA1BP	apolipoprotein A-I binding protein
128308	MRPL55	mitochondrial ribosomal protein L55
128387	TATDN3	TatD DNase domain containing 3
129531	MITD1	MIT, microtubule interacting and transport, domain containing 1
129563	DIS3L2	DIS3 mitotic control homolog (S. cerevisiae)-like 2
130507	UBR3	ubiquitin protein ligase E3 component n-recognin 3 (putative)
130872	AHSA2	AHA1, activator of heat shock 90kDa protein ATPase homolog 2 (yeast)
131601	TPRA1	transmembrane protein, adipocyte asscociated 1
132160	PPM1M	protein phosphatase, Mg2+/Mn2+ dependent, 1M
132321	C4orf33	chromosome 4 open reading frame 33
132989	C4orf36	chromosome 4 open reading frame 36
133015	PACRGL	PARK2 co-regulated-like
134145	FAM173B	family with sequence similarity 173, member B
134359	POC5	POC5 centriolar protein homolog (Chlamydomonas)
134637	ADAT2	adenosine deaminase, tRNA-specific 2
134957	STXBP5	syntaxin binding protein 5 (tomosyn)
136051	ZNF786	zinc finger protein 786
137695	TMEM68	transmembrane protein 68
137886	UBXN2B	UBX domain protein 2B
140564	APOBEC3D	apolipoprotein B mRNA editing enzyme, catalytic polypeptide-like 3D
140890	SREK1	splicing regulatory glutamine/lysine-rich protein 1
143570	XRRA1	X-ray radiation resistance associated 1
143684	FAM76B	family with sequence similarity 76, member B
144132	DNHD1	dynein heavy chain domain 1
144348	ZNF664	zinc finger protein 664
144453	BEST3	bestrophin 3
145389	SLC38A6	solute carrier family 38, member 6
145508	CEP128	centrosomal protein 128kDa
146057	TTBK2	tau tubulin kinase 2
146206	RLTPR	RGD motif, leucine rich repeats, tropomodulin domain and proline-rich containing
146542	ZNF688	zinc finger protein 688
147172	LRRC37BP1	leucine rich repeat containing 37B pseudogene 1
147991	DPY19L3	dpy-19-like 3 (C. elegans)
148203	ZNF738	zinc finger protein 738
148206	ZNF714	zinc finger protein 714
148223	C19orf25	chromosome 19 open reading frame 25
148423	C1orf52	chromosome 1 open reading frame 52
1486	CTBS	chitobiase, di-N-acetyl-
148789	B3GALNT2	beta-1,3-N-acetylgalactosaminyltransferase 2
149175	MANEAL	mannosidase, endo-alpha-like
149986	LSM14B	LSM14B, SCD6 homolog B (S. cerevisiae)
150223	YDJC	YdjC homolog (bacterial)
150465	TTL	tubulin tyrosine ligase
150472	CBWD2	COBW domain containing 2
150590	C2orf15	chromosome 2 open reading frame 15
151194	METTL21A	methyltransferase like 21A
151613	TTC14	tetratricopeptide repeat domain 14
152006	RNF38	ring finger protein 38
153129	SLC38A9	solute carrier family 38, member 9
155400	NSUN5P1	NOP2/Sun domain family, member 5 pseudogene 1
155435	RBM33	RNA binding motif protein 33

157574	FBXO16	F-box protein 16
157680	VPS13B	vacuolar protein sorting 13 homolog B (yeast)
158427	TSTD2	thiosulfate sulfurtransferase (rhodanese)-like domain containing 2
158431	ZNF782	zinc finger protein 782
158584	FAAH2	fatty acid amide hydrolase 2
160365	CLECL1	C-type lectin-like 1
160897	GPR180	G protein-coupled receptor 180
162681	C18orf54	chromosome 18 open reading frame 54
163486	DENND1B	DENN/MADD domain containing 1B
165055	CCDC138	coiled-coil domain containing 138
165631	PARP15	poly (ADP-ribose) polymerase family, member 15
167153	PAPD4	PAP associated domain containing 4
169200	TMEM64	transmembrane protein 64
169714	QSOX2	quiescin Q6 sulfhydryl oxidase 2
169981	SPIN3	spindlin family, member 3
170622	COMMD6	COMM domain containing 6
170960	ZNF721	zinc finger protein 721
171017	ZNF384	zinc finger protein 384
171023	ASXL1	additional sex combs like 1 (Drosophila)
171586	ABHD3	abhydrolase domain containing 3
172	AFG3L1P	AFG3 ATPase family gene 3-like 1 (S. cerevisiae), pseudogene
1774	DNASE1L1	deoxyribonuclease I-like 1
1801	DPH1	DPH1 homolog (S. cerevisiae)
1911	PHC1	polyhomeotic homolog 1 (Drosophila)
1939	EIF2D	eukaryotic translation initiation factor 2D
196264	MPZL3	myelin protein zero-like 3
196394	AMN1	antagonist of mitotic exit network 1 homolog (S. cerevisiae)
199746	U2AF1L4	U2 small nuclear RNA auxiliary factor 1-like 4
200014	CC2D1B	coiled-coil and C2 domain containing 1B
200030	NBPF11	neuroblastoma breakpoint family, member 11
200185	KRTCAP2	keratinocyte associated protein 2
201254	STRA13	stimulated by retinoic acid 13 homolog (mouse)
201266	SLC39A11	solute carrier family 39 (metal ion transporter), member 11
201283	AMZ2P1	archaelysin family metallopeptidase 2 pseudogene 1
201292	TRIM65	tripartite motif containing 65
201931	TMEM192	transmembrane protein 192
202018	TAPT1	transmembrane anterior posterior transformation 1
202243	CCDC125	coiled-coil domain containing 125
2140	EYA3	eyes absent homolog 3 (Drosophila)
2186	BPTF	bromodomain PHD finger transcription factor
219899	TBCEL	tubulin folding cofactor E-like
219931	TPCN2	two pore segment channel 2
220074	LRTOMT	leucine rich transmembrane and 0-methyltransferase domain containing
221016	CCDC7	coiled-coil domain containing 7
221711	SYCP2L	synaptonemal complex protein 2-like
2218	FKTN	fukutin
221927	BRAT1	BRCA1-associated ATM activator 1
222255	ATXN7L1	ataxin 7-like 1
222484	LNX2	ligand of numb-protein X 2
22834	ZNF652	zinc finger protein 652
22838	RNF44	ring finger protein 44
22839	DLGAP4	discs, large (Drosophila) homolog-associated protein 4
22858	ICK	intestinal cell (MAK-like) kinase
22878	TRAPPC8	trafficking protein particle complex 8
22890	ZBTB1	zinc finger and BTB domain containing 1
22902	RUFY3	RUN and FYVE domain containing 3
22907	DHX30	DEAH (Asp-Glu-Ala-His) box polypeptide 30
22913	RALY	RNA binding protein, autoantigenic (hnRNP-associated with lethal yellow homolog (mouse))
22955	SCMH1	sex comb on midleg homolog 1 (Drosophila)
22980	TCF25	transcription factor 25 (basic helix-loop-helix)
22982	DIP2C	DIP2 disco-interacting protein 2 homolog C (Drosophila)
22984	PDCD11	programmed cell death 11
22995	CEP152	centrosomal protein 152kDa
23023	TMCC1	transmembrane and coiled-coil domain family 1
23042	PDXDC1	pyridoxal-dependent decarboxylase domain containing 1
23064	SETX	senataxin
23074	UHRF1BP1L	UHRF1 binding protein 1-like
23077	MYCBP2	MYC binding protein 2
23091	ZC3H13	zinc finger CCCH-type containing 13
23093	TTLL5	tubulin tyrosine ligase-like family, member 5
23113	CUL9	cullin 9
23125	CAMTA2	calmodulin binding transcription activator 2
23126	POGZ	pogo transposable element with ZNF domain
23130	ATG2A	ATG2 autophagy related 2 homolog A (S. cerevisiae)
23131	GPATCH8	G patch domain containing 8
23132	RAD54L2	RAD54-like 2 (S. cerevisiae)
23137	SMC5	structural maintenance of chromosomes 5
23140	ZZEF1	zinc finger, ZZ-type with EF-hand domain 1
23142	DCUN1D4	DCN1, defective in cullin neddylation 1, domain containing 4 (S. cerevisiae)
23155	CLCC1	chloride channel CLIC-like 1
23190	UBXN4	UBX domain protein 4
23214	XPO6	exportin 6
23215	PRRC2C	proline-rich coiled-coil 2C
23216	TBC1D1	TBC1 (tre-2/USP6, BUB2, cdc16) domain family, member 1
23231	SEL1L3	sel-1 suppressor of lin-12-like 3 (C. elegans)
23241	PACS2	phosphofurin acidic cluster sorting protein 2
23253	ANKRD12	ankyrin repeat domain 12
23261	CAMTA1	calmodulin binding transcription activator 1
23262	PPIP5K2	diphosphoinositol pentakisphosphate kinase 2
23269	MGA	MAX gene associated
23274	CLEC16A	C-type lectin domain family 16, member A
23279	NUP160	nucleoporin 160kDa
23301	EHBP1	EH domain binding protein 1
23312	DMXL2	Dmx-like 2
23316	CUX2	cut-like homeobox 2
23318	ZCCHC11	zinc finger, CCHC domain containing 11
23334	SZT2	seizure threshold 2 homolog (mouse)
23347	SMCHD1	structural maintenance of chromosomes flexible hinge domain containing 1
23353	SUN1	Sad1 and UNC84 domain containing 1
23354	HAUS5	HAUS augmin-like complex, subunit 5
23355	VPS8	vacuolar protein sorting 8 homolog (S. cerevisiae)
23360	FNBP4	formin binding protein 4
23366	KIAA0895	KIAA0895
23369	PUM2	pumilio homolog 2 (Drosophila)
23384	SPECC1L	sperm antigen with calponin homology and coiled-coil domains 1-like
23386	NUDCD3	NudC domain containing 3
23473	CAPN7	calpain 7
23483	TGDS	TDP-glucose 4,6-dehydratase
23499	MACF1	microtubule-actin crosslinking factor 1
23513	SCRIB	scribbled homolog (Drosophila)
23534	TNPO3	transportin 3
23559	WBP1	WW domain binding protein 1
23613	ZMYND8	zinc finger, MYND-type containing 8
23642	SNHG1	small nucleolar RNA host gene 1 (non-protein coding)
23787	MTCH1	mitochondrial carrier 1
24139	EML2	echinoderm microtubule associated protein like 2
253725	FAM21C	family with sequence similarity 21, member C
253769	WDR27	WD repeat domain 27
253832	ZDHHC20	zinc finger, DHHC-type containing 20
254048	UBN2	ubinuclein 2
254102	EHBP1L1	EH domain binding protein 1-like 1
254122	SNX32	sorting nexin 32
254295	PHYHD1	phytanoyl-CoA dioxygenase domain containing 1
2551	GABPA	GA binding protein transcription factor, alpha subunit 60kDa
255231	MCOLN2	mucolipin 2
2553	GABPB1	GA binding protein transcription factor, beta subunit 1
255967	PAN3	PAN3 poly(A) specific ribonuclease subunit homolog (S. cerevisiae)
256051	ZNF549	zinc finger protein 549
256356	GK5	glycerol kinase 5 (putative)
256364	EML3	echinoderm microtubule associated protein like 3
256987	SERINC5	serine incorporator 5
257218	SHPRH	SNF2 histone linker PHD RING helicase
25771	TBC1D22A	TBC1 domain family, member 22A
25831	HECTD1	HECT domain containing 1
25852	ARMC8	armadillo repeat containing 8
25864	ABHD14A	abhydrolase domain containing 14A
25886	POC1A	POC1 centriolar protein homolog A (Chlamydomonas)
25914	RTTN	rotatin
25917	THUMPD3	THUMP domain containing 3
25930	PTPN23	protein tyrosine phosphatase, non-receptor type 23
25950	RWDD3	RWD domain containing 3
25956	SEC31B	SEC31 homolog B (S. cerevisiae)
25957	PNISR	PNN-interacting serine/arginine-rich protein
25963	TMEM87A	transmembrane protein 87A
25988	HINFP	histone H4 transcription factor
26001	RNF167	ring finger protein 167
26009	ZZZ3	zinc finger, ZZ-type containing 3
26013	L3MBTL1	l(3)mbt-like 1 (Drosophila)
26031	OSBPL3	oxysterol binding protein-like 3
26036	ZNF451	zinc finger protein 451
26054	SENP6	SUMO1/sentrin specific peptidase 6
26057	ANKRD17	ankyrin repeat domain 17
26058	GIGYF2	GRB10 interacting GYF protein 2
26065	LSM14A	LSM14A, SCD6 homolog A (S. cerevisiae)
26100	WIPI2	WD repeat domain, phosphoinositide interacting 2
26123	TCTN3	tectonic family member 3
26140	TTLL3	tubulin tyrosine ligase-like family, member 3
26151	NAT9	N-acetyltransferase 9 (GCN5-related, putative)
26205	GMEB2	glucocorticoid modulatory element binding protein 2
26233	FBXL6	F-box and leucine-rich repeat protein 6
26235	FBXL4	F-box and leucine-rich repeat protein 4
26260	FBXO25	F-box protein 25
26262	TSPAN17	tetraspanin 17
26276	VPS33B	vacuolar protein sorting 33 homolog B (yeast)
2630	GBAP1	glucosidase, beta, acid pseudogene 1
26750	RPS6KC1	ribosomal protein S6 kinase, 52kDa, polypeptide 1
26960	NBEA	neurobeachin
26996	GPR160	G protein-coupled receptor 160
27004	TCL6	T-cell leukemia/lymphoma 6 (non-protein coding)
27037	TRMT2A	TRM2 tRNA methyltransferase 2 homolog A (S. cerevisiae)
27067	STAU2	staufen, RNA binding protein, homolog 2 (Drosophila)
27072	VPS41	vacuolar protein sorting 41 homolog (S. cerevisiae)
27077	B9D1	B9 protein domain 1
27086	FOXP1	forkhead box P1
27141	CIDEB	cell death-inducing DFFA-like effector b
27163	NAAA	N-acylethanolamine acid amidase
27229	TUBGCP4	tubulin, gamma complex associated protein 4
27315	PGAP2	post-GPI attachment to proteins 2
2733	GLE1	GLE1 RNA export mediator homolog (yeast)
27333	GOLIM4	golgi integral membrane protein 4
27352	SGSM3	small G protein signaling modulator 3
283248	RCOR2	REST corepressor 2
283643	C14orf80	chromosome 14 open reading frame 80
283755	HERC2P3	hect domain and RLD 2 pseudogene 3
284001	CCDC57	coiled-coil domain containing 57
284004	HEXDC	hexosaminidase (glycosyl hydrolase family 20, catalytic domain) containing
284047	CCDC144B	coiled-coil domain containing 144B (pseudogene)
284129	SLC26A11	solute carrier family 26, member 11
284323	ZNF780A	zinc finger protein 780A
284459	HKR1	HKR1, GLI-Kruppel zinc finger family member
284565	NBPF15	neuroblastoma breakpoint family, member 15
284613	CYB561D1	cytochrome b-561 domain containing 1
284716	RIMKLA	ribosomal modification protein rimK-like family member A
284900	TTC28-AS1	TTC28 antisense RNA 1 (non-protein coding)
285025	CCDC141	coiled-coil domain containing 141
285193	DUSP28	dual specificity phosphatase 28
285331	CCDC66	coiled-coil domain containing 66
285346	ZNF852	zinc finger protein 852
285367	RPUSD3	RNA pseudouridylate synthase domain containing 3
285527	FRYL	FRY-like
285550	FAM200B	family with sequence similarity 200, member B
285613	RELL2	RELT-like 2
285753	CEP57L1	centrosomal protein 57kDa-like 1
285971	ZNF775	zinc finger protein 775
285989	ZNF789	zinc finger protein 789
28942	IGKV1-8	immunoglobulin kappa variable 1-8
28957	MRPS28	mitochondrial ribosomal protein S28
28969	BZW2	basic leucine zipper and W2 domains 2
28990	ASTE1	asteroid homolog 1 (Drosophila)
29066	ZC3H7A	zinc finger CCCH-type containing 7A
29087	THYN1	thymocyte nuclear protein 1
29093	MRPL22	mitochondrial ribosomal protein L22
29097	CNIH4	cornichon homolog 4 (Drosophila)
29098	RANGRF	RAN guanine nucleotide release factor
29115	SAP30BP	SAP30 binding protein
29123	ANKRD11	ankyrin repeat domain 11
2975	GTF3C1	general transcription factor IIIC, polypeptide 1, alpha 220kDa
29761	USP25	ubiquitin specific peptidase 25
29789	OLA1	Obg-like ATPase 1
29803	REPIN1	replication initiator 1
29916	SNX11	sorting nexin 11
29952	DPP7	dipeptidyl-peptidase 7
29966	STRN3	striatin, calmodulin binding protein 3
29994	BAZ2B	bromodomain adjacent to zinc finger domain, 2B
3136	HLA-H	major histocompatibility complex, class I, H (pseudogene)
3141	HLCS	holocarboxylase synthetase (biotin-(proprionyl-CoA-carboxylase (ATP-hydrolysing)) ligase)
317662	FAM149B1	family with sequence similarity 149, member B1
321	APBA2	amyloid beta (A4) precursor protein-binding, family A, member 2
3257	HPS1	Hermansky-Pudlak syndrome 1
3299	HSF4	heat shock transcription factor 4
338440	ANO9	anoctamin 9
338692	ANKRD13D	ankyrin repeat domain 13 family, member D
339123	JMJD8	jumonji domain containing 8
339487	ZBTB8OS	zinc finger and BTB domain containing 8 opposite strand
339804	C2orf74	chromosome 2 open reading frame 74
340252	ZNF680	zinc finger protein 680
340371	NRBP2	nuclear receptor binding protein 2
343099	CCDC18	coiled-coil domain containing 18
346389	MACC1	metastasis associated in colon cancer 1
347240	KIF24	kinesin family member 24
347344	ZNF81	zinc finger protein 81
347735	SERINC2	serine incorporator 2
348180	CTU2	cytosolic thiouridylase subunit 2 homolog (S. pombe)
349136	WDR86	WD repeat domain 86
349152	DPY19L2P2	dpy-19-like 2 pseudogene 2 (C. elegans)
353088	ZNF429	zinc finger protein 429
353322	ANKRD37	ankyrin repeat domain 37
353511	PKD1P6	polycystic kidney disease 1 (autosomal dominant) pseudogene 6
359948	IRF2BP2	interferon regulatory factor 2 binding protein 2
374393	FAM111B	family with sequence similarity 111, member B
374403	TBC1D10C	TBC1 domain family, member 10C
374618	TEX9	testis expressed 9
375248	ANKRD36	ankyrin repeat domain 36
377007	KLHL30	kelch-like 30 (Drosophila)
386593	CHKB-CPT1B	CHKB-CPT1B readthrough (non-protein coding)
387036	GUSBP2	glucuronidase, beta pseudogene 2
387066	SNHG5	small nucleolar RNA host gene 5 (non-protein coding)
387338	NSUN4	NOP2/Sun domain family, member 4
387921	NHLRC3	NHL repeat containing 3
388558	ZNF808	zinc finger protein 808
391059	FRRS1	ferric-chelate reductase 1
399818	METTL10	methyltransferase like 10
399909	PCNXL3	pecanex-like 3 (Drosophila)
399979	SNX19	sorting nexin 19
400073	C12orf76	chromosome 12 open reading frame 76
400954	EML6	echinoderm microtubule associated protein like 6
404636	FAM45A	family with sequence similarity 45, member A
4077	NBR1	neighbor of BRCA1 gene 1
4134	MAP4	microtubule-associated protein 4
4152	MBD1	methyl-CpG binding domain protein 1
4154	MBNL1	muscleblind-like (Drosophila)
4253	CTAGE5	CTAGE family, member 5
4287	ATXN3	ataxin 3
440193	CCDC88C	coiled-coil domain containing 88C
440823	MIAT	myocardial infarction associated transcript (non-protein coding)
4515	MTCP1	mature T-cell proliferation 1
4649	MYO9A	myosin IXA
4676	NAP1L4	nucleosome assembly protein 1-like 4
4752	NEK3	NIMA (never in mitosis gene a)-related kinase 3
4817	NIT1	nitrilase 1
4820	NKTR	natural killer-tumor recognition sequence
4898	NRD1	nardilysin (N-arginine dibasic convertase)
4925	NUCB2	nucleobindin 2
4931	NVL	nuclear VCP-like
497661	C18orf32	chromosome 18 open reading frame 32
503538	A1BG-AS1	A1BG antisense RNA 1 (non-protein coding)
50488	MINK1	misshapen-like kinase 1
50717	DCAF8	DDB1 and CUL4 associated factor 8
50809	HP1BP3	heterochromatin protein 1, binding protein 3
50813	COPS7A	COP9 constitutive photomorphogenic homolog subunit 7A (Arabidopsis)
50854	C6orf48	chromosome 6 open reading frame 48
51000	SLC35B3	solute carrier family 35, member B3
51006	SLC35C2	solute carrier family 35, member C2
51011	FAHD2A	fumarylacetoacetate hydrolase domain containing 2A
51023	MRPS18C	mitochondrial ribosomal protein S18C
51043	ZBTB7B	zinc finger and BTB domain containing 7B
51057	WDPCP	WD repeat containing planar cell polarity effector
51076	CUTC	cutC copper transporter homolog (E. coli)
51088	KLHL5	kelch-like 5 (Drosophila)
51102	MECR	mitochondrial trans-2-enoyl-CoA reductase
51117	COQ4	coenzyme Q4 homolog (S. cerevisiae)
51147	ING4	inhibitor of growth family, member 4
51149	C5orf45	chromosome 5 open reading frame 45
51175	TUBE1	tubulin, epsilon 1
51194	IPO11	importin 11
51230	PHF20	PHD finger protein 20
51237	MZB1	marginal zone B and B1 cell-specific protein
51239	ANKRD39	ankyrin repeat domain 39
51249	TMEM69	transmembrane protein 69
51255	RNF181	ring finger protein 181
5127	CDK16	cyclin-dependent kinase 16
51279	C1RL	complement component 1, r subcomponent-like
51281	ANKMY1	ankyrin repeat and MYND domain containing 1
5129	CDK18	cyclin-dependent kinase 18
51307	FAM53C	family with sequence similarity 53, member C
51329	ARL6IP4	ADP-ribosylation-like factor 6 interacting protein 4

51351	ZNF117	zinc finger protein 117										
51389	RWDD1	RWD domain containing 1										
51427	ZNF107	zinc finger protein 107										
51428	DDX41	DEAD (Asp-Glu-Ala-Asp) box polypeptide 41										
51501	C11orf73	chromosome 11 open reading frame 73										
51535	PPHLN1	periphilin 1										
51542	VPS54	vacuolar protein sorting 54 homolog (S. cerevisiae)										
51562	MBIP	MAP3K12 binding inhibitory protein 1										
51574	LARP7	La ribonucleoprotein domain family, member 7										
51594	NBAS	neuroblastoma amplified sequence										
51601	LIPT1	lipoyltransferase 1										
51611	DPH5	DPH5 homolog (S. cerevisiae)										
51629	SLC25A39	solute carrier family 25, member 39										
51631	LUC7L2	LUC7-like 2 (S. cerevisiae)										
51642	MRPL48	mitochondrial ribosomal protein L48										
51657	STYXL1	serine/threonine/tyrosine interacting-like 1										
51693	TRAPPC2L	trafficking protein particle complex 2-like										
51699	VPS29	vacuolar protein sorting 29 homolog (S. cerevisiae)										
51755	CDK12	cyclin-dependent kinase 12										
5252	PHF1	PHD finger protein 1										
53346	TM6SF1	transmembrane 6 superfamily member 1										
5379	PMS2P1	postmeiotic segregation increased 2 pseudogene 1										
53917	RAB24	RAB24, member RAS oncogene family										
53938	PPIL3	peptidylprolyl isomerase (cyclophilin)-like 3										
54093	SETD4	SET domain containing 4										
541565	C8orf58	chromosome 8 open reading frame 58										
5428	POLG	polymerase (DNA directed), gamma										
54431	DNAJC10	DnaJ (Hsp40) homolog, subfamily C, member 10										
54436	SH3TC1	SH3 domain and tetratricopeptide repeats 1										
54476	RNF216	ring finger protein 216										
54503	ZDHHC13	zinc finger, DHHC-type containing 13										
54517	PUS7	pseudouridylate synthase 7 homolog (S. cerevisiae)										
54522	ANKRD16	ankyrin repeat domain 16										
54535	CCHCR1	coiled-coil alpha-helical rod protein 1										
54540	FAM193B	family with sequence similarity 193, member B										
54584	GNB1L	guanine nucleotide binding protein (G protein), beta polypeptide 1-like										
54585	LZTFL1	leucine zipper transcription factor-like 1										
54619	CCNJ	cyclin J										
54620	FBXL19	F-box and leucine-rich repeat protein 19										
54676	GTPBP2	GTP binding protein 2										
54758	KLHDC4	kelch domain containing 4										
54793	KCTD9	potassium channel tetramerisation domain containing 9										
54799	MBTD1	mbt domain containing 1										
54806	AHI1	Abelson helper integration site 1										
54808	DYM	dymeclin										
54814	QPCTL	glutaminyl-peptide cyclotransferase-like										
54823	SWT1	SWT1 RNA endoribonuclease homolog (S. cerevisiae)										
54830	NUP62CL	nucleoporin 62kDa C-terminal like										
54834	GDAP2	ganglioside induced differentiation associated protein 2										
54850	FBXL12	F-box and leucine-rich repeat protein 12										
54853	WDR55	WD repeat domain 55										
548645	DNAJC25	DnaJ (Hsp40) homolog, subfamily C , member 25										
54878	DPP8	dipeptidyl-peptidase 8										
54883	CWC25	CWC25 spliceosome-associated protein homolog (S. cerevisiae)										
54892	NCAPG2	non-SMC condensin II complex, subunit G2										
54896	PQLC2	PQ loop repeat containing 2										
54899	PXK	PX domain containing serine/threonine kinase										
54930	HAUS4	HAUS augmin-like complex, subunit 4										
54939	COMMD4	COMM domain containing 4										
54940	OCIAD1	OCIA domain containing 1										
54952	TRNAU1AP	tRNA selenocysteine 1 associated protein 1										
54973	CPSF3L	cleavage and polyadenylation specific factor 3-like										
54985	HCFC1R1	host cell factor C1 regulator 1 (XPO1 dependent)										
54991	C1orf159	chromosome 1 open reading frame 159										
550	AUP1	ancient ubiquitous protein 1										
55000	TUG1	taurine upregulated 1 (non-protein coding)										
55003	PAK1IP1	PAK1 interacting protein 1										
55005	RMND1	required for meiotic nuclear division 1 homolog (S. cerevisiae)										
55006	TRMT61B	tRNA methyltransferase 61 homolog B (S. cerevisiae)										
55007	FAM118A	family with sequence similarity 118, member A										
55015	PRPF39	PRP39 pre-mRNA processing factor 39 homolog (S. cerevisiae)										
55041	PLEKHB2	pleckstrin homology domain containing, family B (evectins) member 2										
55049	C19orf60	chromosome 19 open reading frame 60										
55052	MRPL20	mitochondrial ribosomal protein L20										
55054	ATG16L1	ATG16 autophagy related 16-like 1 (S. cerevisiae)										
55063	ZCWPW1	zinc finger, CW type with PWWP domain 1										
55086	CXorf57	chromosome X open reading frame 57										
55095	SAMD4B	sterile alpha motif domain containing 4B										
55102	ATG2B	ATG2 autophagy related 2 homolog B (S. cerevisiae)										
55132	LARP1B	La ribonucleoprotein domain family, member 1B										
55133	SRBD1	S1 RNA binding domain 1										
55147	RBM23	RNA binding motif protein 23										
55152	DALRD3	DALR anticodon binding domain containing 3										
55154	MSTO1	misato homolog 1 (Drosophila)										
55159	RFWD3	ring finger and WD repeat domain 3										
55161	TMEM33	transmembrane protein 33										
55180	LINS	lines homolog (Drosophila)										
55186	SLC25A36	solute carrier family 25, member 36										
55206	SBNO1	strawberry notch homolog 1 (Drosophila)										
55209	SETD5	SET domain containing 5										
55248	TMEM206	transmembrane protein 206										
55249	YY1AP1	YY1 associated protein 1										
55255	WDR41	WD repeat domain 41										
55262	C7orf43	chromosome 7 open reading frame 43										
55280	CWF19L1	CWF19-like 1, cell cycle control (S. pombe)										
55285	RBM41	RNA binding motif protein 41										
55291	PPP6R3	protein phosphatase 6, regulatory subunit 3										
55293	UEVLD	UEV and lactate/malate dehyrogenase domains										
55297	CCDC91	coiled-coil domain containing 91										
55322	C5orf22	chromosome 5 open reading frame 22										
55327	LIN7C	lin-7 homolog C (C. elegans)										
55335	NIPSNAP3B	nipsnap homolog 3B (C. elegans)										
55370	PPP4R1L	protein phosphatase 4, regulatory subunit 1-like										
55486	PARL	presenilin associated, rhomboid-like										
55536	CDCA7L	cell division cycle associated 7-like										
5554	PRH1	proline-rich protein HaeIII subfamily 1										
55582	KIF27	kinesin family member 27										
55608	ANKRD10	ankyrin repeat domain 10										
55613	MTMR8	myotubularin related protein 8										
55617	TASP1	taspase, threonine aspartase, 1										
55621	TRMT1	TRM1 tRNA methyltransferase 1 homolog (S. cerevisiae)										
55624	POMGNT1	protein O-linked mannose beta1,2-N-acetylglucosaminyltransferase										
55626	AMBRA1	autophagy/beclin-1 regulator 1										
55632	G2E3	G2/M-phase specific E3 ubiquitin protein ligase										
55633	TBC1D22B	TBC1 domain family, member 22B										
55644	OSGEP	O-sialoglycoprotein endopeptidase										
55653	BCAS4	breast carcinoma amplified sequence 4										
55657	ZNF692	zinc finger protein 692										
55665	URGCP	upregulator of cell proliferation										
55671	SMEK1	SMEK homolog 1, suppressor of mek1 (Dictyostelium)										
55687	TRMU	tRNA 5-methylaminomethyl-2-thiouridylate methyltransferase										
55689	YEATS2	YEATS domain containing 2										
55692	LUC7L	LUC7-like (S. cerevisiae)										
55700	MAP7D1	MAP7 domain containing 1										
55701	ARHGEF40	Rho guanine nucleotide exchange factor (GEF) 40										
55717	WDR11	WD repeat domain 11										
55728	N4BP2	NEDD4 binding protein 2										
55732	C1orf112	chromosome 1 open reading frame 112										
55739	CARKD	carbohydrate kinase domain containing										
55761	TTC17	tetratricopeptide repeat domain 17										
55764	IFT122	intraflagellar transport 122 homolog (Chlamydomonas)										
55769	ZNF83	zinc finger protein 83										
55777	MBD5	methyl-CpG binding domain protein 5										
55795	PCID2	PCI domain containing 2										
55813	UTP6	UTP6, small subunit (SSU) processome component, homolog (yeast)										
55814	BDP1	B double prime 1, subunit of RNA polymerase III transcription initiation factor IIIB										
55827	DCAF6	DDB1 and CUL4 associated factor 6										
55830	GLT8D1	glycosyltransferase 8 domain containing 1										
55833	UBAP2	ubiquitin associated protein 2										
55863	TMEM126B	transmembrane protein 126B										
55885	LMO3	LIM domain only 3 (rhombotin-like 2)										
55892	MYNN	myoneurin										
56061	UBFD1	ubiquitin family domain containing 1										
56063	TMEM234	transmembrane protein 234										
5612	PRKRIR	protein-kinase, interferon-inducible double stranded RNA dependent inhibitor, repressor of (P58 repressor)										
5616	PRKY	protein kinase, Y-linked, pseudogene										
56252	YLPM1	YLP motif containing 1										
56681	SAR1A	SAR1 homolog A (S. cerevisiae)										
56683	C21orf59	chromosome 21 open reading frame 59										
56731	SLC2A4RG	SLC2A4 regulator										
56834	GPR137	G protein-coupled receptor 137										
56890	MDM1	Mdm1 nuclear protein homolog (mouse)										
56919	DHX33	DEAH (Asp-Glu-Ala-His) box polypeptide 33										
56943	ENY2	enhancer of yellow 2 homolog (Drosophila)										
56946	C11orf30	chromosome 11 open reading frame 30										
56947	MFF	mitochondrial fission factor										
56948	SDR39U1	short chain dehydrogenase/reductase family 39U, member 1										
56986	DTWD1	DTW domain containing 1										
57092	PCNP	PEST proteolytic signal containing nuclear protein										
57116	ZNF695	zinc finger protein 695										
57117	INTS12	integrator complex subunit 12										
57130	ATP13A1	ATPase type 13A1										
57143	ADCK1	aarF domain containing kinase 1										
57148	RALGAPB	Ral GTPase activating protein, beta subunit (non-catalytic)										
57149	LYRM1	LYR motif containing 1										
57179	KIAA1191	KIAA1191										
57217	TTC7A	tetratricopeptide repeat domain 7A										
57223	SMEK2	SMEK homolog 2, suppressor of mek1 (Dictyostelium)										
57337	SENP7	SUMO1/sentrin specific peptidase 7										
57380	MRS2	MRS2 magnesium homeostasis factor homolog (S. cerevisiae)										
57409	MIF4GD	MIF4G domain containing										
57410	SCYL1	SCY1-like 1 (S. cerevisiae)										
57478	USP31	ubiquitin specific peptidase 31										
57492	ARID1B	AT rich interactive domain 1B (SWI1-like)										
57507	ZNF608	zinc finger protein 608										
57508	INTS2	integrator complex subunit 2										
57531	HACE1	HECT domain and ankyrin repeat containing, E3 ubiquitin protein ligase 1										
57539	WDR35	WD repeat domain 35										
57544	TXNDC16	thioredoxin domain containing 16										
57552	NCEH1	neutral cholesterol ester hydrolase 1										
57560	IFT80	intraflagellar transport 80 homolog (Chlamydomonas)										
57563	KLHL8	kelch-like 8 (Drosophila)										
57597	BAHCC1	BAH domain and coiled-coil containing 1										
57602	USP36	ubiquitin specific peptidase 36										
57605	PITPNM2	phosphatidylinositol transfer protein, membrane-associated 2										
57614	KIAA1468	KIAA1468										
57634	EP400	E1A binding protein p400										
57666	FBRSL1	fibrosin-like 1										
57669	EPB41L5	erythrocyte membrane protein band 4.1 like 5										
57683	ZDBF2	zinc finger, DBF-type containing 2										
57695	USP37	ubiquitin specific peptidase 37										
57696	DDX55	DEAD (Asp-Glu-Ala-Asp) box polypeptide 55										
57730	ANKRD36B	ankyrin repeat domain 36B										
57826	RAP2C	RAP2C, member of RAS oncogene family										
58487	CREBZF	CREB/ATF bZIP transcription factor										
58492	ZNF77	zinc finger protein 77										
585	BBS4	Bardet-Biedl syndrome 4										
58516	FAM60A	family with sequence similarity 60, member A										
5863	RGL2	ral guanine nucleotide dissociation stimulator-like 2										
5965	RECQL	RecQ protein-like (DNA helicase Q1-like)										
5990	RFX2	regulatory factor X, 2 (influences HLA class II expression)										
60343	FAM3A	family with sequence similarity 3, member A										
60528	ELAC2	elaC homolog 2 (E. coli)										
60558	GUF1	GUF1 GTPase homolog (S. cerevisiae)										
60561	RINT1	RAD50 interactor 1										
60592	SCOC	short coiled-coil protein										
60674	GAS5	growth arrest-specific 5 (non-protein coding)										
60686	C14orf93	chromosome 14 open reading frame 93										
6293	VPS52	vacuolar protein sorting 52 homolog (S. cerevisiae)										
6314	ATXN7	ataxin 7										
63877	FAM204A	family with sequence similarity 204, member A										
63892	THADA	thyroid adenoma associated										
63901	FAM111A	family with sequence similarity 111, member A										
63922	CHTF18	CTF18, chromosome transmission fidelity factor 18 homolog (S. cerevisiae)										
63979	FIGNL1	fidgetin-like 1										
6399	TRAPPC2	trafficking protein particle complex 2										
64005	MYO1G	myosin IG										
64062	RBM26	RNA binding motif protein 26										
64118	DUS1L	dihydrouridine synthase 1-like (S. cerevisiae)										
641590	NBPF20	neuroblastoma breakpoint family, member 20										
641638	SNHG6	small nucleolar RNA host gene 6 (non-protein coding)										
64172	OSGEPL1	O-sialoglycoprotein endopeptidase-like 1										
64210	MMS19	MMS19 nucleotide excision repair homolog (S. cerevisiae)										
64374	SIL1	SIL1 homolog, endoplasmic reticulum chaperone (S. cerevisiae)										
643749	TRAF3IP2-AS1	TRAF3IP2 antisense RNA 1 (non-protein coding)										
643836	ZFP62	zinc finger protein 62 homolog (mouse)										
643911	CRNDE	colorectal neoplasia differentially expressed (non-protein coding)										
64400	AKTIP	AKT interacting protein										
64418	TMEM168	transmembrane protein 168										
64420	SUSD1	sushi domain containing 1										
64427	TTC31	tetratricopeptide repeat domain 31										
64434	NOM1	nucleolar protein with MIF4G domain 1										
645784	ANKRD36BP2	ankyrin repeat domain 36B pseudogene 2										
64595	TTTY15	testis-specific transcript, Y-linked 15 (non-protein coding)										
64753	CCDC136	coiled-coil domain containing 136										
64754	SMYD3	SET and MYND domain containing 3										
64756	ATPAF1	ATP synthase mitochondrial F1 complex assembly factor 1										
64766	S100PBP	S100P binding protein										
64770	CCDC14	coiled-coil domain containing 14										
64776	C11orf1	chromosome 11 open reading frame 1										
64780	MICAL1	microtubule associated monoxygenase, calponin and LIM domain containing 1										
64788	LMF1	lipase maturation factor 1										
64847	SPATA20	spermatogenesis associated 20										
64927	TTC23	tetratricopeptide repeat domain 23										
64940	STAG3L4	stromal antigen 3-like 4										
64983	MRPL32	mitochondrial ribosomal protein L32										
65056	GPBP1	GC-rich promoter binding protein 1										
65065	NBEAL1	neurobeachin-like 1										
65084	TMEM135	transmembrane protein 135										
65117	RSRC2	arginine/serine-rich coiled-coil 2										
65123	INTS3	integrator complex subunit 3										
65125	WNK1	WNK lysine deficient protein kinase 1										
65250	C5orf42	chromosome 5 open reading frame 42										
65258	MPPE1	metallophosphoesterase 1										
65265	C8orf33	chromosome 8 open reading frame 33										
653121	ZBTB8A	zinc finger and BTB domain containing 8A										
653319	KIAA0895L	KIAA0895-like										
653784	MZT2A	mitotic spindle organizing protein 2A										
65979	PHACTR4	phosphatase and actin regulator 4										
65980	BRD9	bromodomain containing 9										
65992	DDRGK1	DDRGK domain containing 1										
65998	C11orf95	chromosome 11 open reading frame 95										
66005	CHID1	chitinase domain containing 1										
6603	SMARCD2	SWI/SNF related, matrix associated, actin dependent regulator of chromatin, subfamily d, member 2										
66036	MTMR9	myotubularin related protein 9										
6691	SPINK2	serine peptidase inhibitor, Kazal type 2 (acrosin-trypsin inhibitor)										
6830	SUPT6H	suppressor of Ty 6 homolog (S. cerevisiae)										
6835	SURF2	surfeit 2										
6871	TADA2A	transcriptional adaptor 2A										
6894	TARBP1	TAR (HIV-1) RNA binding protein 1										
6944	VPS72	vacuolar protein sorting 72 homolog (S. cerevisiae)										
7072	TIA1	TIA1 cytotoxic granule-associated RNA binding protein										
7075	TIE1	tyrosine kinase with immunoglobulin-like and EGF-like domains 1										
7226	TRPM2	transient receptor potential cation channel, subfamily M, member 2										
7227	TRPS1	trichorhinophalangeal syndrome I										
724102	SNHG4	small nucleolar RNA host gene 4 (non-protein coding)										
728411	GUSBP1	glucuronidase, beta pseudogene 1										
7342	UBP1	upstream binding protein 1 (LBP-1a)										
7347	UCHL3	ubiquitin carboxyl-terminal esterase L3 (ubiquitin thiolesterase)										
7404	UTY	ubiquitously transcribed tetratricopeptide repeat gene, Y-linked										
7443	VRK1	vaccinia related kinase 1										
752	FMNL1	formin-like 1										
7543	ZFX	zinc finger protein, X-linked										
7553	ZNF7	zinc finger protein 7										
757	TMEM50B	transmembrane protein 50B										
7597	ZBTB25	zinc finger and BTB domain containing 25										
7644	ZNF91	zinc finger protein 91										
7692	ZNF133	zinc finger protein 133										
7694	ZNF135	zinc finger protein 135										
7705	ZNF146	zinc finger protein 146										
7748	ZNF195	zinc finger protein 195										
7756	ZNF207	zinc finger protein 207										
7862	BRPF1	bromodomain and PHD finger containing, 1										
78995	C17orf53	chromosome 17 open reading frame 53										
79012	CAMKV	CaM kinase-like vesicle-associated										
79022	TMEM106C	transmembrane protein 106C										
79048	SECISBP2	SECIS binding protein 2										
79054	TRPM8	transient receptor potential cation channel, subfamily M, member 8										
79065	ATG9A	ATG9 autophagy related 9 homolog A (S. cerevisiae)										
79090	TRAPPC6A	trafficking protein particle complex 6A										
79091	METTL22	methyltransferase like 22										
79137	FAM134A	family with sequence similarity 134, member A										
79140	CCDC28B	coiled-coil domain containing 28B										
79149	ZSCAN5A	zinc finger and SCAN domain containing 5A										
79154	DHRS11	dehydrogenase/reductase (SDR family) member 11										
7917	BAG6	BCL2-associated athanogene 6										
79180	EFHD2	EF-hand domain family, member D2										
79228	THOC6	THO complex 6 homolog (Drosophila)										
79364	ZXDC	ZXD family zinc finger C										
79443	FYCO1	FYVE and coiled-coil domain containing 1										
79446	WDR25	WD repeat domain 25										
79568	C2orf47	chromosome 2 open reading frame 47										
79573	TTC13	tetratricopeptide repeat domain 13										
79590	MRPL24	mitochondrial ribosomal protein L24										
79600	TCTN1	tectonic family member 1										
79618	HMBOX1	homeobox containing 1										

79630	C1orf54	chromosome 1 open reading frame 54										
79673	ZNF329	zinc finger protein 329										
79675	FASTKD1	FAST kinase domains 1										
79703	C11orf80	chromosome 11 open reading frame 80										
79707	NOL9	nucleolar protein 9										
79712	GTDC1	glycosyltransferase-like domain containing 1										
79713	IGFLR1	IGF-like family receptor 1										
79719	AAGAB	alpha- and gamma-adaptin binding protein										
79734	KCTD17	potassium channel tetramerisation domain containing 17										
79763	ISOC2	isochorismatase domain containing 2										
79777	ACBD4	acyl-CoA binding domain containing 4										
79789	CLMN	calmin (calponin-like, transmembrane)										
79809	TTC21B	tetratricopeptide repeat domain 21B										
7982	ST7	suppression of tumorigenicity 7										
79830	ZMYM1	zinc finger, MYM-type 1										
79847	TMEM180	transmembrane protein 180										
79867	TCTN2	tectonic family member 2										
79915	ATAD5	ATPase family, AAA domain containing 5										
79918	SETD6	SET domain containing 6										
79930	DOK3	docking protein 3										
79932	KIAA0319L	KIAA0319-like										
79934	ADCK4	aarF domain containing kinase 4										
79956	ERMP1	endoplasmic reticulum metallopeptidase 1										
79957	PAQR6	progestin and adipoQ receptor family member VI										
79958	DENND1C	DENN/MADD domain containing 1C										
79963	ABCA11P	ATP-binding cassette, sub-family A (ABC1), member 11, pseudogene										
79979	TRMT2B	TRM2 tRNA methyltransferase 2 homolog B (S. cerevisiae)										
80017	C14orf159	chromosome 14 open reading frame 159										
80018	NAA25	N(alpha)-acetyltransferase 25, NatB auxiliary subunit										
80021	TMEM62	transmembrane protein 62										
80067	DCAF17	DDB1 and CUL4 associated factor 17										
80169	CTC1	CTS telomere maintenance complex component 1										
80174	DBF4B	DBF4 homolog B (S. cerevisiae)										
80179	MYO19	myosin XIX										
80199	FUZ	fuzzy homolog (Drosophila)										
80205	CHD9	chromodomain helicase DNA binding protein 9										
80208	SPG11	spastic paraplegia 11 (autosomal recessive)										
80210	ARMC9	armadillo repeat containing 9										
80213	TM2D3	TM2 domain containing 3										
80279	CDK5RAP3	CDK5 regulatory subunit associated protein 3										
80305	TRABD	TraB domain containing										
80307	FER1L4	fer-1-like 4 (C. elegans) pseudogene										
80342	TRAF3IP3	TRAF3 interacting protein 3										
80705	TSGA10	testis specific, 10										
80724	ACAD10	acyl-CoA dehydrogenase family, member 10										
80746	TSEN2	tRNA splicing endonuclease 2 homolog (S. cerevisiae)										
80755	AARSD1	alanyl-tRNA synthetase domain containing 1										
80764	THAP7	THAP domain containing 7										
8087	FXR1	fragile X mental retardation, autosomal homolog 1										
8131	NPRL3	nitrogen permease regulator-like 3 (S. cerevisiae)										
8148	TAF15	TAF15 RNA polymerase II, TATA box binding protein (TBP)-associated factor, 68kDa										
81532	MOB2	MOB kinase activator 2										
81550	TDRD3	tudor domain containing 3										
81576	CCDC130	coiled-coil domain containing 130										
81614	NIPA2	non imprinted in Prader-Willi/Angelman syndrome 2										
81669	CCNL2	cyclin L2										
81671	VMP1	vacuole membrane protein 1										
81790	RNF170	ring finger protein 170										
81844	TRIM56	tripartite motif containing 56										
81847	RNF146	ring finger protein 146										
81858	SHARPIN	SHANK-associated RH domain interactor										
81890	QTRT1	queuine tRNA-ribosyltransferase 1										
819	CAMLG	calcium modulating ligand										
8241	RBM10	RNA binding motif protein 10										
8242	KDM5C	lysine (K)-specific demethylase 5C										
8284	KDM5D	lysine (K)-specific demethylase 5D										
83416	FCRL5	Fc receptor-like 5										
83440	ADPGK	ADP-dependent glucokinase										
83449	PMFBP1	polyamine modulated factor 1 binding protein 1										
83451	ABHD11	abhydrolase domain containing 11										
83479	DDX59	DEAD (Asp-Glu-Ala-Asp) box polypeptide 59										
83480	PUS3	pseudouridylate synthase 3										
83596	BCL2L12	BCL2-like 12 (proline rich)										
83694	RPS6KL1	ribosomal protein S6 kinase-like 1										
83696	TRAPPC9	trafficking protein particle complex 9										
83707	TRPT1	tRNA phosphotransferase 1										
83787	ARMC10	armadillo repeat containing 10										
83856	FSD1L	fibronectin type III and SPRY domain containing 1-like										
83862	TMEM120A	transmembrane protein 120A										
83985	SPNS1	spinster homolog 1 (Drosophila)										
83989	FAM172A	family with sequence similarity 172, member A										
84058	WDR54	WD repeat domain 54										
84064	HDHD2	haloacid dehalogenase-like hydrolase domain containing 2										
84077	C3orf20	chromosome 3 open reading frame 20										
84083	ZRANB3	zinc finger, RAN-binding domain containing 3										
84105	PCBD2	pterin-4 alpha-carbinolamine dehydratase/dimerization cofactor of hepatocyte nuclear factor 1 alpha (TCF1) 2										
84108	PCGF6	polycomb group ring finger 6										
84128	WDR75	WD repeat domain 75										
84129	ACAD11	acyl-CoA dehydrogenase family, member 11										
84131	CEP78	centrosomal protein 78kDa										
84140	FAM161A	family with sequence similarity 161, member A										
84162	KIAA1109	KIAA1109										
84164	ASCC2	activating signal cointegrator 1 complex subunit 2										
84181	CHD6	chromodomain helicase DNA binding protein 6										
84196	USP48	ubiquitin specific peptidase 48										
84232	MAF1	MAF1 homolog (S. cerevisiae)										
84236	RHBDD1	rhomboid domain containing 1										
84239	ATP13A4	ATPase type 13A4										
84262	PSMG3	proteasome (prosome, macropain) assembly chaperone 3										
84268	RPAIN	RPA interacting protein										
84270	C9orf89	chromosome 9 open reading frame 89										
84286	TMEM175	transmembrane protein 175										
84287	ZDHHC16	zinc finger, DHHC-type containing 16										
84288	EFCAB2	EF-hand calcium binding domain 2										
84292	WDR83	WD repeat domain 83										
84304	NUDT22	nudix (nucleoside diphosphate linked moiety X)-type motif 22										
84314	TMEM107	transmembrane protein 107										
84326	C16orf13	chromosome 16 open reading frame 13										
84450	ZNF512	zinc finger protein 512										
84503	ZNF527	zinc finger protein 527										
84513	PPAPDC1B	phosphatidic acid phosphatase type 2 domain containing 1B										
84548	TMEM185A	transmembrane protein 185A										
8458	TTF2	transcription termination factor, RNA polymerase II										
84629	TNRC18	trinucleotide repeat containing 18										
84650	EBPL	emopamil binding protein-like										
84722	PSRC1	proline/serine-rich coiled-coil 1										
84725	PLEKHA8	pleckstrin homology domain containing, family A (phosphoinositide binding specific) member 8										
84726	PRRC2B	proline-rich coiled-coil 2B										
8473	OGT	O-linked N-acetylglucosamine (GlcNAc) transferase (UDP-N-acetylglucosamine:polypeptide-N-acetylglucosaminyl transferase)										
84749	USP30	ubiquitin specific peptidase 30										
84759	PCGF1	polycomb group ring finger 1										
84795	PYROXD2	pyridine nucleotide-disulphide oxidoreductase domain 2										
84820	POLR2J4	polymerase (RNA) II (DNA directed) polypeptide J4, pseudogene										
84824	FCRLA	Fc receptor-like A										
84833	USMG5	up-regulated during skeletal muscle growth 5 homolog (mouse)										
84901	NFATC2IP	nuclear factor of activated T-cells, cytoplasmic, calcineurin-dependent 2 interacting protein										
84902	CEP89	centrosomal protein 89kDa										
84923	FAM104A	family with sequence similarity 104, member A										
84940	CORO6	coronin 6										
84942	WDR73	WD repeat domain 73										
84958	SYTL1	synaptotagmin-like 1										
84964	ALKBH6	alkB, alkylation repair homolog 6 (E. coli)										
85014	TMEM141	transmembrane protein 141										
85015	USP45	ubiquitin specific peptidase 45										
85028	SNHG12	small nucleolar RNA host gene 12 (non-protein coding)										
8505	PARG	poly (ADP-ribose) glycohydrolase										
85302	FBF1	Fas (TNFRSF6) binding factor 1										
85444	LRRCC1	leucine rich repeat and coiled-coil domain containing 1										
8545	CGGBP1	CGG triplet repeat binding protein 1										
8563	THOC5	THO complex 5										
8576	STK16	serine/threonine kinase 16										
8635	RNASET2	ribonuclease T2										
8832	CD84	CD84 molecule										
8847	DLEU2	deleted in lymphocytic leukemia 2 (non-protein coding)										
8859	STK19	serine/threonine kinase 19										
8880	FUBP1	far upstream element (FUSE) binding protein 1										
8888	MCM3AP	minichromosome maintenance complex component 3 associated protein										
889	KRIT1	KRIT1, ankyrin repeat containing										
8904	CPNE1	copine I										
8906	AP1G2	adaptor-related protein complex 1, gamma 2 subunit										
89790	SIGLEC10	sialic acid binding Ig-like lectin 10										
89796	NAV1	neuron navigator 1										
89849	ATG16L2	ATG16 autophagy related 16-like 2 (S. cerevisiae)										
89894	TMEM116	transmembrane protein 116										
90007	MIDN	midnolin										
9016	SLC25A14	solute carrier family 25 (mitochondrial carrier, brain), member 14										
9044	BTAF1	BTAF1 RNA polymerase II, B-TFIID transcription factor-associated, 170kDa (Mot1 homolog, S. cerevisiae)										
90592	ZNF700	zinc finger protein 700										
90649	ZNF486	zinc finger protein 486										
90673	PPP1R3E	protein phosphatase 1, regulatory subunit 3E										
90835	C16orf93	chromosome 16 open reading frame 93										
91151	TIGD7	tigger transposable element derived 7										
91272	BOD1	biorientation of chromosomes in cell division 1										
91298	C12orf29	chromosome 12 open reading frame 29										
91316	GUSBP11	glucuronidase, beta pseudogene 11										
91319	DERL3	Der1-like domain family, member 3										
9139	CBFA2T2	core-binding factor, runt domain, alpha subunit 2; translocated to, 2										
9159	PCSK7	proprotein convertase subtilisin/kexin type 7										
91612	CHURC1	churchill domain containing 1										
91754	NEK9	NIMA (never in mitosis gene a)- related kinase 9										
9205	ZMYM5	zinc finger, MYM-type 5										
9208	LRRFIP1	leucine rich repeat (in FLII) interacting protein 1										
92105	INTS4	integrator complex subunit 4										
9213	XPR1	xenotropic and polytropic retrovirus receptor 1										
92170	MTG1	mitochondrial GTPase 1 homolog (S. cerevisiae)										
92270	ATP6AP1L	ATPase, H+ transporting, lysosomal accessory protein 1-like										
92305	TMEM129	transmembrane protein 129										
92399	MRRF	mitochondrial ribosome recycling factor										
92595	ZNF764	zinc finger protein 764										
92714	ARRDC1	arrestin domain containing 1										
9275	BCL7B	B-cell CLL/lymphoma 7B										
93129	ORAI3	ORAI calcium release-activated calcium modulator 3										
9317	PTER	phosphotriesterase related										
9324	HMGN3	high mobility group nucleosomal binding domain 3										
93627	TBCK	TBC1 domain containing kinase										
9382	COG1	component of oligomeric golgi complex 1										
9399	STOML1	stomatin (EPB72)-like 1										
9404	LPXN	leupaxin										
9406	ZRANB2	zinc finger, RAN-binding domain containing 2										
94101	ORMDL1	ORM1-like 1 (S. cerevisiae)										
94120	SYTL3	synaptotagmin-like 3										
9526	MPDU1	mannose-P-dolichol utilization defect 1										
9546	APBA3	amyloid beta (A4) precursor protein-binding, family A, member 3										
9557	CHD1L	chromodomain helicase DNA binding protein 1-like										
9567	GTPBP1	GTP binding protein 1										
9581	PREPL	prolyl endopeptidase-like										
9584	RBM39	RNA binding motif protein 39										
9643	MORF4L2	mortality factor 4 like 2										
9657	IQCB1	IQ motif containing B1										
9658	ZNF516	zinc finger protein 516										
9681	DEPDC5	DEP domain containing 5										
9685	CLINT1	clathrin interactor 1										
9699	RIMS2	regulating synaptic membrane exocytosis 2										
9701	PPP6R2	protein phosphatase 6, regulatory subunit 2										
9704	DHX34	DEAH (Asp-Glu-Ala-His) box polypeptide 34										
9725	TMEM63A	transmembrane protein 63A										
9753	ZSCAN12	zinc finger and SCAN domain containing 12										
9772	KIAA0195	KIAA0195										
9774	BCLAF1	BCL2-associated transcription factor 1										
9779	TBC1D5	TBC1 domain family, member 5										
9782	MATR3	matrin 3										
9790	BMS1	BMS1 homolog, ribosome assembly protein (yeast)										
9812	KIAA0141	KIAA0141										
9814	SFI1	Sfi1 homolog, spindle assembly associated (yeast)										
9849	ZNF518A	zinc finger protein 518A										
9851	KIAA0753	KIAA0753										
9859	CEP170	centrosomal protein 170kDa										
9860	LRIG2	leucine-rich repeats and immunoglobulin-like domains 2										
9877	ZC3H11A	zinc finger CCCH-type containing 11A										
9898	UBAP2L	ubiquitin associated protein 2-like										
9906	SLC35E2	solute carrier family 35, member E2										
9937	DCLRE1A	DNA cross-link repair 1A										
9960	USP3	ubiquitin specific peptidase 3										
9989	PPP4R1	protein phosphatase 4, regulatory subunit 1										

Supplementary Table 5: SU-DHL-6 Alternative Splicing MsigDB Analysis

Overlap Results												
Collection(s):	CP, CP:BIOCARTA, CP:KEGG, CP:REACTOME, H											
# overlaps shown:	10											
# genesets in collections:	1379											
# genes in comparison (n):	1824											
# genes in universe (N):	45956											
Gene Set Name	# Genes in Gene Set (K)	Description	# Genes in Overlap (k)	k/K	p-value	FDR q-value						
REACTOME_METABOLISM_OF_PROTEINS	518	Genes involved in Metabolism of proteins	78	0.1447	8.91E-29	1.23E-25						
REACTOME_IMMUNE_SYSTEM	933	Genes involved in Immune System	68	0.1615	1.88E-26	1.30E-23						
REACTOME_ADAPTIVE_IMMUNE_SYSTEM	539	Genes involved in Adaptive Immune System	57	0.1754	5.92E-23	2.42E-20						
REACTOME_CELL_CYCLE	421	Genes involved in Cell Cycle	85	0.1641	7.03E-23	2.42E-20						
REACTOME_CELL_CYCLE_MITOTIC	325	Genes involved in Cell Cycle, Mitotic	114	0.1222	3.66E-21	1.01E-18						
REACTOME_METABOLISM_OF_RNA	330	Genes involved in Metabolism of RNA	55	0.1667	2.06E-19	4.74E-17						
HALLMARK_E2F_TARGETS	200	Genes encoding cell cycle related targets of E2F transcription factors.	41	0.205	3.91E-18	6.74E-16						
HALLMARK_MYC_TARGETS_V1	200	A subgroup of genes regulated by MYC - version 1 (v1).	41	0.205	3.91E-18	6.74E-16						
REACTOME_METABOLISM_OF_MRNA	284	Genes involved in Metabolism of mRNA	47	0.1655	1.13E-16	1.73E-14						
HALLMARK_MITOTIC_SPINDLE	200	Genes important for mitotic spindle assembly.	39	0.195	1.53E-16	2.11E-14						
Gene/Gene Set Overlap Matrix												
Entrez Gene Id	Gene Symbol	Gene Description	REACTOME_METABOLISM_OF_PROTEINS	REACTOME_IMMUNE_SYSTEM	REACTOME_ADAPTIVE_IMMUNE_SYSTEM	REACTOME_CELL_CYCLE	REACTOME_CELL_CYCLE_MITOTIC	REACTOME_METABOLISM_OF_RNA	HALLMARK_E2F_TARGETS	HALLMARK_MYC_TARGETS_V1	REACTOME_METABOLISM_OF_MRNA	HALLMARK_MITOTIC_SPINDLE
6233	RPS27A	ribosomal protein S27a	REACTOME_METABOLISM_OF_PROTEINS	REACTOME_IMMUNE_SYSTEM	REACTOME_ADAPTIVE_IMMUNE_SYSTEM	REACTOME_CELL_CYCLE	REACTOME_CELL_CYCLE_MITOTIC	REACTOME_METABOLISM_OF_RNA			REACTOME_METABOLISM_OF_MRNA	
7311	UBA52	ubiquitin A-52 residue ribosomal protein fusion product 1	REACTOME_METABOLISM_OF_PROTEINS	REACTOME_IMMUNE_SYSTEM	REACTOME_ADAPTIVE_IMMUNE_SYSTEM	REACTOME_CELL_CYCLE	REACTOME_CELL_CYCLE_MITOTIC	REACTOME_METABOLISM_OF_RNA			REACTOME_METABOLISM_OF_MRNA	
55294	FBXW7	F-box and WD repeat domain containing 7	REACTOME_METABOLISM_OF_PROTEINS	REACTOME_IMMUNE_SYSTEM	REACTOME_ADAPTIVE_IMMUNE_SYSTEM							
9871	SEC24D	SEC24 family, member D (S. cerevisiae)	REACTOME_METABOLISM_OF_PROTEINS	REACTOME_IMMUNE_SYSTEM	REACTOME_ADAPTIVE_IMMUNE_SYSTEM							
9632	SEC24C	SEC24 family, member C (S. cerevisiae)	REACTOME_METABOLISM_OF_PROTEINS	REACTOME_IMMUNE_SYSTEM	REACTOME_ADAPTIVE_IMMUNE_SYSTEM							
1981	EIF4G1	eukaryotic translation initiation factor 4 gamma, 1	REACTOME_METABOLISM_OF_PROTEINS	REACTOME_IMMUNE_SYSTEM				REACTOME_METABOLISM_OF_RNA			REACTOME_METABOLISM_OF_MRNA	
1974	EIF4A2	eukaryotic translation initiation factor 4A2	REACTOME_METABOLISM_OF_PROTEINS	REACTOME_IMMUNE_SYSTEM				REACTOME_METABOLISM_OF_RNA			REACTOME_METABOLISM_OF_MRNA	
6194	RPS6	ribosomal protein S6	REACTOME_METABOLISM_OF_PROTEINS					REACTOME_METABOLISM_OF_RNA		HALLMARK_MYC_TARGETS_V1	REACTOME_METABOLISM_OF_MRNA	
6188	RPS3	ribosomal protein S3	REACTOME_METABOLISM_OF_PROTEINS					REACTOME_METABOLISM_OF_RNA		HALLMARK_MYC_TARGETS_V1	REACTOME_METABOLISM_OF_MRNA	
6193	RPS5	ribosomal protein S5	REACTOME_METABOLISM_OF_PROTEINS					REACTOME_METABOLISM_OF_RNA		HALLMARK_MYC_TARGETS_V1	REACTOME_METABOLISM_OF_MRNA	
6204	RPS10	ribosomal protein S10	REACTOME_METABOLISM_OF_PROTEINS					REACTOME_METABOLISM_OF_RNA		HALLMARK_MYC_TARGETS_V1	REACTOME_METABOLISM_OF_MRNA	
6164	RPL34	ribosomal protein L34	REACTOME_METABOLISM_OF_PROTEINS					REACTOME_METABOLISM_OF_RNA		HALLMARK_MYC_TARGETS_V1	REACTOME_METABOLISM_OF_MRNA	
6175	RPLP0	ribosomal protein, large, P0	REACTOME_METABOLISM_OF_PROTEINS					REACTOME_METABOLISM_OF_RNA		HALLMARK_MYC_TARGETS_V1	REACTOME_METABOLISM_OF_MRNA	
26986	PABPC1	poly(A) binding protein, cytoplasmic 1	REACTOME_METABOLISM_OF_PROTEINS					REACTOME_METABOLISM_OF_RNA		HALLMARK_MYC_TARGETS_V1	REACTOME_METABOLISM_OF_MRNA	
2107	ETF1	eukaryotic translation termination factor 1	REACTOME_METABOLISM_OF_PROTEINS					REACTOME_METABOLISM_OF_RNA		HALLMARK_MYC_TARGETS_V1	REACTOME_METABOLISM_OF_MRNA	
6206	RPS12	ribosomal protein S12	REACTOME_METABOLISM_OF_PROTEINS					REACTOME_METABOLISM_OF_RNA			REACTOME_METABOLISM_OF_MRNA	
6191	RPS4X	ribosomal protein S4, X-linked	REACTOME_METABOLISM_OF_PROTEINS					REACTOME_METABOLISM_OF_RNA			REACTOME_METABOLISM_OF_MRNA	
6201	RPS7	ribosomal protein S7	REACTOME_METABOLISM_OF_PROTEINS					REACTOME_METABOLISM_OF_RNA			REACTOME_METABOLISM_OF_MRNA	
6209	RPS15	ribosomal protein S15	REACTOME_METABOLISM_OF_PROTEINS					REACTOME_METABOLISM_OF_RNA			REACTOME_METABOLISM_OF_MRNA	
6230	RPS25	ribosomal protein S25	REACTOME_METABOLISM_OF_PROTEINS					REACTOME_METABOLISM_OF_RNA			REACTOME_METABOLISM_OF_MRNA	
6125	RPL5	ribosomal protein L5	REACTOME_METABOLISM_OF_PROTEINS					REACTOME_METABOLISM_OF_RNA			REACTOME_METABOLISM_OF_MRNA	
23521	RPL13A	ribosomal protein L13a	REACTOME_METABOLISM_OF_PROTEINS					REACTOME_METABOLISM_OF_RNA			REACTOME_METABOLISM_OF_MRNA	
11224	RPL35	ribosomal protein L35	REACTOME_METABOLISM_OF_PROTEINS					REACTOME_METABOLISM_OF_RNA			REACTOME_METABOLISM_OF_MRNA	
4736	RPL10A	ribosomal protein L10a	REACTOME_METABOLISM_OF_PROTEINS					REACTOME_METABOLISM_OF_RNA			REACTOME_METABOLISM_OF_MRNA	
6130	RPL7A	ribosomal protein L7a	REACTOME_METABOLISM_OF_PROTEINS					REACTOME_METABOLISM_OF_RNA			REACTOME_METABOLISM_OF_MRNA	
6134	RPL10	ribosomal protein L10	REACTOME_METABOLISM_OF_PROTEINS					REACTOME_METABOLISM_OF_RNA			REACTOME_METABOLISM_OF_MRNA	
6139	RPL17	ribosomal protein L17	REACTOME_METABOLISM_OF_PROTEINS					REACTOME_METABOLISM_OF_RNA			REACTOME_METABOLISM_OF_MRNA	
6144	RPL21	ribosomal protein L21	REACTOME_METABOLISM_OF_PROTEINS					REACTOME_METABOLISM_OF_RNA			REACTOME_METABOLISM_OF_MRNA	
6154	RPL26	ribosomal protein L26	REACTOME_METABOLISM_OF_PROTEINS					REACTOME_METABOLISM_OF_RNA			REACTOME_METABOLISM_OF_MRNA	
6156	RPL30	ribosomal protein L30	REACTOME_METABOLISM_OF_PROTEINS					REACTOME_METABOLISM_OF_RNA			REACTOME_METABOLISM_OF_MRNA	
6159	RPL29	ribosomal protein L29	REACTOME_METABOLISM_OF_PROTEINS					REACTOME_METABOLISM_OF_RNA			REACTOME_METABOLISM_OF_MRNA	
6171	RPL41	ribosomal protein L41	REACTOME_METABOLISM_OF_PROTEINS					REACTOME_METABOLISM_OF_RNA			REACTOME_METABOLISM_OF_MRNA	
6173	RPL36A	ribosomal protein L36a	REACTOME_METABOLISM_OF_PROTEINS					REACTOME_METABOLISM_OF_RNA			REACTOME_METABOLISM_OF_MRNA	
6208	RPS14	ribosomal protein S14	REACTOME_METABOLISM_OF_PROTEINS					REACTOME_METABOLISM_OF_RNA			REACTOME_METABOLISM_OF_MRNA	
10528	NOP56	NOP56 ribonucleoprotein homolog (yeast)	REACTOME_METABOLISM_OF_PROTEINS						HALLMARK_E2F_TARGETS	HALLMARK_MYC_TARGETS_V1		
3329	HSPD1	heat shock 60kDa protein 1 (chaperonin)	REACTOME_METABOLISM_OF_PROTEINS							HALLMARK_MYC_TARGETS_V1		
6950	TCP1	t-complex 1	REACTOME_METABOLISM_OF_PROTEINS							HALLMARK_MYC_TARGETS_V1		
1968	EIF2S3	eukaryotic translation initiation factor 2, subunit 3 gamma, 52kDa	REACTOME_METABOLISM_OF_PROTEINS									
3646	EIF3E	eukaryotic translation initiation factor 3, subunit E	REACTOME_METABOLISM_OF_PROTEINS									
27335	EIF3K	eukaryotic translation initiation factor 3, subunit K	REACTOME_METABOLISM_OF_PROTEINS									
6747	SSR3	signal sequence receptor, gamma (translocon-associated protein gamma)	REACTOME_METABOLISM_OF_PROTEINS									
8893	EIF2B5	eukaryotic translation initiation factor 2B, subunit 5 epsilon, 82kDa	REACTOME_METABOLISM_OF_PROTEINS									
8890	EIF2B4	eukaryotic translation initiation factor 2B, subunit 4 delta, 67kDa	REACTOME_METABOLISM_OF_PROTEINS									
1936	EEF1D	eukaryotic translation elongation factor 1 delta (guanine nucleotide exchange protein	REACTOME_METABOLISM_OF_PROTEINS									
10063	COX17	COX17 cytochrome c oxidase assembly homolog (S. cerevisiae)	REACTOME_METABOLISM_OF_PROTEINS									
25870	SUMF2	sulfatase modifying factor 2	REACTOME_METABOLISM_OF_PROTEINS									
6901	TAZ	tafazzin	REACTOME_METABOLISM_OF_PROTEINS									
8813	DPM1	dolichyl-phosphate mannosyltransferase polypeptide 1, catalytic subunit	REACTOME_METABOLISM_OF_PROTEINS									
56052	ALG1	asparagine-linked glycosylation 1, beta-1,4-mannosyltransferase homolog (S. cerevisia	REACTOME_METABOLISM_OF_PROTEINS									
57171	DOLPP1	dolichyl pyrophosphate phosphatase 1	REACTOME_METABOLISM_OF_PROTEINS									
85365	ALG2	sparagine-linked glycosylation 2, alpha-1,3-mannosyltransferase homolog (S. cerevisia	REACTOME_METABOLISM_OF_PROTEINS									
10195	ALG3	sparagine-linked glycosylation 3, alpha-1,3- mannosyltransferase homolog (S. cerevisia	REACTOME_METABOLISM_OF_PROTEINS									
440138	ALG11	asparagine-linked glycosylation 11, alpha-1,2-mannosyltransferase homolog (yeast)	REACTOME_METABOLISM_OF_PROTEINS									
79868	ALG13	asparagine-linked glycosylation 13 homolog (S. cerevisiae)	REACTOME_METABOLISM_OF_PROTEINS									
4351	MPI	mannose phosphate isomerase	REACTOME_METABOLISM_OF_PROTEINS									
29925	GMPPB	GDP-mannose pyrophosphorylase B	REACTOME_METABOLISM_OF_PROTEINS									
29926	GMPPA	GDP-mannose pyrophosphorylase A	REACTOME_METABOLISM_OF_PROTEINS									
8703	B4GALT3	UDP-Gal:betaGlcNAc beta 1,4- galactosyltransferase, polypeptide 3	REACTOME_METABOLISM_OF_PROTEINS									
23193	GANAB	glucosidase, alpha; neutral AB	REACTOME_METABOLISM_OF_PROTEINS									
4245	MGAT1	mannosyl (alpha-1,3-)-glycoprotein beta-1,2-N-acetylglucosaminyltransferase	REACTOME_METABOLISM_OF_PROTEINS									
10113	PREB	prolactin regulatory element binding	REACTOME_METABOLISM_OF_PROTEINS									
55741	EDEM2	ER degradation enhancer, mannosidase alpha-like 2	REACTOME_METABOLISM_OF_PROTEINS									
55757	UGGT2	UDP-glucose glycoprotein glucosyltransferase 2	REACTOME_METABOLISM_OF_PROTEINS									
80267	EDEM3	ER degradation enhancer, mannosidase alpha-like 3	REACTOME_METABOLISM_OF_PROTEINS									
2650	GCNT1	glucosaminyl (N-acetyl) transferase 1, core 2	REACTOME_METABOLISM_OF_PROTEINS									
23556	PIGN	phosphatidylinositol glycan anchor biosynthesis, class N	REACTOME_METABOLISM_OF_PROTEINS									
51227	PIGP	phosphatidylinositol glycan anchor biosynthesis, class P	REACTOME_METABOLISM_OF_PROTEINS									
5279	PIGC	phosphatidylinositol glycan anchor biosynthesis, class C	REACTOME_METABOLISM_OF_PROTEINS									
5281	PIGF	phosphatidylinositol glycan anchor biosynthesis, class F	REACTOME_METABOLISM_OF_PROTEINS									
84720	PIGO	phosphatidylinositol glycan anchor biosynthesis, class O	REACTOME_METABOLISM_OF_PROTEINS									
51604	PIGT	phosphatidylinositol glycan anchor biosynthesis, class T	REACTOME_METABOLISM_OF_PROTEINS									
8733	GPAA1	glycosylphosphatidylinositol anchor attachment protein 1 homolog (yeast)	REACTOME_METABOLISM_OF_PROTEINS									
2677	GGCX	gamma-glutamyl carboxylase	REACTOME_METABOLISM_OF_PROTEINS									
146712	B3GNTL1	UDP-GlcNAc:betaGal beta-1,3-N-acetylglucosaminyltransferase-like 1	REACTOME_METABOLISM_OF_PROTEINS									
1431	CS	citrate synthase	REACTOME_METABOLISM_OF_PROTEINS									
3421	IDH3G	isocitrate dehydrogenase 3 (NAD+) gamma	REACTOME_METABOLISM_OF_PROTEINS									
150274	HSCB	HscB iron-sulfur cluster co-chaperone homolog (E. coli)	REACTOME_METABOLISM_OF_PROTEINS									
4580	MTX1	metaxin 1	REACTOME_METABOLISM_OF_PROTEINS									
54543	TOMM7	translocase of outer mitochondrial membrane 7 homolog (yeast)	REACTOME_METABOLISM_OF_PROTEINS									
617	BCS1L	BCS1-like (S. cerevisiae)	REACTOME_METABOLISM_OF_PROTEINS									
653252	TIMM23B	translocase of inner mitochondrial membrane 23 homolog B (yeast)	REACTOME_METABOLISM_OF_PROTEINS									
10694	CCT8	chaperonin containing TCP1, subunit 8 (theta)	REACTOME_METABOLISM_OF_PROTEINS									
54461	FBXW5	F-box and WD repeat domain containing 5	REACTOME_METABOLISM_OF_PROTEINS									
5204	PFDN5	prefoldin subunit 5	REACTOME_METABOLISM_OF_PROTEINS									
6902	TBCA	tubulin folding cofactor A	REACTOME_METABOLISM_OF_PROTEINS									
5699	PSMB10	proteasome (prosome, macropain) subunit, beta type, 10		REACTOME_IMMUNE_SYSTEM	REACTOME_ADAPTIVE_IMMUNE_SYSTEM	REACTOME_CELL_CYCLE	REACTOME_CELL_CYCLE_MITOTIC	REACTOME_METABOLISM_OF_RNA			REACTOME_METABOLISM_OF_MRNA	
5720	PSME1	proteasome (prosome, macropain) activator subunit 1 (PA28 alpha)		REACTOME_IMMUNE_SYSTEM	REACTOME_ADAPTIVE_IMMUNE_SYSTEM	REACTOME_CELL_CYCLE	REACTOME_CELL_CYCLE_MITOTIC	REACTOME_METABOLISM_OF_RNA			REACTOME_METABOLISM_OF_MRNA	
5717	PSMD11	proteasome (prosome, macropain) 26S subunit, non-ATPase, 11		REACTOME_IMMUNE_SYSTEM	REACTOME_ADAPTIVE_IMMUNE_SYSTEM	REACTOME_CELL_CYCLE	REACTOME_CELL_CYCLE_MITOTIC	REACTOME_METABOLISM_OF_RNA			REACTOME_METABOLISM_OF_MRNA	
9861	PSMD6	proteasome (prosome, macropain) 26S subunit, non-ATPase, 6		REACTOME_IMMUNE_SYSTEM	REACTOME_ADAPTIVE_IMMUNE_SYSTEM	REACTOME_CELL_CYCLE	REACTOME_CELL_CYCLE_MITOTIC	REACTOME_METABOLISM_OF_RNA			REACTOME_METABOLISM_OF_MRNA	
5705	PSMC5	proteasome (prosome, macropain) 26S subunit, ATPase, 5		REACTOME_IMMUNE_SYSTEM	REACTOME_ADAPTIVE_IMMUNE_SYSTEM	REACTOME_CELL_CYCLE	REACTOME_CELL_CYCLE_MITOTIC	REACTOME_METABOLISM_OF_RNA			REACTOME_METABOLISM_OF_MRNA	
5710	PSMD4	proteasome (prosome, macropain) 26S subunit, non-ATPase, 4		REACTOME_IMMUNE_SYSTEM	REACTOME_ADAPTIVE_IMMUNE_SYSTEM	REACTOME_CELL_CYCLE	REACTOME_CELL_CYCLE_MITOTIC	REACTOME_METABOLISM_OF_RNA			REACTOME_METABOLISM_OF_MRNA	
9493	KIF23	kinesin family member 23		REACTOME_IMMUNE_SYSTEM	REACTOME_ADAPTIVE_IMMUNE_SYSTEM	REACTOME_CELL_CYCLE	REACTOME_CELL_CYCLE_MITOTIC					HALLMARK_MITOTIC_SPINDLE
11065	UBE2C	ubiquitin-conjugating enzyme E2C		REACTOME_IMMUNE_SYSTEM	REACTOME_ADAPTIVE_IMMUNE_SYSTEM	REACTOME_CELL_CYCLE	REACTOME_CELL_CYCLE_MITOTIC					
81930	KIF18A	kinesin family member 18A		REACTOME_IMMUNE_SYSTEM	REACTOME_ADAPTIVE_IMMUNE_SYSTEM	REACTOME_CELL_CYCLE	REACTOME_CELL_CYCLE_MITOTIC					
5527	PPP2R5C	protein phosphatase 2, regulatory subunit B', gamma		REACTOME_IMMUNE_SYSTEM	REACTOME_ADAPTIVE_IMMUNE_SYSTEM	REACTOME_CELL_CYCLE	REACTOME_CELL_CYCLE_MITOTIC					
10121	ACTR1A	ARP1 actin-related protein 1 homolog A, centractin alpha (yeast)		REACTOME_IMMUNE_SYSTEM	REACTOME_ADAPTIVE_IMMUNE_SYSTEM	REACTOME_CELL_CYCLE	REACTOME_CELL_CYCLE_MITOTIC					
11258	DCTN3	dynactin 3 (p22)		REACTOME_IMMUNE_SYSTEM	REACTOME_ADAPTIVE_IMMUNE_SYSTEM	REACTOME_CELL_CYCLE	REACTOME_CELL_CYCLE_MITOTIC					
51434	ANAPC7	anaphase promoting complex subunit 7		REACTOME_IMMUNE_SYSTEM	REACTOME_ADAPTIVE_IMMUNE_SYSTEM	REACTOME_CELL_CYCLE	REACTOME_CELL_CYCLE_MITOTIC					
8881	CDC16	cell division cycle 16 homolog (S. cerevisiae)		REACTOME_IMMUNE_SYSTEM	REACTOME_ADAPTIVE_IMMUNE_SYSTEM	REACTOME_CELL_CYCLE	REACTOME_CELL_CYCLE_MITOTIC					
10054	UBA2	ubiquitin-like modifier activating enzyme 2		REACTOME_IMMUNE_SYSTEM	REACTOME_ADAPTIVE_IMMUNE_SYSTEM					HALLMARK_MYC_TARGETS_V1		
3832	KIF11	kinesin family member 11		REACTOME_IMMUNE_SYSTEM	REACTOME_ADAPTIVE_IMMUNE_SYSTEM							HALLMARK_MITOTIC_SPINDLE
4690	NCK1	NCK adaptor protein 1		REACTOME_IMMUNE_SYSTEM	REACTOME_ADAPTIVE_IMMUNE_SYSTEM							HALLMARK_MITOTIC_SPINDLE
3831	KLC1	kinesin light chain 1		REACTOME_IMMUNE_SYSTEM	REACTOME_ADAPTIVE_IMMUNE_SYSTEM							HALLMARK_MITOTIC_SPINDLE
6921	TCEB1	transcription elongation factor B (SIII), polypeptide 1 (15kDa, elongin C)		REACTOME_IMMUNE_SYSTEM	REACTOME_ADAPTIVE_IMMUNE_SYSTEM							
3932	LCK	lymphocyte-specific protein tyrosine kinase		REACTOME_IMMUNE_SYSTEM	REACTOME_ADAPTIVE_IMMUNE_SYSTEM							
164	AP1G1	adaptor-related protein complex 1, gamma 1 subunit		REACTOME_IMMUNE_SYSTEM	REACTOME_ADAPTIVE_IMMUNE_SYSTEM							
8905	AP1S2	adaptor-related protein complex 1, sigma 2 subunit		REACTOME_IMMUNE_SYSTEM	REACTOME_ADAPTIVE_IMMUNE_SYSTEM							
10075	HUWE1	HECT, UBA and WWE domain containing 1		REACTOME_IMMUNE_SYSTEM	REACTOME_ADAPTIVE_IMMUNE_SYSTEM							
9820	CUL7	cullin 7		REACTOME_IMMUNE_SYSTEM	REACTOME_ADAPTIVE_IMMUNE_SYSTEM							
7317	UBA1	ubiquitin-like modifier activating enzyme 1		REACTOME_IMMUNE_SYSTEM	REACTOME_ADAPTIVE_IMMUNE_SYSTEM							
7326	UBE2G1	ubiquitin-conjugating enzyme E2G 1		REACTOME_IMMUNE_SYSTEM	REACTOME_ADAPTIVE_IMMUNE_SYSTEM							
118424	UBE2J2	ubiquitin-conjugating enzyme E2, J2		REACTOME_IMMUNE_SYSTEM	REACTOME_ADAPTIVE_IMMUNE_SYSTEM							
26259	FBXW8	F-box and WD repeat domain containing 8		REACTOME_IMMUNE_SYSTEM	REACTOME_ADAPTIVE_IMMUNE_SYSTEM							
55236	UBA6	ubiquitin-like modifier activating enzyme 6		REACTOME_IMMUNE_SYSTEM	REACTOME_ADAPTIVE_IMMUNE_SYSTEM							
65264	UBE2Z	ubiquitin-conjugating enzyme E2Z		REACTOME_IMMUNE_SYSTEM	REACTOME_ADAPTIVE_IMMUNE_SYSTEM							
89910	UBE3B	ubiquitin protein ligase E3B		REACTOME_IMMUNE_SYSTEM	REACTOME_ADAPTIVE_IMMUNE_SYSTEM							
92912	UBE2Q2	ubiquitin-conjugating enzyme E2Q family member 2		REACTOME_IMMUNE_SYSTEM	REACTOME_ADAPTIVE_IMMUNE_SYSTEM							
10193	RNF41	ring finger protein 41		REACTOME_IMMUNE_SYSTEM	REACTOME_ADAPTIVE_IMMUNE_SYSTEM							
4012	LNPEP	leucyl/cystinyl aminopeptidase		REACTOME_IMMUNE_SYSTEM	REACTOME_ADAPTIVE_IMMUNE_SYSTEM							
27252	KLHL20	kelch-like 20 (Drosophila)		REACTOME_IMMUNE_SYSTEM	REACTOME_ADAPTIVE_IMMUNE_SYSTEM							
63891	RNF123	ring finger protein 123		REACTOME_IMMUNE_SYSTEM	REACTOME_ADAPTIVE_IMMUNE_SYSTEM							
55182	RNF220	ring finger protein 220		REACTOME_IMMUNE_SYSTEM	REACTOME_ADAPTIVE_IMMUNE_SYSTEM							
7174	TPP2	tripeptidyl peptidase II		REACTOME_IMMUNE_SYSTEM	REACTOME_ADAPTIVE_IMMUNE_SYSTEM							
79876	UBA5	ubiquitin-like modifier activating enzyme 5		REACTOME_IMMUNE_SYSTEM	REACTOME_ADAPTIVE_IMMUNE_SYSTEM							
90678	LRSAM1	leucine rich repeat and sterile alpha motif containing 1		REACTOME_IMMUNE_SYSTEM	REACTOME_ADAPTIVE_IMMUNE_SYSTEM							
9666	DZIP3	DAZ interacting protein 3, zinc finger		REACTOME_IMMUNE_SYSTEM	REACTOME_ADAPTIVE_IMMUNE_SYSTEM							
5476	CTSA	cathepsin A		REACTOME_IMMUNE_SYSTEM	REACTOME_ADAPTIVE_IMMUNE_SYSTEM							
5291	PIK3CB	phosphoinositide-3-kinase, catalytic, beta polypeptide		REACTOME_IMMUNE_SYSTEM	REACTOME_ADAPTIVE_IMMUNE_SYSTEM							
5335	PLCG1	phospholipase C, gamma 1		REACTOME_IMMUNE_SYSTEM	REACTOME_ADAPTIVE_IMMUNE_SYSTEM							
3551	IKBKB	inhibitor of kappa light polypeptide gene enhancer in B-cells, kinase beta		REACTOME_IMMUNE_SYSTEM	REACTOME_ADAPTIVE_IMMUNE_SYSTEM							
973	CD79A	CD79a molecule, immunoglobulin-associated alpha		REACTOME_IMMUNE_SYSTEM	REACTOME_ADAPTIVE_IMMUNE_SYSTEM							
1147	CHUK	conserved helix-loop-helix ubiquitous kinase		REACTOME_IMMUNE_SYSTEM	REACTOME_ADAPTIVE_IMMUNE_SYSTEM							
5336	PLCG2	phospholipase C, gamma 2 (phosphatidylinositol-specific)		REACTOME_IMMUNE_SYSTEM	REACTOME_ADAPTIVE_IMMUNE_SYSTEM							
930	CD19	CD19 molecule		REACTOME_IMMUNE_SYSTEM	REACTOME_ADAPTIVE_IMMUNE_SYSTEM							
974	CD79B	CD79b molecule, immunoglobulin-associated beta		REACTOME_IMMUNE_SYSTEM	REACTOME_ADAPTIVE_IMMUNE_SYSTEM							
118788	PIK3AP1	phosphoinositide-3-kinase adaptor protein 1		REACTOME_IMMUNE_SYSTEM	REACTOME_ADAPTIVE_IMMUNE_SYSTEM							
208	AKT2	v-akt murine thymoma viral oncogene homolog 2		REACTOME_IMMUNE_SYSTEM	REACTOME_ADAPTIVE_IMMUNE_SYSTEM							
6199	RPS6KB2	ribosomal protein S6 kinase, 70kDa, polypeptide 2		REACTOME_IMMUNE_SYSTEM	REACTOME_ADAPTIVE_IMMUNE_SYSTEM							
2475	MTOR	mechanistic target of rapamycin (serine/threonine kinase)		REACTOME_IMMUNE_SYSTEM	REACTOME_ADAPTIVE_IMMUNE_SYSTEM							
7249	TSC2	tuberous sclerosis 2		REACTOME_IMMUNE_SYSTEM	REACTOME_ADAPTIVE_IMMUNE_SYSTEM							
64223	MLST8	MTOR associated protein, LST8 homolog (S. cerevisiae)		REACTOME_IMMUNE_SYSTEM	REACTOME_ADAPTIVE_IMMUNE_SYSTEM							
25780	RASGRP3	RAS guanyl releasing protein 3 (calcium and DAG-regulated)		REACTOME_IMMUNE_SYSTEM	REACTOME_ADAPTIVE_IMMUNE_SYSTEM							
5966	REL	v-rel reticuloendotheliosis viral oncogene homolog (avian)		REACTOME_IMMUNE_SYSTEM	REACTOME_ADAPTIVE_IMMUNE_SYSTEM							
3710	ITPR3	inositol 1,4,5-trisphosphate receptor, type 3		REACTOME_IMMUNE_SYSTEM	REACTOME_ADAPTIVE_IMMUNE_SYSTEM							
4689	NCF4	neutrophil cytosolic factor 4, 40kDa		REACTOME_IMMUNE_SYSTEM	REACTOME_ADAPTIVE_IMMUNE_SYSTEM							
5788	PTPRC	protein tyrosine phosphatase, receptor type, C		REACTOME_IMMUNE_SYSTEM	REACTOME_ADAPTIVE_IMMUNE_SYSTEM							
55824	PAG1	phosphoprotein associated with glycosphingolipid microdomains 1		REACTOME_IMMUNE_SYSTEM	REACTOME_ADAPTIVE_IMMUNE_SYSTEM							
3108	HLA-DMA	major histocompatibility complex, class II, DM alpha		REACTOME_IMMUNE_SYSTEM	REACTOME_ADAPTIVE_IMMUNE_SYSTEM							
3112	HLA-DOB	major histocompatibility complex, class II, DO beta		REACTOME_IMMUNE_SYSTEM	REACTOME_ADAPTIVE_IMMUNE_SYSTEM							
3683	ITGAL	, alpha L (antigen CD11A (p180), lymphocyte function-associated antigen 1; alpha polypeptide)		REACTOME_IMMUNE_SYSTEM	REACTOME_ADAPTIVE_IMMUNE_SYSTEM							
1785	DNM2	dynamin 2		REACTOME_IMMUNE_SYSTEM	REACTOME_ADAPTIVE_IMMUNE_SYSTEM							
10120	ACTR1B	ARP1 actin-related protein 1 homolog B, centractin beta (yeast)		REACTOME_IMMUNE_SYSTEM	REACTOME_ADAPTIVE_IMMUNE_SYSTEM							
114876	OSBPL1A	oxysterol binding protein-like 1A		REACTOME_IMMUNE_SYSTEM	REACTOME_ADAPTIVE_IMMUNE_SYSTEM							
5908	RAP1B	RAP1B, member of RAS oncogene family		REACTOME_IMMUNE_SYSTEM	REACTOME_ADAPTIVE_IMMUNE_SYSTEM							
3695	ITGB7	integrin, beta 7		REACTOME_IMMUNE_SYSTEM	REACTOME_ADAPTIVE_IMMUNE_SYSTEM							
120425	AMICA1	adhesion molecule, interacts with CXADR antigen 1		REACTOME_IMMUNE_SYSTEM	REACTOME_ADAPTIVE_IMMUNE_SYSTEM							
3385	ICAM3	intercellular adhesion molecule 3		REACTOME_IMMUNE_SYSTEM	REACTOME_ADAPTIVE_IMMUNE_SYSTEM							
23108	RAP1GAP2	RAP1 GTPase activating protein 2		REACTOME_IMMUNE_SYSTEM	REACTOME_ADAPTIVE_IMMUNE_SYSTEM							
9972	NUP153	nucleoporin 153kDa		REACTOME_IMMUNE_SYSTEM				REACTOME_METABOLISM_OF_RNA	HALLMARK_E2F_TARGETS			
9883	POM121	POM121 membrane glycoprotein		REACTOME_IMMUNE_SYSTEM				REACTOME_METABOLISM_OF_RNA				
8086	AAAS	achalasia, adrenocortical insufficiency, alacrimia		REACTOME_IMMUNE_SYSTEM				REACTOME_METABOLISM_OF_RNA				
11097	NUPL2	nucleoporin like 2		REACTOME_IMMUNE_SYSTEM				REACTOME_METABOLISM_OF_RNA				
23225	NUP210	nucleoporin 210kDa		REACTOME_IMMUNE_SYSTEM				REACTOME_METABOLISM_OF_RNA				
23511	NUP188	nucleoporin 188kDa		REACTOME_IMMUNE_SYSTEM				REACTOME_METABOLISM_OF_RNA				
23636	NUP62	nucleoporin 62kDa		REACTOME_IMMUNE_SYSTEM				REACTOME_METABOLISM_OF_RNA				
1982	EIF4G2	eukaryotic translation initiation factor 4 gamma, 2		REACTOME_IMMUNE_SYSTEM						HALLMARK_MYC_TARGETS_V1		
5094	PCBP2	poly(rC) binding protein 2		REACTOME_IMMUNE_SYSTEM								
3836	KPNA1	karyopherin alpha 1 (importin alpha 5)		REACTOME_IMMUNE_SYSTEM								
3636	INPPL1	inositol polyphosphate phosphatase-like 1		REACTOME_IMMUNE_SYSTEM								
9470	EIF4E2	eukaryotic translation initiation factor 4E family member 2		REACTOME_IMMUNE_SYSTEM								
3661	IRF3	interferon regulatory factor 3		REACTOME_IMMUNE_SYSTEM								
4600	MX2	myxovirus (influenza virus) resistance 2 (mouse)		REACTOME_IMMUNE_SYSTEM								
5599	MAPK8	mitogen-activated protein kinase 8		REACTOME_IMMUNE_SYSTEM								
3665	IRF7	interferon regulatory factor 7		REACTOME_IMMUNE_SYSTEM								
7186	TRAF2	TNF receptor-associated factor 2		REACTOME_IMMUNE_SYSTEM								
6773	STAT2	signal transducer and activator of transcription 2, 113kDa		REACTOME_IMMUNE_SYSTEM								
51447	IP6K2	inositol hexakisphosphate kinase 2		REACTOME_IMMUNE_SYSTEM								
7297	TYK2	tyrosine kinase 2		REACTOME_IMMUNE_SYSTEM								
6672	SP100	SP100 nuclear antigen		REACTOME_IMMUNE_SYSTEM								
51135	IRAK4	interleukin-1 receptor-associated kinase 4		REACTOME_IMMUNE_SYSTEM								
25970	SH2B1	SH2B adaptor protein 1		REACTOME_IMMUNE_SYSTEM								
4205	MEF2A	myocyte enhancer factor 2A		REACTOME_IMMUNE_SYSTEM								
1386	ATF2	activating transcription factor 2		REACTOME_IMMUNE_SYSTEM								
9641	IKBKE	inhibitor of kappa light polypeptide gene enhancer in B-cells, kinase epsilon		REACTOME_IMMUNE_SYSTEM								
51295	ECSIT	ECSIT homolog (Drosophila)		REACTOME_IMMUNE_SYSTEM								
59307	SIGIRR	single immunoglobulin and toll-interleukin 1 receptor (TIR) domain		REACTOME_IMMUNE_SYSTEM								
81793	TLR10	toll-like receptor 10		REACTOME_IMMUNE_SYSTEM								
10695	CNPY3	canopy 3 homolog (zebrafish)		REACTOME_IMMUNE_SYSTEM								
6300	MAPK12	mitogen-activated protein kinase 12		REACTOME_IMMUNE_SYSTEM								
841	CASP8	caspase 8, apoptosis-related cysteine peptidase		REACTOME_IMMUNE_SYSTEM								
10010	TANK	TRAF family member-associated NFKB activator		REACTOME_IMMUNE_SYSTEM								
8887	TAX1BP1	Tax1 (human T-cell leukemia virus type I) binding protein 1		REACTOME_IMMUNE_SYSTEM								
7514	XPO1	exportin 1 (CRM1 homolog, yeast)				REACTOME_CELL_CYCLE	REACTOME_CELL_CYCLE_MITOTIC	REACTOME_METABOLISM_OF_RNA	HALLMARK_E2F_TARGETS	HALLMARK_MYC_TARGETS_V1	REACTOME_METABOLISM_OF_MRNA	
5425	POLD2	polymerase (DNA directed), delta 2, regulatory subunit 50kDa				REACTOME_CELL_CYCLE	REACTOME_CELL_CYCLE_MITOTIC		HALLMARK_E2F_TARGETS	HALLMARK_MYC_TARGETS_V1		
4999	ORC2	origin recognition complex, subunit 2				REACTOME_CELL_CYCLE	REACTOME_CELL_CYCLE_MITOTIC		HALLMARK_E2F_TARGETS	HALLMARK_MYC_TARGETS_V1		
8243	SMC1A	structural maintenance of chromosomes 1A				REACTOME_CELL_CYCLE	REACTOME_CELL_CYCLE_MITOTIC		HALLMARK_E2F_TARGETS			HALLMARK_MITOTIC_SPINDLE
4172	MCM3	minichromosome maintenance complex component 3				REACTOME_CELL_CYCLE	REACTOME_CELL_CYCLE_MITOTIC		HALLMARK_E2F_TARGETS			
10274	STAG1	stromal antigen 1				REACTOME_CELL_CYCLE	REACTOME_CELL_CYCLE_MITOTIC		HALLMARK_E2F_TARGETS			
9212	AURKB	aurora kinase B				REACTOME_CELL_CYCLE	REACTOME_CELL_CYCLE_MITOTIC		HALLMARK_E2F_TARGETS			
994	CDC25B	cell division cycle 25 homolog B (S. pombe)				REACTOME_CELL_CYCLE	REACTOME_CELL_CYCLE_MITOTIC		HALLMARK_E2F_TARGETS			
3978	LIG1	ligase I, DNA, ATP-dependent				REACTOME_CELL_CYCLE	REACTOME_CELL_CYCLE_MITOTIC		HALLMARK_E2F_TARGETS			
5424	POLD1	polymerase (DNA directed), delta 1, catalytic subunit 125kDa				REACTOME_CELL_CYCLE	REACTOME_CELL_CYCLE_MITOTIC		HALLMARK_E2F_TARGETS			
701	BUB1B	budding uninhibited by benzimidazoles 1 homolog beta (yeast)				REACTOME_CELL_CYCLE	REACTOME_CELL_CYCLE_MITOTIC		HALLMARK_E2F_TARGETS			
7465	WEE1	WEE1 homolog (S. pombe)				REACTOME_CELL_CYCLE	REACTOME_CELL_CYCLE_MITOTIC		HALLMARK_E2F_TARGETS			
9735	KNTC1	kinetochore associated 1				REACTOME_CELL_CYCLE	REACTOME_CELL_CYCLE_MITOTIC					HALLMARK_MITOTIC_SPINDLE
11064	CNTRL	centriolin				REACTOME_CELL_CYCLE	REACTOME_CELL_CYCLE_MITOTIC					HALLMARK_MITOTIC_SPINDLE
5116	PCNT	pericentrin				REACTOME_CELL_CYCLE	REACTOME_CELL_CYCLE_MITOTIC					HALLMARK_MITOTIC_SPINDLE
11190	CEP250	centrosomal protein 250kDa				REACTOME_CELL_CYCLE	REACTOME_CELL_CYCLE_MITOTIC					HALLMARK_MITOTIC_SPINDLE
55125	CEP192	centrosomal protein 192kDa				REACTOME_CELL_CYCLE	REACTOME_CELL_CYCLE_MITOTIC					HALLMARK_MITOTIC_SPINDLE
55755	CDK5RAP2	CDK5 regulatory subunit associated protein 2				REACTOME_CELL_CYCLE	REACTOME_CELL_CYCLE_MITOTIC					HALLMARK_MITOTIC_SPINDLE
7840	ALMS1	Alstrom syndrome 1				REACTOME_CELL_CYCLE	REACTOME_CELL_CYCLE_MITOTIC					HALLMARK_MITOTIC_SPINDLE
10844	TUBGCP2	tubulin, gamma complex associated protein 2				REACTOME_CELL_CYCLE	REACTOME_CELL_CYCLE_MITOTIC					HALLMARK_MITOTIC_SPINDLE
114791	TUBGCP5	tubulin, gamma complex associated protein 5				REACTOME_CELL_CYCLE	REACTOME_CELL_CYCLE_MITOTIC					HALLMARK_MITOTIC_SPINDLE
85378	TUBGCP6	tubulin, gamma complex associated protein 6				REACTOME_CELL_CYCLE	REACTOME_CELL_CYCLE_MITOTIC					HALLMARK_MITOTIC_SPINDLE
5985	RFC5	replication factor C (activator 1) 5, 36.5kDa				REACTOME_CELL_CYCLE	REACTOME_CELL_CYCLE_MITOTIC					
5905	RANGAP1	Ran GTPase activating protein 1				REACTOME_CELL_CYCLE	REACTOME_CELL_CYCLE_MITOTIC					
5422	POLA1	polymerase (DNA directed), alpha 1, catalytic subunit				REACTOME_CELL_CYCLE	REACTOME_CELL_CYCLE_MITOTIC					
11130	ZWINT	ZW10 interactor				REACTOME_CELL_CYCLE	REACTOME_CELL_CYCLE_MITOTIC					
84515	MCM8	minichromosome maintenance complex component 8				REACTOME_CELL_CYCLE	REACTOME_CELL_CYCLE_MITOTIC					
10735	STAG2	stromal antigen 2				REACTOME_CELL_CYCLE	REACTOME_CELL_CYCLE_MITOTIC					

2491	CENPI	centromere protein I				REACTOME_CELL_CYCLE	REACTOME_CELL_CYCLE_MITOTIC					
25936	NSL1	NSL1, MIND kinetochore complex component, homolog (S. cerevisiae)				REACTOME_CELL_CYCLE	REACTOME_CELL_CYCLE_MITOTIC					
348235	SKA2	spindle and kinetochore associated complex subunit 2				REACTOME_CELL_CYCLE	REACTOME_CELL_CYCLE_MITOTIC					
80152	CENPT	centromere protein T				REACTOME_CELL_CYCLE	REACTOME_CELL_CYCLE_MITOTIC					
64689	GORASP1	golgi reassembly stacking protein 1, 65kDa				REACTOME_CELL_CYCLE	REACTOME_CELL_CYCLE_MITOTIC					
121441	NEDD1	neural precursor cell expressed, developmentally down-regulated 1				REACTOME_CELL_CYCLE	REACTOME_CELL_CYCLE_MITOTIC					
22897	CEP164	centrosomal protein 164kDa				REACTOME_CELL_CYCLE	REACTOME_CELL_CYCLE_MITOTIC					
10142	AKAP9	A kinase (PRKA) anchor protein (yotiao) 9				REACTOME_CELL_CYCLE	REACTOME_CELL_CYCLE_MITOTIC					
8481	OFD1	oral-facial-digital syndrome 1				REACTOME_CELL_CYCLE	REACTOME_CELL_CYCLE_MITOTIC					
8636	SSNA1	Sjogren syndrome nuclear autoantigen 1				REACTOME_CELL_CYCLE	REACTOME_CELL_CYCLE_MITOTIC					
27175	TUBG2	tubulin, gamma 2				REACTOME_CELL_CYCLE	REACTOME_CELL_CYCLE_MITOTIC					
995	CDC25C	cell division cycle 25 homolog C (S. pombe)				REACTOME_CELL_CYCLE	REACTOME_CELL_CYCLE_MITOTIC					
1859	DYRK1A	dual-specificity tyrosine-(Y)-phosphorylation regulated kinase 1A				REACTOME_CELL_CYCLE	REACTOME_CELL_CYCLE_MITOTIC					
672	BRCA1	breast cancer 1, early onset				REACTOME_CELL_CYCLE			HALLMARK_E2F_TARGETS			
5931	RBBP7	retinoblastoma binding protein 7				REACTOME_CELL_CYCLE			HALLMARK_E2F_TARGETS			
4869	NPM1	nucleophosmin (nucleolar phosphoprotein B23, numatrin)				REACTOME_CELL_CYCLE				HALLMARK_MYC_TARGETS_V1		
25777	SUN2	Sad1 and UNC84 domain containing 2				REACTOME_CELL_CYCLE						HALLMARK_MITOTIC_SPINDLE
472	ATM	ataxia telangiectasia mutated				REACTOME_CELL_CYCLE						
5883	RAD9A	RAD9 homolog A (S. pombe)				REACTOME_CELL_CYCLE						
8341	HIST1H2BN	histone cluster 1, H2bn				REACTOME_CELL_CYCLE						
65057	ACD	adrenocortical dysplasia homolog (mouse)				REACTOME_CELL_CYCLE						
55135	WRAP53	WD repeat containing, antisense to TP53				REACTOME_CELL_CYCLE						
10734	STAG3	stromal antigen 3				REACTOME_CELL_CYCLE						
51773	RSF1	remodeling and spacing factor 1				REACTOME_CELL_CYCLE						
10921	RNPS1	RNA binding protein S1, serine-rich domain						REACTOME_METABOLISM_OF_RNA		HALLMARK_MYC_TARGETS_V1	REACTOME_METABOLISM_OF_MRNA	
23293	SMG6	smg-6 homolog, nonsense mediated mRNA decay factor (C. elegans)						REACTOME_METABOLISM_OF_RNA			REACTOME_METABOLISM_OF_MRNA	
65110	UPF3A	UPF3 regulator of nonsense transcripts homolog A (yeast)						REACTOME_METABOLISM_OF_RNA			REACTOME_METABOLISM_OF_MRNA	
167227	DCP2	DCP2 decapping enzyme homolog (S. cerevisiae)						REACTOME_METABOLISM_OF_RNA			REACTOME_METABOLISM_OF_MRNA	
23404	EXOSC2	exosome component 2						REACTOME_METABOLISM_OF_RNA			REACTOME_METABOLISM_OF_MRNA	
23644	EDC4	enhancer of mRNA decapping 4						REACTOME_METABOLISM_OF_RNA			REACTOME_METABOLISM_OF_MRNA	
27257	LSM1	LSM1 homolog, U6 small nuclear RNA associated (S. cerevisiae)						REACTOME_METABOLISM_OF_RNA			REACTOME_METABOLISM_OF_MRNA	
28960	DCPS	decapping enzyme, scavenger						REACTOME_METABOLISM_OF_RNA			REACTOME_METABOLISM_OF_MRNA	
25904	CNOT10	CCR4-NOT transcription complex, subunit 10						REACTOME_METABOLISM_OF_RNA			REACTOME_METABOLISM_OF_MRNA	
79760	GEMIN7	gem (nuclear organelle) associated protein 7						REACTOME_METABOLISM_OF_RNA				
6434	TRA2B	transformer 2 beta homolog (Drosophila)							HALLMARK_E2F_TARGETS	HALLMARK_MYC_TARGETS_V1		
4673	NAP1L1	nucleosome assembly protein 1-like 1							HALLMARK_E2F_TARGETS	HALLMARK_MYC_TARGETS_V1		
7913	DEK	DEK oncogene							HALLMARK_E2F_TARGETS	HALLMARK_MYC_TARGETS_V1		
10051	SMC4	structural maintenance of chromosomes 4							HALLMARK_E2F_TARGETS			HALLMARK_MITOTIC_SPINDLE
3159	HMGA1	high mobility group AT-hook 1							HALLMARK_E2F_TARGETS			
4678	NASP	nuclear autoantigenic sperm protein (histone-binding)							HALLMARK_E2F_TARGETS			
4288	MKI67	antigen identified by monoclonal antibody Ki-67							HALLMARK_E2F_TARGETS			
7112	TMPO	thymopoietin							HALLMARK_E2F_TARGETS			
5591	PRKDC	protein kinase, DNA-activated, catalytic polypeptide							HALLMARK_E2F_TARGETS			
51691	NAA38	N(alpha)-acetyltransferase 38, NatC auxiliary subunit							HALLMARK_E2F_TARGETS			
4292	MLH1	mutL homolog 1, colon cancer, nonpolyposis type 2 (E. coli)							HALLMARK_E2F_TARGETS			
5889	RAD51C	RAD51 homolog C (S. cerevisiae)							HALLMARK_E2F_TARGETS			
9238	TBRG4	transforming growth factor beta regulator 4							HALLMARK_E2F_TARGETS			
81624	DIAPH3	diaphanous homolog 3 (Drosophila)							HALLMARK_E2F_TARGETS			
8914	TIMELESS	timeless homolog (Drosophila)							HALLMARK_E2F_TARGETS			
10615	SPAG5	sperm associated antigen 5							HALLMARK_E2F_TARGETS			
197335	WDR90	WD repeat domain 90							HALLMARK_E2F_TARGETS			
29893	PSMC3IP	PSMC3 interacting protein							HALLMARK_E2F_TARGETS			
29980	DONSON	downstream neighbor of SON							HALLMARK_E2F_TARGETS			
64785	GINS3	GINS complex subunit 3 (Psf3 homolog)							HALLMARK_E2F_TARGETS			
6941	TCF19	transcription factor 19							HALLMARK_E2F_TARGETS			
83461	CDCA3	cell division cycle associated 3							HALLMARK_E2F_TARGETS			
83463	MXD3	MAX dimerization protein 3							HALLMARK_E2F_TARGETS			
9833	MELK	maternal embryonic leucine zipper kinase							HALLMARK_E2F_TARGETS			
9924	PAN2	PAN2 poly(A) specific ribonuclease subunit homolog (S. cerevisiae)							HALLMARK_E2F_TARGETS			
6432	SRSF7	serine/arginine-rich splicing factor 7								HALLMARK_MYC_TARGETS_V1		
3183	HNRNPC	heterogeneous nuclear ribonucleoprotein C (C1/C2)								HALLMARK_MYC_TARGETS_V1		
10236	HNRNPR	heterogeneous nuclear ribonucleoprotein R								HALLMARK_MYC_TARGETS_V1		
3181	HNRNPA2B1	heterogeneous nuclear ribonucleoprotein A2/B1								HALLMARK_MYC_TARGETS_V1		
6599	SMARCC1	NF related, matrix associated, actin dependent regulator of chromatin, subfamily c, member 1								HALLMARK_MYC_TARGETS_V1		
5478	PPIA	peptidylprolyl isomerase A (cyclophilin A)								HALLMARK_MYC_TARGETS_V1		
3615	IMPDH2	IMP (inosine 5'-monophosphate) dehydrogenase 2								HALLMARK_MYC_TARGETS_V1		
790	CAD	carbamoyl-phosphate synthetase 2, aspartate transcarbamylase, and dihydroorotase								HALLMARK_MYC_TARGETS_V1		
10935	PRDX3	peroxiredoxin 3								HALLMARK_MYC_TARGETS_V1		
4706	NDUFAB1	NADH dehydrogenase (ubiquinone) 1, alpha/beta subcomplex, 1, 8kDa								HALLMARK_MYC_TARGETS_V1		
7555	CNBP	CCHC-type zinc finger, nucleic acid binding protein								HALLMARK_MYC_TARGETS_V1		
52	ACP1	acid phosphatase 1, soluble								HALLMARK_MYC_TARGETS_V1		
10987	COPS5	COP9 constitutive photomorphogenic homolog subunit 5 (Arabidopsis)								HALLMARK_MYC_TARGETS_V1		
7965	AIMP2	aminoacyl tRNA synthetase complex-interacting multifunctional protein 2								HALLMARK_MYC_TARGETS_V1		
1665	DHX15	DEAH (Asp-Glu-Ala-His) box polypeptide 15								HALLMARK_MYC_TARGETS_V1		
51491	NOP16	NOP16 nucleolar protein homolog (yeast)								HALLMARK_MYC_TARGETS_V1		
23435	TARDBP	TAR DNA binding protein								HALLMARK_MYC_TARGETS_V1		
5496	PPM1G	protein phosphatase, Mg2+/Mn2+ dependent, 1G								HALLMARK_MYC_TARGETS_V1		
6732	SRPK1	SRSF protein kinase 1								HALLMARK_MYC_TARGETS_V1		
8761	PABPC4	poly(A) binding protein, cytoplasmic 4 (inducible form)								HALLMARK_MYC_TARGETS_V1		
9055	PRC1	protein regulator of cytokinesis 1										HALLMARK_MITOTIC_SPINDLE
29901	SAC3D1	SAC3 domain containing 1										HALLMARK_MITOTIC_SPINDLE
2316	FLNA	filamin A, alpha										HALLMARK_MITOTIC_SPINDLE
89941	RHOT2	ras homolog gene family, member T2										HALLMARK_MITOTIC_SPINDLE
393	ARHGAP4	Rho GTPase activating protein 4										HALLMARK_MITOTIC_SPINDLE
9181	ARHGEF2	Rho/Rac guanine nucleotide exchange factor (GEF) 2										HALLMARK_MITOTIC_SPINDLE
64857	PLEKHG2	pleckstrin homology domain containing, family G (with RhoGef domain) member 2										HALLMARK_MITOTIC_SPINDLE
80119	PIF1	PIF1 5'-to-3' DNA helicase homolog (S. cerevisiae)										HALLMARK_MITOTIC_SPINDLE
8936	WASF1	WAS protein family, member 1										HALLMARK_MITOTIC_SPINDLE
1739	DLG1	discs, large homolog 1 (Drosophila)										HALLMARK_MITOTIC_SPINDLE
4739	NEDD9	neural precursor cell expressed, developmentally down-regulated 9										HALLMARK_MITOTIC_SPINDLE
9344	TAOK2	TAO kinase 2										HALLMARK_MITOTIC_SPINDLE
5922	RASA2	RAS p21 protein activator 2										HALLMARK_MITOTIC_SPINDLE
2037	EPB41L2	erythrocyte membrane protein band 4.1-like 2										HALLMARK_MITOTIC_SPINDLE
51199	NIN	ninein (GSK3B interacting protein)										HALLMARK_MITOTIC_SPINDLE
116840	CNTROB	centrobin, centrosomal BRCA2 interacting protein										HALLMARK_MITOTIC_SPINDLE
201176	ARHGAP27	Rho GTPase activating protein 27										HALLMARK_MITOTIC_SPINDLE
22930	RAB3GAP1	RAB3 GTPase activating protein subunit 1 (catalytic)										HALLMARK_MITOTIC_SPINDLE
23637	RABGAP1	RAB GTPase activating protein 1										HALLMARK_MITOTIC_SPINDLE
51174	TUBD1	tubulin, delta 1										HALLMARK_MITOTIC_SPINDLE
54443	ANLN	anillin, actin binding protein										HALLMARK_MITOTIC_SPINDLE
8470	SORBS2	sorbin and SH3 domain containing 2										HALLMARK_MITOTIC_SPINDLE
4799	NFX1	nuclear transcription factor, X-box binding 1										
10914	PAPOLA	poly(A) polymerase alpha										
29894	CPSF1	cleavage and polyadenylation specific factor 1, 160kDa										
53981	CPSF2	cleavage and polyadenylation specific factor 2, 100kDa										
9785	DHX38	DEAH (Asp-Glu-Ala-His) box polypeptide 38										
9295	SRSF11	serine/arginine-rich splicing factor 11										
79869	CPSF7	cleavage and polyadenylation specific factor 7, 59kDa										
2521	FUS	fused in sarcoma										
10594	PRPF8	PRP8 pre-mRNA processing factor 8 homolog (S. cerevisiae)										
3190	HNRNPK	heterogeneous nuclear ribonucleoprotein K										
10181	RBM5	RNA binding motif protein 5										
3187	HNRNPH1	heterogeneous nuclear ribonucleoprotein H1 (H)										
55749	CCAR1	cell division cycle and apoptosis regulator 1										
5725	PTBP1	polypyrimidine tract binding protein 1										
56339	METTL3	methyltransferase like 3										
2071	ERCC3	lementing rodent repair deficiency, complementation group 3 (xeroderma pigmentosum group B complementing)										
2968	GTF2H4	general transcription factor IIH, polypeptide 4, 52kDa										
8732	RNGTT	RNA guanylyltransferase and 5'-phosphatase										
104	ADARB1	adenosine deaminase, RNA-specific, B1										
4691	NCL	nucleolin										
1841	DTYMK	deoxythymidylate kinase (thymidylate kinase)										
2130	EWSR1	Ewing sarcoma breakpoint region 1										
602	BCL3	B-cell CLL/lymphoma 3										
4957	ODF2	outer dense fiber of sperm tails 2										
10772	SRSF10	serine/arginine-rich splicing factor 10										
9156	EXO1	exonuclease 1										
387893	SETD8	SET domain containing (lysine methyltransferase) 8										
7468	WHSC1	Wolf-Hirschhorn syndrome candidate 1										
9994	CASP8AP2	caspase 8 associated protein 2										
1756	DMD	dystrophin										
10024	TROAP	trophinin associated protein (tastin)										
10721	POLQ	polymerase (DNA directed), theta										
1112	FOXN3	forkhead box N3										
22823	MTF2	metal response element binding transcription factor 2										
30000	TNPO2	transportin 2										
6760	SS18	synovial sarcoma translocation, chromosome 18										
8899	PRPF4B	PRP4 pre-mRNA processing factor 4 homolog B (yeast)										
6917	TCEA1	transcription elongation factor A (SII), 1										
1643	DDB2	damage-specific DNA binding protein 2, 48kDa										
2067	ERCC1	complementing rodent repair deficiency, complementation group 1 (includes overlapping antisense sequence)										
5893	RAD52	RAD52 homolog (S. cerevisiae)										
5980	REV3L	REV3-like, catalytic subunit of DNA polymerase zeta (yeast)										
55120	FANCL	Fanconi anemia, complementation group L										
2177	FANCD2	Fanconi anemia, complementation group D2										
2178	FANCE	Fanconi anemia, complementation group E										
2189	FANCG	Fanconi anemia, complementation group G										
2175	FANCA	Fanconi anemia, complementation group A										
2187	FANCB	Fanconi anemia, complementation group B										
4913	NTHL1	nth endonuclease III-like 1 (E. coli)										
4968	OGG1	8-oxoguanine DNA glycosylase										
4595	MUTYH	mutY homolog (E. coli)										
7507	XPA	xeroderma pigmentosum, complementation group A										
51160	VPS28	vacuolar protein sorting 28 homolog (S. cerevisiae)										
51606	ATP6V1H	ATPase, H+ transporting, lysosomal 50/57kDa, V1 subunit H										
905	CCNT2	cyclin T2										
6875	TAF4B	TAF4b RNA polymerase II, TATA box binding protein (TBP)-associated factor, 105kDa										
30833	NT5C	5', 3'-nucleotidase, cytosolic										
30834	ZNRD1	zinc ribbon domain containing 1										
124583	CANT1	calcium activated nucleotidase 1										
50808	AK3	adenylate kinase 3										
3059	HCLS1	hematopoietic cell-specific Lyn substrate 1										
2987	GUK1	guanylate kinase 1										
1716	DGUOK	deoxyguanosine kinase										
9013	TAF1C	TATA box binding protein (TBP)-associated factor, RNA polymerase I, C, 110kDa										
10302	SNAPC5	small nuclear RNA activating complex, polypeptide 5, 19kDa										
27343	POLL	polymerase (DNA directed), lambda										
6895	TARBP2	TAR (HIV-1) RNA binding protein 2										
6604	SMARCD3	NF related, matrix associated, actin dependent regulator of chromatin, subfamily d, member 3										
8202	NCOA3	nuclear receptor coactivator 3										
79718	TBL1XR1	transducin (beta)-like 1 X-linked receptor 1										
9968	MED12	mediator complex subunit 12										
81857	MED25	mediator complex subunit 25										
9439	MED23	mediator complex subunit 23										
3073	HEXA	hexosaminidase A (alpha polypeptide)										
6609	SMPD1	sphingomyelin phosphodiesterase 1, acid lysosomal										
2629	GBA	glucosidase, beta, acid										
8898	MTMR2	myotubularin related protein 2										
8776	MTMR1	myotubularin related protein 1										
57153	SLC44A2	solute carrier family 44, member 2										
10554	AGPAT1	cylglycerol-3-phosphate O-acyltransferase 1 (lysophosphatidic acid acyltransferase, alpha)										
254531	LPCAT4	lysophosphatidylcholine acyltransferase 4										
1120	CHKB	choline kinase beta										
55500	ETNK1	ethanolamine kinase 1										
79143	MBOAT7	membrane bound O-acyltransferase domain containing 7										
81490	PTDSS2	phosphatidylserine synthase 2										
9663	LPIN2	lipin 2										
200576	PIKFYVE	phosphoinositide kinase, FYVE finger containing										
85465	EPT1	ethanolaminephosphotransferase 1 (CDP-ethanolamine-specific)										
23646	PLD3	phospholipase D family, member 3										
56261	GPCPD1	glycerophosphocholine phosphodiesterase GDE1 homolog (S. cerevisiae)										
55627	SMPD4	sphingomyelin phosphodiesterase 4, neutral membrane (neutral sphingomyelinase-3)										
22908	SACM1L	SAC1 suppressor of actin mutations 1-like (yeast)										
9517	SPTLC2	serine palmitoyltransferase, long chain base subunit 2										
30	ACAA1	acetyl-CoA acyltransferase 1										
37	ACADVL	acyl-CoA dehydrogenase, very long chain										
211	ALAS1	aminolevulinate, delta-, synthase 1										
51	ACOX1	acyl-CoA oxidase 1, palmitoyl										
2224	FDPS	farnesyl diphosphate synthase										
31	ACACA	acetyl-CoA carboxylase alpha										
2181	ACSL3	acyl-CoA synthetase long-chain family member 3										
47	ACLY	ATP citrate lyase										
3156	HMGCR	3-hydroxy-3-methylglutaryl-CoA reductase										
134429	STARD4	StAR-related lipid transfer (START) domain containing 4										
6715	SRD5A1	5-alpha-reductase, alpha polypeptide 1 (3-oxo-5 alpha-steroid delta 4-dehydrogenase alpha 1)										
4800	NFYA	nuclear transcription factor Y, alpha										
8310	ACOX3	acyl-CoA oxidase 3, pristanoyl										
949	SCARB1	scavenger receptor class B, member 1										
64834	ELOVL1	ELOVL fatty acid elongase 1										
5019	OXCT1	3-oxoacid CoA transferase 1										
10998	SLC27A5	solute carrier family 27 (fatty acid transporter), member 5										
4597	MVD	mevalonate (diphospho) decarboxylase										
51478	HSD17B7	hydroxysteroid (17-beta) dehydrogenase 7										
4047	LSS	lanosterol synthase (2,3-oxidosqualene-lanosterol cyclase)										
51302	CYP39A1	cytochrome P450, family 39, subfamily A, polypeptide 1										
120227	CYP2R1	cytochrome P450, family 2, subfamily R, polypeptide 1										
83737	ITCH	itchy E3 ubiquitin protein ligase homolog (mouse)										
23295	MGRN1	mahogunin, ring finger 1										
26091	HERC4	hect domain and RLD 4										
51366	UBR5	ubiquitin protein ligase E3 component n-recognin 5										
57448	BIRC6	baculoviral IAP repeat containing 6										
8925	HERC1	nologous to the E6-AP (UBE3A) carboxyl terminus) domain and RCC1 (CHC1)-like domain (RLD) 1										
2648	KAT2A	K(lysine) acetyltransferase 2A										
8013	NR4A3	nuclear receptor subfamily 4, group A, member 3										
4087	SMAD2	SMAD family member 2										
6257	RXRB	retinoid X receptor, beta										
126382	NR2C2AP	nuclear receptor 2C2-associated protein										
10781	ZNF266	zinc finger protein 266										
10793	ZNF273	zinc finger protein 273										
121274	ZNF641	zinc finger protein 641										
147923	ZNF420	zinc finger protein 420										
148268	ZNF570	zinc finger protein 570										
162967	ZNF320	zinc finger protein 320										
169841	ZNF169	zinc finger protein 169										
22869	ZNF510	zinc finger protein 510										
284443	ZNF493	zinc finger protein 493										
51276	ZNF571	zinc finger protein 571										
55422	ZNF331	zinc finger protein 331										
55786	ZNF415	zinc finger protein 415										
57335	ZNF286A	zinc finger protein 286A										
57693	ZNF317	zinc finger protein 317										
57711	ZNF529	zinc finger protein 529										
7582	ZNF33B	zinc finger protein 33B										
7587	ZNF37A	zinc finger protein 37A										
7596	ZNF45	zinc finger protein 45										
7695	ZNF136	zinc finger protein 136										
7697	ZNF138	zinc finger protein 138										

7743	ZNF189	zinc finger protein 189
7753	ZNF202	zinc finger protein 202
79027	ZNF655	zinc finger protein 655
90233	ZNF551	zinc finger protein 551
92285	ZNF585B	zinc finger protein 585B
93134	ZNF561	zinc finger protein 561
94039	ZNF101	zinc finger protein 101
9534	ZNF254	zinc finger protein 254
5394	EXOSC10	exosome component 10
10200	MPHOSPH6	M-phase phosphoprotein 6
10438	C1D	C1D nuclear receptor corepressor
23019	CNOT1	CCR4-NOT transcription complex, subunit 1
6499	SKIV2L	superkiller viralicidic activity 2-like (S. cerevisiae)
84186	ZCCHC7	zinc finger, CCHC domain containing 7
4831	NME2	non-metastatic cells 2, protein (NM23B) expressed in
5213	PFKM	phosphofructokinase, muscle
8295	TRRAP	transformation/transcription domain-associated protein
9913	SUPT7L	suppressor of Ty 7 (S. cerevisiae)-like
11235	PDCD10	programmed cell death 10
83879	CDCA7	cell division cycle associated 7
92140	MTDH	metadherin
11184	MAP4K1	mitogen-activated protein kinase kinase kinase kinase 1
933	CD22	CD22 molecule
7410	VAV2	vav 2 guanine nucleotide exchange factor
971	CD72	CD72 molecule
1796	DOK1	docking protein 1, 62kDa (downstream of tyrosine kinase 1)
10622	POLR3G	polymerase (RNA) III (DNA directed) polypeptide G (32kD)
171568	POLR3H	polymerase (RNA) III (DNA directed) polypeptide H (22.9kD)
56953	NT5M	5',3'-nucleotidase, mitochondrial
957	ENTPD5	ectonucleoside triphosphate diphosphohydrolase 5
548644	POLR2J3	polymerase (RNA) II (DNA directed) polypeptide J3
10201	NME6	non-metastatic cells 6, protein expressed in (nucleoside-diphosphate kinase)
955	ENTPD6	ectonucleoside triphosphate diphosphohydrolase 6 (putative)
56474	CTPS2	CTP synthase II
7453	WARS	tryptophanyl-tRNA synthetase
7163	TPD52	tumor protein D52
961	CD47	CD47 molecule
3594	IL12RB1	interleukin 12 receptor, beta 1
9466	IL27RA	interleukin 27 receptor, alpha
11146	GLMN	glomulin, FKBP associated protein
4528	MTIF2	mitochondrial translational initiation factor 2
55215	FANCI	Fanconi anemia, complementation group I
80010	RMI1	RMI1, RecQ mediated genome instability 1, homolog (S. cerevisiae)
10312	TCIRG1	T-cell, immune regulator 1, ATPase, H+ transporting, lysosomal V0 subunit A3
53	ACP2	acid phosphatase 2, lysosomal
2990	GUSB	glucuronidase, beta
23659	PLA2G15	phospholipase A2, group XV
3920	LAMP2	lysosomal-associated membrane protein 2
4669	NAGLU	N-acetylglucosaminidase, alpha
4125	MAN2B1	mannosidase, alpha, class 2B, member 1
1201	CLN3	ceroid-lipofuscinosis, neuronal 3
26088	GGA1	golgi-associated, gamma adaptin ear containing, ARF binding protein 1
84572	GNPTG	N-acetylglucosamine-1-phosphate transferase, gamma subunit
9179	AP4M1	adaptor-related protein complex 4, mu 1 subunit
9476	NAPSA	napsin A aspartic peptidase
593	BCKDHA	branched chain keto acid dehydrogenase E1, alpha polypeptide
4713	NDUFB7	NADH dehydrogenase (ubiquinone) 1 beta subcomplex, 7, 18kDa
10449	ACAA2	acetyl-CoA acyltransferase 2
1743	DLST	dihydrolipoamide S-succinyltransferase (E2 component of 2-oxo-glutarate complex)
23479	ISCU	iron-sulfur cluster scaffold homolog (E. coli)
3420	IDH3B	isocitrate dehydrogenase 3 (NAD+) beta
23530	NNT	nicotinamide nucleotide transhydrogenase
4696	NDUFA3	NADH dehydrogenase (ubiquinone) 1 alpha subcomplex, 3, 9kDa
4708	NDUFB2	NADH dehydrogenase (ubiquinone) 1 beta subcomplex, 2, 8kDa
10632	ATP5L	ATP synthase, H+ transporting, mitochondrial Fo complex, subunit G
9551	ATP5J2	ATP synthase, H+ transporting, mitochondrial Fo complex, subunit F2
5018	OXA1L	oxidase (cytochrome c) assembly 1-like
4835	NQO2	NAD(P)H dehydrogenase, quinone 2
9617	MTRF1	mitochondrial translational release factor 1
5052	PRDX1	peroxiredoxin 1
54982	CLN6	ceroid-lipofuscinosis, neuronal 6, late infantile, variant
6648	SOD2	superoxide dismutase 2, mitochondrial
373156	GSTK1	glutathione S-transferase kappa 1
5190	PEX6	peroxisomal biogenesis factor 6
5828	PEX2	peroxisomal biogenesis factor 2
3416	IDE	insulin-degrading enzyme
60386	SLC25A19	solute carrier family 25 (mitochondrial thiamine pyrophosphate carrier), member 19
9270	ITGB1BP1	integrin beta 1 binding protein 1
5859	QARS	glutaminyl-tRNA synthetase
57176	VARS2	valyl-tRNA synthetase 2, mitochondrial (putative)
23438	HARS2	histidyl-tRNA synthetase 2, mitochondrial (putative)
57038	RARS2	arginyl-tRNA synthetase 2, mitochondrial
57505	AARS2	alanyl-tRNA synthetase 2, mitochondrial (putative)
79587	CARS2	cysteinyl-tRNA synthetase 2, mitochondrial (putative)
79731	NARS2	asparaginyl-tRNA synthetase 2, mitochondrial (putative)
80222	TARS2	threonyl-tRNA synthetase 2, mitochondrial (putative)
5169	ENPP3	ectonucleotide pyrophosphatase/phosphodiesterase 3
29085	PHPT1	phosphohistidine phosphatase 1
80308	FLAD1	FAD1 flavin adenine dinucleotide synthetase homolog (S. cerevisiae)
4122	MAN2A2	mannosidase, alpha, class 2A, member 2
271	AMPD2	adenosine monophosphate deaminase 2
5151	PDE8A	phosphodiesterase 8A
58497	PRUNE	prune homolog (Drosophila)
2542	SLC37A4	solute carrier family 37 (glucose-6-phosphate transporter), member 4
6533	SLC6A6	solute carrier family 6 (neurotransmitter transporter, taurine), member 6
5211	PFKL	phosphofructokinase, liver
2805	GOT1	glutamic-oxaloacetic transaminase 1, soluble (aspartate aminotransferase 1)
440	ASNS	asparagine synthetase (glutamine-hydrolyzing)
441024	MTHFD2L	methylenetetrahydrofolate dehydrogenase (NADP+ dependent) 2-like
3145	HMBS	hydroxymethylbilane synthase
6472	SHMT2	serine hydroxymethyltransferase 2 (mitochondrial)
120	ADD3	adducin 3 (gamma)
11151	CORO1A	coronin, actin binding protein, 1A
2997	GYS1	glycogen synthase 1 (muscle)
673	BRAF	v-raf murine sarcoma viral oncogene homolog B1
2319	FLOT2	flotillin 2
10211	FLOT1	flotillin 1
23265	EXOC7	exocyst complex component 7
5106	PCK2	phosphoenolpyruvate carboxykinase 2 (mitochondrial)
5261	PHKG2	phosphorylase kinase, gamma 2 (testis)
9322	TRIP10	thyroid hormone receptor interactor 10
122809	SOCS4	suppressor of cytokine signaling 4
10919	EHMT2	euchromatic histone-lysine N-methyltransferase 2
3020	H3F3A	H3 histone, family 3A
9015	TAF1A	TATA box binding protein (TBP)-associated factor, RNA polymerase I, A, 48kDa
2976	GTF3C2	general transcription factor IIIC, polypeptide 2, beta 110kDa
23443	SLC35A3	lute carrier family 35 (UDP-N-acetylglucosamine (UDP-GlcNAc) transporter), member A3
6574	SLC20A1	solute carrier family 20 (phosphate transporter), member 1
6560	SLC12A4	solute carrier family 12 (potassium/chloride transporters), member 4
8501	SLC43A1	solute carrier family 43, member 1
92745	SLC38A5	solute carrier family 38, member 5
121260	SLC15A4	solute carrier family 15, member 4
51296	SLC15A3	solute carrier family 15, member 3
6522	SLC4A2	e carrier family 4, anion exchanger, member 2 (erythrocyte membrane protein band 3-like 1)
6575	SLC20A2	solute carrier family 20 (phosphate transporter), member 2
84679	SLC9A7	solute carrier family 9 (sodium/hydrogen exchanger), member 7
9497	SLC4A7	solute carrier family 4, sodium bicarbonate cotransporter, member 7
9990	SLC12A6	solute carrier family 12 (potassium/chloride transporters), member 6
2030	SLC29A1	solute carrier family 29 (nucleoside transporters), member 1
29988	SLC2A8	solute carrier family 2 (facilitated glucose transporter), member 8
84912	SLC35B4	solute carrier family 35, member B4
254428	SLC41A1	solute carrier family 41, member 1
7264	TSTA3	tissue specific transplantation antigen P35B
7360	UGP2	UDP-glucose pyrophosphorylase 2
2582	GALE	UDP-galactose-4-epimerase
25796	PGLS	6-phosphogluconolactonase
4731	NDUFV3	NADH dehydrogenase (ubiquinone) flavoprotein 3, 10kDa
4282	MIF	macrophage migration inhibitory factor (glycosylation-inhibiting factor)
55139	ANKZF1	ankyrin repeat and zinc finger domain containing 1
10380	BPNT1	3'(2'), 5'-bisphosphate nucleotidase 1
10327	AKR1A1	aldo-keto reductase family 1, member A1 (aldehyde reductase)
10456	HAX1	HCLS1 associated protein X-1
156	ADRBK1	adrenergic, beta, receptor kinase 1
23370	ARHGEF18	Rho/Rac guanine nucleotide exchange factor (GEF) 18
221472	FGD2	FYVE, RhoGEF and PH domain containing 2
9138	ARHGEF1	Rho guanine nucleotide exchange factor (GEF) 1
55247	NEIL3	nei endonuclease VIII-like 3 (E. coli)
79661	NEIL1	nei endonuclease VIII-like 1 (E. coli)
2592	GALT	galactose-1-phosphate uridylyltransferase
51005	AMDHD2	amidohydrolase domain containing 2
80896	NPL	N-acetylneuraminate pyruvate lyase (dihydrodipicolinate synthase)
5871	MAP4K2	mitogen-activated protein kinase kinase kinase kinase 2
1616	DAXX	death-domain associated protein
4209	MEF2D	myocyte enhancer factor 2D
4293	MAP3K9	mitogen-activated protein kinase kinase kinase 9
23368	PPP1R13B	protein phosphatase 1, regulatory subunit 13B
7818	DAP3	death associated protein 3
1606	DGKA	diacylglycerol kinase, alpha 80kDa
8681	JMJD7-PLA2G4B	JMJD7-PLA2G4B readthrough
8525	DGKZ	diacylglycerol kinase, zeta
10434	LYPLA1	lysophospholipase I
11313	LYPLA2	lysophospholipase II
10897	YIF1A	Yip1 interacting factor homolog A (S. cerevisiae)
10954	PDIA5	protein disulfide isomerase family A, member 5
63967	CLSPN	claspin
2801	GOLGA2	golgin A2
5189	PEX1	peroxisomal biogenesis factor 1
55670	PEX26	peroxisomal biogenesis factor 26
9409	PEX16	peroxisomal biogenesis factor 16
4358	MPV17	MpV17 mitochondrial inner membrane protein
2260	FGFR1	fibroblast growth factor receptor 1
11052	CPSF6	cleavage and polyadenylation specific factor 6, 68kDa
1523	CUX1	cut-like homeobox 1
7750	ZMYM2	zinc finger, MYM-type 2
22985	ACIN1	apoptotic chromatin condensation inducer 1
10084	PQBP1	polyglutamine binding protein 1
10713	USP39	ubiquitin specific peptidase 39
10915	TCERG1	transcription elongation regulator 1
11325	DDX42	DEAD (Asp-Glu-Ala-Asp) box polypeptide 42
22827	PUF60	poly-U binding splicing factor 60KDa
29896	TRA2A	transformer 2 alpha homolog (Drosophila)
55119	PRPF38B	PRP38 pre-mRNA processing factor 38 (yeast) domain containing B
10284	SAP18	Sin3A-associated protein, 18kDa
6670	SP3	Sp3 transcription factor
54984	PINX1	PIN2/TERF1 interacting, telomerase inhibitor 1
7316	UBC	ubiquitin C
11194	ABCB8	ATP-binding cassette, sub-family B (MDR/TAP), member 8
11176	BAZ2A	bromodomain adjacent to zinc finger domain, 2A
128338	DRAM2	DNA-damage regulated autophagy modulator 2
130814	PQLC3	PQ loop repeat containing 3
51805	COQ3	coenzyme Q3 homolog, methyltransferase (S. cerevisiae)
55037	PTCD3	Pentatricopeptide repeat domain 3
84173	ELMOD3	ELMO/CED-12 domain containing 3
2956	MSH6	mutS homolog 6 (E. coli)
29904	EEF2K	eukaryotic elongation factor-2 kinase
3692	EIF6	eukaryotic translation initiation factor 6
9380	GRHPR	glyoxylate reductase/hydroxypyruvate reductase
125061	AFMID	arylformamidase
4522	MTHFD1	dehydrogenase (NADP+ dependent) 1, methenyltetrahydrofolate cyclohydrolase, formyltetrahydrofolate synthetase
132158	GLYCTK	glycerate kinase
25989	ULK3	unc-51-like kinase 3 (C. elegans)
4049	LTA	lymphotoxin alpha (TNF superfamily, member 1)
9869	SETDB1	SET domain, bifurcated 1
8985	PLOD3	procollagen-lysine, 2-oxoglutarate 5-dioxygenase 3
29072	SETD2	SET domain containing 2
83852	SETDB2	SET domain, bifurcated 2
84444	DOT1L	DOT1-like, histone H3 methyltransferase (S. cerevisiae)
54331	GNG2	guanine nucleotide binding protein (G protein), gamma 2
5331	PLCB3	phospholipase C, beta 3 (phosphatidylinositol-specific)
54434	SSH1	slingshot homolog 1 (Drosophila)
5996	RGS1	regulator of G-protein signaling 1
6303	SAT1	spermidine/spermine N1-acetyltransferase 1
10906	TRAFD1	TRAF-type zinc finger domain containing 1
9618	TRAF4	TNF receptor-associated factor 4
50619	DEF6	differentially expressed in FDCP 6 homolog (mouse)
51181	DCXR	dicarbonyl/L-xylulose reductase
23654	PLXNB2	plexin B2
23378	RRP8	ribosomal RNA processing 8, methyltransferase, homolog (yeast)
3094	HINT1	histidine triad nucleotide binding protein 1
4033	LRMP	lymphoid-restricted membrane protein
49856	WRAP73	WD repeat containing, antisense to TP73
51002	TPRKB	TP53RK binding protein
51768	TM7SF3	transmembrane 7 superfamily member 3
8729	GBF1	golgi brefeldin A resistant guanine nucleotide exchange factor 1
1315	COPB1	coatomer protein complex, subunit beta 1
6810	STX4	syntaxin 4
8301	PICALM	phosphatidylinositol binding clathrin assembly protein
25977	NECAP1	NECAP endocytosis associated 1
27131	SNX5	sorting nexin 5
2664	GDI1	GDP dissociation inhibitor 1
2665	GDI2	GDP dissociation inhibitor 2
4796	TONSL	tonsoku-like, DNA repair protein
538	ATP7A	ATPase, Cu++ transporting, alpha polypeptide
3163	HMOX2	heme oxygenase (decycling) 2
28982	FLVCR1	feline leukemia virus subgroup C cellular receptor 1
10347	ABCA7	ATP-binding cassette, sub-family A (ABC1), member 7
2562	GABRB3	gamma-aminobutyric acid (GABA) A receptor, beta 3
9101	USP8	ubiquitin specific peptidase 8
10094	ARPC3	actin related protein 2/3 complex, subunit 3, 21kDa
4839	NOP2	NOP2 nucleolar protein homolog (yeast)
10196	PRMT3	protein arginine methyltransferase 3
6949	TCOF1	Treacher Collins-Franceschetti syndrome 1
79711	IPO4	importin 4
3985	LIMK2	LIM domain kinase 2
128239	IQGAP3	IQ motif containing GTPase activating protein 3
26018	LRIG1	leucine-rich repeats and immunoglobulin-like domains 1
58513	EPS15L1	epidermal growth factor receptor pathway substrate 15-like 1

10059	DNM1L	dynamin 1-like
8675	STX16	syntaxin 16
11079	RER1	RER1 retention in endoplasmic reticulum 1 homolog (S. cerevisiae)
2803	GOLGA4	golgin A4
8774	NAPG	N-ethylmaleimide-sensitive factor attachment protein, gamma
29940	DSE	dermatan sulfate epimerase
435	ASL	argininosuccinate lyase
58478	ENOPH1	enolase-phosphatase 1
84245	MRI1	methylthioribose-1-phosphate isomerase homolog (S. cerevisiae)
4548	MTR	5-methyltetrahydrofolate-homocysteine methyltransferase
23474	ETHE1	ethylmalonic encephalopathy 1
5831	PYCR1	pyrroline-5-carboxylate reductase 1
587	BCAT2	branched chain amino-acid transaminase 2, mitochondrial
728294	D2HGDH	D-2-hydroxyglutarate dehydrogenase
55066	PDPR	pyruvate dehydrogenase phosphatase regulatory subunit
4705	NDUFA10	NADH dehydrogenase (ubiquinone) 1 alpha subcomplex, 10, 42kDa
55967	NDUFA12	NADH dehydrogenase (ubiquinone) 1 alpha subcomplex, 12
91703	ACY3	aspartoacylase (aminocyclase) 3
7915	ALDH5A1	aldehyde dehydrogenase 5 family, member A1
56954	NIT2	nitrilase family, member 2
8312	AXIN1	axin 1
162989	DEDD2	death effector domain containing 2
90427	BMF	Bcl2 modifying factor
51176	LEF1	lymphoid enhancer-binding factor 1
8567	MADD	MAP-kinase activating death domain
10950	BTG3	BTG family, member 3
8682	PEA15	phosphoprotein enriched in astrocytes 15
27347	STK39	serine threonine kinase 39
2339	FNTA	farnesyltransferase, CAAX box, alpha
23324	MAN2B2	mannosidase, alpha, class 2B, member 2
4123	MAN2C1	mannosidase, alpha, class 2C, member 1
64421	DCLRE1C	DNA cross-link repair 1C
27434	POLM	polymerase (DNA directed), mu
11284	PNKP	polynucleotide kinase 3'-phosphatase
7094	TLN1	talin 1
1663	DDX11	DEAD/H (Asp-Glu-Ala-Asp/His) box polypeptide 11
6778	STAT6	signal transducer and activator of transcription 6, interleukin-4 induced
22800	RRAS2	related RAS viral (r-ras) oncogene homolog 2
6789	STK4	serine/threonine kinase 4
5971	RELB	v-rel reticuloendotheliosis viral oncogene homolog B
55072	RNF31	ring finger protein 31
9830	TRIM14	tripartite motif containing 14
1997	ELF1	E74-like factor 1 (ets domain transcription factor)
135112	NCOA7	nuclear receptor coactivator 7
85363	TRIM5	tripartite motif containing 5
116985	ARAP1	ArfGAP with RhoGAP domain, ankyrin repeat and PH domain 1
257106	ARHGAP30	Rho GTPase activating protein 30
51291	GMIP	GEM interacting protein
51306	FAM13B	family with sequence similarity 13, member B
58504	ARHGAP22	Rho GTPase activating protein 22
9712	USP6NL	USP6 N-terminal like
146956	EME1	essential meiotic endonuclease 1 homolog 1 (S. pombe)
25788	RAD54B	RAD54 homolog B (S. cerevisiae)
4439	MSH5	mutS homolog 5 (E. coli)
3633	INPP5B	inositol polyphosphate-5-phosphatase, 75kDa
3150	HMGN1	high mobility group nucleosome binding domain 1
3682	ITGAE	tegrin, alpha E (antigen CD103, human mucosal lymphocyte antigen 1; alpha polypeptide)
10152	ABI2	abl-interactor 2
4636	MYL5	myosin, light chain 5, regulatory
1730	DIAPH2	diaphanous homolog 2 (Drosophila)
221955	DAGLB	diacylglycerol lipase, beta
3833	KIFC1	kinesin family member C1
81704	DOCK8	dedicator of cytokinesis 8
7804	LRP8	low density lipoprotein receptor-related protein 8, apolipoprotein e receptor
813	CALU	calumenin
56938	ARNTL2	aryl hydrocarbon receptor nuclear translocator-like 2
1299	COL9A3	collagen, type IX, alpha 3
83933	HDAC10	histone deacetylase 10
7181	NR2C1	nuclear receptor subfamily 2, group C, member 1
51548	SIRT6	sirtuin 6
51547	SIRT7	sirtuin 7
1107	CHD3	chromodomain helicase DNA binding protein 3
54583	EGLN1	egl nine homolog 1 (C. elegans)
2950	GSTP1	glutathione S-transferase pi 1
1857	DVL3	dishevelled, dsh homolog 3 (Drosophila)
27148	STK36	serine/threonine kinase 36
3611	ILK	integrin-linked kinase
1268	CNR1	cannabinoid receptor 1 (brain)
5498	PPOX	protoporphyrinogen oxidase
6446	SGK1	serum/glucocorticoid regulated kinase 1
55763	EXOC1	exocyst complex component 1
65220	NADK	NAD kinase
4194	MDM4	Mdm4 p53 binding protein homolog (mouse)
1877	E4F1	E4F transcription factor 1
27	ABL2	v-abl Abelson murine leukemia viral oncogene homolog 2
6443	SGCB	sarcoglycan, beta (43kDa dystrophin-associated glycoprotein)
79947	DHDDS	dehydrodolichyl diphosphate synthase
144455	E2F7	E2F transcription factor 7
1876	E2F6	E2F transcription factor 6
9744	ACAP1	ArfGAP with coiled-coil, ankyrin repeat and PH domains 1
9135	RABEP1	rabaptin, RAB GTPase binding effector protein 1
116986	AGAP2	ArfGAP with GTPase domain, ankyrin repeat and PH domain 2
5869	RAB5B	RAB5B, member RAS oncogene family
56904	SH3GLB2	SH3-domain GRB2-like endophilin B2
23550	PSD4	pleckstrin and Sec7 domain containing 4
84364	ARFGAP2	ADP-ribosylation factor GTPase activating protein 2
115817	DHRS1	dehydrogenase/reductase (SDR family) member 1
4485	MST1	macrophage stimulating 1 (hepatocyte growth factor-like)
3587	IL10RA	interleukin 10 receptor, alpha
55229	PANK4	pantothenate kinase 4
80347	COASY	CoA synthase
54888	NSUN2	NOP2/Sun domain family, member 2
6929	TCF3	transcription factor 3 (E2A immunoglobulin enhancer binding factors E12/E47)
9855	FARP2	FERM, RhoGEF and pleckstrin domain protein 2
4637	MYL6	myosin, light chain 6, alkali, smooth muscle and non-muscle
64218	SEMA4A	munoglobulin domain (Ig), transmembrane domain (TM) and short cytoplasmic domain, (semaphorin) 4A
57731	SPTBN4	spectrin, beta, non-erythrocytic 4
326625	MMAB	methylmalonic aciduria (cobalamin deficiency) cblB type
10325	RRAGB	Ras-related GTP binding B
10956	OS9	osteosarcoma amplified 9, endoplasmic reticulum lectin
4771	NF2	neurofibromin 2 (merlin)
1265	CNN2	calponin 2
5310	PKD1	polycystic kidney disease 1 (autosomal dominant)
8910	SGCE	sarcoglycan, epsilon
1039	CDR2	cerebellar degeneration-related protein 2, 62kDa
26268	FBXO9	F-box protein 9
6397	SEC14L1	SEC14-like 1 (S. cerevisiae)
831	CAST	calpastatin
84809	CROCCP2	ciliary rootlet coiled-coil, rootletin pseudogene 2
9144	SYNGR2	synaptogyrin 2
9910	RABGAP1L	RAB GTPase activating protein 1-like
22807	IKZF2	IKAROS family zinc finger 2 (Helios)
23075	SWAP70	SWAP switching B-cell complex 70kDa subunit
56990	CDC42SE2	CDC42 small effector 2
57157	PHTF2	putative homeodomain transcription factor 2
57231	SNX14	sorting nexin 14
64332	NFKBIZ	nuclear factor of kappa light polypeptide gene enhancer in B-cells inhibitor, zeta
26053	AUTS2	autism susceptibility candidate 2
4046	LSP1	lymphocyte-specific protein 1
9260	PDLIM7	PDZ and LIM domain 7 (enigma)
2314	FLII	flightless I homolog (Drosophila)
5348	FXYD1	FXYD domain containing ion transport regulator 1
57534	MIB1	mindbomb homolog 1 (Drosophila)
27327	TNRC6A	trinucleotide repeat containing 6A
4242	MFNG	MFNG O-fucosylpeptide 3-beta-N-acetylglucosaminyltransferase
56983	POGLUT1	protein O-glucosyltransferase 1
5586	PKN2	protein kinase N2
2011	MARK2	MAP/microtubule affinity-regulating kinase 2
114790	STK11IP	serine/threonine kinase 11 interacting protein
1787	TRDMT1	tRNA aspartic acid methyltransferase 1
1789	DNMT3B	DNA (cytosine-5-)-methyltransferase 3 beta
7335	UBE2V1	ubiquitin-conjugating enzyme E2 variant 1
22900	CARD8	caspase recruitment domain family, member 8
55914	ERBB2IP	erbb2 interacting protein
7462	LAT2	linker for activation of T cells family, member 2
8437	RASAL1	RAS protein activator like 1 (GAP1 like)
475	ATOX1	ATX1 antioxidant protein 1 homolog (yeast)
4155	MBP	myelin basic protein
7511	XPNPEP1	X-prolyl aminopeptidase (aminopeptidase P) 1, soluble
10994	ILVBL	ilvB (bacterial acetolactate synthase)-like
23210	JMJD6	jumonji domain containing 6
2356	FPGS	folylpolyglutamate synthase
11315	PARK7	parkinson protein 7
120892	LRRK2	leucine-rich repeat kinase 2
83605	CCM2	cerebral cavernous malformation 2
2289	FKBP5	FK506 binding protein 5
55768	NGLY1	N-glycanase 1
112483	SAT2	spermidine/spermine N1-acetyltransferase family member 2
29920	PYCR2	pyrroline-5-carboxylate reductase family, member 2
6597	SMARCA4	NF related, matrix associated, actin dependent regulator of chromatin, subfamily a, member 4
6598	SMARCB1	NF related, matrix associated, actin dependent regulator of chromatin, subfamily b, member 1
9942	XYLB	xylulokinase homolog (H. influenzae)
114049	WBSCR22	Williams Beuren syndrome chromosome region 22
131965	METTL6	methyltransferase like 6
60487	TRMT11	tRNA methyltransferase 11 homolog (S. cerevisiae)
1600	DAB1	disabled homolog 1 (Drosophila)
2054	STX2	syntaxin 2
3397	ID1	inhibitor of DNA binding 1, dominant negative helix-loop-helix protein
22884	WDR37	WD repeat domain 37
25836	NIPBL	Nipped-B homolog (Drosophila)
26037	SIPA1L1	signal-induced proliferation-associated 1 like 1
27109	ATP5S	ATP synthase, H+ transporting, mitochondrial Fo complex, subunit s (factor B)
55349	CHDH	choline dehydrogenase
23081	KDM4C	lysine (K)-specific demethylase 4C
23598	PATZ1	POZ (BTB) and AT hook containing zinc finger 1
3064	HTT	huntingtin
25981	DNAH1	dynein, axonemal, heavy chain 1
286151	FBXO43	F-box protein 43
6601	SMARCC2	NF related, matrix associated, actin dependent regulator of chromatin, subfamily c, member 2
57060	PCBP4	poly(rC) binding protein 4
8836	GGH	gamma-glutamyl hydrolase (conjugase, folylpolygammaglutamyl hydrolase)
6474	SHOX2	short stature homeobox 2
51512	GTSE1	G-2 and S-phase expressed 1
55540	IL17RB	interleukin 17 receptor B
203062	TSNARE1	t-SNARE domain containing 1
7391	USF1	upstream transcription factor 1
9765	ZFYVE16	zinc finger, FYVE domain containing 16
196294	IMMP1L	IMP1 inner mitochondrial membrane peptidase-like (S. cerevisiae)
7095	SEC62	SEC62 homolog (S. cerevisiae)
84264	HAGHL	hydroxyacylglutathione hydrolase-like
27065	D4S234E	DNA segment on chromosome 4 (unique) 234 expressed sequence
3297	HSF1	heat shock transcription factor 1
6935	ZEB1	zinc finger E-box binding homeobox 1
80895	ILKAP	integrin-linked kinase-associated serine/threonine phosphatase
57018	CCNL1	cyclin L1
9120	SLC16A6	solute carrier family 16, member 6 (monocarboxylic acid transporter 7)
84233	TMEM126A	transmembrane protein 126A
4603	MYBL1	v-myb myeloblastosis viral oncogene homolog (avian)-like 1
6046	BRD2	bromodomain containing 2
444	ASPH	aspartate beta-hydroxylase
23523	CABIN1	calcineurin binding protein 1
9988	DMTF1	cyclin D binding myb-like transcription factor 1
23373	CRTC1	CREB regulated transcription coactivator 1
79017	GGCT	gamma-glutamylcyclotransferase
59343	SENP2	SUMO1/sentrin/SMT3 specific peptidase 2
23623	RUSC1	RUN and SH3 domain containing 1
55743	CHFR	checkpoint with forkhead and ring finger domains
79882	ZC3H14	zinc finger CCCH-type containing 14
8028	MLLT10	oid/lymphoid or mixed-lineage leukemia (trithorax homolog, Drosophila); translocated to, 10
10629	TAF6L	AF6-like RNA polymerase II, p300/CBP-associated factor (PCAF)-associated factor, 65kDa
22937	SCAP	SREBF chaperone
966	CD59	CD59 molecule, complement regulatory protein
55793	FAM63A	family with sequence similarity 63, member A
84187	TMEM164	transmembrane protein 164
9094	UNC119	unc-119 homolog (C. elegans)
951	CD37	CD37 molecule
3783	KCNN4	assium intermediate/small conductance calcium-activated channel, subfamily N, member 4
23531	MMD	monocyte to macrophage differentiation-associated
3936	LCP1	lymphocyte cytosolic protein 1 (L-plastin)
83734	ATG10	ATG10 autophagy related 10 homolog (S. cerevisiae)
9555	H2AFY	H2A histone family, member Y
5875	RABGGTA	Rab geranylgeranyltransferase, alpha subunit
8073	PTP4A2	protein tyrosine phosphatase type IVA, member 2
89845	ABCC10	ATP-binding cassette, sub-family C (CFTR/MRP), member 10
931	MS4A1	membrane-spanning 4-domains, subfamily A, member 1
84498	FAM120B	family with sequence similarity 120B
51399	TRAPPC4	trafficking protein particle complex 4
10777	ARPP21	cAMP-regulated phosphoprotein, 21kDa
22849	CPEB3	cytoplasmic polyadenylation element binding protein 3
54901	CDKAL1	CDK5 regulatory subunit associated protein 1-like 1
79085	SLC25A23	solute carrier family 25 (mitochondrial carrier; phosphate carrier), member 23
8678	BECN1	beclin 1, autophagy related
7392	USF2	upstream transcription factor 2, c-fos interacting
10505	SEMA4F	munoglobulin domain (Ig), transmembrane domain (TM) and short cytoplasmic domain, (semaphorin) 4F
240	ALOX5	arachidonate 5-lipoxygenase
29121	CLEC2D	C-type lectin domain family 2, member D
311	ANXA11	annexin A11
5272	SERPINB9	serpin peptidase inhibitor, clade B (ovalbumin), member 9
79676	OGFOD2	2-oxoglutarate and iron-dependent oxygenase domain containing 2
84957	RELT	RELT tumor necrosis factor receptor
1393	CRHBP	corticotropin releasing hormone binding protein
1954	MEGF8	multiple EGF-like-domains 8
29915	HCFC2	host cell factor C2
203522	DDX26B	DEAD/H (Asp-Glu-Ala-Asp/His) box polypeptide 26B
79290	OR13A1	olfactory receptor, family 13, subfamily A, member 1
654817	NCF1C	neutrophil cytosolic factor 1C pseudogene
7011	TEP1	telomerase-associated protein 1

55250	ELP2	elongation protein 2 homolog (S. cerevisiae)										
100048912	CDKN2B-AS	CDKN2B antisense RNA (non-protein coding)										
100093630	SNHG8	small nucleolar RNA host gene 8 (non-protein coding)										
100131017	ZNF316	zinc finger protein 316										
100131187	TSTD1	thiosulfate sulfurtransferase (rhodanese)-like domain containing 1										
100131998	RRN3P3	RNA polymerase I transcription factor homolog (S. cerevisiae) pseudogene 3										
100289678	ZNF783	zinc finger family member 783										
100302692	FTX	FTX transcript, XIST regulator (non-protein coding)										
10045	SH2D3A	SH2 domain containing 3A										
10101	NUBP2	nucleotide binding protein 2										
10139	ARFRP1	ADP-ribosylation factor related protein 1										
10147	SUGP2	SURP and G patch domain containing 2										
10169	SERF2	small EDRK-rich factor 2										
10180	RBM6	RNA binding motif protein 6										
10184	LHFPL2	lipoma HMGIC fusion partner-like 2										
10198	MPHOSPH9	M-phase phosphoprotein 9										
10227	MFSD10	major facilitator superfamily domain containing 10										
10260	DENND4A	DENN/MADD domain containing 4A										
10295	BCKDK	branched chain ketoacid dehydrogenase kinase										
10307	APBB3	amyloid beta (A4) precursor protein-binding, family B, member 3										
10336	PCGF3	polycomb group ring finger 3										
10346	TRIM22	tripartite motif containing 22										
10467	ZNHIT1	zinc finger, HIT-type containing 1										
10475	TRIM38	tripartite motif containing 38										
10534	SSSCA1	Sjogren syndrome/scleroderma autoantigen 1										
10539	GLRX3	glutaredoxin 3										
10585	POMT1	protein-O-mannosyltransferase 1										
10625	IVNS1ABP	influenza virus NS1A binding protein										
10626	TRIM16	tripartite motif containing 16										
10641	NPRL2	nitrogen permease regulator-like 2 (S. cerevisiae)										
10658	CELF1	CUGBP, Elav-like family member 1										
10749	KIF1C	kinesin family member 1C										
10807	SDCCAG3	serologically defined colon cancer antigen 3										
10922	FASTK	Fas-activated serine/threonine kinase										
10944	C11orf58	chromosome 11 open reading frame 58										
11011	TLK2	tousled-like kinase 2										
11019	LIAS	lipoic acid synthetase										
11020	IFT27	intraflagellar transport 27 homolog (Chlamydomonas)										
11101	ATE1	arginyltransferase 1										
11102	RPP14	ribonuclease P/MRP 14kDa subunit										
11119	BTN3A1	butyrophilin, subfamily 3, member A1										
11120	BTN2A1	butyrophilin, subfamily 2, member A1										
11123	RCAN3	RCAN family member 3										
11129	CLASRP	CLK4-associating serine/arginine rich protein										
11132	CAPN10	calpain 10										
11177	BAZ1A	bromodomain adjacent to zinc finger domain, 1A										
11212	PROSC	proline synthetase co-transcribed homolog (bacterial)										
11219	TREX2	three prime repair exonuclease 2										
11234	HPS5	Hermansky-Pudlak syndrome 5										
11257	TP53TG1	TP53 target 1 (non-protein coding)										
11262	SP140	SP140 nuclear body protein										
11272	PRR4	proline rich 4 (lacrimal)										
113000	RPUSD1	RNA pseudouridylate synthase domain containing 1										
113177	IZUMO4	IZUMO family member 4										
11322	TMC6	transmembrane channel-like 6										
113277	TMEM106A	transmembrane protein 106A										
113510	HELQ	helicase, POLQ-like										
113655	MFSD3	major facilitator superfamily domain containing 3										
114881	OSBPL7	oxysterol binding protein-like 7										
114883	OSBPL9	oxysterol binding protein-like 9										
114884	OSBPL10	oxysterol binding protein-like 10										
115209	OMA1	OMA1 homolog, zinc metallopeptidase (S. cerevisiae)										
115286	SLC25A26	solute carrier family 25, member 26										
1153	CIRBP	cold inducible RNA binding protein										
115350	FCRL1	Fc receptor-like 1										
115352	FCRL3	Fc receptor-like 3										
115353	LRRC42	leucine rich repeat containing 42										
115426	UHRF2	ubiquitin-like with PHD and ring finger domains 2										
115811	IQCD	IQ motif containing D										
116931	MED12L	mediator complex subunit 12-like										
118433	RPL23AP7	ribosomal protein L23a pseudogene 7										
1185	CLCN6	chloride channel 6										
118813	ZFYVE27	zinc finger, FYVE domain containing 27										
118980	SFXN2	sideroflexin 2										
119504	ANAPC16	anaphase promoting complex subunit 16										
122509	IFI27L1	interferon, alpha-inducible protein 27-like 1										
122704	MRPL52	mitochondrial ribosomal protein L52										
123606	NIPA1	non imprinted in Prader-Willi/Angelman syndrome 1										
123803	NTAN1	N-terminal asparagine amidase										
124093	CCDC78	coiled-coil domain containing 78										
124401	ANKS3	ankyrin repeat and sterile alpha motif domain containing 3										
124512	METTL23	methyltransferase like 23										
124540	MSI2	musashi homolog 2 (Drosophila)										
124790	HEXIM2	hexamethylene bis-acetamide inducible 2										
124997	WDR81	WD repeat domain 81										
125150	ZSWIM7	zinc finger, SWIM-type containing 7										
125488	TTC39C	tetratricopeptide repeat domain 39C										
126133	ALDH16A1	aldehyde dehydrogenase 16 family, member A1										
126526	C19orf47	chromosome 19 open reading frame 47										
126549	ANKLE1	ankyrin repeat and LEM domain containing 1										
127002	ATXN7L2	ataxin 7-like 2										
127262	TPRG1L	tumor protein p63 regulated 1-like										
128308	MRPL55	mitochondrial ribosomal protein L55										
128387	TATDN3	TatD DNase domain containing 3										
129530	LYG1	lysozyme G-like 1										
129531	MITD1	MIT, microtubule interacting and transport, domain containing 1										
129563	DIS3L2	DIS3 mitotic control homolog (S. cerevisiae)-like 2										
129685	TAF8	TAF8 RNA polymerase II, TATA box binding protein (TBP)-associated factor, 43kDa										
130507	UBR3	ubiquitin protein ligase E3 component n-recognin 3 (putative)										
131601	TPRA1	transmembrane protein, adipocyte asscociated 1										
131616	TMEM42	transmembrane protein 42										
133015	PACRGL	PARK2 co-regulated-like										
134492	NUDCD2	NudC domain containing 2										
134637	ADAT2	adenosine deaminase, tRNA-specific 2										
137695	TMEM68	transmembrane protein 68										
14	AAMP	angio-associated, migratory cell protein										
140890	SREK1	splicing regulatory glutamine/lysine-rich protein 1										
141	ADPRH	ADP-ribosylarginine hydrolase										
1416	CRYBB2P1	crystallin, beta B2 pseudogene 1										
144097	C11orf84	chromosome 11 open reading frame 84										
144132	DNHD1	dynein heavy chain domain 1										
144406	WDR66	WD repeat domain 66										
145773	FAM81A	family with sequence similarity 81, member A										
146059	CDAN1	congenital dyserythropoietic anemia, type I										
146206	RLTPR	RGD motif, leucine rich repeats, tropomodulin domain and proline-rich containing										
147179	WIPF2	WAS/WASL interacting protein family, member 2										
147965	FAM98C	family with sequence similarity 98, member C										
147991	DPY19L3	dpy-19-like 3 (C. elegans)										
148206	ZNF714	zinc finger protein 714										
149986	LSM14B	LSM14B, SCD6 homolog B (S. cerevisiae)										
150223	YDJC	YdjC homolog (bacterial)										
150472	CBWD2	COBW domain containing 2										
150726	FBXO41	F-box protein 41										
150771	ITPRIPL1	inositol 1,4,5-trisphosphate receptor interacting protein-like 1										
150962	PUS10	pseudouridylate synthase 10										
151194	METTL21A	methyltransferase like 21A										
151195	CCNYL1	cyclin Y-like 1										
151525	WDSUB1	WD repeat, sterile alpha motif and U-box domain containing 1										
151613	TTC14	tetratricopeptide repeat domain 14										
152006	RNF38	ring finger protein 38										
152815	THAP6	THAP domain containing 6										
153129	SLC38A9	solute carrier family 38, member 9										
154313	C6orf165	chromosome 6 open reading frame 165										
157657	C8orf37	chromosome 8 open reading frame 37										
158056	MAMDC4	MAM domain containing 4										
158293	FAM120AOS	family with sequence similarity 120A opposite strand										
160418	TMTC3	transmembrane and tetratricopeptide repeat containing 3										
160518	DENND5B	DENN/MADD domain containing 5B										
163115	ZNF781	zinc finger protein 781										
163131	ZNF780B	zinc finger protein 780B										
163486	DENND1B	DENN/MADD domain containing 1B										
164118	TTC24	tetratricopeptide repeat domain 24										
165055	CCDC138	coiled-coil domain containing 138										
166824	RASSF6	Ras association (RalGDS/AF-6) domain family member 6										
167153	PAPD4	PAP associated domain containing 4										
168451	THAP5	THAP domain containing 5										
169714	QSOX2	quiescin Q6 sulfhydryl oxidase 2										
170958	ZNF525	zinc finger protein 525										
170960	ZNF721	zinc finger protein 721										
171017	ZNF384	zinc finger protein 384										
171586	ABHD3	abhydrolase domain containing 3										
172	AFG3L1P	AFG3 ATPase family gene 3-like 1 (S. cerevisiae), pseudogene										
1773	DNASE1	deoxyribonuclease I										
1802	DPH2	DPH2 homolog (S. cerevisiae)										
1939	EIF2D	eukaryotic translation initiation factor 2D										
197322	ACSF3	acyl-CoA synthetase family member 3										
199786	FAM129C	family with sequence similarity 129, member C										
200014	CC2D1B	coiled-coil and C2 domain containing 1B										
200030	NBPF11	neuroblastoma breakpoint family, member 11										
200081	TXLNA	taxilin alpha										
200894	ARL13B	ADP-ribosylation factor-like 13B										
201254	STRA13	stimulated by retinoic acid 13 homolog (mouse)										
201255	LRRC45	leucine rich repeat containing 45										
201283	AMZ2P1	archaelysin family metallopeptidase 2 pseudogene 1										
201292	TRIM65	tripartite motif containing 65										
201299	RDM1	RAD52 motif 1										
201305	SPNS3	spinster homolog 3 (Drosophila)										
201799	TMEM154	transmembrane protein 154										
201931	TMEM192	transmembrane protein 192										
2140	EYA3	eyes absent homolog 3 (Drosophila)										
2186	BPTF	bromodomain PHD finger transcription factor										
219790	RTKN2	rhotekin 2										
219899	TBCEL	tubulin folding cofactor E-like										
219931	TPCN2	two pore segment channel 2										
220074	LRTOMT	leucine rich transmembrane and 0-methyltransferase domain containing										
221016	CCDC7	coiled-coil domain containing 7										
221078	NSUN6	NOP2/Sun domain family, member 6										
221302	ZUFSP	zinc finger with UFM1-specific peptidase domain										
221491	C6orf1	chromosome 6 open reading frame 1										
2218	FKTN	fukutin										
222068	TMED4	transmembrane emp24 protein transport domain containing 4										
222256	CDHR3	cadherin-related family member 3										
22864	R3HDM2	R3H domain containing 2										
22870	PPP6R1	protein phosphatase 6, regulatory subunit 1										
22880	MORC2	MORC family CW-type zinc finger 2										
22883	CLSTN1	calsyntenin 1										
22898	DENND3	DENN/MADD domain containing 3										
22902	RUFY3	RUN and FYVE domain containing 3										
22907	DHX30	DEAH (Asp-Glu-Ala-His) box polypeptide 30										
22980	TCF25	transcription factor 25 (basic helix-loop-helix)										
23023	TMCC1	transmembrane and coiled-coil domain family 1										
23032	USP33	ubiquitin specific peptidase 33										
23038	WDTC1	WD and tetratricopeptide repeats 1										
23048	FNBP1	formin binding protein 1										
23064	SETX	senataxin										
23077	MYCBP2	MYC binding protein 2										
23093	TTLL5	tubulin tyrosine ligase-like family, member 5										
23107	MRPS27	mitochondrial ribosomal protein S27										
23113	CUL9	cullin 9										
23122	CLASP2	cytoplasmic linker associated protein 2										
23125	CAMTA2	calmodulin binding transcription activator 2										
23130	ATG2A	ATG2 autophagy related 2 homolog A (S. cerevisiae)										
23131	GPATCH8	G patch domain containing 8										
23155	CLCC1	chloride channel CLIC-like 1										
23180	RFTN1	raftlin, lipid raft linker 1										
23181	DIP2A	DIP2 disco-interacting protein 2 homolog A (Drosophila)										
23189	KANK1	KN motif and ankyrin repeat domains 1										
23207	PLEKHM2	pleckstrin homology domain containing, family M (with RUN domain) member 2										
23211	ZC3H4	zinc finger CCCH-type containing 4										
23214	XPO6	exportin 6										
23215	PRRC2C	proline-rich coiled-coil 2C										
23216	TBC1D1	TBC1 (tre-2/USP6, BUB2, cdc16) domain family, member 1										
23218	NBEAL2	neurobeachin-like 2										
23231	SEL1L3	sel-1 suppressor of lin-12-like 3 (C. elegans)										
23247	KIAA0556	KIAA0556										
23262	PPIP5K2	diphosphoinositol pentakisphosphate kinase 2										
23269	MGA	MAX gene associated										
23274	CLEC16A	C-type lectin domain family 16, member A										
23276	KLHL18	kelch-like 18 (Drosophila)										
23301	EHBP1	EH domain binding protein 1										
23316	CUX2	cut-like homeobox 2										
23334	SZT2	seizure threshold 2 homolog (mouse)										
2334	AFF2	AF4/FMR2 family, member 2										
23341	DNAJC16	DnaJ (Hsp40) homolog, subfamily C, member 16										
23353	SUN1	Sad1 and UNC84 domain containing 1										
23354	HAUS5	HAUS augmin-like complex, subunit 5										
23355	VPS8	vacuolar protein sorting 8 homolog (S. cerevisiae)										
23358	USP24	ubiquitin specific peptidase 24										
23360	FNBP4	formin binding protein 4										
23367	LARP1	La ribonucleoprotein domain family, member 1										
23369	PUM2	pumilio homolog 2 (Drosophila)										
23383	MAU2	MAU2 chromatid cohesion factor homolog (C. elegans)										
23384	SPECC1L	sperm antigen with calponin homology and coiled-coil domains 1-like										
23386	NUDCD3	NudC domain containing 3										
23400	ATP13A2	ATPase type 13A2										
23412	COMMD3	COMM domain containing 3										
23512	SUZ12	suppressor of zeste 12 homolog (Drosophila)										
23513	SCRIB	scribbled homolog (Drosophila)										
23518	R3HDM1	R3H domain containing 1										
23559	WBP1	WW domain binding protein 1										
23609	MKRN2	makorin ring finger protein 2										
23642	SNHG1	small nucleolar RNA host gene 1 (non-protein coding)										
23683	PRKD3	protein kinase D3										
23787	MTCH1	mitochondrial carrier 1										
24139	EML2	echinoderm microtubule associated protein like 2										
246329	STAC3	SH3 and cysteine rich domain 3										
253725	FAM21C	family with sequence similarity 21, member C										

253769	WDR27	WD repeat domain 27										
253959	RALGAPA1	Ral GTPase activating protein, alpha subunit 1 (catalytic)										
254295	PHYHD1	phytanoyl-CoA dioxygenase domain containing 1										
2551	GABPA	GA binding protein transcription factor, alpha subunit 60kDa										
256236	NAPSB	napsin B aspartic peptidase pseudogene										
256364	EML3	echinoderm microtubule associated protein like 3										
257218	SHPRH	SNF2 histone linker PHD RING helicase										
25814	ATXN10	ataxin 10										
25844	YIPF3	Yip1 domain family, member 3										
25852	ARMC8	armadillo repeat containing 8										
25865	PRKD2	protein kinase D2										
25875	LETMD1	LETM1 domain containing 1										
25900	IFFO1	intermediate filament family orphan 1										
25912	C1orf43	chromosome 1 open reading frame 43										
25917	THUMPD3	THUMP domain containing 3										
25925	ZNF521	zinc finger protein 521										
259266	ASPM	asp (abnormal spindle) homolog, microcephaly associated (Drosophila)										
25956	SEC31B	SEC31 homolog B (S. cerevisiae)										
25957	PNISR	PNN-interacting serine/arginine-rich protein										
25959	KANK2	KN motif and ankyrin repeat domains 2										
25961	NUDT13	nudix (nucleoside diphosphate linked moiety X)-type motif 13										
26000	TBC1D10B	TBC1 domain family, member 10B										
26005	C2CD3	C2 calcium-dependent domain containing 3										
26009	ZZZ3	zinc finger, ZZ-type containing 3										
26013	L3MBTL1	l(3)mbt-like 1 (Drosophila)										
26031	OSBPL3	oxysterol binding protein-like 3										
26036	ZNF451	zinc finger protein 451										
26039	SS18L1	synovial sarcoma translocation gene on chromosome 18-like 1										
26058	GIGYF2	GRB10 interacting GYF protein 2										
26065	LSM14A	LSM14A, SCD6 homolog A (S. cerevisiae)										
26123	TCTN3	tectonic family member 3										
26140	TTLL3	tubulin tyrosine ligase-like family, member 3										
26151	NAT9	N-acetyltransferase 9 (GCN5-related, putative)										
26233	FBXL6	F-box and leucine-rich repeat protein 6										
26276	VPS33B	vacuolar protein sorting 33 homolog B (yeast)										
2630	GBAP1	glucosidase, beta, acid pseudogene 1										
26751	SH3YL1	SH3 domain containing, Ysc84-like 1 (S. cerevisiae)										
26960	NBEA	neurobeachin										
26993	AKAP8L	A kinase (PRKA) anchor protein 8-like										
27005	USP21	ubiquitin specific peptidase 21										
27037	TRMT2A	TRM2 tRNA methyltransferase 2 homolog A (S. cerevisiae)										
27130	INVS	inversin										
27158	NDOR1	NADPH dependent diflavin oxidoreductase 1										
27314	RAB30	RAB30, member RAS oncogene family										
27315	PGAP2	post-GPI attachment to proteins 2										
27352	SGSM3	small G protein signaling modulator 3										
27433	TOR2A	torsin family 2, member A										
283234	CCDC88B	coiled-coil domain containing 88B										
283237	TTC9C	tetratricopeptide repeat domain 9C										
283373	ANKRD52	ankyrin repeat domain 52										
283989	TSEN54	tRNA splicing endonuclease 54 homolog (S. cerevisiae)										
284323	ZNF780A	zinc finger protein 780A										
284349	ZNF283	zinc finger protein 283										
284403	WDR62	WD repeat domain 62										
284565	NBPF15	neuroblastoma breakpoint family, member 15										
284697	BTBD8	BTB (POZ) domain containing 8										
284900	TTC28-AS1	TTC28 antisense RNA 1 (non-protein coding)										
284992	CCDC150	coiled-coil domain containing 150										
28511	NKIRAS2	NFKB inhibitor interacting Ras-like 2										
285193	DUSP28	dual specificity phosphatase 28										
285331	CCDC66	coiled-coil domain containing 66										
285989	ZNF789	zinc finger protein 789										
286257	C9orf142	chromosome 9 open reading frame 142										
2874	GPS2	G protein pathway suppressor 2										
28956	LAMTOR2	late endosomal/lysosomal adaptor, MAPK and MTOR activator 2										
28957	MRPS28	mitochondrial ribosomal protein S28										
28976	ACAD9	acyl-CoA dehydrogenase family, member 9										
29015	SLC43A3	solute carrier family 43, member 3										
29066	ZC3H7A	zinc finger CCCH-type containing 7A										
29083	GTPBP8	GTP-binding protein 8 (putative)										
29086	BABAM1	BRISC and BRCA1 A complex member 1										
29087	THYN1	thymocyte nuclear protein 1										
29098	RANGRF	RAN guanine nucleotide release factor										
29115	SAP30BP	SAP30 binding protein										
29123	ANKRD11	ankyrin repeat domain 11										
2975	GTF3C1	general transcription factor IIIC, polypeptide 1, alpha 220kDa										
29761	USP25	ubiquitin specific peptidase 25										
29789	OLA1	Obg-like ATPase 1										
29855	UBN1	ubinuclein 1										
29941	PKN3	protein kinase N3										
29952	DPP7	dipeptidyl-peptidase 7										
29960	FTSJ2	FtsJ homolog 2 (E. coli)										
29990	PILRB	paired immunoglobin-like type 2 receptor beta										
29994	BAZ2B	bromodomain adjacent to zinc finger domain, 2B										
30968	STOML2	stomatin (EPB72)-like 2										
317662	FAM149B1	family with sequence similarity 149, member B1										
317781	DDX51	DEAD (Asp-Glu-Ala-Asp) box polypeptide 51										
3257	HPS1	Hermansky-Pudlak syndrome 1										
327	APEH	N-acylaminoacyl-peptide hydrolase										
3300	DNAJB2	DnaJ (Hsp40) homolog, subfamily B, member 2										
3344	FOXN2	forkhead box N2										
338657	CCDC84	coiled-coil domain containing 84										
338692	ANKRD13D	ankyrin repeat domain 13 family, member D										
339123	JMJD8	jumonji domain containing 8										
339487	ZBTB8OS	zinc finger and BTB domain containing 8 opposite strand										
339500	ZNF678	zinc finger protein 678										
339804	C2orf74	chromosome 2 open reading frame 74										
340252	ZNF680	zinc finger protein 680										
343099	CCDC18	coiled-coil domain containing 18										
346389	MACC1	metastasis associated in colon cancer 1										
347240	KIF24	kinesin family member 24										
347862	PDDC1	Parkinson disease 7 domain containing 1										
348180	CTU2	cytosolic thiouridylase subunit 2 homolog (S. pombe)										
3508	IGHMBP2	immunoglobulin mu binding protein 2										
353497	POLN	polymerase (DNA directed) nu										
374395	TMEM179B	transmembrane protein 179B										
374403	TBC1D10C	TBC1 domain family, member 10C										
374618	TEX9	testis expressed 9										
375248	ANKRD36	ankyrin repeat domain 36										
378938	MALAT1	metastasis associated lung adenocarcinoma transcript 1 (non-protein coding)										
386593	CHKB-CPT1B	CHKB-CPT1B readthrough (non-protein coding)										
387066	SNHG5	small nucleolar RNA host gene 5 (non-protein coding)										
387103	CENPW	centromere protein W										
387338	NSUN4	NOP2/Sun domain family, member 4										
388558	ZNF808	zinc finger protein 808										
3899	AFF3	AF4/FMR2 family, member 3										
399818	METTL10	methyltransferase like 10										
400322	HERC2P2	hect domain and RLD 2 pseudogene 2										
400818	NBPF9	neuroblastoma breakpoint family, member 9										
400954	EML6	echinoderm microtubule associated protein like 6										
401262	CRIP3	cysteine-rich protein 3										
4034	LRCH4	leucine-rich repeats and calponin homology (CH) domain containing 4										
4152	MBD1	methyl-CpG binding domain protein 1										
4154	MBNL1	muscleblind-like (Drosophila)										
4302	MLLT6	loid/lymphoid or mixed-lineage leukemia (trithorax homolog, Drosophila); translocated to, 6										
440590	ZYG11A	zyg-11 homolog A (C. elegans)										
440712	C1orf186	chromosome 1 open reading frame 186										
445571	CBWD3	COBW domain containing 3										
4649	MYO9A	myosin IXA										
4676	NAP1L4	nucleosome assembly protein 1-like 4										
4798	NFRKB	nuclear factor related to kappaB binding protein										
4817	NIT1	nitrilase 1										
4925	NUCB2	nucleobindin 2										
4931	NVL	nuclear VCP-like										
494143	CHAC2	ChaC, cation transport regulator homolog 2 (E. coli)										
497661	C18orf32	chromosome 18 open reading frame 32										
50488	MINK1	misshapen-like kinase 1										
50717	DCAF8	DDB1 and CUL4 associated factor 8										
50809	HP1BP3	heterochromatin protein 1, binding protein 3										
51011	FAHD2A	fumarylacetoacetate hydrolase domain containing 2A										
51078	THAP4	THAP domain containing 4										
51088	KLHL5	kelch-like 5 (Drosophila)										
51093	RRNAD1	ribosomal RNA adenine dimethylase domain containing 1										
51095	TRNT1	tRNA nucleotidyl transferase, CCA-adding, 1										
5110	PCMT1	protein-L-isoaspartate (D-aspartate) O-methyltransferase										
51185	CRBN	cereblon										
51249	TMEM69	transmembrane protein 69										
51250	C6orf203	chromosome 6 open reading frame 203										
5127	CDK16	cyclin-dependent kinase 16										
51271	UBAP1	ubiquitin associated protein 1										
5128	CDK17	cyclin-dependent kinase 17										
51304	ZDHHC3	zinc finger, DHHC-type containing 3										
51307	FAM53C	family with sequence similarity 53, member C										
51329	ARL6IP4	ADP-ribosylation-like factor 6 interacting protein 4										
51367	POP5	processing of precursor 5, ribonuclease P/MRP subunit (S. cerevisiae)										
51368	TEX264	testis expressed 264										
51426	POLK	polymerase (DNA directed) kappa										
51463	GPR89B	G protein-coupled receptor 89B										
51479	ANKFY1	ankyrin repeat and FYVE domain containing 1										
51490	C9orf114	chromosome 9 open reading frame 114										
51496	CTDSPL2	TD (carboxy-terminal domain, RNA polymerase II, polypeptide A) small phosphatase like 2										
51501	C11orf73	chromosome 11 open reading frame 73										
51522	TMEM14C	transmembrane protein 14C										
51523	CXXC5	CXXC finger protein 5										
51571	FAM49B	family with sequence similarity 49, member B										
51574	LARP7	La ribonucleoprotein domain family, member 7										
51629	SLC25A39	solute carrier family 25, member 39										
51643	TMBIM4	transmembrane BAX inhibitor motif containing 4										
51654	CDK5RAP1	CDK5 regulatory subunit associated protein 1										
51657	STYXL1	serine/threonine/tyrosine interacting-like 1										
51699	VPS29	vacuolar protein sorting 29 homolog (S. cerevisiae)										
51755	CDK12	cyclin-dependent kinase 12										
5218	CDK14	cyclin-dependent kinase 14										
5252	PHF1	PHD finger protein 1										
53373	TPCN1	two pore segment channel 1										
5378	PMS1	PMS1 postmeiotic segregation increased 1 (S. cerevisiae)										
5379	PMS2P1	postmeiotic segregation increased 2 pseudogene 1										
53827	FXYD5	FXYD domain containing ion transport regulator 5										
53917	RAB24	RAB24, member RAS oncogene family										
53938	PPIL3	peptidylprolyl isomerase (cyclophilin)-like 3										
54058	C21orf58	chromosome 21 open reading frame 58										
54332	GDAP1	ganglioside-induced differentiation-associated protein 1										
54431	DNAJC10	DnaJ (Hsp40) homolog, subfamily C, member 10										
54432	YIPF1	Yip1 domain family, member 1										
54454	ATAD2B	ATPase family, AAA domain containing 2B										
54476	RNF216	ring finger protein 216										
54499	TMCO1	transmembrane and coiled-coil domains 1										
54517	PUS7	pseudouridylate synthase 7 homolog (S. cerevisiae)										
54537	FAM35A	family with sequence similarity 35, member A										
54540	FAM193B	family with sequence similarity 193, member B										
54552	GNL3L	guanine nucleotide binding protein-like 3 (nucleolar)-like										
54621	VSIG10	V-set and immunoglobulin domain containing 10										
54676	GTPBP2	GTP binding protein 2										
54758	KLHDC4	kelch domain containing 4										
54790	TET2	tet methylcytosine dioxygenase 2										
54799	MBTD1	mbt domain containing 1										
54814	QPCTL	glutaminyl-peptide cyclotransferase-like										
54826	GIN1	gypsy retrotransposon integrase 1										
54849	DEF8	differentially expressed in FDCP 8 homolog (mouse)										
54851	ANKRD49	ankyrin repeat domain 49										
54853	WDR55	WD repeat domain 55										
54862	CC2D1A	coiled-coil and C2 domain containing 1A										
54867	TMEM214	transmembrane protein 214										
54874	FNBP1L	formin binding protein 1-like										
54876	DCAF16	DDB1 and CUL4 associated factor 16										
54878	DPP8	dipeptidyl-peptidase 8										
54881	TEX10	testis expressed 10										
54883	CWC25	CWC25 spliceosome-associated protein homolog (S. cerevisiae)										
54887	UHRF1BP1	UHRF1 binding protein 1										
54892	NCAPG2	non-SMC condensin II complex, subunit G2										
54921	CHTF8	CTF8, chromosome transmission fidelity factor 8 homolog (S. cerevisiae)										
54930	HAUS4	HAUS augmin-like complex, subunit 4										
54940	OCIAD1	OCIA domain containing 1										
54952	TRNAU1AP	tRNA selenocysteine 1 associated protein 1										
54973	CPSF3L	cleavage and polyadenylation specific factor 3-like										
54976	C20orf27	chromosome 20 open reading frame 27										
54986	ULK4	unc-51-like kinase 4 (C. elegans)										
54987	C1orf123	chromosome 1 open reading frame 123										
54995	OXSM	3-oxoacyl-ACP synthase, mitochondrial										
550	AUP1	ancient ubiquitous protein 1										
55000	TUG1	taurine upregulated 1 (non-protein coding)										
55011	PIH1D1	PIH1 domain containing 1										
55017	C14orf119	chromosome 14 open reading frame 119										
55031	USP47	ubiquitin specific peptidase 47										
55041	PLEKHB2	pleckstrin homology domain containing, family B (evectins) member 2										
55049	C19orf60	chromosome 19 open reading frame 60										
55063	ZCWPW1	zinc finger, CW type with PWWP domain 1										
55080	TAPBPL	TAP binding protein-like										
55084	SOBP	sine oculis binding protein homolog (Drosophila)										
55086	CXorf57	chromosome X open reading frame 57										
55101	ATP5SL	ATP5S-like										
55116	TMEM39B	transmembrane protein 39B										
55133	SRBD1	S1 RNA binding domain 1										
55152	DALRD3	DALR anticodon binding domain containing 3										
55161	TMEM33	transmembrane protein 33										
55173	MRPS10	mitochondrial ribosomal protein S10										
55183	RIF1	RAP1 interacting factor homolog (yeast)										
55187	VPS13D	vacuolar protein sorting 13 homolog D (S. cerevisiae)										
55198	APPL2	daptor protein, phosphotyrosine interaction, PH domain and leucine zipper containing 2										
55208	DCUN1D2	DCN1, defective in cullin neddylation 1, domain containing 2 (S. cerevisiae)										
55209	SETD5	SET domain containing 5										
55249	YY1AP1	YY1 associated protein 1										
55285	RBM41	RNA binding motif protein 41										
55291	PPP6R3	protein phosphatase 6, regulatory subunit 3										
55297	CCDC91	coiled-coil domain containing 91										
55308	DDX19A	DEAD (Asp-Glu-Ala-As) box polypeptide 19A										
55311	ZNF444	zinc finger protein 444										

55316	RSAD1	radical S-adenosyl methionine domain containing 1										
55324	ABCF3	ATP-binding cassette, sub-family F (GCN20), member 3										
55327	LIN7C	lin-7 homolog C (C. elegans)										
554203	JPX	JPX transcript, XIST activator (non-protein coding)										
55536	CDCA7L	cell division cycle associated 7-like										
5554	PRH1	proline-rich protein HaeIII subfamily 1										
55556	ENOSF1	enolase superfamily member 1										
55565	ZNF821	zinc finger protein 821										
55605	KIF21A	kinesin family member 21A										
55617	TASP1	taspase, threonine aspartase, 1										
55621	TRMT1	TRM1 tRNA methyltransferase 1 homolog (S. cerevisiae)										
55624	POMGNT1	protein O-linked mannose beta1,2-N-acetylglucosaminyltransferase										
55644	OSGEP	O-sialoglycoprotein endopeptidase										
55657	ZNF692	zinc finger protein 692										
55658	RNF126	ring finger protein 126										
55672	NBPF1	neuroblastoma breakpoint family, member 1										
55687	TRMU	tRNA 5-methylaminomethyl-2-thiouridylate methyltransferase										
55689	YEATS2	YEATS domain containing 2										
55692	LUC7L	LUC7-like (S. cerevisiae)										
55720	TSR1	TSR1, 20S rRNA accumulation, homolog (S. cerevisiae)										
55756	INTS9	integrator complex subunit 9										
55758	RCOR3	REST corepressor 3										
55761	TTC17	tetratricopeptide repeat domain 17										
55769	ZNF83	zinc finger protein 83										
55784	MCTP2	multiple C2 domains, transmembrane 2										
55803	ADAP2	ArfGAP with dual PH domains 2										
55814	BDP1	B double prime 1, subunit of RNA polymerase III transcription initiation factor IIIB										
55823	VPS11	vacuolar protein sorting 11 homolog (S. cerevisiae)										
55830	GLT8D1	glycosyltransferase 8 domain containing 1										
55858	TMEM165	transmembrane protein 165										
55862	ECHDC1	enoyl CoA hydratase domain containing 1										
55871	CBWD1	COBW domain containing 1										
55876	GSDMB	gasdermin B										
55892	MYNN	myoneurin										
55954	ZMAT5	zinc finger, matrin-type 5										
55972	SLC25A40	solute carrier family 25, member 40										
55974	SLC50A1	solute carrier family 50 (sugar transporter), member 1										
56063	TMEM234	transmembrane protein 234										
5612	PRKRIR	nase, interferon-inducible double stranded RNA dependent inhibitor, repressor of (P58 repressor)										
5635	PRPSAP1	phosphoribosyl pyrophosphate synthetase-associated protein 1										
5636	PRPSAP2	phosphoribosyl pyrophosphate synthetase-associated protein 2										
56681	SAR1A	SAR1 homolog A (S. cerevisiae)										
56850	GRIPAP1	GRIP1 associated protein 1										
56882	CDC42SE1	CDC42 small effector 1										
56897	WRNIP1	Werner helicase interacting protein 1										
56927	GPR108	G protein-coupled receptor 108										
56928	SPPL2B	signal peptide peptidase-like 2B										
56943	ENY2	enhancer of yellow 2 homolog (Drosophila)										
56947	MFF	mitochondrial fission factor										
56948	SDR39U1	short chain dehydrogenase/reductase family 39U, member 1										
56987	BBX	bobby sox homolog (Drosophila)										
56995	TULP4	tubby like protein 4										
56996	SLC12A9	solute carrier family 12 (potassium/chloride transporters), member 9										
57092	PCNP	PEST proteolytic signal containing nuclear protein										
57130	ATP13A1	ATPase type 13A1										
57140	RNPEPL1	arginyl aminopeptidase (aminopeptidase B)-like 1										
57148	RALGAPB	Ral GTPase activating protein, beta subunit (non-catalytic)										
57156	TMEM63C	transmembrane protein 63C										
57175	CORO1B	coronin, actin binding protein, 1B										
57179	KIAA1191	KIAA1191										
57404	CYP20A1	cytochrome P450, family 20, subfamily A, polypeptide 1										
57409	MIF4GD	MIF4G domain containing										
57410	SCYL1	SCY1-like 1 (S. cerevisiae)										
57465	TBC1D24	TBC1 domain family, member 24										
57473	ZNF512B	zinc finger protein 512B										
57480	PLEKHG1	pleckstrin homology domain containing, family G (with RhoGef domain) member 1										
57507	ZNF608	zinc finger protein 608										
57531	HACE1	HECT domain and ankyrin repeat containing, E3 ubiquitin protein ligase 1										
57536	KIAA1328	KIAA1328										
57560	IFT80	intraflagellar transport 80 homolog (Chlamydomonas)										
57579	FAM135A	family with sequence similarity 135, member A										
57609	DIP2B	DIP2 disco-interacting protein 2 homolog B (Drosophila)										
57614	KIAA1468	KIAA1468										
57646	USP28	ubiquitin specific peptidase 28										
57683	ZDBF2	zinc finger, DBF-type containing 2										
57695	USP37	ubiquitin specific peptidase 37										
57699	CPNE5	copine V										
57730	ANKRD36B	ankyrin repeat domain 36B										
583	BBS2	Bardet-Biedl syndrome 2										
58475	MS4A7	membrane-spanning 4-domains, subfamily A, member 7										
585	BBS4	Bardet-Biedl syndrome 4										
5863	RGL2	ral guanine nucleotide dissociation stimulator-like 2										
59286	UBL5	ubiquitin-like 5										
5965	RECQL	RecQ protein-like (DNA helicase Q1-like)										
6003	RGS13	regulator of G-protein signaling 13										
60313	GPBP1L1	GC-rich promoter binding protein 1-like 1										
60343	FAM3A	family with sequence similarity 3, member A										
60468	BACH2	BTB and CNC homology 1, basic leucine zipper transcription factor 2										
60528	ELAC2	elaC homolog 2 (E. coli)										
60672	MIIP	migration and invasion inhibitory protein										
60674	GAS5	growth arrest-specific 5 (non-protein coding)										
6100	RP9	retinitis pigmentosa 9 (autosomal dominant)										
6293	VPS52	vacuolar protein sorting 52 homolog (S. cerevisiae)										
6302	TSPAN31	tetraspanin 31										
636	BICD1	bicaudal D homolog 1 (Drosophila)										
63892	THADA	thyroid adenoma associated										
63901	FAM111A	family with sequence similarity 111, member A										
63908	NAPB	N-ethylmaleimide-sensitive factor attachment protein, beta										
63922	CHTF18	CTF18, chromosome transmission fidelity factor 18 homolog (S. cerevisiae)										
63925	ZNF335	zinc finger protein 335										
64005	MYO1G	myosin IG										
64062	RBM26	RNA binding motif protein 26										
64118	DUS1L	dihydrouridine synthase 1-like (S. cerevisiae)										
641590	NBPF20	neuroblastoma breakpoint family, member 20										
641638	SNHG6	small nucleolar RNA host gene 6 (non-protein coding)										
641977	SEPT7P2	septin 7 pseudogene 2										
64210	MMS19	MMS19 nucleotide excision repair homolog (S. cerevisiae)										
64319	FBRS	fibrosin										
643836	ZFP62	zinc finger protein 62 homolog (mouse)										
64418	TMEM168	transmembrane protein 168										
64427	TTC31	tetratricopeptide repeat domain 31										
64432	MRPS25	mitochondrial ribosomal protein S25										
645784	ANKRD36BP2	ankyrin repeat domain 36B pseudogene 2										
64601	VPS16	vacuolar protein sorting 16 homolog (S. cerevisiae)										
64770	CCDC14	coiled-coil domain containing 14										
64777	RMND5B	required for meiotic nuclear division 5 homolog B (S. cerevisiae)										
64780	MICAL1	microtubule associated monoxygenase, calponin and LIM domain containing 1										
64783	RBM15	RNA binding motif protein 15										
64852	TUT1	terminal uridylyl transferase 1, U6 snRNA-specific										
64926	RASAL3	RAS protein activator like 3										
64927	TTC23	tetratricopeptide repeat domain 23										
64940	STAG3L4	stromal antigen 3-like 4										
64943	NT5DC2	5'-nucleotidase domain containing 2										
65056	GPBP1	GC-rich promoter binding protein 1										
65083	NOL6	nucleolar protein family 6 (RNA-associated)										
65084	TMEM135	transmembrane protein 135										
65094	JMJD4	jumonji domain containing 4										
65095	KRI1	KRI1 homolog (S. cerevisiae)										
65117	RSRC2	arginine/serine-rich coiled-coil 2										
65123	INTS3	integrator complex subunit 3										
65250	C5orf42	chromosome 5 open reading frame 42										
65258	MPPE1	metallophosphoesterase 1										
654816	NCF1B	neutrophil cytosolic factor 1B pseudogene										
65980	BRD9	bromodomain containing 9										
65981	CAPRIN2	caprin family member 2										
66036	MTMR9	myotubularin related protein 9										
6683	SPAST	spastin										
6687	SPG7	spastic paraplegia 7 (pure and complicated autosomal recessive)										
6813	STXBP2	syntaxin binding protein 2										
6835	SURF2	surfeit 2										
6871	TADA2A	transcriptional adaptor 2A										
6894	TARBP1	TAR (HIV-1) RNA binding protein 1										
7036	TFR2	transferrin receptor 2										
7072	TIA1	TIA1 cytotoxic granule-associated RNA binding protein										
724102	SNHG4	small nucleolar RNA host gene 4 (non-protein coding)										
7260	TSSC1	tumor suppressing subtransferable candidate 1										
7267	TTC3	tetratricopeptide repeat domain 3										
728411	GUSBP1	glucuronidase, beta pseudogene 1										
729440	CCDC61	coiled-coil domain containing 61										
7542	ZFPL1	zinc finger protein-like 1										
7584	ZNF35	zinc finger protein 35										
7629	ZNF76	zinc finger protein 76										
7643	ZNF90	zinc finger protein 90										
7705	ZNF146	zinc finger protein 146										
7748	ZNF195	zinc finger protein 195										
7756	ZNF207	zinc finger protein 207										
7776	ZNF236	zinc finger protein 236										
7866	IFRD2	interferon-related developmental regulator 2										
7871	SLMAP	sarcolemma associated protein										
78996	C7orf49	chromosome 7 open reading frame 49										
79029	SPATA5L1	spermatogenesis associated 5-like 1										
79065	ATG9A	ATG9 autophagy related 9 homolog A (S. cerevisiae)										
79089	TMUB2	transmembrane and ubiquitin-like domain containing 2										
79091	METTL22	methyltransferase like 22										
79101	TAF1D	TATA box binding protein (TBP)-associated factor, RNA polymerase I, D, 41kDa										
79140	CCDC28B	coiled-coil domain containing 28B										
79145	CHCHD7	coiled-coil-helix-coiled-coil-helix domain containing 7										
79154	DHRS11	dehydrogenase/reductase (SDR family) member 11										
7917	BAG6	BCL2-associated athanogene 6										
79228	THOC6	THO complex 6 homolog (Drosophila)										
79363	RSG1	REM2 and RAB-like small GTPase 1										
79368	FCRL2	Fc receptor-like 2										
79415	C17orf62	chromosome 17 open reading frame 62										
79443	FYCO1	FYVE and coiled-coil domain containing 1										
79568	C2orf47	chromosome 2 open reading frame 47										
79590	MRPL24	mitochondrial ribosomal protein L24										
79600	TCTN1	tectonic family member 1										
79618	HMBOX1	homeobox containing 1										
79639	TMEM53	transmembrane protein 53										
79703	C11orf80	chromosome 11 open reading frame 80										
79706	PRKRIP1	PRKR interacting protein 1 (IL11 inducible)										
79713	IGFLR1	IGF-like family receptor 1										
79726	WDR59	WD repeat domain 59										
79734	KCTD17	potassium channel tetramerisation domain containing 17										
79735	TBC1D17	TBC1 domain family, member 17										
79752	ZFAND1	zinc finger, AN1-type domain 1										
79763	ISOC2	isochorismatase domain containing 2										
79811	SLTM	SAFB-like, transcription modulator										
79828	METTL8	methyltransferase like 8										
79839	CCDC102B	coiled-coil domain containing 102B										
79856	SNX22	sorting nexin 22										
79897	RPP21	ribonuclease P/MRP 21kDa subunit										
79930	DOK3	docking protein 3										
79932	KIAA0319L	KIAA0319-like										
79934	ADCK4	aarF domain containing kinase 4										
79956	ERMP1	endoplasmic reticulum metallopeptidase 1										
79958	DENND1C	DENN/MADD domain containing 1C										
79979	TRMT2B	TRM2 tRNA methyltransferase 2 homolog B (S. cerevisiae)										
80011	FAM192A	family with sequence similarity 192, member A										
80012	PHC3	polyhomeotic homolog 3 (Drosophila)										
80013	FAM188A	family with sequence similarity 188, member A										
80017	C14orf159	chromosome 14 open reading frame 159										
80021	TMEM62	transmembrane protein 62										
80063	ATF7IP2	activating transcription factor 7 interacting protein 2										
80067	DCAF17	DDB1 and CUL4 associated factor 17										
80169	CTC1	CTS telomere maintenance complex component 1										
80179	MYO19	myosin XIX										
80199	FUZ	fuzzy homolog (Drosophila)										
80208	SPG11	spastic paraplegia 11 (autosomal recessive)										
80237	ELL3	elongation factor RNA polymerase II-like 3										
80279	CDK5RAP3	CDK5 regulatory subunit associated protein 3										
80305	TRABD	TraB domain containing										
80342	TRAF3IP3	TRAF3 interacting protein 3										
80705	TSGA10	testis specific, 10										
80709	AKNA	AT-hook transcription factor										
80745	THUMPD2	THUMP domain containing 2										
80775	TMEM177	transmembrane protein 177										
80821	DDHD1	DDHD domain containing 1										
81502	HM13	histocompatibility (minor) 13										
81532	MOB2	MOB kinase activator 2										
81533	ITFG1	integrin alpha FG-GAP repeat containing 1										
81573	ANKRD13C	ankyrin repeat domain 13C										
81576	CCDC130	coiled-coil domain containing 130										
81608	FIP1L1	FIP1 like 1 (S. cerevisiae)										
81669	CCNL2	cyclin L2										
81831	NETO2	neuropilin (NRP) and tolloid (TLL)-like 2										
81846	SBF2	SET binding factor 2										
81853	TMEM14B	transmembrane protein 14B										
81890	QTRT1	queuine tRNA-ribosyltransferase 1										
8216	LZTR1	leucine-zipper-like transcription regulator 1										
8242	KDM5C	lysine (K)-specific demethylase 5C										
83451	ABHD11	abhydrolase domain containing 11										
83480	PUS3	pseudouridylate synthase 3										
83596	BCL2L12	BCL2-like 12 (proline rich)										
83707	TRPT1	tRNA phosphotransferase 1										
83746	L3MBTL2	l(3)mbt-like 2 (Drosophila)										
83786	FRMD8	FERM domain containing 8										
83858	ATAD3B	ATPase family, AAA domain containing 3B										
83862	TMEM120A	transmembrane protein 120A										
83891	SNX25	sorting nexin 25										
84064	HDHD2	haloacid dehalogenase-like hydrolase domain containing 2										
84083	ZRANB3	zinc finger, RAN-binding domain containing 3										
84128	WDR75	WD repeat domain 75										
8418	CMAHP	cytidine monophospho-N-acetylneuraminic acid hydroxylase, pseudogene										
84232	MAF1	MAF1 homolog (S. cerevisiae)										
84236	RHBDD1	rhomboid domain containing 1										
84268	RPAIN	RPA interacting protein										

84276	NICN1	nicolin 1										
84278	HIATL2	hippocampus abundant transcript-like 2										
84286	TMEM175	transmembrane protein 175										
84292	WDR83	WD repeat domain 83										
84304	NUDT22	nudix (nucleoside diphosphate linked moiety X)-type motif 22										
84305	WIBG	within bgcn homolog (Drosophila)										
84307	ZNF397	zinc finger protein 397										
84310	C7orf50	chromosome 7 open reading frame 50										
84314	TMEM107	transmembrane protein 107										
84318	CCDC77	coiled-coil domain containing 77										
84324	SARNP	SAP domain containing ribonucleoprotein										
84326	C16orf13	chromosome 16 open reading frame 13										
84331	FAM195A	family with sequence similarity 195, member A										
84456	L3MBTL3	l(3)mbt-like 3 (Drosophila)										
84513	PPAPDC1B	phosphatidic acid phosphatase type 2 domain containing 1B										
84545	MRPL43	mitochondrial ribosomal protein L43										
8458	TTF2	transcription termination factor, RNA polymerase II										
84629	TNRC18	trinucleotide repeat containing 18										
84656	GLYR1	glyoxylate reductase 1 homolog (Arabidopsis)										
84668	FAM126A	family with sequence similarity 126, member A										
84722	PSRC1	proline/serine-rich coiled-coil 1										
8473	OGT	ucosamine (GlcNAc) transferase (UDP-N-acetylglucosamine:polypeptide-N-acetylglucosaminyl transferase)										
84747	UNC119B	unc-119 homolog B (C. elegans)										
84749	USP30	ubiquitin specific peptidase 30										
84824	FCRLA	Fc receptor-like A										
84865	CCDC142	coiled-coil domain containing 142										
84879	MFSD2A	major facilitator superfamily domain containing 2A										
84881	RPUSD4	RNA pseudouridylate synthase domain containing 4										
8490	RGS5	regulator of G-protein signaling 5										
84901	NFATC2IP	iclear factor of activated T-cells, cytoplasmic, calcineurin-dependent 2 interacting protein										
84923	FAM104A	family with sequence similarity 104, member A										
84926	SPRYD3	SPRY domain containing 3										
84936	ZFYVE19	zinc finger, FYVE domain containing 19										
84941	HSH2D	hematopoietic SH2 domain containing										
84942	WDR73	WD repeat domain 73										
84958	SYTL1	synaptotagmin-like 1										
85014	TMEM141	transmembrane protein 141										
85015	USP45	ubiquitin specific peptidase 45										
85302	FBF1	Fas (TNFRSF6) binding factor 1										
8545	CGGBP1	CGG triplet repeat binding protein 1										
85476	GFM1	G elongation factor, mitochondrial 1										
8548	BLZF1	basic leucine zipper nuclear factor 1										
8576	STK16	serine/threonine kinase 16										
85865	GTPBP10	GTP-binding protein 10 (putative)										
8603	FAM193A	family with sequence similarity 193, member A										
8798	DYRK4	dual-specificity tyrosine-(Y)-phosphorylation regulated kinase 4										
88455	ANKRD13A	ankyrin repeat domain 13A										
8847	DLEU2	deleted in lymphocytic leukemia 2 (non-protein coding)										
8859	STK19	serine/threonine kinase 19										
889	KRIT1	KRIT1, ankyrin repeat containing										
8904	CPNE1	copine I										
8906	AP1G2	adaptor-related protein complex 1, gamma 2 subunit										
89848	FCHSD1	FCH and double SH3 domains 1										
89849	ATG16L2	ATG16 autophagy related 16-like 2 (S. cerevisiae)										
89894	TMEM116	transmembrane protein 116										
89970	RSPRY1	ring finger and SPRY domain containing 1										
90313	TP53I13	tumor protein p53 inducible protein 13										
9044	BTAF1	NA polymerase II, B-TFIID transcription factor-associated, 170kDa (Mot1 homolog, S. cerevisiae)										
90799	CEP95	centrosomal protein 95kDa										
90835	C16orf93	chromosome 16 open reading frame 93										
90987	ZNF251	zinc finger protein 251										
91252	SLC39A13	solute carrier family 39 (zinc transporter), member 13										
91289	LMF2	lipase maturation factor 2										
91373	UAP1L1	UDP-N-acteylglucosamine pyrophosphorylase 1-like 1										
91433	RCCD1	RCC1 domain containing 1										
9147	NEMF	nuclear export mediator factor										
91544	UBXN11	UBX domain protein 11										
91582	RPS19BP1	ribosomal protein S19 binding protein 1										
91833	WDR20	WD repeat domain 20										
91860	CALML4	calmodulin-like 4										
9204	ZMYM6	zinc finger, MYM-type 6										
9205	ZMYM5	zinc finger, MYM-type 5										
9209	LRRFIP2	leucine rich repeat (in FLII) interacting protein 2										
92105	INTS4	integrator complex subunit 4										
92170	MTG1	mitochondrial GTPase 1 homolog (S. cerevisiae)										
92305	TMEM129	transmembrane protein 129										
92344	GORAB	golgin, RAB6-interacting										
92714	ARRDC1	arrestin domain containing 1										
92799	SHKBP1	SH3KBP1 binding protein 1										
92822	ZNF276	zinc finger protein 276										
9321	TRIP11	thyroid hormone receptor interactor 11										
93210	PGAP3	post-GPI attachment to proteins 3										
93436	ARMC6	armadillo repeat containing 6										
93627	TBCK	TBC1 domain containing kinase										
9364	RAB28	RAB28, member RAS oncogene family										
9391	CIAO1	cytosolic iron-sulfur protein assembly 1										
9404	LPXN	leupaxin										
94101	ORMDL1	ORM1-like 1 (S. cerevisiae)										
9481	SLC25A27	solute carrier family 25, member 27										
9526	MPDU1	mannose-P-dolichol utilization defect 1										
9535	GMFG	glia maturation factor, gamma										
9545	RAB3D	RAB3D, member RAS oncogene family										
9546	APBA3	amyloid beta (A4) precursor protein-binding, family A, member 3										
9553	MRPL33	mitochondrial ribosomal protein L33										
9557	CHD1L	chromodomain helicase DNA binding protein 1-like										
9581	PREPL	prolyl endopeptidase-like										
9584	RBM39	RNA binding motif protein 39										
9589	WTAP	Wilms tumor 1 associated protein										
9643	MORF4L2	mortality factor 4 like 2										
9647	PPM1F	protein phosphatase, Mg2+/Mn2+ dependent, 1F										
9657	IQCB1	IQ motif containing B1										
9670	IPO13	importin 13										
9681	DEPDC5	DEP domain containing 5										
9701	PPP6R2	protein phosphatase 6, regulatory subunit 2										
9703	KIAA0100	KIAA0100										
9728	SECISBP2L	SECIS binding protein 2-like										
9750	FAM65B	family with sequence similarity 65, member B										
9772	KIAA0195	KIAA0195										
9779	TBC1D5	TBC1 domain family, member 5										
9782	MATR3	matrin 3										
9812	KIAA0141	KIAA0141										
9814	SFI1	Sfi1 homolog, spindle assembly associated (yeast)										
9841	ZBTB24	zinc finger and BTB domain containing 24										
9854	C2CD2L	C2CD2-like										
9857	CEP350	centrosomal protein 350kDa										
987	LRBA	LPS-responsive vesicle trafficking, beach and anchor containing										
9894	TELO2	TEL2, telomere maintenance 2, homolog (S. cerevisiae)										
9898	UBAP2L	ubiquitin associated protein 2-like										
9903	KLHL21	kelch-like 21 (Drosophila)										
9905	SGSM2	small G protein signaling modulator 2										
9909	DENND4B	DENN/MADD domain containing 4B										
9919	SEC16A	SEC16 homolog A (S. cerevisiae)										

Supplementary Table 6: JVM-2 Alternative Splicing MsigDB Analysis

Overlap Results												
Collection(s):	CP, CP:BIOCARTA, CP:KEGG, CP:REACTOME, H											
# overlaps shown:	10											
# genesets in collections:	1379											
# genes in comparison (n):	996											
# genes in universe (N):	45956											
Gene Set Name	# Genes in Gene Set (K)	Description	# Genes in Overlap (k)	k/K	p-value	FDR q-value						
REACTOME_CELL_CYCLE	421	Genes involved in Cell Cycle	39	0.0926	4.16E-14	2.87E-11						
REACTOME_IMMUNE_SYSTEM	933	Genes involved in Immune System	66	0.0707	6.95E-14	9.58E-11						
REACTOME_CELL_CYCLE_MITOTIC	325	Genes involved in Cell Cycle, Mitotic	32	0.0985	1.56E-12	7.18E-10						
HALLMARK_G2M_CHECKPOINT	200	Genes involved in the G2/M checkpoint, as in progression through the cell division cycle.	23	0.115	9.62E-11	3.32E-08						
REACTOME_ADAPTIVE_IMMUNE_SYSTEM	539	Genes involved in Adaptive Immune System	38	0.0705	3.08E-10	8.50E-08						
REACTOME_METABOLISM_OF_PROTEINS	518	Genes involved in Metabolism of proteins	36	0.0695	1.32E-09	3.04E-07						
HALLMARK_INTERFERON_GAMMA_RESPONSE	200	Genes up-regulated in response to IFNG [GeneID=3458].	21	0.105	3.29E-09	5.68E-07						
HALLMARK_MITOTIC_SPINDLE	200	Genes important for mitotic spindle assembly.	21	0.105	3.29E-09	5.68E-07						
KEGG_RIBOSOME	88	Ribosome	14	0.1591	6.70E-09	1.03E-06						
REACTOME_CELL_CYCLE_CHECKPOINTS	124	Genes involved in Cell Cycle Checkpoints	16	0.129	1.29E-08	1.78E-06						
Gene/Gene Set Overlap Matrix												
Entrez Gene Id	Gene Symbol	Gene Description	REACTOME_CELL_CYCLE	REACTOME_IMMUNE_SYSTEM	REACTOME_CELL_CYCLE_MITOTIC	HALLMARK_G2M_CHECKPOINT	REACTOME_ADAPTIVE_IMMUNE_SYSTEM	REACTOME_METABOLISM_OF_PROTEINS	HALLMARK_INTERFERON_GAMMA_RESPONSE	HALLMARK_MITOTIC_SPINDLE	KEGG_RIBOSOME	REACTOME_CELL_CYCLE_CHECKPOINTS
996	CDC27	cell division cycle 27 homolog (S. cerevisiae)	REACTOME_CELL_CYCLE	REACTOME_IMMUNE_SYSTEM	REACTOME_CELL_CYCLE_MITOTIC	HALLMARK_G2M_CHECKPOINT	REACTOME_ADAPTIVE_IMMUNE_SYSTEM			HALLMARK_MITOTIC_SPINDLE		REACTOME_CELL_CYCLE_CHECKPOINTS
11065	UBE2C	ubiquitin-conjugating enzyme E2C	REACTOME_CELL_CYCLE	REACTOME_IMMUNE_SYSTEM	REACTOME_CELL_CYCLE_MITOTIC	HALLMARK_G2M_CHECKPOINT	REACTOME_ADAPTIVE_IMMUNE_SYSTEM					REACTOME_CELL_CYCLE_CHECKPOINTS
7311	UBA52	ubiquitin A-52 residue ribosomal protein fusion product 1	REACTOME_CELL_CYCLE	REACTOME_IMMUNE_SYSTEM	REACTOME_CELL_CYCLE_MITOTIC		REACTOME_ADAPTIVE_IMMUNE_SYSTEM	REACTOME_METABOLISM_OF_PROTEINS			KEGG_RIBOSOME	REACTOME_CELL_CYCLE_CHECKPOINTS
5687	PSMA6	proteasome (prosome, macropain) subunit, alpha type, 6	REACTOME_CELL_CYCLE	REACTOME_IMMUNE_SYSTEM	REACTOME_CELL_CYCLE_MITOTIC		REACTOME_ADAPTIVE_IMMUNE_SYSTEM					REACTOME_CELL_CYCLE_CHECKPOINTS
5717	PSMD11	proteasome (prosome, macropain) 26S subunit, non-ATPase, 11	REACTOME_CELL_CYCLE	REACTOME_IMMUNE_SYSTEM	REACTOME_CELL_CYCLE_MITOTIC		REACTOME_ADAPTIVE_IMMUNE_SYSTEM					REACTOME_CELL_CYCLE_CHECKPOINTS
5710	PSMD4	proteasome (prosome, macropain) 26S subunit, non-ATPase, 4	REACTOME_CELL_CYCLE	REACTOME_IMMUNE_SYSTEM	REACTOME_CELL_CYCLE_MITOTIC		REACTOME_ADAPTIVE_IMMUNE_SYSTEM					REACTOME_CELL_CYCLE_CHECKPOINTS
7324	UBE2E1	ubiquitin-conjugating enzyme E2E 1	REACTOME_CELL_CYCLE	REACTOME_IMMUNE_SYSTEM	REACTOME_CELL_CYCLE_MITOTIC		REACTOME_ADAPTIVE_IMMUNE_SYSTEM					REACTOME_CELL_CYCLE_CHECKPOINTS
8881	CDC16	cell division cycle 16 homolog (S. cerevisiae)	REACTOME_CELL_CYCLE	REACTOME_IMMUNE_SYSTEM	REACTOME_CELL_CYCLE_MITOTIC		REACTOME_ADAPTIVE_IMMUNE_SYSTEM					REACTOME_CELL_CYCLE_CHECKPOINTS
5527	PPP2R5C	protein phosphatase 2, regulatory subunit B', gamma	REACTOME_CELL_CYCLE	REACTOME_IMMUNE_SYSTEM	REACTOME_CELL_CYCLE_MITOTIC		REACTOME_ADAPTIVE_IMMUNE_SYSTEM					
79902	NUP85	nucleoporin 85kDa	REACTOME_CELL_CYCLE	REACTOME_IMMUNE_SYSTEM	REACTOME_CELL_CYCLE_MITOTIC							
8243	SMC1A	structural maintenance of chromosomes 1A	REACTOME_CELL_CYCLE		REACTOME_CELL_CYCLE_MITOTIC	HALLMARK_G2M_CHECKPOINT				HALLMARK_MITOTIC_SPINDLE		
4926	NUMA1	nuclear mitotic apparatus protein 1	REACTOME_CELL_CYCLE		REACTOME_CELL_CYCLE_MITOTIC	HALLMARK_G2M_CHECKPOINT				HALLMARK_MITOTIC_SPINDLE		
4172	MCM3	minichromosome maintenance complex component 3	REACTOME_CELL_CYCLE		REACTOME_CELL_CYCLE_MITOTIC	HALLMARK_G2M_CHECKPOINT						REACTOME_CELL_CYCLE_CHECKPOINTS
7514	XPO1	exportin 1 (CRM1 homolog, yeast)	REACTOME_CELL_CYCLE		REACTOME_CELL_CYCLE_MITOTIC	HALLMARK_G2M_CHECKPOINT						
9735	KNTC1	kinetochore associated 1	REACTOME_CELL_CYCLE		REACTOME_CELL_CYCLE_MITOTIC					HALLMARK_MITOTIC_SPINDLE		
5116	PCNT	pericentrin	REACTOME_CELL_CYCLE		REACTOME_CELL_CYCLE_MITOTIC					HALLMARK_MITOTIC_SPINDLE		
55125	CEP192	centrosomal protein 192kDa	REACTOME_CELL_CYCLE		REACTOME_CELL_CYCLE_MITOTIC					HALLMARK_MITOTIC_SPINDLE		
55755	CDK5RAP2	CDK5 regulatory subunit associated protein 2	REACTOME_CELL_CYCLE		REACTOME_CELL_CYCLE_MITOTIC					HALLMARK_MITOTIC_SPINDLE		
701	BUB1B	budding uninhibited by benzimidazoles 1 homolog beta (yeast)	REACTOME_CELL_CYCLE		REACTOME_CELL_CYCLE_MITOTIC							REACTOME_CELL_CYCLE_CHECKPOINTS
898	CCNE1	cyclin E1	REACTOME_CELL_CYCLE		REACTOME_CELL_CYCLE_MITOTIC							REACTOME_CELL_CYCLE_CHECKPOINTS
84515	MCM8	minichromosome maintenance complex component 8	REACTOME_CELL_CYCLE		REACTOME_CELL_CYCLE_MITOTIC							REACTOME_CELL_CYCLE_CHECKPOINTS
9837	GINS1	GINS complex subunit 1 (Psf1 homolog)	REACTOME_CELL_CYCLE		REACTOME_CELL_CYCLE_MITOTIC							
5905	RANGAP1	Ran GTPase activating protein 1	REACTOME_CELL_CYCLE		REACTOME_CELL_CYCLE_MITOTIC							
10735	STAG2	stromal antigen 2	REACTOME_CELL_CYCLE		REACTOME_CELL_CYCLE_MITOTIC							
79003	MIS12	MIS12, MIND kinetochore complex component, homolog (S. pombe)	REACTOME_CELL_CYCLE		REACTOME_CELL_CYCLE_MITOTIC							
5422	POLA1	polymerase (DNA directed), alpha 1, catalytic subunit	REACTOME_CELL_CYCLE		REACTOME_CELL_CYCLE_MITOTIC							
4149	MAX	MYC associated factor X	REACTOME_CELL_CYCLE		REACTOME_CELL_CYCLE_MITOTIC							
5928	RBBP4	retinoblastoma binding protein 4	REACTOME_CELL_CYCLE		REACTOME_CELL_CYCLE_MITOTIC							
1859	DYRK1A	dual-specificity tyrosine-(Y)-phosphorylation regulated kinase 1A	REACTOME_CELL_CYCLE		REACTOME_CELL_CYCLE_MITOTIC							
22897	CEP164	centrosomal protein 164kDa	REACTOME_CELL_CYCLE		REACTOME_CELL_CYCLE_MITOTIC							
80321	CEP70	centrosomal protein 70kDa	REACTOME_CELL_CYCLE		REACTOME_CELL_CYCLE_MITOTIC							
8636	SSNA1	Sjogren syndrome nuclear autoantigen 1	REACTOME_CELL_CYCLE		REACTOME_CELL_CYCLE_MITOTIC							
5810	RAD1	RAD1 homolog (S. pombe)	REACTOME_CELL_CYCLE									REACTOME_CELL_CYCLE_CHECKPOINTS
11200	CHEK2	CHK2 checkpoint homolog (S. pombe)	REACTOME_CELL_CYCLE									REACTOME_CELL_CYCLE_CHECKPOINTS
5883	RAD9A	RAD9 homolog A (S. pombe)	REACTOME_CELL_CYCLE									REACTOME_CELL_CYCLE_CHECKPOINTS
472	ATM	ataxia telangiectasia mutated	REACTOME_CELL_CYCLE									REACTOME_CELL_CYCLE_CHECKPOINTS
7014	TERF2	telomeric repeat binding factor 2	REACTOME_CELL_CYCLE									
7013	TERF1	telomeric repeat binding factor (NIMA-interacting) 1	REACTOME_CELL_CYCLE									
23224	SYNE2	spectrin repeat containing, nuclear envelope 2	REACTOME_CELL_CYCLE									
29127	RACGAP1	Rac GTPase activating protein 1		REACTOME_IMMUNE_SYSTEM		HALLMARK_G2M_CHECKPOINT	REACTOME_ADAPTIVE_IMMUNE_SYSTEM			HALLMARK_MITOTIC_SPINDLE		
821	CANX	calnexin		REACTOME_IMMUNE_SYSTEM			REACTOME_ADAPTIVE_IMMUNE_SYSTEM	REACTOME_METABOLISM_OF_PROTEINS				
23480	SEC61G	Sec61 gamma subunit		REACTOME_IMMUNE_SYSTEM			REACTOME_ADAPTIVE_IMMUNE_SYSTEM	REACTOME_METABOLISM_OF_PROTEINS				
55294	FBXW7	F-box and WD repeat domain containing 7		REACTOME_IMMUNE_SYSTEM			REACTOME_ADAPTIVE_IMMUNE_SYSTEM	REACTOME_METABOLISM_OF_PROTEINS				
9632	SEC24C	SEC24 family, member C (S. cerevisiae)		REACTOME_IMMUNE_SYSTEM			REACTOME_ADAPTIVE_IMMUNE_SYSTEM	REACTOME_METABOLISM_OF_PROTEINS				
5777	PTPN6	protein tyrosine phosphatase, non-receptor type 6		REACTOME_IMMUNE_SYSTEM			REACTOME_ADAPTIVE_IMMUNE_SYSTEM		HALLMARK_INTERFERON_GAMMA_RESPONSE			
3108	HLA-DMA	major histocompatibility complex, class II, DM alpha		REACTOME_IMMUNE_SYSTEM			REACTOME_ADAPTIVE_IMMUNE_SYSTEM		HALLMARK_INTERFERON_GAMMA_RESPONSE			
3695	ITGB7	integrin, beta 7		REACTOME_IMMUNE_SYSTEM			REACTOME_ADAPTIVE_IMMUNE_SYSTEM		HALLMARK_INTERFERON_GAMMA_RESPONSE			
3831	KLC1	kinesin light chain 1		REACTOME_IMMUNE_SYSTEM			REACTOME_ADAPTIVE_IMMUNE_SYSTEM			HALLMARK_MITOTIC_SPINDLE		
253260	RICTOR	RPTOR independent companion of MTOR, complex 2		REACTOME_IMMUNE_SYSTEM			REACTOME_ADAPTIVE_IMMUNE_SYSTEM			HALLMARK_MITOTIC_SPINDLE		
7318	UBA7	ubiquitin-like modifier activating enzyme 7		REACTOME_IMMUNE_SYSTEM			REACTOME_ADAPTIVE_IMMUNE_SYSTEM					
23291	FBXW11	F-box and WD repeat domain containing 11		REACTOME_IMMUNE_SYSTEM			REACTOME_ADAPTIVE_IMMUNE_SYSTEM					
11059	WWP1	WW domain containing E3 ubiquitin protein ligase 1		REACTOME_IMMUNE_SYSTEM			REACTOME_ADAPTIVE_IMMUNE_SYSTEM					
868	CBLB	Cas-Br-M (murine) ecotropic retroviral transforming sequence b		REACTOME_IMMUNE_SYSTEM			REACTOME_ADAPTIVE_IMMUNE_SYSTEM					
4591	TRIM37	tripartite motif containing 37		REACTOME_IMMUNE_SYSTEM			REACTOME_ADAPTIVE_IMMUNE_SYSTEM					
9320	TRIP12	thyroid hormone receptor interactor 12		REACTOME_IMMUNE_SYSTEM			REACTOME_ADAPTIVE_IMMUNE_SYSTEM					
63891	RNF123	ring finger protein 123		REACTOME_IMMUNE_SYSTEM			REACTOME_ADAPTIVE_IMMUNE_SYSTEM					
10425	ARIH2	ariadne homolog 2 (Drosophila)		REACTOME_IMMUNE_SYSTEM			REACTOME_ADAPTIVE_IMMUNE_SYSTEM					
9520	NPEPPS	aminopeptidase puromycin sensitive		REACTOME_IMMUNE_SYSTEM			REACTOME_ADAPTIVE_IMMUNE_SYSTEM					
2534	FYN	FYN oncogene related to SRC, FGR, YES		REACTOME_IMMUNE_SYSTEM			REACTOME_ADAPTIVE_IMMUNE_SYSTEM					
3689	ITGB2	integrin, beta 2 (complement component 3 receptor 3 and 4 subunit)		REACTOME_IMMUNE_SYSTEM			REACTOME_ADAPTIVE_IMMUNE_SYSTEM					
808	CALM3	calmodulin 3 (phosphorylase kinase, delta)		REACTOME_IMMUNE_SYSTEM			REACTOME_ADAPTIVE_IMMUNE_SYSTEM					
5788	PTPRC	protein tyrosine phosphatase, receptor type, C		REACTOME_IMMUNE_SYSTEM			REACTOME_ADAPTIVE_IMMUNE_SYSTEM					
375	ARF1	ADP-ribosylation factor 1		REACTOME_IMMUNE_SYSTEM			REACTOME_ADAPTIVE_IMMUNE_SYSTEM					
163	AP2B1	adaptor-related protein complex 2, beta 1 subunit		REACTOME_IMMUNE_SYSTEM			REACTOME_ADAPTIVE_IMMUNE_SYSTEM					
8905	AP1S2	adaptor-related protein complex 1, sigma 2 subunit		REACTOME_IMMUNE_SYSTEM			REACTOME_ADAPTIVE_IMMUNE_SYSTEM					
973	CD79A	CD79a molecule, immunoglobulin-associated alpha		REACTOME_IMMUNE_SYSTEM			REACTOME_ADAPTIVE_IMMUNE_SYSTEM					
2213	FCGR2B	Fc fragment of IgG, low affinity IIb, receptor (CD32)		REACTOME_IMMUNE_SYSTEM			REACTOME_ADAPTIVE_IMMUNE_SYSTEM					
3821	KLRC1	killer cell lectin-like receptor subfamily C, member 1		REACTOME_IMMUNE_SYSTEM			REACTOME_ADAPTIVE_IMMUNE_SYSTEM					
3665	IRF7	interferon regulatory factor 7		REACTOME_IMMUNE_SYSTEM					HALLMARK_INTERFERON_GAMMA_RESPONSE			
6773	STAT2	signal transducer and activator of transcription 2, 113kDa		REACTOME_IMMUNE_SYSTEM					HALLMARK_INTERFERON_GAMMA_RESPONSE			
6772	STAT1	signal transducer and activator of transcription 1, 91kDa		REACTOME_IMMUNE_SYSTEM					HALLMARK_INTERFERON_GAMMA_RESPONSE			
834	CASP1	caspase 1, apoptosis-related cysteine peptidase (interleukin 1, beta, convertase)		REACTOME_IMMUNE_SYSTEM					HALLMARK_INTERFERON_GAMMA_RESPONSE			
5771	PTPN2	protein tyrosine phosphatase, non-receptor type 2		REACTOME_IMMUNE_SYSTEM					HALLMARK_INTERFERON_GAMMA_RESPONSE			
54739	XAF1	XIAP associated factor 1		REACTOME_IMMUNE_SYSTEM					HALLMARK_INTERFERON_GAMMA_RESPONSE			
7128	TNFAIP3	tumor necrosis factor, alpha-induced protein 3		REACTOME_IMMUNE_SYSTEM					HALLMARK_INTERFERON_GAMMA_RESPONSE			
23636	NUP62	nucleoporin 62kDa		REACTOME_IMMUNE_SYSTEM								
3082	HGF	hepatocyte growth factor (hepapoietin A; scatter factor)		REACTOME_IMMUNE_SYSTEM								
351	APP	amyloid beta (A4) precursor protein		REACTOME_IMMUNE_SYSTEM								
2185	PTK2B	PTK2B protein tyrosine kinase 2 beta		REACTOME_IMMUNE_SYSTEM								
960	CD44	CD44 molecule (Indian blood group)		REACTOME_IMMUNE_SYSTEM								
51447	IP6K2	inositol hexakisphosphate kinase 2		REACTOME_IMMUNE_SYSTEM								
6672	SP100	SP100 nuclear antigen		REACTOME_IMMUNE_SYSTEM								
51135	IRAK4	interleukin-1 receptor-associated kinase 4		REACTOME_IMMUNE_SYSTEM								
3575	IL7R	interleukin 7 receptor		REACTOME_IMMUNE_SYSTEM								
5094	PCBP2	poly(rC) binding protein 2		REACTOME_IMMUNE_SYSTEM								
84282	RNF135	ring finger protein 135		REACTOME_IMMUNE_SYSTEM								
9140	ATG12	ATG12 autophagy related 12 homolog (S. cerevisiae)		REACTOME_IMMUNE_SYSTEM								
8887	TAX1BP1	Tax1 (human T-cell leukemia virus type I) binding protein 1		REACTOME_IMMUNE_SYSTEM								
10010	TANK	TRAF family member-associated NFKB activator		REACTOME_IMMUNE_SYSTEM								
4205	MEF2A	myocyte enhancer factor 2A		REACTOME_IMMUNE_SYSTEM								
1386	ATF2	activating transcription factor 2		REACTOME_IMMUNE_SYSTEM								
7096	TLR1	toll-like receptor 1		REACTOME_IMMUNE_SYSTEM								
9450	LY86	lymphocyte antigen 86		REACTOME_IMMUNE_SYSTEM								
4179	CD46	CD46 molecule, complement regulatory protein		REACTOME_IMMUNE_SYSTEM								
22861	NLRP1	NLR family, pyrin domain containing 1		REACTOME_IMMUNE_SYSTEM								
9055	PRC1	protein regulator of cytokinesis 1				HALLMARK_G2M_CHECKPOINT				HALLMARK_MITOTIC_SPINDLE		
3925	STMN1	stathmin 1				HALLMARK_G2M_CHECKPOINT						
7112	TMPO	thymopoietin				HALLMARK_G2M_CHECKPOINT						
4957	ODF2	outer dense fiber of sperm tails 2				HALLMARK_G2M_CHECKPOINT						
387893	SETD8	SET domain containing (lysine methyltransferase) 8				HALLMARK_G2M_CHECKPOINT						
2130	EWSR1	Ewing sarcoma breakpoint region 1				HALLMARK_G2M_CHECKPOINT						
10772	SRSF10	serine/arginine-rich splicing factor 10				HALLMARK_G2M_CHECKPOINT						
8438	RAD54L	RAD54-like (S. cerevisiae)				HALLMARK_G2M_CHECKPOINT						
81539	SLC38A1	solute carrier family 38, member 1				HALLMARK_G2M_CHECKPOINT						
10024	TROAP	trophinin associated protein (tastin)				HALLMARK_G2M_CHECKPOINT						
10721	POLQ	polymerase (DNA directed), theta				HALLMARK_G2M_CHECKPOINT						
22823	MTF2	metal response element binding transcription factor 2				HALLMARK_G2M_CHECKPOINT						
3151	HMGN2	high mobility group nucleosomal binding domain 2				HALLMARK_G2M_CHECKPOINT						
4212	MEIS2	Meis homeobox 2				HALLMARK_G2M_CHECKPOINT						
6760	SS18	synovial sarcoma translocation, chromosome 18				HALLMARK_G2M_CHECKPOINT						
8899	PRPF4B	PRP4 pre-mRNA processing factor 4 homolog B (yeast)				HALLMARK_G2M_CHECKPOINT						
6223	RPS19	ribosomal protein S19						REACTOME_METABOLISM_OF_PROTEINS			KEGG_RIBOSOME	
6188	RPS3	ribosomal protein S3						REACTOME_METABOLISM_OF_PROTEINS			KEGG_RIBOSOME	
6204	RPS10	ribosomal protein S10						REACTOME_METABOLISM_OF_PROTEINS			KEGG_RIBOSOME	
6175	RPLP0	ribosomal protein, large, P0						REACTOME_METABOLISM_OF_PROTEINS			KEGG_RIBOSOME	
6201	RPS7	ribosomal protein S7						REACTOME_METABOLISM_OF_PROTEINS			KEGG_RIBOSOME	
6205	RPS11	ribosomal protein S11						REACTOME_METABOLISM_OF_PROTEINS			KEGG_RIBOSOME	
6234	RPS28	ribosomal protein S28						REACTOME_METABOLISM_OF_PROTEINS			KEGG_RIBOSOME	
6125	RPL5	ribosomal protein L5						REACTOME_METABOLISM_OF_PROTEINS			KEGG_RIBOSOME	
23521	RPL13A	ribosomal protein L13a						REACTOME_METABOLISM_OF_PROTEINS			KEGG_RIBOSOME	
6134	RPL10	ribosomal protein L10						REACTOME_METABOLISM_OF_PROTEINS			KEGG_RIBOSOME	
6139	RPL17	ribosomal protein L17						REACTOME_METABOLISM_OF_PROTEINS			KEGG_RIBOSOME	
6161	RPL32	ribosomal protein L32						REACTOME_METABOLISM_OF_PROTEINS			KEGG_RIBOSOME	
6171	RPL41	ribosomal protein L41						REACTOME_METABOLISM_OF_PROTEINS			KEGG_RIBOSOME	
6747	SSR3	signal sequence receptor, gamma (translocon-associated protein gamma)						REACTOME_METABOLISM_OF_PROTEINS				
6748	SSR4	signal sequence receptor, delta						REACTOME_METABOLISM_OF_PROTEINS				
3646	EIF3E	eukaryotic translation initiation factor 3, subunit E						REACTOME_METABOLISM_OF_PROTEINS				
27335	EIF3K	eukaryotic translation initiation factor 3, subunit K						REACTOME_METABOLISM_OF_PROTEINS				
10113	PREB	prolactin regulatory element binding						REACTOME_METABOLISM_OF_PROTEINS				
8813	DPM1	dolichyl-phosphate mannosyltransferase polypeptide 1, catalytic subunit						REACTOME_METABOLISM_OF_PROTEINS				
8703	B4GALT3	UDP-Gal:betaGlcNAc beta 1,4- galactosyltransferase, polypeptide 3						REACTOME_METABOLISM_OF_PROTEINS				
1798	DPAGT1	dolichyl-phosphate (UDP-N-acetylglucosamine) N-acetylglucosaminephosphotransferase 1 (GlcNAc-1-P transferase)						REACTOME_METABOLISM_OF_PROTEINS				
79868	ALG13	asparagine-linked glycosylation 13 homolog (S. cerevisiae)						REACTOME_METABOLISM_OF_PROTEINS				
7991	TUSC3	tumor suppressor candidate 3						REACTOME_METABOLISM_OF_PROTEINS				
9695	EDEM1	ER degradation enhancer, mannosidase alpha-like 1						REACTOME_METABOLISM_OF_PROTEINS				
285362	SUMF1	sulfatase modifying factor 1						REACTOME_METABOLISM_OF_PROTEINS				
10610	ST6GALNAC2	ST6 (alpha-N-acetyl-neuraminyl-2,3-beta-galactosyl-1,3)-N-acetylgalactosaminide alpha-2,6-sialyltransferase 2						REACTOME_METABOLISM_OF_PROTEINS				
80031	SEMA6D	sema domain, transmembrane domain (TM), and cytoplasmic domain, (semaphorin) 6D						REACTOME_METABOLISM_OF_PROTEINS				
51604	PIGT	phosphatidylinositol glycan anchor biosynthesis, class T						REACTOME_METABOLISM_OF_PROTEINS				
283463	MUC19	mucin 19, oligomeric						REACTOME_METABOLISM_OF_PROTEINS				
5204	PFDN5	prefoldin subunit 5						REACTOME_METABOLISM_OF_PROTEINS				
150274	HSCB	HscB iron-sulfur cluster co-chaperone homolog (E. coli)						REACTOME_METABOLISM_OF_PROTEINS				
10797	MTHFD2	methylenetetrahydrofolate dehydrogenase (NADP+ dependent) 2, methenyltetrahydrofolate cyclohydrolase							HALLMARK_INTERFERON_GAMMA_RESPONSE			
10964	IFI44L	interferon-induced protein 44-like							HALLMARK_INTERFERON_GAMMA_RESPONSE			
10561	IFI44	interferon-induced protein 44							HALLMARK_INTERFERON_GAMMA_RESPONSE			
219285	SAMD9L	sterile alpha motif domain containing 9-like							HALLMARK_INTERFERON_GAMMA_RESPONSE			
64761	PARP12	poly (ADP-ribose) polymerase family, member 12							HALLMARK_INTERFERON_GAMMA_RESPONSE			
7726	TRIM26	tripartite motif containing 26							HALLMARK_INTERFERON_GAMMA_RESPONSE			
6648	SOD2	superoxide dismutase 2, mitochondrial							HALLMARK_INTERFERON_GAMMA_RESPONSE			
3601	IL15RA	interleukin 15 receptor, alpha							HALLMARK_INTERFERON_GAMMA_RESPONSE			
3587	IL10RA	interleukin 10 receptor, alpha							HALLMARK_INTERFERON_GAMMA_RESPONSE			
10068	IL18BP	interleukin 18 binding protein							HALLMARK_INTERFERON_GAMMA_RESPONSE			
57823	SLAMF7	SLAM family member 7							HALLMARK_INTERFERON_GAMMA_RESPONSE			
667	DST	dystonin								HALLMARK_MITOTIC_SPINDLE		
324	APC	adenomatous polyposis coli								HALLMARK_MITOTIC_SPINDLE		
274	BIN1	bridging integrator 1								HALLMARK_MITOTIC_SPINDLE		
89941	RHOT2	ras homolog gene family, member T2								HALLMARK_MITOTIC_SPINDLE		
11214	AKAP13	A kinase (PRKA) anchor protein 13								HALLMARK_MITOTIC_SPINDLE		
4650	MYO9B	myosin IXB								HALLMARK_MITOTIC_SPINDLE		
6709	SPTAN1	spectrin, alpha, non-erythrocytic 1 (alpha-fodrin)								HALLMARK_MITOTIC_SPINDLE		
22930	RAB3GAP1	RAB3 GTPase activating protein subunit 1 (catalytic)								HALLMARK_MITOTIC_SPINDLE		
51174	TUBD1	tubulin, delta 1								HALLMARK_MITOTIC_SPINDLE		
5311	PKD2	polycystic kidney disease 2 (autosomal dominant)								HALLMARK_MITOTIC_SPINDLE		
5889	RAD51C	RAD51 homolog C (S. cerevisiae)										
6470	SHMT1	serine hydroxymethyltransferase 1 (soluble)										
51691	NAA38	N(alpha)-acetyltransferase 38, NatC auxiliary subunit										
4292	MLH1	mutL homolog 1, colon cancer, nonpolyposis type 2 (E. coli)										
29893	PSMC3IP	PSMC3 interacting protein										
6941	TCF19	transcription factor 19										
83461	CDCA3	cell division cycle associated 3										
9924	PAN2	PAN2 poly(A) specific ribonuclease subunit homolog (S. cerevisiae)										
5431	POLR2B	polymerase (RNA) II (DNA directed) polypeptide B, 140kDa										
23644	EDC4	enhancer of mRNA decapping 4										
80153	EDC3	enhancer of mRNA decapping 3 homolog (S. cerevisiae)										
6635	SNRPE	small nuclear ribonucleoprotein polypeptide E										
1207	CLNS1A	chloride channel, nucleotide-sensitive, 1A										
387	RHOA	ras homolog gene family, member A										
3675	ITGA3	integrin, alpha 3 (antigen CD49C, alpha 3 subunit of VLA-3 receptor)										
2771	GNAI2	guanine nucleotide binding protein (G protein), alpha inhibiting activity polypeptide 2										
146850	PIK3R6	phosphoinositide-3-kinase, regulatory subunit 6										
5478	PPIA	peptidylprolyl isomerase A (cyclophilin A)										
961	CD47	CD47 molecule										
27154	BRPF3	bromodomain and PHD finger containing, 3										
5153	PDE1B	phosphodiesterase 1B, calmodulin-dependent										
4602	MYB	v-myb myeloblastosis viral oncogene homolog (avian)										
22915	MMRN1	multimerin 1										
813	CALU	calumenin										
9162	DGKI	diacylglycerol kinase, iota										
7804	LRP8	low density lipoprotein receptor-related protein 8, apolipoprotein e receptor										
9057	SLC7A6	solute carrier family 7 (amino acid transporter light chain, y+L system), member 6										
10362	HMG20B	high mobility group 20B										
3046	HBE1	hemoglobin, epsilon 1										
55669	MFN1	mitofusin 1										
10242	KCNMB2	potassium large conductance calcium-activated channel, subfamily M, beta member 2										
6667	SP1	Sp1 transcription factor										
6198	RPS6KB1	ribosomal protein S6 kinase, 70kDa, polypeptide 1										
3183	HNRNPC	heterogeneous nuclear ribonucleoprotein C (C1/C2)										
3676	ITGA4	integrin, alpha 4 (antigen CD49D, alpha 4 subunit of VLA-4 receptor)										
905	CCNT2	cyclin T2										
6878	TAF6	TAF6 RNA polymerase II, TATA box binding protein (TBP)-associated factor, 80kDa										
6882	TAF11	TAF11 RNA polymerase II, TATA box binding protein (TBP)-associated factor, 28kDa										
2801	GOLGA2	golgin A2										
7178	TPT1	tumor protein, translationally-controlled 1										
2175	FANCA	Fanconi anemia, complementation group A										
2187	FANCB	Fanconi anemia, complementation group B										
22909	FAN1	FANCD2/FANCI-associated nuclease 1										
55215	FANCI	Fanconi anemia, complementation group I										
64421	DCLRE1C	DNA cross-link repair 1C										
9025	RNF8	ring finger protein 8										
85363	TRIM5	tripartite motif containing 5										
8550	MAPKAPK5	mitogen-activated protein kinase-activated protein kinase 5										
9448	MAP4K4	mitogen-activated protein kinase kinase kinase kinase 4										
5871	MAP4K2	mitogen-activated protein kinase kinase kinase kinase 2										
7786	MAP3K12	mitogen-activated protein kinase kinase kinase 12										
4968	OGG1	8-oxoguanine DNA glycosylase										
2319	FLOT2	flotillin 2										
4240	MFGE8	milk fat globule-EGF factor 8 protein										
26155	NOC2L	nucleolar complex associated 2 homolog (S. cerevisiae)										
9612	NCOR2	nuclear receptor corepressor 2										
79718	TBL1XR1	transducin (beta)-like 1 X-linked receptor 1										
5465	PPARA	peroxisome proliferator-activated receptor alpha										
9439	MED23	mediator complex subunit 23										
126382	NR2C2AP	nuclear receptor 2C2-associated protein										
10781	ZNF266	zinc finger protein 266										
147657	ZNF480	zinc finger protein 480										
147694	ZNF548	zinc finger protein 548										
163227	ZNF100	zinc finger protein 100										
284443	ZNF493	zinc finger protein 493										
348327	ZNF530	zinc finger protein 530										
51385	ZNF589	zinc finger protein 589										
57711	ZNF529	zinc finger protein 529										

7587	ZNF37A	zinc finger protein 37A										
7768	ZNF225	zinc finger protein 225										
79175	ZNF343	zinc finger protein 343										
79898	ZNF613	zinc finger protein 613										
81856	ZNF611	zinc finger protein 611										
5264	PHYH	phytanoyl-CoA 2-hydroxylase										
8443	GNPAT	glyceronephosphate O-acyltransferase										
26061	HACL1	2-hydroxyacyl-CoA lyase 1										
8310	ACOX3	acyl-CoA oxidase 3, pristanoyl										
55670	PEX26	peroxisomal biogenesis factor 26										
5828	PEX2	peroxisomal biogenesis factor 2										
5830	PEX5	peroxisomal biogenesis factor 5										
4358	MPV17	MpV17 mitochondrial inner membrane protein										
57448	BIRC6	baculoviral IAP repeat containing 6										
10914	PAPOLA	poly(A) polymerase alpha										
29894	CPSF1	cleavage and polyadenylation specific factor 1, 160kDa										
79869	CPSF7	cleavage and polyadenylation specific factor 7, 59kDa										
23451	SF3B1	splicing factor 3b, subunit 1, 155kDa										
3187	HNRNPH1	heterogeneous nuclear ribonucleoprotein H1 (H)										
1452	CSNK1A1	casein kinase 1, alpha 1										
27148	STK36	serine/threonine kinase 36										
8100	IFT88	intraflagellar transport 88 homolog (Chlamydomonas)										
3594	IL12RB1	interleukin 12 receptor, beta 1										
55540	IL17RB	interleukin 17 receptor B										
23765	IL17RA	interleukin 17 receptor A										
10645	CAMKK2	calcium/calmodulin-dependent protein kinase kinase 2, beta										
4528	MTIF2	mitochondrial translational initiation factor 2										
8886	DDX18	DEAD (Asp-Glu-Ala-Asp) box polypeptide 18										
3376	IARS	isoleucyl-tRNA synthetase										
52	ACP1	acid phosphatase 1, soluble										
8761	PABPC4	poly(A) binding protein, cytoplasmic 4 (inducible form)										
51181	DCXR	dicarbonyl/L-xylulose reductase										
50619	DEF6	differentially expressed in FDCP 6 homolog (mouse)										
23479	ISCU	iron-sulfur cluster scaffold homolog (E. coli)										
4033	LRMP	lymphoid-restricted membrane protein										
49856	WRAP73	WD repeat containing, antisense to TP73										
5820	PVT1	Pvt1 oncogene (non-protein coding)										
200576	PIKFYVE	phosphoinositide kinase, FYVE finger containing										
5305	PIP4K2A	phosphatidylinositol-5-phosphate 4-kinase, type II, alpha										
5287	PIK3C2B	phosphoinositide-3-kinase, class 2, beta polypeptide										
34	ACADM	acyl-CoA dehydrogenase, C-4 to C-12 straight chain										
9926	LPGAT1	lysophosphatidylglycerol acyltransferase 1										
81490	PTDSS2	phosphatidylserine synthase 2										
55627	SMPD4	sphingomyelin phosphodiesterase 4, neutral membrane (neutral sphingomyelinase-3)										
79644	SRD5A3	steroid 5 alpha-reductase 3										
6837	MED22	mediator complex subunit 22										
134429	STARD4	StAR-related lipid transfer (START) domain containing 4										
80765	STARD5	StAR-related lipid transfer (START) domain containing 5										
4597	MVD	mevalonate (diphospho) decarboxylase										
622	BDH1	3-hydroxybutyrate dehydrogenase, type 1										
6595	SMARCA2	SWI/SNF related, matrix associated, actin dependent regulator of chromatin, subfamily a, member 2										
144455	E2F7	E2F transcription factor 7										
3150	HMGN1	high mobility group nucleosome binding domain 1										
3682	ITGAE	integrin, alpha E (antigen CD103, human mucosal lymphocyte antigen 1; alpha polypeptide)										
1315	COPB1	coatomer protein complex, subunit beta 1										
10059	DNM1L	dynamin 1-like										
538	ATP7A	ATPase, Cu++ transporting, alpha polypeptide										
5066	PAM	peptidylglycine alpha-amidating monooxygenase										
8675	STX16	syntaxin 16										
23033	DOPEY1	dopey family member 1										
4839	NOP2	NOP2 nucleolar protein homolog (yeast)										
80324	PUS1	pseudouridylate synthase 1										
2193	FARSA	phenylalanyl-tRNA synthetase, alpha subunit										
10196	PRMT3	protein arginine methyltransferase 3										
23082	PPRC1	peroxisome proliferator-activated receptor gamma, coactivator-related 1										
81887	LAS1L	LAS1-like (S. cerevisiae)										
55276	PGM2	phosphoglucomutase 2										
5213	PFKM	phosphofructokinase, muscle										
6120	RPE	ribulose-5-phosphate-3-epimerase										
230	ALDOC	aldolase C, fructose-bisphosphate										
9563	H6PD	hexose-6-phosphate dehydrogenase (glucose 1-dehydrogenase)										
171568	POLR3H	polymerase (RNA) III (DNA directed) polypeptide H (22.9kD)										
30833	NT5C	5', 3'-nucleotidase, cytosolic										
22978	NT5C2	5'-nucleotidase, cytosolic II										
30834	ZNRD1	zinc ribbon domain containing 1										
4832	NME3	non-metastatic cells 3, protein expressed in										
953	ENTPD1	ectonucleoside triphosphate diphosphohydrolase 1										
54963	UCKL1	uridine-cytidine kinase 1-like 1										
6778	STAT6	signal transducer and activator of transcription 6, interleukin-4 induced										
123263	MTFMT	mitochondrial methionyl-tRNA formyltransferase										
275	AMT	aminomethyltransferase										
8567	MADD	MAP-kinase activating death domain										
23043	TNIK	TRAF2 and NCK interacting kinase										
9101	USP8	ubiquitin specific peptidase 8										
8942	KYNU	kynureninase										
9958	USP15	ubiquitin specific peptidase 15										
7511	XPNPEP1	X-prolyl aminopeptidase (aminopeptidase P) 1, soluble										
2323	FLT3LG	fms-related tyrosine kinase 3 ligand										
26018	LRIG1	leucine-rich repeats and immunoglobulin-like domains 1										
5025	P2RX4	purinergic receptor P2X, ligand-gated ion channel, 4										
22807	IKZF2	IKAROS family zinc finger 2 (Helios)										
285172	FAM126B	family with sequence similarity 126, member B										
57157	PHTF2	putative homeodomain transcription factor 2										
57231	SNX14	sorting nexin 14										
29082	CHMP4A	charged multivesicular body protein 4A										
22818	COPZ1	coatomer protein complex, subunit zeta 1										
8301	PICALM	phosphatidylinositol binding clathrin assembly protein										
2580	GAK	cyclin G associated kinase										
83933	HDAC10	histone deacetylase 10										
7181	NR2C1	nuclear receptor subfamily 2, group C, member 1										
1500	CTNND1	catenin (cadherin-associated protein), delta 1										
23265	EXOC7	exocyst complex component 7										
116985	ARAP1	ArfGAP with RhoGAP domain, ankyrin repeat and PH domain 1										
157	ADRBK2	adrenergic, beta, receptor kinase 2										
10617	STAMBP	STAM binding protein										
116986	AGAP2	ArfGAP with GTPase domain, ankyrin repeat and PH domain 2										
23527	ACAP2	ArfGAP with coiled-coil, ankyrin repeat and PH domains 2										
6621	SNAPC4	small nuclear RNA activating complex, polypeptide 4, 190kDa										
9013	TAF1C	TATA box binding protein (TBP)-associated factor, RNA polymerase I, C, 110kDa										
10919	EHMT2	euchromatic histone-lysine N-methyltransferase 2										
2976	GTF3C2	general transcription factor IIIC, polypeptide 2, beta 110kDa										
10325	RRAGB	Ras-related GTP binding B										
9776	ATG13	ATG13 autophagy related 13 homolog (S. cerevisiae)										
1716	DGUOK	deoxyguanosine kinase										
5150	PDE7A	phosphodiesterase 7A										
4913	NTHL1	nth endonuclease III-like 1 (E. coli)										
4595	MUTYH	mutY homolog (E. coli)										
55775	TDP1	tyrosyl-DNA phosphodiesterase 1										
55288	RHOT1	ras homolog gene family, member T1										
221472	FGD2	FYVE, RhoGEF and PH domain containing 2										
84986	ARHGAP19	Rho GTPase activating protein 19										
9748	SLK	STE20-like kinase										
3708	ITPR1	inositol 1,4,5-trisphosphate receptor, type 1										
10627	MYL12A	myosin, light chain 12A, regulatory, non-sarcomeric										
128239	IQGAP3	IQ motif containing GTPase activating protein 3										
1131	CHRM3	cholinergic receptor, muscarinic 3										
1730	DIAPH2	diaphanous homolog 2 (Drosophila)										
224	ALDH3A2	aldehyde dehydrogenase 3 family, member A2										
5364	PLXNB1	plexin B1										
2289	FKBP5	FK506 binding protein 5										
3547	IGSF1	immunoglobulin superfamily, member 1										
51599	LSR	lipolysis stimulated lipoprotein receptor										
8837	CFLAR	CASP8 and FADD-like apoptosis regulator										
22985	ACIN1	apoptotic chromatin condensation inducer 1										
2339	FNTA	farnesyltransferase, CAAX box, alpha										
10200	MPHOSPH6	M-phase phosphoprotein 6										
84186	ZCCHC7	zinc finger, CCHC domain containing 7										
4194	MDM4	Mdm4 p53 binding protein homolog (mouse)										
27327	TNRC6A	trinucleotide repeat containing 6A										
4242	MFNG	MFNG O-fucosylpeptide 3-beta-N-acetylglucosaminyltransferase										
56983	POGLUT1	protein O-glucosyltransferase 1										
833	CARS	cysteinyl-tRNA synthetase										
123283	TARSL2	threonyl-tRNA synthetase-like 2										
50615	IL21R	interleukin 21 receptor										
84498	FAM120B	family with sequence similarity 120B										
2208	FCER2	Fc fragment of IgE, low affinity II, receptor for (CD23)										
931	MS4A1	membrane-spanning 4-domains, subfamily A, member 1										
29102	DROSHA	drosha, ribonuclease type III										
3276	PRMT1	protein arginine methyltransferase 1										
57060	PCBP4	poly(rC) binding protein 4										
114049	WBSCR22	Williams Beuren syndrome chromosome region 22										
131965	METTL6	methyltransferase like 6										
60487	TRMT11	tRNA methyltransferase 11 homolog (S. cerevisiae)										
1600	DAB1	disabled homolog 1 (Drosophila)										
27032	ATP2C1	ATPase, Ca++ transporting, type 2C, member 1										
6310	ATXN1	ataxin 1										
4052	LTBP1	latent transforming growth factor beta binding protein 1										
10659	CELF2	CUGBP, Elav-like family member 2										
23469	PHF3	PHD finger protein 3										
26037	SIPA1L1	signal-induced proliferation-associated 1 like 1										
27109	ATP5S	ATP synthase, H+ transporting, mitochondrial Fo complex, subunit s (factor B)										
3420	IDH3B	isocitrate dehydrogenase 3 (NAD+) beta										
1349	COX7B	cytochrome c oxidase subunit VIIb										
522	ATP5J	ATP synthase, H+ transporting, mitochondrial Fo complex, subunit F6										
7388	UQCRH	ubiquinol-cytochrome c reductase hinge protein										
2108	ETFA	electron-transfer-flavoprotein, alpha polypeptide										
9617	MTRF1	mitochondrial translational release factor 1										
5831	PYCR1	pyrroline-5-carboxylate reductase 1										
5723	PSPH	phosphoserine phosphatase										
2744	GLS	glutaminase										
26275	HIBCH	3-hydroxyisobutyryl-CoA hydrolase										
9380	GRHPR	glyoxylate reductase/hydroxypyruvate reductase										
55748	CNDP2	CNDP dipeptidase 2 (metallopeptidase M20 family)										
4776	NFATC4	nuclear factor of activated T-cells, cytoplasmic, calcineurin-dependent 4										
10725	NFAT5	nuclear factor of activated T-cells 5, tonicity-responsive										
80326	WNT10A	wingless-type MMTV integration site family, member 10A										
10396	ATP8A1	ATPase, aminophospholipid transporter (APLT), class I, type 8A, member 1										
374868	ATP9B	ATPase, class II, type 9B										
5261	PHKG2	phosphorylase kinase, gamma 2 (testis)										
10039	PARP3	poly (ADP-ribose) polymerase family, member 3										
1429	CRYZ	crystallin, zeta (quinone reductase)										
7391	USF1	upstream transcription factor 1										
6867	TACC1	transforming, acidic coiled-coil containing protein 1										
23461	ABCA5	ATP-binding cassette, sub-family A (ABC1), member 5										
6789	STK4	serine/threonine kinase 4										
6788	STK3	serine/threonine kinase 3										
261734	NPHP4	nephronophthisis 4										
55586	MIOX	myo-inositol oxygenase										
3069	HDLBP	high density lipoprotein binding protein										
55139	ANKZF1	ankyrin repeat and zinc finger domain containing 1										
57018	CCNL1	cyclin L1										
53	ACP2	acid phosphatase 2, lysosomal										
115817	DHRS1	dehydrogenase/reductase (SDR family) member 1										
6925	TCF4	transcription factor 4										
8506	CNTNAP1	contactin associated protein 1										
6326	SCN2A	sodium channel, voltage-gated, type II, alpha subunit										
65220	NADK	NAD kinase										
11231	SEC63	SEC63 homolog (S. cerevisiae)										
196294	IMMP1L	IMP1 inner mitochondrial membrane peptidase-like (S. cerevisiae)										
4796	TONSL	tonsoku-like, DNA repair protein										
51512	GTSE1	G-2 and S-phase expressed 1										
3795	KHK	ketohexokinase (fructokinase)										
7919	DDX39B	DEAD (Asp-Glu-Ala-Asp) box polypeptide 39B										
79717	PPCS	phosphopantothenoylcysteine synthetase										
2356	FPGS	folylpolyglutamate synthase										
10434	LYPLA1	lysophospholipase I										
5783	PTPN13	protein tyrosine phosphatase, non-receptor type 13 (APO-1/CD95 (Fas)-associated phosphatase)										
29843	SENP1	SUMO1/sentrin specific peptidase 1										
11235	PDCD10	programmed cell death 10										
3297	HSF1	heat shock transcription factor 1										
535	ATP6V0A1	ATPase, H+ transporting, lysosomal V0 subunit a1										
56180	MOSPD1	motile sperm domain containing 1										
6397	SEC14L1	SEC14-like 1 (S. cerevisiae)										
80148	PQLC1	PQ loop repeat containing 1										
8273	SLC10A3	solute carrier family 10 (sodium/bile acid cotransporter family), member 3										
4481	MSR1	macrophage scavenger receptor 1										
5536	PPP5C	protein phosphatase 5, catalytic subunit										
11315	PARK7	parkinson protein 7										
1268	CNR1	cannabinoid receptor 1 (brain)										
9709	HERPUD1	homocysteine-inducible, endoplasmic reticulum stress-inducible, ubiquitin-like domain member 1										
57107	PDSS2	prenyl (decaprenyl) diphosphate synthase, subunit 2										
10910	SUGT1	SGT1, suppressor of G2 allele of SKP1 (S. cerevisiae)										
4660	PPP1R12B	protein phosphatase 1, regulatory subunit 12B										
4126	MANBA	mannosidase, beta A, lysosomal										
4123	MAN2C1	mannosidase, alpha, class 2C, member 1										
11284	PNKP	polynucleotide kinase 3'-phosphatase										
9463	PICK1	protein interacting with PRKCA 1										
23163	GGA3	golgi-associated, gamma adaptin ear containing, ARF binding protein 3										
7750	ZMYM2	zinc finger, MYM-type 2										
6801	STRN	striatin, calmodulin binding protein										
60401	EDA2R	ectodysplasin A2 receptor										
1663	DDX11	DEAD/H (Asp-Glu-Ala-Asp/His) box polypeptide 11										
4644	MYO5A	myosin VA (heavy chain 12, myoxin)										
590	BCHE	butyrylcholinesterase										
11194	ABCB8	ATP-binding cassette, sub-family B (MDR/TAP), member 8										
84895	FAM73B	family with sequence similarity 73, member B										
8751	ADAM15	ADAM metallopeptidase domain 15										
5310	PKD1	polycystic kidney disease 1 (autosomal dominant)										
80895	ILKAP	integrin-linked kinase-associated serine/threonine phosphatase										
1390	CREM	cAMP responsive element modulator										
2635	GBP3	guanylate binding protein 3										
55066	PDPR	pyruvate dehydrogenase phosphatase regulatory subunit										
6602	SMARCD1	SWI/SNF related, matrix associated, actin dependent regulator of chromatin, subfamily d, member 1										
11279	KLF8	Kruppel-like factor 8										
5396	PRRX1	paired related homeobox 1										
5936	RBM4	RNA binding motif protein 4										
23081	KDM4C	lysine (K)-specific demethylase 4C										
3782	KCNN3	potassium intermediate/small conductance calcium-activated channel, subfamily N, member 3										
84458	LCOR	ligand dependent nuclear receptor corepressor										
29121	CLEC2D	C-type lectin domain family 2, member D										
309	ANXA6	annexin A6										
3965	LGALS9	lectin, galactoside-binding, soluble, 9										
55743	CHFR	checkpoint with forkhead and ring finger domains										
8028	MLLT10	myeloid/lymphoid or mixed-lineage leukemia (trithorax homolog, Drosophila); translocated to, 10										
27334	P2RY10	purinergic receptor P2Y, G-protein coupled, 10										
10911	UTS2	urotensin 2										
203522	DDX26B	DEAD/H (Asp-Glu-Ala-Asp/His) box polypeptide 26B										
22932	POMZP3	POM121 and ZP3 fusion										

1310	COL19A1	collagen, type XIX, alpha 1										
6738	TROVE2	TROVE domain family, member 2										
9628	RGS6	regulator of G-protein signaling 6										
11174	ADAMTS6	ADAM metallopeptidase with thrombospondin type 1 motif, 6										
53616	ADAM22	ADAM metallopeptidase domain 22										
81792	ADAMTS12	ADAM metallopeptidase with thrombospondin type 1 motif, 12										
1954	MEGF8	multiple EGF-like-domains 8										
2329	FMO4	flavin containing monooxygenase 4										
1787	TRDMT1	tRNA aspartic acid methyltransferase 1										
23623	RUSC1	RUN and SH3 domain containing 1										
444	ASPH	aspartate beta-hydroxylase										
7155	TOP2B	topoisomerase (DNA) II beta 180kDa										
100093630	SNHG8	small nucleolar RNA host gene 8 (non-protein coding)										
100129482	ZNF37BP	zinc finger protein 37B, pseudogene										
10049	DNAJB6	DnaJ (Hsp40) homolog, subfamily B, member 6										
10101	NUBP2	nucleotide binding protein 2										
10138	YAF2	YY1 associated factor 2										
10150	MBNL2	muscleblind-like 2 (Drosophila)										
10208	USPL1	ubiquitin specific peptidase like 1										
10227	MFSD10	major facilitator superfamily domain containing 10										
10233	LRRC23	leucine rich repeat containing 23										
10295	BCKDK	branched chain ketoacid dehydrogenase kinase										
10307	APBB3	amyloid beta (A4) precursor protein-binding, family B, member 3										
10329	TMEM5	transmembrane protein 5										
10336	PCGF3	polycomb group ring finger 3										
10346	TRIM22	tripartite motif containing 22										
10384	BTN3A3	butyrophilin, subfamily 3, member A3										
10385	BTN2A2	butyrophilin, subfamily 2, member A2										
10495	ENOX2	ecto-NOX disulfide-thiol exchanger 2										
10585	POMT1	protein-O-mannosyltransferase 1										
10613	ERLIN1	ER lipid raft associated 1										
10625	IVNS1ABP	influenza virus NS1A binding protein										
10641	NPRL2	nitrogen permease regulator-like 2 (S. cerevisiae)										
10658	CELF1	CUGBP, Elav-like family member 1										
10730	YME1L1	YME1-like 1 (S. cerevisiae)										
10783	NEK6	NIMA (never in mitosis gene a)-related kinase 6										
10807	SDCCAG3	serologically defined colon cancer antigen 3										
10867	TSPAN9	tetraspanin 9										
10923	SUB1	SUB1 homolog (S. cerevisiae)										
10927	SPIN1	spindlin 1										
1102	RCBTB2	regulator of chromosome condensation (RCC1) and BTB (POZ) domain containing protein 2										
11101	ATE1	arginyltransferase 1										
11118	BTN3A2	butyrophilin, subfamily 3, member A2										
11119	BTN3A1	butyrophilin, subfamily 3, member A1										
1121	CHM	choroideremia (Rab escort protein 1)										
11212	PROSC	proline synthetase co-transcribed homolog (bacterial)										
112611	RWDD2A	RWD domain containing 2A										
11262	SP140	SP140 nuclear body protein										
112752	IFT43	intraflagellar transport 43 homolog (Chlamydomonas)										
113177	IZUMO4	IZUMO family member 4										
11322	TMC6	transmembrane channel-like 6										
113402	SFT2D1	SFT2 domain containing 1										
114614	MIR155HG	MIR155 host gene (non-protein coding)										
114800	CCDC85A	coiled-coil domain containing 85A										
114803	MYSM1	Myb-like, SWIRM and MPN domains 1										
119504	ANAPC16	anaphase promoting complex subunit 16										
121273	C12orf54	chromosome 12 open reading frame 54										
122704	MRPL52	mitochondrial ribosomal protein L52										
122786	FRMD6	FERM domain containing 6										
123606	NIPA1	non imprinted in Prader-Willi/Angelman syndrome 1										
124093	CCDC78	coiled-coil domain containing 78										
125150	ZSWIM7	zinc finger, SWIM-type containing 7										
126075	CCDC159	coiled-coil domain containing 159										
126133	ALDH16A1	aldehyde dehydrogenase 16 family, member A1										
126526	C19orf47	chromosome 19 open reading frame 47										
128308	MRPL55	mitochondrial ribosomal protein L55										
129563	DIS3L2	DIS3 mitotic control homolog (S. cerevisiae)-like 2										
130872	AHSA2	AHA1, activator of heat shock 90kDa protein ATPase homolog 2 (yeast)										
133015	PACRGL	PARK2 co-regulated-like										
134637	ADAT2	adenosine deaminase, tRNA-specific 2										
135114	HINT3	histidine triad nucleotide binding protein 3										
137695	TMEM68	transmembrane protein 68										
138241	C9orf85	chromosome 9 open reading frame 85										
1388	ATF6B	activating transcription factor 6 beta										
1396	CRIP1	cysteine-rich protein 1 (intestinal)										
140469	MYO3B	myosin IIIB										
140733	MACROD2	MACRO domain containing 2										
140890	SREK1	splicing regulatory glutamine/lysine-rich protein 1										
143162	FRMPD2	FERM and PDZ domain containing 2										
146206	RLTPR	RGD motif, leucine rich repeats, tropomodulin domain and proline-rich containing										
146857	SLFN13	schlafen family member 13										
147172	LRRC37BP1	leucine rich repeat containing 37B pseudogene 1										
147991	DPY19L3	dpy-19-like 3 (C. elegans)										
150465	TTL	tubulin tyrosine ligase										
150468	CKAP2L	cytoskeleton associated protein 2-like										
150472	CBWD2	COBW domain containing 2										
150962	PUS10	pseudouridylate synthase 10										
152815	THAP6	THAP domain containing 6										
153241	CEP120	centrosomal protein 120kDa										
155400	NSUN5P1	NOP2/Sun domain family, member 5 pseudogene 1										
157574	FBXO16	F-box protein 16										
158056	MAMDC4	MAM domain containing 4										
158427	TSTD2	thiosulfate sulfurtransferase (rhodanese)-like domain containing 2										
158431	ZNF782	zinc finger protein 782										
158511	CSAG1	chondrosarcoma associated gene 1										
160760	PPTC7	PTC7 protein phosphatase homolog (S. cerevisiae)										
162282	ANKFN1	ankyrin-repeat and fibronectin type III domain containing 1										
162427	FAM134C	family with sequence similarity 134, member C										
165631	PARP15	poly (ADP-ribose) polymerase family, member 15										
166824	RASSF6	Ras association (RalGDS/AF-6) domain family member 6										
167153	PAPD4	PAP associated domain containing 4										
170371	C10orf128	chromosome 10 open reading frame 128										
170958	ZNF525	zinc finger protein 525										
171483	FAM9B	family with sequence similarity 9, member B										
172	AFG3L1P	AFG3 ATPase family gene 3-like 1 (S. cerevisiae), pseudogene										
1770	DNAH9	dynein, axonemal, heavy chain 9										
1912	PHC2	polyhomeotic homolog 2 (Drosophila)										
192683	SCAMP5	secretory carrier membrane protein 5										
197342	EME2	essential meiotic endonuclease 1 homolog 2 (S. pombe)										
200030	NBPF11	neuroblastoma breakpoint family, member 11										
201294	UNC13D	unc-13 homolog D (C. elegans)										
203054	ADCK5	aarF domain containing kinase 5										
2140	EYA3	eyes absent homolog 3 (Drosophila)										
2186	BPTF	bromodomain PHD finger transcription factor										
220869	CBWD5	COBW domain containing 5										
2218	FKTN	fukutin										
221927	BRAT1	BRCA1-associated ATM activator 1										
222234	FAM185A	family with sequence similarity 185, member A										
222235	FBXL13	F-box and leucine-rich repeat protein 13										
222658	KCTD20	potassium channel tetramerisation domain containing 20										
22880	MORC2	MORC family CW-type zinc finger 2										
22890	ZBTB1	zinc finger and BTB domain containing 1										
22902	RUFY3	RUN and FYVE domain containing 3										
22913	RALY	RNA binding protein, autoantigenic (hnRNP-associated with lethal yellow homolog (mouse))										
22931	RAB18	RAB18, member RAS oncogene family										
23023	TMCC1	transmembrane and coiled-coil domain family 1										
23077	MYCBP2	MYC binding protein 2										
23087	TRIM35	tripartite motif containing 35										
23107	MRPS27	mitochondrial ribosomal protein S27										
23142	DCUN1D4	DCN1, defective in cullin neddylation 1, domain containing 4 (S. cerevisiae)										
23155	CLCC1	chloride channel CLIC-like 1										
23179	RGL1	ral guanine nucleotide dissociation stimulator-like 1										
23214	XPO6	exportin 6										
23215	PRRC2C	proline-rich coiled-coil 2C										
23230	VPS13A	vacuolar protein sorting 13 homolog A (S. cerevisiae)										
23262	PPIP5K2	diphosphoinositol pentakisphosphate kinase 2										
23301	EHBP1	EH domain binding protein 1										
23353	SUN1	Sad1 and UNC84 domain containing 1										
23355	VPS8	vacuolar protein sorting 8 homolog (S. cerevisiae)										
23367	LARP1	La ribonucleoprotein domain family, member 1										
23383	MAU2	MAU2 chromatid cohesion factor homolog (C. elegans)										
23399	CTDNEP1	CTD nuclear envelope phosphatase 1										
23412	COMMD3	COMM domain containing 3										
23473	CAPN7	calpain 7										
23513	SCRIB	scribbled homolog (Drosophila)										
23534	TNPO3	transportin 3										
23554	TSPAN12	tetraspanin 12										
23559	WBP1	WW domain binding protein 1										
23567	ZNF346	zinc finger protein 346										
23613	ZMYND8	zinc finger, MYND-type containing 8										
23642	SNHG1	small nucleolar RNA host gene 1 (non-protein coding)										
23670	TMEM2	transmembrane protein 2										
23787	MTCH1	mitochondrial carrier 1										
24139	EML2	echinoderm microtubule associated protein like 2										
245	ALOX12P2	arachidonate 12-lipoxygenase pseudogene 2										
253461	ZBTB38	zinc finger and BTB domain containing 38										
253832	ZDHHC20	zinc finger, DHHC-type containing 20										
254251	LCORL	ligand dependent nuclear receptor corepressor-like										
254295	PHYHD1	phytanoyl-CoA dioxygenase domain containing 1										
2551	GABPA	GA binding protein transcription factor, alpha subunit 60kDa										
255104	TMCO4	transmembrane and coiled-coil domains 4										
255252	LRRC57	leucine rich repeat containing 57										
255812	SDHAP1	succinate dehydrogenase complex, subunit A, flavoprotein pseudogene 1										
257160	RNF214	ring finger protein 214										
25792	CIZ1	CDKN1A interacting zinc finger protein 1										
25864	ABHD14A	abhydrolase domain containing 14A										
25875	LETMD1	LETM1 domain containing 1										
25912	C1orf43	chromosome 1 open reading frame 43										
25934	NIPSNAP3A	nipsnap homolog 3A (C. elegans)										
25957	PNISR	PNN-interacting serine/arginine-rich protein										
25959	KANK2	KN motif and ankyrin repeat domains 2										
26001	RNF167	ring finger protein 167										
26049	FAM169A	family with sequence similarity 169, member A										
26137	ZBTB20	zinc finger and BTB domain containing 20										
26151	NAT9	N-acetyltransferase 9 (GCN5-related, putative)										
26191	PTPN22	protein tyrosine phosphatase, non-receptor type 22 (lymphoid)										
2622	GAS8	growth arrest-specific 8										
26233	FBXL6	F-box and leucine-rich repeat protein 6										
2630	GBAP1	glucosidase, beta, acid pseudogene 1										
26751	SH3YL1	SH3 domain containing, Ysc84-like 1 (S. cerevisiae)										
26960	NBEA	neurobeachin										
26993	AKAP8L	A kinase (PRKA) anchor protein 8-like										
27020	NPTN	neuroplastin										
27136	MORC1	MORC family CW-type zinc finger 1										
27141	CIDEB	cell death-inducing DFFA-like effector b										
27241	BBS9	Bardet-Biedl syndrome 9										
27315	PGAP2	post-GPI attachment to proteins 2										
27352	SGSM3	small G protein signaling modulator 3										
284349	ZNF283	zinc finger protein 283										
284371	ZNF841	zinc finger protein 841										
284403	WDR62	WD repeat domain 62										
284565	NBPF15	neuroblastoma breakpoint family, member 15										
285989	ZNF789	zinc finger protein 789										
286257	C9orf142	chromosome 9 open reading frame 142										
2874	GPS2	G protein pathway suppressor 2										
28957	MRPS28	mitochondrial ribosomal protein S28										
29066	ZC3H7A	zinc finger CCCH-type containing 7A										
29068	ZBTB44	zinc finger and BTB domain containing 44										
29087	THYN1	thymocyte nuclear protein 1										
29093	MRPL22	mitochondrial ribosomal protein L22										
29098	RANGRF	RAN guanine nucleotide release factor										
29109	FHOD1	formin homology 2 domain containing 1										
29115	SAP30BP	SAP30 binding protein										
29123	ANKRD11	ankyrin repeat domain 11										
2975	GTF3C1	general transcription factor IIIC, polypeptide 1, alpha 220kDa										
29911	HOOK2	hook homolog 2 (Drosophila)										
29960	FTSJ2	FtsJ homolog 2 (E. coli)										
29966	STRN3	striatin, calmodulin binding protein 3										
3104	ZBTB48	zinc finger and BTB domain containing 48										
317781	DDX51	DEAD (Asp-Glu-Ala-Asp) box polypeptide 51										
3268	AGFG2	ArfGAP with FG repeats 2										
327	APEH	N-acylaminoacyl-peptide hydrolase										
3338	DNAJC4	DnaJ (Hsp40) homolog, subfamily C, member 4										
338440	ANO9	anoctamin 9										
338657	CCDC84	coiled-coil domain containing 84										
339318	ZNF181	zinc finger protein 181										
339487	ZBTB8OS	zinc finger and BTB domain containing 8 opposite strand										
346389	MACC1	metastasis associated in colon cancer 1										
347240	KIF24	kinesin family member 24										
348094	ANKDD1A	ankyrin repeat and death domain containing 1A										
348645	C22orf34	chromosome 22 open reading frame 34										
348654	GEN1	Gen homolog 1, endonuclease (Drosophila)										
349152	DPY19L2P2	dpy-19-like 2 pseudogene 2 (C. elegans)										
359948	IRF2BP2	interferon regulatory factor 2 binding protein 2										
374618	TEX9	testis expressed 9										
374666	WASH3P	WAS protein family homolog 3 pseudogene										
375444	C5orf34	chromosome 5 open reading frame 34										
378938	MALAT1	metastasis associated lung adenocarcinoma transcript 1 (non-protein coding)										
386593	CHKB-CPT1B	CHKB-CPT1B readthrough (non-protein coding)										
387036	GUSBP2	glucuronidase, beta pseudogene 2										
387103	CENPW	centromere protein W										
388558	ZNF808	zinc finger protein 808										
399979	SNX19	sorting nexin 19										
400713	ZNF880	zinc finger protein 880										
400818	NBPF9	neuroblastoma breakpoint family, member 9										
4034	LRCH4	leucine-rich repeats and calponin homology (CH) domain containing 4										
4152	MBD1	methyl-CpG binding domain protein 1										
4253	CTAGE5	CTAGE family, member 5										
4285	MIPEP	mitochondrial intermediate peptidase										
4287	ATXN3	ataxin 3										
441194	PMS2CL	PMS2 C-terminal like pseudogene										
4649	MYO9A	myosin IXA										
4664	NAB1	NGFI-A binding protein 1 (EGR1 binding protein 1)										
4676	NAP1L4	nucleosome assembly protein 1-like 4										
4798	NFRKB	nuclear factor related to kappaB binding protein										
4820	NKTR	natural killer-tumor recognition sequence										
4925	NUCB2	nucleobindin 2										
494143	CHAC2	ChaC, cation transport regulator homolog 2 (E. coli)										
503538	A1BG-AS1	A1BG antisense RNA 1 (non-protein coding)										
50717	DCAF8	DDB1 and CUL4 associated factor 8										
5090	PBX3	pre-B-cell leukemia homeobox 3										
51000	SLC35B3	solute carrier family 35, member B3										
51011	FAHD2A	fumarylacetoacetate hydrolase domain containing 2A										
51095	TRNT1	tRNA nucleotidyl transferase, CCA-adding, 1										
51228	GLTP	glycolipid transfer protein										
51249	TMEM69	transmembrane protein 69										
51255	RNF181	ring finger protein 181										
51304	ZDHHC3	zinc finger, DHHC-type containing 3										

51329	ARL6IP4	ADP-ribosylation-like factor 6 interacting protein 4										
51397	COMMD10	COMM domain containing 10										
51522	TMEM14C	transmembrane protein 14C										
51555	PEX5L	peroxisomal biogenesis factor 5-like										
51654	CDK5RAP1	CDK5 regulatory subunit associated protein 1										
51657	STYXL1	serine/threonine/tyrosine interacting-like 1										
51661	FKBP7	FK506 binding protein 7										
51678	MPP6	membrane protein, palmitoylated 6 (MAGUK p55 subfamily member 6)										
5379	PMS2P1	postmeiotic segregation increased 2 pseudogene 1										
53917	RAB24	RAB24, member RAS oncogene family										
54058	C21orf58	chromosome 21 open reading frame 58										
54476	RNF216	ring finger protein 216										
54496	PRMT7	protein arginine methyltransferase 7										
54499	TMCO1	transmembrane and coiled-coil domains 1										
54502	RBM47	RNA binding motif protein 47										
54552	GNL3L	guanine nucleotide binding protein-like 3 (nucleolar)-like										
54619	CCNJ	cyclin J										
54620	FBXL19	F-box and leucine-rich repeat protein 19										
54758	KLHDC4	kelch domain containing 4										
54808	DYM	dymeclin										
54811	ZNF562	zinc finger protein 562										
54814	QPCTL	glutaminyl-peptide cyclotransferase-like										
54851	ANKRD49	ankyrin repeat domain 49										
54879	ST7L	suppression of tumorigenicity 7 like										
54900	LAX1	lymphocyte transmembrane adaptor 1										
54929	TMEM161A	transmembrane protein 161A										
54952	TRNAU1AP	tRNA selenocysteine 1 associated protein 1										
55005	RMND1	required for meiotic nuclear division 1 homolog (S. cerevisiae)										
55007	FAM118A	family with sequence similarity 118, member A										
55041	PLEKHB2	pleckstrin homology domain containing, family B (evectins) member 2										
55052	MRPL20	mitochondrial ribosomal protein L20										
55074	OXR1	oxidation resistance 1										
55080	TAPBPL	TAP binding protein-like										
55095	SAMD4B	sterile alpha motif domain containing 4B										
55133	SRBD1	S1 RNA binding domain 1										
55161	TMEM33	transmembrane protein 33										
55168	MRPS18A	mitochondrial ribosomal protein S18A										
55183	RIF1	RAP1 interacting factor homolog (yeast)										
55186	SLC25A36	solute carrier family 25, member 36										
55187	VPS13D	vacuolar protein sorting 13 homolog D (S. cerevisiae)										
55193	PBRM1	polybromo 1										
55205	ZNF532	zinc finger protein 532										
55206	SBNO1	strawberry notch homolog 1 (Drosophila)										
55209	SETD5	SET domain containing 5										
55213	RCBTB1	regulator of chromosome condensation (RCC1) and BTB (POZ) domain containing protein 1										
55268	ECHDC2	enoyl CoA hydratase domain containing 2										
55280	CWF19L1	CWF19-like 1, cell cycle control (S. pombe)										
55291	PPP6R3	protein phosphatase 6, regulatory subunit 3										
55297	CCDC91	coiled-coil domain containing 91										
55322	C5orf22	chromosome 5 open reading frame 22										
55536	CDCA7L	cell division cycle associated 7-like										
55552	ZNF823	zinc finger protein 823										
55565	ZNF821	zinc finger protein 821										
55591	VEZT	vezatin, adherens junctions transmembrane protein										
55626	AMBRA1	autophagy/beclin-1 regulator 1										
55656	INTS8	integrator complex subunit 8										
55676	SLC30A6	solute carrier family 30 (zinc transporter), member 6										
55687	TRMU	tRNA 5-methylaminomethyl-2-thiouridylate methyltransferase										
55689	YEATS2	YEATS domain containing 2										
55700	MAP7D1	MAP7 domain containing 1										
55717	WDR11	WD repeat domain 11										
55739	CARKD	carbohydrate kinase domain containing										
55769	ZNF83	zinc finger protein 83										
55813	UTP6	UTP6, small subunit (SSU) processome component, homolog (yeast)										
55814	BDP1	B double prime 1, subunit of RNA polymerase III transcription initiation factor IIIB										
55862	ECHDC1	enoyl CoA hydratase domain containing 1										
55972	SLC25A40	solute carrier family 25, member 40										
5612	PRKRIR	protein-kinase, interferon-inducible double stranded RNA dependent inhibitor, repressor of (P58 repressor)										
56943	ENY2	enhancer of yellow 2 homolog (Drosophila)										
56948	SDR39U1	short chain dehydrogenase/reductase family 39U, member 1										
57147	SCYL3	SCY1-like 3 (S. cerevisiae)										
57149	LYRM1	LYR motif containing 1										
57223	SMEK2	SMEK homolog 2, suppressor of mek1 (Dictyostelium)										
57396	CLK4	CDC-like kinase 4										
57531	HACE1	HECT domain and ankyrin repeat containing, E3 ubiquitin protein ligase 1										
57544	TXNDC16	thioredoxin domain containing 16										
57646	USP28	ubiquitin specific peptidase 28										
57661	PHRF1	PHD and ring finger domains 1										
57669	EPB41L5	erythrocyte membrane protein band 4.1 like 5										
57683	ZDBF2	zinc finger, DBF-type containing 2										
57705	WDFY4	WDFY family member 4										
57706	DENND1A	DENN/MADD domain containing 1A										
57730	ANKRD36B	ankyrin repeat domain 36B										
5775	PTPN4	protein tyrosine phosphatase, non-receptor type 4 (megakaryocyte)										
5780	PTPN9	protein tyrosine phosphatase, non-receptor type 9										
5863	RGL2	ral guanine nucleotide dissociation stimulator-like 2										
5935	RBM3	RNA binding motif (RNP1, RRM) protein 3										
5991	RFX3	regulatory factor X, 3 (influences HLA class II expression)										
6103	RPGR	retinitis pigmentosa GTPase regulator										
63892	THADA	thyroid adenoma associated										
63897	HEATR6	HEAT repeat containing 6										
63901	FAM111A	family with sequence similarity 111, member A										
63979	FIGNL1	fidgetin-like 1										
63982	ANO3	anoctamin 3										
64172	OSGEPL1	O-sialoglycoprotein endopeptidase-like 1										
64210	MMS19	MMS19 nucleotide excision repair homolog (S. cerevisiae)										
643911	CRNDE	colorectal neoplasia differentially expressed (non-protein coding)										
64427	TTC31	tetratricopeptide repeat domain 31										
645784	ANKRD36BP2	ankyrin repeat domain 36B pseudogene 2										
64601	VPS16	vacuolar protein sorting 16 homolog (S. cerevisiae)										
64780	MICAL1	microtubule associated monoxygenase, calponin and LIM domain containing 1										
64860	ARMCX5	armadillo repeat containing, X-linked 5										
65056	GPBP1	GC-rich promoter binding protein 1										
65094	JMJD4	jumonji domain containing 4										
65250	C5orf42	chromosome 5 open reading frame 42										
653390	RRN3P2	RNA polymerase I transcription factor homolog (S. cerevisiae) pseudogene 2										
65981	CAPRIN2	caprin family member 2										
66036	MTMR9	myotubularin related protein 9										
664701	ZNF826P	zinc finger protein 826, pseudogene										
6793	STK10	serine/threonine kinase 10										
6830	SUPT6H	suppressor of Ty 6 homolog (S. cerevisiae)										
6894	TARBP1	TAR (HIV-1) RNA binding protein 1										
724102	SNHG4	small nucleolar RNA host gene 4 (non-protein coding)										
7444	VRK2	vaccinia related kinase 2										
7543	ZFX	zinc finger protein, X-linked										
7552	ZNF711	zinc finger protein 711										
7626	ZNF75D	zinc finger protein 75D										
7692	ZNF133	zinc finger protein 133										
7707	ZNF148	zinc finger protein 148										
7748	ZNF195	zinc finger protein 195										
7775	ZNF232	zinc finger protein 232										
780	DDR1	discoidin domain receptor tyrosine kinase 1										
78995	C17orf53	chromosome 17 open reading frame 53										
78996	C7orf49	chromosome 7 open reading frame 49										
79022	TMEM106C	transmembrane protein 106C										
79065	ATG9A	ATG9 autophagy related 9 homolog A (S. cerevisiae)										
79101	TAF1D	TATA box binding protein (TBP)-associated factor, RNA polymerase I, D, 41kDa										
79144	PPDPF	pancreatic progenitor cell differentiation and proliferation factor homolog (zebrafish)										
79145	CHCHD7	coiled-coil-helix-coiled-coil-helix domain containing 7										
79147	FKRP	fukutin related protein										
7917	BAG6	BCL2-associated athanogene 6										
79183	TTPAL	tocopherol (alpha) transfer protein-like										
7920	ABHD16A	abhydrolase domain containing 16A										
79415	C17orf62	chromosome 17 open reading frame 62										
79441	HAUS3	HAUS augmin-like complex, subunit 3										
79608	RIC3	resistance to inhibitors of cholinesterase 3 homolog (C. elegans)										
79618	HMBOX1	homeobox containing 1										
79641	ROGDI	rogdi homolog (Drosophila)										
79706	PRKRIP1	PRKR interacting protein 1 (IL11 inducible)										
79716	NPEPL1	aminopeptidase-like 1										
79745	CLIP4	CAP-GLY domain containing linker protein family, member 4										
79772	MCTP1	multiple C2 domains, transmembrane 1										
79792	GSDMD	gasdermin D										
7982	ST7	suppression of tumorigenicity 7										
79897	RPP21	ribonuclease P/MRP 21kDa subunit										
79913	ACTR5	ARP5 actin-related protein 5 homolog (yeast)										
79930	DOK3	docking protein 3										
79932	KIAA0319L	KIAA0319-like										
79961	DENND2D	DENN/MADD domain containing 2D										
79979	TRMT2B	TRM2 tRNA methyltransferase 2 homolog B (S. cerevisiae)										
79982	DNAJB14	DnaJ (Hsp40) homolog, subfamily B, member 14										
79990	PLEKHH3	pleckstrin homology domain containing, family H (with MyTH4 domain) member 3										
80011	FAM192A	family with sequence similarity 192, member A										
80012	PHC3	polyhomeotic homolog 3 (Drosophila)										
80021	TMEM62	transmembrane protein 62										
80059	LRRTM4	leucine rich repeat transmembrane neuronal 4										
80063	ATF7IP2	activating transcription factor 7 interacting protein 2										
80067	DCAF17	DDB1 and CUL4 associated factor 17										
80071	CCDC15	coiled-coil domain containing 15										
80179	MYO19	myosin XIX										
80199	FUZ	fuzzy homolog (Drosophila)										
80205	CHD9	chromodomain helicase DNA binding protein 9										
80221	ACSF2	acyl-CoA synthetase family member 2										
80279	CDK5RAP3	CDK5 regulatory subunit associated protein 3										
8045	RASSF7	Ras association (RalGDS/AF-6) domain family (N-terminal) member 7										
80705	TSGA10	testis specific, 10										
80724	ACAD10	acyl-CoA dehydrogenase family, member 10										
80727	TTYH3	tweety homolog 3 (Drosophila)										
80746	TSEN2	tRNA splicing endonuclease 2 homolog (S. cerevisiae)										
80817	CEP44	centrosomal protein 44kDa										
80821	DDHD1	DDHD domain containing 1										
80833	APOL3	apolipoprotein L, 3										
81532	MOB2	MOB kinase activator 2										
81796	SLCO5A1	solute carrier organic anion transporter family, member 5A1										
81831	NETO2	neuropilin (NRP) and tolloid (TLL)-like 2										
81846	SBF2	SET binding factor 2										
81847	RNF146	ring finger protein 146										
81890	QTRT1	queuine tRNA-ribosyltransferase 1										
8193	DPF1	D4, zinc and double PHD fingers family 1										
8233	ZRSR2	zinc finger (CCCH type), RNA-binding motif and serine/arginine rich 2										
8314	BAP1	BRCA1 associated protein-1 (ubiquitin carboxy-terminal hydrolase)										
83416	FCRL5	Fc receptor-like 5										
83642	SELO	selenoprotein O										
83707	TRPT1	tRNA phosphotransferase 1										
83856	FSD1L	fibronectin type III and SPRY domain containing 1-like										
83875	BCO2	beta-carotene oxygenase 2										
83931	STK40	serine/threonine kinase 40										
83941	TM2D1	TM2 domain containing 1										
84064	HDHD2	haloacid dehalogenase-like hydrolase domain containing 2										
84081	NSRP1	nuclear speckle splicing regulatory protein 1										
84129	ACAD11	acyl-CoA dehydrogenase family, member 11										
84131	CEP78	centrosomal protein 78kDa										
84140	FAM161A	family with sequence similarity 161, member A										
84232	MAF1	MAF1 homolog (S. cerevisiae)										
84268	RPAIN	RPA interacting protein										
84269	CHCHD5	coiled-coil-helix-coiled-coil-helix domain containing 5										
84276	NICN1	nicolin 1										
84286	TMEM175	transmembrane protein 175										
84326	C16orf13	chromosome 16 open reading frame 13										
84542	KIAA1841	KIAA1841										
84545	MRPL43	mitochondrial ribosomal protein L43										
84629	TNRC18	trinucleotide repeat containing 18										
84661	DPY30	dpy-30 homolog (C. elegans)										
84668	FAM126A	family with sequence similarity 126, member A										
84678	KDM2B	lysine (K)-specific demethylase 2B										
84722	PSRC1	proline/serine-rich coiled-coil 1										
84824	FCRLA	Fc receptor-like A										
84833	USMG5	up-regulated during skeletal muscle growth 5 homolog (mouse)										
84901	NFATC2IP	nuclear factor of activated T-cells, cytoplasmic, calcineurin-dependent 2 interacting protein										
84993	UBL7	ubiquitin-like 7 (bone marrow stromal cell-derived)										
85012	TCEAL3	transcription elongation factor A (SII)-like 3										
85014	TMEM141	transmembrane protein 141										
85445	CNTNAP4	contactin associated protein-like 4										
8631	SKAP1	src kinase associated phosphoprotein 1										
8859	STK19	serine/threonine kinase 19										
889	KRIT1	KRIT1, ankyrin repeat containing										
8904	CPNE1	copine I										
8906	AP1G2	adaptor-related protein complex 1, gamma 2 subunit										
89781	HPS4	Hermansky-Pudlak syndrome 4										
89790	SIGLEC10	sialic acid binding Ig-like lectin 10										
89894	TMEM116	transmembrane protein 116										
9044	BTAF1	BTAF1 RNA polymerase II, B-TFIID transcription factor-associated, 170kDa (Mot1 homolog, S. cerevisiae)										
90990	KIFC2	kinesin family member C2										
91612	CHURC1	churchill domain containing 1										
9202	ZMYM4	zinc finger, MYM-type 4										
92170	MTG1	mitochondrial GTPase 1 homolog (S. cerevisiae)										
92270	ATP6AP1L	ATPase, H+ transporting, lysosomal accessory protein 1-like										
9229	DLGAP1	discs, large (Drosophila) homolog-associated protein 1										
92482	BBIP1	BBSome interacting protein 1										
93517	SDR42E1	short chain dehydrogenase/reductase family 42E, member 1										
94120	SYTL3	synaptotagmin-like 3										
9526	MPDU1	mannose-P-dolichol utilization defect 1										
9559	VPS26A	vacuolar protein sorting 26 homolog A (S. pombe)										
9567	GTPBP1	GTP binding protein 1										
963	CD53	CD53 molecule										
9643	MORF4L2	mortality factor 4 like 2										
9678	PHF14	PHD finger protein 14										
9692	KIAA0391	KIAA0391										
9701	PPP6R2	protein phosphatase 6, regulatory subunit 2										
9736	USP34	ubiquitin specific peptidase 34										
9770	RASSF2	Ras association (RalGDS/AF-6) domain family member 2										
9814	SFI1	Sfi1 homolog, spindle assembly associated (yeast)										
9819	TSC22D2	TSC22 domain family, member 2										
9841	ZBTB24	zinc finger and BTB domain containing 24										
9849	ZNF518A	zinc finger protein 518A										
9854	C2CD2L	C2CD2-like										
9897	KIAA0196	KIAA0196										
9898	UBAP2L	ubiquitin associated protein 2-like										
9919	SEC16A	SEC16 homolog A (S. cerevisiae)										

Supplementary Table 7: SU-DHL-5 Alternative Splicing MsigDB Analysis

Overlap Results						
Collection(s):	CP, CP:BIOCARTA, CP:KEGG, CP:REACTOME, H					
# overlaps shown:	10					
# genesets in collections:	1379					
# genes in comparison (n):	1176					
# genes in universe (N):	45956					
Gene Set Name	# Genes in Gene Set (K)	Description	# Genes in Overlap (k)	k/K	p-value	FDR q-value
REACTOME_CELL_CYCLE	421	Genes involved in Cell Cycle	49	0.1164	1.47E-18	1.65E-15
HALLMARK_E2F_TARGETS	200	Genes encoding cell cycle related targets of E2F transcription factors.	34	0.17	2.40E-18	1.65E-15
REACTOME_CELL_CYCLE_MITOTIC	325	Genes involved in Cell Cycle, Mitotic	42	0.1292	9.32E-18	4.29E-15
REACTOME_IMMUNE_SYSTEM	933	Genes involved in Immune System	72	0.0772	1.66E-16	5.74E-14
HALLMARK_MITOTIC_SPINDLE	200	Genes important for mitotic spindle assembly.	30	0.15	7.81E-15	2.16E-12
KEGG_UBIQUITIN_MEDIATED_PROTEOLYSIS	138	Ubiquitin mediated proteolysis	25	0.1812	1.58E-14	3.63E-12
KEGG_RIBOSOME	88	Ribosome	20	0.2273	7.32E-14	1.44E-11
REACTOME_ADAPTIVE_IMMUNE_SYSTEM	539	Genes involved in Adaptive Immune System	47	0.0872	4.37E-13	7.53E-11
REACTOME_METABOLISM_OF_PROTEINS	518	Genes involved in Metabolism of proteins	45	0.0869	1.56E-12	2.40E-10
REACTOME_METABOLISM_OF_RNA	330	Genes involved in Metabolism of RNA	35	0.1061	1.80E-12	2.44E-10

Gene/Gene Set Overlap Matrix

Entrez Gene Id	Gene Symbol	Gene Description	REACTOME_CELL_CYCLE	HALLMARK_E2F_TARGETS	REACTOME_CELL_CYCLE_MITOTIC	REACTOME_IMMUNE_SYSTEM	HALLMARK_MITOTIC_SPINDLE	KEGG_UBIQUITIN_MEDIATED_PROTEOLYSIS	KEGG_RIBOSOME	REACTOME_ADAPTIVE_IMMUNE_SYSTEM	REACTOME_METABOLISM_OF_PROTEINS	REACTOME_METABOLISM_OF_RNA
7514	XPO1	exportin 1 (CRM1 homolog, yeast)	REACTOME_CELL_CYCLE	HALLMARK_E2F_TARGETS	REACTOME_CELL_CYCLE_MITOTIC							REACTOME_METABOLISM_OF_RNA
994	CDC25B	cell division cycle 25 homolog B (S. pombe)	REACTOME_CELL_CYCLE	HALLMARK_E2F_TARGETS	REACTOME_CELL_CYCLE_MITOTIC							
23594	ORC6	origin recognition complex, subunit 6	REACTOME_CELL_CYCLE	HALLMARK_E2F_TARGETS	REACTOME_CELL_CYCLE_MITOTIC							
9212	AURKB	aurora kinase B	REACTOME_CELL_CYCLE	HALLMARK_E2F_TARGETS	REACTOME_CELL_CYCLE_MITOTIC							
5424	POLD1	polymerase (DNA directed), delta 1, catalytic subunit 125kDa	REACTOME_CELL_CYCLE	HALLMARK_E2F_TARGETS	REACTOME_CELL_CYCLE_MITOTIC							
4173	MCM4	minichromosome maintenance complex component 4	REACTOME_CELL_CYCLE	HALLMARK_E2F_TARGETS	REACTOME_CELL_CYCLE_MITOTIC							
9837	GINS1	GINS complex subunit 1 (Psf1 homolog)	REACTOME_CELL_CYCLE	HALLMARK_E2F_TARGETS	REACTOME_CELL_CYCLE_MITOTIC							
672	BRCA1	breast cancer 1, early onset	REACTOME_CELL_CYCLE	HALLMARK_E2F_TARGETS				KEGG_UBIQUITIN_MEDIATED_PROTEOLYSIS				
11200	CHEK2	CHK2 checkpoint homolog (S. pombe)	REACTOME_CELL_CYCLE	HALLMARK_E2F_TARGETS								
5810	RAD1	RAD1 homolog (S. pombe)	REACTOME_CELL_CYCLE	HALLMARK_E2F_TARGETS								
9493	KIF23	kinesin family member 23	REACTOME_CELL_CYCLE		REACTOME_CELL_CYCLE_MITOTIC	REACTOME_IMMUNE_SYSTEM	HALLMARK_MITOTIC_SPINDLE			REACTOME_ADAPTIVE_IMMUNE_SYSTEM		
11065	UBE2C	ubiquitin-conjugating enzyme E2C	REACTOME_CELL_CYCLE		REACTOME_CELL_CYCLE_MITOTIC	REACTOME_IMMUNE_SYSTEM		KEGG_UBIQUITIN_MEDIATED_PROTEOLYSIS		REACTOME_ADAPTIVE_IMMUNE_SYSTEM		
29882	ANAPC2	anaphase promoting complex subunit 2	REACTOME_CELL_CYCLE		REACTOME_CELL_CYCLE_MITOTIC	REACTOME_IMMUNE_SYSTEM		KEGG_UBIQUITIN_MEDIATED_PROTEOLYSIS		REACTOME_ADAPTIVE_IMMUNE_SYSTEM		
6233	RPS27A	ribosomal protein S27a	REACTOME_CELL_CYCLE		REACTOME_CELL_CYCLE_MITOTIC	REACTOME_IMMUNE_SYSTEM			KEGG_RIBOSOME	REACTOME_ADAPTIVE_IMMUNE_SYSTEM	REACTOME_METABOLISM_OF_PROTEINS	REACTOME_METABOLISM_OF_RNA
5720	PSME1	proteasome (prosome, macropain) activator subunit 1 (PA28 alpha)	REACTOME_CELL_CYCLE		REACTOME_CELL_CYCLE_MITOTIC	REACTOME_IMMUNE_SYSTEM				REACTOME_ADAPTIVE_IMMUNE_SYSTEM		REACTOME_METABOLISM_OF_RNA
5717	PSMD11	proteasome (prosome, macropain) 26S subunit, non-ATPase, 11	REACTOME_CELL_CYCLE		REACTOME_CELL_CYCLE_MITOTIC	REACTOME_IMMUNE_SYSTEM				REACTOME_ADAPTIVE_IMMUNE_SYSTEM		REACTOME_METABOLISM_OF_RNA
10121	ACTR1A	ARP1 actin-related protein 1 homolog A, centractin alpha (yeast)	REACTOME_CELL_CYCLE		REACTOME_CELL_CYCLE_MITOTIC	REACTOME_IMMUNE_SYSTEM				REACTOME_ADAPTIVE_IMMUNE_SYSTEM		
11258	DCTN3	dynactin 3 (p22)	REACTOME_CELL_CYCLE		REACTOME_CELL_CYCLE_MITOTIC	REACTOME_IMMUNE_SYSTEM				REACTOME_ADAPTIVE_IMMUNE_SYSTEM		
4926	NUMA1	nuclear mitotic apparatus protein 1	REACTOME_CELL_CYCLE		REACTOME_CELL_CYCLE_MITOTIC		HALLMARK_MITOTIC_SPINDLE					
11064	CNTRL	centriolin	REACTOME_CELL_CYCLE		REACTOME_CELL_CYCLE_MITOTIC		HALLMARK_MITOTIC_SPINDLE					
5116	PCNT	pericentrin	REACTOME_CELL_CYCLE		REACTOME_CELL_CYCLE_MITOTIC		HALLMARK_MITOTIC_SPINDLE					
11190	CEP250	centrosomal protein 250kDa	REACTOME_CELL_CYCLE		REACTOME_CELL_CYCLE_MITOTIC		HALLMARK_MITOTIC_SPINDLE					
55125	CEP192	centrosomal protein 192kDa	REACTOME_CELL_CYCLE		REACTOME_CELL_CYCLE_MITOTIC		HALLMARK_MITOTIC_SPINDLE					
55755	CDK5RAP2	CDK5 regulatory subunit associated protein 2	REACTOME_CELL_CYCLE		REACTOME_CELL_CYCLE_MITOTIC		HALLMARK_MITOTIC_SPINDLE					
7840	ALMS1	Alstrom syndrome 1	REACTOME_CELL_CYCLE		REACTOME_CELL_CYCLE_MITOTIC		HALLMARK_MITOTIC_SPINDLE					
9702	CEP57	centrosomal protein 57kDa	REACTOME_CELL_CYCLE		REACTOME_CELL_CYCLE_MITOTIC		HALLMARK_MITOTIC_SPINDLE					
9735	KNTC1	kinetochore associated 1	REACTOME_CELL_CYCLE		REACTOME_CELL_CYCLE_MITOTIC		HALLMARK_MITOTIC_SPINDLE					
1869	E2F1	E2F transcription factor 1	REACTOME_CELL_CYCLE		REACTOME_CELL_CYCLE_MITOTIC							
995	CDC25C	cell division cycle 25 homolog C (S. pombe)	REACTOME_CELL_CYCLE		REACTOME_CELL_CYCLE_MITOTIC							
1454	CSNK1E	casein kinase 1, epsilon	REACTOME_CELL_CYCLE		REACTOME_CELL_CYCLE_MITOTIC							
22897	CEP164	centrosomal protein 164kDa	REACTOME_CELL_CYCLE		REACTOME_CELL_CYCLE_MITOTIC							
8481	OFD1	oral-facial-digital syndrome 1	REACTOME_CELL_CYCLE		REACTOME_CELL_CYCLE_MITOTIC							
8636	SSNA1	Sjogren syndrome nuclear autoantigen 1	REACTOME_CELL_CYCLE		REACTOME_CELL_CYCLE_MITOTIC							
8318	CDC45	cell division cycle 45 homolog (S. cerevisiae)	REACTOME_CELL_CYCLE		REACTOME_CELL_CYCLE_MITOTIC							
7027	TFDP1	transcription factor Dp-1	REACTOME_CELL_CYCLE		REACTOME_CELL_CYCLE_MITOTIC							
8379	MAD1L1	MAD1 mitotic arrest deficient-like 1 (yeast)	REACTOME_CELL_CYCLE		REACTOME_CELL_CYCLE_MITOTIC							
84515	MCM8	minichromosome maintenance complex component 8	REACTOME_CELL_CYCLE		REACTOME_CELL_CYCLE_MITOTIC							
10735	STAG2	stromal antigen 2	REACTOME_CELL_CYCLE		REACTOME_CELL_CYCLE_MITOTIC							
5905	RANGAP1	Ran GTPase activating protein 1	REACTOME_CELL_CYCLE		REACTOME_CELL_CYCLE_MITOTIC							
23421	ITGB3BP	integrin beta 3 binding protein (beta3-endonexin)	REACTOME_CELL_CYCLE		REACTOME_CELL_CYCLE_MITOTIC							
25936	NSL1	NSL1, MIND kinetochore complex component, homolog (S. cerevisiae)	REACTOME_CELL_CYCLE		REACTOME_CELL_CYCLE_MITOTIC							
348235	SKA2	spindle and kinetochore associated complex subunit 2	REACTOME_CELL_CYCLE		REACTOME_CELL_CYCLE_MITOTIC							
64689	GORASP1	golgi reassembly stacking protein 1, 65kDa	REACTOME_CELL_CYCLE		REACTOME_CELL_CYCLE_MITOTIC							
1763	DNA2	DNA replication helicase 2 homolog (yeast)	REACTOME_CELL_CYCLE		REACTOME_CELL_CYCLE_MITOTIC							
132660	LIN54	lin-54 homolog (C. elegans)	REACTOME_CELL_CYCLE		REACTOME_CELL_CYCLE_MITOTIC							
4193	MDM2	Mdm2 p53 binding protein homolog (mouse)	REACTOME_CELL_CYCLE			REACTOME_IMMUNE_SYSTEM		KEGG_UBIQUITIN_MEDIATED_PROTEOLYSIS		REACTOME_ADAPTIVE_IMMUNE_SYSTEM		
64326	RFWD2	ring finger and WD repeat domain 2	REACTOME_CELL_CYCLE					KEGG_UBIQUITIN_MEDIATED_PROTEOLYSIS				
472	ATM	ataxia telangiectasia mutated	REACTOME_CELL_CYCLE									
5884	RAD17	RAD17 homolog (S. pombe)	REACTOME_CELL_CYCLE									
9700	ESPL1	extra spindle pole bodies homolog 1 (S. cerevisiae)		HALLMARK_E2F_TARGETS			HALLMARK_MITOTIC_SPINDLE					
1062	CENPE	centromere protein E, 312kDa		HALLMARK_E2F_TARGETS			HALLMARK_MITOTIC_SPINDLE					
10528	NOP56	NOP56 ribonucleoprotein homolog (yeast)		HALLMARK_E2F_TARGETS							REACTOME_METABOLISM_OF_PROTEINS	
6426	SRSF1	serine/arginine-rich splicing factor 1		HALLMARK_E2F_TARGETS								
4678	NASP	nuclear autoantigenic sperm protein (histone-binding)		HALLMARK_E2F_TARGETS								
4288	MKI67	antigen identified by monoclonal antibody Ki-67		HALLMARK_E2F_TARGETS								
7112	TMPO	thymopoietin		HALLMARK_E2F_TARGETS								
4673	NAP1L1	nucleosome assembly protein 1-like 1		HALLMARK_E2F_TARGETS								
10606	PAICS	phosphoribosylaminoimidazole carboxylase, phosphoribosylaminoimidazole succinocarboxamide synthetase		HALLMARK_E2F_TARGETS								
4292	MLH1	mutL homolog 1, colon cancer, nonpolyposis type 2 (E. coli)		HALLMARK_E2F_TARGETS								
51691	NAA38	N(alpha)-acetyltransferase 38, NatC auxiliary subunit		HALLMARK_E2F_TARGETS								
3148	HMGB2	high mobility group box 2		HALLMARK_E2F_TARGETS								
1786	DNMT1	DNA (cytosine-5-)-methyltransferase 1		HALLMARK_E2F_TARGETS								
81624	DIAPH3	diaphanous homolog 3 (Drosophila)		HALLMARK_E2F_TARGETS								
9238	TBRG4	transforming growth factor beta regulator 4		HALLMARK_E2F_TARGETS								
10527	IPO7	importin 7		HALLMARK_E2F_TARGETS								
10635	RAD51AP1	RAD51 associated protein 1		HALLMARK_E2F_TARGETS								
197335	WDR90	WD repeat domain 90		HALLMARK_E2F_TARGETS								
29893	PSMC3IP	PSMC3 interacting protein		HALLMARK_E2F_TARGETS								
29980	DONSON	downstream neighbor of SON		HALLMARK_E2F_TARGETS								
6941	TCF19	transcription factor 19		HALLMARK_E2F_TARGETS								
83461	CDCA3	cell division cycle associated 3		HALLMARK_E2F_TARGETS								
9833	MELK	maternal embryonic leucine zipper kinase		HALLMARK_E2F_TARGETS								
9924	PAN2	PAN2 poly(A) specific ribonuclease subunit homolog (S. cerevisiae)		HALLMARK_E2F_TARGETS								
55294	FBXW7	F-box and WD repeat domain containing 7				REACTOME_IMMUNE_SYSTEM		KEGG_UBIQUITIN_MEDIATED_PROTEOLYSIS		REACTOME_ADAPTIVE_IMMUNE_SYSTEM	REACTOME_METABOLISM_OF_PROTEINS	
7318	UBA7	ubiquitin-like modifier activating enzyme 7				REACTOME_IMMUNE_SYSTEM		KEGG_UBIQUITIN_MEDIATED_PROTEOLYSIS		REACTOME_ADAPTIVE_IMMUNE_SYSTEM		
7322	UBE2D2	ubiquitin-conjugating enzyme E2D 2				REACTOME_IMMUNE_SYSTEM		KEGG_UBIQUITIN_MEDIATED_PROTEOLYSIS		REACTOME_ADAPTIVE_IMMUNE_SYSTEM		
6921	TCEB1	transcription elongation factor B (SIII), polypeptide 1 (15kDa, elongin C)				REACTOME_IMMUNE_SYSTEM		KEGG_UBIQUITIN_MEDIATED_PROTEOLYSIS		REACTOME_ADAPTIVE_IMMUNE_SYSTEM		
6923	TCEB2	transcription elongation factor B (SIII), polypeptide 2 (18kDa, elongin B)				REACTOME_IMMUNE_SYSTEM		KEGG_UBIQUITIN_MEDIATED_PROTEOLYSIS		REACTOME_ADAPTIVE_IMMUNE_SYSTEM		
7317	UBA1	ubiquitin-like modifier activating enzyme 1				REACTOME_IMMUNE_SYSTEM		KEGG_UBIQUITIN_MEDIATED_PROTEOLYSIS		REACTOME_ADAPTIVE_IMMUNE_SYSTEM		
7327	UBE2G2	ubiquitin-conjugating enzyme E2G 2				REACTOME_IMMUNE_SYSTEM		KEGG_UBIQUITIN_MEDIATED_PROTEOLYSIS		REACTOME_ADAPTIVE_IMMUNE_SYSTEM		
7319	UBE2A	ubiquitin-conjugating enzyme E2A				REACTOME_IMMUNE_SYSTEM		KEGG_UBIQUITIN_MEDIATED_PROTEOLYSIS		REACTOME_ADAPTIVE_IMMUNE_SYSTEM		
4591	TRIM37	tripartite motif containing 37				REACTOME_IMMUNE_SYSTEM		KEGG_UBIQUITIN_MEDIATED_PROTEOLYSIS		REACTOME_ADAPTIVE_IMMUNE_SYSTEM		
29927	SEC61A1	Sec61 alpha 1 subunit (S. cerevisiae)				REACTOME_IMMUNE_SYSTEM				REACTOME_ADAPTIVE_IMMUNE_SYSTEM	REACTOME_METABOLISM_OF_PROTEINS	
55176	SEC61A2	Sec61 alpha 2 subunit (S. cerevisiae)				REACTOME_IMMUNE_SYSTEM				REACTOME_ADAPTIVE_IMMUNE_SYSTEM	REACTOME_METABOLISM_OF_PROTEINS	
10427	SEC24B	SEC24 family, member B (S. cerevisiae)				REACTOME_IMMUNE_SYSTEM				REACTOME_ADAPTIVE_IMMUNE_SYSTEM	REACTOME_METABOLISM_OF_PROTEINS	
972	CD74	CD74 molecule, major histocompatibility complex, class II invariant chain				REACTOME_IMMUNE_SYSTEM				REACTOME_ADAPTIVE_IMMUNE_SYSTEM		
5777	PTPN6	protein tyrosine phosphatase, non-receptor type 6				REACTOME_IMMUNE_SYSTEM				REACTOME_ADAPTIVE_IMMUNE_SYSTEM		
5788	PTPRC	protein tyrosine phosphatase, receptor type, C				REACTOME_IMMUNE_SYSTEM				REACTOME_ADAPTIVE_IMMUNE_SYSTEM		
973	CD79A	CD79a molecule, immunoglobulin-associated alpha				REACTOME_IMMUNE_SYSTEM				REACTOME_ADAPTIVE_IMMUNE_SYSTEM		
2213	FCGR2B	Fc fragment of IgG, low affinity IIb, receptor (CD32)				REACTOME_IMMUNE_SYSTEM				REACTOME_ADAPTIVE_IMMUNE_SYSTEM		
919	CD247	CD247 molecule				REACTOME_IMMUNE_SYSTEM				REACTOME_ADAPTIVE_IMMUNE_SYSTEM		
916	CD3E	CD3e molecule, epsilon (CD3-TCR complex)				REACTOME_IMMUNE_SYSTEM				REACTOME_ADAPTIVE_IMMUNE_SYSTEM		
3108	HLA-DMA	major histocompatibility complex, class II, DM alpha				REACTOME_IMMUNE_SYSTEM				REACTOME_ADAPTIVE_IMMUNE_SYSTEM		
3112	HLA-DOB	major histocompatibility complex, class II, DO beta				REACTOME_IMMUNE_SYSTEM				REACTOME_ADAPTIVE_IMMUNE_SYSTEM		
255488	RNF144B	ring finger protein 144B				REACTOME_IMMUNE_SYSTEM				REACTOME_ADAPTIVE_IMMUNE_SYSTEM		
10533	ATG7	ATG7 autophagy related 7 homolog (S. cerevisiae)				REACTOME_IMMUNE_SYSTEM				REACTOME_ADAPTIVE_IMMUNE_SYSTEM		
26118	WSB1	WD repeat and SOCS box containing 1				REACTOME_IMMUNE_SYSTEM				REACTOME_ADAPTIVE_IMMUNE_SYSTEM		
55182	RNF220	ring finger protein 220				REACTOME_IMMUNE_SYSTEM				REACTOME_ADAPTIVE_IMMUNE_SYSTEM		
93611	FBXO44	F-box protein 44				REACTOME_IMMUNE_SYSTEM				REACTOME_ADAPTIVE_IMMUNE_SYSTEM		
9666	DZIP3	DAZ interacting protein 3, zinc finger				REACTOME_IMMUNE_SYSTEM				REACTOME_ADAPTIVE_IMMUNE_SYSTEM		
2475	MTOR	mechanistic target of rapamycin (serine/threonine kinase)				REACTOME_IMMUNE_SYSTEM				REACTOME_ADAPTIVE_IMMUNE_SYSTEM		
64223	MLST8	MTOR associated protein, LST8 homolog (S. cerevisiae)				REACTOME_IMMUNE_SYSTEM				REACTOME_ADAPTIVE_IMMUNE_SYSTEM		
5728	PTEN	phosphatase and tensin homolog				REACTOME_IMMUNE_SYSTEM				REACTOME_ADAPTIVE_IMMUNE_SYSTEM		
1147	CHUK	conserved helix-loop-helix ubiquitous kinase				REACTOME_IMMUNE_SYSTEM				REACTOME_ADAPTIVE_IMMUNE_SYSTEM		
5336	PLCG2	phospholipase C, gamma 2 (phosphatidylinositol-specific)				REACTOME_IMMUNE_SYSTEM				REACTOME_ADAPTIVE_IMMUNE_SYSTEM		
118788	PIK3AP1	phosphoinositide-3-kinase adaptor protein 1				REACTOME_IMMUNE_SYSTEM				REACTOME_ADAPTIVE_IMMUNE_SYSTEM		
5908	RAP1B	RAP1B, member of RAS oncogene family				REACTOME_IMMUNE_SYSTEM				REACTOME_ADAPTIVE_IMMUNE_SYSTEM		
3710	ITPR3	inositol 1,4,5-trisphosphate receptor, type 3				REACTOME_IMMUNE_SYSTEM				REACTOME_ADAPTIVE_IMMUNE_SYSTEM		
8905	AP1S2	adaptor-related protein complex 1, sigma 2 subunit				REACTOME_IMMUNE_SYSTEM				REACTOME_ADAPTIVE_IMMUNE_SYSTEM		
10120	ACTR1B	ARP1 actin-related protein 1 homolog B, centractin beta (yeast)				REACTOME_IMMUNE_SYSTEM				REACTOME_ADAPTIVE_IMMUNE_SYSTEM		
120425	AMICA1	adhesion molecule, interacts with CXADR antigen 1				REACTOME_IMMUNE_SYSTEM				REACTOME_ADAPTIVE_IMMUNE_SYSTEM		
1981	EIF4G1	eukaryotic translation initiation factor 4 gamma, 1				REACTOME_IMMUNE_SYSTEM					REACTOME_METABOLISM_OF_PROTEINS	REACTOME_METABOLISM_OF_RNA
1974	EIF4A2	eukaryotic translation initiation factor 4A2				REACTOME_IMMUNE_SYSTEM					REACTOME_METABOLISM_OF_PROTEINS	REACTOME_METABOLISM_OF_RNA
8086	AAAS	achalasia, adrenocortical insufficiency, alacrimia				REACTOME_IMMUNE_SYSTEM						REACTOME_METABOLISM_OF_RNA
23636	NUP62	nucleoporin 62kDa				REACTOME_IMMUNE_SYSTEM						REACTOME_METABOLISM_OF_RNA
51447	IP6K2	inositol hexakisphosphate kinase 2				REACTOME_IMMUNE_SYSTEM						
3665	IRF7	interferon regulatory factor 7				REACTOME_IMMUNE_SYSTEM						
6773	STAT2	signal transducer and activator of transcription 2, 113kDa				REACTOME_IMMUNE_SYSTEM						
7706	TRIM25	tripartite motif containing 25				REACTOME_IMMUNE_SYSTEM						
7096	TLR1	toll-like receptor 1				REACTOME_IMMUNE_SYSTEM						
6776	STAT5A	signal transducer and activator of transcription 5A				REACTOME_IMMUNE_SYSTEM						
3663	IRF5	interferon regulatory factor 5				REACTOME_IMMUNE_SYSTEM						
5094	PCBP2	poly(rC) binding protein 2				REACTOME_IMMUNE_SYSTEM						
3661	IRF3	interferon regulatory factor 3				REACTOME_IMMUNE_SYSTEM						
55593	OTUD5	OTU domain containing 5				REACTOME_IMMUNE_SYSTEM						
7297	TYK2	tyrosine kinase 2				REACTOME_IMMUNE_SYSTEM						
25820	ARIH1	ariadne homolog, ubiquitin-conjugating enzyme E2 binding protein, 1 (Drosophila)				REACTOME_IMMUNE_SYSTEM						
10010	TANK	TRAF family member-associated NFKB activator				REACTOME_IMMUNE_SYSTEM						
3718	JAK3	Janus kinase 3				REACTOME_IMMUNE_SYSTEM						
3572	IL6ST	interleukin 6 signal transducer (gp130, oncostatin M receptor)				REACTOME_IMMUNE_SYSTEM						
25970	SH2B1	SH2B adaptor protein 1				REACTOME_IMMUNE_SYSTEM						
4205	MEF2A	myocyte enhancer factor 2A				REACTOME_IMMUNE_SYSTEM						
1386	ATF2	activating transcription factor 2				REACTOME_IMMUNE_SYSTEM						
10695	CNPY3	canopy 3 homolog (zebrafish)				REACTOME_IMMUNE_SYSTEM						
9447	AIM2	absent in melanoma 2				REACTOME_IMMUNE_SYSTEM						
4179	CD46	CD46 molecule, complement regulatory protein				REACTOME_IMMUNE_SYSTEM						
29901	SAC3D1	SAC3 domain containing 1					HALLMARK_MITOTIC_SPINDLE					
2316	FLNA	filamin A, alpha					HALLMARK_MITOTIC_SPINDLE					
7248	TSC1	tuberous sclerosis 1					HALLMARK_MITOTIC_SPINDLE					
89941	RHOT2	ras homolog gene family, member T2					HALLMARK_MITOTIC_SPINDLE					
393	ARHGAP4	Rho GTPase activating protein 4					HALLMARK_MITOTIC_SPINDLE					
11214	AKAP13	A kinase (PRKA) anchor protein 13					HALLMARK_MITOTIC_SPINDLE					
2934	GSN	gelsolin					HALLMARK_MITOTIC_SPINDLE					
81	ACTN4	actinin, alpha 4					HALLMARK_MITOTIC_SPINDLE					
9344	TAOK2	TAO kinase 2					HALLMARK_MITOTIC_SPINDLE					
80119	PIF1	PIF1 5'-to-3' DNA helicase homolog (S. cerevisiae)					HALLMARK_MITOTIC_SPINDLE					
1739	DLG1	discs, large homolog 1 (Drosophila)					HALLMARK_MITOTIC_SPINDLE					
2035	EPB41	erythrocyte membrane protein band 4.1 (elliptocytosis 1, RH-linked)					HALLMARK_MITOTIC_SPINDLE					
23607	CD2AP	CD2-associated protein					HALLMARK_MITOTIC_SPINDLE					
3996	LLGL1	lethal giant larvae homolog 1 (Drosophila)					HALLMARK_MITOTIC_SPINDLE					
151987	PPP4R2	protein phosphatase 4, regulatory subunit 2					HALLMARK_MITOTIC_SPINDLE					
201176	ARHGAP27	Rho GTPase activating protein 27					HALLMARK_MITOTIC_SPINDLE					
51174	TUBD1	tubulin, delta 1					HALLMARK_MITOTIC_SPINDLE					
9771	RAPGEF5	Rap guanine nucleotide exchange factor (GEF) 5					HALLMARK_MITOTIC_SPINDLE					
8451	CUL4A	cullin 4A						KEGG_UBIQUITIN_MEDIATED_PROTEOLYSIS				
1643	DDB2	damage-specific DNA binding protein 2, 48kDa						KEGG_UBIQUITIN_MEDIATED_PROTEOLYSIS				
55120	FANCL	Fanconi anemia, complementation group L						KEGG_UBIQUITIN_MEDIATED_PROTEOLYSIS				
84447	SYVN1	synovial apoptosis inhibitor 1, synoviolin						KEGG_UBIQUITIN_MEDIATED_PROTEOLYSIS				
331	XIAP	X-linked inhibitor of apoptosis						KEGG_UBIQUITIN_MEDIATED_PROTEOLYSIS				
23759	PPIL2	peptidylprolyl isomerase (cyclophilin)-like 2						KEGG_UBIQUITIN_MEDIATED_PROTEOLYSIS				
8916	HERC3	hect domain and RLD 3						KEGG_UBIQUITIN_MEDIATED_PROTEOLYSIS				
23295	MGRN1	mahogunin, ring finger 1						KEGG_UBIQUITIN_MEDIATED_PROTEOLYSIS				
51366	UBR5	ubiquitin protein ligase E3 component n-recognin 5						KEGG_UBIQUITIN_MEDIATED_PROTEOLYSIS				
57448	BIRC6	baculoviral IAP repeat containing 6						KEGG_UBIQUITIN_MEDIATED_PROTEOLYSIS				
8925	HERC1	hect (homologous to the E6-AP (UBE3A) carboxyl terminus) domain and RCC1 (CHC1)-like domain (RLD) 1						KEGG_UBIQUITIN_MEDIATED_PROTEOLYSIS				
6206	RPS12	ribosomal protein S12							KEGG_RIBOSOME		REACTOME_METABOLISM_OF_PROTEINS	REACTOME_METABOLISM_OF_RNA
25873	RPL36	ribosomal protein L36							KEGG_RIBOSOME		REACTOME_METABOLISM_OF_PROTEINS	REACTOME_METABOLISM_OF_RNA
6223	RPS19	ribosomal protein S19							KEGG_RIBOSOME		REACTOME_METABOLISM_OF_PROTEINS	REACTOME_METABOLISM_OF_RNA
6194	RPS6	ribosomal protein S6							KEGG_RIBOSOME		REACTOME_METABOLISM_OF_PROTEINS	REACTOME_METABOLISM_OF_RNA
6175	RPLP0	ribosomal protein, large, P0							KEGG_RIBOSOME		REACTOME_METABOLISM_OF_PROTEINS	REACTOME_METABOLISM_OF_RNA
6135	RPL11	ribosomal protein L11							KEGG_RIBOSOME		REACTOME_METABOLISM_OF_PROTEINS	REACTOME_METABOLISM_OF_RNA
6125	RPL5	ribosomal protein L5							KEGG_RIBOSOME		REACTOME_METABOLISM_OF_PROTEINS	REACTOME_METABOLISM_OF_RNA
9349	RPL23	ribosomal protein L23							KEGG_RIBOSOME		REACTOME_METABOLISM_OF_PROTEINS	REACTOME_METABOLISM_OF_RNA
2197	FAU	Finkel-Biskis-Reilly murine sarcoma virus (FBR-MuSV) ubiquitously expressed							KEGG_RIBOSOME		REACTOME_METABOLISM_OF_PROTEINS	REACTOME_METABOLISM_OF_RNA
6201	RPS7	ribosomal protein S7							KEGG_RIBOSOME		REACTOME_METABOLISM_OF_PROTEINS	REACTOME_METABOLISM_OF_RNA
6217	RPS16	ribosomal protein S16							KEGG_RIBOSOME		REACTOME_METABOLISM_OF_PROTEINS	REACTOME_METABOLISM_OF_RNA
23521	RPL13A	ribosomal protein L13a							KEGG_RIBOSOME		REACTOME_METABOLISM_OF_PROTEINS	REACTOME_METABOLISM_OF_RNA
6134	RPL10	ribosomal protein L10							KEGG_RIBOSOME		REACTOME_METABOLISM_OF_PROTEINS	REACTOME_METABOLISM_OF_RNA
6157	RPL27A	ribosomal protein L27a							KEGG_RIBOSOME		REACTOME_METABOLISM_OF_PROTEINS	REACTOME_METABOLISM_OF_RNA
6159	RPL29	ribosomal protein L29							KEGG_RIBOSOME		REACTOME_METABOLISM_OF_PROTEINS	REACTOME_METABOLISM_OF_RNA
6161	RPL32	ribosomal protein L32							KEGG_RIBOSOME		REACTOME_METABOLISM_OF_PROTEINS	REACTOME_METABOLISM_OF_RNA
6167	RPL37	ribosomal protein L37							KEGG_RIBOSOME		REACTOME_METABOLISM_OF_PROTEINS	REACTOME_METABOLISM_OF_RNA
6171	RPL41	ribosomal protein L41							KEGG_RIBOSOME		REACTOME_METABOLISM_OF_PROTEINS	REACTOME_METABOLISM_OF_RNA
6173	RPL36A	ribosomal protein L36a							KEGG_RIBOSOME		REACTOME_METABOLISM_OF_PROTEINS	REACTOME_METABOLISM_OF_RNA
7458	EIF4H	eukaryotic translation initiation factor 4H									REACTOME_METABOLISM_OF_PROTEINS	
27335	EIF3K	eukaryotic translation initiation factor 3, subunit K									REACTOME_METABOLISM_OF_PROTEINS	
90701	SEC11C	SEC11 homolog C (S. cerevisiae)									REACTOME_METABOLISM_OF_PROTEINS	
1936	EEF1D	eukaryotic translation elongation factor 1 delta (guanine nucleotide exchange protein)									REACTOME_METABOLISM_OF_PROTEINS	
3421	IDH3G	isocitrate dehydrogenase 3 (NAD+) gamma									REACTOME_METABOLISM_OF_PROTEINS	
9695	EDEM1	ER degradation enhancer, mannosidase alpha-like 1									REACTOME_METABOLISM_OF_PROTEINS	
10113	PREB	prolactin regulatory element binding									REACTOME_METABOLISM_OF_PROTEINS	
5204	PFDN5	prefoldin subunit 5									REACTOME_METABOLISM_OF_PROTEINS	
29925	GMPPB	GDP-mannose pyrophosphorylase B									REACTOME_METABOLISM_OF_PROTEINS	
9091	PIGQ	phosphatidylinositol glycan anchor biosynthesis, class Q									REACTOME_METABOLISM_OF_PROTEINS	
55757	UGGT2	UDP-glucose glycoprotein glucosyltransferase 2									REACTOME_METABOLISM_OF_PROTEINS	
4249	MGAT5	mannosyl (alpha-1,6-)-glycoprotein beta-1,6-N-acetyl-glucosaminyltransferase									REACTOME_METABOLISM_OF_PROTEINS	
54461	FBXW5	F-box and WD repeat domain containing 5									REACTOME_METABOLISM_OF_PROTEINS	
8733	GPAA1	glycosylphosphatidylinositol anchor attachment protein 1 homolog (yeast)									REACTOME_METABOLISM_OF_PROTEINS	
79623	GALNT14	UDP-N-acetyl-alpha-D-galactosamine:polypeptide N-acetylgalactosaminyltransferase 14 (GalNAc-T14)									REACTOME_METABOLISM_OF_PROTEINS	
131118	DNAJC19	DnaJ (Hsp40) homolog, subfamily C, member 19									REACTOME_METABOLISM_OF_PROTEINS	
51025	PAM16	presequence translocase-associated motor 16 homolog (S. cerevisiae)									REACTOME_METABOLISM_OF_PROTEINS	
617	BCS1L	BCS1-like (S. cerevisiae)									REACTOME_METABOLISM_OF_PROTEINS	
65110	UPF3A	UPF3 regulator of nonsense transcripts homolog A (yeast)										REACTOME_METABOLISM_OF_RNA
23404	EXOSC2	exosome component 2										REACTOME_METABOLISM_OF_RNA
4849	CNOT3	CCR4-NOT transcription complex, subunit 3										REACTOME_METABOLISM_OF_RNA
9337	CNOT8	CCR4-NOT transcription complex, subunit 8										REACTOME_METABOLISM_OF_RNA
54464	XRN1	5'-3' exoribonuclease 1										REACTOME_METABOLISM_OF_RNA
80153	EDC3	enhancer of mRNA decapping 3 homolog (S. cerevisiae)										REACTOME_METABOLISM_OF_RNA
6635	SNRPE	small nuclear ribonucleoprotein polypeptide E										REACTOME_METABOLISM_OF_RNA
6636	SNRPF	small nuclear ribonucleoprotein polypeptide F										REACTOME_METABOLISM_OF_RNA
2130	EWSR1	Ewing sarcoma breakpoint region 1										
10772	SRSF10	serine/arginine-rich splicing factor 10										
4957	ODF2	outer dense fiber of sperm tails 2										
9156	EXO1	exonuclease 1										
6558	SLC12A2	solute carrier family 12 (sodium/potassium/chloride transporters), member 2										
7468	WHSC1	Wolf-Hirschhorn syndrome candidate 1										
1756	DMD	dystrophin										
1112	FOXN3	forkhead box N3										
6760	SS18	synovial sarcoma translocation, chromosome 18										
8899	PRPF4B	PRP4 pre-mRNA processing factor 4 homolog B (yeast)										
5433	POLR2D	polymerase (RNA) II (DNA directed) polypeptide D										
5431	POLR2B	polymerase (RNA) II (DNA directed) polypeptide B, 140kDa										
2177	FANCD2	Fanconi anemia, complementation group D2										
2071	ERCC3	excision repair cross-complementing rodent repair deficiency, complementation group 3 (xeroderma pigmentosum group B complementing)										
328	APEX1	APEX nuclease (multifunctional DNA repair enzyme) 1										
2189	FANCG	Fanconi anemia, complementation group G										
2175	FANCA	Fanconi anemia, complementation group A										
4968	OGG1	8-oxoguanine DNA glycosylase										
4913	NTHL1	nth endonuclease III-like 1 (E. coli)										
23583	SMUG1	single-strand-selective monofunctional uracil-DNA glycosylase 1										
4595	MUTYH	mutY homolog (E. coli)										
121642	ALKBH2	alkB, alkylation repair homolog 2 (E. coli)										
51455	REV1	REV1 homolog (S. cerevisiae)										
5932	RBBP8	retinoblastoma binding protein 8										
637	BID	BH3 interacting domain death agonist										
7156	TOP3A	topoisomerase (DNA) III alpha										
10906	TRAFD1	TRAF-type zinc finger domain containing 1										
100	ADA	adenosine deaminase										
1647	GADD45A	growth arrest and DNA-damage-inducible, alpha										
581	BAX	BCL2-associated X protein										
9538	EI24	etoposide induced 2.4 mRNA										
1606	DGKA	diacylglycerol kinase, alpha 80kDa										
6303	SAT1	spermidine/spermine N1-acetyltransferase 1										
27165	GLS2	glutaminase 2 (liver, mitochondrial)										

10608	MXD4	MAX dimerization protein 4										
51181	DCXR	dicarbonyl/L-xylulose reductase										
56997	ADCK3	aarF domain containing kinase 3										
3094	HINT1	histidine triad nucleotide binding protein 1										
4033	LRMP	lymphoid-restricted membrane protein										
49856	WRAP73	WD repeat containing, antisense to TP73										
51002	TPRKB	TP53RK binding protein										
5820	PVT1	Pvt1 oncogene (non-protein coding)										
7453	WARS	tryptophanyl-tRNA synthetase										
3566	IL4R	interleukin 4 receptor										
961	CD47	CD47 molecule										
135112	NCOA7	nuclear receptor coactivator 7										
55072	RNF31	ring finger protein 31										
64761	PARP12	poly (ADP-ribose) polymerase family, member 12										
1997	ELF1	E74-like factor 1 (ets domain transcription factor)										
83666	PARP9	poly (ADP-ribose) polymerase family, member 9										
85363	TRIM5	tripartite motif containing 5										
3615	IMPDH2	IMP (inosine 5'-monophosphate) dehydrogenase 2										
30833	NT5C	5', 3'-nucleotidase, cytosolic										
1716	DGUOK	deoxyguanosine kinase										
27343	POLL	polymerase (DNA directed), lambda										
30834	ZNRD1	zinc ribbon domain containing 1										
124583	CANT1	calcium activated nucleotidase 1										
9013	TAF1C	TATA box binding protein (TBP)-associated factor, RNA polymerase I, C, 110kDa										
9328	GTF3C5	general transcription factor IIIC, polypeptide 5, 63kDa										
10302	SNAPC5	small nuclear RNA activating complex, polypeptide 5, 19kDa										
5898	RALA	v-ral simian leukemia viral oncogene homolog A (ras related)										
286075	ZNF707	zinc finger protein 707										
11184	MAP4K1	mitogen-activated protein kinase kinase kinase kinase 1										
6892	TAPBP	TAP binding protein (tapasin)										
586	BCAT1	branched chain amino-acid transaminase 1, cytosolic										
3594	IL12RB1	interleukin 12 receptor, beta 1										
4528	MTIF2	mitochondrial translational initiation factor 2										
11331	PHB2	prohibitin 2										
3183	HNRNPC	heterogeneous nuclear ribonucleoprotein C (C1/C2)										
5478	PPIA	peptidylprolyl isomerase A (cyclophilin A)										
10987	COPS5	COP9 constitutive photomorphogenic homolog subunit 5 (Arabidopsis)										
1665	DHX15	DEAH (Asp-Glu-Ala-His) box polypeptide 15										
708	C1QBP	complement component 1, q subcomponent binding protein										
7965	AIMP2	aminoacyl tRNA synthetase complex-interacting multifunctional protein 2										
2079	ERH	enhancer of rudimentary homolog (Drosophila)										
6732	SRPK1	SRSF protein kinase 1										
8761	PABPC4	poly(A) binding protein, cytoplasmic 4 (inducible form)										
4194	MDM4	Mdm4 p53 binding protein homolog (mouse)										
1616	DAXX	death-domain associated protein										
80854	SETD7	SET domain containing (lysine methyltransferase) 7										
1877	E4F1	E4F transcription factor 1										
7029	TFDP2	transcription factor Dp-2 (E2F dimerization partner 2)										
29904	EEF2K	eukaryotic elongation factor-2 kinase										
5562	PRKAA1	protein kinase, AMP-activated, alpha 1 catalytic subunit										
5494	PPM1A	protein phosphatase, Mg2+/Mn2+ dependent, 1A										
933	CD22	CD22 molecule										
5452	POU2F2	POU class 2 homeobox 2										
22978	NT5C2	5'-nucleotidase, cytosolic II										
8833	GMPS	guanine monphosphate synthetase										
158	ADSL	adenylosuccinate lyase										
2782	GNB1	guanine nucleotide binding protein (G protein), beta polypeptide 1										
2870	GRK6	G protein-coupled receptor kinase 6										
5996	RGS1	regulator of G-protein signaling 1										
79661	NEIL1	nei endonuclease VIII-like 1 (E. coli)										
4800	NFYA	nuclear transcription factor Y, alpha										
2648	KAT2A	K(lysine) acetyltransferase 2A										
57060	PCBP4	poly(rC) binding protein 4										
171568	POLR3H	polymerase (RNA) III (DNA directed) polypeptide H (22.9kD)										
9583	ENTPD4	ectonucleoside triphosphate diphosphohydrolase 4										
10201	NME6	non-metastatic cells 6, protein expressed in (nucleoside-diphosphate kinase)										
8202	NCOA3	nuclear receptor coactivator 3										
522	ATP5J	ATP synthase, H+ transporting, mitochondrial Fo complex, subunit F6										
9612	NCOR2	nuclear receptor corepressor 2										
57658	CALCOCO1	calcium binding and coiled-coil domain 1										
84458	LCOR	ligand dependent nuclear receptor corepressor										
83549	UCK1	uridine-cytidine kinase 1										
54963	UCKL1	uridine-cytidine kinase 1-like 1										
9785	DHX38	DEAH (Asp-Glu-Ala-His) box polypeptide 38										
29894	CPSF1	cleavage and polyadenylation specific factor 1, 160kDa										
79869	CPSF7	cleavage and polyadenylation specific factor 7, 59kDa										
3190	HNRNPK	heterogeneous nuclear ribonucleoprotein K										
3187	HNRNPH1	heterogeneous nuclear ribonucleoprotein H1 (H)										
3587	IL10RA	interleukin 10 receptor, alpha										
2643	GCH1	GTP cyclohydrolase 1										
37	ACADVL	acyl-CoA dehydrogenase, very long chain										
211	ALAS1	aminolevulinate, delta-, synthase 1										
10312	TCIRG1	T-cell, immune regulator 1, ATPase, H+ transporting, lysosomal V0 subunit A3										
10632	ATP5L	ATP synthase, H+ transporting, mitochondrial Fo complex, subunit G										
9551	ATP5J2	ATP synthase, H+ transporting, mitochondrial Fo complex, subunit F2										
3420	IDH3B	isocitrate dehydrogenase 3 (NAD+) beta										
3419	IDH3A	isocitrate dehydrogenase 3 (NAD+) alpha										
1350	COX7C	cytochrome c oxidase subunit VIIc										
7388	UQCRH	ubiquinol-cytochrome c reductase hinge protein										
4728	NDUFS8	NADH dehydrogenase (ubiquinone) Fe-S protein 8, 23kDa (NADH-coenzyme Q reductase)										
8402	SLC25A11	solute carrier family 25 (mitochondrial carrier; oxoglutarate carrier), member 11										
4976	OPA1	optic atrophy 1 (autosomal dominant)										
2026	ENO2	enolase 2 (gamma, neuronal)										
80349	WDR61	WD repeat domain 61										
84186	ZCCHC7	zinc finger, CCHC domain containing 7										
200576	PIKFYVE	phosphoinositide kinase, FYVE finger containing										
51763	INPP5K	inositol polyphosphate-5-phosphatase K										
5298	PI4KB	phosphatidylinositol 4-kinase, catalytic, beta										
25942	SIN3A	SIN3 homolog A, transcription regulator (yeast)										
31	ACACA	acetyl-CoA carboxylase alpha										
2181	ACSL3	acyl-CoA synthetase long-chain family member 3										
3156	HMGCR	3-hydroxy-3-methylglutaryl-CoA reductase										
51703	ACSL5	acyl-CoA synthetase long-chain family member 5										
64834	ELOVL1	ELOVL fatty acid elongase 1										
10554	AGPAT1	1-acylglycerol-3-phosphate O-acyltransferase 1 (lysophosphatidic acid acyltransferase, alpha)										
9282	MED14	mediator complex subunit 14										
622	BDH1	3-hydroxybutyrate dehydrogenase, type 1										
8398	PLA2G6	phospholipase A2, group VI (cytosolic, calcium-independent)										
134429	STARD4	StAR-related lipid transfer (START) domain containing 4										
3073	HEXA	hexosaminidase A (alpha polypeptide)										
4597	MVD	mevalonate (diphospho) decarboxylase										
1120	CHKB	choline kinase beta										
55500	ETNK1	ethanolamine kinase 1										
122618	PLD4	phospholipase D family, member 4										
56261	GPCPD1	glycerophosphocholine phosphodiesterase GDE1 homolog (S. cerevisiae)										
57704	GBA2	glucosidase, beta (bile acid) 2										
2172	FABP6	fatty acid binding protein 6, ileal										
79644	SRD5A3	steroid 5 alpha-reductase 3										
3708	ITPR1	inositol 1,4,5-trisphosphate receptor, type 1										
8527	DGKD	diacylglycerol kinase, delta 130kDa										
3633	INPP5B	inositol polyphosphate-5-phosphatase, 75kDa										
8932	MBD2	methyl-CpG binding domain protein 2										
10919	EHMT2	euchromatic histone-lysine N-methyltransferase 2										
8815	BANF1	barrier to autointegration factor 1										
55738	ARFGAP1	ADP-ribosylation factor GTPase activating protein 1										
10897	YIF1A	Yip1 interacting factor homolog A (S. cerevisiae)										
1663	DDX11	DEAD/H (Asp-Glu-Ala-Asp/His) box polypeptide 11										
468	ATF4	activating transcription factor 4 (tax-responsive enhancer element B67)										
55869	HDAC8	histone deacetylase 8										
51547	SIRT7	sirtuin 7										
51548	SIRT6	sirtuin 6										
1107	CHD3	chromodomain helicase DNA binding protein 3										
5581	PRKCE	protein kinase C, epsilon										
10818	FRS2	fibroblast growth factor receptor substrate 2										
5923	RASGRF1	Ras protein-specific guanine nucleotide-releasing factor 1										
221472	FGD2	FYVE, RhoGEF and PH domain containing 2										
9138	ARHGEF1	Rho guanine nucleotide exchange factor (GEF) 1										
4855	NOTCH4	notch 4										
27327	TNRC6A	trinucleotide repeat containing 6A										
56983	POGLUT1	protein O-glucosyltransferase 1										
113878	DTX2	deltex homolog 2 (Drosophila)										
984	CDK11B	cyclin-dependent kinase 11B										
6867	TACC1	transforming, acidic coiled-coil containing protein 1										
5727	PTCH1	patched 1										
2319	FLOT2	flotillin 2										
10644	IGF2BP2	insulin-like growth factor 2 mRNA binding protein 2										
10059	DNM1L	dynamin 1-like										
10109	ARPC2	actin related protein 2/3 complex, subunit 2, 34kDa										
55616	ASAP3	ArfGAP with SH3 domain, ankyrin repeat and PH domain 3										
27148	STK36	serine/threonine kinase 36										
9788	MTSS1	metastasis suppressor 1										
2597	GAPDH	glyceraldehyde-3-phosphate dehydrogenase										
5052	PRDX1	peroxiredoxin 1										
8624	PSMG1	proteasome (prosome, macropain) assembly chaperone 1										
6472	SHMT2	serine hydroxymethyltransferase 2 (mitochondrial)										
5993	RFX5	regulatory factor X, 5 (influences HLA class II expression)										
2778	GNAS	GNAS complex locus										
6789	STK4	serine/threonine kinase 4										
2956	MSH6	mutS homolog 6 (E. coli)										
1857	DVL3	dishevelled, dsh homolog 3 (Drosophila)										
10342	TFG	TRK-fused gene										
221955	DAGLB	diacylglycerol lipase, beta										
7813	EVI5	ecotropic viral integration site 5										
54888	NSUN2	NOP2/Sun domain family, member 2										
64151	NCAPG	non-SMC condensin I complex, subunit G										
22985	ACIN1	apoptotic chromatin condensation inducer 1										
151903	CCDC12	coiled-coil domain containing 12										
3692	EIF6	eukaryotic translation initiation factor 6										
55229	PANK4	pantothenate kinase 4										
79717	PPCS	phosphopantothenoylcysteine synthetase										
8875	VNN2	vanin 2										
116984	ARAP2	ArfGAP with RhoGAP domain, ankyrin repeat and PH domain 2										
2665	GDI2	GDP dissociation inhibitor 2										
257106	ARHGAP30	Rho GTPase activating protein 30										
51291	GMIP	GEM interacting protein										
9912	ARHGAP44	Rho GTPase activating protein 44										
51561	IL23A	interleukin 23, alpha subunit p19										
122809	SOCS4	suppressor of cytokine signaling 4										
50615	IL21R	interleukin 21 receptor										
11316	COPE	coatomer protein complex, subunit epsilon										
1315	COPB1	coatomer protein complex, subunit beta 1										
8675	STX16	syntaxin 16										
10067	SCAMP3	secretory carrier membrane protein 3										
11079	RER1	RER1 retention in endoplasmic reticulum 1 homolog (S. cerevisiae)										
23033	DOPEY1	dopey family member 1										
2803	GOLGA4	golgin A4										
5213	PFKM	phosphofructokinase, muscle										
9913	SUPT7L	suppressor of Ty 7 (S. cerevisiae)-like										
10487	CAP1	CAP, adenylate cyclase-associated protein 1 (yeast)										
483	ATP1B3	ATPase, Na+/K+ transporting, beta 3 polypeptide										
81704	DOCK8	dedicator of cytokinesis 8										
4125	MAN2B1	mannosidase, alpha, class 2B, member 1										
4126	MANBA	mannosidase, beta A, lysosomal										
53	ACP2	acid phosphatase 2, lysosomal										
3423	IDS	iduronate 2-sulfatase										
1201	CLN3	ceroid-lipofuscinosis, neuronal 3										
84572	GNPTG	N-acetylglucosamine-1-phosphate transferase, gamma subunit										
252983	STXBP4	syntaxin binding protein 4										
2118	ETV4	ets variant 4										
114790	STK11IP	serine/threonine kinase 11 interacting protein										
11016	ATF7	activating transcription factor 7										
4485	MST1	macrophage stimulating 1 (hepatocyte growth factor-like)										
5871	MAP4K2	mitogen-activated protein kinase kinase kinase kinase 2										
23162	MAPK8IP3	mitogen-activated protein kinase 8 interacting protein 3										
27243	CHMP2A	charged multivesicular body protein 2A										
8301	PICALM	phosphatidylinositol binding clathrin assembly protein										
27131	SNX5	sorting nexin 5										
80308	FLAD1	FAD1 flavin adenine dinucleotide synthetase homolog (S. cerevisiae)										
65220	NADK	NAD kinase										
2356	FPGS	folylpolyglutamate synthase										
55163	PNPO	pyridoxamine 5'-phosphate oxidase										
3119	HLA-DQB1	major histocompatibility complex, class II, DQ beta 1										
8751	ADAM15	ADAM metallopeptidase domain 15										
4771	NF2	neurofibromin 2 (merlin)										
5310	PKD1	polycystic kidney disease 1 (autosomal dominant)										
25989	ULK3	unc-51-like kinase 3 (C. elegans)										
5106	PCK2	phosphoenolpyruvate carboxykinase 2 (mitochondrial)										
2744	GLS	glutaminase										
23085	ERC1	ELKS/RAB6-interacting/CAST family member 1										
23527	ACAP2	ArfGAP with coiled-coil, ankyrin repeat and PH domains 2										
116986	AGAP2	ArfGAP with GTPase domain, ankyrin repeat and PH domain 2										
10617	STAMBP	STAM binding protein										
80230	RUFY1	RUN and FYVE domain containing 1										
5261	PHKG2	phosphorylase kinase, gamma 2 (testis)										
55066	PDPR	pyruvate dehydrogenase phosphatase regulatory subunit										
4839	NOP2	NOP2 nucleolar protein homolog (yeast)										
10244	RABEPK	Rab9 effector protein with kelch motifs										
3297	HSF1	heat shock transcription factor 1										
3035	HARS	histidyl-tRNA synthetase										
27068	PPA2	pyrophosphatase (inorganic) 2										
57505	AARS2	alanyl-tRNA synthetase 2, mitochondrial (putative)										
1285	COL4A3	collagen, type IV, alpha 3 (Goodpasture antigen)										
51005	AMDHD2	amidohydrolase domain containing 2										
54187	NANS	N-acetylneuraminic acid synthase										
80896	NPL	N-acetylneuraminate pyruvate lyase (dihydrodipicolinate synthase)										
9869	SETDB1	SET domain, bifurcated 1										
83852	SETDB2	SET domain, bifurcated 2										
9712	USP6NL	USP6 N-terminal like										
23590	PDSS1	prenyl (decaprenyl) diphosphate synthase, subunit 1										
4603	MYBL1	v-myb myeloblastosis viral oncogene homolog (avian)-like 1										
55	ACPP	acid phosphatase, prostate										
27434	POLM	polymerase (DNA directed), mu										
11284	PNKP	polynucleotide kinase 3'-phosphatase										
3956	LGALS1	lectin, galactoside-binding, soluble, 1										
10325	RRAGB	Ras-related GTP binding B										
9776	ATG13	ATG13 autophagy related 13 homolog (S. cerevisiae)										
56954	NIT2	nitrilase family, member 2										
5831	PYCR1	pyrroline-5-carboxylate reductase 1										
27034	ACAD8	acyl-CoA dehydrogenase family, member 8										
3712	IVD	isovaleryl-CoA dehydrogenase										
8678	BECN1	beclin 1, autophagy related										
7086	TKT	transketolase										
128338	DRAM2	DNA-damage regulated autophagy modulator 2										
2145	EZH1	enhancer of zeste homolog 1 (Drosophila)										
26268	FBXO9	F-box protein 9										
4779	NFE2L1	nuclear factor (erythroid-derived 2)-like 1										
55589	BMP2K	BMP2 inducible kinase										
56180	MOSPD1	motile sperm domain containing 1										
6397	SEC14L1	SEC14-like 1 (S. cerevisiae)										
51074	APIP	APAF1 interacting protein										
125061	AFMID	arylformamidase										
5830	PEX5	peroxisomal biogenesis factor 5										
5826	ABCD4	ATP-binding cassette, sub-family D (ALD), member 4										
55670	PEX26	peroxisomal biogenesis factor 26										
51024	FIS1	fission 1 (mitochondrial outer membrane) homolog (S. cerevisiae)										
9270	ITGB1BP1	integrin beta 1 binding protein 1										
83660	TLN2	talin 2										
3150	HMGN1	high mobility group nucleosome binding domain 1										
126382	NR2C2AP	nuclear receptor 2C2-associated protein										
162655	ZNF519	zinc finger protein 519										
57474	ZNF490	zinc finger protein 490										

7587	ZNF37A	zinc finger protein 37A										
79027	ZNF655	zinc finger protein 655										
84124	ZNF394	zinc finger protein 394										
90594	ZNF439	zinc finger protein 439										
93134	ZNF561	zinc finger protein 561										
490	ATP2B1	ATPase, Ca++ transporting, plasma membrane 1										
22797	TFEC	transcription factor EC										
10327	AKR1A1	aldo-keto reductase family 1, member A1 (aldehyde reductase)										
11315	PARK7	parkinson protein 7										
4796	TONSL	tonsoku-like, DNA repair protein										
9941	EXOG	endo/exonuclease (5'-3'), endonuclease G-like										
4311	MME	membrane metallo-endopeptidase										
951	CD37	CD37 molecule										
931	MS4A1	membrane-spanning 4-domains, subfamily A, member 1										
55454	CSGALNACT2	chondroitin sulfate N-acetylgalactosaminyltransferase 2										
4637	MYL6	myosin, light chain 6, alkali, smooth muscle and non-muscle										
64333	ARHGAP9	Rho GTPase activating protein 9										
4052	LTBP1	latent transforming growth factor beta binding protein 1										
22884	WDR37	WD repeat domain 37										
26037	SIPA1L1	signal-induced proliferation-associated 1 like 1										
27109	ATP5S	ATP synthase, H+ transporting, mitochondrial Fo complex, subunit s (factor B)										
3069	HDLBP	high density lipoprotein binding protein										
55139	ANKZF1	ankyrin repeat and zinc finger domain containing 1										
10957	PNRC1	proline-rich nuclear receptor coactivator 1										
7511	XPNPEP1	X-prolyl aminopeptidase (aminopeptidase P) 1, soluble										
10994	ILVBL	ilvB (bacterial acetolactate synthase)-like										
9144	SYNGR2	synaptogyrin 2										
9910	RABGAP1L	RAB GTPase activating protein 1-like										
1690	COCH	coagulation factor C homolog, cochlin (Limulus polyphemus)										
57231	SNX14	sorting nexin 14										
2014	EMP3	epithelial membrane protein 3										
9260	PDLIM7	PDZ and LIM domain 7 (enigma)										
1760	DMPK	dystrophia myotonica-protein kinase										
6525	SMTN	smoothelin										
7516	XRCC2	X-ray repair complementing defective repair in Chinese hamster cells 2										
51742	ARID4B	AT rich interactive domain 4B (RBP1-like)										
286410	ATP11C	ATPase, class VI, type 11C										
374868	ATP9B	ATPase, class II, type 9B										
6601	SMARCC2	SWI/SNF related, matrix associated, actin dependent regulator of chromatin, subfamily c, member 2										
10347	ABCA7	ATP-binding cassette, sub-family A (ABC1), member 7										
2030	SLC29A1	solute carrier family 29 (nucleoside transporters), member 1										
29988	SLC2A8	solute carrier family 2 (facilitated glucose transporter), member 8										
6560	SLC12A4	solute carrier family 12 (potassium/chloride transporters), member 4										
10559	SLC35A1	solute carrier family 35 (CMP-sialic acid transporter), member A1										
121260	SLC15A4	solute carrier family 15, member 4										
55630	SLC39A4	solute carrier family 39 (zinc transporter), member 4										
10380	BPNT1	3'(2'), 5'-bisphosphate nucleotidase 1										
143187	VTI1A	vesicle transport through interaction with t-SNAREs homolog 1A (yeast)										
203062	TSNARE1	t-SNARE domain containing 1										
7391	USF1	upstream transcription factor 1										
267	AMFR	autocrine motility factor receptor										
54020	SLC37A1	solute carrier family 37 (glycerol-3-phosphate transporter), member 1										
9094	UNC119	unc-119 homolog (C. elegans)										
8498	RANBP3	RAN binding protein 3										
340481	ZDHHC21	zinc finger, DHHC-type containing 21										
4093	SMAD9	SMAD family member 9										
89845	ABCC10	ATP-binding cassette, sub-family C (CFTR/MRP), member 10										
6935	ZEB1	zinc finger E-box binding homeobox 1										
80895	ILKAP	integrin-linked kinase-associated serine/threonine phosphatase										
1390	CREM	cAMP responsive element modulator										
7168	TPM1	tropomyosin 1 (alpha)										
5959	RDH5	retinol dehydrogenase 5 (11-cis/9-cis)										
59339	PLEKHA2	pleckstrin homology domain containing, family A (phosphoinositide binding specific) member 2										
2595	GANC	glucosidase, alpha; neutral C										
114049	WBSCR22	Williams Beuren syndrome chromosome region 22										
60487	TRMT11	tRNA methyltransferase 11 homolog (S. cerevisiae)										
8630	HSD17B6	hydroxysteroid (17-beta) dehydrogenase 6 homolog (mouse)										
27334	P2RY10	purinergic receptor P2Y, G-protein coupled, 10										
5051	PAFAH2	platelet-activating factor acetylhydrolase 2, 40kDa										
55914	ERBB2IP	erbb2 interacting protein										
10219	KLRG1	killer cell lectin-like receptor subfamily G, member 1										
10629	TAF6L	TAF6-like RNA polymerase II, p300/CBP-associated factor (PCAF)-associated factor, 65kDa										
2969	GTF2I	general transcription factor IIi										
7392	USF2	upstream transcription factor 2, c-fos interacting										
83734	ATG10	ATG10 autophagy related 10 homolog (S. cerevisiae)										
654817	NCF1C	neutrophil cytosolic factor 1C pseudogene										
4008	LMO7	LIM domain 7										
4122	MAN2A2	mannosidase, alpha, class 2A, member 2										
25776	CBY1	chibby homolog 1 (Drosophila)										
4155	MBP	myelin basic protein										
3782	KCNN3	potassium intermediate/small conductance calcium-activated channel, subfamily N, member 3										
240	ALOX5	arachidonate 5-lipoxygenase										
79017	GGCT	gamma-glutamylcyclotransferase										
55610	CCDC132	coiled-coil domain containing 132										
8115	TCL1A	T-cell leukemia/lymphoma 1A										
29121	CLEC2D	C-type lectin domain family 2, member D										
64581	CLEC7A	C-type lectin domain family 7, member A										
327657	SERPINA9	serpin peptidase inhibitor, clade A (alpha-1 antiproteinase, antitrypsin), member 9										
5176	SERPINF1	serpin peptidase inhibitor, clade F (alpha-2 antiplasmin, pigment epithelium derived factor), member 1										
79676	OGFOD2	2-oxoglutarate and iron-dependent oxygenase domain containing 2										
1954	MEGF8	multiple EGF-like-domains 8										
2329	FMO4	flavin containing monooxygenase 4										
4026	LPP	LIM domain containing preferred translocation partner in lipoma										
100129460	DPY19L1P1	dpy-19-like 1 pseudogene 1 (C. elegans)										
100289678	ZNF783	zinc finger family member 783										
100302692	FTX	FTX transcript, XIST regulator (non-protein coding)										
10069	RWDD2B	RWD domain containing 2B										
10101	NUBP2	nucleotide binding protein 2										
10138	YAF2	YY1 associated factor 2										
10169	SERF2	small EDRK-rich factor 2										
10198	MPHOSPH9	M-phase phosphoprotein 9										
10227	MFSD10	major facilitator superfamily domain containing 10										
10230	NBR2	neighbor of BRCA1 gene 2 (non-protein coding)										
10346	TRIM22	tripartite motif containing 22										
10428	CFDP1	craniofacial development protein 1										
10444	ZER1	zer-1 homolog (C. elegans)										
10539	GLRX3	glutaredoxin 3										
10625	IVNS1ABP	influenza virus NS1A binding protein										
10712	FAM189B	family with sequence similarity 189, member B										
10745	PHTF1	putative homeodomain transcription factor 1										
10807	SDCCAG3	serologically defined colon cancer antigen 3										
10865	ARID5A	AT rich interactive domain 5A (MRF1-like)										
10902	BRD8	bromodomain containing 8										
10922	FASTK	Fas-activated serine/threonine kinase										
10980	COPS6	COP9 constitutive photomorphogenic homolog subunit 6 (Arabidopsis)										
11020	IFT27	intraflagellar transport 27 homolog (Chlamydomonas)										
11177	BAZ1A	bromodomain adjacent to zinc finger domain, 1A										
11201	POLI	polymerase (DNA directed) iota										
11219	TREX2	three prime repair exonuclease 2										
112479	ERI2	ERI1 exoribonuclease family member 2										
112616	CMTM7	CKLF-like MARVEL transmembrane domain containing 7										
11275	KLHL2	kelch-like 2, Mayven (Drosophila)										
113000	RPUSD1	RNA pseudouridylate synthase domain containing 1										
113177	IZUMO4	IZUMO family member 4										
11322	TMC6	transmembrane channel-like 6										
114659	LRRC37B	leucine rich repeat containing 37B										
114800	CCDC85A	coiled-coil domain containing 85A										
114803	MYSM1	Myb-like, SWIRM and MPN domains 1										
114825	PWWP2A	PWWP domain containing 2A										
114882	OSBPL8	oxysterol binding protein-like 8										
114883	OSBPL9	oxysterol binding protein-like 9										
115209	OMA1	OMA1 homolog, zinc metallopeptidase (S. cerevisiae)										
115294	PCMTD1	protein-L-isoaspartate (D-aspartate) O-methyltransferase domain containing 1										
1153	CIRBP	cold inducible RNA binding protein										
115353	LRRC42	leucine rich repeat containing 42										
115399	LRRC56	leucine rich repeat containing 56										
115426	UHRF2	ubiquitin-like with PHD and ring finger domains 2										
116931	MED12L	mediator complex subunit 12-like										
117177	RAB3IP	RAB3A interacting protein (rabin3)										
118813	ZFYVE27	zinc finger, FYVE domain containing 27										
118980	SFXN2	sideroflexin 2										
119504	ANAPC16	anaphase promoting complex subunit 16										
122553	TRAPPC6B	trafficking protein particle complex 6B										
123803	NTAN1	N-terminal asparagine amidase										
124093	CCDC78	coiled-coil domain containing 78										
124401	ANKS3	ankyrin repeat and sterile alpha motif domain containing 3										
124512	METTL23	methyltransferase like 23										
124944	C17orf49	chromosome 17 open reading frame 49										
125150	ZSWIM7	zinc finger, SWIM-type containing 7										
125476	INO80C	INO80 complex subunit C										
126075	CCDC159	coiled-coil domain containing 159										
126133	ALDH16A1	aldehyde dehydrogenase 16 family, member A1										
126526	C19orf47	chromosome 19 open reading frame 47										
128308	MRPL55	mitochondrial ribosomal protein L55										
130872	AHSA2	AHA1, activator of heat shock 90kDa protein ATPase homolog 2 (yeast)										
132299	OCIAD2	OCIA domain containing 2										
136051	ZNF786	zinc finger protein 786										
137695	TMEM68	transmembrane protein 68										
140890	SREK1	splicing regulatory glutamine/lysine-rich protein 1										
144132	DNHD1	dynein heavy chain domain 1										
144348	ZNF664	zinc finger protein 664										
145389	SLC38A6	solute carrier family 38, member 6										
145773	FAM81A	family with sequence similarity 81, member A										
146059	CDAN1	congenital dyserythropoietic anemia, type I										
146206	RLTPR	RGD motif, leucine rich repeats, tropomodulin domain and proline-rich containing										
146857	SLFN13	schlafen family member 13										
147138	TMC8	transmembrane channel-like 8										
148206	ZNF714	zinc finger protein 714										
149175	MANEAL	mannosidase, endo-alpha-like										
149775	GNAS-AS1	GNAS antisense RNA 1 (non-protein coding)										
150223	YDJC	YdjC homolog (bacterial)										
150472	CBWD2	COBW domain containing 2										
150726	FBXO41	F-box protein 41										
150962	PUS10	pseudouridylate synthase 10										
151188	ARL6IP6	ADP-ribosylation-like factor 6 interacting protein 6										
151194	METTL21A	methyltransferase like 21A										
151613	TTC14	tetratricopeptide repeat domain 14										
151827	LRRC34	leucine rich repeat containing 34										
152485	ZNF827	zinc finger protein 827										
153129	SLC38A9	solute carrier family 38, member 9										
157657	C8orf37	chromosome 8 open reading frame 37										
158056	MAMDC4	MAM domain containing 4										
158405	KIAA1958	KIAA1958										
158584	FAAH2	fatty acid amide hydrolase 2										
161582	DYX1C1	dyslexia susceptibility 1 candidate 1										
163486	DENND1B	DENN/MADD domain containing 1B										
164118	TTC24	tetratricopeptide repeat domain 24										
166824	RASSF6	Ras association (RalGDS/AF-6) domain family member 6										
171017	ZNF384	zinc finger protein 384										
171586	ABHD3	abhydrolase domain containing 3										
1912	PHC2	polyhomeotic homolog 2 (Drosophila)										
197320	ZNF778	zinc finger protein 778										
200844	C3orf67	chromosome 3 open reading frame 67										
201254	STRA13	stimulated by retinoic acid 13 homolog (mouse)										
201292	TRIM65	tripartite motif containing 65										
2029	ENSA	endosulfine alpha										
203328	SUSD3	sushi domain containing 3										
2077	ERF	Ets2 repressor factor										
2140	EYA3	eyes absent homolog 3 (Drosophila)										
222068	TMED4	transmembrane emp24 protein transport domain containing 4										
222229	LRWD1	leucine-rich repeats and WD repeat domain containing 1										
22864	R3HDM2	R3H domain containing 2										
22883	CLSTN1	calsyntenin 1										
22902	RUFY3	RUN and FYVE domain containing 3										
22907	DHX30	DEAH (Asp-Glu-Ala-His) box polypeptide 30										
22990	PCNX	pecanex homolog (Drosophila)										
23023	TMCC1	transmembrane and coiled-coil domain family 1										
23032	USP33	ubiquitin specific peptidase 33										
23064	SETX	senataxin										
23077	MYCBP2	MYC binding protein 2										
23093	TTLL5	tubulin tyrosine ligase-like family, member 5										
23113	CUL9	cullin 9										
23155	CLCC1	chloride channel CLIC-like 1										
23164	MPRIP	myosin phosphatase Rho interacting protein										
23180	RFTN1	raftlin, lipid raft linker 1										
23181	DIP2A	DIP2 disco-interacting protein 2 homolog A (Drosophila)										
23189	KANK1	KN motif and ankyrin repeat domains 1										
23215	PRRC2C	proline-rich coiled-coil 2C										
23243	ANKRD28	ankyrin repeat domain 28										
23244	PDS5A	PDS5, regulator of cohesion maintenance, homolog A (S. cerevisiae)										
23247	KIAA0556	KIAA0556										
23253	ANKRD12	ankyrin repeat domain 12										
23269	MGA	MAX gene associated										
23274	CLEC16A	C-type lectin domain family 16, member A										
23279	NUP160	nucleoporin 160kDa										
23318	ZCCHC11	zinc finger, CCHC domain containing 11										
2334	AFF2	AF4/FMR2 family, member 2										
23341	DNAJC16	DnaJ (Hsp40) homolog, subfamily C, member 16										
23353	SUN1	Sad1 and UNC84 domain containing 1										
23354	HAUS5	HAUS augmin-like complex, subunit 5										
23355	VPS8	vacuolar protein sorting 8 homolog (S. cerevisiae)										
23367	LARP1	La ribonucleoprotein domain family, member 1										
23392	KIAA0368	KIAA0368										
23394	ADNP	activity-dependent neuroprotector homeobox										
23400	ATP13A2	ATPase type 13A2										
23518	R3HDM1	R3H domain containing 1										
23559	WBP1	WW domain binding protein 1										
23597	ACOT9	acyl-CoA thioesterase 9										
23613	ZMYND8	zinc finger, MYND-type containing 8										
23642	SNHG1	small nucleolar RNA host gene 1 (non-protein coding)										
23787	MTCH1	mitochondrial carrier 1										
24139	EML2	echinoderm microtubule associated protein like 2										
253943	YTHDF3	YTH domain family, member 3										
255119	C4orf22	chromosome 4 open reading frame 22										
255394	TCP11L2	t-complex 11 (mouse)-like 2										
256364	EML3	echinoderm microtubule associated protein like 3										
257160	RNF214	ring finger protein 214										
25864	ABHD14A	abhydrolase domain containing 14A										
25875	LETMD1	LETM1 domain containing 1										
25912	C1orf43	chromosome 1 open reading frame 43										
25926	NOL11	nucleolar protein 11										
259266	ASPM	asp (abnormal spindle) homolog, microcephaly associated (Drosophila)										
26015	RPAP1	RNA polymerase II associated protein 1										
260294	NSUN5P2	NOP2/Sun domain family, member 5 pseudogene 2										
26036	ZNF451	zinc finger protein 451										
26046	LTN1	listerin E3 ubiquitin protein ligase 1										
26054	SENP6	SUMO1/sentrin specific peptidase 6										
26065	LSM14A	LSM14A, SCD6 homolog A (S. cerevisiae)										
26123	TCTN3	tectonic family member 3										
26140	TTLL3	tubulin tyrosine ligase-like family, member 3										
26151	NAT9	N-acetyltransferase 9 (GCN5-related, putative)										
26205	GMEB2	glucocorticoid modulatory element binding protein 2										
26233	FBXL6	F-box and leucine-rich repeat protein 6										
26297	SERGEF	secretion regulating guanine nucleotide exchange factor										
27037	TRMT2A	TRM2 tRNA methyltransferase 2 homolog A (S. cerevisiae)										
27158	NDOR1	NADPH dependent diflavin oxidoreductase 1										
27315	PGAP2	post-GPI attachment to proteins 2										
27341	RRP7A	ribosomal RNA processing 7 homolog A (S. cerevisiae)										
27352	SGSM3	small G protein signaling modulator 3										
283234	CCDC88B	coiled-coil domain containing 88B										
283373	ANKRD52	ankyrin repeat domain 52										

283596	SNHG10	small nucleolar RNA host gene 10 (non-protein coding)										
283899	INO80E	INO80 complex subunit E										
283970	PDXDC2P	pyridoxal-dependent decarboxylase domain containing 2, pseudogene										
283989	TSEN54	tRNA splicing endonuclease 54 homolog (S. cerevisiae)										
284004	HEXDC	hexosaminidase (glycosyl hydrolase family 20, catalytic domain) containing										
284403	WDR62	WD repeat domain 62										
284992	CCDC150	coiled-coil domain containing 150										
285331	CCDC66	coiled-coil domain containing 66										
28638	TRBC2	T cell receptor beta constant 2										
2873	GPS1	G protein pathway suppressor 1										
28977	MRPL42	mitochondrial ribosomal protein L42										
29066	ZC3H7A	zinc finger CCCH-type containing 7A										
29087	THYN1	thymocyte nuclear protein 1										
29091	STXBP6	syntaxin binding protein 6 (amisyn)										
29098	RANGRF	RAN guanine nucleotide release factor										
29104	N6AMT1	N-6 adenine-specific DNA methyltransferase 1 (putative)										
29117	BRD7	bromodomain containing 7										
29123	ANKRD11	ankyrin repeat domain 11										
29128	UHRF1	ubiquitin-like with PHD and ring finger domains 1										
29789	OLA1	Obg-like ATPase 1										
29803	REPIN1	replication initiator 1										
29888	STRN4	striatin, calmodulin binding protein 4										
29952	DPP7	dipeptidyl-peptidase 7										
29954	POMT2	protein-O-mannosyltransferase 2										
29966	STRN3	striatin, calmodulin binding protein 3										
317662	FAM149B1	family with sequence similarity 149, member B1										
317781	DDX51	DEAD (Asp-Glu-Ala-Asp) box polypeptide 51										
3257	HPS1	Hermansky-Pudlak syndrome 1										
327	APEH	N-acylaminoacyl-peptide hydrolase										
3338	DNAJC4	DnaJ (Hsp40) homolog, subfamily C, member 4										
338692	ANKRD13D	ankyrin repeat domain 13 family, member D										
339123	JMJD8	jumonji domain containing 8										
339451	KLHL17	kelch-like 17 (Drosophila)										
339487	ZBTB8OS	zinc finger and BTB domain containing 8 opposite strand										
339500	ZNF678	zinc finger protein 678										
340252	ZNF680	zinc finger protein 680										
343099	CCDC18	coiled-coil domain containing 18										
345778	MTX3	metaxin 3										
346389	MACC1	metastasis associated in colon cancer 1										
347862	PDDC1	Parkinson disease 7 domain containing 1										
349152	DPY19L2P2	dpy-19-like 2 pseudogene 2 (C. elegans)										
353497	POLN	polymerase (DNA directed) nu										
3607	FOXK2	forkhead box K2										
374618	TEX9	testis expressed 9										
374659	HDDC3	HD domain containing 3										
375248	ANKRD36	ankyrin repeat domain 36										
376267	RAB15	RAB15, member RAS onocogene family										
378938	MALAT1	metastasis associated lung adenocarcinoma transcript 1 (non-protein coding)										
386593	CHKB-CPT1B	CHKB-CPT1B readthrough (non-protein coding)										
388561	ZNF761	zinc finger protein 761										
389677	RBM12B	RNA binding motif protein 12B										
3899	AFF3	AF4/FMR2 family, member 3										
390980	ZNF805	zinc finger protein 805										
400818	NBPF9	neuroblastoma breakpoint family, member 9										
400954	EML6	echinoderm microtubule associated protein like 6										
404636	FAM45A	family with sequence similarity 45, member A										
4152	MBD1	methyl-CpG binding domain protein 1										
4154	MBNL1	muscleblind-like (Drosophila)										
4253	CTAGE5	CTAGE family, member 5										
439	ASNA1	arsA arsenite transporter, ATP-binding, homolog 1 (bacterial)										
440400	RNASEK	ribonuclease, RNase K										
440590	ZYG11A	zyg-11 homolog A (C. elegans)										
4515	MTCP1	mature T-cell proliferation 1										
4649	MYO9A	myosin IXA										
4664	NAB1	NGFI-A binding protein 1 (EGR1 binding protein 1)										
4676	NAP1L4	nucleosome assembly protein 1-like 4										
4861	NPAS1	neuronal PAS domain protein 1										
494143	CHAC2	ChaC, cation transport regulator homolog 2 (E. coli)										
4943	TBC1D25	TBC1 domain family, member 25										
497661	C18orf32	chromosome 18 open reading frame 32										
50488	MINK1	misshapen-like kinase 1										
51076	CUTC	cutC copper transporter homolog (E. coli)										
51088	KLHL5	kelch-like 5 (Drosophila)										
51093	RRNAD1	ribosomal RNA adenine dimethylase domain containing 1										
5110	PCMT1	protein-L-isoaspartate (D-aspartate) O-methyltransferase										
51133	KCTD3	potassium channel tetramerisation domain containing 3										
51175	TUBE1	tubulin, epsilon 1										
51230	PHF20	PHD finger protein 20										
51239	ANKRD39	ankyrin repeat domain 39										
51249	TMEM69	transmembrane protein 69										
5127	CDK16	cyclin-dependent kinase 16										
51279	C1RL	complement component 1, r subcomponent-like										
51290	ERGIC2	ERGIC and golgi 2										
51329	ARL6IP4	ADP-ribosylation-like factor 6 interacting protein 4										
51517	NCKIPSD	NCK interacting protein with SH3 domain										
51524	TMEM138	transmembrane protein 138										
51559	NT5DC3	5'-nucleotidase domain containing 3										
51574	LARP7	La ribonucleoprotein domain family, member 7										
51594	NBAS	neuroblastoma amplified sequence										
51629	SLC25A39	solute carrier family 25, member 39										
51654	CDK5RAP1	CDK5 regulatory subunit associated protein 1										
51699	VPS29	vacuolar protein sorting 29 homolog (S. cerevisiae)										
51710	ZNF44	zinc finger protein 44										
5253	PHF2	PHD finger protein 2										
5379	PMS2P1	postmeiotic segregation increased 2 pseudogene 1										
53827	FXYD5	FXYD domain containing ion transport regulator 5										
5428	POLG	polymerase (DNA directed), gamma										
54431	DNAJC10	DnaJ (Hsp40) homolog, subfamily C, member 10										
54476	RNF216	ring finger protein 216										
54477	PLEKHA5	pleckstrin homology domain containing, family A member 5										
54495	TMX3	thioredoxin-related transmembrane protein 3										
54520	CCDC93	coiled-coil domain containing 93										
54540	FAM193B	family with sequence similarity 193, member B										
54552	GNL3L	guanine nucleotide binding protein-like 3 (nucleolar)-like										
54584	GNB1L	guanine nucleotide binding protein (G protein), beta polypeptide 1-like										
54620	FBXL19	F-box and leucine-rich repeat protein 19										
54662	TBC1D13	TBC1 domain family, member 13										
54758	KLHDC4	kelch domain containing 4										
54851	ANKRD49	ankyrin repeat domain 49										
54853	WDR55	WD repeat domain 55										
54856	GON4L	gon-4-like (C. elegans)										
54867	TMEM214	transmembrane protein 214										
54876	DCAF16	DDB1 and CUL4 associated factor 16										
54878	DPP8	dipeptidyl-peptidase 8										
54883	CWC25	CWC25 spliceosome-associated protein homolog (S. cerevisiae)										
54892	NCAPG2	non-SMC condensin II complex, subunit G2										
54899	PXK	PX domain containing serine/threonine kinase										
54902	TTC19	tetratricopeptide repeat domain 19										
54940	OCIAD1	OCIA domain containing 1										
54976	C20orf27	chromosome 20 open reading frame 27										
54986	ULK4	unc-51-like kinase 4 (C. elegans)										
55002	TMCO3	transmembrane and coiled-coil domains 3										
55011	PIH1D1	PIH1 domain containing 1										
55041	PLEKHB2	pleckstrin homology domain containing, family B (evectins) member 2										
55054	ATG16L1	ATG16 autophagy related 16-like 1 (S. cerevisiae)										
55084	SOBP	sine oculis binding protein homolog (Drosophila)										
55133	SRBD1	S1 RNA binding domain 1										
55147	RBM23	RNA binding motif protein 23										
55152	DALRD3	DALR anticodon binding domain containing 3										
55154	MSTO1	misato homolog 1 (Drosophila)										
55159	RFWD3	ring finger and WD repeat domain 3										
55161	TMEM33	transmembrane protein 33										
55183	RIF1	RAP1 interacting factor homolog (yeast)										
55186	SLC25A36	solute carrier family 25, member 36										
55209	SETD5	SET domain containing 5										
55218	EXD2	exonuclease 3'-5' domain containing 2										
55291	PPP6R3	protein phosphatase 6, regulatory subunit 3										
55322	C5orf22	chromosome 5 open reading frame 22										
55334	SLC39A9	solute carrier family 39 (zinc transporter), member 9										
55370	PPP4R1L	protein phosphatase 4, regulatory subunit 1-like										
554203	JPX	JPX transcript, XIST activator (non-protein coding)										
55435	AP1AR	adaptor-related protein complex 1 associated regulatory protein										
55486	PARL	presenilin associated, rhomboid-like										
55508	SLC35E3	solute carrier family 35, member E3										
55536	CDCA7L	cell division cycle associated 7-like										
55552	ZNF823	zinc finger protein 823										
55565	ZNF821	zinc finger protein 821										
55582	KIF27	kinesin family member 27										
55608	ANKRD10	ankyrin repeat domain 10										
55621	TRMT1	TRM1 tRNA methyltransferase 1 homolog (S. cerevisiae)										
55655	NLRP2	NLR family, pyrin domain containing 2										
55657	ZNF692	zinc finger protein 692										
55658	RNF126	ring finger protein 126										
55665	URGCP	upregulator of cell proliferation										
55689	YEATS2	YEATS domain containing 2										
55692	LUC7L	LUC7-like (S. cerevisiae)										
55716	LMBR1L	limb region 1 homolog (mouse)-like										
55728	N4BP2	NEDD4 binding protein 2										
55739	CARKD	carbohydrate kinase domain containing										
55756	INTS9	integrator complex subunit 9										
55761	TTC17	tetratricopeptide repeat domain 17										
55778	ZNF839	zinc finger protein 839										
55827	DCAF6	DDB1 and CUL4 associated factor 6										
55860	ACTR10	actin-related protein 10 homolog (S. cerevisiae)										
55871	CBWD1	COBW domain containing 1										
55892	MYNN	myoneurin										
5612	PRKRIR	protein-kinase, interferon-inducible double stranded RNA dependent inhibitor, repressor of (P58 repressor)										
56311	ANKRD7	ankyrin repeat domain 7										
5639	PRRG2	proline rich Gla (G-carboxyglutamic acid) 2										
56681	SAR1A	SAR1 homolog A (S. cerevisiae)										
56850	GRIPAP1	GRIP1 associated protein 1										
56943	ENY2	enhancer of yellow 2 homolog (Drosophila)										
56948	SDR39U1	short chain dehydrogenase/reductase family 39U, member 1										
56996	SLC12A9	solute carrier family 12 (potassium/chloride transporters), member 9										
57175	CORO1B	coronin, actin binding protein, 1B										
57179	KIAA1191	KIAA1191										
57407	NMRAL1	NmrA-like family domain containing 1										
57409	MIF4GD	MIF4G domain containing										
57410	SCYL1	SCY1-like 1 (S. cerevisiae)										
57507	ZNF608	zinc finger protein 608										
57531	HACE1	HECT domain and ankyrin repeat containing, E3 ubiquitin protein ligase 1										
57560	IFT80	intraflagellar transport 80 homolog (Chlamydomonas)										
57634	EP400	E1A binding protein p400										
57646	USP28	ubiquitin specific peptidase 28										
57699	CPNE5	copine V										
57705	WDFY4	WDFY family member 4										
57724	EPG5	ectopic P-granules autophagy protein 5 homolog (C. elegans)										
58516	FAM60A	family with sequence similarity 60, member A										
5919	RARRES2	retinoic acid receptor responder (tazarotene induced) 2										
59286	UBL5	ubiquitin-like 5										
5929	RBBP5	retinoblastoma binding protein 5										
60343	FAM3A	family with sequence similarity 3, member A										
60468	BACH2	BTB and CNC homology 1, basic leucine zipper transcription factor 2										
60558	GUF1	GUF1 GTPase homolog (S. cerevisiae)										
60674	GAS5	growth arrest-specific 5 (non-protein coding)										
6388	SDF2	stromal cell-derived factor 2										
63892	THADA	thyroid adenoma associated										
63910	SLC17A9	solute carrier family 17, member 9										
63922	CHTF18	CTF18, chromosome transmission fidelity factor 18 homolog (S. cerevisiae)										
64005	MYO1G	myosin IG										
64118	DUS1L	dihydrouridine synthase 1-like (S. cerevisiae)										
641590	NBPF20	neuroblastoma breakpoint family, member 20										
64210	MMS19	MMS19 nucleotide excision repair homolog (S. cerevisiae)										
64417	C5orf28	chromosome 5 open reading frame 28										
64427	TTC31	tetratricopeptide repeat domain 31										
64432	MRPS25	mitochondrial ribosomal protein S25										
645784	ANKRD36BP2	ankyrin repeat domain 36B pseudogene 2										
64601	VPS16	vacuolar protein sorting 16 homolog (S. cerevisiae)										
64708	COPS7B	COP9 constitutive photomorphogenic homolog subunit 7B (Arabidopsis)										
64769	MEAF6	MYST/Esa1-associated factor 6										
64771	C6orf106	chromosome 6 open reading frame 106										
64780	MICAL1	microtubule associated monoxygenase, calponin and LIM domain containing 1										
64848	YTHDC2	YTH domain containing 2										
65056	GPBP1	GC-rich promoter binding protein 1										
65083	NOL6	nucleolar protein family 6 (RNA-associated)										
65244	SPATS2	spermatogenesis associated, serine-rich 2										
65980	BRD9	bromodomain containing 9										
65981	CAPRIN2	caprin family member 2										
66036	MTMR9	myotubularin related protein 9										
6660	SOX5	SRY (sex determining region Y)-box 5										
6813	STXBP2	syntaxin binding protein 2										
6835	SURF2	surfeit 2										
6992	PPP1R11	protein phosphatase 1, regulatory (inhibitor) subunit 11										
7072	TIA1	TIA1 cytotoxic granule-associated RNA binding protein										
7109	TRAPPC10	trafficking protein particle complex 10										
724102	SNHG4	small nucleolar RNA host gene 4 (non-protein coding)										
728013	CBWD7	COBW domain containing 7										
7444	VRK2	vaccinia related kinase 2										
755	C21orf2	chromosome 21 open reading frame 2										
7629	ZNF76	zinc finger protein 76										
7690	ZNF131	zinc finger protein 131										
7705	ZNF146	zinc finger protein 146										
7748	ZNF195	zinc finger protein 195										
78996	C7orf49	chromosome 7 open reading frame 49										
79009	DDX50	DEAD (Asp-Glu-Ala-Asp) box polypeptide 50										
79065	ATG9A	ATG9 autophagy related 9 homolog A (S. cerevisiae)										
79101	TAF1D	TATA box binding protein (TBP)-associated factor, RNA polymerase I, D, 41kDa										
79145	CHCHD7	coiled-coil-helix-coiled-coil-helix domain containing 7										
79157	MFSD11	major facilitator superfamily domain containing 11										
7916	PRRC2A	proline-rich coiled-coil 2A										
7917	BAG6	BCL2-associated athanogene 6										
79180	EFHD2	EF-hand domain family, member D2										
79568	C2orf47	chromosome 2 open reading frame 47										
7957	EPM2A	epilepsy, progressive myoclonus type 2A, Lafora disease (laforin)										
79600	TCTN1	tectonic family member 1										
79634	SCRN3	secernin 3										
79675	FASTKD1	FAST kinase domains 1										
79691	QTRTD1	queuine tRNA-ribosyltransferase domain containing 1										
79706	PRKRIP1	PRKR interacting protein 1 (IL11 inducible)										
79712	GTDC1	glycosyltransferase-like domain containing 1										
79823	CAMKMT	calmodulin-lysine N-methyltransferase										
79828	METTL8	methyltransferase like 8										
79830	ZMYM1	zinc finger, MYM-type 1										
79856	SNX22	sorting nexin 22										
79893	GGNBP2	gametogenetin binding protein 2										
79930	DOK3	docking protein 3										
79934	ADCK4	aarF domain containing kinase 4										
79963	ABCA11P	ATP-binding cassette, sub-family A (ABC1), member 11, pseudogene										
79969	ATAT1	alpha tubulin acetyltransferase 1										
79982	DNAJB14	DnaJ (Hsp40) homolog, subfamily B, member 14										
80011	FAM192A	family with sequence similarity 192, member A										
80013	FAM188A	family with sequence similarity 188, member A										
80018	NAA25	N(alpha)-acetyltransferase 25, NatB auxiliary subunit										
80021	TMEM62	transmembrane protein 62										
80063	ATF7IP2	activating transcription factor 7 interacting protein 2										
80097	MZT2B	mitotic spindle organizing protein 2B										
80178	C16orf59	chromosome 16 open reading frame 59										
80179	MYO19	myosin XIX										
80199	FUZ	fuzzy homolog (Drosophila)										
80212	CCDC92	coiled-coil domain containing 92										
80227	PAAF1	proteasomal ATPase-associated factor 1										

80279	CDK5RAP3	CDK5 regulatory subunit associated protein 3										
80305	TRABD	TraB domain containing										
80318	GKAP1	G kinase anchoring protein 1										
80700	UBXN6	UBX domain protein 6										
80742	PRR3	proline rich 3										
80746	TSEN2	tRNA splicing endonuclease 2 homolog (S. cerevisiae)										
80817	CEP44	centrosomal protein 44kDa										
80862	ZNRD1-AS1	ZNRD1 antisense RNA 1 (non-protein coding)										
8087	FXR1	fragile X mental retardation, autosomal homolog 1										
81037	CLPTM1L	CLPTM1-like										
81532	MOB2	MOB kinase activator 2										
81544	GDPD5	glycerophosphodiester phosphodiesterase domain containing 5										
81608	FIP1L1	FIP1 like 1 (S. cerevisiae)										
81619	TSPAN14	tetraspanin 14										
81788	NUAK2	NUAK family, SNF1-like kinase, 2										
81846	SBF2	SET binding factor 2										
81853	TMEM14B	transmembrane protein 14B										
81890	QTRT1	queuine tRNA-ribosyltransferase 1										
8233	ZRSR2	zinc finger (CCCH type), RNA-binding motif and serine/arginine rich 2										
83440	ADPGK	ADP-dependent glucokinase										
83451	ABHD11	abhydrolase domain containing 11										
83596	BCL2L12	BCL2-like 12 (proline rich)										
83694	RPS6KL1	ribosomal protein S6 kinase-like 1										
83707	TRPT1	tRNA phosphotransferase 1										
83746	L3MBTL2	l(3)mbt-like 2 (Drosophila)										
83862	TMEM120A	transmembrane protein 120A										
83940	TATDN1	TatD DNase domain containing 1										
83989	FAM172A	family with sequence similarity 172, member A										
84064	HDHD2	haloacid dehalogenase-like hydrolase domain containing 2										
84129	ACAD11	acyl-CoA dehydrogenase family, member 11										
84162	KIAA1109	KIAA1109										
84224	NBPF3	neuroblastoma breakpoint family, member 3										
84232	MAF1	MAF1 homolog (S. cerevisiae)										
84268	RPAIN	RPA interacting protein										
84288	EFCAB2	EF-hand calcium binding domain 2										
84292	WDR83	WD repeat domain 83										
84301	DDI2	DNA-damage inducible 1 homolog 2 (S. cerevisiae)										
84304	NUDT22	nudix (nucleoside diphosphate linked moiety X)-type motif 22										
84305	WIBG	within bgcn homolog (Drosophila)										
84314	TMEM107	transmembrane protein 107										
84326	C16orf13	chromosome 16 open reading frame 13										
84545	MRPL43	mitochondrial ribosomal protein L43										
84547	PGBD1	piggyBac transposable element derived 1										
84629	TNRC18	trinucleotide repeat containing 18										
84680	ACCS	1-aminocyclopropane-1-carboxylate synthase homolog (Arabidopsis)(non-functional)										
84722	PSRC1	proline/serine-rich coiled-coil 1										
84726	PRRC2B	proline-rich coiled-coil 2B										
8473	OGT	O-linked N-acetylglucosamine (GlcNAc) transferase (UDP-N-acetylglucosamine:polypeptide-N-acetylglucosaminyl transferase)										
8479	HIRIP3	HIRA interacting protein 3										
84866	TMEM25	transmembrane protein 25										
84901	NFATC2IP	nuclear factor of activated T-cells, cytoplasmic, calcineurin-dependent 2 interacting protein										
84922	FIZ1	FLT3-interacting zinc finger 1										
84941	HSH2D	hematopoietic SH2 domain containing										
84942	WDR73	WD repeat domain 73										
84958	SYTL1	synaptotagmin-like 1										
85014	TMEM141	transmembrane protein 141										
8502	PKP4	plakophilin 4										
85028	SNHG12	small nucleolar RNA host gene 12 (non-protein coding)										
8545	CGGBP1	CGG triplet repeat binding protein 1										
8558	CDK10	cyclin-dependent kinase 10										
8618	CADPS	Ca++-dependent secretion activator										
88455	ANKRD13A	ankyrin repeat domain 13A										
8847	DLEU2	deleted in lymphocytic leukemia 2 (non-protein coding)										
8859	STK19	serine/threonine kinase 19										
8904	CPNE1	copine I										
8906	AP1G2	adaptor-related protein complex 1, gamma 2 subunit										
89790	SIGLEC10	sialic acid binding Ig-like lectin 10										
89848	FCHSD1	FCH and double SH3 domains 1										
89857	KLHL6	kelch-like 6 (Drosophila)										
89894	TMEM116	transmembrane protein 116										
89970	RSPRY1	ring finger and SPRY domain containing 1										
9044	BTAF1	BTAF1 RNA polymerase II, B-TFIID transcription factor-associated, 170kDa (Mot1 homolog, S. cerevisiae)										
90592	ZNF700	zinc finger protein 700										
90668	LRRC16B	leucine rich repeat containing 16B										
90736	FAM104B	family with sequence similarity 104, member B										
90835	C16orf93	chromosome 16 open reading frame 93										
90933	TRIM41	tripartite motif containing 41										
9100	USP10	ubiquitin specific peptidase 10										
91289	LMF2	lipase maturation factor 2										
91319	DERL3	Der1-like domain family, member 3										
91445	RNF185	ring finger protein 185										
9147	NEMF	nuclear export mediator factor										
91544	UBXN11	UBX domain protein 11										
91801	ALKBH8	alkB, alkylation repair homolog 8 (E. coli)										
91833	WDR20	WD repeat domain 20										
92345	NAF1	nuclear assembly factor 1 homolog (S. cerevisiae)										
92558	CCDC64	coiled-coil domain containing 64										
9262	STK17B	serine/threonine kinase 17b										
93627	TBCK	TBC1 domain containing kinase										
9404	LPXN	leupaxin										
9526	MPDU1	mannose-P-dolichol utilization defect 1										
9546	APBA3	amyloid beta (A4) precursor protein-binding, family A, member 3										
9553	MRPL33	mitochondrial ribosomal protein L33										
9557	CHD1L	chromodomain helicase DNA binding protein 1-like										
9581	PREPL	prolyl endopeptidase-like										
9584	RBM39	RNA binding motif protein 39										
9648	GCC2	GRIP and coiled-coil domain containing 2										
9649	RALGPS1	Ral GEF with PH domain and SH3 binding motif 1										
9681	DEPDC5	DEP domain containing 5										
9701	PPP6R2	protein phosphatase 6, regulatory subunit 2										
9725	TMEM63A	transmembrane protein 63A										
9750	FAM65B	family with sequence similarity 65, member B										
9770	RASSF2	Ras association (RalGDS/AF-6) domain family member 2										
9772	KIAA0195	KIAA0195										
9779	TBC1D5	TBC1 domain family, member 5										
9786	KIAA0586	KIAA0586										
9814	SFI1	Sfi1 homolog, spindle assembly associated (yeast)										
9823	ARMCX2	armadillo repeat containing, X-linked 2										
9827	RGP1	RGP1 retrograde golgi transport homolog (S. cerevisiae)										
9842	PLEKHM1	pleckstrin homology domain containing, family M (with RUN domain) member 1										
9854	C2CD2L	C2CD2-like										
987	LRBA	LPS-responsive vesicle trafficking, beach and anchor containing										
9877	ZC3H11A	zinc finger CCCH-type containing 11A										
9894	TELO2	TEL2, telomere maintenance 2, homolog (S. cerevisiae)										
9898	UBAP2L	ubiquitin associated protein 2-like										
9905	SGSM2	small G protein signaling modulator 2										
9909	DENND4B	DENN/MADD domain containing 4B										
9967	THRAP3	thyroid hormone receptor associated protein 3										
9993	DGCR2	DiGeorge syndrome critical region gene 2										

Supplementary Table 8: MAVER-1 Alternative Splicing MsigDB Analysis

Overlap Results						
Collection(s):	CP, CP:BIOCARTA, CP:KEGG, CP:REACTOME, H					
# overlaps shown:	10					
# genesets in collections:	1379					
# genes in comparison (n):	1159					
# genes in universe (N):	45956					
Gene Set Name	# Genes in Gene Set (K)	Description	# Genes in Overlap (k)	k/K	p-value	FDR q-value
REACTOME_CELL_CYCLE	421	Genes involved in Cell Cycle	46	0.1093	6.46E-18	8.91E-15
REACTOME_IMMUNE_SYSTEM	933	Genes involved in Immune System	74	0.0793	1.12E-16	7.69E-14
HALLMARK_MITOTIC_SPINDLE	200	Genes important for mitotic spindle assembly.	31	0.155	7.37E-16	3.39E-13
REACTOME_CELL_CYCLE_MITOTIC	325	Genes involved in Cell Cycle, Mitotic	39	0.12	1.06E-15	3.65E-13
REACTOME_ADAPTIVE_IMMUNE_SYSTEM	539	Genes involved in Adaptive Immune System	46	0.0853	9.98E-13	2.75E-10
KEGG_UBIQUITIN_MEDIATED_PROTEOLYSIS	138	Ubiquitin mediated proteolysis	22	0.1594	6.41E-12	1.47E-09
REACTOME_MITOTIC_G2_G2_M_PHASES	81	Genes involved in Mitotic G2-G2/M phases	17	0.2099	1.70E-11	3.34E-09
REACTOME_RECRUITMENT_OF_MITOTIC_CENTROSOME_PROTEINS_AND_COMPLEXES	66	Genes involved in Recruitment of mitotic centrosome proteins and complexes	14	0.2121	8.90E-10	1.53E-07
HALLMARK_G2M_CHECKPOINT	200	Genes involved in the G2/M checkpoint, as in progression through the cell division cycle.	23	0.115	1.77E-09	2.71E-07
REACTOME_DNA_REPAIR	112	Genes involved in DNA Repair	17	0.1518	3.41E-09	4.70E-07

Gene/Gene Set Overlap Matrix

Entrez Gene Id	Gene Symbol	Gene Description	REACTOME_CELL_CYCLE	REACTOME_IMMUNE_SYSTEM	HALLMARK_MITOTIC_SPINDLE	REACTOME_CELL_CYCLE_MITOTIC	REACTOME_ADAPTIVE_IMMUNE_SYSTEM	KEGG_UBIQUITIN_MEDIATED_PROTEOLYSIS	REACTOME_MITOTIC_G2_G2_M_PHASES	REACTOME_RECRUITMENT_OF_MITOTIC_CENTROSOME_PROTEINS_AND_COMPLEXES	HALLMARK_G2M_CHECKPOINT	REACTOME_DNA_REPAIR
996	CDC27	cell division cycle 27 homolog (S. cerevisiae)	REACTOME_CELL_CYCLE	REACTOME_IMMUNE_SYSTEM	HALLMARK_MITOTIC_SPINDLE	REACTOME_CELL_CYCLE_MITOTIC	REACTOME_ADAPTIVE_IMMUNE_SYSTEM	KEGG_UBIQUITIN_MEDIATED_PROTEOLYSIS			HALLMARK_G2M_CHECKPOINT	
11065	UBE2C	ubiquitin-conjugating enzyme E2C	REACTOME_CELL_CYCLE	REACTOME_IMMUNE_SYSTEM		REACTOME_CELL_CYCLE_MITOTIC	REACTOME_ADAPTIVE_IMMUNE_SYSTEM	KEGG_UBIQUITIN_MEDIATED_PROTEOLYSIS			HALLMARK_G2M_CHECKPOINT	
10393	ANAPC10	anaphase promoting complex subunit 10	REACTOME_CELL_CYCLE	REACTOME_IMMUNE_SYSTEM		REACTOME_CELL_CYCLE_MITOTIC	REACTOME_ADAPTIVE_IMMUNE_SYSTEM	KEGG_UBIQUITIN_MEDIATED_PROTEOLYSIS			HALLMARK_G2M_CHECKPOINT	
1639	DCTN1	dynactin 1	REACTOME_CELL_CYCLE	REACTOME_IMMUNE_SYSTEM		REACTOME_CELL_CYCLE_MITOTIC	REACTOME_ADAPTIVE_IMMUNE_SYSTEM		REACTOME_MITOTIC_G2_G2_M_PHASES	REACTOME_RECRUITMENT_OF_MITOTIC_CENTROSOME_PROTEINS_AND_COMPLEXES		
5687	PSMA6	proteasome (prosome, macropain) subunit, alpha type, 6	REACTOME_CELL_CYCLE	REACTOME_IMMUNE_SYSTEM		REACTOME_CELL_CYCLE_MITOTIC	REACTOME_ADAPTIVE_IMMUNE_SYSTEM					
5686	PSMA5	proteasome (prosome, macropain) subunit, alpha type, 5	REACTOME_CELL_CYCLE	REACTOME_IMMUNE_SYSTEM		REACTOME_CELL_CYCLE_MITOTIC	REACTOME_ADAPTIVE_IMMUNE_SYSTEM					
5717	PSMD11	proteasome (prosome, macropain) 26S subunit, non-ATPase, 11	REACTOME_CELL_CYCLE	REACTOME_IMMUNE_SYSTEM		REACTOME_CELL_CYCLE_MITOTIC	REACTOME_ADAPTIVE_IMMUNE_SYSTEM					
143471	PSMA8	proteasome (prosome, macropain) subunit, alpha type, 8	REACTOME_CELL_CYCLE	REACTOME_IMMUNE_SYSTEM		REACTOME_CELL_CYCLE_MITOTIC	REACTOME_ADAPTIVE_IMMUNE_SYSTEM					
5527	PPP2R5C	protein phosphatase 2, regulatory subunit B', gamma	REACTOME_CELL_CYCLE	REACTOME_IMMUNE_SYSTEM		REACTOME_CELL_CYCLE_MITOTIC	REACTOME_ADAPTIVE_IMMUNE_SYSTEM					
29127	RACGAP1	Rac GTPase activating protein 1		REACTOME_IMMUNE_SYSTEM	HALLMARK_MITOTIC_SPINDLE		REACTOME_ADAPTIVE_IMMUNE_SYSTEM				HALLMARK_G2M_CHECKPOINT	
7318	UBA7	ubiquitin-like modifier activating enzyme 7		REACTOME_IMMUNE_SYSTEM			REACTOME_ADAPTIVE_IMMUNE_SYSTEM	KEGG_UBIQUITIN_MEDIATED_PROTEOLYSIS				
55294	FBXW7	F-box and WD repeat domain containing 7		REACTOME_IMMUNE_SYSTEM			REACTOME_ADAPTIVE_IMMUNE_SYSTEM	KEGG_UBIQUITIN_MEDIATED_PROTEOLYSIS				
6921	TCEB1	transcription elongation factor B (SIII), polypeptide 1 (15kDa, elongin C)		REACTOME_IMMUNE_SYSTEM			REACTOME_ADAPTIVE_IMMUNE_SYSTEM	KEGG_UBIQUITIN_MEDIATED_PROTEOLYSIS				
7428	VHL	von Hippel-Lindau tumor suppressor		REACTOME_IMMUNE_SYSTEM			REACTOME_ADAPTIVE_IMMUNE_SYSTEM	KEGG_UBIQUITIN_MEDIATED_PROTEOLYSIS				
7337	UBE3A	ubiquitin protein ligase E3A		REACTOME_IMMUNE_SYSTEM			REACTOME_ADAPTIVE_IMMUNE_SYSTEM	KEGG_UBIQUITIN_MEDIATED_PROTEOLYSIS				
10075	HUWE1	HECT, UBA and WWE domain containing 1		REACTOME_IMMUNE_SYSTEM			REACTOME_ADAPTIVE_IMMUNE_SYSTEM	KEGG_UBIQUITIN_MEDIATED_PROTEOLYSIS				
118424	UBE2J2	ubiquitin-conjugating enzyme E2, J2		REACTOME_IMMUNE_SYSTEM			REACTOME_ADAPTIVE_IMMUNE_SYSTEM	KEGG_UBIQUITIN_MEDIATED_PROTEOLYSIS				
26259	FBXW8	F-box and WD repeat domain containing 8		REACTOME_IMMUNE_SYSTEM			REACTOME_ADAPTIVE_IMMUNE_SYSTEM	KEGG_UBIQUITIN_MEDIATED_PROTEOLYSIS				
4591	TRIM37	tripartite motif containing 37		REACTOME_IMMUNE_SYSTEM			REACTOME_ADAPTIVE_IMMUNE_SYSTEM	KEGG_UBIQUITIN_MEDIATED_PROTEOLYSIS				
51619	UBE2D4	ubiquitin-conjugating enzyme E2D 4 (putative)		REACTOME_IMMUNE_SYSTEM			REACTOME_ADAPTIVE_IMMUNE_SYSTEM	KEGG_UBIQUITIN_MEDIATED_PROTEOLYSIS				
55284	UBE2W	ubiquitin-conjugating enzyme E2W (putative)		REACTOME_IMMUNE_SYSTEM			REACTOME_ADAPTIVE_IMMUNE_SYSTEM	KEGG_UBIQUITIN_MEDIATED_PROTEOLYSIS				
9354	UBE4A	ubiquitination factor E4A		REACTOME_IMMUNE_SYSTEM			REACTOME_ADAPTIVE_IMMUNE_SYSTEM	KEGG_UBIQUITIN_MEDIATED_PROTEOLYSIS				
27252	KLHL20	kelch-like 20 (Drosophila)		REACTOME_IMMUNE_SYSTEM			REACTOME_ADAPTIVE_IMMUNE_SYSTEM					
9520	NPEPPS	aminopeptidase puromycin sensitive		REACTOME_IMMUNE_SYSTEM			REACTOME_ADAPTIVE_IMMUNE_SYSTEM					
3106	HLA-B	major histocompatibility complex, class I, B		REACTOME_IMMUNE_SYSTEM			REACTOME_ADAPTIVE_IMMUNE_SYSTEM					
9632	SEC24C	SEC24 family, member C (S. cerevisiae)		REACTOME_IMMUNE_SYSTEM			REACTOME_ADAPTIVE_IMMUNE_SYSTEM					
972	CD74	CD74 molecule, major histocompatibility complex, class II invariant chain		REACTOME_IMMUNE_SYSTEM			REACTOME_ADAPTIVE_IMMUNE_SYSTEM					
5293	PIK3CD	phosphoinositide-3-kinase, catalytic, delta polypeptide		REACTOME_IMMUNE_SYSTEM			REACTOME_ADAPTIVE_IMMUNE_SYSTEM					
5894	RAF1	v-raf-1 murine leukemia viral oncogene homolog 1		REACTOME_IMMUNE_SYSTEM			REACTOME_ADAPTIVE_IMMUNE_SYSTEM					
3551	IKBKB	inhibitor of kappa light polypeptide gene enhancer in B-cells, kinase beta		REACTOME_IMMUNE_SYSTEM			REACTOME_ADAPTIVE_IMMUNE_SYSTEM					
8905	AP1S2	adaptor-related protein complex 1, sigma 2 subunit		REACTOME_IMMUNE_SYSTEM			REACTOME_ADAPTIVE_IMMUNE_SYSTEM					
1075	CTSC	cathepsin C		REACTOME_IMMUNE_SYSTEM			REACTOME_ADAPTIVE_IMMUNE_SYSTEM					
5335	PLCG1	phospholipase C, gamma 1		REACTOME_IMMUNE_SYSTEM			REACTOME_ADAPTIVE_IMMUNE_SYSTEM					
6885	MAP3K7	mitogen-activated protein kinase kinase kinase 7		REACTOME_IMMUNE_SYSTEM			REACTOME_ADAPTIVE_IMMUNE_SYSTEM					
163	AP2B1	adaptor-related protein complex 2, beta 1 subunit		REACTOME_IMMUNE_SYSTEM			REACTOME_ADAPTIVE_IMMUNE_SYSTEM					
3108	HLA-DMA	major histocompatibility complex, class II, DM alpha		REACTOME_IMMUNE_SYSTEM			REACTOME_ADAPTIVE_IMMUNE_SYSTEM					
5336	PLCG2	phospholipase C, gamma 2 (phosphatidylinositol-specific)		REACTOME_IMMUNE_SYSTEM			REACTOME_ADAPTIVE_IMMUNE_SYSTEM					
973	CD79A	CD79a molecule, immunoglobulin-associated alpha		REACTOME_IMMUNE_SYSTEM			REACTOME_ADAPTIVE_IMMUNE_SYSTEM					
930	CD19	CD19 molecule		REACTOME_IMMUNE_SYSTEM			REACTOME_ADAPTIVE_IMMUNE_SYSTEM					
974	CD79B	CD79b molecule, immunoglobulin-associated beta		REACTOME_IMMUNE_SYSTEM			REACTOME_ADAPTIVE_IMMUNE_SYSTEM					
3710	ITPR3	inositol 1,4,5-trisphosphate receptor, type 3		REACTOME_IMMUNE_SYSTEM			REACTOME_ADAPTIVE_IMMUNE_SYSTEM					
5058	PAK1	p21 protein (Cdc42/Rac)-activated kinase 1		REACTOME_IMMUNE_SYSTEM			REACTOME_ADAPTIVE_IMMUNE_SYSTEM					
51626	DYNC2LI1	dynein, cytoplasmic 2, light intermediate chain 1		REACTOME_IMMUNE_SYSTEM			REACTOME_ADAPTIVE_IMMUNE_SYSTEM					
10120	ACTR1B	ARP1 actin-related protein 1 homolog B, centractin beta (yeast)		REACTOME_IMMUNE_SYSTEM			REACTOME_ADAPTIVE_IMMUNE_SYSTEM					
8764	TNFRSF14	tumor necrosis factor receptor superfamily, member 14		REACTOME_IMMUNE_SYSTEM			REACTOME_ADAPTIVE_IMMUNE_SYSTEM					
11006	LILRB4	leukocyte immunoglobulin-like receptor, subfamily B (with TM and ITIM domains), member 4		REACTOME_IMMUNE_SYSTEM			REACTOME_ADAPTIVE_IMMUNE_SYSTEM					
10762	NUP50	nucleoporin 50kDa		REACTOME_IMMUNE_SYSTEM							HALLMARK_G2M_CHECKPOINT	
8086	AAAS	achalasia, adrenocortical insufficiency, alacrimia		REACTOME_IMMUNE_SYSTEM								
23225	NUP210	nucleoporin 210kDa		REACTOME_IMMUNE_SYSTEM								
23636	NUP62	nucleoporin 62kDa		REACTOME_IMMUNE_SYSTEM								
5094	PCBP2	poly(rC) binding protein 2		REACTOME_IMMUNE_SYSTEM								
7706	TRIM25	tripartite motif containing 25		REACTOME_IMMUNE_SYSTEM								
3665	IRF7	interferon regulatory factor 7		REACTOME_IMMUNE_SYSTEM								
834	CASP1	caspase 1, apoptosis-related cysteine peptidase (interleukin 1, beta, convertase)		REACTOME_IMMUNE_SYSTEM								
841	CASP8	caspase 8, apoptosis-related cysteine peptidase		REACTOME_IMMUNE_SYSTEM								
9470	EIF4E2	eukaryotic translation initiation factor 4E family member 2		REACTOME_IMMUNE_SYSTEM								
2889	RAPGEF1	Rap guanine nucleotide exchange factor (GEF) 1		REACTOME_IMMUNE_SYSTEM								
1981	EIF4G1	eukaryotic translation initiation factor 4 gamma, 1		REACTOME_IMMUNE_SYSTEM								
5771	PTPN2	protein tyrosine phosphatase, non-receptor type 2		REACTOME_IMMUNE_SYSTEM								
3663	IRF5	interferon regulatory factor 5		REACTOME_IMMUNE_SYSTEM								
4261	CIITA	class II, major histocompatibility complex, transactivator		REACTOME_IMMUNE_SYSTEM								
51447	IP6K2	inositol hexakisphosphate kinase 2		REACTOME_IMMUNE_SYSTEM								
7297	TYK2	tyrosine kinase 2		REACTOME_IMMUNE_SYSTEM								
1439	CSF2RB	colony stimulating factor 2 receptor, beta, low-affinity (granulocyte-macrophage)		REACTOME_IMMUNE_SYSTEM								
5600	MAPK11	mitogen-activated protein kinase 11		REACTOME_IMMUNE_SYSTEM								
10010	TANK	TRAF family member-associated NFKB activator		REACTOME_IMMUNE_SYSTEM								
4205	MEF2A	myocyte enhancer factor 2A		REACTOME_IMMUNE_SYSTEM								
177	AGER	advanced glycosylation end product-specific receptor		REACTOME_IMMUNE_SYSTEM								
55593	OTUD5	OTU domain containing 5		REACTOME_IMMUNE_SYSTEM								
10333	TLR6	toll-like receptor 6		REACTOME_IMMUNE_SYSTEM								
81793	TLR10	toll-like receptor 10		REACTOME_IMMUNE_SYSTEM								
10695	CNPY3	canopy 3 homolog (zebrafish)		REACTOME_IMMUNE_SYSTEM								
4064	CD180	CD180 molecule		REACTOME_IMMUNE_SYSTEM								
9447	AIM2	absent in melanoma 2		REACTOME_IMMUNE_SYSTEM								
4926	NUMA1	nuclear mitotic apparatus protein 1	REACTOME_CELL_CYCLE		HALLMARK_MITOTIC_SPINDLE	REACTOME_CELL_CYCLE_MITOTIC			REACTOME_MITOTIC_G2_G2_M_PHASES	REACTOME_RECRUITMENT_OF_MITOTIC_CENTROSOME_PROTEINS_AND_COMPLEXES	HALLMARK_G2M_CHECKPOINT	
11064	CNTRL	centriolin	REACTOME_CELL_CYCLE		HALLMARK_MITOTIC_SPINDLE	REACTOME_CELL_CYCLE_MITOTIC			REACTOME_MITOTIC_G2_G2_M_PHASES	REACTOME_RECRUITMENT_OF_MITOTIC_CENTROSOME_PROTEINS_AND_COMPLEXES		
5116	PCNT	pericentrin	REACTOME_CELL_CYCLE		HALLMARK_MITOTIC_SPINDLE	REACTOME_CELL_CYCLE_MITOTIC			REACTOME_MITOTIC_G2_G2_M_PHASES	REACTOME_RECRUITMENT_OF_MITOTIC_CENTROSOME_PROTEINS_AND_COMPLEXES		
11190	CEP250	centrosomal protein 250kDa	REACTOME_CELL_CYCLE		HALLMARK_MITOTIC_SPINDLE	REACTOME_CELL_CYCLE_MITOTIC			REACTOME_MITOTIC_G2_G2_M_PHASES	REACTOME_RECRUITMENT_OF_MITOTIC_CENTROSOME_PROTEINS_AND_COMPLEXES		
5108	PCM1	pericentriolar material 1	REACTOME_CELL_CYCLE		HALLMARK_MITOTIC_SPINDLE	REACTOME_CELL_CYCLE_MITOTIC			REACTOME_MITOTIC_G2_G2_M_PHASES	REACTOME_RECRUITMENT_OF_MITOTIC_CENTROSOME_PROTEINS_AND_COMPLEXES		
55125	CEP192	centrosomal protein 192kDa	REACTOME_CELL_CYCLE		HALLMARK_MITOTIC_SPINDLE	REACTOME_CELL_CYCLE_MITOTIC			REACTOME_MITOTIC_G2_G2_M_PHASES	REACTOME_RECRUITMENT_OF_MITOTIC_CENTROSOME_PROTEINS_AND_COMPLEXES		
55755	CDK5RAP2	CDK5 regulatory subunit associated protein 2	REACTOME_CELL_CYCLE		HALLMARK_MITOTIC_SPINDLE	REACTOME_CELL_CYCLE_MITOTIC			REACTOME_MITOTIC_G2_G2_M_PHASES	REACTOME_RECRUITMENT_OF_MITOTIC_CENTROSOME_PROTEINS_AND_COMPLEXES		
7840	ALMS1	Alstrom syndrome 1	REACTOME_CELL_CYCLE		HALLMARK_MITOTIC_SPINDLE	REACTOME_CELL_CYCLE_MITOTIC			REACTOME_MITOTIC_G2_G2_M_PHASES	REACTOME_RECRUITMENT_OF_MITOTIC_CENTROSOME_PROTEINS_AND_COMPLEXES		
85378	TUBGCP6	tubulin, gamma complex associated protein 6	REACTOME_CELL_CYCLE		HALLMARK_MITOTIC_SPINDLE	REACTOME_CELL_CYCLE_MITOTIC			REACTOME_MITOTIC_G2_G2_M_PHASES	REACTOME_RECRUITMENT_OF_MITOTIC_CENTROSOME_PROTEINS_AND_COMPLEXES		
3619	INCENP	inner centromere protein antigens 135/155kDa	REACTOME_CELL_CYCLE		HALLMARK_MITOTIC_SPINDLE	REACTOME_CELL_CYCLE_MITOTIC					HALLMARK_G2M_CHECKPOINT	
9735	KNTC1	kinetochore associated 1	REACTOME_CELL_CYCLE		HALLMARK_MITOTIC_SPINDLE	REACTOME_CELL_CYCLE_MITOTIC						
80254	CEP63	centrosomal protein 63kDa	REACTOME_CELL_CYCLE			REACTOME_CELL_CYCLE_MITOTIC			REACTOME_MITOTIC_G2_G2_M_PHASES	REACTOME_RECRUITMENT_OF_MITOTIC_CENTROSOME_PROTEINS_AND_COMPLEXES		
80321	CEP70	centrosomal protein 70kDa	REACTOME_CELL_CYCLE			REACTOME_CELL_CYCLE_MITOTIC			REACTOME_MITOTIC_G2_G2_M_PHASES	REACTOME_RECRUITMENT_OF_MITOTIC_CENTROSOME_PROTEINS_AND_COMPLEXES		
8481	OFD1	oral-facial-digital syndrome 1	REACTOME_CELL_CYCLE			REACTOME_CELL_CYCLE_MITOTIC			REACTOME_MITOTIC_G2_G2_M_PHASES	REACTOME_RECRUITMENT_OF_MITOTIC_CENTROSOME_PROTEINS_AND_COMPLEXES		
8636	SSNA1	Sjogren syndrome nuclear autoantigen 1	REACTOME_CELL_CYCLE			REACTOME_CELL_CYCLE_MITOTIC			REACTOME_MITOTIC_G2_G2_M_PHASES	REACTOME_RECRUITMENT_OF_MITOTIC_CENTROSOME_PROTEINS_AND_COMPLEXES		
4331	MNAT1	menage a trois homolog 1, cyclin H assembly factor (Xenopus laevis)	REACTOME_CELL_CYCLE			REACTOME_CELL_CYCLE_MITOTIC			REACTOME_MITOTIC_G2_G2_M_PHASES		HALLMARK_G2M_CHECKPOINT	REACTOME_DNA_REPAIR
7514	XPO1	exportin 1 (CRM1 homolog, yeast)	REACTOME_CELL_CYCLE			REACTOME_CELL_CYCLE_MITOTIC			REACTOME_MITOTIC_G2_G2_M_PHASES		HALLMARK_G2M_CHECKPOINT	
995	CDC25C	cell division cycle 25 homolog C (S. pombe)	REACTOME_CELL_CYCLE			REACTOME_CELL_CYCLE_MITOTIC			REACTOME_MITOTIC_G2_G2_M_PHASES			
9212	AURKB	aurora kinase B	REACTOME_CELL_CYCLE			REACTOME_CELL_CYCLE_MITOTIC					HALLMARK_G2M_CHECKPOINT	
4605	MYBL2	v-myb myeloblastosis viral oncogene homolog (avian)-like 2	REACTOME_CELL_CYCLE			REACTOME_CELL_CYCLE_MITOTIC					HALLMARK_G2M_CHECKPOINT	
8318	CDC45	cell division cycle 45 homolog (S. cerevisiae)	REACTOME_CELL_CYCLE			REACTOME_CELL_CYCLE_MITOTIC					HALLMARK_G2M_CHECKPOINT	
7027	TFDP1	transcription factor Dp-1	REACTOME_CELL_CYCLE			REACTOME_CELL_CYCLE_MITOTIC					HALLMARK_G2M_CHECKPOINT	
10714	POLD3	polymerase (DNA-directed), delta 3, accessory subunit	REACTOME_CELL_CYCLE			REACTOME_CELL_CYCLE_MITOTIC						REACTOME_DNA_REPAIR
5424	POLD1	polymerase (DNA directed), delta 1, catalytic subunit 125kDa	REACTOME_CELL_CYCLE			REACTOME_CELL_CYCLE_MITOTIC						REACTOME_DNA_REPAIR
5422	POLA1	polymerase (DNA directed), alpha 1, catalytic subunit	REACTOME_CELL_CYCLE			REACTOME_CELL_CYCLE_MITOTIC						
84515	MCM8	minichromosome maintenance complex component 8	REACTOME_CELL_CYCLE			REACTOME_CELL_CYCLE_MITOTIC						
23421	ITGB3BP	integrin beta 3 binding protein (beta3-endonexin)	REACTOME_CELL_CYCLE			REACTOME_CELL_CYCLE_MITOTIC						
80152	CENPT	centromere protein T	REACTOME_CELL_CYCLE			REACTOME_CELL_CYCLE_MITOTIC						
83540	NUF2	NUF2, NDC80 kinetochore complex component, homolog (S. cerevisiae)	REACTOME_CELL_CYCLE			REACTOME_CELL_CYCLE_MITOTIC						
91750	LIN52	lin-52 homolog (C. elegans)	REACTOME_CELL_CYCLE			REACTOME_CELL_CYCLE_MITOTIC						
64326	RFWD2	ring finger and WD repeat domain 2	REACTOME_CELL_CYCLE					KEGG_UBIQUITIN_MEDIATED_PROTEOLYSIS				
472	ATM	ataxia telangiectasia mutated	REACTOME_CELL_CYCLE									REACTOME_DNA_REPAIR
5884	RAD17	RAD17 homolog (S. pombe)	REACTOME_CELL_CYCLE									
84126	ATRIP	ATR interacting protein	REACTOME_CELL_CYCLE									
7015	TERT	telomerase reverse transcriptase	REACTOME_CELL_CYCLE									
26277	TINF2	TERF1 (TRF1)-interacting nuclear factor 2	REACTOME_CELL_CYCLE									
50511	SYCP3	synaptonemal complex protein 3	REACTOME_CELL_CYCLE									
1062	CENPE	centromere protein E, 312kDa			HALLMARK_MITOTIC_SPINDLE						HALLMARK_G2M_CHECKPOINT	
89941	RHOT2	ras homolog gene family, member T2			HALLMARK_MITOTIC_SPINDLE							
7204	TRIO	triple functional domain (PTPRF interacting)			HALLMARK_MITOTIC_SPINDLE							
393	ARHGAP4	Rho GTPase activating protein 4			HALLMARK_MITOTIC_SPINDLE							
64857	PLEKHG2	pleckstrin homology domain containing, family G (with RhoGef domain) member 2			HALLMARK_MITOTIC_SPINDLE							
6709	SPTAN1	spectrin, alpha, non-erythrocytic 1 (alpha-fodrin)			HALLMARK_MITOTIC_SPINDLE							
2316	FLNA	filamin A, alpha			HALLMARK_MITOTIC_SPINDLE							
85464	SSH2	slingshot homolog 2 (Drosophila)			HALLMARK_MITOTIC_SPINDLE							
10160	FARP1	FERM, RhoGEF (ARHGEF) and pleckstrin domain protein 1 (chondrocyte-derived)			HALLMARK_MITOTIC_SPINDLE							
51199	NIN	ninein (GSK3B interacting protein)			HALLMARK_MITOTIC_SPINDLE							
23192	ATG4B	ATG4 autophagy related 4 homolog B (S. cerevisiae)			HALLMARK_MITOTIC_SPINDLE							
10300	KATNB1	katanin p80 (WD repeat containing) subunit B 1			HALLMARK_MITOTIC_SPINDLE							
201176	ARHGAP27	Rho GTPase activating protein 27			HALLMARK_MITOTIC_SPINDLE							
51174	TUBD1	tubulin, delta 1			HALLMARK_MITOTIC_SPINDLE							
5311	PKD2	polycystic kidney disease 2 (autosomal dominant)			HALLMARK_MITOTIC_SPINDLE							
54443	ANLN	anillin, actin binding protein			HALLMARK_MITOTIC_SPINDLE							
84376	HOOK3	hook homolog 3 (Drosophila)			HALLMARK_MITOTIC_SPINDLE							
9771	RAPGEF5	Rap guanine nucleotide exchange factor (GEF) 5			HALLMARK_MITOTIC_SPINDLE							
83737	ITCH	itchy E3 ubiquitin protein ligase homolog (mouse)						KEGG_UBIQUITIN_MEDIATED_PROTEOLYSIS				
26091	HERC4	hect domain and RLD 4						KEGG_UBIQUITIN_MEDIATED_PROTEOLYSIS				
51366	UBR5	ubiquitin protein ligase E3 component n-recognin 5						KEGG_UBIQUITIN_MEDIATED_PROTEOLYSIS				
57448	BIRC6	baculoviral IAP repeat containing 6						KEGG_UBIQUITIN_MEDIATED_PROTEOLYSIS				
8925	HERC1	hect (homologous to the E6-AP (UBE3A) carboxyl terminus) domain and RCC1 (CHC1)-like domain (RLD) 1						KEGG_UBIQUITIN_MEDIATED_PROTEOLYSIS				
9616	RNF7	ring finger protein 7						KEGG_UBIQUITIN_MEDIATED_PROTEOLYSIS				
6434	TRA2B	transformer 2 beta homolog (Drosophila)									HALLMARK_G2M_CHECKPOINT	
7112	TMPO	thymopoietin									HALLMARK_G2M_CHECKPOINT	
10772	SRSF10	serine/arginine-rich splicing factor 10									HALLMARK_G2M_CHECKPOINT	
7090	TLE3	transducin-like enhancer of split 3 (E(sp1) homolog, Drosophila)									HALLMARK_G2M_CHECKPOINT	
81539	SLC38A1	solute carrier family 38, member 1									HALLMARK_G2M_CHECKPOINT	
1756	DMD	dystrophin									HALLMARK_G2M_CHECKPOINT	
10293	TRAIP	TRAF interacting protein									HALLMARK_G2M_CHECKPOINT	
10721	POLQ	polymerase (DNA directed), theta									HALLMARK_G2M_CHECKPOINT	
6760	SS18	synovial sarcoma translocation, chromosome 18									HALLMARK_G2M_CHECKPOINT	
8899	PRPF4B	PRP4 pre-mRNA processing factor 4 homolog B (yeast)									HALLMARK_G2M_CHECKPOINT	
5435	POLR2F	polymerase (RNA) II (DNA directed) polypeptide F										REACTOME_DNA_REPAIR
5431	POLR2B	polymerase (RNA) II (DNA directed) polypeptide B, 140kDa										REACTOME_DNA_REPAIR
2968	GTF2H4	general transcription factor IIH, polypeptide 4, 52kDa										REACTOME_DNA_REPAIR
4913	NTHL1	nth endonuclease III-like 1 (E. coli)										REACTOME_DNA_REPAIR
2547	XRCC6	X-ray repair complementing defective repair in Chinese hamster cells 6										REACTOME_DNA_REPAIR
4361	MRE11A	MRE11 meiotic recombination 11 homolog A (S. cerevisiae)										REACTOME_DNA_REPAIR
3981	LIG4	ligase IV, DNA, ATP-dependent										REACTOME_DNA_REPAIR
2189	FANCG	Fanconi anemia, complementation group G										REACTOME_DNA_REPAIR
2175	FANCA	Fanconi anemia, complementation group A										REACTOME_DNA_REPAIR
2187	FANCB	Fanconi anemia, complementation group B										REACTOME_DNA_REPAIR
2074	ERCC6	excision repair cross-complementing rodent repair deficiency, complementation group 6										REACTOME_DNA_REPAIR
5893	RAD52	RAD52 homolog (S. cerevisiae)										REACTOME_DNA_REPAIR
51455	REV1	REV1 homolog (S. cerevisiae)										REACTOME_DNA_REPAIR
3183	HNRNPC	heterogeneous nuclear ribonucleoprotein C (C1/C2)										
3181	HNRNPA2B1	heterogeneous nuclear ribonucleoprotein A2/B1										
23451	SF3B1	splicing factor 3b, subunit 1, 155kDa										
6625	SNRNP70	small nuclear ribonucleoprotein 70kDa (U1)										
10914	PAPOLA	poly(A) polymerase alpha										
10181	RBM5	RNA binding motif protein 5										
29894	CPSF1	cleavage and polyadenylation specific factor 1, 160kDa										
79869	CPSF7	cleavage and polyadenylation specific factor 7, 59kDa										
3187	HNRNPH1	heterogeneous nuclear ribonucleoprotein H1 (H)										
56339	METTL3	methyltransferase like 3										
104	ADARB1	adenosine deaminase, RNA-specific, B1										
10906	TRAFD1	TRAF-type zinc finger domain containing 1										
7453	WARS	tryptophanyl-tRNA synthetase										
5359	PLSCR1	phospholipid scramblase 1										
10561	IFI44	interferon-induced protein 44										
64761	PARP12	poly (ADP-ribose) polymerase family, member 12										
961	CD47	CD47 molecule										
135112	NCOA7	nuclear receptor coactivator 7										
85363	TRIM5	tripartite motif containing 5										
30833	NT5C	5', 3'-nucleotidase, cytosolic										
3704	ITPA	inosine triphosphatase (nucleoside triphosphate pyrophosphatase)										
4831	NME2	non-metastatic cells 2, protein (NM23B) expressed in										
171568	POLR3H	polymerase (RNA) III (DNA directed) polypeptide H (22.9kD)										
957	ENTPD5	ectonucleoside triphosphate diphosphohydrolase 5										
318	NUDT2	nudix (nucleoside diphosphate linked moiety X)-type motif 2										
955	ENTPD6	ectonucleoside triphosphate diphosphohydrolase 6 (putative)										
7296	TXNRD1	thioredoxin reductase 1										
1635	DCTD	dCMP deaminase										
2194	FASN	fatty acid synthase										
51763	INPP5K	inositol polyphosphate-5-phosphatase K										
32	ACACB	acetyl-CoA carboxylase beta										
369	ARAF	v-raf murine sarcoma 3611 viral oncogene homolog										
673	BRAF	v-raf murine sarcoma viral oncogene homolog B1										
5106	PCK2	phosphoenolpyruvate carboxykinase 2 (mitochondrial)										
2319	FLOT2	flotillin 2										
5256	PHKA2	phosphorylase kinase, alpha 2 (liver)										
5261	PHKG2	phosphorylase kinase, gamma 2 (testis)										
5257	PHKB	phosphorylase kinase, beta										
23265	EXOC7	exocyst complex component 7										
10312	TCIRG1	T-cell, immune regulator 1, ATPase, H+ transporting, lysosomal V0 subunit A3										
533	ATP6V0B	ATPase, H+ transporting, lysosomal 21kDa, V0 subunit b										
4891	SLC11A2	solute carrier family 11 (proton-coupled divalent metal ion transporters), member 2										
1497	CTNS	cystinosin, lysosomal cystine transporter										
3423	IDS	iduronate 2-sulfatase										
6448	SGSH	N-sulfoglucosamine sulfohydrolase										
4125	MAN2B1	mannosidase, alpha, class 2B, member 1										
4126	MANBA	mannosidase, beta A, lysosomal										
53	ACP2	acid phosphatase 2, lysosomal										
2588	GALNS	galactosamine (N-acetyl)-6-sulfate sulfatase										
1201	CLN3	ceroid-lipofuscinosis, neuronal 3										
256471	MFSD8	major facilitator superfamily domain containing 8										
26088	GGA1	golgi-associated, gamma adaptin ear containing, ARF binding protein 1										
3421	IDH3G	isocitrate dehydrogenase 3 (NAD+) gamma										
6188	RPS3	ribosomal protein S3										
6204	RPS10	ribosomal protein S10										
5372	PMM1	phosphomannomutase 1										
6203	RPS9	ribosomal protein S9										
6205	RPS11	ribosomal protein S11										
6134	RPL10	ribosomal protein L10										
6139	RPL17	ribosomal protein L17										
6142	RPL18A	ribosomal protein L18a										
6155	RPL27	ribosomal protein L27										
6171	RPL41	ribosomal protein L41										
1431	CS	citrate synthase										
10113	PREB	prolactin regulatory element binding										
8703	B4GALT3	UDP-Gal:betaGlcNAc beta 1,4- galactosyltransferase, polypeptide 3										
6185	RPN2	ribophorin II										
10195	ALG3	asparagine-linked glycosylation 3, alpha-1,3- mannosyltransferase homolog (S. cerevisiae)										
1798	DPAGT1	dolichyl-phosphate (UDP-N-acetylglucosamine) N-acetylglucosaminephosphotransferase 1 (GlcNAc-1-P transferase)										
79053	ALG8	asparagine-linked glycosylation 8, alpha-1,3-glucosyltransferase homolog (S. cerevisiae)										
79868	ALG13	asparagine-linked glycosylation 13 homolog (S. cerevisiae)										
6480	ST6GAL1	ST6 beta-galactosamide alpha-2,6-sialyltranferase 1										
3703	STT3A	STT3, subunit of the oligosaccharyltransferase complex, homolog A (S. cerevisiae)										
5045	FURIN	furin (paired basic amino acid cleaving enzyme)										
22803	XRN2	5'-3' exoribonuclease 2										
23556	PIGN	phosphatidylinositol glycan anchor biosynthesis, class N										
5281	PIGF	phosphatidylinositol glycan anchor biosynthesis, class F										
51809	GALNT7	UDP-N-acetyl-alpha-D-galactosamine:polypeptide N-acetylgalactosaminyltransferase 7 (GalNAc-T7)										
5204	PFDN5	prefoldin subunit 5										
8890	EIF2B4	eukaryotic translation initiation factor 2B, subunit 4 delta, 67kDa										
1936	EEF1D	eukaryotic translation elongation factor 1 delta (guanine nucleotide exchange protein)										
54461	FBXW5	F-box and WD repeat domain containing 5										
10245	TIMM17B	translocase of inner mitochondrial membrane 17 homolog B (yeast)										
1892	ECHS1	enoyl CoA hydratase, short chain, 1, mitochondrial										
1666	DECR1	2,4-dienoyl CoA reductase 1, mitochondrial										
37	ACADVL	acyl-CoA dehydrogenase, very long chain										
3155	HMGCL	3-hydroxymethyl-3-methylglutaryl-CoA lyase										
3295	HSD17B4	hydroxysteroid (17-beta) dehydrogenase 4										
10005	ACOT8	acyl-CoA thioesterase 8										
54884	RETSAT	retinol saturase (all-trans-retinol 13,14-reductase)										
1743	DLST	dihydrolipoamide S-succinyltransferase (E2 component of 2-oxo-glutarate complex)										
3420	IDH3B	isocitrate dehydrogenase 3 (NAD+) beta										
912	CD1D	CD1d molecule										
7390	UROS	uroporphyrinogen III synthase										
549	AUH	AU RNA binding protein/enoyl-CoA hydratase										
259307	IL4I1	interleukin 4 induced 1										
10961	ERP29	endoplasmic reticulum protein 29										
113	ADCY7	adenylate cyclase 7										
100	ADA	adenosine deaminase										
1716	DGUOK	deoxyguanosine kinase										
5151	PDE8A	phosphodiesterase 8A										
58497	PRUNE	prune homolog (Drosophila)										
4952	OCRL	oculocerebrorenal syndrome of Lowe										
79718	TBL1XR1	transducin (beta)-like 1 X-linked receptor 1										
200576	PIKFYVE	phosphoinositide kinase, FYVE finger containing										
23054	NCOA6	nuclear receptor coactivator 6										
23389	MED13L	mediator complex subunit 13-like										
219541	MED19	mediator complex subunit 19										
8398	PLA2G6	phospholipase A2, group VI (cytosolic, calcium-independent)										
81579	PLA2G12A	phospholipase A2, group XIIA										
26279	PLA2G2D	phospholipase A2, group IID										
4598	MVK	mevalonate kinase										
23760	PITPNB	phosphatidylinositol transfer protein, beta										
64419	MTMR14	myotubularin related protein 14										
1120	CHKB	choline kinase beta										
85465	EPT1	ethanolaminephosphotransferase 1 (CDP-ethanolamine-specific)										
56261	GPCPD1	glycerophosphocholine phosphodiesterase GDE1 homolog (S. cerevisiae)										
55331	ACER3	alkaline ceramidase 3										
57704	GBA2	glucosidase, beta (bile acid) 2										
5019	OXCT1	3-oxoacid CoA transferase 1										
4597	MVD	mevalonate (diphospho) decarboxylase										
4047	LSS	lanosterol synthase (2,3-oxidosqualene-lanosterol cyclase)										
6715	SRD5A1	steroid-5-alpha-reductase, alpha polypeptide 1 (3-oxo-5 alpha-steroid delta 4-dehydrogenase alpha 1)										
4673	NAP1L1	nucleosome assembly protein 1-like 1										
9238	TBRG4	transforming growth factor beta regulator 4										
197335	WDR90	WD repeat domain 90										
253714	MMS22L	MMS22-like, DNA repair protein										
29893	PSMC3IP	PSMC3 interacting protein										
6941	TCF19	transcription factor 19										
83461	CDCA3	cell division cycle associated 3										
9924	PAN2	PAN2 poly(A) specific ribonuclease subunit homolog (S. cerevisiae)										

5478	PPIA	peptidylprolyl isomerase A (cyclophilin A)										
11331	PHB2	prohibitin 2										
1665	DHX15	DEAH (Asp-Glu-Ala-His) box polypeptide 15										
10399	GNB2L1	guanine nucleotide binding protein (G protein), beta polypeptide 2-like 1										
8886	DDX18	DEAD (Asp-Glu-Ala-Asp) box polypeptide 18										
2079	ERH	enhancer of rudimentary homolog (Drosophila)										
26156	RSL1D1	ribosomal L1 domain containing 1										
6732	SRPK1	SRSF protein kinase 1										
2582	GALE	UDP-galactose-4-epimerase										
2592	GALT	galactose-1-phosphate uridylyltransferase										
2585	GALK2	galactokinase 2										
55577	NAGK	N-acetylglucosamine kinase										
197258	FUK	fucokinase										
132789	GNPDA2	glucosamine-6-phosphate deaminase 2										
5973	RENBP	renin binding protein										
80896	NPL	N-acetylneuraminate pyruvate lyase (dihydrodipicolinate synthase)										
4848	CNOT2	CCR4-NOT transcription complex, subunit 2										
57472	CNOT6	CCR4-NOT transcription complex, subunit 6										
23644	EDC4	enhancer of mRNA decapping 4										
1656	DDX6	DEAD (Asp-Glu-Ala-Asp) box polypeptide 6										
26019	UPF2	UPF2 regulator of nonsense transcripts homolog (yeast)										
3587	IL10RA	interleukin 10 receptor, alpha										
26053	AUTS2	autism susceptibility candidate 2										
8992	ATP6V0E1	ATPase, H+ transporting, lysosomal 9kDa, V0 subunit e1										
4729	NDUFV2	NADH dehydrogenase (ubiquinone) flavoprotein 2, 24kDa										
9551	ATP5J2	ATP synthase, H+ transporting, mitochondrial Fo complex, subunit F2										
1347	COX7A2	cytochrome c oxidase subunit VIIa polypeptide 2 (liver)										
9617	MTRF1	mitochondrial translational release factor 1										
22985	ACIN1	apoptotic chromatin condensation inducer 1										
10084	PQBP1	polyglutamine binding protein 1										
10450	PPIE	peptidylprolyl isomerase E (cyclophilin E)										
10713	USP39	ubiquitin specific peptidase 39										
51503	CWC15	CWC15 spliceosome-associated protein homolog (S. cerevisiae)										
55660	PRPF40A	PRP40 pre-mRNA processing factor 40 homolog A (S. cerevisiae)										
9879	DDX46	DEAD (Asp-Glu-Ala-Asp) box polypeptide 46										
9984	THOC1	THO complex 1										
23229	ARHGEF9	Cdc42 guanine nucleotide exchange factor (GEF) 9										
221472	FGD2	FYVE, RhoGEF and PH domain containing 2										
1124	CHN2	chimerin (chimaerin) 2										
399	RHOH	ras homolog gene family, member H										
55843	ARHGAP15	Rho GTPase activating protein 15										
83478	ARHGAP24	Rho GTPase activating protein 24										
84986	ARHGAP19	Rho GTPase activating protein 19										
2113	ETS1	v-ets erythroblastosis virus E26 oncogene homolog 1 (avian)										
112399	EGLN3	egl nine homolog 3 (C. elegans)										
9734	HDAC9	histone deacetylase 9										
51564	HDAC7	histone deacetylase 7										
55869	HDAC8	histone deacetylase 8										
2648	KAT2A	K(lysine) acetyltransferase 2A										
142678	MIB2	mindbomb homolog 2 (Drosophila)										
27327	TNRC6A	trinucleotide repeat containing 6A										
56983	POGLUT1	protein O-glucosyltransferase 1										
1487	CTBP1	C-terminal binding protein 1										
225	ABCD2	ATP-binding cassette, sub-family D (ALD), member 2										
373156	GSTK1	glutathione S-transferase kappa 1										
5828	PEX2	peroxisomal biogenesis factor 2										
2100	ESR2	estrogen receptor 2 (ER beta)										
3416	IDE	insulin-degrading enzyme										
51024	FIS1	fission 1 (mitochondrial outer membrane) homolog (S. cerevisiae)										
9270	ITGB1BP1	integrin beta 1 binding protein 1										
4638	MYLK	myosin light chain kinase										
5168	ENPP2	ectonucleotide pyrophosphatase/phosphodiesterase 2										
11194	ABCB8	ATP-binding cassette, sub-family B (MDR/TAP), member 8										
11176	BAZ2A	bromodomain adjacent to zinc finger domain, 2A										
128338	DRAM2	DNA-damage regulated autophagy modulator 2										
23344	ESYT1	extended synaptotagmin-like protein 1										
57017	COQ9	coenzyme Q9 homolog (S. cerevisiae)										
64225	ATL2	atlastin GTPase 2										
84173	ELMOD3	ELMO/CED-12 domain containing 3										
1499	CTNNB1	catenin (cadherin-associated protein), beta 1, 88kDa										
51176	LEF1	lymphoid enhancer-binding factor 1										
6303	SAT1	spermidine/spermine N1-acetyltransferase 1										
1676	DFFA	DNA fragmentation factor, 45kDa, alpha polypeptide										
8567	MADD	MAP-kinase activating death domain										
10059	DNM1L	dynamin 1-like										
6574	SLC20A1	solute carrier family 20 (phosphate transporter), member 1										
7818	DAP3	death associated protein 3										
10950	BTG3	BTG family, member 3										
5394	EXOSC10	exosome component 10										
23019	CNOT1	CCR4-NOT transcription complex, subunit 1										
84186	ZCCHC7	zinc finger, CCHC domain containing 7										
55215	FANCI	Fanconi anemia, complementation group I										
1857	DVL3	dishevelled, dsh homolog 3 (Drosophila)										
56998	CTNNBIP1	catenin, beta interacting protein 1										
818	CAMK2G	calcium/calmodulin-dependent protein kinase II gamma										
9618	TRAF4	TNF receptor-associated factor 4										
1606	DGKA	diacylglycerol kinase, alpha 80kDa										
23654	PLXNB2	plexin B2										
23303	KIF13B	kinesin family member 13B										
49856	WRAP73	WD repeat containing, antisense to TP73										
5820	PVT1	Pvt1 oncogene (non-protein coding)										
79651	RHBDF2	rhomboid 5 homolog 2 (Drosophila)										
3633	INPP5B	inositol polyphosphate-5-phosphatase, 75kDa										
51477	ISYNA1	inositol-3-phosphate synthase 1										
8550	MAPKAPK5	mitogen-activated protein kinase-activated protein kinase 5										
4294	MAP3K10	mitogen-activated protein kinase kinase kinase 10										
6929	TCF3	transcription factor 3 (E2A immunoglobulin enhancer binding factors E12/E47)										
6938	TCF12	transcription factor 12										
4637	MYL6	myosin, light chain 6, alkali, smooth muscle and non-muscle										
1020	CDK5	cyclin-dependent kinase 5										
2242	FES	feline sarcoma oncogene										
55558	PLXNA3	plexin A3										
64218	SEMA4A	sema domain, immunoglobulin domain (Ig), transmembrane domain (TM) and short cytoplasmic domain, (semaphorin) 4A										
1299	COL9A3	collagen, type IX, alpha 3										
3897	L1CAM	L1 cell adhesion molecule										
6478	SIAH2	seven in absentia homolog 2 (Drosophila)										
11124	FAF1	Fas (TNFRSF6) associated factor 1										
6789	STK4	serine/threonine kinase 4										
7170	TPM3	tropomyosin 3										
27148	STK36	serine/threonine kinase 36										
10319	LAMC3	laminin, gamma 3										
4775	NFATC3	nuclear factor of activated T-cells, cytoplasmic, calcineurin-dependent 3										
5091	PC	pyruvate carboxylase										
5191	PEX7	peroxisomal biogenesis factor 7										
11264	PXMP4	peroxisomal membrane protein 4, 24kDa										
4528	MTIF2	mitochondrial translational initiation factor 2										
440	ASNS	asparagine synthetase (glutamine-hydrolyzing)										
8974	P4HA2	prolyl 4-hydroxylase, alpha polypeptide II										
8301	PICALM	phosphatidylinositol binding clathrin assembly protein										
3145	HMBS	hydroxymethylbilane synthase										
26130	GAPVD1	GTPase activating protein and VPS9 domains 1										
26268	FBXO9	F-box protein 9										
51312	SLC25A37	solute carrier family 25, member 37										
6397	SEC14L1	SEC14-like 1 (S. cerevisiae)										
84809	CROCCP2	ciliary rootlet coiled-coil, rootletin pseudogene 2										
8932	MBD2	methyl-CpG binding domain protein 2										
7181	NR2C1	nuclear receptor subfamily 2, group C, member 1										
51547	SIRT7	sirtuin 7										
54815	GATAD2A	GATA zinc finger domain containing 2A										
80308	FLAD1	FAD1 flavin adenine dinucleotide synthetase homolog (S. cerevisiae)										
8034	SLC25A16	solute carrier family 25 (mitochondrial carrier; Graves disease autoantigen), member 16										
9963	SLC23A1	solute carrier family 23 (nucleobase transporters), member 1										
65220	NADK	NAD kinase										
2356	FPGS	folylpolyglutamate synthase										
79178	THTPA	thiamine triphosphatase										
9013	TAF1C	TATA box binding protein (TBP)-associated factor, RNA polymerase I, C, 110kDa										
10919	EHMT2	euchromatic histone-lysine N-methyltransferase 2										
2339	FNTA	farnesyltransferase, CAAX box, alpha										
9540	TP53I3	tumor protein p53 inducible protein 3										
1728	NQO1	NAD(P)H dehydrogenase, quinone 1										
6895	TARBP2	TAR (HIV-1) RNA binding protein 2										
468	ATF4	activating transcription factor 4 (tax-responsive enhancer element B67)										
9709	HERPUD1	homocysteine-inducible, endoplasmic reticulum stress-inducible, ubiquitin-like domain member 1										
3340	NDST1	N-deacetylase/N-sulfotransferase (heparan glucosaminyl) 1										
55454	CSGALNACT2	chondroitin sulfate N-acetylgalactosaminyltransferase 2										
4731	NDUFV3	NADH dehydrogenase (ubiquinone) flavoprotein 3, 10kDa										
10776	ARPP19	cAMP-regulated phosphoprotein, 19kDa										
64077	LHPP	phospholysine phosphohistidine inorganic pyrophosphate phosphatase										
3069	HDLBP	high density lipoprotein binding protein										
55139	ANKZF1	ankyrin repeat and zinc finger domain containing 1										
10327	AKR1A1	aldo-keto reductase family 1, member A1 (aldehyde reductase)										
6676	SPAG4	sperm associated antigen 4										
25788	RAD54B	RAD54 homolog B (S. cerevisiae)										
23162	MAPK8IP3	mitogen-activated protein kinase 8 interacting protein 3										
9112	MTA1	metastasis associated 1										
92140	MTDH	metadherin										
2782	GNB1	guanine nucleotide binding protein (G protein), beta polypeptide 1										
2060	EPS15	epidermal growth factor receptor pathway substrate 15										
1663	DDX11	DEAD/H (Asp-Glu-Ala-Asp/His) box polypeptide 11										
8720	MBTPS1	membrane-bound transcription factor peptidase, site 1										
63967	CLSPN	claspin										
2801	GOLGA2	golgin A2										
5051	PAFAH2	platelet-activating factor acetylhydrolase 2, 40kDa										
221955	DAGLB	diacylglycerol lipase, beta										
51131	PHF11	PHD finger protein 11										
22841	RAB11FIP2	RAB11 family interacting protein 2 (class I)										
3163	HMOX2	heme oxygenase (decycling) 2										
9056	SLC7A7	solute carrier family 7 (amino acid transporter light chain, y+L system), member 7										
8501	SLC43A1	solute carrier family 43, member 1										
9497	SLC4A7	solute carrier family 4, sodium bicarbonate cotransporter, member 7										
254428	SLC41A1	solute carrier family 41, member 1										
84912	SLC35B4	solute carrier family 35, member B4										
374868	ATP9B	ATPase, class II, type 9B										
29904	EEF2K	eukaryotic elongation factor-2 kinase										
5494	PPM1A	protein phosphatase, Mg2+/Mn2+ dependent, 1A										
9744	ACAP1	ArfGAP with coiled-coil, ankyrin repeat and PH domains 1										
56904	SH3GLB2	SH3-domain GRB2-like endophilin B2										
10015	PDCD6IP	programmed cell death 6 interacting protein										
64744	SMAP2	small ArfGAP2										
84364	ARFGAP2	ADP-ribosylation factor GTPase activating protein 2										
10652	YKT6	YKT6 v-SNARE homolog (S. cerevisiae)										
8675	STX16	syntaxin 16										
6810	STX4	syntaxin 4										
203062	TSNARE1	t-SNARE domain containing 1										
55014	STX17	syntaxin 17										
6867	TACC1	transforming, acidic coiled-coil containing protein 1										
8729	GBF1	golgi brefeldin A resistant guanine nucleotide exchange factor 1										
11316	COPE	coatomer protein complex, subunit epsilon										
984	CDK11B	cyclin-dependent kinase 11B										
728642	CDK11A	cyclin-dependent kinase 11A										
27131	SNX5	sorting nexin 5										
22900	CARD8	caspase recruitment domain family, member 8										
132949	AASDH	aminoadipate-semialdehyde dehydrogenase										
83852	SETDB2	SET domain, bifurcated 2										
6935	ZEB1	zinc finger E-box binding homeobox 1										
26472	PPP1R14B	protein phosphatase 1, regulatory (inhibitor) subunit 14B										
80895	ILKAP	integrin-linked kinase-associated serine/threonine phosphatase										
84458	LCOR	ligand dependent nuclear receptor corepressor										
10643	IGF2BP3	insulin-like growth factor 2 mRNA binding protein 3										
3692	EIF6	eukaryotic translation initiation factor 6										
441024	MTHFD2L	methylenetetrahydrofolate dehydrogenase (NADP+ dependent) 2-like										
81888	HYI	hydroxypyruvate isomerase (putative)										
23324	MAN2B2	mannosidase, alpha, class 2B, member 2										
29085	PHPT1	phosphohistidine phosphatase 1										
11284	PNKP	polynucleotide kinase 3'-phosphatase										
8100	IFT88	intraflagellar transport 88 homolog (Chlamydomonas)										
8405	SPOP	speckle-type POZ protein										
55066	PDPR	pyruvate dehydrogenase phosphatase regulatory subunit										
9943	OXSR1	oxidative-stress responsive 1										
5831	PYCR1	pyrroline-5-carboxylate reductase 1										
2744	GLS	glutaminase										
126382	NR2C2AP	nuclear receptor 2C2-associated protein										
10781	ZNF266	zinc finger protein 266										
121274	ZNF641	zinc finger protein 641										
147923	ZNF420	zinc finger protein 420										
7571	ZNF23	zinc finger protein 23 (KOX 16)										
7574	ZNF26	zinc finger protein 26										
7581	ZNF33A	zinc finger protein 33A										
7594	ZNF43	zinc finger protein 43										
7695	ZNF136	zinc finger protein 136										
80264	ZNF430	zinc finger protein 430										
90333	ZNF468	zinc finger protein 468										
7335	UBE2V1	ubiquitin-conjugating enzyme E2 variant 1										
83637	ZMIZ2	zinc finger, MIZ-type containing 2										
144455	E2F7	E2F transcription factor 7										
112483	SAT2	spermidine/spermine N1-acetyltransferase family member 2										
7059	THBS3	thrombospondin 3										
10237	SLC35B1	solute carrier family 35, member B1										
115817	DHRS1	dehydrogenase/reductase (SDR family) member 1										
9988	DMTF1	cyclin D binding myb-like transcription factor 1										
7392	USF2	upstream transcription factor 2, c-fos interacting										
7391	USF1	upstream transcription factor 1										
64333	ARHGAP9	Rho GTPase activating protein 9										
5025	P2RX4	purinergic receptor P2X, ligand-gated ion channel, 4										
57038	RARS2	arginyl-tRNA synthetase 2, mitochondrial										
80222	TARS2	threonyl-tRNA synthetase 2, mitochondrial (putative)										
123283	TARSL2	threonyl-tRNA synthetase-like 2										
6801	STRN	striatin, calmodulin binding protein										
10043	TOM1	target of myb1 (chicken)										
22797	TFEC	transcription factor EC										
51087	YBX2	Y box binding protein 2										
7247	TSN	translin										
8028	MLLT10	myeloid/lymphoid or mixed-lineage leukemia (trithorax homolog, Drosophila); translocated to, 10										
1730	DIAPH2	diaphanous homolog 2 (Drosophila)										
3071	NCKAP1L	NCK-associated protein 1-like										
54961	SSH3	slingshot homolog 3 (Drosophila)										
57060	PCBP4	poly(rC) binding protein 4										
7159	TP53BP2	tumor protein p53 binding protein, 2										
8973	CHRNA6	cholinergic receptor, nicotinic, alpha 6										
3783	KCNN4	potassium intermediate/small conductance calcium-activated channel, subfamily N, member 4										
3782	KCNN3	potassium intermediate/small conductance calcium-activated channel, subfamily N, member 3										
9046	DOK2	docking protein 2, 56kDa										
6640	SNTA1	syntrophin, alpha 1 (dystrophin-associated protein A1, 59kDa, acidic component)										
1390	CREM	cAMP responsive element modulator										
7511	XPNPEP1	X-prolyl aminopeptidase (aminopeptidase P) 1, soluble										
4839	NOP2	NOP2 nucleolar protein homolog (yeast)										
22807	IKZF2	IKAROS family zinc finger 2 (Helios)										
2314	FLII	flightless I homolog (Drosophila)										
79629	OCEL1	occludin/ELL domain containing 1										
59339	PLEKHA2	pleckstrin homology domain containing, family A (phosphoinositide binding specific) member 2										
7804	LRP8	low density lipoprotein receptor-related protein 8, apolipoprotein e receptor										
23523	CABIN1	calcineurin binding protein 1										
25989	ULK3	unc-51-like kinase 3 (C. elegans)										
9519	TBPL1	TBP-like 1										
11315	PARK7	parkinson protein 7										
7345	UCHL1	ubiquitin carboxyl-terminal esterase L1 (ubiquitin thiolesterase)										
2686	GGT7	gamma-glutamyltransferase 7										
50624	CUZD1	CUB and zona pellucida-like domains 1										
26037	SIPA1L1	signal-induced proliferation-associated 1 like 1										
27109	ATP5S	ATP synthase, H+ transporting, mitochondrial Fo complex, subunit s (factor B)										
51460	SFMBT1	Scm-like with four mbt domains 1										
27128	CYTH4	cytohesin 4										
2305	FOXM1	forkhead box M1										
79017	GGCT	gamma-glutamylcyclotransferase										
4008	LMO7	LIM domain 7										
8189	SYMPK	symplekin										
5310	PKD1	polycystic kidney disease 1 (autosomal dominant)										
7073	TIAL1	TIA1 cytotoxic granule-associated RNA binding protein-like 1										
2289	FKBP5	FK506 binding protein 5										
23596	OPN3	opsin 3										
55793	FAM63A	family with sequence similarity 63, member A										
9094	UNC119	unc-119 homolog (C. elegans)										
29887	SNX10	sorting nexin 10										
5936	RBM4	RNA binding motif protein 4										
114049	WBSCR22	Williams Beuren syndrome chromosome region 22										
1787	TRDMT1	tRNA aspartic acid methyltransferase 1										
55768	NGLY1	N-glycanase 1										
27334	P2RY10	purinergic receptor P2Y, G-protein coupled, 10										
89932	PAPLN	papilin, proteoglycan-like sulfated glycoprotein										
286205	SCAI	suppressor of cancer cell invasion										
5586	PKN2	protein kinase N2										
970	CD70	CD70 molecule										
93643	TJAP1	tight junction associated protein 1 (peripheral)										
3965	LGALS9	lectin, galactoside-binding, soluble, 9										
10863	ADAM28	ADAM metallopeptidase domain 28										
8425	LTBP4	latent transforming growth factor beta binding protein 4										
6738	TROVE2	TROVE domain family, member 2										
10777	ARPP21	cAMP-regulated phosphoprotein, 21kDa										
84233	TMEM126A	transmembrane protein 126A										
196294	IMMP1L	IMP1 inner mitochondrial membrane peptidase-like (S. cerevisiae)										
5875	RABGGTA	Rab geranylgeranyltransferase, alpha subunit										
55733	HHAT	hedgehog acyltransferase										
8875	VNN2	vanin 2										
100131187	TSTD1	thiosulfate sulfurtransferase (rhodanese)-like domain containing 1										
100131998	RRN3P3	RNA polymerase I transcription factor homolog (S. cerevisiae) pseudogene 3										
10101	NUBP2	nucleotide binding protein 2										
10184	LHFPL2	lipoma HMGIC fusion partner-like 2										
10198	MPHOSPH9	M-phase phosphoprotein 9										
10227	MFSD10	major facilitator superfamily domain containing 10										
10230	NBR2	neighbor of BRCA1 gene 2 (non-protein coding)										
10260	DENND4A	DENN/MADD domain containing 4A										
10296	MAEA	macrophage erythroblast attacher										
10307	APBB3	amyloid beta (A4) precursor protein-binding, family B, member 3										
10346	TRIM22	tripartite motif containing 22										
10384	BTN3A3	butyrophilin, subfamily 3, member A3										
10385	BTN2A2	butyrophilin, subfamily 2, member A2										

10428	CFDP1	craniofacial development protein 1
10443	N4BP2L2	NEDD4 binding protein 2-like 2
10495	ENOX2	ecto-NOX disulfide-thiol exchanger 2
10518	CIB2	calcium and integrin binding family member 2
10641	NPRL2	nitrogen permease regulator-like 2 (S. cerevisiae)
10658	CELF1	CUGBP, Elav-like family member 1
10807	SDCCAG3	serologically defined colon cancer antigen 3
10847	SRCAP	Snf2-related CREBBP activator protein
10898	CPSF4	cleavage and polyadenylation specific factor 4, 30kDa
10922	FASTK	Fas-activated serine/threonine kinase
10927	SPIN1	spindlin 1
10944	C11orf58	chromosome 11 open reading frame 58
10973	ASCC3	activating signal cointegrator 1 complex subunit 3
10985	GCN1L1	GCN1 general control of amino-acid synthesis 1-like 1 (yeast)
1102	RCBTB2	regulator of chromosome condensation (RCC1) and BTB (POZ) domain containing protein 2
11020	IFT27	intraflagellar transport 27 homolog (Chlamydomonas)
11132	CAPN10	calpain 10
11201	POLI	polymerase (DNA directed) iota
1121	CHM	choroideremia (Rab escort protein 1)
11212	PROSC	proline synthetase co-transcribed homolog (bacterial)
11234	HPS5	Hermansky-Pudlak syndrome 5
112479	ERI2	ERI1 exoribonuclease family member 2
11262	SP140	SP140 nuclear body protein
11270	NRM	nurim (nuclear envelope membrane protein)
11272	PRR4	proline rich 4 (lacrimal)
112840	WDR89	WD repeat domain 89
11322	TMC6	transmembrane channel-like 6
114659	LRRC37B	leucine rich repeat containing 37B
114803	MYSM1	Myb-like, SWIRM and MPN domains 1
114822	RHPN1	rhophilin, Rho GTPase binding protein 1
114879	OSBPL5	oxysterol binding protein-like 5
115209	OMA1	OMA1 homolog, zinc metallopeptidase (S. cerevisiae)
115294	PCMTD1	protein-L-isoaspartate (D-aspartate) O-methyltransferase domain containing 1
1153	CIRBP	cold inducible RNA binding protein
115811	IQCD	IQ motif containing D
116966	WDR17	WD repeat domain 17
116988	AGAP3	ArfGAP with GTPase domain, ankyrin repeat and PH domain 3
118433	RPL23AP7	ribosomal protein L23a pseudogene 7
1196	CLK2	CDC-like kinase 2
122509	IFI27L1	interferon, alpha-inducible protein 27-like 1
123096	SLC25A29	solute carrier family 25, member 29
123606	NIPA1	non imprinted in Prader-Willi/Angelman syndrome 1
124540	MSI2	musashi homolog 2 (Drosophila)
124790	HEXIM2	hexamethylene bis-acetamide inducible 2
124944	C17orf49	chromosome 17 open reading frame 49
126133	ALDH16A1	aldehyde dehydrogenase 16 family, member A1
126526	C19orf47	chromosome 19 open reading frame 47
127002	ATXN7L2	ataxin 7-like 2
128308	MRPL55	mitochondrial ribosomal protein L55
128387	TATDN3	TatD DNase domain containing 3
128977	C22orf39	chromosome 22 open reading frame 39
130026	ICA1L	islet cell autoantigen 1,69kDa-like
131408	FAM131A	family with sequence similarity 131, member A
131601	TPRA1	transmembrane protein, adipocyte asscociated 1
132320	SCLT1	sodium channel and clathrin linker 1
137695	TMEM68	transmembrane protein 68
138241	C9orf85	chromosome 9 open reading frame 85
140564	APOBEC3D	apolipoprotein B mRNA editing enzyme, catalytic polypeptide-like 3D
140691	TRIM69	tripartite motif containing 69
140890	SREK1	splicing regulatory glutamine/lysine-rich protein 1
143570	XRRA1	X-ray radiation resistance associated 1
145508	CEP128	centrosomal protein 128kDa
146059	CDAN1	congenital dyserythropoietic anemia, type I
146206	RLTPR	RGD motif, leucine rich repeats, tropomodulin domain and proline-rich containing
148206	ZNF714	zinc finger protein 714
149628	PYHIN1	pyrin and HIN domain family, member 1
150365	MEI1	meiosis inhibitor 1
150709	ANKAR	ankyrin and armadillo repeat containing
150962	PUS10	pseudouridylate synthase 10
151556	GPR155	G protein-coupled receptor 155
152815	THAP6	THAP domain containing 6
153129	SLC38A9	solute carrier family 38, member 9
157680	VPS13B	vacuolar protein sorting 13 homolog B (yeast)
159091	FAM122C	family with sequence similarity 122C
159195	USP54	ubiquitin specific peptidase 54
163486	DENND1B	DENN/MADD domain containing 1B
165055	CCDC138	coiled-coil domain containing 138
166378	SPATA5	spermatogenesis associated 5
167153	PAPD4	PAP associated domain containing 4
171017	ZNF384	zinc finger protein 384
1939	EIF2D	eukaryotic translation initiation factor 2D
196441	ZFC3H1	zinc finger, C3H1-type containing
197135	PATL2	protein associated with topoisomerase II homolog 2 (yeast)
199786	FAM129C	family with sequence similarity 129, member C
200081	TXLNA	taxilin alpha
200845	KCTD6	potassium channel tetramerisation domain containing 6
201254	STRA13	stimulated by retinoic acid 13 homolog (mouse)
201255	LRRC45	leucine rich repeat containing 45
201283	AMZ2P1	archaelysin family metallopeptidase 2 pseudogene 1
203054	ADCK5	aarF domain containing kinase 5
203328	SUSD3	sushi domain containing 3
205564	SENP5	SUMO1/sentrin specific peptidase 5
219402	MTIF3	mitochondrial translational initiation factor 3
219736	STOX1	storkhead box 1
219931	TPCN2	two pore segment channel 2
221002	RASGEF1A	RasGEF domain family, member 1A
221302	ZUFSP	zinc finger with UFM1-specific peptidase domain
2217	FCGRT	Fc fragment of IgG, receptor, transporter, alpha
22877	MLXIP	MLX interacting protein
22878	TRAPPC8	trafficking protein particle complex 8
22902	RUFY3	RUN and FYVE domain containing 3
23023	TMCC1	transmembrane and coiled-coil domain family 1
23042	PDXDC1	pyridoxal-dependent decarboxylase domain containing 1
23051	ZHX3	zinc fingers and homeoboxes 3
23064	SETX	senataxin
23077	MYCBP2	MYC binding protein 2
23113	CUL9	cullin 9
23130	ATG2A	ATG2 autophagy related 2 homolog A (S. cerevisiae)
23141	ANKLE2	ankyrin repeat and LEM domain containing 2
23149	FCHO1	FCH domain only 1
23167	EFR3A	EFR3 homolog A (S. cerevisiae)
23243	ANKRD28	ankyrin repeat domain 28
23244	PDS5A	PDS5, regulator of cohesion maintenance, homolog A (S. cerevisiae)
23247	KIAA0556	KIAA0556
23262	PPIP5K2	diphosphoinositol pentakisphosphate kinase 2
23301	EHBP1	EH domain binding protein 1
23334	SZT2	seizure threshold 2 homolog (mouse)
23353	SUN1	Sad1 and UNC84 domain containing 1
23354	HAUS5	HAUS augmin-like complex, subunit 5
23367	LARP1	La ribonucleoprotein domain family, member 1
23369	PUM2	pumilio homolog 2 (Drosophila)
23384	SPECC1L	sperm antigen with calponin homology and coiled-coil domains 1-like
23394	ADNP	activity-dependent neuroprotector homeobox
23400	ATP13A2	ATPase type 13A2
23473	CAPN7	calpain 7
23503	ZFYVE26	zinc finger, FYVE domain containing 26
23512	SUZ12	suppressor of zeste 12 homolog (Drosophila)
23513	SCRIB	scribbled homolog (Drosophila)
23549	DNPEP	aspartyl aminopeptidase
23559	WBP1	WW domain binding protein 1
23567	ZNF346	zinc finger protein 346
23613	ZMYND8	zinc finger, MYND-type containing 8
23642	SNHG1	small nucleolar RNA host gene 1 (non-protein coding)
23648	SSBP3	single stranded DNA binding protein 3
23787	MTCH1	mitochondrial carrier 1
24139	EML2	echinoderm microtubule associated protein like 2
254102	EHBP1L1	EH domain binding protein 1-like 1
254295	PHYHD1	phytanoyl-CoA dioxygenase domain containing 1
254887	ZDHHC23	zinc finger, DHHC-type containing 23
255104	TMCO4	transmembrane and coiled-coil domains 4
256380	SCML4	sex comb on midleg-like 4 (Drosophila)
25771	TBC1D22A	TBC1 domain family, member 22A
25792	CIZ1	CDKN1A interacting zinc finger protein 1
25831	HECTD1	HECT domain containing 1
25832	NBPF14	neuroblastoma breakpoint family, member 14
25837	RAB26	RAB26, member RAS oncogene family
25875	LETMD1	LETM1 domain containing 1
259266	ASPM	asp (abnormal spindle) homolog, microcephaly associated (Drosophila)
25956	SEC31B	SEC31 homolog B (S. cerevisiae)
25963	TMEM87A	transmembrane protein 87A
26013	L3MBTL1	l(3)mbt-like 1 (Drosophila)
26015	RPAP1	RNA polymerase II associated protein 1
26040	SETBP1	SET binding protein 1
26054	SENP6	SUMO1/sentrin specific peptidase 6
26058	GIGYF2	GRB10 interacting GYF protein 2
26065	LSM14A	LSM14A, SCD6 homolog A (S. cerevisiae)
26151	NAT9	N-acetyltransferase 9 (GCN5-related, putative)
26262	TSPAN17	tetraspanin 17
26297	SERGEF	secretion regulating guanine nucleotide exchange factor
2630	GBAP1	glucosidase, beta, acid pseudogene 1
26528	DAZAP1	DAZ associated protein 1
26750	RPS6KC1	ribosomal protein S6 kinase, 52kDa, polypeptide 1
26751	SH3YL1	SH3 domain containing, Ysc84-like 1 (S. cerevisiae)
26996	GPR160	G protein-coupled receptor 160
27004	TCL6	T-cell leukemia/lymphoma 6 (non-protein coding)
27020	NPTN	neuroplastin
27158	NDOR1	NADPH dependent diflavin oxidoreductase 1
27163	NAAA	N-acylethanolamine acid amidase
27315	PGAP2	post-GPI attachment to proteins 2
27332	ZNF638	zinc finger protein 638
27352	SGSM3	small G protein signaling modulator 3
27433	TOR2A	torsin family 2, member A
2804	GOLGB1	golgin B1
282809	POC1B	POC1 centriolar protein homolog B (Chlamydomonas)
283237	TTC9C	tetratricopeptide repeat domain 9C
283578	TMED8	transmembrane emp24 protein transport domain containing 8
283755	HERC2P3	hect domain and RLD 2 pseudogene 3
283989	TSEN54	tRNA splicing endonuclease 54 homolog (S. cerevisiae)
284001	CCDC57	coiled-coil domain containing 57
284004	HEXDC	hexosaminidase (glycosyl hydrolase family 20, catalytic domain) containing
284403	WDR62	WD repeat domain 62
284565	NBPF15	neuroblastoma breakpoint family, member 15
284695	ZNF326	zinc finger protein 326
284900	TTC28-AS1	TTC28 antisense RNA 1 (non-protein coding)
284992	CCDC150	coiled-coil domain containing 150
285025	CCDC141	coiled-coil domain containing 141
286053	NSMCE2	non-SMC element 2, MMS21 homolog (S. cerevisiae)
286257	C9orf142	chromosome 9 open reading frame 142
28638	TRBC2	T cell receptor beta constant 2
28957	MRPS28	mitochondrial ribosomal protein S28
28969	BZW2	basic leucine zipper and W2 domains 2
29066	ZC3H7A	zinc finger CCCH-type containing 7A
29083	GTPBP8	GTP-binding protein 8 (putative)
29087	THYN1	thymocyte nuclear protein 1
29109	FHOD1	formin homology 2 domain containing 1
29115	SAP30BP	SAP30 binding protein
29116	MYLIP	myosin regulatory light chain interacting protein
2975	GTF3C1	general transcription factor IIIC, polypeptide 1, alpha 220kDa
29899	GPSM2	G-protein signaling modulator 2
29952	DPP7	dipeptidyl-peptidase 7
29954	POMT2	protein-O-mannosyltransferase 2
3257	HPS1	Hermansky-Pudlak syndrome 1
3338	DNAJC4	DnaJ (Hsp40) homolog, subfamily C, member 4
339123	JMJD8	jumonji domain containing 8
340252	ZNF680	zinc finger protein 680
340351	AGBL3	ATP/GTP binding protein-like 3
347862	PDDC1	Parkinson disease 7 domain containing 1
349075	ZNF713	zinc finger protein 713
349152	DPY19L2P2	dpy-19-like 2 pseudogene 2 (C. elegans)
353322	ANKRD37	ankyrin repeat domain 37
374900	ZNF568	zinc finger protein 568
375248	ANKRD36	ankyrin repeat domain 36
386593	CHKB-CPT1B	CHKB-CPT1B readthrough (non-protein coding)
387103	CENPW	centromere protein W
388561	ZNF761	zinc finger protein 761
3895	KTN1	kinectin 1 (kinesin receptor)
3903	LAIR1	leukocyte-associated immunoglobulin-like receptor 1
3995	FADS3	fatty acid desaturase 3
400410	ST20	suppressor of tumorigenicity 20
400954	EML6	echinoderm microtubule associated protein like 6
4034	LRCH4	leucine-rich repeats and calponin homology (CH) domain containing 4
404636	FAM45A	family with sequence similarity 45, member A
4063	LY9	lymphocyte antigen 9
4154	MBNL1	muscleblind-like (Drosophila)
4204	MECP2	methyl CpG binding protein 2 (Rett syndrome)
4302	MLLT6	myeloid/lymphoid or mixed-lineage leukemia (trithorax homolog, Drosophila); translocated to, 6
440400	RNASEK	ribonuclease, RNase K
440590	ZYG11A	zyg-11 homolog A (C. elegans)
440675	NBPF12	neuroblastoma breakpoint family, member 12
4664	NAB1	NGFI-A binding protein 1 (EGR1 binding protein 1)
4752	NEK3	NIMA (never in mitosis gene a)-related kinase 3
4817	NIT1	nitrilase 1
494143	CHAC2	ChaC, cation transport regulator homolog 2 (E. coli)
4948	OCA2	oculocutaneous albinism II
49855	SCAPER	S-phase cyclin A-associated protein in the ER
50854	C6orf48	chromosome 6 open reading frame 48
51136	RNFT1	ring finger protein, transmembrane 1
51237	MZB1	marginal zone B and B1 cell-specific protein
51249	TMEM69	transmembrane protein 69
51256	TBC1D7	TBC1 domain family, member 7
5127	CDK16	cyclin-dependent kinase 16
51281	ANKMY1	ankyrin repeat and MYND domain containing 1
51317	PHF21A	PHD finger protein 21A
51338	MS4A4A	membrane-spanning 4-domains, subfamily A, member 4
51426	POLK	polymerase (DNA directed) kappa
51542	VPS54	vacuolar protein sorting 54 homolog (S. cerevisiae)
51571	FAM49B	family with sequence similarity 49, member B
51574	LARP7	La ribonucleoprotein domain family, member 7
51605	TRMT6	tRNA methyltransferase 6 homolog (S. cerevisiae)
51654	CDK5RAP1	CDK5 regulatory subunit associated protein 1
51693	TRAPPC2L	trafficking protein particle complex 2-like
51699	VPS29	vacuolar protein sorting 29 homolog (S. cerevisiae)
51816	CECR1	cat eye syndrome chromosome region, candidate 1
53346	TM6SF1	transmembrane 6 superfamily member 1
5378	PMS1	PMS1 postmeiotic segregation increased 1 (S. cerevisiae)
5379	PMS2P1	postmeiotic segregation increased 2 pseudogene 1
53827	FXYD5	FXYD domain containing ion transport regulator 5
5387	PMS2P3	postmeiotic segregation increased 2 pseudogene 3
53917	RAB24	RAB24, member RAS oncogene family
53938	PPIL3	peptidylprolyl isomerase (cyclophilin)-like 3
54058	C21orf58	chromosome 21 open reading frame 58
54148	MRPL39	mitochondrial ribosomal protein L39
5428	POLG	polymerase (DNA directed), gamma
54332	GDAP1	ganglioside-induced differentiation-associated protein 1
54476	RNF216	ring finger protein 216
54517	PUS7	pseudouridylate synthase 7 homolog (S. cerevisiae)
54621	VSIG10	V-set and immunoglobulin domain containing 10
54676	GTPBP2	GTP binding protein 2
54799	MBTD1	mbt domain containing 1
54808	DYM	dymeclin
54828	BCAS3	breast carcinoma amplified sequence 3
54836	BSPRY	B-box and SPRY domain containing
54850	FBXL12	F-box and leucine-rich repeat protein 12
54851	ANKRD49	ankyrin repeat domain 49
54853	WDR55	WD repeat domain 55
54874	FNBP1L	formin binding protein 1-like
54878	DPP8	dipeptidyl-peptidase 8
54879	ST7L	suppression of tumorigenicity 7 like
54887	UHRF1BP1	UHRF1 binding protein 1
54896	PQLC2	PQ loop repeat containing 2
54900	LAX1	lymphocyte transmembrane adaptor 1
54929	TMEM161A	transmembrane protein 161A
54949	SDHAF2	succinate dehydrogenase complex assembly factor 2
54973	CPSF3L	cleavage and polyadenylation specific factor 3-like
54976	C20orf27	chromosome 20 open reading frame 27
54986	ULK4	unc-51-like kinase 4 (C. elegans)
55020	TTC38	tetratricopeptide repeat domain 38
55041	PLEKHB2	pleckstrin homology domain containing, family B (evectins) member 2
55101	ATP5SL	ATP5S-like
55132	LARP1B	La ribonucleoprotein domain family, member 1B
55152	DALRD3	DALR anticodon binding domain containing 3
55173	MRPS10	mitochondrial ribosomal protein S10
55180	LINS	lines homolog (Drosophila)
55209	SETD5	SET domain containing 5
55213	RCBTB1	regulator of chromosome condensation (RCC1) and BTB (POZ) domain containing protein 1
55218	EXD2	exonuclease 3'-5' domain containing 2
55222	LRRC20	leucine rich repeat containing 20
55249	YY1AP1	YY1 associated protein 1
55251	PCMTD2	protein-L-isoaspartate (D-aspartate) O-methyltransferase domain containing 2
55291	PPP6R3	protein phosphatase 6, regulatory subunit 3
55297	CCDC91	coiled-coil domain containing 91
55327	LIN7C	lin-7 homolog C (C. elegans)
55370	PPP4R1L	protein phosphatase 4, regulatory subunit 1-like
55486	PARL	presenilin associated, rhomboid-like
55526	DHTKD1	dehydrogenase E1 and transketolase domain containing 1
55536	CDCA7L	cell division cycle associated 7-like
5554	PRH1	proline-rich protein HaeIII subfamily 1
55613	MTMR8	myotubularin related protein 8
55617	TASP1	taspase, threonine aspartase, 1
55620	STAP2	signal transducing adaptor family member 2
55644	OSGEP	O-sialoglycoprotein endopeptidase
55665	URGCP	upregulator of cell proliferation
55667	DENND4C	DENN/MADD domain containing 4C
55672	NBPF1	neuroblastoma breakpoint family, member 1
55689	YEATS2	YEATS domain containing 2
55700	MAP7D1	MAP7 domain containing 1

55705	IPO9	importin 9										
55717	WDR11	WD repeat domain 11										
55735	DNAJC11	DnaJ (Hsp40) homolog, subfamily C, member 11										
55737	VPS35	vacuolar protein sorting 35 homolog (S. cerevisiae)										
55756	INTS9	integrator complex subunit 9										
55795	PCID2	PCI domain containing 2										
55823	VPS11	vacuolar protein sorting 11 homolog (S. cerevisiae)										
55833	UBAP2	ubiquitin associated protein 2										
55863	TMEM126B	transmembrane protein 126B										
5612	PRKRIR	protein-kinase, interferon-inducible double stranded RNA dependent inhibitor, repressor of (P58 repressor)										
56681	SAR1A	SAR1 homolog A (S. cerevisiae)										
56834	GPR137	G protein-coupled receptor 137										
56850	GRIPAP1	GRIP1 associated protein 1										
56940	DUSP22	dual specificity phosphatase 22										
56943	ENY2	enhancer of yellow 2 homolog (Drosophila)										
56947	MFF	mitochondrial fission factor										
56965	PARP6	poly (ADP-ribose) polymerase family, member 6										
56996	SLC12A9	solute carrier family 12 (potassium/chloride transporters), member 9										
57140	RNPEPL1	arginyl aminopeptidase (aminopeptidase B)-like 1										
57149	LYRM1	LYR motif containing 1										
57186	RALGAPA2	Ral GTPase activating protein, alpha subunit 2 (catalytic)										
57223	SMEK2	SMEK homolog 2, suppressor of mek1 (Dictyostelium)										
57396	CLK4	CDC-like kinase 4										
57404	CYP20A1	cytochrome P450, family 20, subfamily A, polypeptide 1										
57410	SCYL1	SCY1-like 1 (S. cerevisiae)										
57489	ODF2L	outer dense fiber of sperm tails 2-like										
57492	ARID1B	AT rich interactive domain 1B (SWI1-like)										
57507	ZNF608	zinc finger protein 608										
57519	STARD9	StAR-related lipid transfer (START) domain containing 9										
57535	KIAA1324	KIAA1324										
57544	TXNDC16	thioredoxin domain containing 16										
57552	NCEH1	neutral cholesterol ester hydrolase 1										
57577	KIAA1407	KIAA1407										
57614	KIAA1468	KIAA1468										
57620	STIM2	stromal interaction molecule 2										
57636	ARHGAP23	Rho GTPase activating protein 23										
57695	USP37	ubiquitin specific peptidase 37										
57724	EPG5	ectopic P-granules autophagy protein 5 homolog (C. elegans)										
5775	PTPN4	protein tyrosine phosphatase, non-receptor type 4 (megakaryocyte)										
58475	MS4A7	membrane-spanning 4-domains, subfamily A, member 7										
58487	CREBZF	CREB/ATF bZIP transcription factor										
585	BBS4	Bardet-Biedl syndrome 4										
58516	FAM60A	family with sequence similarity 60, member A										
5863	RGL2	ral guanine nucleotide dissociation stimulator-like 2										
60674	GAS5	growth arrest-specific 5 (non-protein coding)										
60686	C14orf93	chromosome 14 open reading frame 93										
6302	TSPAN31	tetraspanin 31										
63892	THADA	thyroid adenoma associated										
63897	HEATR6	HEAT repeat containing 6										
63901	FAM111A	family with sequence similarity 111, member A										
63922	CHTF18	CTF18, chromosome transmission fidelity factor 18 homolog (S. cerevisiae)										
63929	XPNPEP3	X-prolyl aminopeptidase (aminopeptidase P) 3, putative										
64005	MYO1G	myosin IG										
64118	DUS1L	dihydrouridine synthase 1-like (S. cerevisiae)										
641590	NBPF20	neuroblastoma breakpoint family, member 20										
64174	DPEP2	dipeptidase 2										
641977	SEPT7P2	septin 7 pseudogene 2										
64210	MMS19	MMS19 nucleotide excision repair homolog (S. cerevisiae)										
642819	ZNF487P	zinc finger protein 487, pseudogene										
64319	FBRS	fibrosin										
64328	XPO4	exportin 4										
64427	TTC31	tetratricopeptide repeat domain 31										
64432	MRPS25	mitochondrial ribosomal protein S25										
644815	FAM83G	family with sequence similarity 83, member G										
645784	ANKRD36BP2	ankyrin repeat domain 36B pseudogene 2										
64595	TTTY15	testis-specific transcript, Y-linked 15 (non-protein coding)										
64755	C16orf58	chromosome 16 open reading frame 58										
64766	S100PBP	S100P binding protein										
64780	MICAL1	microtubule associated monoxygenase, calponin and LIM domain containing 1										
64783	RBM15	RNA binding motif protein 15										
64943	NT5DC2	5'-nucleotidase domain containing 2										
65056	GPBP1	GC-rich promoter binding protein 1										
65094	JMJD4	jumonji domain containing 4										
651746	ANKRD33B	ankyrin repeat domain 33B										
65250	C5orf42	chromosome 5 open reading frame 42										
65258	MPPE1	metallophosphoesterase 1										
65979	PHACTR4	phosphatase and actin regulator 4										
65980	BRD9	bromodomain containing 9										
6793	STK10	serine/threonine kinase 10										
6894	TARBP1	TAR (HIV-1) RNA binding protein 1										
6919	TCEA2	transcription elongation factor A (SII), 2										
7023	TFAP4	transcription factor AP-4 (activating enhancer binding protein 4)										
7072	TIA1	TIA1 cytotoxic granule-associated RNA binding protein										
7165	TPD52L2	tumor protein D52-like 2										
7260	TSSC1	tumor suppressing subtransferable candidate 1										
730092	RRN3P1	RNA polymerase I transcription factor homolog (S. cerevisiae) pseudogene 1										
7543	ZFX	zinc finger protein, X-linked										
755	C21orf2	chromosome 21 open reading frame 2										
7626	ZNF75D	zinc finger protein 75D										
7627	ZNF75A	zinc finger protein 75a										
7678	ZNF124	zinc finger protein 124										
7748	ZNF195	zinc finger protein 195										
7775	ZNF232	zinc finger protein 232										
7871	SLMAP	sarcolemma associated protein										
78994	PRR14	proline rich 14										
78996	C7orf49	chromosome 7 open reading frame 49										
79022	TMEM106C	transmembrane protein 106C										
79065	ATG9A	ATG9 autophagy related 9 homolog A (S. cerevisiae)										
79090	TRAPPC6A	trafficking protein particle complex 6A										
79091	METTL22	methyltransferase like 22										
79157	MFSD11	major facilitator superfamily domain containing 11										
79415	C17orf62	chromosome 17 open reading frame 62										
79568	C2orf47	chromosome 2 open reading frame 47										
79600	TCTN1	tectonic family member 1										
79675	FASTKD1	FAST kinase domains 1										
79703	C11orf80	chromosome 11 open reading frame 80										
79705	LRRK1	leucine-rich repeat kinase 1										
79712	GTDC1	glycosyltransferase-like domain containing 1										
79719	AAGAB	alpha- and gamma-adaptin binding protein										
79752	ZFAND1	zinc finger, AN1-type domain 1										
79763	ISOC2	isochorismatase domain containing 2										
79792	GSDMD	gasdermin D										
79819	WDR78	WD repeat domain 78										
79848	CSPP1	centrosome and spindle pole associated protein 1										
79879	CCDC134	coiled-coil domain containing 134										
79893	GGNBP2	gametogenetin binding protein 2										
79930	DOK3	docking protein 3										
79958	DENND1C	DENN/MADD domain containing 1C										
79979	TRMT2B	TRM2 tRNA methyltransferase 2 homolog B (S. cerevisiae)										
80011	FAM192A	family with sequence similarity 192, member A										
80013	FAM188A	family with sequence similarity 188, member A										
80021	TMEM62	transmembrane protein 62										
80174	DBF4B	DBF4 homolog B (S. cerevisiae)										
80205	CHD9	chromodomain helicase DNA binding protein 9										
80208	SPG11	spastic paraplegia 11 (autosomal recessive)										
80221	ACSF2	acyl-CoA synthetase family member 2										
80255	SLC35F5	solute carrier family 35, member F5										
80279	CDK5RAP3	CDK5 regulatory subunit associated protein 3										
80305	TRABD	TraB domain containing										
80724	ACAD10	acyl-CoA dehydrogenase family, member 10										
80790	CMIP	c-Maf inducing protein										
80817	CEP44	centrosomal protein 44kDa										
8131	NPRL3	nitrogen permease regulator-like 3 (S. cerevisiae)										
81502	HM13	histocompatibility (minor) 13										
81532	MOB2	MOB kinase activator 2										
81573	ANKRD13C	ankyrin repeat domain 13C										
81628	TSC22D4	TSC22 domain family, member 4										
81844	TRIM56	tripartite motif containing 56										
81847	RNF146	ring finger protein 146										
81853	TMEM14B	transmembrane protein 14B										
81890	QTRT1	queuine tRNA-ribosyltransferase 1										
8287	USP9Y	ubiquitin specific peptidase 9, Y-linked										
83596	BCL2L12	BCL2-like 12 (proline rich)										
83607	AMMECR1L	AMME chromosomal region gene 1-like										
83692	CD99L2	CD99 molecule-like 2										
83706	FERMT3	fermitin family member 3										
83707	TRPT1	tRNA phosphotransferase 1										
83746	L3MBTL2	l(3)mbt-like 2 (Drosophila)										
83930	STARD3NL	STARD3 N-terminal like										
83985	SPNS1	spinster homolog 1 (Drosophila)										
84074	QRICH2	glutamine rich 2										
84081	NSRP1	nuclear speckle splicing regulatory protein 1										
84083	ZRANB3	zinc finger, RAN-binding domain containing 3										
84100	ARL6	ADP-ribosylation factor-like 6										
84128	WDR75	WD repeat domain 75										
84129	ACAD11	acyl-CoA dehydrogenase family, member 11										
84162	KIAA1109	KIAA1109										
8418	CMAHP	cytidine monophospho-N-acetylneuraminic acid hydroxylase, pseudogene										
84181	CHD6	chromodomain helicase DNA binding protein 6										
84196	USP48	ubiquitin specific peptidase 48										
84248	FYTTD1	forty-two-three domain containing 1										
84268	RPAIN	RPA interacting protein										
84287	ZDHHC16	zinc finger, DHHC-type containing 16										
84304	NUDT22	nudix (nucleoside diphosphate linked moiety X)-type motif 22										
84307	ZNF397	zinc finger protein 397										
84318	CCDC77	coiled-coil domain containing 77										
84324	SARNP	SAP domain containing ribonucleoprotein										
84326	C16orf13	chromosome 16 open reading frame 13										
84456	L3MBTL3	l(3)mbt-like 3 (Drosophila)										
84461	NEURL4	neuralized homolog 4 (Drosophila)										
8458	TTF2	transcription termination factor, RNA polymerase II										
84614	ZBTB37	zinc finger and BTB domain containing 37										
84656	GLYR1	glyoxylate reductase 1 homolog (Arabidopsis)										
84680	ACCS	1-aminocyclopropane-1-carboxylate synthase homolog (Arabidopsis)(non-functional)										
84722	PSRC1	proline/serine-rich coiled-coil 1										
8473	OGT	O-linked N-acetylglucosamine (GlcNAc) transferase (UDP-N-acetylglucosamine:polypeptide-N-acetylglucosaminyl transferase)										
84750	FUT10	fucosyltransferase 10 (alpha (1,3) fucosyltransferase)										
84824	FCRLA	Fc receptor-like A										
84896	ATAD1	ATPase family, AAA domain containing 1										
84901	NFATC2IP	nuclear factor of activated T-cells, cytoplasmic, calcineurin-dependent 2 interacting protein										
84908	FAM136A	family with sequence similarity 136, member A										
8495	PPFIBP2	PTPRF interacting protein, binding protein 2 (liprin beta 2)										
84958	SYTL1	synaptotagmin-like 1										
85014	TMEM141	transmembrane protein 141										
85451	UNK	unkempt homolog (Drosophila)										
8548	BLZF1	basic leucine zipper nuclear factor 1										
8576	STK16	serine/threonine kinase 16										
889	KRIT1	KRIT1, ankyrin repeat containing										
8904	CPNE1	copine I										
8939	FUBP3	far upstream element (FUSE) binding protein 3										
8975	USP13	ubiquitin specific peptidase 13 (isopeptidase T-3)										
89790	SIGLEC10	sialic acid binding Ig-like lectin 10										
89849	ATG16L2	ATG16 autophagy related 16-like 2 (S. cerevisiae)										
89891	WDR34	WD repeat domain 34										
89894	TMEM116	transmembrane protein 116										
89970	RSPRY1	ring finger and SPRY domain containing 1										
90313	TP53I13	tumor protein p53 inducible protein 13										
90835	C16orf93	chromosome 16 open reading frame 93										
91272	BOD1	biorientation of chromosomes in cell division 1										
91298	C12orf29	chromosome 12 open reading frame 29										
91316	GUSBP11	glucuronidase, beta pseudogene 11										
91319	DERL3	Der1-like domain family, member 3										
91355	LRP5L	low density lipoprotein receptor-related protein 5-like										
91433	RCCD1	RCC1 domain containing 1										
91526	ANKRD44	ankyrin repeat domain 44										
9159	PCSK7	proprotein convertase subtilisin/kexin type 7										
91607	SLFN11	schlafen family member 11										
92017	SNX29	sorting nexin 29										
9204	ZMYM6	zinc finger, MYM-type 6										
9205	ZMYM5	zinc finger, MYM-type 5										
92105	INTS4	integrator complex subunit 4										
92270	ATP6AP1L	ATPase, H+ transporting, lysosomal accessory protein 1-like										
92399	MRRF	mitochondrial ribosome recycling factor										
92482	BBIP1	BBSome interacting protein 1										
92714	ARRDC1	arrestin domain containing 1										
9317	PTER	phosphotriesterase related										
9324	HMGN3	high mobility group nucleosomal binding domain 3										
9325	TRIP4	thyroid hormone receptor interactor 4										
93323	HAUS8	HAUS augmin-like complex, subunit 8										
93627	TBCK	TBC1 domain containing kinase										
94097	SFXN5	sideroflexin 5										
9535	GMFG	glia maturation factor, gamma										
9557	CHD1L	chromodomain helicase DNA binding protein 1-like										
9584	RBM39	RNA binding motif protein 39										
963	CD53	CD53 molecule										
9637	FEZ2	fasciculation and elongation protein zeta 2 (zygin II)										
9681	DEPDC5	DEP domain containing 5										
9701	PPP6R2	protein phosphatase 6, regulatory subunit 2										
9753	ZSCAN12	zinc finger and SCAN domain containing 12										
9772	KIAA0195	KIAA0195										
9783	RIMS3	regulating synaptic membrane exocytosis 3										
9841	ZBTB24	zinc finger and BTB domain containing 24										
9849	ZNF518A	zinc finger protein 518A										
987	LRBA	LPS-responsive vesicle trafficking, beach and anchor containing										
9877	ZC3H11A	zinc finger CCCH-type containing 11A										
9898	UBAP2L	ubiquitin associated protein 2-like										
9909	DENND4B	DENN/MADD domain containing 4B										
9923	ZBTB40	zinc finger and BTB domain containing 40										

Supplementary Table 9: MINO Alternative Splicing MsigDB Analysis

Overlap Results	
Collection(s):	CP, CP:BIOCARTA, CP:KEGG, CP:REACTOME, H
# overlaps shown:	10
# genesets in collections:	1379
# genes in comparison (n):	983
# genes in universe (N):	45956

Gene Set Name	# Genes in Gene Set (K)	Description	# Genes in Overlap (k)	k/K	p-value	FDR q-value
REACTOME_CELL_CYCLE	421	Genes involved in Cell Cycle	39	0.0926	2.75E-14	3.79E-11
HALLMARK_MITOTIC_SPINDLE	200	Genes important for mitotic spindle assembly.	26	0.13	2.49E-13	1.17E-10
REACTOME_METABOLISM_OF_RNA	330	Genes involved in Metabolism of RNA	33	0.1	3.18E-13	1.17E-10
REACTOME_DNA_REPAIR	112	Genes involved in DNA Repair	20	0.1786	3.40E-13	1.17E-10
HALLMARK_E2F_TARGETS	200	Genes encoding cell cycle related targets of E2F transcription factors.	25	0.125	1.74E-12	4.81E-10
REACTOME_IMMUNE_SYSTEM	933	Genes involved in Immune System	57	0.0611	2.34E-12	5.38E-10
KEGG_RIBOSOME	88	Ribosome	17	0.1932	5.30E-12	1.04E-09
REACTOME_TRANSLATION	222	Genes involved in Translation	25	0.1126	1.79E-11	3.08E-09
REACTOME_METABOLISM_OF_MRNA	284	Genes involved in Metabolism of mRNA	28	0.0986	2.75E-11	3.90E-09
REACTOME_CELL_CYCLE_MITOTIC	325	Genes involved in Cell Cycle, Mitotic	30	0.0923	2.83E-11	3.90E-09

Gene/Gene Set Overlap Matrix

Entrez Gene Id	Gene Symbol	Gene Description	REACTOME_CELL_CYCLE	HALLMARK_MITOTIC_SPINDLE	REACTOME_METABOLISM_OF_RNA	REACTOME_DNA_REPAIR	HALLMARK_E2F_TARGETS	REACTOME_IMMUNE_SYSTEM	KEGG_RIBOSOME	REACTOME_TRANSLATION	REACTOME_METABOLISM_OF_MRNA	REACTOME_CELL_CYCLE_MITOTIC
996	CDC27	cell division cycle 27 homolog (S. cerevisiae)	REACTOME_CELL_CYCLE	HALLMARK_MITOTIC_SPINDLE				REACTOME_IMMUNE_SYSTEM				REACTOME_CELL_CYCLE_MITOTIC
23332	CLASP1	cytoplasmic linker associated protein 1	REACTOME_CELL_CYCLE	HALLMARK_MITOTIC_SPINDLE								REACTOME_CELL_CYCLE_MITOTIC
9735	KNTC1	kinetochore associated 1	REACTOME_CELL_CYCLE	HALLMARK_MITOTIC_SPINDLE								REACTOME_CELL_CYCLE_MITOTIC
5116	PCNT	pericentrin	REACTOME_CELL_CYCLE	HALLMARK_MITOTIC_SPINDLE								REACTOME_CELL_CYCLE_MITOTIC
5108	PCM1	pericentriolar material 1	REACTOME_CELL_CYCLE	HALLMARK_MITOTIC_SPINDLE								REACTOME_CELL_CYCLE_MITOTIC
55125	CEP192	centrosomal protein 192kDa	REACTOME_CELL_CYCLE	HALLMARK_MITOTIC_SPINDLE								REACTOME_CELL_CYCLE_MITOTIC
55835	CENPJ	centromere protein J	REACTOME_CELL_CYCLE	HALLMARK_MITOTIC_SPINDLE								REACTOME_CELL_CYCLE_MITOTIC
57122	NUP107	nucleoporin 107kDa	REACTOME_CELL_CYCLE		REACTOME_METABOLISM_OF_RNA		HALLMARK_E2F_TARGETS	REACTOME_IMMUNE_SYSTEM				REACTOME_CELL_CYCLE_MITOTIC
7514	XPO1	exportin 1 (CRM1 homolog, yeast)	REACTOME_CELL_CYCLE		REACTOME_METABOLISM_OF_RNA		HALLMARK_E2F_TARGETS				REACTOME_METABOLISM_OF_MRNA	REACTOME_CELL_CYCLE_MITOTIC
5682	PSMA1	proteasome (prosome, macropain) subunit, alpha type, 1	REACTOME_CELL_CYCLE		REACTOME_METABOLISM_OF_RNA			REACTOME_IMMUNE_SYSTEM			REACTOME_METABOLISM_OF_MRNA	REACTOME_CELL_CYCLE_MITOTIC
5717	PSMD11	proteasome (prosome, macropain) 26S subunit, non-ATPase, 11	REACTOME_CELL_CYCLE		REACTOME_METABOLISM_OF_RNA			REACTOME_IMMUNE_SYSTEM			REACTOME_METABOLISM_OF_MRNA	REACTOME_CELL_CYCLE_MITOTIC
348995	NUP43	nucleoporin 43kDa	REACTOME_CELL_CYCLE		REACTOME_METABOLISM_OF_RNA			REACTOME_IMMUNE_SYSTEM				REACTOME_CELL_CYCLE_MITOTIC
5424	POLD1	polymerase (DNA directed), delta 1, catalytic subunit 125kDa	REACTOME_CELL_CYCLE			REACTOME_DNA_REPAIR	HALLMARK_E2F_TARGETS					REACTOME_CELL_CYCLE_MITOTIC
472	ATM	ataxia telangiectasia mutated	REACTOME_CELL_CYCLE			REACTOME_DNA_REPAIR						
23649	POLA2	polymerase (DNA directed), alpha 2 (70kD subunit)	REACTOME_CELL_CYCLE				HALLMARK_E2F_TARGETS					REACTOME_CELL_CYCLE_MITOTIC
5810	RAD1	RAD1 homolog (S. pombe)	REACTOME_CELL_CYCLE				HALLMARK_E2F_TARGETS					
11200	CHEK2	CHK2 checkpoint homolog (S. pombe)	REACTOME_CELL_CYCLE				HALLMARK_E2F_TARGETS					
51529	ANAPC11	anaphase promoting complex subunit 11	REACTOME_CELL_CYCLE					REACTOME_IMMUNE_SYSTEM				REACTOME_CELL_CYCLE_MITOTIC
4193	MDM2	Mdm2 p53 binding protein homolog (mouse)	REACTOME_CELL_CYCLE					REACTOME_IMMUNE_SYSTEM				
8318	CDC45	cell division cycle 45 homolog (S. cerevisiae)	REACTOME_CELL_CYCLE									REACTOME_CELL_CYCLE_MITOTIC
5933	RBL1	retinoblastoma-like 1 (p107)	REACTOME_CELL_CYCLE									REACTOME_CELL_CYCLE_MITOTIC
5001	ORC5	origin recognition complex, subunit 5	REACTOME_CELL_CYCLE									REACTOME_CELL_CYCLE_MITOTIC
5000	ORC4	origin recognition complex, subunit 4	REACTOME_CELL_CYCLE									REACTOME_CELL_CYCLE_MITOTIC
84515	MCM8	minichromosome maintenance complex component 8	REACTOME_CELL_CYCLE									REACTOME_CELL_CYCLE_MITOTIC
995	CDC25C	cell division cycle 25 homolog C (S. pombe)	REACTOME_CELL_CYCLE									REACTOME_CELL_CYCLE_MITOTIC
11130	ZWINT	ZW10 interactor	REACTOME_CELL_CYCLE									REACTOME_CELL_CYCLE_MITOTIC
2491	CENPI	centromere protein I	REACTOME_CELL_CYCLE									REACTOME_CELL_CYCLE_MITOTIC
79003	MIS12	MIS12, MIND kinetochore complex component, homolog (S. pombe)	REACTOME_CELL_CYCLE									REACTOME_CELL_CYCLE_MITOTIC
80152	CENPT	centromere protein T	REACTOME_CELL_CYCLE									REACTOME_CELL_CYCLE_MITOTIC
22897	CEP164	centrosomal protein 164kDa	REACTOME_CELL_CYCLE									REACTOME_CELL_CYCLE_MITOTIC
10142	AKAP9	A kinase (PRKA) anchor protein (yotiao) 9	REACTOME_CELL_CYCLE									REACTOME_CELL_CYCLE_MITOTIC
8636	SSNA1	Sjogren syndrome nuclear autoantigen 1	REACTOME_CELL_CYCLE									REACTOME_CELL_CYCLE_MITOTIC
1859	DYRK1A	dual-specificity tyrosine-(Y)-phosphorylation regulated kinase 1A	REACTOME_CELL_CYCLE									REACTOME_CELL_CYCLE_MITOTIC
132660	LIN54	lin-54 homolog (C. elegans)	REACTOME_CELL_CYCLE									REACTOME_CELL_CYCLE_MITOTIC
7329	UBE2I	ubiquitin-conjugating enzyme E2I	REACTOME_CELL_CYCLE									
84126	ATRIP	ATR interacting protein	REACTOME_CELL_CYCLE									
27127	SMC1B	structural maintenance of chromosomes 1B	REACTOME_CELL_CYCLE									
23345	SYNE1	spectrin repeat containing, nuclear envelope 1	REACTOME_CELL_CYCLE									
10388	SYCP2	synaptonemal complex protein 2	REACTOME_CELL_CYCLE									
3831	KLC1	kinesin light chain 1		HALLMARK_MITOTIC_SPINDLE				REACTOME_IMMUNE_SYSTEM				
6904	TBCD	tubulin folding cofactor D		HALLMARK_MITOTIC_SPINDLE								
9055	PRC1	protein regulator of cytokinesis 1		HALLMARK_MITOTIC_SPINDLE								
1794	DOCK2	dedicator of cytokinesis 2		HALLMARK_MITOTIC_SPINDLE								
6709	SPTAN1	spectrin, alpha, non-erythrocytic 1 (alpha-fodrin)		HALLMARK_MITOTIC_SPINDLE								
1739	DLG1	discs, large homolog 1 (Drosophila)		HALLMARK_MITOTIC_SPINDLE								
10928	RALBP1	ralA binding protein 1		HALLMARK_MITOTIC_SPINDLE								
10013	HDAC6	histone deacetylase 6		HALLMARK_MITOTIC_SPINDLE								
2316	FLNA	filamin A, alpha		HALLMARK_MITOTIC_SPINDLE								
4763	NF1	neurofibromin 1		HALLMARK_MITOTIC_SPINDLE								
64857	PLEKHG2	pleckstrin homology domain containing, family G (with RhoGef domain) member 2		HALLMARK_MITOTIC_SPINDLE								
10128	LRPPRC	leucine-rich PPR-motif containing		HALLMARK_MITOTIC_SPINDLE								
51735	RAPGEF6	Rap guanine nucleotide exchange factor (GEF) 6		HALLMARK_MITOTIC_SPINDLE								
23192	ATG4B	ATG4 autophagy related 4 homolog B (S. cerevisiae)		HALLMARK_MITOTIC_SPINDLE								
51174	TUBD1	tubulin, delta 1		HALLMARK_MITOTIC_SPINDLE								
5311	PKD2	polycystic kidney disease 2 (autosomal dominant)		HALLMARK_MITOTIC_SPINDLE								
54443	ANLN	anillin, actin binding protein		HALLMARK_MITOTIC_SPINDLE								
84376	HOOK3	hook homolog 3 (Drosophila)		HALLMARK_MITOTIC_SPINDLE								
9771	RAPGEF5	Rap guanine nucleotide exchange factor (GEF) 5		HALLMARK_MITOTIC_SPINDLE								
23165	NUP205	nucleoporin 205kDa			REACTOME_METABOLISM_OF_RNA		HALLMARK_E2F_TARGETS	REACTOME_IMMUNE_SYSTEM				
9883	POM121	POM121 membrane glycoprotein			REACTOME_METABOLISM_OF_RNA			REACTOME_IMMUNE_SYSTEM				
6194	RPS6	ribosomal protein S6			REACTOME_METABOLISM_OF_RNA				KEGG_RIBOSOME	REACTOME_TRANSLATION	REACTOME_METABOLISM_OF_MRNA	
6128	RPL6	ribosomal protein L6			REACTOME_METABOLISM_OF_RNA				KEGG_RIBOSOME	REACTOME_TRANSLATION	REACTOME_METABOLISM_OF_MRNA	
6164	RPL34	ribosomal protein L34			REACTOME_METABOLISM_OF_RNA				KEGG_RIBOSOME	REACTOME_TRANSLATION	REACTOME_METABOLISM_OF_MRNA	
6175	RPLP0	ribosomal protein, large, P0			REACTOME_METABOLISM_OF_RNA				KEGG_RIBOSOME	REACTOME_TRANSLATION	REACTOME_METABOLISM_OF_MRNA	
6203	RPS9	ribosomal protein S9			REACTOME_METABOLISM_OF_RNA				KEGG_RIBOSOME	REACTOME_TRANSLATION	REACTOME_METABOLISM_OF_MRNA	
6223	RPS19	ribosomal protein S19			REACTOME_METABOLISM_OF_RNA				KEGG_RIBOSOME	REACTOME_TRANSLATION	REACTOME_METABOLISM_OF_MRNA	
6201	RPS7	ribosomal protein S7			REACTOME_METABOLISM_OF_RNA				KEGG_RIBOSOME	REACTOME_TRANSLATION	REACTOME_METABOLISM_OF_MRNA	
6227	RPS21	ribosomal protein S21			REACTOME_METABOLISM_OF_RNA				KEGG_RIBOSOME	REACTOME_TRANSLATION	REACTOME_METABOLISM_OF_MRNA	
6230	RPS25	ribosomal protein S25			REACTOME_METABOLISM_OF_RNA				KEGG_RIBOSOME	REACTOME_TRANSLATION	REACTOME_METABOLISM_OF_MRNA	
6134	RPL10	ribosomal protein L10			REACTOME_METABOLISM_OF_RNA				KEGG_RIBOSOME	REACTOME_TRANSLATION	REACTOME_METABOLISM_OF_MRNA	
6139	RPL17	ribosomal protein L17			REACTOME_METABOLISM_OF_RNA				KEGG_RIBOSOME	REACTOME_TRANSLATION	REACTOME_METABOLISM_OF_MRNA	
6142	RPL18A	ribosomal protein L18a			REACTOME_METABOLISM_OF_RNA				KEGG_RIBOSOME	REACTOME_TRANSLATION	REACTOME_METABOLISM_OF_MRNA	
6144	RPL21	ribosomal protein L21			REACTOME_METABOLISM_OF_RNA				KEGG_RIBOSOME	REACTOME_TRANSLATION	REACTOME_METABOLISM_OF_MRNA	
6156	RPL30	ribosomal protein L30			REACTOME_METABOLISM_OF_RNA				KEGG_RIBOSOME	REACTOME_TRANSLATION	REACTOME_METABOLISM_OF_MRNA	
6160	RPL31	ribosomal protein L31			REACTOME_METABOLISM_OF_RNA				KEGG_RIBOSOME	REACTOME_TRANSLATION	REACTOME_METABOLISM_OF_MRNA	
6161	RPL32	ribosomal protein L32			REACTOME_METABOLISM_OF_RNA				KEGG_RIBOSOME	REACTOME_TRANSLATION	REACTOME_METABOLISM_OF_MRNA	
6171	RPL41	ribosomal protein L41			REACTOME_METABOLISM_OF_RNA				KEGG_RIBOSOME	REACTOME_TRANSLATION	REACTOME_METABOLISM_OF_MRNA	
6208	RPS14	ribosomal protein S14			REACTOME_METABOLISM_OF_RNA					REACTOME_TRANSLATION	REACTOME_METABOLISM_OF_MRNA	
51013	EXOSC1	exosome component 1			REACTOME_METABOLISM_OF_RNA						REACTOME_METABOLISM_OF_MRNA	
5393	EXOSC9	exosome component 9			REACTOME_METABOLISM_OF_RNA						REACTOME_METABOLISM_OF_MRNA	
4848	CNOT2	CCR4-NOT transcription complex, subunit 2			REACTOME_METABOLISM_OF_RNA						REACTOME_METABOLISM_OF_MRNA	
4850	CNOT4	CCR4-NOT transcription complex, subunit 4			REACTOME_METABOLISM_OF_RNA						REACTOME_METABOLISM_OF_MRNA	
57472	CNOT6	CCR4-NOT transcription complex, subunit 6			REACTOME_METABOLISM_OF_RNA						REACTOME_METABOLISM_OF_MRNA	
10605	PAIP1	poly(A) binding protein interacting protein 1			REACTOME_METABOLISM_OF_RNA						REACTOME_METABOLISM_OF_MRNA	
8125	ANP32A	acidic (leucine-rich) nuclear phosphoprotein 32 family, member A			REACTOME_METABOLISM_OF_RNA						REACTOME_METABOLISM_OF_MRNA	
8487	GEMIN2	gem (nuclear organelle) associated protein 2			REACTOME_METABOLISM_OF_RNA							
4361	MRE11A	MRE11 meiotic recombination 11 homolog A (S. cerevisiae)				REACTOME_DNA_REPAIR	HALLMARK_E2F_TARGETS					
1161	ERCC8	excision repair cross-complementing rodent repair deficiency, complementation group 8				REACTOME_DNA_REPAIR						
328	APEX1	APEX nuclease (multifunctional DNA repair enzyme) 1				REACTOME_DNA_REPAIR						
5423	POLB	polymerase (DNA directed), beta				REACTOME_DNA_REPAIR						
4913	NTHL1	nth endonuclease III-like 1 (E. coli)				REACTOME_DNA_REPAIR						
4968	OGG1	8-oxoguanine DNA glycosylase				REACTOME_DNA_REPAIR						
23583	SMUG1	single-strand-selective monofunctional uracil-DNA glycosylase 1				REACTOME_DNA_REPAIR						
4595	MUTYH	mutY homolog (E. coli)				REACTOME_DNA_REPAIR						
2177	FANCD2	Fanconi anemia, complementation group D2				REACTOME_DNA_REPAIR						
2178	FANCE	Fanconi anemia, complementation group E				REACTOME_DNA_REPAIR						
2175	FANCA	Fanconi anemia, complementation group A				REACTOME_DNA_REPAIR						
57697	FANCM	Fanconi anemia, complementation group M				REACTOME_DNA_REPAIR						
2965	GTF2H1	general transcription factor IIH, polypeptide 1, 62kDa				REACTOME_DNA_REPAIR						
5893	RAD52	RAD52 homolog (S. cerevisiae)				REACTOME_DNA_REPAIR						
2074	ERCC6	excision repair cross-complementing rodent repair deficiency, complementation group 6				REACTOME_DNA_REPAIR						
55775	TDP1	tyrosyl-DNA phosphodiesterase 1				REACTOME_DNA_REPAIR						
7507	XPA	xeroderma pigmentosum, complementation group A				REACTOME_DNA_REPAIR						
121642	ALKBH2	alkB, alkylation repair homolog 2 (E. coli)				REACTOME_DNA_REPAIR						
6434	TRA2B	transformer 2 beta homolog (Drosophila)					HALLMARK_E2F_TARGETS					
4673	NAP1L1	nucleosome assembly protein 1-like 1					HALLMARK_E2F_TARGETS					
4288	MKI67	antigen identified by monoclonal antibody Ki-67					HALLMARK_E2F_TARGETS					
7112	TMPO	thymopoietin					HALLMARK_E2F_TARGETS					
51691	NAA38	N(alpha)-acetyltransferase 38, NatC auxiliary subunit					HALLMARK_E2F_TARGETS					
6470	SHMT1	serine hydroxymethyltransferase 1 (soluble)					HALLMARK_E2F_TARGETS					
4292	MLH1	mutL homolog 1, colon cancer, nonpolyposis type 2 (E. coli)					HALLMARK_E2F_TARGETS					
9738	CCP110	centriolar coiled coil protein 110kDa					HALLMARK_E2F_TARGETS					
146909	KIF18B	kinesin family member 18B					HALLMARK_E2F_TARGETS					
253714	MMS22L	MMS22-like, DNA repair protein					HALLMARK_E2F_TARGETS					
29893	PSMC3IP	PSMC3 interacting protein					HALLMARK_E2F_TARGETS					
6941	TCF19	transcription factor 19					HALLMARK_E2F_TARGETS					
79677	SMC6	structural maintenance of chromosomes 6					HALLMARK_E2F_TARGETS					
83461	CDCA3	cell division cycle associated 3					HALLMARK_E2F_TARGETS					
8726	EED	embryonic ectoderm development					HALLMARK_E2F_TARGETS					
9833	MELK	maternal embryonic leucine zipper kinase					HALLMARK_E2F_TARGETS					
9924	PAN2	PAN2 poly(A) specific ribonuclease subunit homolog (S. cerevisiae)					HALLMARK_E2F_TARGETS					
5589	PRKCSH	protein kinase C substrate 80K-H						REACTOME_IMMUNE_SYSTEM				
7318	UBA7	ubiquitin-like modifier activating enzyme 7						REACTOME_IMMUNE_SYSTEM				
10075	HUWE1	HECT, UBA and WWE domain containing 1						REACTOME_IMMUNE_SYSTEM				
9820	CUL7	cullin 7						REACTOME_IMMUNE_SYSTEM				
6921	TCEB1	transcription elongation factor B (SIII), polypeptide 1 (15kDa, elongin C)						REACTOME_IMMUNE_SYSTEM				
8453	CUL2	cullin 2						REACTOME_IMMUNE_SYSTEM				
7337	UBE3A	ubiquitin protein ligase E3A						REACTOME_IMMUNE_SYSTEM				
7317	UBA1	ubiquitin-like modifier activating enzyme 1						REACTOME_IMMUNE_SYSTEM				
4591	TRIM37	tripartite motif containing 37						REACTOME_IMMUNE_SYSTEM				
55070	DET1	de-etiolated homolog 1 (Arabidopsis)						REACTOME_IMMUNE_SYSTEM				
3326	HSP90AB1	heat shock protein 90kDa alpha (cytosolic), class B member 1						REACTOME_IMMUNE_SYSTEM				
164	AP1G1	adaptor-related protein complex 1, gamma 1 subunit						REACTOME_IMMUNE_SYSTEM				
5970	RELA	v-rel reticuloendotheliosis viral oncogene homolog A (avian)						REACTOME_IMMUNE_SYSTEM				
3551	IKBKB	inhibitor of kappa light polypeptide gene enhancer in B-cells, kinase beta						REACTOME_IMMUNE_SYSTEM				
6885	MAP3K7	mitogen-activated protein kinase kinase kinase 7						REACTOME_IMMUNE_SYSTEM				
5335	PLCG1	phospholipase C, gamma 1						REACTOME_IMMUNE_SYSTEM				
3106	HLA-B	major histocompatibility complex, class I, B						REACTOME_IMMUNE_SYSTEM				
122769	LRR1	leucine rich repeat protein 1						REACTOME_IMMUNE_SYSTEM				
9520	NPEPPS	aminopeptidase puromycin sensitive						REACTOME_IMMUNE_SYSTEM				
5728	PTEN	phosphatase and tensin homolog						REACTOME_IMMUNE_SYSTEM				
973	CD79A	CD79a molecule, immunoglobulin-associated alpha						REACTOME_IMMUNE_SYSTEM				
5788	PTPRC	protein tyrosine phosphatase, receptor type, C						REACTOME_IMMUNE_SYSTEM				
2213	FCGR2B	Fc fragment of IgG, low affinity IIb, receptor (CD32)						REACTOME_IMMUNE_SYSTEM				
2475	MTOR	mechanistic target of rapamycin (serine/threonine kinase)						REACTOME_IMMUNE_SYSTEM				
3108	HLA-DMA	major histocompatibility complex, class II, DM alpha						REACTOME_IMMUNE_SYSTEM				
3111	HLA-DOA	major histocompatibility complex, class II, DO alpha						REACTOME_IMMUNE_SYSTEM				
5908	RAP1B	RAP1B, member of RAS oncogene family						REACTOME_IMMUNE_SYSTEM				
10120	ACTR1B	ARP1 actin-related protein 1 homolog B, centractin beta (yeast)						REACTOME_IMMUNE_SYSTEM				
5300	PIN1	peptidylprolyl cis/trans isomerase, NIMA-interacting 1						REACTOME_IMMUNE_SYSTEM				
4615	MYD88	myeloid differentiation primary response gene (88)						REACTOME_IMMUNE_SYSTEM				
51135	IRAK4	interleukin-1 receptor-associated kinase 4						REACTOME_IMMUNE_SYSTEM				
8672	EIF4G3	eukaryotic translation initiation factor 4 gamma, 3						REACTOME_IMMUNE_SYSTEM				
3841	KPNA5	karyopherin alpha 5 (importin alpha 6)						REACTOME_IMMUNE_SYSTEM				
960	CD44	CD44 molecule (Indian blood group)						REACTOME_IMMUNE_SYSTEM				
3663	IRF5	interferon regulatory factor 5						REACTOME_IMMUNE_SYSTEM				
6773	STAT2	signal transducer and activator of transcription 2, 113kDa						REACTOME_IMMUNE_SYSTEM				
79155	TNIP2	TNFAIP3 interacting protein 2						REACTOME_IMMUNE_SYSTEM				
5094	PCBP2	poly(rC) binding protein 2						REACTOME_IMMUNE_SYSTEM				
351	APP	amyloid beta (A4) precursor protein						REACTOME_IMMUNE_SYSTEM				
10010	TANK	TRAF family member-associated NFKB activator						REACTOME_IMMUNE_SYSTEM				
55593	OTUD5	OTU domain containing 5						REACTOME_IMMUNE_SYSTEM				
4205	MEF2A	myocyte enhancer factor 2A						REACTOME_IMMUNE_SYSTEM				
1386	ATF2	activating transcription factor 2						REACTOME_IMMUNE_SYSTEM				
81793	TLR10	toll-like receptor 10						REACTOME_IMMUNE_SYSTEM				
10695	CNPY3	canopy 3 homolog (zebrafish)						REACTOME_IMMUNE_SYSTEM				
6300	MAPK12	mitogen-activated protein kinase 12						REACTOME_IMMUNE_SYSTEM				
22861	NLRP1	NLR family, pyrin domain containing 1						REACTOME_IMMUNE_SYSTEM				
1968	EIF2S3	eukaryotic translation initiation factor 2, subunit 3 gamma, 52kDa								REACTOME_TRANSLATION		
3646	EIF3E	eukaryotic translation initiation factor 3, subunit E								REACTOME_TRANSLATION		
27335	EIF3K	eukaryotic translation initiation factor 3, subunit K								REACTOME_TRANSLATION		
1915	EEF1A1	eukaryotic translation elongation factor 1 alpha 1								REACTOME_TRANSLATION		
90701	SEC11C	SEC11 homolog C (S. cerevisiae)								REACTOME_TRANSLATION		
6748	SSR4	signal sequence receptor, delta								REACTOME_TRANSLATION		
1936	EEF1D	eukaryotic translation elongation factor 1 delta (guanine nucleotide exchange protein)								REACTOME_TRANSLATION		
10564	ARFGEF2	ADP-ribosylation factor guanine nucleotide-exchange factor 2 (brefeldin A-inhibited)										
9695	EDEM1	ER degradation enhancer, mannosidase alpha-like 1										
55741	EDEM2	ER degradation enhancer, mannosidase alpha-like 2										
56886	UGGT1	UDP-glucose glycoprotein glucosyltransferase 1										
5204	PFDN5	prefoldin subunit 5										
4121	MAN1A1	mannosidase, alpha, class 1A, member 1										
6480	ST6GAL1	ST6 beta-galactosamide alpha-2,6-sialyltranferase 1										
79868	ALG13	asparagine-linked glycosylation 13 homolog (S. cerevisiae)										
54872	PIGG	phosphatidylinositol glycan anchor biosynthesis, class G										
8733	GPAA1	glycosylphosphatidylinositol anchor attachment protein 1 homolog (yeast)										
2677	GGCX	gamma-glutamyl carboxylase										
331	XIAP	X-linked inhibitor of apoptosis										
10401	PIAS3	protein inhibitor of activated STAT, 3										
83737	ITCH	itchy E3 ubiquitin protein ligase homolog (mouse)										
26091	HERC4	hect domain and RLD 4										
8925	HERC1	hect (homologous to the E6-AP (UBE3A) carboxyl terminus) domain and RCC1 (CHC1)-like domain (RLD) 1										
3183	HNRNPC	heterogeneous nuclear ribonucleoprotein C (C1/C2)										
3376	IARS	isoleucyl-tRNA synthetase										
5478	PPIA	peptidylprolyl isomerase A (cyclophilin A)										
790	CAD	carbamoyl-phosphate synthetase 2, aspartate transcarbamylase, and dihydroorotase										
10399	GNB2L1	guanine nucleotide binding protein (G protein), beta polypeptide 2-like 1										
1665	DHX15	DEAH (Asp-Glu-Ala-His) box polypeptide 15										
2079	ERH	enhancer of rudimentary homolog (Drosophila)										
26156	RSL1D1	ribosomal L1 domain containing 1										
8761	PABPC4	poly(A) binding protein, cytoplasmic 4 (inducible form)										
11073	TOPBP1	topoisomerase (DNA) II binding protein 1										
22909	FAN1	FANCD2/FANCI-associated nuclease 1										
4087	SMAD2	SMAD family member 2										
9612	NCOR2	nuclear receptor corepressor 2										
9968	MED12	mediator complex subunit 12										
9477	MED20	mediator complex subunit 20										
905	CCNT2	cyclin T2										
2648	KAT2A	K(lysine) acetyltransferase 2A										
4221	MEN1	multiple endocrine neoplasia I										
10781	ZNF266	zinc finger protein 266										

10793	ZNF273	zinc finger protein 273										
148156	ZNF558	zinc finger protein 558										
162963	ZNF610	zinc finger protein 610										
285268	ZNF621	zinc finger protein 621										
57693	ZNF317	zinc finger protein 317										
7551	ZNF3	zinc finger protein 3										
7556	ZNF10	zinc finger protein 10										
7571	ZNF23	zinc finger protein 23 (KOX 16)										
7574	ZNF26	zinc finger protein 26										
7596	ZNF45	zinc finger protein 45										
7697	ZNF138	zinc finger protein 138										
7700	ZNF141	zinc finger protein 141										
7753	ZNF202	zinc finger protein 202										
7767	ZNF224	zinc finger protein 224										
84874	ZNF514	zinc finger protein 514										
90233	ZNF551	zinc finger protein 551										
55247	NEIL3	nei endonuclease VIII-like 3 (E. coli)										
8729	GBF1	golgi brefeldin A resistant guanine nucleotide exchange factor 1										
9276	COPB2	coatomer protein complex, subunit beta 2 (beta prime)										
11316	COPE	coatomer protein complex, subunit epsilon										
1315	COPB1	coatomer protein complex, subunit beta 1										
55738	ARFGAP1	ADP-ribosylation factor GTPase activating protein 1										
19	ABCA1	ATP-binding cassette, sub-family A (ABC1), member 1										
10059	DNM1L	dynamin 1-like										
5066	PAM	peptidylglycine alpha-amidating monooxygenase										
8675	STX16	syntaxin 16										
10282	BET1	blocked early in transport 1 homolog (S. cerevisiae)										
23033	DOPEY1	dopey family member 1										
2130	EWSR1	Ewing sarcoma breakpoint region 1										
7709	ZBTB17	zinc finger and BTB domain containing 17										
51720	UIMC1	ubiquitin interaction motif containing 1										
10914	PAPOLA	poly(A) polymerase alpha										
29894	CPSF1	cleavage and polyadenylation specific factor 1, 160kDa										
27316	RBMX	RNA binding motif protein, X-linked										
79869	CPSF7	cleavage and polyadenylation specific factor 7, 59kDa										
10181	RBM5	RNA binding motif protein 5										
3187	HNRNPH1	heterogeneous nuclear ribonucleoprotein H1 (H)										
55749	CCAR1	cell division cycle and apoptosis regulator 1										
9013	TAF1C	TATA box binding protein (TBP)-associated factor, RNA polymerase I, C, 110kDa										
30833	NT5C	5', 3'-nucleotidase, cytosolic										
30834	ZNRD1	zinc ribbon domain containing 1										
54487	DGCR8	DiGeorge syndrome critical region gene 8										
1716	DGUOK	deoxyguanosine kinase										
28996	HIPK2	homeodomain interacting protein kinase 2										
4194	MDM4	Mdm4 p53 binding protein homolog (mouse)										
1877	E4F1	E4F transcription factor 1										
3033	HADH	hydroxyacyl-CoA dehydrogenase										
38	ACAT1	acetyl-CoA acetyltransferase 1										
10919	EHMT2	euchromatic histone-lysine N-methyltransferase 2										
9869	SETDB1	SET domain, bifurcated 1										
6419	SETMAR	SET domain and mariner transposase fusion gene										
83852	SETDB2	SET domain, bifurcated 2										
84787	SUV420H2	suppressor of variegation 4-20 homolog 2 (Drosophila)										
34	ACADM	acyl-CoA dehydrogenase, C-4 to C-12 straight chain										
3155	HMGCL	3-hydroxymethyl-3-methylglutaryl-CoA lyase										
84693	MCEE	methylmalonyl CoA epimerase										
26275	HIBCH	3-hydroxyisobutyryl-CoA hydrolase										
10449	ACAA2	acetyl-CoA acyltransferase 2										
118	ADD1	adducin 1 (alpha)										
10106	CTDSP2	CTD (carboxy-terminal domain, RNA polymerase II, polypeptide A) small phosphatase 2										
1663	DDX11	DEAD/H (Asp-Glu-Ala-Asp/His) box polypeptide 11										
3156	HMGCR	3-hydroxy-3-methylglutaryl-CoA reductase										
254531	LPCAT4	lysophosphatidylcholine acyltransferase 4										
5244	ABCB4	ATP-binding cassette, sub-family B (MDR/TAP), member 4										
29841	GRHL1	grainyhead-like 1 (Drosophila)										
818	CAMK2G	calcium/calmodulin-dependent protein kinase II gamma										
1841	DTYMK	deoxythymidylate kinase (thymidylate kinase)										
10772	SRSF10	serine/arginine-rich splicing factor 10										
8899	PRPF4B	PRP4 pre-mRNA processing factor 4 homolog B (yeast)										
4644	MYO5A	myosin VA (heavy chain 12, myoxin)										
9015	TAF1A	TATA box binding protein (TBP)-associated factor, RNA polymerase I, A, 48kDa										
137492	VPS37A	vacuolar protein sorting 37 homolog A (S. cerevisiae)										
1806	DPYD	dihydropyrimidine dehydrogenase										
51176	LEF1	lymphoid enhancer-binding factor 1										
2232	FDXR	ferredoxin reductase										
6303	SAT1	spermidine/spermine N1-acetyltransferase 1										
6648	SOD2	superoxide dismutase 2, mitochondrial										
10622	POLR3G	polymerase (RNA) III (DNA directed) polypeptide G (32kD)										
56474	CTPS2	CTP synthase II										
3423	IDS	iduronate 2-sulfatase										
4669	NAGLU	N-acetylglucosaminidase, alpha										
10312	TCIRG1	T-cell, immune regulator 1, ATPase, H+ transporting, lysosomal V0 subunit A3										
1497	CTNS	cystinosin, lysosomal cystine transporter										
4125	MAN2B1	mannosidase, alpha, class 2B, member 1										
4126	MANBA	mannosidase, beta A, lysosomal										
23163	GGA3	golgi-associated, gamma adaptin ear containing, ARF binding protein 3										
1201	CLN3	ceroid-lipofuscinosis, neuronal 3										
26088	GGA1	golgi-associated, gamma adaptin ear containing, ARF binding protein 1										
9112	MTA1	metastasis associated 1										
4363	ABCC1	ATP-binding cassette, sub-family C (CFTR/MRP), member 1										
23116	FAM179B	family with sequence similarity 179, member B										
23469	PHF3	PHD finger protein 3										
27109	ATP5S	ATP synthase, H+ transporting, mitochondrial Fo complex, subunit s (factor B)										
7799	PRDM2	PR domain containing 2, with ZNF domain										
8295	TRRAP	transformation/transcription domain-associated protein										
5213	PFKM	phosphofructokinase, muscle										
83879	CDCA7	cell division cycle associated 7										
2260	FGFR1	fibroblast growth factor receptor 1										
1856	DVL2	dishevelled, dsh homolog 2 (Drosophila)										
10342	TFG	TRK-fused gene										
10498	CARM1	coactivator-associated arginine methyltransferase 1										
984	CDK11B	cyclin-dependent kinase 11B										
6925	TCF4	transcription factor 4										
9908	G3BP2	GTPase activating protein (SH3 domain) binding protein 2										
3419	IDH3A	isocitrate dehydrogenase 3 (NAD+) alpha										
2184	FAH	fumarylacetoacetate hydrolase (fumarylacetoacetase)										
128338	DRAM2	DNA-damage regulated autophagy modulator 2										
23788	MTCH2	mitochondrial carrier 2										
64225	ATL2	atlastin GTPase 2										
84173	ELMOD3	ELMO/CED-12 domain containing 3										
7181	NR2C1	nuclear receptor subfamily 2, group C, member 1										
23409	SIRT4	sirtuin 4										
4776	NFATC4	nuclear factor of activated T-cells, cytoplasmic, calcineurin-dependent 4										
4775	NFATC3	nuclear factor of activated T-cells, cytoplasmic, calcineurin-dependent 3										
8550	MAPKAPK5	mitogen-activated protein kinase-activated protein kinase 5										
5871	MAP4K2	mitogen-activated protein kinase kinase kinase kinase 2										
116985	ARAP1	ArfGAP with RhoGAP domain, ankyrin repeat and PH domain 1										
9101	USP8	ubiquitin specific peptidase 8										
116986	AGAP2	ArfGAP with GTPase domain, ankyrin repeat and PH domain 2										
116987	AGAP1	ArfGAP with GTPase domain, ankyrin repeat and PH domain 1										
22905	EPN2	epsin 2										
64744	SMAP2	small ArfGAP2										
8658	TNKS	tankyrase, TRF1-interacting ankyrin-related ADP-ribose polymerase										
3420	IDH3B	isocitrate dehydrogenase 3 (NAD+) beta										
10961	ERP29	endoplasmic reticulum protein 29										
84263	HSDL2	hydroxysteroid dehydrogenase like 2										
7915	ALDH5A1	aldehyde dehydrogenase 5 family, member A1										
116285	ACSM1	acyl-CoA synthetase medium-chain family member 1										
2782	GNB1	guanine nucleotide binding protein (G protein), beta polypeptide 1										
60490	PPCDC	phosphopantothenoylcysteine decarboxylase										
79717	PPCS	phosphopantothenoylcysteine synthetase										
9963	SLC23A1	solute carrier family 23 (nucleobase transporters), member 1										
65220	NADK	NAD kinase										
80308	FLAD1	FAD1 flavin adenine dinucleotide synthetase homolog (S. cerevisiae)										
94241	TP53INP1	tumor protein p53 inducible nuclear protein 1										
2319	FLOT2	flotillin 2										
961	CD47	CD47 molecule										
29904	EEF2K	eukaryotic elongation factor-2 kinase										
728642	CDK11A	cyclin-dependent kinase 11A										
3150	HMGN1	high mobility group nucleosome binding domain 1										
10818	FRS2	fibroblast growth factor receptor substrate 2										
10019	SH2B3	SH2B adaptor protein 3										
4597	MVD	mevalonate (diphospho) decarboxylase										
9619	ABCG1	ATP-binding cassette, sub-family G (WHITE), member 1										
134429	STARD4	StAR-related lipid transfer (START) domain containing 4										
1120	CHKB	choline kinase beta										
81490	PTDSS2	phosphatidylserine synthase 2										
55300	PI4K2B	phosphatidylinositol 4-kinase type 2 beta										
55627	SMPD4	sphingomyelin phosphodiesterase 4, neutral membrane (neutral sphingomyelinase-3)										
57176	VARS2	valyl-tRNA synthetase 2, mitochondrial (putative)										
57038	RARS2	arginyl-tRNA synthetase 2, mitochondrial										
57505	AARS2	alanyl-tRNA synthetase 2, mitochondrial (putative)										
118672	PSTK	phosphoseryl-tRNA kinase										
22985	ACIN1	apoptotic chromatin condensation inducer 1										
29896	TRA2A	transformer 2 alpha homolog (Drosophila)										
7919	DDX39B	DEAD (Asp-Glu-Ala-Asp) box polypeptide 39B										
7360	UGP2	UDP-glucose pyrophosphorylase 2										
2582	GALE	UDP-galactose-4-epimerase										
2585	GALK2	galactokinase 2										
114049	WBSCR22	Williams Beuren syndrome chromosome region 22										
51409	HEMK1	HemK methyltransferase family member 1										
60487	TRMT11	tRNA methyltransferase 11 homolog (S. cerevisiae)										
2686	GGT7	gamma-glutamyltransferase 7										
80146	UXS1	UDP-glucuronate decarboxylase 1										
80896	NPL	N-acetylneuraminate pyruvate lyase (dihydrodipicolinate synthase)										
4637	MYL6	myosin, light chain 6, alkali, smooth muscle and non-muscle										
5364	PLXNB1	plexin B1										
64218	SEMA4A	sema domain, immunoglobulin domain (Ig), transmembrane domain (TM) and short cytoplasmic domain, (semaphorin) 4A										
5261	PHKG2	phosphorylase kinase, gamma 2 (testis)										
5257	PHKB	phosphorylase kinase, beta										
55454	CSGALNACT2	chondroitin sulfate N-acetylgalactosaminyltransferase 2										
8940	TOP3B	topoisomerase (DNA) III beta										
6452	SH3BP2	SH3-domain binding protein 2										
2030	SLC29A1	solute carrier family 29 (nucleoside transporters), member 1										
9197	SLC33A1	solute carrier family 33 (acetyl-CoA transporter), member 1										
6560	SLC12A4	solute carrier family 12 (potassium/chloride transporters), member 4										
121260	SLC15A4	solute carrier family 15, member 4										
23315	SLC9A8	solute carrier family 9 (sodium/hydrogen exchanger), member 8										
9497	SLC4A7	solute carrier family 4, sodium bicarbonate cotransporter, member 7										
55244	SLC47A1	solute carrier family 47, member 1										
10396	ATP8A1	ATPase, aminophospholipid transporter (APLT), class I, type 8A, member 1										
286410	ATP11C	ATPase, class VI, type 11C										
374868	ATP9B	ATPase, class II, type 9B										
3145	HMBS	hydroxymethylbilane synthase										
23112	TNRC6B	trinucleotide repeat containing 6B										
2145	EZH1	enhancer of zeste homolog 1 (Drosophila)										
4779	NFE2L1	nuclear factor (erythroid-derived 2)-like 1										
55589	BMP2K	BMP2 inducible kinase										
6397	SEC14L1	SEC14-like 1 (S. cerevisiae)										
80148	PQLC1	PQ loop repeat containing 1										
6472	SHMT2	serine hydroxymethyltransferase 2 (mitochondrial)										
871	SERPINH1	serpin peptidase inhibitor, clade H (heat shock protein 47), member 1, (collagen binding protein 1)										
1606	DGKA	diacylglycerol kinase, alpha 80kDa										
23303	KIF13B	kinesin family member 13B										
63874	ABHD4	abhydrolase domain containing 4										
79651	RHBDF2	rhomboid 5 homolog 2 (Drosophila)										
23324	MAN2B2	mannosidase, alpha, class 2B, member 2										
8681	JMJD7-PLA2G4B	JMJD7-PLA2G4B readthrough										
2976	GTF3C2	general transcription factor IIIC, polypeptide 2, beta 110kDa										
2339	FNTA	farnesyltransferase, CAAX box, alpha										
8527	DGKD	diacylglycerol kinase, delta 130kDa										
27131	SNX5	sorting nexin 5										
57534	MIB1	mindbomb homolog 1 (Drosophila)										
142678	MIB2	mindbomb homolog 2 (Drosophila)										
4343	MOV10	Mov10, Moloney leukemia virus 10, homolog (mouse)										
7391	USF1	upstream transcription factor 1										
145957	NRG4	neuregulin 4										
22797	TFEC	transcription factor EC										
83478	ARHGAP24	Rho GTPase activating protein 24										
84144	SYDE2	synapse defective 1, Rho GTPase, homolog 2 (C. elegans)										
93663	ARHGAP18	Rho GTPase activating protein 18										
7388	UQCRH	ubiquinol-cytochrome c reductase hinge protein										
4708	NDUFB2	NADH dehydrogenase (ubiquinone) 1 beta subcomplex, 2, 8kDa										
9617	MTRF1	mitochondrial translational release factor 1										
11052	CPSF6	cleavage and polyadenylation specific factor 6, 68kDa										
57591	MKL1	megakaryoblastic leukemia (translocation) 1										
83605	CCM2	cerebral cavernous malformation 2										
9830	TRIM14	tripartite motif containing 14										
85363	TRIM5	tripartite motif containing 5										
6311	ATXN2	ataxin 2										
4301	MLLT4	myeloid/lymphoid or mixed-lineage leukemia (trithorax homolog, Drosophila); translocated to, 4										
5828	PEX2	peroxisomal biogenesis factor 2										
3416	IDE	insulin-degrading enzyme										
60386	SLC25A19	solute carrier family 25 (mitochondrial thiamine pyrophosphate carrier), member 19										
221178	SPATA13	spermatogenesis associated 13										
5191	PEX7	peroxisomal biogenesis factor 7										
4358	MPV17	MpV17 mitochondrial inner membrane protein										
122809	SOCS4	suppressor of cytokine signaling 4										
23036	ZNF292	zinc finger protein 292										
55139	ANKZF1	ankyrin repeat and zinc finger domain containing 1										
2744	GLS	glutaminase										
56267	CCBL2	cysteine conjugate-beta lyase 2										
8573	CASK	calcium/calmodulin-dependent serine protein kinase (MAGUK family)										
84458	LCOR	ligand dependent nuclear receptor corepressor										
5150	PDE7A	phosphodiesterase 7A										
10325	RRAGB	Ras-related GTP binding B										
7169	TPM2	tropomyosin 2 (beta)										

165	AEBP1	AE binding protein 1										
2314	FLII	flightless I homolog (Drosophila)										
8498	RANBP3	RAN binding protein 3										
6935	ZEB1	zinc finger E-box binding homeobox 1										
80895	ILKAP	integrin-linked kinase-associated serine/threonine phosphatase										
9046	DOK2	docking protein 2, 56kDa										
5023	P2RX1	purinergic receptor P2X, ligand-gated ion channel, 1										
7804	LRP8	low density lipoprotein receptor-related protein 8, apolipoprotein e receptor										
221037	JMJD1C	jumonji domain containing 1C										
8100	IFT88	intraflagellar transport 88 homolog (Chlamydomonas)										
11238	CA5B	carbonic anhydrase VB, mitochondrial										
59339	PLEKHA2	pleckstrin homology domain containing, family A (phosphoinositide binding specific) member 2										
25989	ULK3	unc-51-like kinase 3 (C. elegans)										
161742	SPRED1	sprouty-related, EVH1 domain containing 1										
10627	MYL12A	myosin, light chain 12A, regulatory, non-sarcomeric										
240	ALOX5	arachidonate 5-lipoxygenase										
255631	COL24A1	collagen, type XXIV, alpha 1										
3783	KCNN4	potassium intermediate/small conductance calcium-activated channel, subfamily N, member 4										
131096	KCNH8	potassium voltage-gated channel, subfamily H (eag-related), member 8										
11315	PARK7	parkinson protein 7										
23623	RUSC1	RUN and SH3 domain containing 1										
6601	SMARCC2	SWI/SNF related, matrix associated, actin dependent regulator of chromatin, subfamily c, member 2										
7511	XPNPEP1	X-prolyl aminopeptidase (aminopeptidase P) 1, soluble										
4839	NOP2	NOP2 nucleolar protein homolog (yeast)										
22807	IKZF2	IKAROS family zinc finger 2 (Helios)										
10237	SLC35B1	solute carrier family 35, member B1										
200186	CRTC2	CREB regulated transcription coactivator 2										
951	CD37	CD37 molecule										
931	MS4A1	membrane-spanning 4-domains, subfamily A, member 1										
56938	ARNTL2	aryl hydrocarbon receptor nuclear translocator-like 2										
81887	LAS1L	LAS1-like (S. cerevisiae)										
203522	DDX26B	DEAD/H (Asp-Glu-Ala-Asp/His) box polypeptide 26B										
220001	VWCE	von Willebrand factor C and EGF domains										
25992	SNED1	sushi, nidogen and EGF-like domains 1										
78987	CRELD1	cysteine-rich with EGF-like domains 1										
29121	CLEC2D	C-type lectin domain family 2, member D										
3965	LGALS9	lectin, galactoside-binding, soluble, 9										
83544	DNAL1	dynein, axonemal, light chain 1										
9094	UNC119	unc-119 homolog (C. elegans)										
4481	MSR1	macrophage scavenger receptor 1										
1730	DIAPH2	diaphanous homolog 2 (Drosophila)										
10863	ADAM28	ADAM metallopeptidase domain 28										
79676	OGFOD2	2-oxoglutarate and iron-dependent oxygenase domain containing 2										
1838	DTNB	dystrobrevin, beta										
8115	TCL1A	T-cell leukemia/lymphoma 1A										
1955	MEGF9	multiple EGF-like-domains 9										
84957	RELT	RELT tumor necrosis factor receptor										
5876	RABGGTB	Rab geranylgeranyltransferase, beta subunit										
340481	ZDHHC21	zinc finger, DHHC-type containing 21										
11284	PNKP	polynucleotide kinase 3'-phosphatase										
100130967	C6orf99	chromosome 6 open reading frame 99										
10045	SH2D3A	SH2 domain containing 3A										
10114	HIPK3	homeodomain interacting protein kinase 3										
10198	MPHOSPH9	M-phase phosphoprotein 9										
10233	LRRC23	leucine rich repeat containing 23										
10346	TRIM22	tripartite motif containing 22										
10385	BTN2A2	butyrophilin, subfamily 2, member A2										
10443	N4BP2L2	NEDD4 binding protein 2-like 2										
10447	FAM3C	family with sequence similarity 3, member C										
10480	EIF3M	eukaryotic translation initiation factor 3, subunit M										
10613	ERLIN1	ER lipid raft associated 1										
10625	IVNS1ABP	influenza virus NS1A binding protein										
10636	RGS14	regulator of G-protein signaling 14										
10641	NPRL2	nitrogen permease regulator-like 2 (S. cerevisiae)										
10650	SLMO1	slowmo homolog 1 (Drosophila)										
10765	KDM5B	lysine (K)-specific demethylase 5B										
10785	WDR4	WD repeat domain 4										
10807	SDCCAG3	serologically defined colon cancer antigen 3										
10922	FASTK	Fas-activated serine/threonine kinase										
10980	COPS6	COP9 constitutive photomorphogenic homolog subunit 6 (Arabidopsis)										
1102	RCBTB2	regulator of chromosome condensation (RCC1) and BTB (POZ) domain containing protein 2										
11020	IFT27	intraflagellar transport 27 homolog (Chlamydomonas)										
1106	CHD2	chromodomain helicase DNA binding protein 2										
11062	DUS4L	dihydrouridine synthase 4-like (S. cerevisiae)										
11101	ATE1	arginyltransferase 1										
11212	PROSC	proline synthetase co-transcribed homolog (bacterial)										
11257	TP53TG1	TP53 target 1 (non-protein coding)										
113263	GLCCI1	glucocorticoid induced transcript 1										
113402	SFT2D1	SFT2 domain containing 1										
114659	LRRC37B	leucine rich repeat containing 37B										
114803	MYSM1	Myb-like, SWIRM and MPN domains 1										
115106	HAUS1	HAUS augmin-like complex, subunit 1										
115426	UHRF2	ubiquitin-like with PHD and ring finger domains 2										
116447	TOP1MT	topoisomerase (DNA) I, mitochondrial										
116988	AGAP3	ArfGAP with GTPase domain, ankyrin repeat and PH domain 3										
118433	RPL23AP7	ribosomal protein L23a pseudogene 7										
119504	ANAPC16	anaphase promoting complex subunit 16										
120103	SLC36A4	solute carrier family 36 (proton/amino acid symporter), member 4										
122704	MRPL52	mitochondrial ribosomal protein L52										
123355	LRRC28	leucine rich repeat containing 28										
124512	METTL23	methyltransferase like 23										
124923	SGK494	uncharacterized serine/threonine-protein kinase SgK494										
124930	ANKRD13B	ankyrin repeat domain 13B										
125476	INO80C	INO80 complex subunit C										
126119	JOSD2	Josephin domain containing 2										
126282	TNFAIP8L1	tumor necrosis factor, alpha-induced protein 8-like 1										
126789	PUSL1	pseudouridylate synthase-like 1										
129138	ANKRD54	ankyrin repeat domain 54										
129531	MITD1	MIT, microtubule interacting and transport, domain containing 1										
132160	PPM1M	protein phosphatase, Mg2+/Mn2+ dependent, 1M										
133015	PACRGL	PARK2 co-regulated-like										
134492	NUDCD2	NudC domain containing 2										
143570	XRRA1	X-ray radiation resistance associated 1										
143684	FAM76B	family with sequence similarity 76, member B										
144348	ZNF664	zinc finger protein 664										
144453	BEST3	bestrophin 3										
145773	FAM81A	family with sequence similarity 81, member A										
146206	RLTPR	RGD motif, leucine rich repeats, tropomodulin domain and proline-rich containing										
147138	TMC8	transmembrane channel-like 8										
147991	DPY19L3	dpy-19-like 3 (C. elegans)										
148423	C1orf52	chromosome 1 open reading frame 52										
149175	MANEAL	mannosidase, endo-alpha-like										
149986	LSM14B	LSM14B, SCD6 homolog B (S. cerevisiae)										
150223	YDJC	YdjC homolog (bacterial)										
150709	ANKAR	ankyrin and armadillo repeat containing										
150771	ITPRIPL1	inositol 1,4,5-trisphosphate receptor interacting protein-like 1										
151613	TTC14	tetratricopeptide repeat domain 14										
157680	VPS13B	vacuolar protein sorting 13 homolog B (yeast)										
158431	ZNF782	zinc finger protein 782										
159195	USP54	ubiquitin specific peptidase 54										
160140	C11orf65	chromosome 11 open reading frame 65										
160335	TMTC2	transmembrane and tetratricopeptide repeat containing 2										
165055	CCDC138	coiled-coil domain containing 138										
166824	RASSF6	Ras association (RalGDS/AF-6) domain family member 6										
167153	PAPD4	PAP associated domain containing 4										
168451	THAP5	THAP domain containing 5										
169200	TMEM64	transmembrane protein 64										
170371	C10orf128	chromosome 10 open reading frame 128										
1768	DNAH6	dynein, axonemal, heavy chain 6										
196074	METTL15	methyltransferase like 15										
199786	FAM129C	family with sequence similarity 129, member C										
200014	CC2D1B	coiled-coil and C2 domain containing 1B										
200030	NBPF11	neuroblastoma breakpoint family, member 11										
200424	TET3	tet methylcytosine dioxygenase 3										
201254	STRA13	stimulated by retinoic acid 13 homolog (mouse)										
201283	AMZ2P1	archaelysin family metallopeptidase 2 pseudogene 1										
201299	RDM1	RAD52 motif 1										
202018	TAPT1	transmembrane anterior posterior transformation 1										
203054	ADCK5	aarF domain containing kinase 5										
203228	C9orf72	chromosome 9 open reading frame 72										
2140	EYA3	eyes absent homolog 3 (Drosophila)										
219899	TBCEL	tubulin folding cofactor E-like										
219931	TPCN2	two pore segment channel 2										
221016	CCDC7	coiled-coil domain containing 7										
222234	FAM185A	family with sequence similarity 185, member A										
222236	NAPEPLD	N-acyl phosphatidylethanolamine phospholipase D										
22835	ZFP30	zinc finger protein 30 homolog (mouse)										
22838	RNF44	ring finger protein 44										
22858	ICK	intestinal cell (MAK-like) kinase										
22890	ZBTB1	zinc finger and BTB domain containing 1										
22980	TCF25	transcription factor 25 (basic helix-loop-helix)										
22990	PCNX	pecanex homolog (Drosophila)										
23011	RAB21	RAB21, member RAS oncogene family										
23023	TMCC1	transmembrane and coiled-coil domain family 1										
23032	USP33	ubiquitin specific peptidase 33										
23035	PHLPP2	PH domain and leucine rich repeat protein phosphatase 2										
23064	SETX	senataxin										
23077	MYCBP2	MYC binding protein 2										
23093	TTLL5	tubulin tyrosine ligase-like family, member 5										
23113	CUL9	cullin 9										
23132	RAD54L2	RAD54-like 2 (S. cerevisiae)										
23155	CLCC1	chloride channel CLIC-like 1										
23158	TBC1D9	TBC1 domain family, member 9 (with GRAM domain)										
23179	RGL1	ral guanine nucleotide dissociation stimulator-like 1										
23189	KANK1	KN motif and ankyrin repeat domains 1										
23195	MDN1	MDN1, midasin homolog (yeast)										
23215	PRRC2C	proline-rich coiled-coil 2C										
23232	TBC1D12	TBC1 domain family, member 12										
23244	PDS5A	PDS5, regulator of cohesion maintenance, homolog A (S. cerevisiae)										
23247	KIAA0556	KIAA0556										
23252	OTUD3	OTU domain containing 3										
23262	PPIP5K2	diphosphoinositol pentakisphosphate kinase 2										
23269	MGA	MAX gene associated										
23307	FKBP15	FK506 binding protein 15, 133kDa										
23316	CUX2	cut-like homeobox 2										
23339	VPS39	vacuolar protein sorting 39 homolog (S. cerevisiae)										
23353	SUN1	Sad1 and UNC84 domain containing 1										
23367	LARP1	La ribonucleoprotein domain family, member 1										
23420	NOMO1	NODAL modulator 1										
23505	TMEM131	transmembrane protein 131										
23534	TNPO3	transportin 3										
23559	WBP1	WW domain binding protein 1										
23603	CORO1C	coronin, actin binding protein, 1C										
23616	SH3BP1	SH3-domain binding protein 1										
23642	SNHG1	small nucleolar RNA host gene 1 (non-protein coding)										
24139	EML2	echinoderm microtubule associated protein like 2										
24144	TFIP11	tuftelin interacting protein 11										
253769	WDR27	WD repeat domain 27										
253832	ZDHHC20	zinc finger, DHHC-type containing 20										
2551	GABPA	GA binding protein transcription factor, alpha subunit 60kDa										
255231	MCOLN2	mucolipin 2										
255252	LRRC57	leucine rich repeat containing 57										
257218	SHPRH	SNF2 histone linker PHD RING helicase										
25807	RHBDD3	rhomboid domain containing 3										
25809	TTLL1	tubulin tyrosine ligase-like family, member 1										
25831	HECTD1	HECT domain containing 1										
25875	LETMD1	LETM1 domain containing 1										
25917	THUMPD3	THUMP domain containing 3										
25926	NOL11	nucleolar protein 11										
259266	ASPM	asp (abnormal spindle) homolog, microcephaly associated (Drosophila)										
25956	SEC31B	SEC31 homolog B (S. cerevisiae)										
26058	GIGYF2	GRB10 interacting GYF protein 2										
26147	PHF19	PHD finger protein 19										
26151	NAT9	N-acetyltransferase 9 (GCN5-related, putative)										
26166	RGS22	regulator of G-protein signaling 22										
26260	FBXO25	F-box protein 25										
26262	TSPAN17	tetraspanin 17										
26284	ERAL1	Era G-protein-like 1 (E. coli)										
2630	GBAP1	glucosidase, beta, acid pseudogene 1										
26751	SH3YL1	SH3 domain containing, Ysc84-like 1 (S. cerevisiae)										
26993	AKAP8L	A kinase (PRKA) anchor protein 8-like										
27004	TCL6	T-cell leukemia/lymphoma 6 (non-protein coding)										
27005	USP21	ubiquitin specific peptidase 21										
27037	TRMT2A	TRM2 tRNA methyltransferase 2 homolog A (S. cerevisiae)										
27163	NAAA	N-acylethanolamine acid amidase										
27315	PGAP2	post-GPI attachment to proteins 2										
27352	SGSM3	small G protein signaling modulator 3										
2794	GNL1	guanine nucleotide binding protein-like 1										
282809	POC1B	POC1 centriolar protein homolog B (Chlamydomonas)										
284403	WDR62	WD repeat domain 62										
28511	NKIRAS2	NFKB inhibitor interacting Ras-like 2										
285550	FAM200B	family with sequence similarity 200, member B										
286257	C9orf142	chromosome 9 open reading frame 142										
28638	TRBC2	T cell receptor beta constant 2										
287	ANK2	ankyrin 2, neuronal										
28970	C11orf54	chromosome 11 open reading frame 54										
28977	MRPL42	mitochondrial ribosomal protein L42										
28990	ASTE1	asteroid homolog 1 (Drosophila)										
29097	CNIH4	cornichon homolog 4 (Drosophila)										
29104	N6AMT1	N-6 adenine-specific DNA methyltransferase 1 (putative)										
29109	FHOD1	formin homology 2 domain containing 1										
29116	MYLIP	myosin regulatory light chain interacting protein										
29761	USP25	ubiquitin specific peptidase 25										
29803	REPIN1	replication initiator 1										
29960	FTSJ2	FtsJ homolog 2 (E. coli)										
29966	STRN3	striatin, calmodulin binding protein 3										
29994	BAZ2B	bromodomain adjacent to zinc finger domain, 2B										

317662	FAM149B1	family with sequence similarity 149, member B1										
338440	ANO9	anoctamin 9										
338657	CCDC84	coiled-coil domain containing 84										
339123	JMJD8	jumonji domain containing 8										
340252	ZNF680	zinc finger protein 680										
3428	IFI16	interferon, gamma-inducible protein 16										
346389	MACC1	metastasis associated in colon cancer 1										
3476	IGBP1	immunoglobulin (CD79A) binding protein 1										
359948	IRF2BP2	interferon regulatory factor 2 binding protein 2										
374395	TMEM179B	transmembrane protein 179B										
374666	WASH3P	WAS protein family homolog 3 pseudogene										
374986	FAM73A	family with sequence similarity 73, member A										
375248	ANKRD36	ankyrin repeat domain 36										
388561	ZNF761	zinc finger protein 761										
389362	PSMG4	proteasome (prosome, macropain) assembly chaperone 4										
3899	AFF3	AF4/FMR2 family, member 3										
391059	FRRS1	ferric-chelate reductase 1										
399818	METTL10	methyltransferase like 10										
400322	HERC2P2	hect domain and RLD 2 pseudogene 2										
400798	C1orf220	chromosome 1 open reading frame 220										
400954	EML6	echinoderm microtubule associated protein like 6										
414332	LCN10	lipocalin 10										
414777	HCG18	HLA complex group 18 (non-protein coding)										
4152	MBD1	methyl-CpG binding domain protein 1										
4154	MBNL1	muscleblind-like (Drosophila)										
440675	NBPF12	neuroblastoma breakpoint family, member 12										
441194	PMS2CL	PMS2 C-terminal like pseudogene										
4649	MYO9A	myosin IXA										
474170	LRRC37A2	leucine rich repeat containing 37, member A2										
4798	NFRKB	nuclear factor related to kappaB binding protein										
493856	CISD2	CDGSH iron sulfur domain 2										
497661	C18orf32	chromosome 18 open reading frame 32										
50717	DCAF8	DDB1 and CUL4 associated factor 8										
51011	FAHD2A	fumarylacetoacetate hydrolase domain containing 2A										
51023	MRPS18C	mitochondrial ribosomal protein S18C										
51043	ZBTB7B	zinc finger and BTB domain containing 7B										
51093	RRNAD1	ribosomal RNA adenine dimethylase domain containing 1										
5110	PCMT1	protein-L-isoaspartate (D-aspartate) O-methyltransferase										
51185	CRBN	cereblon										
51231	VRK3	vaccinia related kinase 3										
51237	MZB1	marginal zone B and B1 cell-specific protein										
51256	TBC1D7	TBC1 domain family, member 7										
51271	UBAP1	ubiquitin associated protein 1										
51299	NRN1	neuritin 1										
51308	REEP2	receptor accessory protein 2										
51319	RSRC1	arginine/serine-rich coiled-coil 1										
51427	ZNF107	zinc finger protein 107										
51523	CXXC5	CXXC finger protein 5										
51571	FAM49B	family with sequence similarity 49, member B										
51574	LARP7	La ribonucleoprotein domain family, member 7										
51631	LUC7L2	LUC7-like 2 (S. cerevisiae)										
51654	CDK5RAP1	CDK5 regulatory subunit associated protein 1										
5378	PMS1	PMS1 postmeiotic segregation increased 1 (S. cerevisiae)										
53904	MYO3A	myosin IIIA										
53917	RAB24	RAB24, member RAS oncogene family										
54058	C21orf58	chromosome 21 open reading frame 58										
54432	YIPF1	Yip1 domain family, member 1										
54455	FBXO42	F-box protein 42										
54467	ANKIB1	ankyrin repeat and IBR domain containing 1										
54469	ZFAND6	zinc finger, AN1-type domain 6										
54476	RNF216	ring finger protein 216										
54496	PRMT7	protein arginine methyltransferase 7										
54517	PUS7	pseudouridylate synthase 7 homolog (S. cerevisiae)										
54540	FAM193B	family with sequence similarity 193, member B										
54585	LZTFL1	leucine zipper transcription factor-like 1										
54619	CCNJ	cyclin J										
54758	KLHDC4	kelch domain containing 4										
54790	TET2	tet methylcytosine dioxygenase 2										
54793	KCTD9	potassium channel tetramerisation domain containing 9										
54811	ZNF562	zinc finger protein 562										
54828	BCAS3	breast carcinoma amplified sequence 3										
54834	GDAP2	ganglioside induced differentiation associated protein 2										
54851	ANKRD49	ankyrin repeat domain 49										
54878	DPP8	dipeptidyl-peptidase 8										
54883	CWC25	CWC25 spliceosome-associated protein homolog (S. cerevisiae)										
54892	NCAPG2	non-SMC condensin II complex, subunit G2										
54899	PXK	PX domain containing serine/threonine kinase										
54900	LAX1	lymphocyte transmembrane adaptor 1										
54923	LIME1	Lck interacting transmembrane adaptor 1										
54930	HAUS4	HAUS augmin-like complex, subunit 4										
54939	COMMD4	COMM domain containing 4										
54952	TRNAU1AP	tRNA selenocysteine 1 associated protein 1										
54953	C1orf27	chromosome 1 open reading frame 27										
54973	CPSF3L	cleavage and polyadenylation specific factor 3-like										
55015	PRPF39	PRP39 pre-mRNA processing factor 39 homolog (S. cerevisiae)										
55041	PLEKHB2	pleckstrin homology domain containing, family B (evectins) member 2										
55080	TAPBPL	TAP binding protein-like										
55102	ATG2B	ATG2 autophagy related 2 homolog B (S. cerevisiae)										
55132	LARP1B	La ribonucleoprotein domain family, member 1B										
55133	SRBD1	S1 RNA binding domain 1										
55144	LRRC8D	leucine rich repeat containing 8 family, member D										
55161	TMEM33	transmembrane protein 33										
55173	MRPS10	mitochondrial ribosomal protein S10										
55180	LINS	lines homolog (Drosophila)										
55183	RIF1	RAP1 interacting factor homolog (yeast)										
55186	SLC25A36	solute carrier family 25, member 36										
55209	SETD5	SET domain containing 5										
55251	PCMTD2	protein-L-isoaspartate (D-aspartate) O-methyltransferase domain containing 2										
55253	TYW1	tRNA-yW synthesizing protein 1 homolog (S. cerevisiae)										
55322	C5orf22	chromosome 5 open reading frame 22										
55327	LIN7C	lin-7 homolog C (C. elegans)										
554203	JPX	JPX transcript, XIST activator (non-protein coding)										
55536	CDCA7L	cell division cycle associated 7-like										
55582	KIF27	kinesin family member 27										
55596	ZCCHC8	zinc finger, CCHC domain containing 8										
55614	KIF16B	kinesin family member 16B										
55625	ZDHHC7	zinc finger, DHHC-type containing 7										
55665	URGCP	upregulator of cell proliferation										
55671	SMEK1	SMEK homolog 1, suppressor of mek1 (Dictyostelium)										
55689	YEATS2	YEATS domain containing 2										
55728	N4BP2	NEDD4 binding protein 2										
55732	C1orf112	chromosome 1 open reading frame 112										
55735	DNAJC11	DnaJ (Hsp40) homolog, subfamily C, member 11										
55764	IFT122	intraflagellar transport 122 homolog (Chlamydomonas)										
55777	MBD5	methyl-CpG binding domain protein 5										
55846	ITFG2	integrin alpha FG-GAP repeat containing 2										
55858	TMEM165	transmembrane protein 165										
55871	CBWD1	COBW domain containing 1										
55885	LMO3	LIM domain only 3 (rhombotin-like 2)										
55974	SLC50A1	solute carrier family 50 (sugar transporter), member 1										
5612	PRKRIR	protein-kinase, interferon-inducible double stranded RNA dependent inhibitor, repressor of (P58 repressor)										
56265	CPXM1	carboxypeptidase X (M14 family), member 1										
56681	SAR1A	SAR1 homolog A (S. cerevisiae)										
56850	GRIPAP1	GRIP1 associated protein 1										
56943	ENY2	enhancer of yellow 2 homolog (Drosophila)										
56947	MFF	mitochondrial fission factor										
56965	PARP6	poly (ADP-ribose) polymerase family, member 6										
56986	DTWD1	DTW domain containing 1										
57037	ANKMY2	ankyrin repeat and MYND domain containing 2										
57143	ADCK1	aarF domain containing kinase 1										
57149	LYRM1	LYR motif containing 1										
57179	KIAA1191	KIAA1191										
57180	ACTR3B	ARP3 actin-related protein 3 homolog B (yeast)										
57380	MRS2	MRS2 magnesium homeostasis factor homolog (S. cerevisiae)										
57410	SCYL1	SCY1-like 1 (S. cerevisiae)										
57473	ZNF512B	zinc finger protein 512B										
57480	PLEKHG1	pleckstrin homology domain containing, family G (with RhoGef domain) member 1										
57488	ESYT2	extended synaptotagmin-like protein 2										
57507	ZNF608	zinc finger protein 608										
57519	STARD9	StAR-related lipid transfer (START) domain containing 9										
57531	HACE1	HECT domain and ankyrin repeat containing, E3 ubiquitin protein ligase 1										
57533	TBC1D14	TBC1 domain family, member 14										
57560	IFT80	intraflagellar transport 80 homolog (Chlamydomonas)										
57727	NCOA5	nuclear receptor coactivator 5										
57730	ANKRD36B	ankyrin repeat domain 36B										
5775	PTPN4	protein tyrosine phosphatase, non-receptor type 4 (megakaryocyte)										
583	BBS2	Bardet-Biedl syndrome 2										
58516	FAM60A	family with sequence similarity 60, member A										
5863	RGL2	ral guanine nucleotide dissociation stimulator-like 2										
59286	UBL5	ubiquitin-like 5										
60343	FAM3A	family with sequence similarity 3, member A										
60492	CCDC90B	coiled-coil domain containing 90B										
6100	RP9	retinitis pigmentosa 9 (autosomal dominant)										
6293	VPS52	vacuolar protein sorting 52 homolog (S. cerevisiae)										
6328	SCN3A	sodium channel, voltage-gated, type III, alpha subunit										
641638	SNHG6	small nucleolar RNA host gene 6 (non-protein coding)										
64210	MMS19	MMS19 nucleotide excision repair homolog (S. cerevisiae)										
643836	ZFP62	zinc finger protein 62 homolog (mouse)										
64427	TTC31	tetratricopeptide repeat domain 31										
645784	ANKRD36BP2	ankyrin repeat domain 36B pseudogene 2										
645811	CCDC154	coiled-coil domain containing 154										
64598	MOSPD3	motile sperm domain containing 3										
64718	UNKL	unkempt homolog (Drosophila)-like										
64754	SMYD3	SET and MYND domain containing 3										
64770	CCDC14	coiled-coil domain containing 14										
64776	C11orf1	chromosome 11 open reading frame 1										
64780	MICAL1	microtubule associated monoxygenase, calponin and LIM domain containing 1										
64921	CASD1	CAS1 domain containing 1										
65056	GPBP1	GC-rich promoter binding protein 1										
65095	KRI1	KRI1 homolog (S. cerevisiae)										
65117	RSRC2	arginine/serine-rich coiled-coil 2										
65258	MPPE1	metallophosphoesterase 1										
65981	CAPRIN2	caprin family member 2										
66036	MTMR9	myotubularin related protein 9										
6894	TARBP1	TAR (HIV-1) RNA binding protein 1										
6919	TCEA2	transcription elongation factor A (SII), 2										
7072	TIA1	TIA1 cytotoxic granule-associated RNA binding protein										
7267	TTC3	tetratricopeptide repeat domain 3										
728568	C12orf73	chromosome 12 open reading frame 73										
730051	ZNF814	zinc finger protein 814										
7444	VRK2	vaccinia related kinase 2										
7553	ZNF7	zinc finger protein 7										
7579	ZSCAN20	zinc finger and SCAN domain containing 20										
7629	ZNF76	zinc finger protein 76										
7748	ZNF195	zinc finger protein 195										
7775	ZNF232	zinc finger protein 232										
78996	C7orf49	chromosome 7 open reading frame 49										
79009	DDX50	DEAD (Asp-Glu-Ala-Asp) box polypeptide 50										
79065	ATG9A	ATG9 autophagy related 9 homolog A (S. cerevisiae)										
79072	FASTKD3	FAST kinase domains 3										
79101	TAF1D	TATA box binding protein (TBP)-associated factor, RNA polymerase I, D, 41kDa										
79140	CCDC28B	coiled-coil domain containing 28B										
79145	CHCHD7	coiled-coil-helix-coiled-coil-helix domain containing 7										
79149	ZSCAN5A	zinc finger and SCAN domain containing 5A										
79180	EFHD2	EF-hand domain family, member D2										
79568	C2orf47	chromosome 2 open reading frame 47										
79618	HMBOX1	homeobox containing 1										
79726	WDR59	WD repeat domain 59										
79745	CLIP4	CAP-GLY domain containing linker protein family, member 4										
79752	ZFAND1	zinc finger, AN1-type domain 1										
79805	VASH2	vasohibin 2										
79867	TCTN2	tectonic family member 2										
79893	GGNBP2	gametogenetin binding protein 2										
79930	DOK3	docking protein 3										
79932	KIAA0319L	KIAA0319-like										
79956	ERMP1	endoplasmic reticulum metallopeptidase 1										
79969	ATAT1	alpha tubulin acetyltransferase 1										
79990	PLEKHH3	pleckstrin homology domain containing, family H (with MyTH4 domain) member 3										
80011	FAM192A	family with sequence similarity 192, member A										
80013	FAM188A	family with sequence similarity 188, member A										
80017	C14orf159	chromosome 14 open reading frame 159										
80067	DCAF17	DDB1 and CUL4 associated factor 17										
80199	FUZ	fuzzy homolog (Drosophila)										
80205	CHD9	chromodomain helicase DNA binding protein 9										
80279	CDK5RAP3	CDK5 regulatory subunit associated protein 3										
80764	THAP7	THAP domain containing 7										
80817	CEP44	centrosomal protein 44kDa										
80862	ZNRD1-AS1	ZNRD1 antisense RNA 1 (non-protein coding)										
81576	CCDC130	coiled-coil domain containing 130										
81627	TRMT1L	TRM1 tRNA methyltransferase 1-like										
81831	NETO2	neuropilin (NRP) and tolloid (TLL)-like 2										
81890	QTRT1	queuine tRNA-ribosyltransferase 1										
8233	ZRSR2	zinc finger (CCCH type), RNA-binding motif and serine/arginine rich 2										
8284	KDM5D	lysine (K)-specific demethylase 5D										
83416	FCRL5	Fc receptor-like 5										
83440	ADPGK	ADP-dependent glucokinase										
83448	PUS7L	pseudouridylate synthase 7 homolog (S. cerevisiae)-like										
83479	DDX59	DEAD (Asp-Glu-Ala-Asp) box polypeptide 59										
83707	TRPT1	tRNA phosphotransferase 1										
83931	STK40	serine/threonine kinase 40										
84064	HDHD2	haloacid dehalogenase-like hydrolase domain containing 2										
84128	WDR75	WD repeat domain 75										
84129	ACAD11	acyl-CoA dehydrogenase family, member 11										
84140	FAM161A	family with sequence similarity 161, member A										
84236	RHBDD1	rhomboid domain containing 1										
84268	RPAIN	RPA interacting protein										
84280	BTBD10	BTB (POZ) domain containing 10										

84307	ZNF397	zinc finger protein 397										
84318	CCDC77	coiled-coil domain containing 77										
84326	C16orf13	chromosome 16 open reading frame 13										
84503	ZNF527	zinc finger protein 527										
84513	PPAPDC1B	phosphatidic acid phosphatase type 2 domain containing 1B										
84614	ZBTB37	zinc finger and BTB domain containing 37										
84722	PSRC1	proline/serine-rich coiled-coil 1										
84747	UNC119B	unc-119 homolog B (C. elegans)										
84807	NFKBID	nuclear factor of kappa light polypeptide gene enhancer in B-cells inhibitor, delta										
84899	TMTC4	transmembrane and tetratricopeptide repeat containing 4										
84930	MASTL	microtubule associated serine/threonine kinase-like										
84939	MUM1	melanoma associated antigen (mutated) 1										
84941	HSH2D	hematopoietic SH2 domain containing										
85014	TMEM141	transmembrane protein 141										
85028	SNHG12	small nucleolar RNA host gene 12 (non-protein coding)										
85406	DNAJC14	DnaJ (Hsp40) homolog, subfamily C, member 14										
8576	STK16	serine/threonine kinase 16										
85865	GTPBP10	GTP-binding protein 10 (putative)										
8847	DLEU2	deleted in lymphocytic leukemia 2 (non-protein coding)										
8859	STK19	serine/threonine kinase 19										
889	KRIT1	KRIT1, ankyrin repeat containing										
8904	CPNE1	copine I										
89122	TRIM4	tripartite motif containing 4										
89781	HPS4	Hermansky-Pudlak syndrome 4										
89894	TMEM116	transmembrane protein 116										
90133	KRT8P12	keratin 8 pseudogene 12										
9016	SLC25A14	solute carrier family 25 (mitochondrial carrier, brain), member 14										
90416	C15orf57	chromosome 15 open reading frame 57										
90835	C16orf93	chromosome 16 open reading frame 93										
90957	DHX57	DEAH (Asp-Glu-Ala-Asp/His) box polypeptide 57										
9147	NEMF	nuclear export mediator factor										
9158	FIBP	fibroblast growth factor (acidic) intracellular binding protein										
91607	SLFN11	schlafen family member 11										
91833	WDR20	WD repeat domain 20										
92017	SNX29	sorting nexin 29										
9204	ZMYM6	zinc finger, MYM-type 6										
9208	LRRFIP1	leucine rich repeat (in FLII) interacting protein 1										
9209	LRRFIP2	leucine rich repeat (in FLII) interacting protein 2										
92105	INTS4	integrator complex subunit 4										
92399	MRRF	mitochondrial ribosome recycling factor										
92822	ZNF276	zinc finger protein 276										
93627	TBCK	TBC1 domain containing kinase										
94120	SYTL3	synaptotagmin-like 3										
9535	GMFG	glia maturation factor, gamma										
9581	PREPL	prolyl endopeptidase-like										
9584	RBM39	RNA binding motif protein 39										
9604	RNF14	ring finger protein 14										
9643	MORF4L2	mortality factor 4 like 2										
96459	FNIP1	folliculin interacting protein 1										
9657	IQCB1	IQ motif containing B1										
9699	RIMS2	regulating synaptic membrane exocytosis 2										
9701	PPP6R2	protein phosphatase 6, regulatory subunit 2										
9703	KIAA0100	KIAA0100										
9711	KIAA0226	KIAA0226										
9777	TM9SF4	transmembrane 9 superfamily protein member 4										
9782	MATR3	matrin 3										
9786	KIAA0586	KIAA0586										
9841	ZBTB24	zinc finger and BTB domain containing 24										
9859	CEP170	centrosomal protein 170kDa										
9877	ZC3H11A	zinc finger CCCH-type containing 11A										
9898	UBAP2L	ubiquitin associated protein 2-like										
9921	RNF10	ring finger protein 10										

Supplementary Table 10: SUDHL4 Alternative Splicing MsigDB Analysis

Overlap Results	
Collection(s):	CP, CP:BIOCARTA, CP:KEGG, CP:REACTOME, H
# overlaps shown:	10
# genesets in collections:	1379
# genes in comparison (n):	1511
# genes in universe (N):	45956

Gene Set Name	# Genes in Gene Set (K)	Description	# Genes in Overlap (k)	k/K	p-value	FDR q-value
REACTOME_CELL_CYCLE	421	Genes involved in Cell Cycle	54	0.1283	1.14E-25	1.58E-22
REACTOME_IMMUNE_SYSTEM	933	Genes involved in Immune System	101	0.1083	6.78E-22	4.68E-19
REACTOME_ADAPTIVE_IMMUNE_SYSTEM	539	Genes involved in Adaptive Immune System	69	0.128	5.77E-19	2.65E-16
REACTOME_METABOLISM_OF_PROTEINS	518	Genes involved in Metabolism of proteins	63	0.1216	2.00E-18	6.89E-16
HALLMARK_MITOTIC_SPINDLE	200	Genes important for mitotic spindle assembly.	38	0.19	1.83E-17	5.03E-15
KEGG_RIBOSOME	88	Ribosome	25	0.2841	5.70E-17	1.31E-14
REACTOME_METABOLISM_OF_RNA	330	Genes involved in Metabolism of RNA	46	0.1394	1.79E-16	3.53E-14
HALLMARK_E2F_TARGETS	200	Genes encoding cell cycle related targets of E2F transcription factors.	35	0.175	6.31E-16	1.09E-13
REACTOME_INFLUENZA_LIFE_CYCLE	203	Genes involved in Influenza Life Cycle	35	0.1724	1.02E-15	1.56E-13
REACTOME_METABOLISM_OF_MRNA	284	Genes involved in Metabolism of mRNA	40	0.1408	1.15E-14	1.59E-12

Gene/Gene Set Overlap Matrix

Entrez Gene Id	Gene Symbol	Gene Description	REACTOME_CELL_CYCLE	REACTOME_IMMUNE_SYSTEM	REACTOME_ADAPTIVE_IMMUNE_SYSTEM	REACTOME_METABOLISM_OF_PROTEINS	HALLMARK_MITOTIC_SPINDLE	KEGG_RIBOSOME	REACTOME_METABOLISM_OF_RNA	HALLMARK_E2F_TARGETS	REACTOME_INFLUENZA_LIFE_CYCLE	REACTOME_METABOLISM_OF_MRNA
7311	UBA52	ubiquitin A-52 residue ribosomal protein fusion product 1	REACTOME_CELL_CYCLE	REACTOME_IMMUNE_SYSTEM	REACTOME_ADAPTIVE_IMMUNE_SYSTEM	REACTOME_METABOLISM_OF_PROTEINS		KEGG_RIBOSOME	REACTOME_METABOLISM_OF_RNA		REACTOME_INFLUENZA_LIFE_CYCLE	REACTOME_METABOLISM_OF_MRNA
6396	SEC13	SEC13 homolog (S. cerevisiae)	REACTOME_CELL_CYCLE	REACTOME_IMMUNE_SYSTEM	REACTOME_ADAPTIVE_IMMUNE_SYSTEM	REACTOME_METABOLISM_OF_PROTEINS						
9493	KIF23	kinesin family member 23	REACTOME_CELL_CYCLE	REACTOME_IMMUNE_SYSTEM	REACTOME_ADAPTIVE_IMMUNE_SYSTEM		HALLMARK_MITOTIC_SPINDLE					
5687	PSMA6	proteasome (prosome, macropain) subunit, alpha type, 6	REACTOME_CELL_CYCLE	REACTOME_IMMUNE_SYSTEM	REACTOME_ADAPTIVE_IMMUNE_SYSTEM				REACTOME_METABOLISM_OF_RNA			REACTOME_METABOLISM_OF_MRNA
5713	PSMD7	proteasome (prosome, macropain) 26S subunit, non-ATPase, 7	REACTOME_CELL_CYCLE	REACTOME_IMMUNE_SYSTEM	REACTOME_ADAPTIVE_IMMUNE_SYSTEM				REACTOME_METABOLISM_OF_RNA			REACTOME_METABOLISM_OF_MRNA
5686	PSMA5	proteasome (prosome, macropain) subunit, alpha type, 5	REACTOME_CELL_CYCLE	REACTOME_IMMUNE_SYSTEM	REACTOME_ADAPTIVE_IMMUNE_SYSTEM				REACTOME_METABOLISM_OF_RNA			REACTOME_METABOLISM_OF_MRNA
5717	PSMD11	proteasome (prosome, macropain) 26S subunit, non-ATPase, 11	REACTOME_CELL_CYCLE	REACTOME_IMMUNE_SYSTEM	REACTOME_ADAPTIVE_IMMUNE_SYSTEM				REACTOME_METABOLISM_OF_RNA			REACTOME_METABOLISM_OF_MRNA
5721	PSME2	proteasome (prosome, macropain) activator subunit 2 (PA28 beta)	REACTOME_CELL_CYCLE	REACTOME_IMMUNE_SYSTEM	REACTOME_ADAPTIVE_IMMUNE_SYSTEM				REACTOME_METABOLISM_OF_RNA			REACTOME_METABOLISM_OF_MRNA
5705	PSMC5	proteasome (prosome, macropain) 26S subunit, ATPase, 5	REACTOME_CELL_CYCLE	REACTOME_IMMUNE_SYSTEM	REACTOME_ADAPTIVE_IMMUNE_SYSTEM				REACTOME_METABOLISM_OF_RNA			REACTOME_METABOLISM_OF_MRNA
11065	UBE2C	ubiquitin-conjugating enzyme E2C	REACTOME_CELL_CYCLE	REACTOME_IMMUNE_SYSTEM	REACTOME_ADAPTIVE_IMMUNE_SYSTEM							
5527	PPP2R5C	protein phosphatase 2, regulatory subunit B', gamma	REACTOME_CELL_CYCLE	REACTOME_IMMUNE_SYSTEM	REACTOME_ADAPTIVE_IMMUNE_SYSTEM							
11258	DCTN3	dynactin 3 (p22)	REACTOME_CELL_CYCLE	REACTOME_IMMUNE_SYSTEM	REACTOME_ADAPTIVE_IMMUNE_SYSTEM							
4926	NUMA1	nuclear mitotic apparatus protein 1	REACTOME_CELL_CYCLE				HALLMARK_MITOTIC_SPINDLE					
5116	PCNT	pericentrin	REACTOME_CELL_CYCLE				HALLMARK_MITOTIC_SPINDLE					
11190	CEP250	centrosomal protein 250kDa	REACTOME_CELL_CYCLE				HALLMARK_MITOTIC_SPINDLE					
55125	CEP192	centrosomal protein 192kDa	REACTOME_CELL_CYCLE				HALLMARK_MITOTIC_SPINDLE					
55722	CEP72	centrosomal protein 72kDa	REACTOME_CELL_CYCLE				HALLMARK_MITOTIC_SPINDLE					
85378	TUBGCP6	tubulin, gamma complex associated protein 6	REACTOME_CELL_CYCLE				HALLMARK_MITOTIC_SPINDLE					
25777	SUN2	Sad1 and UNC84 domain containing 2	REACTOME_CELL_CYCLE				HALLMARK_MITOTIC_SPINDLE					
7514	XPO1	exportin 1 (CRM1 homolog, yeast)	REACTOME_CELL_CYCLE						REACTOME_METABOLISM_OF_RNA	HALLMARK_E2F_TARGETS	REACTOME_INFLUENZA_LIFE_CYCLE	REACTOME_METABOLISM_OF_MRNA
4172	MCM3	minichromosome maintenance complex component 3	REACTOME_CELL_CYCLE							HALLMARK_E2F_TARGETS		
9212	AURKB	aurora kinase B	REACTOME_CELL_CYCLE							HALLMARK_E2F_TARGETS		
10274	STAG1	stromal antigen 1	REACTOME_CELL_CYCLE							HALLMARK_E2F_TARGETS		
5424	POLD1	polymerase (DNA directed), delta 1, catalytic subunit 125kDa	REACTOME_CELL_CYCLE							HALLMARK_E2F_TARGETS		
147841	SPC24	SPC24, NDC80 kinetochore complex component, homolog (S. cerevisiae)	REACTOME_CELL_CYCLE							HALLMARK_E2F_TARGETS		
1111	CHEK1	CHK1 checkpoint homolog (S. pombe)	REACTOME_CELL_CYCLE							HALLMARK_E2F_TARGETS		
11200	CHEK2	CHK2 checkpoint homolog (S. pombe)	REACTOME_CELL_CYCLE							HALLMARK_E2F_TARGETS		
5931	RBBP7	retinoblastoma binding protein 7	REACTOME_CELL_CYCLE							HALLMARK_E2F_TARGETS		
4869	NPM1	nucleophosmin (nucleolar phosphoprotein B23, numatrin)	REACTOME_CELL_CYCLE									
5905	RANGAP1	Ran GTPase activating protein 1	REACTOME_CELL_CYCLE									
8318	CDC45	cell division cycle 45 homolog (S. cerevisiae)	REACTOME_CELL_CYCLE									
4998	ORC1	origin recognition complex, subunit 1	REACTOME_CELL_CYCLE									
84515	MCM8	minichromosome maintenance complex component 8	REACTOME_CELL_CYCLE									
23421	ITGB3BP	integrin beta 3 binding protein (beta3-endonexin)	REACTOME_CELL_CYCLE									
151648	SGOL1	shugoshin-like 1 (S. pombe)	REACTOME_CELL_CYCLE									
10735	STAG2	stromal antigen 2	REACTOME_CELL_CYCLE									
83540	NUF2	NUF2, NDC80 kinetochore complex component, homolog (S. cerevisiae)	REACTOME_CELL_CYCLE									
91687	CENPL	centromere protein L	REACTOME_CELL_CYCLE									
1763	DNA2	DNA replication helicase 2 homolog (yeast)	REACTOME_CELL_CYCLE									
995	CDC25C	cell division cycle 25 homolog C (S. pombe)	REACTOME_CELL_CYCLE									
3065	HDAC1	histone deacetylase 1	REACTOME_CELL_CYCLE									
22897	CEP164	centrosomal protein 164kDa	REACTOME_CELL_CYCLE									
121441	NEDD1	neural precursor cell expressed, developmentally down-regulated 1	REACTOME_CELL_CYCLE									
8636	SSNA1	Sjogren syndrome nuclear autoantigen 1	REACTOME_CELL_CYCLE									
1859	DYRK1A	dual-specificity tyrosine-(Y)-phosphorylation regulated kinase 1A	REACTOME_CELL_CYCLE									
472	ATM	ataxia telangiectasia mutated	REACTOME_CELL_CYCLE									
64326	RFWD2	ring finger and WD repeat domain 2	REACTOME_CELL_CYCLE									
5883	RAD9A	RAD9 homolog A (S. pombe)	REACTOME_CELL_CYCLE									
5884	RAD17	RAD17 homolog (S. pombe)	REACTOME_CELL_CYCLE									
84126	ATRIP	ATR interacting protein	REACTOME_CELL_CYCLE									
65057	ACD	adrenocortical dysplasia homolog (mouse)	REACTOME_CELL_CYCLE									
23224	SYNE2	spectrin repeat containing, nuclear envelope 2	REACTOME_CELL_CYCLE									
55320	MIS18BP1	MIS18 binding protein 1	REACTOME_CELL_CYCLE									
55355	HJURP	Holliday junction recognition protein	REACTOME_CELL_CYCLE									
55294	FBXW7	F-box and WD repeat domain containing 7		REACTOME_IMMUNE_SYSTEM	REACTOME_ADAPTIVE_IMMUNE_SYSTEM	REACTOME_METABOLISM_OF_PROTEINS						
29127	RACGAP1	Rac GTPase activating protein 1		REACTOME_IMMUNE_SYSTEM	REACTOME_ADAPTIVE_IMMUNE_SYSTEM		HALLMARK_MITOTIC_SPINDLE			HALLMARK_E2F_TARGETS		
4690	NCK1	NCK adaptor protein 1		REACTOME_IMMUNE_SYSTEM	REACTOME_ADAPTIVE_IMMUNE_SYSTEM		HALLMARK_MITOTIC_SPINDLE					
2534	FYN	FYN oncogene related to SRC, FGR, YES		REACTOME_IMMUNE_SYSTEM	REACTOME_ADAPTIVE_IMMUNE_SYSTEM							
6923	TCEB2	transcription elongation factor B (SIII), polypeptide 2 (18kDa, elongin B)		REACTOME_IMMUNE_SYSTEM	REACTOME_ADAPTIVE_IMMUNE_SYSTEM							
3932	LCK	lymphocyte-specific protein tyrosine kinase		REACTOME_IMMUNE_SYSTEM	REACTOME_ADAPTIVE_IMMUNE_SYSTEM							
919	CD247	CD247 molecule		REACTOME_IMMUNE_SYSTEM	REACTOME_ADAPTIVE_IMMUNE_SYSTEM							
8905	AP1S2	adaptor-related protein complex 1, sigma 2 subunit		REACTOME_IMMUNE_SYSTEM	REACTOME_ADAPTIVE_IMMUNE_SYSTEM							
23291	FBXW11	F-box and WD repeat domain containing 11		REACTOME_IMMUNE_SYSTEM	REACTOME_ADAPTIVE_IMMUNE_SYSTEM							
7409	VAV1	vav 1 guanine nucleotide exchange factor		REACTOME_IMMUNE_SYSTEM	REACTOME_ADAPTIVE_IMMUNE_SYSTEM							
5335	PLCG1	phospholipase C, gamma 1		REACTOME_IMMUNE_SYSTEM	REACTOME_ADAPTIVE_IMMUNE_SYSTEM							
5579	PRKCB	protein kinase C, beta		REACTOME_IMMUNE_SYSTEM	REACTOME_ADAPTIVE_IMMUNE_SYSTEM							
208	AKT2	v-akt murine thymoma viral oncogene homolog 2		REACTOME_IMMUNE_SYSTEM	REACTOME_ADAPTIVE_IMMUNE_SYSTEM							
5336	PLCG2	phospholipase C, gamma 2 (phosphatidylinositol-specific)		REACTOME_IMMUNE_SYSTEM	REACTOME_ADAPTIVE_IMMUNE_SYSTEM							
6199	RPS6KB2	ribosomal protein S6 kinase, 70kDa, polypeptide 2		REACTOME_IMMUNE_SYSTEM	REACTOME_ADAPTIVE_IMMUNE_SYSTEM							
3709	ITPR2	inositol 1,4,5-trisphosphate receptor, type 2		REACTOME_IMMUNE_SYSTEM	REACTOME_ADAPTIVE_IMMUNE_SYSTEM							
3710	ITPR3	inositol 1,4,5-trisphosphate receptor, type 3		REACTOME_IMMUNE_SYSTEM	REACTOME_ADAPTIVE_IMMUNE_SYSTEM							
808	CALM3	calmodulin 3 (phosphorylase kinase, delta)		REACTOME_IMMUNE_SYSTEM	REACTOME_ADAPTIVE_IMMUNE_SYSTEM							
64223	MLST8	MTOR associated protein, LST8 homolog (S. cerevisiae)		REACTOME_IMMUNE_SYSTEM	REACTOME_ADAPTIVE_IMMUNE_SYSTEM							
6885	MAP3K7	mitogen-activated protein kinase kinase kinase 7		REACTOME_IMMUNE_SYSTEM	REACTOME_ADAPTIVE_IMMUNE_SYSTEM							
3551	IKBKB	inhibitor of kappa light polypeptide gene enhancer in B-cells, kinase beta		REACTOME_IMMUNE_SYSTEM	REACTOME_ADAPTIVE_IMMUNE_SYSTEM							
3164	NR4A1	nuclear receptor subfamily 4, group A, member 1		REACTOME_IMMUNE_SYSTEM	REACTOME_ADAPTIVE_IMMUNE_SYSTEM							
25780	RASGRP3	RAS guanyl releasing protein 3 (calcium and DAG-regulated)		REACTOME_IMMUNE_SYSTEM	REACTOME_ADAPTIVE_IMMUNE_SYSTEM							
973	CD79A	CD79a molecule, immunoglobulin-associated alpha		REACTOME_IMMUNE_SYSTEM	REACTOME_ADAPTIVE_IMMUNE_SYSTEM							
10054	UBA2	ubiquitin-like modifier activating enzyme 2		REACTOME_IMMUNE_SYSTEM	REACTOME_ADAPTIVE_IMMUNE_SYSTEM							
10075	HUWE1	HECT, UBA and WWE domain containing 1		REACTOME_IMMUNE_SYSTEM	REACTOME_ADAPTIVE_IMMUNE_SYSTEM							
7337	UBE3A	ubiquitin protein ligase E3A		REACTOME_IMMUNE_SYSTEM	REACTOME_ADAPTIVE_IMMUNE_SYSTEM							
8453	CUL2	cullin 2		REACTOME_IMMUNE_SYSTEM	REACTOME_ADAPTIVE_IMMUNE_SYSTEM							
7317	UBA1	ubiquitin-like modifier activating enzyme 1		REACTOME_IMMUNE_SYSTEM	REACTOME_ADAPTIVE_IMMUNE_SYSTEM							
7326	UBE2G1	ubiquitin-conjugating enzyme E2G 1		REACTOME_IMMUNE_SYSTEM	REACTOME_ADAPTIVE_IMMUNE_SYSTEM							
7327	UBE2G2	ubiquitin-conjugating enzyme E2G 2		REACTOME_IMMUNE_SYSTEM	REACTOME_ADAPTIVE_IMMUNE_SYSTEM							
140739	UBE2F	ubiquitin-conjugating enzyme E2F (putative)		REACTOME_IMMUNE_SYSTEM	REACTOME_ADAPTIVE_IMMUNE_SYSTEM							
22888	UBOX5	U-box domain containing 5		REACTOME_IMMUNE_SYSTEM	REACTOME_ADAPTIVE_IMMUNE_SYSTEM							
26259	FBXW8	F-box and WD repeat domain containing 8		REACTOME_IMMUNE_SYSTEM	REACTOME_ADAPTIVE_IMMUNE_SYSTEM							
55585	UBE2Q1	ubiquitin-conjugating enzyme E2Q family member 1		REACTOME_IMMUNE_SYSTEM	REACTOME_ADAPTIVE_IMMUNE_SYSTEM							
26118	WSB1	WD repeat and SOCS box containing 1		REACTOME_IMMUNE_SYSTEM	REACTOME_ADAPTIVE_IMMUNE_SYSTEM							
151636	DTX3L	deltex 3-like (Drosophila)		REACTOME_IMMUNE_SYSTEM	REACTOME_ADAPTIVE_IMMUNE_SYSTEM							
255488	RNF144B	ring finger protein 144B		REACTOME_IMMUNE_SYSTEM	REACTOME_ADAPTIVE_IMMUNE_SYSTEM							
55182	RNF220	ring finger protein 220		REACTOME_IMMUNE_SYSTEM	REACTOME_ADAPTIVE_IMMUNE_SYSTEM							
6049	RNF6	ring finger protein (C3H2C3 type) 6		REACTOME_IMMUNE_SYSTEM	REACTOME_ADAPTIVE_IMMUNE_SYSTEM							
7174	TPP2	tripeptidyl peptidase II		REACTOME_IMMUNE_SYSTEM	REACTOME_ADAPTIVE_IMMUNE_SYSTEM							
80196	RNF34	ring finger protein 34		REACTOME_IMMUNE_SYSTEM	REACTOME_ADAPTIVE_IMMUNE_SYSTEM							
9666	DZIP3	DAZ interacting protein 3, zinc finger		REACTOME_IMMUNE_SYSTEM	REACTOME_ADAPTIVE_IMMUNE_SYSTEM							
4689	NCF4	neutrophil cytosolic factor 4, 40kDa		REACTOME_IMMUNE_SYSTEM	REACTOME_ADAPTIVE_IMMUNE_SYSTEM							
5641	LGMN	legumain		REACTOME_IMMUNE_SYSTEM	REACTOME_ADAPTIVE_IMMUNE_SYSTEM							
5788	PTPRC	protein tyrosine phosphatase, receptor type, C		REACTOME_IMMUNE_SYSTEM	REACTOME_ADAPTIVE_IMMUNE_SYSTEM							
5908	RAP1B	RAP1B, member of RAS oncogene family		REACTOME_IMMUNE_SYSTEM	REACTOME_ADAPTIVE_IMMUNE_SYSTEM							
3689	ITGB2	integrin, beta 2 (complement component 3 receptor 3 and 4 subunit)		REACTOME_IMMUNE_SYSTEM	REACTOME_ADAPTIVE_IMMUNE_SYSTEM							
55824	PAG1	phosphoprotein associated with glycosphingolipid microdomains 1		REACTOME_IMMUNE_SYSTEM	REACTOME_ADAPTIVE_IMMUNE_SYSTEM							
3113	HLA-DPA1	major histocompatibility complex, class II, DP alpha 1		REACTOME_IMMUNE_SYSTEM	REACTOME_ADAPTIVE_IMMUNE_SYSTEM							
3108	HLA-DMA	major histocompatibility complex, class II, DM alpha		REACTOME_IMMUNE_SYSTEM	REACTOME_ADAPTIVE_IMMUNE_SYSTEM							
972	CD74	CD74 molecule, major histocompatibility complex, class II invariant chain		REACTOME_IMMUNE_SYSTEM	REACTOME_ADAPTIVE_IMMUNE_SYSTEM							
958	CD40	CD40 molecule, TNF receptor superfamily member 5		REACTOME_IMMUNE_SYSTEM	REACTOME_ADAPTIVE_IMMUNE_SYSTEM							
10671	DCTN6	dynactin 6		REACTOME_IMMUNE_SYSTEM	REACTOME_ADAPTIVE_IMMUNE_SYSTEM							
10120	ACTR1B	ARP1 actin-related protein 1 homolog B, centractin beta (yeast)		REACTOME_IMMUNE_SYSTEM	REACTOME_ADAPTIVE_IMMUNE_SYSTEM							
3695	ITGB7	integrin, beta 7		REACTOME_IMMUNE_SYSTEM	REACTOME_ADAPTIVE_IMMUNE_SYSTEM							
10859	LILRB1	leukocyte immunoglobulin-like receptor, subfamily B (with TM and ITIM domains), member 1		REACTOME_IMMUNE_SYSTEM	REACTOME_ADAPTIVE_IMMUNE_SYSTEM							
9972	NUP153	nucleoporin 153kDa		REACTOME_IMMUNE_SYSTEM					REACTOME_METABOLISM_OF_RNA	HALLMARK_E2F_TARGETS	REACTOME_INFLUENZA_LIFE_CYCLE	
8086	AAAS	achalasia, adrenocortical insufficiency, alacrimia		REACTOME_IMMUNE_SYSTEM					REACTOME_METABOLISM_OF_RNA		REACTOME_INFLUENZA_LIFE_CYCLE	
9818	NUPL1	nucleoporin like 1		REACTOME_IMMUNE_SYSTEM					REACTOME_METABOLISM_OF_RNA		REACTOME_INFLUENZA_LIFE_CYCLE	
11097	NUPL2	nucleoporin like 2		REACTOME_IMMUNE_SYSTEM					REACTOME_METABOLISM_OF_RNA		REACTOME_INFLUENZA_LIFE_CYCLE	
23511	NUP188	nucleoporin 188kDa		REACTOME_IMMUNE_SYSTEM					REACTOME_METABOLISM_OF_RNA		REACTOME_INFLUENZA_LIFE_CYCLE	
23636	NUP62	nucleoporin 62kDa		REACTOME_IMMUNE_SYSTEM					REACTOME_METABOLISM_OF_RNA		REACTOME_INFLUENZA_LIFE_CYCLE	
2033	EP300	E1A binding protein p300		REACTOME_IMMUNE_SYSTEM								
5289	PIK3C3	phosphoinositide-3-kinase, class 3		REACTOME_IMMUNE_SYSTEM								
3661	IRF3	interferon regulatory factor 3		REACTOME_IMMUNE_SYSTEM								
25970	SH2B1	SH2B adaptor protein 1		REACTOME_IMMUNE_SYSTEM								
6773	STAT2	signal transducer and activator of transcription 2, 113kDa		REACTOME_IMMUNE_SYSTEM								
6776	STAT5A	signal transducer and activator of transcription 5A		REACTOME_IMMUNE_SYSTEM								
8878	SQSTM1	sequestosome 1		REACTOME_IMMUNE_SYSTEM								
51135	IRAK4	interleukin-1 receptor-associated kinase 4		REACTOME_IMMUNE_SYSTEM								
5602	MAPK10	mitogen-activated protein kinase 10		REACTOME_IMMUNE_SYSTEM								
51295	ECSIT	ECSIT homolog (Drosophila)		REACTOME_IMMUNE_SYSTEM								
4205	MEF2A	myocyte enhancer factor 2A		REACTOME_IMMUNE_SYSTEM								
10010	TANK	TRAF family member-associated NFKB activator		REACTOME_IMMUNE_SYSTEM								
51447	IP6K2	inositol hexakisphosphate kinase 2		REACTOME_IMMUNE_SYSTEM								
8672	EIF4G3	eukaryotic translation initiation factor 4 gamma, 3		REACTOME_IMMUNE_SYSTEM								
817	CAMK2D	calcium/calmodulin-dependent protein kinase II delta		REACTOME_IMMUNE_SYSTEM								
7706	TRIM25	tripartite motif containing 25		REACTOME_IMMUNE_SYSTEM								
7297	TYK2	tyrosine kinase 2		REACTOME_IMMUNE_SYSTEM								
5771	PTPN2	protein tyrosine phosphatase, non-receptor type 2		REACTOME_IMMUNE_SYSTEM								
6672	SP100	SP100 nuclear antigen		REACTOME_IMMUNE_SYSTEM								
3575	IL7R	interleukin 7 receptor		REACTOME_IMMUNE_SYSTEM								
5094	PCBP2	poly(rC) binding protein 2		REACTOME_IMMUNE_SYSTEM								
1386	ATF2	activating transcription factor 2		REACTOME_IMMUNE_SYSTEM								
177	AGER	advanced glycosylation end product-specific receptor		REACTOME_IMMUNE_SYSTEM								
81793	TLR10	toll-like receptor 10		REACTOME_IMMUNE_SYSTEM								
10695	CNPY3	canopy 3 homolog (zebrafish)		REACTOME_IMMUNE_SYSTEM								
55593	OTUD5	OTU domain containing 5		REACTOME_IMMUNE_SYSTEM								
6194	RPS6	ribosomal protein S6				REACTOME_METABOLISM_OF_PROTEINS		KEGG_RIBOSOME	REACTOME_METABOLISM_OF_RNA		REACTOME_INFLUENZA_LIFE_CYCLE	REACTOME_METABOLISM_OF_MRNA
6187	RPS2	ribosomal protein S2				REACTOME_METABOLISM_OF_PROTEINS		KEGG_RIBOSOME	REACTOME_METABOLISM_OF_RNA		REACTOME_INFLUENZA_LIFE_CYCLE	REACTOME_METABOLISM_OF_MRNA
6188	RPS3	ribosomal protein S3				REACTOME_METABOLISM_OF_PROTEINS		KEGG_RIBOSOME	REACTOME_METABOLISM_OF_RNA		REACTOME_INFLUENZA_LIFE_CYCLE	REACTOME_METABOLISM_OF_MRNA
6175	RPLP0	ribosomal protein, large, P0				REACTOME_METABOLISM_OF_PROTEINS		KEGG_RIBOSOME	REACTOME_METABOLISM_OF_RNA		REACTOME_INFLUENZA_LIFE_CYCLE	REACTOME_METABOLISM_OF_MRNA
6135	RPL11	ribosomal protein L11				REACTOME_METABOLISM_OF_PROTEINS		KEGG_RIBOSOME	REACTOME_METABOLISM_OF_RNA		REACTOME_INFLUENZA_LIFE_CYCLE	REACTOME_METABOLISM_OF_MRNA
6125	RPL5	ribosomal protein L5				REACTOME_METABOLISM_OF_PROTEINS		KEGG_RIBOSOME	REACTOME_METABOLISM_OF_RNA		REACTOME_INFLUENZA_LIFE_CYCLE	REACTOME_METABOLISM_OF_MRNA
9349	RPL23	ribosomal protein L23				REACTOME_METABOLISM_OF_PROTEINS		KEGG_RIBOSOME	REACTOME_METABOLISM_OF_RNA		REACTOME_INFLUENZA_LIFE_CYCLE	REACTOME_METABOLISM_OF_MRNA
6206	RPS12	ribosomal protein S12				REACTOME_METABOLISM_OF_PROTEINS		KEGG_RIBOSOME	REACTOME_METABOLISM_OF_RNA		REACTOME_INFLUENZA_LIFE_CYCLE	REACTOME_METABOLISM_OF_MRNA
25873	RPL36	ribosomal protein L36				REACTOME_METABOLISM_OF_PROTEINS		KEGG_RIBOSOME	REACTOME_METABOLISM_OF_RNA		REACTOME_INFLUENZA_LIFE_CYCLE	REACTOME_METABOLISM_OF_MRNA
6189	RPS3A	ribosomal protein S3A				REACTOME_METABOLISM_OF_PROTEINS		KEGG_RIBOSOME	REACTOME_METABOLISM_OF_RNA		REACTOME_INFLUENZA_LIFE_CYCLE	REACTOME_METABOLISM_OF_MRNA
6201	RPS7	ribosomal protein S7				REACTOME_METABOLISM_OF_PROTEINS		KEGG_RIBOSOME	REACTOME_METABOLISM_OF_RNA		REACTOME_INFLUENZA_LIFE_CYCLE	REACTOME_METABOLISM_OF_MRNA
6205	RPS11	ribosomal protein S11				REACTOME_METABOLISM_OF_PROTEINS		KEGG_RIBOSOME	REACTOME_METABOLISM_OF_RNA		REACTOME_INFLUENZA_LIFE_CYCLE	REACTOME_METABOLISM_OF_MRNA
6222	RPS18	ribosomal protein S18				REACTOME_METABOLISM_OF_PROTEINS		KEGG_RIBOSOME	REACTOME_METABOLISM_OF_RNA		REACTOME_INFLUENZA_LIFE_CYCLE	REACTOME_METABOLISM_OF_MRNA
6234	RPS28	ribosomal protein S28				REACTOME_METABOLISM_OF_PROTEINS		KEGG_RIBOSOME	REACTOME_METABOLISM_OF_RNA		REACTOME_INFLUENZA_LIFE_CYCLE	REACTOME_METABOLISM_OF_MRNA
23521	RPL13A	ribosomal protein L13a				REACTOME_METABOLISM_OF_PROTEINS		KEGG_RIBOSOME	REACTOME_METABOLISM_OF_RNA		REACTOME_INFLUENZA_LIFE_CYCLE	REACTOME_METABOLISM_OF_MRNA
11224	RPL35	ribosomal protein L35				REACTOME_METABOLISM_OF_PROTEINS		KEGG_RIBOSOME	REACTOME_METABOLISM_OF_RNA		REACTOME_INFLUENZA_LIFE_CYCLE	REACTOME_METABOLISM_OF_MRNA
6124	RPL4	ribosomal protein L4				REACTOME_METABOLISM_OF_PROTEINS		KEGG_RIBOSOME	REACTOME_METABOLISM_OF_RNA		REACTOME_INFLUENZA_LIFE_CYCLE	REACTOME_METABOLISM_OF_MRNA
6134	RPL10	ribosomal protein L10				REACTOME_METABOLISM_OF_PROTEINS		KEGG_RIBOSOME	REACTOME_METABOLISM_OF_RNA		REACTOME_INFLUENZA_LIFE_CYCLE	REACTOME_METABOLISM_OF_MRNA
6137	RPL13	ribosomal protein L13				REACTOME_METABOLISM_OF_PROTEINS		KEGG_RIBOSOME	REACTOME_METABOLISM_OF_RNA		REACTOME_INFLUENZA_LIFE_CYCLE	REACTOME_METABOLISM_OF_MRNA
6139	RPL17	ribosomal protein L17				REACTOME_METABOLISM_OF_PROTEINS		KEGG_RIBOSOME	REACTOME_METABOLISM_OF_RNA		REACTOME_INFLUENZA_LIFE_CYCLE	REACTOME_METABOLISM_OF_MRNA
6142	RPL18A	ribosomal protein L18a				REACTOME_METABOLISM_OF_PROTEINS		KEGG_RIBOSOME	REACTOME_METABOLISM_OF_RNA		REACTOME_INFLUENZA_LIFE_CYCLE	REACTOME_METABOLISM_OF_MRNA
6159	RPL29	ribosomal protein L29				REACTOME_METABOLISM_OF_PROTEINS		KEGG_RIBOSOME	REACTOME_METABOLISM_OF_RNA		REACTOME_INFLUENZA_LIFE_CYCLE	REACTOME_METABOLISM_OF_MRNA
6160	RPL31	ribosomal protein L31				REACTOME_METABOLISM_OF_PROTEINS		KEGG_RIBOSOME	REACTOME_METABOLISM_OF_RNA		REACTOME_INFLUENZA_LIFE_CYCLE	REACTOME_METABOLISM_OF_MRNA
6171	RPL41	ribosomal protein L41				REACTOME_METABOLISM_OF_PROTEINS		KEGG_RIBOSOME	REACTOME_METABOLISM_OF_RNA		REACTOME_INFLUENZA_LIFE_CYCLE	REACTOME_METABOLISM_OF_MRNA
6208	RPS14	ribosomal protein S14				REACTOME_METABOLISM_OF_PROTEINS			REACTOME_METABOLISM_OF_RNA		REACTOME_INFLUENZA_LIFE_CYCLE	REACTOME_METABOLISM_OF_MRNA
2107	ETF1	eukaryotic translation termination factor 1				REACTOME_METABOLISM_OF_PROTEINS			REACTOME_METABOLISM_OF_RNA			REACTOME_METABOLISM_OF_MRNA
10528	NOP56	NOP56 ribonucleoprotein homolog (yeast)				REACTOME_METABOLISM_OF_PROTEINS				HALLMARK_E2F_TARGETS		
1968	EIF2S3	eukaryotic translation initiation factor 2, subunit 3 gamma, 52kDa				REACTOME_METABOLISM_OF_PROTEINS						
27335	EIF3K	eukaryotic translation initiation factor 3, subunit K				REACTOME_METABOLISM_OF_PROTEINS						
90701	SEC11C	SEC11 homolog C (S. cerevisiae)				REACTOME_METABOLISM_OF_PROTEINS						
6748	SSR4	signal sequence receptor, delta				REACTOME_METABOLISM_OF_PROTEINS						
1936	EEF1D	eukaryotic translation elongation factor 1 delta (guanine nucleotide exchange protein)				REACTOME_METABOLISM_OF_PROTEINS						
1937	EEF1G	eukaryotic translation elongation factor 1 gamma				REACTOME_METABOLISM_OF_PROTEINS						
3329	HSPD1	heat shock 60kDa protein 1 (chaperonin)				REACTOME_METABOLISM_OF_PROTEINS						
3421	IDH3G	isocitrate dehydrogenase 3 (NAD+) gamma				REACTOME_METABOLISM_OF_PROTEINS						
1431	CS	citrate synthase				REACTOME_METABOLISM_OF_PROTEINS						
153642	ARSK	arylsulfatase family, member K				REACTOME_METABOLISM_OF_PROTEINS						
9695	EDEM1	ER degradation enhancer, mannosidase alpha-like 1				REACTOME_METABOLISM_OF_PROTEINS						
10113	PREB	prolactin regulatory element binding				REACTOME_METABOLISM_OF_PROTEINS						
5372	PMM1	phosphomannomutase 1				REACTOME_METABOLISM_OF_PROTEINS						
29925	GMPPB	GDP-mannose pyrophosphorylase B				REACTOME_METABOLISM_OF_PROTEINS						
29926	GMPPA	GDP-mannose pyrophosphorylase A				REACTOME_METABOLISM_OF_PROTEINS						
55741	EDEM2	ER degradation enhancer, mannosidase alpha-like 2				REACTOME_METABOLISM_OF_PROTEINS						
55757	UGGT2	UDP-glucose glycoprotein glucosyltransferase 2				REACTOME_METABOLISM_OF_PROTEINS						
56886	UGGT1	UDP-glucose glycoprotein glucosyltransferase 1				REACTOME_METABOLISM_OF_PROTEINS						
80267	EDEM3	ER degradation enhancer, mannosidase alpha-like 3				REACTOME_METABOLISM_OF_PROTEINS						
57171	DOLPP1	dolichyl pyrophosphate phosphatase 1				REACTOME_METABOLISM_OF_PROTEINS						
3998	LMAN1	lectin, mannose-binding, 1				REACTOME_METABOLISM_OF_PROTEINS						
10195	ALG3	asparagine-linked glycosylation 3, alpha-1,3- mannosyltransferase homolog (S. cerevisiae)				REACTOME_METABOLISM_OF_PROTEINS						
23556	PIGN	phosphatidylinositol glycan anchor biosynthesis, class N				REACTOME_METABOLISM_OF_PROTEINS						
5277	PIGA	phosphatidylinositol glycan anchor biosynthesis, class A				REACTOME_METABOLISM_OF_PROTEINS						
84720	PIGO	phosphatidylinositol glycan anchor biosynthesis, class O				REACTOME_METABOLISM_OF_PROTEINS						
2677	GGCX	gamma-glutamyl carboxylase				REACTOME_METABOLISM_OF_PROTEINS						
56913	C1GALT1	core 1 synthase, glycoprotein-N-acetylgalactosamine 3-beta-galactosyltransferase, 1				REACTOME_METABOLISM_OF_PROTEINS						
79623	GALNT14	UDP-N-acetyl-alpha-D-galactosamine:polypeptide N-acetylgalactosaminyltransferase 14 (GalNAc-T14)				REACTOME_METABOLISM_OF_PROTEINS						
10694	CCT8	chaperonin containing TCP1, subunit 8 (theta)				REACTOME_METABOLISM_OF_PROTEINS						
54461	FBXW5	F-box and WD repeat domain containing 5				REACTOME_METABOLISM_OF_PROTEINS						
5204	PFDN5	prefoldin subunit 5				REACTOME_METABOLISM_OF_PROTEINS						
5201	PFDN1	prefoldin subunit 1				REACTOME_METABOLISM_OF_PROTEINS						
131118	DNAJC19	DnaJ (Hsp40) homolog, subfamily C, member 19				REACTOME_METABOLISM_OF_PROTEINS						
9700	ESPL1	extra spindle pole bodies homolog 1 (S. cerevisiae)					HALLMARK_MITOTIC_SPINDLE			HALLMARK_E2F_TARGETS		
1794	DOCK2	dedicator of cytokinesis 2					HALLMARK_MITOTIC_SPINDLE					
9055	PRC1	protein regulator of cytokinesis 1					HALLMARK_MITOTIC_SPINDLE					
89941	RHOT2	ras homolog gene family, member T2					HALLMARK_MITOTIC_SPINDLE					
2934	GSN	gelsolin					HALLMARK_MITOTIC_SPINDLE					
7204	TRIO	triple functional domain (PTPRF interacting)					HALLMARK_MITOTIC_SPINDLE					
9181	ARHGEF2	Rho/Rac guanine nucleotide exchange factor (GEF) 2					HALLMARK_MITOTIC_SPINDLE					
393	ARHGAP4	Rho GTPase activating protein 4					HALLMARK_MITOTIC_SPINDLE					
80119	PIF1	PIF1 5'-to-3' DNA helicase homolog (S. cerevisiae)					HALLMARK_MITOTIC_SPINDLE					
10928	RALBP1	ralA binding protein 1					HALLMARK_MITOTIC_SPINDLE					
8874	ARHGEF7	Rho guanine nucleotide exchange factor (GEF) 7					HALLMARK_MITOTIC_SPINDLE					
9344	TAOK2	TAO kinase 2					HALLMARK_MITOTIC_SPINDLE					
4763	NF1	neurofibromin 1					HALLMARK_MITOTIC_SPINDLE					
1739	DLG1	discs, large homolog 1 (Drosophila)					HALLMARK_MITOTIC_SPINDLE					
667	DST	dystonin					HALLMARK_MITOTIC_SPINDLE					
2037	EPB41L2	erythrocyte membrane protein band 4.1-like 2					HALLMARK_MITOTIC_SPINDLE					
394	ARHGAP5	Rho GTPase activating protein 5					HALLMARK_MITOTIC_SPINDLE					
23192	ATG4B	ATG4 autophagy related 4 homolog B (S. cerevisiae)					HALLMARK_MITOTIC_SPINDLE					
10565	ARFGEF1	ADP-ribosylation factor guanine nucleotide-exchange factor 1 (brefeldin A-inhibited)					HALLMARK_MITOTIC_SPINDLE					
2035	EPB41	erythrocyte membrane protein band 4.1 (elliptocytosis 1, RH-linked)					HALLMARK_MITOTIC_SPINDLE					
51199	NIN	ninein (GSK3B interacting protein)					HALLMARK_MITOTIC_SPINDLE					
3996	LLGL1	lethal giant larvae homolog 1 (Drosophila)					HALLMARK_MITOTIC_SPINDLE					
116840	CNTROB	centrobin, centrosomal BRCA2 interacting protein					HALLMARK_MITOTIC_SPINDLE					
201176	ARHGAP27	Rho GTPase activating protein 27					HALLMARK_MITOTIC_SPINDLE					
6780	STAU1	staufen, RNA binding protein, homolog 1 (Drosophila)					HALLMARK_MITOTIC_SPINDLE					
7461	CLIP2	CAP-GLY domain containing linker protein 2					HALLMARK_MITOTIC_SPINDLE					
84376	HOOK3	hook homolog 3 (Drosophila)					HALLMARK_MITOTIC_SPINDLE					
9771	RAPGEF5	Rap guanine nucleotide exchange factor (GEF) 5					HALLMARK_MITOTIC_SPINDLE					

65110	UPF3A	UPF3 regulator of nonsense transcripts homolog A (yeast)							REACTOME_METABOLISM_OF_RNA			REACTOME_METABOLISM_OF_MRNA
22894	DIS3	DIS3 mitotic control homolog (S. cerevisiae)							REACTOME_METABOLISM_OF_RNA			REACTOME_METABOLISM_OF_MRNA
27257	LSM1	LSM1 homolog, U6 small nuclear RNA associated (S. cerevisiae)							REACTOME_METABOLISM_OF_RNA			REACTOME_METABOLISM_OF_MRNA
4848	CNOT2	CCR4-NOT transcription complex, subunit 2							REACTOME_METABOLISM_OF_RNA			REACTOME_METABOLISM_OF_MRNA
54464	XRN1	5'-3' exoribonuclease 1							REACTOME_METABOLISM_OF_RNA			REACTOME_METABOLISM_OF_MRNA
9337	CNOT8	CCR4-NOT transcription complex, subunit 8							REACTOME_METABOLISM_OF_RNA			REACTOME_METABOLISM_OF_MRNA
5902	RANBP1	RAN binding protein 1								HALLMARK_E2F_TARGETS		
11168	PSIP1	PC4 and SFRS1 interacting protein 1								HALLMARK_E2F_TARGETS		
6434	TRA2B	transformer 2 beta homolog (Drosophila)								HALLMARK_E2F_TARGETS		
4673	NAP1L1	nucleosome assembly protein 1-like 1								HALLMARK_E2F_TARGETS		
4678	NASP	nuclear autoantigenic sperm protein (histone-binding)								HALLMARK_E2F_TARGETS		
4288	MKI67	antigen identified by monoclonal antibody Ki-67								HALLMARK_E2F_TARGETS		
7112	TMPO	thymopoietin								HALLMARK_E2F_TARGETS		
7037	TFRC	transferrin receptor (p90, CD71)								HALLMARK_E2F_TARGETS		
51691	NAA38	N(alpha)-acetyltransferase 38, NatC auxiliary subunit								HALLMARK_E2F_TARGETS		
84844	PHF5A	PHD finger protein 5A								HALLMARK_E2F_TARGETS		
4292	MLH1	mutL homolog 1, colon cancer, nonpolyposis type 2 (E. coli)								HALLMARK_E2F_TARGETS		
9238	TBRG4	transforming growth factor beta regulator 4								HALLMARK_E2F_TARGETS		
5395	PMS2	PMS2 postmeiotic segregation increased 2 (S. cerevisiae)								HALLMARK_E2F_TARGETS		
10615	SPAG5	sperm associated antigen 5								HALLMARK_E2F_TARGETS		
146909	KIF18B	kinesin family member 18B								HALLMARK_E2F_TARGETS		
29893	PSMC3IP	PSMC3 interacting protein								HALLMARK_E2F_TARGETS		
29980	DONSON	downstream neighbor of SON								HALLMARK_E2F_TARGETS		
6941	TCF19	transcription factor 19								HALLMARK_E2F_TARGETS		
79677	SMC6	structural maintenance of chromosomes 6								HALLMARK_E2F_TARGETS		
83461	CDCA3	cell division cycle associated 3								HALLMARK_E2F_TARGETS		
83463	MXD3	MAX dimerization protein 3								HALLMARK_E2F_TARGETS		
9924	PAN2	PAN2 poly(A) specific ribonuclease subunit homolog (S. cerevisiae)								HALLMARK_E2F_TARGETS		
5434	POLR2E	polymerase (RNA) II (DNA directed) polypeptide E, 25kDa									REACTOME_INFLUENZA_LIFE_CYCLE	
2926	GRSF1	G-rich RNA sequence binding factor 1									REACTOME_INFLUENZA_LIFE_CYCLE	
5478	PPIA	peptidylprolyl isomerase A (cyclophilin A)										
9844	ELMO1	engulfment and cell motility 1										
6917	TCEA1	transcription elongation factor A (SII), 1										
7520	XRCC5	X-ray repair complementing defective repair in Chinese hamster cells 5 (double-strand-break rejoining)										
6875	TAF4B	TAF4b RNA polymerase II, TATA box binding protein (TBP)-associated factor, 105kDa										
905	CCNT2	cyclin T2										
2961	GTF2E2	general transcription factor IIE, polypeptide 2, beta 34kDa										
10146	G3BP1	GTPase activating protein (SH3 domain) binding protein 1										
10399	GNB2L1	guanine nucleotide binding protein (G protein), beta polypeptide 2-like 1										
10987	COPS5	COP9 constitutive photomorphogenic homolog subunit 5 (Arabidopsis)										
10155	TRIM28	tripartite motif containing 28										
790	CAD	carbamoyl-phosphate synthetase 2, aspartate transcarbamylase, and dihydroorotase										
3183	HNRNPC	heterogeneous nuclear ribonucleoprotein C (C1/C2)										
3615	IMPDH2	IMP (inosine 5'-monophosphate) dehydrogenase 2										
3181	HNRNPA2B1	heterogeneous nuclear ribonucleoprotein A2/B1										
3068	HDGF	hepatoma-derived growth factor										
11260	XPOT	exportin, tRNA (nuclear export receptor for tRNAs)										
2079	ERH	enhancer of rudimentary homolog (Drosophila)										
3608	ILF2	interleukin enhancer binding factor 2, 45kDa										
6732	SRPK1	SRSF protein kinase 1										
8761	PABPC4	poly(A) binding protein, cytoplasmic 4 (inducible form)										
1841	DTYMK	deoxythymidylate kinase (thymidylate kinase)										
7486	WRN	Werner syndrome, RecQ helicase-like										
10772	SRSF10	serine/arginine-rich splicing factor 10										
2130	EWSR1	Ewing sarcoma breakpoint region 1										
6558	SLC12A2	solute carrier family 12 (sodium/potassium/chloride transporters), member 2										
10592	SMC2	structural maintenance of chromosomes 2										
9156	EXO1	exonuclease 1										
7468	WHSC1	Wolf-Hirschhorn syndrome candidate 1										
10721	POLQ	polymerase (DNA directed), theta										
6760	SS18	synovial sarcoma translocation, chromosome 18										
7290	HIRA	HIR histone cell cycle regulation defective homolog A (S. cerevisiae)										
8899	PRPF4B	PRP4 pre-mRNA processing factor 4 homolog B (yeast)										
37	ACADVL	acyl-CoA dehydrogenase, very long chain										
30	ACAA1	acetyl-CoA acyltransferase 1										
10989	IMMT	inner membrane protein, mitochondrial										
54884	RETSAT	retinol saturase (all-trans-retinol 13,14-reductase)										
3419	IDH3A	isocitrate dehydrogenase 3 (NAD+) alpha										
10449	ACAA2	acetyl-CoA acyltransferase 2										
10312	TCIRG1	T-cell, immune regulator 1, ATPase, H+ transporting, lysosomal V0 subunit A3										
522	ATP5J	ATP synthase, H+ transporting, mitochondrial Fo complex, subunit F6										
509	ATP5C1	ATP synthase, H+ transporting, mitochondrial F1 complex, gamma polypeptide 1										
374291	NDUFS7	NADH dehydrogenase (ubiquinone) Fe-S protein 7, 20kDa (NADH-coenzyme Q reductase)										
4696	NDUFA3	NADH dehydrogenase (ubiquinone) 1 alpha subcomplex, 3, 9kDa										
4708	NDUFB2	NADH dehydrogenase (ubiquinone) 1 beta subcomplex, 2, 8kDa										
4726	NDUFS6	NADH dehydrogenase (ubiquinone) Fe-S protein 6, 13kDa (NADH-coenzyme Q reductase)										
4728	NDUFS8	NADH dehydrogenase (ubiquinone) Fe-S protein 8, 23kDa (NADH-coenzyme Q reductase)										
4729	NDUFV2	NADH dehydrogenase (ubiquinone) flavoprotein 2, 24kDa										
518	ATP5G3	ATP synthase, H+ transporting, mitochondrial Fo complex, subunit C3 (subunit 9)										
10632	ATP5L	ATP synthase, H+ transporting, mitochondrial Fo complex, subunit G										
9551	ATP5J2	ATP synthase, H+ transporting, mitochondrial Fo complex, subunit F2										
3420	IDH3B	isocitrate dehydrogenase 3 (NAD+) beta										
9296	ATP6V1F	ATPase, H+ transporting, lysosomal 14kDa, V1 subunit F										
1353	COX11	COX11 cytochrome c oxidase assembly homolog (yeast)										
55288	RHOT1	ras homolog gene family, member T1										
8402	SLC25A11	solute carrier family 25 (mitochondrial carrier; oxoglutarate carrier), member 11										
4976	OPA1	optic atrophy 1 (autosomal dominant)										
64963	MRPS11	mitochondrial ribosomal protein S11										
9617	MTRF1	mitochondrial translational release factor 1										
5294	PIK3CG	phosphoinositide-3-kinase, catalytic, gamma polypeptide										
200576	PIKFYVE	phosphoinositide kinase, FYVE finger containing										
23396	PIP5K1C	phosphatidylinositol-4-phosphate 5-kinase, type I, gamma										
8396	PIP4K2B	phosphatidylinositol-5-phosphate 4-kinase, type II, beta										
5338	PLD2	phospholipase D2										
81579	PLA2G12A	phospholipase A2, group XIIA										
134429	STARD4	StAR-related lipid transfer (START) domain containing 4										
26230	TIAM2	T-cell lymphoma invasion and metastasis 2										
5297	PI4KA	phosphatidylinositol 4-kinase, catalytic, alpha										
5298	PI4KB	phosphatidylinositol 4-kinase, catalytic, beta										
5287	PIK3C2B	phosphoinositide-3-kinase, class 2, beta polypeptide										
23309	SIN3B	SIN3 homolog B, transcription regulator (yeast)										
9612	NCOR2	nuclear receptor corepressor 2										
9611	NCOR1	nuclear receptor corepressor 1										
8694	DGAT1	diacylglycerol O-acyltransferase 1										
3073	HEXA	hexosaminidase A (alpha polypeptide)										
23646	PLD3	phospholipase D family, member 3										
8776	MTMR1	myotubularin related protein 1										
9110	MTMR4	myotubularin related protein 4										
254531	LPCAT4	lysophosphatidylcholine acyltransferase 4										
5833	PCYT2	phosphate cytidylyltransferase 2, ethanolamine										
1120	CHKB	choline kinase beta										
55500	ETNK1	ethanolamine kinase 1										
50640	PNPLA8	patatin-like phospholipase domain containing 8										
122618	PLD4	phospholipase D family, member 4										
57704	GBA2	glucosidase, beta (bile acid) 2										
2531	KDSR	3-ketodihydrosphingosine reductase										
6576	SLC25A1	solute carrier family 25 (mitochondrial carrier; citrate transporter), member 1										
949	SCARB1	scavenger receptor class B, member 1										
79718	TBL1XR1	transducin (beta)-like 1 X-linked receptor 1										
8202	NCOA3	nuclear receptor coactivator 3										
10025	MED16	mediator complex subunit 16										
6720	SREBF1	sterol regulatory element binding transcription factor 1										
5565	PRKAB2	protein kinase, AMP-activated, beta 2 non-catalytic subunit										
4899	NRF1	nuclear respiratory factor 1										
84693	MCEE	methylmalonyl CoA epimerase										
5019	OXCT1	3-oxoacid CoA transferase 1										
51478	HSD17B7	hydroxysteroid (17-beta) dehydrogenase 7										
4597	MVD	mevalonate (diphospho) decarboxylase										
10478	SLC25A17	solute carrier family 25 (mitochondrial carrier; peroxisomal membrane protein, 34kDa), member 17										
1643	DDB2	damage-specific DNA binding protein 2, 48kDa										
1642	DDB1	damage-specific DNA binding protein 1, 127kDa										
2067	ERCC1	excision repair cross-complementing rodent repair deficiency, complementation group 1 (includes overlapping antisense sequence)										
5893	RAD52	RAD52 homolog (S. cerevisiae)										
5980	REV3L	REV3-like, catalytic subunit of DNA polymerase zeta (yeast)										
2177	FANCD2	Fanconi anemia, complementation group D2										
2189	FANCG	Fanconi anemia, complementation group G										
2175	FANCA	Fanconi anemia, complementation group A										
83990	BRIP1	BRCA1 interacting protein C-terminal helicase 1										
4255	MGMT	O-6-methylguanine-DNA methyltransferase										
4968	OGG1	8-oxoguanine DNA glycosylase										
4913	NTHL1	nth endonuclease III-like 1 (E. coli)										
2074	ERCC6	excision repair cross-complementing rodent repair deficiency, complementation group 6										
121642	ALKBH2	alkB, alkylation repair homolog 2 (E. coli)										
10094	ARPC3	actin related protein 2/3 complex, subunit 3, 21kDa										
100137049	PLA2G4B	phospholipase A2, group IVB (cytosolic)										
3985	LIMK2	LIM domain kinase 2										
10059	DNM1L	dynamin 1-like										
2268	FGR	Gardner-Rasheed feline sarcoma viral (v-fgr) oncogene homolog										
5747	PTK2	PTK2 protein tyrosine kinase 2										
54331	GNG2	guanine nucleotide binding protein (G protein), gamma 2										
5573	PRKAR1A	protein kinase, cAMP-dependent, regulatory, type I, alpha (tissue specific extinguisher 1)										
1606	DGKA	diacylglycerol kinase, alpha 80kDa										
6647	SOD1	superoxide dismutase 1, soluble										
483	ATP1B3	ATPase, Na+/K+ transporting, beta 3 polypeptide										
961	CD47	CD47 molecule										
7422	VEGFA	vascular endothelial growth factor A										
1020	CDK5	cyclin-dependent kinase 5										
10257	ABCC4	ATP-binding cassette, sub-family C (CFTR/MRP), member 4										
6810	STX4	syntaxin 4										
813	CALU	calumenin										
9948	WDR1	WD repeat domain 1										
9056	SLC7A7	solute carrier family 7 (amino acid transporter light chain, y+L system), member 7										
27345	KCNMB4	potassium large conductance calcium-activated channel, subfamily M, beta member 4										
9046	DOK2	docking protein 2, 56kDa										
3020	H3F3A	H3 histone, family 3A										
7804	LRP8	low density lipoprotein receptor-related protein 8, apolipoprotein e receptor										
81704	DOCK8	dedicator of cytokinesis 8										
156	ADRBK1	adrenergic, beta, receptor kinase 1										
29979	UBQLN1	ubiquilin 1										
3676	ITGA4	integrin, alpha 4 (antigen CD49D, alpha 4 subunit of VLA-4 receptor)										
1649	DDIT3	DNA-damage-inducible transcript 3										
10298	PAK4	p21 protein (Cdc42/Rac)-activated kinase 4										
4087	SMAD2	SMAD family member 2										
6510	SLC1A5	solute carrier family 1 (neutral amino acid transporter), member 5										
79602	ADIPOR2	adiponectin receptor 2										
2597	GAPDH	glyceraldehyde-3-phosphate dehydrogenase										
23062	GGA2	golgi-associated, gamma adaptin ear containing, ARF binding protein 2										
8833	GMPS	guanine monphosphate synthetase										
10622	POLR3G	polymerase (RNA) III (DNA directed) polypeptide G (32kD)										
10963	STIP1	stress-induced-phosphoprotein 1										
440	ASNS	asparagine synthetase (glutamine-hydrolyzing)										
7436	VLDLR	very low density lipoprotein receptor										
9521	EEF1E1	eukaryotic translation elongation factor 1 epsilon 1										
5723	PSPH	phosphoserine phosphatase										
11151	CORO1A	coronin, actin binding protein, 1A										
673	BRAF	v-raf murine sarcoma viral oncogene homolog B1										
23385	NCSTN	nicastrin										
221472	FGD2	FYVE, RhoGEF and PH domain containing 2										
9138	ARHGEF1	Rho guanine nucleotide exchange factor (GEF) 1										
10818	FRS2	fibroblast growth factor receptor substrate 2										
4790	NFKB1	nuclear factor of kappa light polypeptide gene enhancer in B-cells 1										
51564	HDAC7	histone deacetylase 7										
83933	HDAC10	histone deacetylase 10										
53615	MBD3	methyl-CpG binding domain protein 3										
51547	SIRT7	sirtuin 7										
1107	CHD3	chromodomain helicase DNA binding protein 3										
4194	MDM4	Mdm4 p53 binding protein homolog (mouse)										
1877	E4F1	E4F transcription factor 1										
80204	FBXO11	F-box protein 11										
8491	MAP4K3	mitogen-activated protein kinase kinase kinase kinase 3										
56922	MCCC1	methylcrotonoyl-CoA carboxylase 1 (alpha)										
7316	UBC	ubiquitin C										
128338	DRAM2	DNA-damage regulated autophagy modulator 2										
23786	BCL2L13	BCL2-like 13 (apoptosis facilitator)										
334	APLP2	amyloid beta (A4) precursor-like protein 2										
84895	FAM73B	family with sequence similarity 73, member B										
50619	DEF6	differentially expressed in FDCP 6 homolog (mouse)										
100	ADA	adenosine deaminase										
9618	TRAF4	TNF receptor-associated factor 4										
7421	VDR	vitamin D (1,25- dihydroxyvitamin D3) receptor										
10057	ABCC5	ATP-binding cassette, sub-family C (CFTR/MRP), member 5										
23654	PLXNB2	plexin B2										
10906	TRAFD1	TRAF-type zinc finger domain containing 1										
3094	HINT1	histidine triad nucleotide binding protein 1										
3275	PRMT2	protein arginine methyltransferase 2										
4033	LRMP	lymphoid-restricted membrane protein										
49856	WRAP73	WD repeat containing, antisense to TP73										
51768	TM7SF3	transmembrane 7 superfamily member 3										
79651	RHBDF2	rhomboid 5 homolog 2 (Drosophila)										
84447	SYVN1	synovial apoptosis inhibitor 1, synoviolin										
4796	TONSL	tonsoku-like, DNA repair protein										
9112	MTA1	metastasis associated 1										
5213	PFKM	phosphofructokinase, muscle										
8295	TRRAP	transformation/transcription domain-associated protein										
7343	UBTF	upstream binding transcription factor, RNA polymerase I										
11235	PDCD10	programmed cell death 10										
8658	TNKS	tankyrase, TRF1-interacting ankyrin-related ADP-ribose polymerase										
10914	PAPOLA	poly(A) polymerase alpha										
2026	ENO2	enolase 2 (gamma, neuronal)										
5394	EXOSC10	exosome component 10										
64895	PAPOLG	poly(A) polymerase gamma										
6499	SKIV2L	superkiller viralicidic activity 2-like (S. cerevisiae)										
9583	ENTPD4	ectonucleoside triphosphate diphosphohydrolase 4										
4864	NPC1	Niemann-Pick disease, type C1										
4126	MANBA	mannosidase, beta A, lysosomal										
53	ACP2	acid phosphatase 2, lysosomal										
2990	GUSB	glucuronidase, beta										
138050	HGSNAT	heparan-alpha-glucosaminide N-acetyltransferase										
1201	CLN3	ceroid-lipofuscinosis, neuronal 3										
26088	GGA1	golgi-associated, gamma adaptin ear containing, ARF binding protein 1										
51172	NAGPA	N-acetylglucosamine-1-phosphodiester alpha-N-acetylglucosaminidase										
84572	GNPTG	N-acetylglucosamine-1-phosphate transferase, gamma subunit										
8943	AP3D1	adaptor-related protein complex 3, delta 1 subunit										
9179	AP4M1	adaptor-related protein complex 4, mu 1 subunit										
30833	NT5C	5', 3'-nucleotidase, cytosolic										
1716	DGUOK	deoxyguanosine kinase										
2987	GUK1	guanylate kinase 1										
30834	ZNRD1	zinc ribbon domain containing 1										
11047	ADRM1	adhesion regulating molecule 1										
9013	TAF1C	TATA box binding protein (TBP)-associated factor, RNA polymerase I, C, 110kDa										
27343	POLL	polymerase (DNA directed), lambda										
4090	SMAD5	SMAD family member 5										
6895	TARBP2	TAR (HIV-1) RNA binding protein 2										
22978	NT5C2	5'-nucleotidase, cytosolic II										
1806	DPYD	dihydropyrimidine dehydrogenase										
56474	CTPS2	CTP synthase II										
5471	PPAT	phosphoribosyl pyrophosphate amidotransferase										
271	AMPD2	adenosine monophosphate deaminase 2										

548644	POLR2J3	polymerase (RNA) II (DNA directed) polypeptide J3										
171568	POLR3H	polymerase (RNA) III (DNA directed) polypeptide H (22.9kD)										
64425	POLR1E	polymerase (RNA) I polypeptide E, 53kDa										
10201	NME6	non-metastatic cells 6, protein expressed in (nucleoside-diphosphate kinase)										
3594	IL12RB1	interleukin 12 receptor, beta 1										
7163	TPD52	tumor protein D52										
16	AARS	alanyl-tRNA synthetase										
1615	DARS	aspartyl-tRNA synthetase										
8683	SRSF9	serine/arginine-rich splicing factor 9										
9416	DDX23	DEAD (Asp-Glu-Ala-Asp) box polypeptide 23										
10250	SRRM1	serine/arginine repetitive matrix 1										
3187	HNRNPH1	heterogeneous nuclear ribonucleoprotein H1 (H)										
55749	CCAR1	cell division cycle and apoptosis regulator 1										
56339	METTL3	methyltransferase like 3										
6789	STK4	serine/threonine kinase 4										
5533	PPP3CC	protein phosphatase 3, catalytic subunit, gamma isozyme										
23162	MAPK8IP3	mitogen-activated protein kinase 8 interacting protein 3										
4616	GADD45B	growth arrest and DNA-damage-inducible, beta										
5871	MAP4K2	mitogen-activated protein kinase kinase kinase kinase 2										
9693	RAPGEF2	Rap guanine nucleotide exchange factor (GEF) 2										
5971	RELB	v-rel reticuloendotheliosis viral oncogene homolog B										
8681	JMJD7-PLA2G4B	JMJD7-PLA2G4B readthrough										
5932	RBBP8	retinoblastoma binding protein 8										
22985	ACIN1	apoptotic chromatin condensation inducer 1										
1655	DDX5	DEAD (Asp-Glu-Ala-Asp) box polypeptide 5										
10465	PPIH	peptidylprolyl isomerase H (cyclophilin H)										
10915	TCERG1	transcription elongation regulator 1										
11325	DDX42	DEAD (Asp-Glu-Ala-Asp) box polypeptide 42										
25766	PRPF40B	PRP40 pre-mRNA processing factor 40 homolog B (S. cerevisiae)										
55119	PRPF38B	PRP38 pre-mRNA processing factor 38 (yeast) domain containing B										
55660	PRPF40A	PRP40 pre-mRNA processing factor 40 homolog A (S. cerevisiae)										
57187	THOC2	THO complex 2										
8896	BUD31	BUD31 homolog (S. cerevisiae)										
5144	PDE4D	phosphodiesterase 4D, cAMP-specific										
83593	RASSF5	Ras association (RalGDS/AF-6) domain family member 5										
28988	DBNL	drebrin-like										
1286	COL4A4	collagen, type IV, alpha 4										
51176	LEF1	lymphoid enhancer-binding factor 1										
3728	JUP	junction plakoglobin										
27148	STK36	serine/threonine kinase 36										
10297	APC2	adenomatosis polyposis coli 2										
2956	MSH6	mutS homolog 6 (E. coli)										
2950	GSTP1	glutathione S-transferase pi 1										
933	CD22	CD22 molecule										
2319	FLOT2	flotillin 2										
10211	FLOT1	flotillin 1										
2908	NR3C1	nuclear receptor subfamily 3, group C, member 1 (glucocorticoid receptor)										
4221	MEN1	multiple endocrine neoplasia I										
126382	NR2C2AP	nuclear receptor 2C2-associated protein										
10781	ZNF266	zinc finger protein 266										
147949	ZNF583	zinc finger protein 583										
148156	ZNF558	zinc finger protein 558										
162967	ZNF320	zinc finger protein 320										
201514	ZNF584	zinc finger protein 584										
284443	ZNF493	zinc finger protein 493										
387032	ZKSCAN4	zinc finger with KRAB and SCAN domains 4										
55786	ZNF415	zinc finger protein 415										
57711	ZNF529	zinc finger protein 529										
7587	ZNF37A	zinc finger protein 37A										
7697	ZNF138	zinc finger protein 138										
7743	ZNF189	zinc finger protein 189										
7762	ZNF215	zinc finger protein 215										
79027	ZNF655	zinc finger protein 655										
80317	ZKSCAN3	zinc finger with KRAB and SCAN domains 3										
10188	TNK2	tyrosine kinase, non-receptor, 2										
113878	DTX2	deltex homolog 2 (Drosophila)										
142678	MIB2	mindbomb homolog 2 (Drosophila)										
4731	NDUFV3	NADH dehydrogenase (ubiquinone) flavoprotein 3, 10kDa										
4725	NDUFS5	NADH dehydrogenase (ubiquinone) Fe-S protein 5, 15kDa (NADH-coenzyme Q reductase)										
8883	NAE1	NEDD8 activating enzyme E1 subunit 1										
55066	PDPR	pyruvate dehydrogenase phosphatase regulatory subunit										
7462	LAT2	linker for activation of T cells family, member 2										
7456	WIPF1	WAS/WASL interacting protein family, member 1										
10219	KLRG1	killer cell lectin-like receptor subfamily G, member 1										
971	CD72	CD72 molecule										
5986	RFNG	RFNG O-fucosylpeptide 3-beta-N-acetylglucosaminyltransferase										
27327	TNRC6A	trinucleotide repeat containing 6A										
5452	POU2F2	POU class 2 homeobox 2										
8543	LMO4	LIM domain only 4										
3177	SLC29A2	solute carrier family 29 (nucleoside transporters), member 2										
7781	SLC30A3	solute carrier family 30 (zinc transporter), member 3										
4363	ABCC1	ATP-binding cassette, sub-family C (CFTR/MRP), member 1										
27032	ATP2C1	ATPase, Ca++ transporting, type 2C, member 1										
23460	ABCA6	ATP-binding cassette, sub-family A (ABC1), member 6										
23456	ABCB10	ATP-binding cassette, sub-family B (MDR/TAP), member 10										
8504	PEX3	peroxisomal biogenesis factor 3										
2030	SLC29A1	solute carrier family 29 (nucleoside transporters), member 1										
11182	SLC2A6	solute carrier family 2 (facilitated glucose transporter), member 6										
51296	SLC15A3	solute carrier family 15, member 3										
6553	SLC9A5	solute carrier family 9 (sodium/hydrogen exchanger), member 5										
9497	SLC4A7	solute carrier family 4, sodium bicarbonate cotransporter, member 7										
254428	SLC41A1	solute carrier family 41, member 1										
10396	ATP8A1	ATPase, aminophospholipid transporter (APLT), class I, type 8A, member 1										
374868	ATP9B	ATPase, class II, type 9B										
1285	COL4A3	collagen, type IV, alpha 3 (Goodpasture antigen)										
4637	MYL6	myosin, light chain 6, alkali, smooth muscle and non-muscle										
64218	SEMA4A	sema domain, immunoglobulin domain (Ig), transmembrane domain (TM) and short cytoplasmic domain, (semaphorin) 4A										
286	ANK1	ankyrin 1, erythrocytic										
288	ANK3	ankyrin 3, node of Ranvier (ankyrin G)										
6925	TCF4	transcription factor 4										
6938	TCF12	transcription factor 12										
197258	FUK	fucokinase										
29085	PHPT1	phosphohistidine phosphatase 1										
3795	KHK	ketohexokinase (fructokinase)										
6778	STAT6	signal transducer and activator of transcription 6, interleukin-4 induced										
23265	EXOC7	exocyst complex component 7										
5261	PHKG2	phosphorylase kinase, gamma 2 (testis)										
122809	SOCS4	suppressor of cytokine signaling 4										
9519	TBPL1	TBP-like 1										
2193	FARSA	phenylalanyl-tRNA synthetase, alpha subunit										
4839	NOP2	NOP2 nucleolar protein homolog (yeast)										
10244	RABEPK	Rab9 effector protein with kelch motifs										
6949	TCOF1	Treacher Collins-Franceschetti syndrome 1										
81887	LAS1L	LAS1-like (S. cerevisiae)										
11052	CPSF6	cleavage and polyadenylation specific factor 6, 68kDa										
2665	GDI2	GDP dissociation inhibitor 2										
51306	FAM13B	family with sequence similarity 13, member B										
55843	ARHGAP15	Rho GTPase activating protein 15										
94134	ARHGAP12	Rho GTPase activating protein 12										
1676	DFFA	DNA fragmentation factor, 45kDa, alpha polypeptide										
6304	SATB1	SATB homeobox 1										
706	TSPO	translocator protein (18kDa)										
4294	MAP3K10	mitogen-activated protein kinase kinase kinase 10										
6595	SMARCA2	SWI/SNF related, matrix associated, actin dependent regulator of chromatin, subfamily a, member 2										
1876	E2F6	E2F transcription factor 6										
6867	TACC1	transforming, acidic coiled-coil containing protein 1										
54888	NSUN2	NOP2/Sun domain family, member 2										
23761	PISD	phosphatidylserine decarboxylase										
10434	LYPLA1	lysophospholipase I										
10919	EHMT2	euchromatic histone-lysine N-methyltransferase 2										
29843	SENP1	SUMO1/sentrin specific peptidase 1										
83637	ZMIZ2	zinc finger, MIZ-type containing 2										
55738	ARFGAP1	ADP-ribosylation factor GTPase activating protein 1										
10897	YIF1A	Yip1 interacting factor homolog A (S. cerevisiae)										
1663	DDX11	DEAD/H (Asp-Glu-Ala-Asp/His) box polypeptide 11										
4240	MFGE8	milk fat globule-EGF factor 8 protein										
26018	LRIG1	leucine-rich repeats and immunoglobulin-like domains 1										
116986	AGAP2	ArfGAP with GTPase domain, ankyrin repeat and PH domain 2										
39	ACAT2	acetyl-CoA acetyltransferase 2										
259307	IL4I1	interleukin 4 induced 1										
2639	GCDH	glutaryl-CoA dehydrogenase										
9588	PRDX6	peroxiredoxin 6										
23173	METAP1	methionyl aminopeptidase 1										
51706	CYB5R1	cytochrome b5 reductase 1										
2011	MARK2	MAP/microtubule affinity-regulating kinase 2										
10494	STK25	serine/threonine kinase 25										
7009	TMBIM6	transmembrane BAX inhibitor motif containing 6										
4660	PPP1R12B	protein phosphatase 1, regulatory subunit 12B										
55577	NAGK	N-acetylglucosamine kinase										
10020	GNE	glucosamine (UDP-N-acetyl)-2-epimerase/N-acetylmannosamine kinase										
3712	IVD	isovaleryl-CoA dehydrogenase										
4301	MLLT4	myeloid/lymphoid or mixed-lineage leukemia (trithorax homolog, Drosophila); translocated to, 4										
201163	FLCN	folliculin										
4661	MYT1	myelin transcription factor 1										
140465	MYL6B	myosin, light chain 6B, alkali, smooth muscle and non-muscle										
2801	GOLGA2	golgin A2										
7178	TPT1	tumor protein, translationally-controlled 1										
92359	CRB3	crumbs homolog 3 (Drosophila)										
8189	SYMPK	symplekin										
8751	ADAM15	ADAM metallopeptidase domain 15										
1265	CNN2	calponin 2										
5310	PKD1	polycystic kidney disease 1 (autosomal dominant)										
3119	HLA-DQB1	major histocompatibility complex, class II, DQ beta 1										
8100	IFT88	intraflagellar transport 88 homolog (Chlamydomonas)										
1997	ELF1	E74-like factor 1 (ets domain transcription factor)										
25989	ULK3	unc-51-like kinase 3 (C. elegans)										
4052	LTBP1	latent transforming growth factor beta binding protein 1										
22884	WDR37	WD repeat domain 37										
23469	PHF3	PHD finger protein 3										
27109	ATP5S	ATP synthase, H+ transporting, mitochondrial Fo complex, subunit s (factor B)										
8729	GBF1	golgi brefeldin A resistant guanine nucleotide exchange factor 1										
8301	PICALM	phosphatidylinositol binding clathrin assembly protein										
27131	SNX5	sorting nexin 5										
9698	PUM1	pumilio homolog 1 (Drosophila)										
11316	COPE	coatomer protein complex, subunit epsilon										
25824	PRDX5	peroxiredoxin 5										
3069	HDLBP	high density lipoprotein binding protein										
55139	ANKZF1	ankyrin repeat and zinc finger domain containing 1										
10994	ILVBL	ilvB (bacterial acetolactate synthase)-like										
54800	KLHL24	kelch-like 24 (Drosophila)										
10643	IGF2BP3	insulin-like growth factor 2 mRNA binding protein 3										
5826	ABCD4	ATP-binding cassette, sub-family D (ALD), member 4										
89845	ABCC10	ATP-binding cassette, sub-family C (CFTR/MRP), member 10										
7335	UBE2V1	ubiquitin-conjugating enzyme E2 variant 1										
8437	RASAL1	RAS protein activator like 1 (GAP1 like)										
84938	ATG4C	ATG4 autophagy related 4 homolog C (S. cerevisiae)										
84971	ATG4D	ATG4 autophagy related 4 homolog D (S. cerevisiae)										
8678	BECN1	beclin 1, autophagy related										
51371	POMP	proteasome maturation protein										
5189	PEX1	peroxisomal biogenesis factor 1										
55670	PEX26	peroxisomal biogenesis factor 26										
10901	DHRS4	dehydrogenase/reductase (SDR family) member 4										
59339	PLEKHA2	pleckstrin homology domain containing, family A (phosphoinositide binding specific) member 2										
9744	ACAP1	ArfGAP with coiled-coil, ankyrin repeat and PH domains 1										
10617	STAMBP	STAM binding protein										
56904	SH3GLB2	SH3-domain GRB2-like endophilin B2										
116983	ACAP3	ArfGAP with coiled-coil, ankyrin repeat and PH domains 3										
23550	PSD4	pleckstrin and Sec7 domain containing 4										
84364	ARFGAP2	ADP-ribosylation factor GTPase activating protein 2										
9015	TAF1A	TATA box binding protein (TBP)-associated factor, RNA polymerase I, A, 48kDa										
8675	STX16	syntaxin 16										
11079	RER1	RER1 retention in endoplasmic reticulum 1 homolog (S. cerevisiae)										
11124	FAF1	Fas (TNFRSF6) associated factor 1										
286151	FBXO43	F-box protein 43										
29904	EEF2K	eukaryotic elongation factor-2 kinase										
3692	EIF6	eukaryotic translation initiation factor 6										
23324	MAN2B2	mannosidase, alpha, class 2B, member 2										
4123	MAN2C1	mannosidase, alpha, class 2C, member 1										
80308	FLAD1	FAD1 flavin adenine dinucleotide synthetase homolog (S. cerevisiae)										
27434	POLM	polymerase (DNA directed), mu										
11284	PNKP	polynucleotide kinase 3'-phosphatase										
3150	HMGN1	high mobility group nucleosome binding domain 1										
146956	EME1	essential meiotic endonuclease 1 homolog 1 (S. pombe)										
7516	XRCC2	X-ray repair complementing defective repair in Chinese hamster cells 2										
8940	TOP3B	topoisomerase (DNA) III beta										
23438	HARS2	histidyl-tRNA synthetase 2, mitochondrial (putative)										
57505	AARS2	alanyl-tRNA synthetase 2, mitochondrial (putative)										
51091	SEPSECS	Sep (O-phosphoserine) tRNA:Sec (selenocysteine) tRNA synthase										
51024	FIS1	fission 1 (mitochondrial outer membrane) homolog (S. cerevisiae)										
9270	ITGB1BP1	integrin beta 1 binding protein 1										
9601	PDIA4	protein disulfide isomerase family A, member 4										
125061	AFMID	arylformamidase										
5831	PYCR1	pyrroline-5-carboxylate reductase 1										
51380	CSAD	cysteine sulfinic acid decarboxylase										
51074	APIP	APAF1 interacting protein										
5998	RGS3	regulator of G-protein signaling 3										
27342	RABGEF1	RAB guanine nucleotide exchange factor (GEF) 1										
64324	NSD1	nuclear receptor binding SET domain protein 1										
84444	DOT1L	DOT1-like, histone H3 methyltransferase (S. cerevisiae)										
91703	ACY3	aspartoacylase (aminocyclase) 3										
5074	PAWR	PRKC, apoptosis, WT1, regulator										
23081	KDM4C	lysine (K)-specific demethylase 4C										
23598	PATZ1	POZ (BTB) and AT hook containing zinc finger 1										
166	AES	amino-terminal enhancer of split										
79412	KREMEN2	kringle containing transmembrane protein 2										
10287	RGS19	regulator of G-protein signaling 19										
79661	NEIL1	nei endonuclease VIII-like 1 (E. coli)										
22797	TFEC	transcription factor EC										
10327	AKR1A1	aldo-keto reductase family 1, member A1 (aldehyde reductase)										
9958	USP15	ubiquitin specific peptidase 15										
26130	GAPVD1	GTPase activating protein and VPS9 domains 1										
51312	SLC25A37	solute carrier family 25, member 37										
8273	SLC10A3	solute carrier family 10 (sodium/bile acid cotransporter family), member 3										
9258	MFHAS1	malignant fibrous histiocytoma amplified sequence 1										
11231	SEC63	SEC63 homolog (S. cerevisiae)										
196294	IMMP1L	IMP1 inner mitochondrial membrane peptidase-like (S. cerevisiae)										
7095	SEC62	SEC62 homolog (S. cerevisiae)										
114049	WBSCR22	Williams Beuren syndrome chromosome region 22										
51451	LCMT1	leucine carboxyl methyltransferase 1										
60487	TRMT11	tRNA methyltransferase 11 homolog (S. cerevisiae)										
2678	GGT1	gamma-glutamyltransferase 1										
4008	LMO7	LIM domain 7										
4026	LPP	LIM domain containing preferred translocation partner in lipoma										
50615	IL21R	interleukin 21 receptor										
132158	GLYCTK	glycerate kinase										
80347	COASY	CoA synthase										
8875	VNN2	vanin 2										
55072	RNF31	ring finger protein 31										

27065	D4S234E	DNA segment on chromosome 4 (unique) 234 expressed sequence										
84883	AIFM2	apoptosis-inducing factor, mitochondrion-associated, 2										
5025	P2RX4	purinergic receptor P2X, ligand-gated ion channel, 4										
9144	SYNGR2	synaptogyrin 2										
1690	COCH	coagulation factor C homolog, cochlin (Limulus polyphemus)										
22807	IKZF2	IKAROS family zinc finger 2 (Helios)										
57231	SNX14	sorting nexin 14										
10320	IKZF1	IKAROS family zinc finger 1 (Ikaros)										
3783	KCNN4	potassium intermediate/small conductance calcium-activated channel, subfamily N, member 4										
6801	STRN	striatin, calmodulin binding protein										
3936	LCP1	lymphocyte cytosolic protein 1 (L-plastin)										
83734	ATG10	ATG10 autophagy related 10 homolog (S. cerevisiae)										
286205	SCAI	suppressor of cancer cell invasion										
7011	TEP1	telomerase-associated protein 1										
9943	OXSR1	oxidative-stress responsive 1										
22937	SCAP	SREBF chaperone										
4750	NEK1	NIMA (never in mitosis gene a)-related kinase 1										
340481	ZDHHC21	zinc finger, DHHC-type containing 21										
9988	DMTF1	cyclin D binding myb-like transcription factor 1										
93643	TJAP1	tight junction associated protein 1 (peripheral)										
7391	USF1	upstream transcription factor 1										
240	ALOX5	arachidonate 5-lipoxygenase										
65220	NADK	NAD kinase										
22900	CARD8	caspase recruitment domain family, member 8										
6935	ZEB1	zinc finger E-box binding homeobox 1										
80895	ILKAP	integrin-linked kinase-associated serine/threonine phosphatase										
4603	MYBL1	v-myb myeloblastosis viral oncogene homolog (avian)-like 1										
2971	GTF3A	general transcription factor IIIA										
57018	CCNL1	cyclin L1										
115817	DHRS1	dehydrogenase/reductase (SDR family) member 1										
23564	DDAH2	dimethylarginine dimethylaminohydrolase 2										
1390	CREM	cAMP responsive element modulator										
4133	MAP2	microtubule-associated protein 2										
2356	FPGS	folylpolyglutamate synthase										
23161	SNX13	sorting nexin 13										
7392	USF2	upstream transcription factor 2, c-fos interacting										
1368	CPM	carboxypeptidase M										
79629	OCEL1	occludin/ELL domain containing 1										
203062	TSNARE1	t-SNARE domain containing 1										
931	MS4A1	membrane-spanning 4-domains, subfamily A, member 1										
11238	CA5B	carbonic anhydrase VB, mitochondrial										
9563	H6PD	hexose-6-phosphate dehydrogenase (glucose 1-dehydrogenase)										
9973	CCS	copper chaperone for superoxide dismutase										
79882	ZC3H14	zinc finger CCCH-type containing 14										
80331	DNAJC5	DnaJ (Hsp40) homolog, subfamily C, member 5										
6738	TROVE2	TROVE domain family, member 2										
84264	HAGHL	hydroxyacylglutathione hydrolase-like										
9094	UNC119	unc-119 homolog (C. elegans)										
112483	SAT2	spermidine/spermine N1-acetyltransferase family member 2										
29121	CLEC2D	C-type lectin domain family 2, member D										
3965	LGALS9	lectin, galactoside-binding, soluble, 9										
137902	PXDNL	peroxidasin homolog (Drosophila)-like										
78987	CRELD1	cysteine-rich with EGF-like domains 1										
60401	EDA2R	ectodysplasin A2 receptor										
79085	SLC25A23	solute carrier family 25 (mitochondrial carrier; phosphate carrier), member 23										
8115	TCL1A	T-cell leukemia/lymphoma 1A										
9746	CLSTN3	calsyntenin 3										
256394	SERPINA11	serpin peptidase inhibitor, clade A (alpha-1 antiproteinase, antitrypsin), member 11										
79676	OGFOD2	2-oxoglutarate and iron-dependent oxygenase domain containing 2										
79290	OR13A1	olfactory receptor, family 13, subfamily A, member 1										
100048912	CDKN2B-AS	CDKN2B antisense RNA (non-protein coding)										
100129482	ZNF37BP	zinc finger protein 37B, pseudogene										
100131187	TSTD1	thiosulfate sulfurtransferase (rhodanese)-like domain containing 1										
100132406	NBPF10	neuroblastoma breakpoint family, member 10										
100289678	ZNF783	zinc finger family member 783										
10049	DNAJB6	DnaJ (Hsp40) homolog, subfamily B, member 6										
10101	NUBP2	nucleotide binding protein 2										
10138	YAF2	YY1 associated factor 2										
10147	SUGP2	SURP and G patch domain containing 2										
10169	SERF2	small EDRK-rich factor 2										
10180	RBM6	RNA binding motif protein 6										
10227	MFSD10	major facilitator superfamily domain containing 10										
10336	PCGF3	polycomb group ring finger 3										
10346	TRIM22	tripartite motif containing 22										
10430	TMEM147	transmembrane protein 147										
10444	ZER1	zer-1 homolog (C. elegans)										
10445	MCRS1	microspherule protein 1										
10480	EIF3M	eukaryotic translation initiation factor 3, subunit M										
10519	CIB1	calcium and integrin binding 1 (calmyrin)										
10534	SSSCA1	Sjogren syndrome/scleroderma autoantigen 1										
10539	GLRX3	glutaredoxin 3										
10585	POMT1	protein-O-mannosyltransferase 1										
10607	TBL3	transducin (beta)-like 3										
10625	IVNS1ABP	influenza virus NS1A binding protein										
10641	NPRL2	nitrogen permease regulator-like 2 (S. cerevisiae)										
10724	MGEA5	meningioma expressed antigen 5 (hyaluronidase)										
10807	SDCCAG3	serologically defined colon cancer antigen 3										
10920	COPS8	COP9 constitutive photomorphogenic homolog subunit 8 (Arabidopsis)										
10922	FASTK	Fas-activated serine/threonine kinase										
10927	SPIN1	spindlin 1										
10980	COPS6	COP9 constitutive photomorphogenic homolog subunit 6 (Arabidopsis)										
11011	TLK2	tousled-like kinase 2										
11027	LILRA2	leukocyte immunoglobulin-like receptor, subfamily A (with TM domain), member 2										
11056	DDX52	DEAD (Asp-Glu-Ala-Asp) box polypeptide 52										
1106	CHD2	chromodomain helicase DNA binding protein 2										
11102	RPP14	ribonuclease P/MRP 14kDa subunit										
11118	BTN3A2	butyrophilin, subfamily 3, member A2										
11152	WDR45	WD repeat domain 45										
11193	WBP4	WW domain binding protein 4 (formin binding protein 21)										
11212	PROSC	proline synthetase co-transcribed homolog (bacterial)										
11234	HPS5	Hermansky-Pudlak syndrome 5										
11257	TP53TG1	TP53 target 1 (non-protein coding)										
11262	SP140	SP140 nuclear body protein										
11278	KLF12	Kruppel-like factor 12										
113000	RPUSD1	RNA pseudouridylate synthase domain containing 1										
113177	IZUMO4	IZUMO family member 4										
11322	TMC6	transmembrane channel-like 6										
11342	RNF13	ring finger protein 13										
114800	CCDC85A	coiled-coil domain containing 85A										
114803	MYSM1	Myb-like, SWIRM and MPN domains 1										
114822	RHPN1	rhophilin, Rho GTPase binding protein 1										
114881	OSBPL7	oxysterol binding protein-like 7										
114884	OSBPL10	oxysterol binding protein-like 10										
114971	PTPMT1	protein tyrosine phosphatase, mitochondrial 1										
115350	FCRL1	Fc receptor-like 1										
115399	LRRC56	leucine rich repeat containing 56										
115426	UHRF2	ubiquitin-like with PHD and ring finger domains 2										
116113	FOXP4	forkhead box P4										
117155	CATSPER2	cation channel, sperm associated 2										
118433	RPL23AP7	ribosomal protein L23a pseudogene 7										
1185	CLCN6	chloride channel 6										
118813	ZFYVE27	zinc finger, FYVE domain containing 27										
118881	COMTD1	catechol-O-methyltransferase domain containing 1										
118924	FRA10AC1	fragile site, folic acid type, rare, fra(10)(q23.3) or fra(10)(q24.2) candidate 1										
118980	SFXN2	sideroflexin 2										
1195	CLK1	CDC-like kinase 1										
119504	ANAPC16	anaphase promoting complex subunit 16										
122704	MRPL52	mitochondrial ribosomal protein L52										
123096	SLC25A29	solute carrier family 25, member 29										
123803	NTAN1	N-terminal asparagine amidase										
124093	CCDC78	coiled-coil domain containing 78										
124401	ANKS3	ankyrin repeat and sterile alpha motif domain containing 3										
124540	MSI2	musashi homolog 2 (Drosophila)										
124923	SGK494	uncharacterized serine/threonine-protein kinase SgK494										
124944	C17orf49	chromosome 17 open reading frame 49										
125150	ZSWIM7	zinc finger, SWIM-type containing 7										
125476	INO80C	INO80 complex subunit C										
126432	RINL	Ras and Rab interactor-like										
126526	C19orf47	chromosome 19 open reading frame 47										
127018	LYPLAL1	lysophospholipase-like 1										
127396	ZNF684	zinc finger protein 684										
128308	MRPL55	mitochondrial ribosomal protein L55										
128387	TATDN3	TatD DNase domain containing 3										
129450	TYW5	tRNA-yW synthesizing protein 5										
129563	DIS3L2	DIS3 mitotic control homolog (S. cerevisiae)-like 2										
130074	FAM168B	family with sequence similarity 168, member B										
130872	AHSA2	AHA1, activator of heat shock 90kDa protein ATPase homolog 2 (yeast)										
131601	TPRA1	transmembrane protein, adipocyte asscociated 1										
132160	PPM1M	protein phosphatase, Mg2+/Mn2+ dependent, 1M										
132884	EVC2	Ellis van Creveld syndrome 2										
133015	PACRGL	PARK2 co-regulated-like										
134637	ADAT2	adenosine deaminase, tRNA-specific 2										
136051	ZNF786	zinc finger protein 786										
137695	TMEM68	transmembrane protein 68										
137886	UBXN2B	UBX domain protein 2B										
1396	CRIP1	cysteine-rich protein 1 (intestinal)										
140700	SAMD10	sterile alpha motif domain containing 10										
143570	XRRA1	X-ray radiation resistance associated 1										
144132	DNHD1	dynein heavy chain domain 1										
145407	C14orf37	chromosome 14 open reading frame 37										
145773	FAM81A	family with sequence similarity 81, member A										
146059	CDAN1	congenital dyserythropoietic anemia, type I										
146206	RLTPR	RGD motif, leucine rich repeats, tropomodulin domain and proline-rich containing										
147138	TMC8	transmembrane channel-like 8										
147172	LRRC37BP1	leucine rich repeat containing 37B pseudogene 1										
147699	PPM1N	protein phosphatase, Mg2+/Mn2+ dependent, 1N (putative)										
147965	FAM98C	family with sequence similarity 98, member C										
148206	ZNF714	zinc finger protein 714										
149111	CNIH3	cornichon homolog 3 (Drosophila)										
149175	MANEAL	mannosidase, endo-alpha-like										
149473	CCDC24	coiled-coil domain containing 24										
149775	GNAS-AS1	GNAS antisense RNA 1 (non-protein coding)										
150223	YDJC	YdjC homolog (bacterial)										
150472	CBWD2	COBW domain containing 2										
151230	KLHL23	kelch-like 23 (Drosophila)										
151613	TTC14	tetratricopeptide repeat domain 14										
152926	PPM1K	protein phosphatase, Mg2+/Mn2+ dependent, 1K										
153579	BTNL9	butyrophilin-like 9										
157570	ESCO2	establishment of cohesion 1 homolog 2 (S. cerevisiae)										
157680	VPS13B	vacuolar protein sorting 13 homolog B (yeast)										
158056	MAMDC4	MAM domain containing 4										
158358	KIAA2026	KIAA2026										
165055	CCDC138	coiled-coil domain containing 138										
1657	DMXL1	Dmx-like 1										
167153	PAPD4	PAP associated domain containing 4										
169714	QSOX2	quiescin Q6 sulfhydryl oxidase 2										
170958	ZNF525	zinc finger protein 525										
171017	ZNF384	zinc finger protein 384										
171023	ASXL1	additional sex combs like 1 (Drosophila)										
1773	DNASE1	deoxyribonuclease I										
1801	DPH1	DPH1 homolog (S. cerevisiae)										
1911	PHC1	polyhomeotic homolog 1 (Drosophila)										
1939	EIF2D	eukaryotic translation initiation factor 2D										
196527	ANO6	anoctamin 6										
197320	ZNF778	zinc finger protein 778										
197342	EME2	essential meiotic endonuclease 1 homolog 2 (S. pombe)										
199786	FAM129C	family with sequence similarity 129, member C										
200845	KCTD6	potassium channel tetramerisation domain containing 6										
200894	ARL13B	ADP-ribosylation factor-like 13B										
201254	STRA13	stimulated by retinoic acid 13 homolog (mouse)										
201255	LRRC45	leucine rich repeat containing 45										
201266	SLC39A11	solute carrier family 39 (metal ion transporter), member 11										
201283	AMZ2P1	archaelysin family metallopeptidase 2 pseudogene 1										
201931	TMEM192	transmembrane protein 192										
201965	RWDD4	RWD domain containing 4										
202243	CCDC125	coiled-coil domain containing 125										
2029	ENSA	endosulfine alpha										
203054	ADCK5	aarF domain containing kinase 5										
203328	SUSD3	sushi domain containing 3										
204851	HIPK1	homeodomain interacting protein kinase 1										
219743	TYSND1	trypsin domain containing 1										
219899	TBCEL	tubulin folding cofactor E-like										
219931	TPCN2	two pore segment channel 2										
221016	CCDC7	coiled-coil domain containing 7										
221150	SKA3	spindle and kinetochore associated complex subunit 3										
221302	ZUFSP	zinc finger with UFM1-specific peptidase domain										
222658	KCTD20	potassium channel tetramerisation domain containing 20										
22821	RASA3	RAS p21 protein activator 3										
22890	ZBTB1	zinc finger and BTB domain containing 1										
22902	RUFY3	RUN and FYVE domain containing 3										
22907	DHX30	DEAH (Asp-Glu-Ala-His) box polypeptide 30										
22913	RALY	RNA binding protein, autoantigenic (hnRNP-associated with lethal yellow homolog (mouse))										
22931	RAB18	RAB18, member RAS oncogene family										
23032	USP33	ubiquitin specific peptidase 33										
23077	MYCBP2	MYC binding protein 2										
23093	TTLL5	tubulin tyrosine ligase-like family, member 5										
23125	CAMTA2	calmodulin binding transcription activator 2										
23132	RAD54L2	RAD54-like 2 (S. cerevisiae)										
23155	CLCC1	chloride channel CLIC-like 1										
23180	RFTN1	raftlin, lipid raft linker 1										
23207	PLEKHM2	pleckstrin homology domain containing, family M (with RUN domain) member 2										
23218	NBEAL2	neurobeachin-like 2										
23231	SEL1L3	sel-1 suppressor of lin-12-like 3 (C. elegans)										
23252	OTUD3	OTU domain containing 3										
23261	CAMTA1	calmodulin binding transcription activator 1										
23262	PPIP5K2	diphosphoinositol pentakisphosphate kinase 2										
23274	CLEC16A	C-type lectin domain family 16, member A										
23275	POFUT2	protein O-fucosyltransferase 2										
23318	ZCCHC11	zinc finger, CCHC domain containing 11										
23334	SZT2	seizure threshold 2 homolog (mouse)										
23353	SUN1	Sad1 and UNC84 domain containing 1										
23354	HAUS5	HAUS augmin-like complex, subunit 5										
23360	FNBP4	formin binding protein 4										
23367	LARP1	La ribonucleoprotein domain family, member 1										
23369	PUM2	pumilio homolog 2 (Drosophila)										
23384	SPECC1L	sperm antigen with calponin homology and coiled-coil domains 1-like										
23412	COMMD3	COMM domain containing 3										
23483	TGDS	TDP-glucose 4,6-dehydratase										
23518	R3HDM1	R3H domain containing 1										
23524	SRRM2	serine/arginine repetitive matrix 2										
23559	WBP1	WW domain binding protein 1										
23567	ZNF346	zinc finger protein 346										
23642	SNHG1	small nucleolar RNA host gene 1 (non-protein coding)										
23683	PRKD3	protein kinase D3										
23774	BRD1	bromodomain containing 1										
23787	MTCH1	mitochondrial carrier 1										
24139	EML2	echinoderm microtubule associated protein like 2										
253769	WDR27	WD repeat domain 27										

253832	ZDHHC20	zinc finger, DHHC-type containing 20									
254102	EHBP1L1	EH domain binding protein 1-like 1									
254359	ZDHHC24	zinc finger, DHHC-type containing 24									
255231	MCOLN2	mucolipin 2									
255812	SDHAP1	succinate dehydrogenase complex, subunit A, flavoprotein pseudogene 1									
256364	EML3	echinoderm microtubule associated protein like 3									
256643	CXorf23	chromosome X open reading frame 23									
257218	SHPRH	SNF2 histone linker PHD RING helicase									
25771	TBC1D22A	TBC1 domain family, member 22A									
25792	CIZ1	CDKN1A interacting zinc finger protein 1									
25851	TECPR1	tectonin beta-propeller repeat containing 1									
25864	ABHD14A	abhydrolase domain containing 14A									
25875	LETMD1	LETM1 domain containing 1									
25894	PLEKHG4	pleckstrin homology domain containing, family G (with RhoGef domain) member 4									
25912	C1orf43	chromosome 1 open reading frame 43									
259266	ASPM	asp (abnormal spindle) homolog, microcephaly associated (Drosophila)									
25934	NIPSNAP3A	nipsnap homolog 3A (C. elegans)									
25956	SEC31B	SEC31 homolog B (S. cerevisiae)									
25957	PNISR	PNN-interacting serine/arginine-rich protein									
25959	KANK2	KN motif and ankyrin repeat domains 2									
26000	TBC1D10B	TBC1 domain family, member 10B									
26009	ZZZ3	zinc finger, ZZ-type containing 3									
26013	L3MBTL1	l(3)mbt-like 1 (Drosophila)									
26065	LSM14A	LSM14A, SCD6 homolog A (S. cerevisiae)									
26140	TTLL3	tubulin tyrosine ligase-like family, member 3									
26233	FBXL6	F-box and leucine-rich repeat protein 6									
26262	TSPAN17	tetraspanin 17									
2630	GBAP1	glucosidase, beta, acid pseudogene 1									
26469	PTPN18	protein tyrosine phosphatase, non-receptor type 18 (brain-derived)									
26586	CKAP2	cytoskeleton associated protein 2									
26973	CHORDC1	cysteine and histidine-rich domain (CHORD) containing 1									
26996	GPR160	G protein-coupled receptor 160									
27037	TRMT2A	TRM2 tRNA methyltransferase 2 homolog A (S. cerevisiae)									
27229	TUBGCP4	tubulin, gamma complex associated protein 4									
27309	ZNF330	zinc finger protein 330									
27314	RAB30	RAB30, member RAS oncogene family									
27315	PGAP2	post-GPI attachment to proteins 2									
27341	RRP7A	ribosomal RNA processing 7 homolog A (S. cerevisiae)									
27352	SGSM3	small G protein signaling modulator 3									
27433	TOR2A	torsin family 2, member A									
2804	GOLGB1	golgin B1									
282809	POC1B	POC1 centriolar protein homolog B (Chlamydomonas)									
283234	CCDC88B	coiled-coil domain containing 88B									
283337	ZNF740	zinc finger protein 740									
283899	INO80E	INO80 complex subunit E									
283989	TSEN54	tRNA splicing endonuclease 54 homolog (S. cerevisiae)									
284001	CCDC57	coiled-coil domain containing 57									
284004	HEXDC	hexosaminidase (glycosyl hydrolase family 20, catalytic domain) containing									
284047	CCDC144B	coiled-coil domain containing 144B (pseudogene)									
284086	NEK8	NIMA (never in mitosis gene a)- related kinase 8									
284565	NBPF15	neuroblastoma breakpoint family, member 15									
284992	CCDC150	coiled-coil domain containing 150									
285025	CCDC141	coiled-coil domain containing 141									
28511	NKIRAS2	NFKB inhibitor interacting Ras-like 2									
285381	DPH3	DPH3, KTI11 homolog (S. cerevisiae)									
285613	RELL2	RELT-like 2									
285636	C5orf51	chromosome 5 open reading frame 51									
285753	CEP57L1	centrosomal protein 57kDa-like 1									
285989	ZNF789	zinc finger protein 789									
286257	C9orf142	chromosome 9 open reading frame 142									
2873	GPS1	G protein pathway suppressor 1									
28957	MRPS28	mitochondrial ribosomal protein S28									
29066	ZC3H7A	zinc finger CCCH-type containing 7A									
29068	ZBTB44	zinc finger and BTB domain containing 44									
29081	METTL5	methyltransferase like 5									
29087	THYN1	thymocyte nuclear protein 1									
29093	MRPL22	mitochondrial ribosomal protein L22									
29098	RANGRF	RAN guanine nucleotide release factor									
29115	SAP30BP	SAP30 binding protein									
29803	REPIN1	replication initiator 1									
29911	HOOK2	hook homolog 2 (Drosophila)									
29952	DPP7	dipeptidyl-peptidase 7									
29954	POMT2	protein-O-mannosyltransferase 2									
29992	PILRA	paired immunoglobin-like type 2 receptor alpha									
3257	HPS1	Hermansky-Pudlak syndrome 1									
327	APEH	N-acylaminoacyl-peptide hydrolase									
3338	DNAJC4	DnaJ (Hsp40) homolog, subfamily C, member 4									
337867	UBAC2	UBA domain containing 2									
338692	ANKRD13D	ankyrin repeat domain 13 family, member D									
339123	JMJD8	jumonji domain containing 8									
339487	ZBTB8OS	zinc finger and BTB domain containing 8 opposite strand									
339804	C2orf74	chromosome 2 open reading frame 74									
343099	CCDC18	coiled-coil domain containing 18									
345778	MTX3	metaxin 3									
346389	MACC1	metastasis associated in colon cancer 1									
347862	PDDC1	Parkinson disease 7 domain containing 1									
353511	PKD1P6	polycystic kidney disease 1 (autosomal dominant) pseudogene 6									
374395	TMEM179B	transmembrane protein 179B									
374403	TBC1D10C	TBC1 domain family, member 10C									
374666	WASH3P	WAS protein family homolog 3 pseudogene									
374986	FAM73A	family with sequence similarity 73, member A									
375248	ANKRD36	ankyrin repeat domain 36									
378938	MALAT1	metastasis associated lung adenocarcinoma transcript 1 (non-protein coding)									
386593	CHKB-CPT1B	CHKB-CPT1B readthrough (non-protein coding)									
387921	NHLRC3	NHL repeat containing 3									
388561	ZNF761	zinc finger protein 761									
399909	PCNXL3	pecanex-like 3 (Drosophila)									
399979	SNX19	sorting nexin 19									
400954	EML6	echinoderm microtubule associated protein like 6									
401262	CRIP3	cysteine-rich protein 3									
4034	LRCH4	leucine-rich repeats and calponin homology (CH) domain containing 4									
4152	MBD1	methyl-CpG binding domain protein 1									
4162	MCAM	melanoma cell adhesion molecule									
440193	CCDC88C	coiled-coil domain containing 88C									
440400	RNASEK	ribonuclease, RNase K									
4648	MYO7B	myosin VIIB									
4649	MYO9A	myosin IXA									
473	RERE	arginine-glutamic acid dipeptide (RE) repeats									
4798	NFRKB	nuclear factor related to kappaB binding protein									
4817	NIT1	nitrilase 1									
4898	NRD1	nardilysin (N-arginine dibasic convertase)									
497661	C18orf32	chromosome 18 open reading frame 32									
50813	COPS7A	COP9 constitutive photomorphogenic homolog subunit 7A (Arabidopsis)									
5090	PBX3	pre-B-cell leukemia homeobox 3									
51000	SLC35B3	solute carrier family 35, member B3									
51088	KLHL5	kelch-like 5 (Drosophila)									
51095	TRNT1	tRNA nucleotidyl transferase, CCA-adding, 1									
51237	MZB1	marginal zone B and B1 cell-specific protein									
51256	TBC1D7	TBC1 domain family, member 7									
51281	ANKMY1	ankyrin repeat and MYND domain containing 1									
51293	CD320	CD320 molecule									
51303	FKBP11	FK506 binding protein 11, 19 kDa									
51319	RSRC1	arginine/serine-rich coiled-coil 1									
51322	WAC	WW domain containing adaptor with coiled-coil									
51390	AIG1	androgen-induced 1									
51397	COMMD10	COMM domain containing 10									
51427	ZNF107	zinc finger protein 107									
51574	LARP7	La ribonucleoprotein domain family, member 7									
51601	LIPT1	lipoyltransferase 1									
51605	TRMT6	tRNA methyltransferase 6 homolog (S. cerevisiae)									
51629	SLC25A39	solute carrier family 25, member 39									
51654	CDK5RAP1	CDK5 regulatory subunit associated protein 1									
51693	TRAPPC2L	trafficking protein particle complex 2-like									
51714	SELT	selenoprotein T									
5252	PHF1	PHD finger protein 1									
5379	PMS2P1	postmeiotic segregation increased 2 pseudogene 1									
53827	FXYD5	FXYD domain containing ion transport regulator 5									
5383	PMS2P5	postmeiotic segregation increased 2 pseudogene 5									
53917	RAB24	RAB24, member RAS oncogene family									
53938	PPIL3	peptidylprolyl isomerase (cyclophilin)-like 3									
5428	POLG	polymerase (DNA directed), gamma									
54476	RNF216	ring finger protein 216									
54496	PRMT7	protein arginine methyltransferase 7									
5450	POU2AF1	POU class 2 associating factor 1									
54516	MTRF1L	mitochondrial translational release factor 1-like									
54542	RC3H2	ring finger and CCCH-type domains 2									
54585	LZTFL1	leucine zipper transcription factor-like 1									
54620	FBXL19	F-box and leucine-rich repeat protein 19									
54664	TMEM106B	transmembrane protein 106B									
54676	GTPBP2	GTP binding protein 2									
54758	KLHDC4	kelch domain containing 4									
54788	DNAJB12	DnaJ (Hsp40) homolog, subfamily B, member 12									
54793	KCTD9	potassium channel tetramerisation domain containing 9									
54808	DYM	dymeclin									
54814	QPCTL	glutaminyl-peptide cyclotransferase-like									
54816	ZNF280D	zinc finger protein 280D									
54849	DEF8	differentially expressed in FDCP 8 homolog (mouse)									
54850	FBXL12	F-box and leucine-rich repeat protein 12									
54851	ANKRD49	ankyrin repeat domain 49									
54853	WDR55	WD repeat domain 55									
54867	TMEM214	transmembrane protein 214									
54876	DCAF16	DDB1 and CUL4 associated factor 16									
54883	CWC25	CWC25 spliceosome-associated protein homolog (S. cerevisiae)									
54891	INO80D	INO80 complex subunit D									
54902	TTC19	tetratricopeptide repeat domain 19									
54930	HAUS4	HAUS augmin-like complex, subunit 4									
54939	COMMD4	COMM domain containing 4									
54940	OCIAD1	OCIA domain containing 1									
54976	C20orf27	chromosome 20 open reading frame 27									
54985	HCFC1R1	host cell factor C1 regulator 1 (XPO1 dependent)									
54986	ULK4	unc-51-like kinase 4 (C. elegans)									
54991	C1orf159	chromosome 1 open reading frame 159									
55000	TUG1	taurine upregulated 1 (non-protein coding)									
55002	TMCO3	transmembrane and coiled-coil domains 3									
55006	TRMT61B	tRNA methyltransferase 61 homolog B (S. cerevisiae)									
55015	PRPF39	PRP39 pre-mRNA processing factor 39 homolog (S. cerevisiae)									
55041	PLEKHB2	pleckstrin homology domain containing, family B (evectins) member 2									
55074	OXR1	oxidation resistance 1									
55084	SOBP	sine oculis binding protein homolog (Drosophila)									
55101	ATP5SL	ATP5S-like									
55103	RALGPS2	Ral GEF with PH domain and SH3 binding motif 2									
55131	RBM28	RNA binding motif protein 28									
55151	TMEM38B	transmembrane protein 38B									
55152	DALRD3	DALR anticodon binding domain containing 3									
55159	RFWD3	ring finger and WD repeat domain 3									
55161	TMEM33	transmembrane protein 33									
55168	MRPS18A	mitochondrial ribosomal protein S18A									
55173	MRPS10	mitochondrial ribosomal protein S10									
55174	INTS10	integrator complex subunit 10									
55186	SLC25A36	solute carrier family 25, member 36									
55187	VPS13D	vacuolar protein sorting 13 homolog D (S. cerevisiae)									
55198	APPL2	adaptor protein, phosphotyrosine interaction, PH domain and leucine zipper containing 2									
55209	SETD5	SET domain containing 5									
55227	LRRC1	leucine rich repeat containing 1									
55251	PCMTD2	protein-L-isoaspartate (D-aspartate) O-methyltransferase domain containing 2									
55278	QRSL1	glutaminyl-tRNA synthase (glutamine-hydrolyzing)-like 1									
55280	CWF19L1	CWF19-like 1, cell cycle control (S. pombe)									
55285	RBM41	RNA binding motif protein 41									
55291	PPP6R3	protein phosphatase 6, regulatory subunit 3									
55297	CCDC91	coiled-coil domain containing 91									
55298	RNF121	ring finger protein 121									
55324	ABCF3	ATP-binding cassette, sub-family F (GCN20), member 3									
55337	C19orf66	chromosome 19 open reading frame 66									
55374	TMCO6	transmembrane and coiled-coil domains 6									
55486	PARL	presenilin associated, rhomboid-like									
55591	VEZT	vezatin, adherens junctions transmembrane protein									
55608	ANKRD10	ankyrin repeat domain 10									
55617	TASP1	taspase, threonine aspartase, 1									
55621	TRMT1	TRM1 tRNA methyltransferase 1 homolog (S. cerevisiae)									
55653	BCAS4	breast carcinoma amplified sequence 4									
55656	INTS8	integrator complex subunit 8									
55657	ZNF692	zinc finger protein 692									
55658	RNF126	ring finger protein 126									
55692	LUC7L	LUC7-like (S. cerevisiae)									
55720	TSR1	TSR1, 20S rRNA accumulation, homolog (S. cerevisiae)									
55728	N4BP2	NEDD4 binding protein 2									
55735	DNAJC11	DnaJ (Hsp40) homolog, subfamily C, member 11									
55812	SPATA7	spermatogenesis associated 7									
55823	VPS11	vacuolar protein sorting 11 homolog (S. cerevisiae)									
55827	DCAF6	DDB1 and CUL4 associated factor 6									
55846	ITFG2	integrin alpha FG-GAP repeat containing 2									
55871	CBWD1	COBW domain containing 1									
55892	MYNN	myoneurin									
55974	SLC50A1	solute carrier family 50 (sugar transporter), member 1									
56063	TMEM234	transmembrane protein 234									
5612	PRKRIR	protein-kinase, interferon-inducible double stranded RNA dependent inhibitor, repressor of (P58 repressor)									
5636	PRPSAP2	phosphoribosyl pyrophosphate synthetase-associated protein 2									
56681	SAR1A	SAR1 homolog A (S. cerevisiae)									
56834	GPR137	G protein-coupled receptor 137									
56850	GRIPAP1	GRIP1 associated protein 1									
56890	MDM1	Mdm1 nuclear protein homolog (mouse)									
56940	DUSP22	dual specificity phosphatase 22									
56943	ENY2	enhancer of yellow 2 homolog (Drosophila)									
56946	C11orf30	chromosome 11 open reading frame 30									
56947	MFF	mitochondrial fission factor									
56948	SDR39U1	short chain dehydrogenase/reductase family 39U, member 1									
56984	PSMG2	proteasome (prosome, macropain) assembly chaperone 2									
56996	SLC12A9	solute carrier family 12 (potassium/chloride transporters), member 9									
57140	RNPEPL1	arginyl aminopeptidase (aminopeptidase B)-like 1									
57396	CLK4	CDC-like kinase 4									
57409	MIF4GD	MIF4G domain containing									
57410	SCYL1	SCY1-like 1 (S. cerevisiae)									
57418	WDR18	WD repeat domain 18									
57494	RIMKLB	ribosomal modification protein rimK-like family member B									
57507	ZNF608	zinc finger protein 608									
57519	STARD9	StAR-related lipid transfer (START) domain containing 9									
57533	TBC1D14	TBC1 domain family, member 14									
57544	TXNDC16	thioredoxin domain containing 16									
57614	KIAA1468	KIAA1468									
57649	PHF12	PHD finger protein 12									
57664	PLEKHA4	pleckstrin homology domain containing, family A (phosphoinositide binding specific) member 4									
57699	CPNE5	copine V									
57730	ANKRD36B	ankyrin repeat domain 36B									
57786	RBAK	RB-associated KRAB zinc finger									
5802	PTPRS	protein tyrosine phosphatase, receptor type, S									
58487	CREBZF	CREB/ATF bZIP transcription factor									
58516	FAM60A	family with sequence similarity 60, member A									
5863	RGL2	ral guanine nucleotide dissociation stimulator-like 2									

5965	RECQL	RecQ protein-like (DNA helicase Q1-like)										
6003	RGS13	regulator of G-protein signaling 13										
60343	FAM3A	family with sequence similarity 3, member A										
60468	BACH2	BTB and CNC homology 1, basic leucine zipper transcription factor 2										
60674	GAS5	growth arrest-specific 5 (non-protein coding)										
6314	ATXN7	ataxin 7										
636	BICD1	bicaudal D homolog 1 (Drosophila)										
63892	THADA	thyroid adenoma associated										
63901	FAM111A	family with sequence similarity 111, member A										
63922	CHTF18	CTF18, chromosome transmission fidelity factor 18 homolog (S. cerevisiae)										
63941	NECAB3	N-terminal EF-hand calcium binding protein 3										
64005	MYO1G	myosin IG										
64118	DUS1L	dihydrouridine synthase 1-like (S. cerevisiae)										
641638	SNHG6	small nucleolar RNA host gene 6 (non-protein coding)										
641649	TMEM91	transmembrane protein 91										
64210	MMS19	MMS19 nucleotide excision repair homolog (S. cerevisiae)										
64236	PDLIM2	PDZ and LIM domain 2 (mystique)										
64319	FBRS	fibrosin										
64328	XPO4	exportin 4										
644128	RPL23AP53	ribosomal protein L23a pseudogene 53										
64418	TMEM168	transmembrane protein 168										
64432	MRPS25	mitochondrial ribosomal protein S25										
64478	CSMD1	CUB and Sushi multiple domains 1										
645027	EVPLL	envoplakin-like										
645784	ANKRD36BP2	ankyrin repeat domain 36B pseudogene 2										
64595	TTTY15	testis-specific transcript, Y-linked 15 (non-protein coding)										
64755	C16orf58	chromosome 16 open reading frame 58										
64780	MICAL1	microtubule associated monoxygenase, calponin and LIM domain containing 1										
64783	RBM15	RNA binding motif protein 15										
64927	TTC23	tetratricopeptide repeat domain 23										
64940	STAG3L4	stromal antigen 3-like 4										
65009	NDRG4	NDRG family member 4										
65056	GPBP1	GC-rich promoter binding protein 1										
65084	TMEM135	transmembrane protein 135										
65095	KRI1	KRI1 homolog (S. cerevisiae)										
651746	ANKRD33B	ankyrin repeat domain 33B										
65268	WNK2	WNK lysine deficient protein kinase 2										
6596	HLTF	helicase-like transcription factor										
65981	CAPRIN2	caprin family member 2										
66036	MTMR9	myotubularin related protein 9										
664701	ZNF826P	zinc finger protein 826, pseudogene										
6835	SURF2	surfeit 2										
6871	TADA2A	transcriptional adaptor 2A										
6919	TCEA2	transcription elongation factor A (SII), 2										
7036	TFR2	transferrin receptor 2										
7072	TIA1	TIA1 cytotoxic granule-associated RNA binding protein										
724102	SNHG4	small nucleolar RNA host gene 4 (non-protein coding)										
7265	TTC1	tetratricopeptide repeat domain 1										
728411	GUSBP1	glucuronidase, beta pseudogene 1										
7375	USP4	ubiquitin specific peptidase 4 (proto-oncogene)										
7444	VRK2	vaccinia related kinase 2										
752	FMNL1	formin-like 1										
7536	SF1	splicing factor 1										
7589	ZSCAN21	zinc finger and SCAN domain containing 21										
7597	ZBTB25	zinc finger and BTB domain containing 25										
7643	ZNF90	zinc finger protein 90										
7748	ZNF195	zinc finger protein 195										
7871	SLMAP	sarcolemma associated protein										
78996	C7orf49	chromosome 7 open reading frame 49										
79006	METRN	meteorin, glial cell differentiation regulator										
79022	TMEM106C	transmembrane protein 106C										
79078	C1orf50	chromosome 1 open reading frame 50										
79089	TMUB2	transmembrane and ubiquitin-like domain containing 2										
79090	TRAPPC6A	trafficking protein particle complex 6A										
79091	METTL22	methyltransferase like 22										
79137	FAM134A	family with sequence similarity 134, member A										
79144	PPDPF	pancreatic progenitor cell differentiation and proliferation factor homolog (zebrafish)										
7917	BAG6	BCL2-associated athanogene 6										
79180	EFHD2	EF-hand domain family, member D2										
79228	THOC6	THO complex 6 homolog (Drosophila)										
79363	RSG1	REM2 and RAB-like small GTPase 1										
79368	FCRL2	Fc receptor-like 2										
79441	HAUS3	HAUS augmin-like complex, subunit 3										
79568	C2orf47	chromosome 2 open reading frame 47										
79572	ATP13A3	ATPase type 13A3										
79573	TTC13	tetratricopeptide repeat domain 13										
79612	NAA16	N(alpha)-acetyltransferase 16, NatA auxiliary subunit										
79639	TMEM53	transmembrane protein 53										
79641	ROGDI	rogdi homolog (Drosophila)										
79668	PARP8	poly (ADP-ribose) polymerase family, member 8										
79706	PRKRIP1	PRKR interacting protein 1 (IL11 inducible)										
79707	NOL9	nucleolar protein 9										
79725	THAP9	THAP domain containing 9										
79734	KCTD17	potassium channel tetramerisation domain containing 17										
79763	ISOC2	isochorismatase domain containing 2										
79772	MCTP1	multiple C2 domains, transmembrane 1										
79777	ACBD4	acyl-CoA binding domain containing 4										
79794	C12orf49	chromosome 12 open reading frame 49										
79844	ZDHHC11	zinc finger, DHHC-type containing 11										
79856	SNX22	sorting nexin 22										
79892	MCMBP	minichromosome maintenance complex binding protein										
79897	RPP21	ribonuclease P/MRP 21kDa subunit										
79930	DOK3	docking protein 3										
79934	ADCK4	aarF domain containing kinase 4										
79960	PHF17	PHD finger protein 17										
79968	WDR76	WD repeat domain 76										
79969	ATAT1	alpha tubulin acetyltransferase 1										
79979	TRMT2B	TRM2 tRNA methyltransferase 2 homolog B (S. cerevisiae)										
80008	TMEM156	transmembrane protein 156										
80011	FAM192A	family with sequence similarity 192, member A										
80017	C14orf159	chromosome 14 open reading frame 159										
80021	TMEM62	transmembrane protein 62										
80034	CSRNP3	cysteine-serine-rich nuclear protein 3										
80063	ATF7IP2	activating transcription factor 7 interacting protein 2										
80067	DCAF17	DDB1 and CUL4 associated factor 17										
80179	MYO19	myosin XIX										
8019	BRD3	bromodomain containing 3										
80208	SPG11	spastic paraplegia 11 (autosomal recessive)										
80210	ARMC9	armadillo repeat containing 9										
80213	TM2D3	TM2 domain containing 3										
80237	ELL3	elongation factor RNA polymerase II-like 3										
80279	CDK5RAP3	CDK5 regulatory subunit associated protein 3										
80305	TRABD	TraB domain containing										
80336	PABPC1L	poly(A) binding protein, cytoplasmic 1-like										
80774	LIMD2	LIM domain containing 2										
80821	DDHD1	DDHD domain containing 1										
8131	NPRL3	nitrogen permease regulator-like 3 (S. cerevisiae)										
81532	MOB2	MOB kinase activator 2										
81609	SNX27	sorting nexin family member 27										
81614	NIPA2	non imprinted in Prader-Willi/Angelman syndrome 2										
81790	RNF170	ring finger protein 170										
81831	NETO2	neuropilin (NRP) and tolloid (TLL)-like 2										
81847	RNF146	ring finger protein 146										
81858	SHARPIN	SHANK-associated RH domain interactor										
81890	QTRT1	queuine tRNA-ribosyltransferase 1										
8216	LZTR1	leucine-zipper-like transcription regulator 1										
8284	KDM5D	lysine (K)-specific demethylase 5D										
8287	USP9Y	ubiquitin specific peptidase 9, Y-linked										
83440	ADPGK	ADP-dependent glucokinase										
83448	PUS7L	pseudouridylate synthase 7 homolog (S. cerevisiae)-like										
83451	ABHD11	abhydrolase domain containing 11										
83479	DDX59	DEAD (Asp-Glu-Ala-Asp) box polypeptide 59										
83596	BCL2L12	BCL2-like 12 (proline rich)										
83706	FERMT3	fermitin family member 3										
83707	TRPT1	tRNA phosphotransferase 1										
83719	YPEL3	yippee-like 3 (Drosophila)										
83746	L3MBTL2	l(3)mbt-like 2 (Drosophila)										
83759	RBM4B	RNA binding motif protein 4B										
83862	TMEM120A	transmembrane protein 120A										
84064	HDHD2	haloacid dehalogenase-like hydrolase domain containing 2										
84081	NSRP1	nuclear speckle splicing regulatory protein 1										
84129	ACAD11	acyl-CoA dehydrogenase family, member 11										
84164	ASCC2	activating signal cointegrator 1 complex subunit 2										
84167	C19orf44	chromosome 19 open reading frame 44										
84193	SETD3	SET domain containing 3										
84232	MAF1	MAF1 homolog (S. cerevisiae)										
84266	ALKBH7	alkB, alkylation repair homolog 7 (E. coli)										
84268	RPAIN	RPA interacting protein										
84304	NUDT22	nudix (nucleoside diphosphate linked moiety X)-type motif 22										
84307	ZNF397	zinc finger protein 397										
84314	TMEM107	transmembrane protein 107										
84326	C16orf13	chromosome 16 open reading frame 13										
84456	L3MBTL3	l(3)mbt-like 3 (Drosophila)										
84668	FAM126A	family with sequence similarity 126, member A										
84705	GTPBP3	GTP binding protein 3 (mitochondrial)										
84722	PSRC1	proline/serine-rich coiled-coil 1										
84726	PRRC2B	proline-rich coiled-coil 2B										
84749	USP30	ubiquitin specific peptidase 30										
84824	FCRLA	Fc receptor-like A										
84859	LRCH3	leucine-rich repeats and calponin homology (CH) domain containing 3										
84899	TMTC4	transmembrane and tetratricopeptide repeat containing 4										
84901	NFATC2IP	nuclear factor of activated T-cells, cytoplasmic, calcineurin-dependent 2 interacting protein										
84922	FIZ1	FLT3-interacting zinc finger 1										
84954	MPND	MPN domain containing										
84958	SYTL1	synaptotagmin-like 1										
84961	FBXL20	F-box and leucine-rich repeat protein 20										
84993	UBL7	ubiquitin-like 7 (bone marrow stromal cell-derived)										
85014	TMEM141	transmembrane protein 141										
85015	USP45	ubiquitin specific peptidase 45										
8502	PKP4	plakophilin 4										
85028	SNHG12	small nucleolar RNA host gene 12 (non-protein coding)										
8533	COPS3	COP9 constitutive photomorphogenic homolog subunit 3 (Arabidopsis)										
85444	LRRCC1	leucine rich repeat and coiled-coil domain containing 1										
8548	BLZF1	basic leucine zipper nuclear factor 1										
8576	STK16	serine/threonine kinase 16										
8635	RNASET2	ribonuclease T2										
8798	DYRK4	dual-specificity tyrosine-(Y)-phosphorylation regulated kinase 4										
8847	DLEU2	deleted in lymphocytic leukemia 2 (non-protein coding)										
8859	STK19	serine/threonine kinase 19										
889	KRIT1	KRIT1, ankyrin repeat containing										
8904	CPNE1	copine I										
8906	AP1G2	adaptor-related protein complex 1, gamma 2 subunit										
8975	USP13	ubiquitin specific peptidase 13 (isopeptidase T-3)										
89781	HPS4	Hermansky-Pudlak syndrome 4										
89782	LMLN	leishmanolysin-like (metallopeptidase M8 family)										
89790	SIGLEC10	sialic acid binding Ig-like lectin 10										
89848	FCHSD1	FCH and double SH3 domains 1										
89894	TMEM116	transmembrane protein 116										
90326	THAP3	THAP domain containing, apoptosis associated protein 3										
90557	CCDC74A	coiled-coil domain containing 74A										
90624	LYRM7	Lyrm7 homolog (mouse)										
90809	TMEM55B	transmembrane protein 55B										
90864	SPSB3	splA/ryanodine receptor domain and SOCS box containing 3										
9100	USP10	ubiquitin specific peptidase 10										
91319	DERL3	Der1-like domain family, member 3										
9147	NEMF	nuclear export mediator factor										
91544	UBXN11	UBX domain protein 11										
91582	RPS19BP1	ribosomal protein S19 binding protein 1										
9159	PCSK7	proprotein convertase subtilisin/kexin type 7										
9169	SCAF11	SR-related CTD-associated factor 11										
91801	ALKBH8	alkB, alkylation repair homolog 8 (E. coli)										
9205	ZMYM5	zinc finger, MYM-type 5										
92170	MTG1	mitochondrial GTPase 1 homolog (S. cerevisiae)										
92270	ATP6AP1L	ATPase, H+ transporting, lysosomal accessory protein 1-like										
92400	RBM18	RNA binding motif protein 18										
92482	BBIP1	BBSome interacting protein 1										
9262	STK17B	serine/threonine kinase 17b										
92714	ARRDC1	arrestin domain containing 1										
9274	BCL7C	B-cell CLL/lymphoma 7C										
9275	BCL7B	B-cell CLL/lymphoma 7B										
92799	SHKBP1	SH3KBP1 binding protein 1										
9317	PTER	phosphotriesterase related										
9324	HMGN3	high mobility group nucleosomal binding domain 3										
9326	ZNHIT3	zinc finger, HIT-type containing 3										
93627	TBCK	TBC1 domain containing kinase										
9364	RAB28	RAB28, member RAS oncogene family										
9399	STOML1	stomatin (EPB72)-like 1										
9404	LPXN	leupaxin										
9526	MPDU1	mannose-P-dolichol utilization defect 1										
9553	MRPL33	mitochondrial ribosomal protein L33										
9556	C14orf2	chromosome 14 open reading frame 2										
9567	GTPBP1	GTP binding protein 1										
9584	RBM39	RNA binding motif protein 39										
963	CD53	CD53 molecule										
9637	FEZ2	fasciculation and elongation protein zeta 2 (zygin II)										
9643	MORF4L2	mortality factor 4 like 2										
9648	GCC2	GRIP and coiled-coil domain containing 2										
9649	RALGPS1	Ral GEF with PH domain and SH3 binding motif 1										
9678	PHF14	PHD finger protein 14										
9679	FAM53B	family with sequence similarity 53, member B										
9684	LRRC14	leucine rich repeat containing 14										
9701	PPP6R2	protein phosphatase 6, regulatory subunit 2										
9731	CEP104	centrosomal protein 104kDa										
9733	SART3	squamous cell carcinoma antigen recognized by T cells 3										
9772	KIAA0195	KIAA0195										
9779	TBC1D5	TBC1 domain family, member 5										
9786	KIAA0586	KIAA0586										
9812	KIAA0141	KIAA0141										
9814	SFI1	Sfi1 homolog, spindle assembly associated (yeast)										
9819	TSC22D2	TSC22 domain family, member 2										
9854	C2CD2L	C2CD2-like										
987	LRBA	LPS-responsive vesicle trafficking, beach and anchor containing										
9875	URB1	URB1 ribosome biogenesis 1 homolog (S. cerevisiae)										
9877	ZC3H11A	zinc finger CCCH-type containing 11A										
9894	TELO2	TEL2, telomere maintenance 2, homolog (S. cerevisiae)										
9898	UBAP2L	ubiquitin associated protein 2-like										
9905	SGSM2	small G protein signaling modulator 2										
9909	DENND4B	DENN/MADD domain containing 4B										

Supplementary Table 11: REC-1 Alternative Splicing MsigDB Analysis

Overlap Results						
Collection(s):	CP, CP:BIOCARTA, CP:KEGG, CP:REACTOME, H					
# overlaps shown:	10					
# genesets in collections:	1379					
# genes in comparison (n):	327					
# genes in universe (N):	45956					
Gene Set Name	# Genes in Gene Set (K)	Description	# Genes in Overlap (k)	k/K	p-value	FDR q-value
REACTOME_IMMUNE_SYSTEM	933	Genes involved in Immune System	23	0.0247	3.25E-07	2.08E-04
REACTOME_SIGNALING_BY_THE_B_CELL_RECEPTOR_BCR	126	Genes involved in Signaling by the B Cell Receptor (BCR)	9	0.0714	3.33E-07	2.08E-04
REACTOME_CELL_CYCLE	421	Genes involved in Cell Cycle	15	0.0356	4.53E-07	2.08E-04
REACTOME_SIGNALING_BY_NOTCH	103	Genes involved in Signaling by NOTCH	8	0.0777	7.98E-07	2.52E-04
REACTOME_NOTCH1_INTRACELLULAR_DOMAIN_REGULATES_TRANSCRIPTION	46	Genes involved in NOTCH1 Intracellular Domain Regulates Transcription	6	0.1304	9.14E-07	2.52E-04
REACTOME_CELL_CYCLE_MITOTIC	325	Genes involved in Cell Cycle, Mitotic	12	0.0369	4.48E-06	9.50E-04
BIOCARTA_CARM_ER_PATHWAY	35	CARM1 and Regulation of the Estrogen Receptor	5	0.1429	4.82E-06	9.50E-04
REACTOME_METABOLISM_OF_PROTEINS	518	Genes involved in Metabolism of proteins	15	0.029	5.75E-06	9.91E-04
PID_P53_DOWNSTREAM_PATHWAY	137	Direct p53 effectors	8	0.0584	6.80E-06	1.04E-03
REACTOME_ADAPTIVE_IMMUNE_SYSTEM	539	Genes involved in Adaptive Immune System	15	0.0278	9.21E-06	1.27E-03

Gene/Gene Set Overlap Matrix

Entrez Gene Id	Gene Symbol	Gene Description	REACTOME_IMMUNE_SYSTEM	REACTOME_SIGNALING_BY_THE_B_CELL_RECEPTOR_BCR	REACTOME_CELL_CYCLE	REACTOME_SIGNALING_BY_NOTCH	REACTOME_NOTCH1_INTRACELLULAR_DOMAIN_REGULATES_TRANSCRIPTION	REACTOME_CELL_CYCLE_MITOTIC	BIOCARTA_CARM_ER_PATHWAY	REACTOME_METABOLISM_OF_PROTEINS	PID_P53_DOWNSTREAM_PATHWAY	REACTOME_ADAPTIVE_IMMUNE_SYSTEM
7311	UBA52	ubiquitin A-52 residue ribosomal protein fusion product 1	REACTOME_IMMUNE_SYSTEM	REACTOME_SIGNALING_BY_THE_B_CELL_RECEPTOR_BCR	REACTOME_CELL_CYCLE	REACTOME_SIGNALING_BY_NOTCH	REACTOME_NOTCH1_INTRACELLULAR_DOMAIN_REGULATES_TRANSCRIPTION	REACTOME_CELL_CYCLE_MITOTIC		REACTOME_METABOLISM_OF_PROTEINS		REACTOME_ADAPTIVE_IMMUNE_SYSTEM
5706	PSMC6	proteasome (prosome, macropain) 26S subunit, ATPase, 6	REACTOME_IMMUNE_SYSTEM	REACTOME_SIGNALING_BY_THE_B_CELL_RECEPTOR_BCR	REACTOME_CELL_CYCLE			REACTOME_CELL_CYCLE_MITOTIC				REACTOME_ADAPTIVE_IMMUNE_SYSTEM
5682	PSMA1	proteasome (prosome, macropain) subunit, alpha type, 1	REACTOME_IMMUNE_SYSTEM	REACTOME_SIGNALING_BY_THE_B_CELL_RECEPTOR_BCR	REACTOME_CELL_CYCLE			REACTOME_CELL_CYCLE_MITOTIC				REACTOME_ADAPTIVE_IMMUNE_SYSTEM
8945	BTRC	beta-transducin repeat containing	REACTOME_IMMUNE_SYSTEM	REACTOME_SIGNALING_BY_THE_B_CELL_RECEPTOR_BCR	REACTOME_CELL_CYCLE			REACTOME_CELL_CYCLE_MITOTIC				REACTOME_ADAPTIVE_IMMUNE_SYSTEM
5728	PTEN	phosphatase and tensin homolog	REACTOME_IMMUNE_SYSTEM	REACTOME_SIGNALING_BY_THE_B_CELL_RECEPTOR_BCR							PID_P53_DOWNSTREAM_PATHWAY	REACTOME_ADAPTIVE_IMMUNE_SYSTEM
842	CASP9	caspase 9, apoptosis-related cysteine peptidase	REACTOME_IMMUNE_SYSTEM	REACTOME_SIGNALING_BY_THE_B_CELL_RECEPTOR_BCR								REACTOME_ADAPTIVE_IMMUNE_SYSTEM
4690	NCK1	NCK adaptor protein 1	REACTOME_IMMUNE_SYSTEM	REACTOME_SIGNALING_BY_THE_B_CELL_RECEPTOR_BCR								REACTOME_ADAPTIVE_IMMUNE_SYSTEM
974	CD79B	CD79b molecule, immunoglobulin-associated beta	REACTOME_IMMUNE_SYSTEM	REACTOME_SIGNALING_BY_THE_B_CELL_RECEPTOR_BCR								REACTOME_ADAPTIVE_IMMUNE_SYSTEM
118788	PIK3AP1	phosphoinositide-3-kinase adaptor protein 1	REACTOME_IMMUNE_SYSTEM	REACTOME_SIGNALING_BY_THE_B_CELL_RECEPTOR_BCR								REACTOME_ADAPTIVE_IMMUNE_SYSTEM
79902	NUP85	nucleoporin 85kDa	REACTOME_IMMUNE_SYSTEM		REACTOME_CELL_CYCLE			REACTOME_CELL_CYCLE_MITOTIC				
25898	RCHY1	ring finger and CHY zinc finger domain containing 1	REACTOME_IMMUNE_SYSTEM								PID_P53_DOWNSTREAM_PATHWAY	REACTOME_ADAPTIVE_IMMUNE_SYSTEM
58484	NLRC4	NLR family, CARD domain containing 4	REACTOME_IMMUNE_SYSTEM								PID_P53_DOWNSTREAM_PATHWAY	
29127	RACGAP1	Rac GTPase activating protein 1	REACTOME_IMMUNE_SYSTEM									REACTOME_ADAPTIVE_IMMUNE_SYSTEM
7326	UBE2G1	ubiquitin-conjugating enzyme E2G 1	REACTOME_IMMUNE_SYSTEM									REACTOME_ADAPTIVE_IMMUNE_SYSTEM
140739	UBE2F	ubiquitin-conjugating enzyme E2F (putative)	REACTOME_IMMUNE_SYSTEM									REACTOME_ADAPTIVE_IMMUNE_SYSTEM
93611	FBXO44	F-box protein 44	REACTOME_IMMUNE_SYSTEM									REACTOME_ADAPTIVE_IMMUNE_SYSTEM
3385	ICAM3	intercellular adhesion molecule 3	REACTOME_IMMUNE_SYSTEM									REACTOME_ADAPTIVE_IMMUNE_SYSTEM
3326	HSP90AB1	heat shock protein 90kDa alpha (cytosolic), class B member 1	REACTOME_IMMUNE_SYSTEM									
3656	IRAK2	interleukin-1 receptor-associated kinase 2	REACTOME_IMMUNE_SYSTEM									
9883	POM121	POM121 membrane glycoprotein	REACTOME_IMMUNE_SYSTEM									
8672	EIF4G3	eukaryotic translation initiation factor 4 gamma, 3	REACTOME_IMMUNE_SYSTEM									
5094	PCBP2	poly(rC) binding protein 2	REACTOME_IMMUNE_SYSTEM									
10695	CNPY3	canopy 3 homolog (zebrafish)	REACTOME_IMMUNE_SYSTEM									
3065	HDAC1	histone deacetylase 1			REACTOME_CELL_CYCLE	REACTOME_SIGNALING_BY_NOTCH	REACTOME_NOTCH1_INTRACELLULAR_DOMAIN_REGULATES_TRANSCRIPTION	REACTOME_CELL_CYCLE_MITOTIC	BIOCARTA_CARM_ER_PATHWAY			
7277	TUBA4A	tubulin, alpha 4a			REACTOME_CELL_CYCLE			REACTOME_CELL_CYCLE_MITOTIC		REACTOME_METABOLISM_OF_PROTEINS		
5111	PCNA	proliferating cell nuclear antigen			REACTOME_CELL_CYCLE			REACTOME_CELL_CYCLE_MITOTIC			PID_P53_DOWNSTREAM_PATHWAY	
5558	PRIM2	primase, DNA, polypeptide 2 (58kDa)			REACTOME_CELL_CYCLE			REACTOME_CELL_CYCLE_MITOTIC				
10733	PLK4	polo-like kinase 4			REACTOME_CELL_CYCLE			REACTOME_CELL_CYCLE_MITOTIC				
5985	RFC5	replication factor C (activator 1) 5, 36.5kDa			REACTOME_CELL_CYCLE			REACTOME_CELL_CYCLE_MITOTIC				
286826	LIN9	lin-9 homolog (C. elegans)			REACTOME_CELL_CYCLE			REACTOME_CELL_CYCLE_MITOTIC				
672	BRCA1	breast cancer 1, early onset			REACTOME_CELL_CYCLE				BIOCARTA_CARM_ER_PATHWAY			
64326	RFWD2	ring finger and WD repeat domain 2			REACTOME_CELL_CYCLE						PID_P53_DOWNSTREAM_PATHWAY	
5884	RAD17	RAD17 homolog (S. pombe)			REACTOME_CELL_CYCLE							
9734	HDAC9	histone deacetylase 9				REACTOME_SIGNALING_BY_NOTCH	REACTOME_NOTCH1_INTRACELLULAR_DOMAIN_REGULATES_TRANSCRIPTION		BIOCARTA_CARM_ER_PATHWAY			
83933	HDAC10	histone deacetylase 10				REACTOME_SIGNALING_BY_NOTCH	REACTOME_NOTCH1_INTRACELLULAR_DOMAIN_REGULATES_TRANSCRIPTION		BIOCARTA_CARM_ER_PATHWAY			
7088	TLE1	transducin-like enhancer of split 1 (E(sp1) homolog, Drosophila)				REACTOME_SIGNALING_BY_NOTCH	REACTOME_NOTCH1_INTRACELLULAR_DOMAIN_REGULATES_TRANSCRIPTION					
79718	TBL1XR1	transducin (beta)-like 1 X-linked receptor 1				REACTOME_SIGNALING_BY_NOTCH	REACTOME_NOTCH1_INTRACELLULAR_DOMAIN_REGULATES_TRANSCRIPTION					
10402	ST3GAL6	ST3 beta-galactoside alpha-2,3-sialyltransferase 6				REACTOME_SIGNALING_BY_NOTCH						
5870	RAB6A	RAB6A, member RAS oncogene family				REACTOME_SIGNALING_BY_NOTCH						
11331	PHB2	prohibitin 2							BIOCARTA_CARM_ER_PATHWAY			
1933	EEF1B2	eukaryotic translation elongation factor 1 beta 2								REACTOME_METABOLISM_OF_PROTEINS		
9349	RPL23	ribosomal protein L23								REACTOME_METABOLISM_OF_PROTEINS		
6207	RPS13	ribosomal protein S13								REACTOME_METABOLISM_OF_PROTEINS		
6139	RPL17	ribosomal protein L17								REACTOME_METABOLISM_OF_PROTEINS		
8666	EIF3G	eukaryotic translation initiation factor 3, subunit G								REACTOME_METABOLISM_OF_PROTEINS		
28972	SPCS1	signal peptidase complex subunit 1 homolog (S. cerevisiae)								REACTOME_METABOLISM_OF_PROTEINS		
1936	EEF1D	eukaryotic translation elongation factor 1 delta (guanine nucleotide exchange protein)								REACTOME_METABOLISM_OF_PROTEINS		
285362	SUMF1	sulfatase modifying factor 1								REACTOME_METABOLISM_OF_PROTEINS		
8818	DPM2	dolichyl-phosphate mannosyltransferase polypeptide 2, regulatory subunit								REACTOME_METABOLISM_OF_PROTEINS		
440138	ALG11	asparagine-linked glycosylation 11, alpha-1,2-mannosyltransferase homolog (yeast)								REACTOME_METABOLISM_OF_PROTEINS		
79868	ALG13	asparagine-linked glycosylation 13 homolog (S. cerevisiae)								REACTOME_METABOLISM_OF_PROTEINS		
5281	PIGF	phosphatidylinositol glycan anchor biosynthesis, class F								REACTOME_METABOLISM_OF_PROTEINS		
63917	GALNT11	UDP-N-acetyl-alpha-D-galactosamine:polypeptide N-acetylgalactosaminyltransferase 11 (GalNAc-T11)								REACTOME_METABOLISM_OF_PROTEINS		
581	BAX	BCL2-associated X protein									PID_P53_DOWNSTREAM_PATHWAY	
4292	MLH1	mutL homolog 1, colon cancer, nonpolyposis type 2 (E. coli)									PID_P53_DOWNSTREAM_PATHWAY	
3276	PRMT1	protein arginine methyltransferase 1									PID_P53_DOWNSTREAM_PATHWAY	
9538	EI24	etoposide induced 2.4 mRNA										
10051	SMC4	structural maintenance of chromosomes 4										
6470	SHMT1	serine hydroxymethyltransferase 1 (soluble)										
4678	NASP	nuclear autoantigenic sperm protein (histone-binding)										
10399	GNB2L1	guanine nucleotide binding protein (G protein), beta polypeptide 2-like 1										
52	ACP1	acid phosphatase 1, soluble										
11260	XPOT	exportin, tRNA (nuclear export receptor for tRNAs)										
1452	CSNK1A1	casein kinase 1, alpha 1										
23054	NCOA6	nuclear receptor coactivator 6										
6720	SREBF1	sterol regulatory element binding transcription factor 1										
57448	BIRC6	baculoviral IAP repeat containing 6										
10006	ABI1	abl-interactor 1										
2035	EPB41	erythrocyte membrane protein band 4.1 (elliptocytosis 1, RH-linked)										
85464	SSH2	slingshot homolog 2 (Drosophila)										
5311	PKD2	polycystic kidney disease 2 (autosomal dominant)										
586	BCAT1	branched chain amino-acid transaminase 1, cytosolic										
64087	MCCC2	methylcrotonoyl-CoA carboxylase 2 (beta)										
5832	ALDH18A1	aldehyde dehydrogenase 18 family, member A1										
883	CCBL1	cysteine conjugate-beta lyase, cytoplasmic										
51074	APIP	APAF1 interacting protein										
4864	NPC1	Niemann-Pick disease, type C1										
8443	GNPAT	glyceronephosphate O-acyltransferase										
2030	SLC29A1	solute carrier family 29 (nucleoside transporters), member 1										
10350	ABCA9	ATP-binding cassette, sub-family A (ABC1), member 9										
10133	OPTN	optineurin										
5189	PEX1	peroxisomal biogenesis factor 1										
7453	WARS	tryptophanyl-tRNA synthetase										
51520	LARS	leucyl-tRNA synthetase										
8565	YARS	tyrosyl-tRNA synthetase										
57038	RARS2	arginyl-tRNA synthetase 2, mitochondrial										
9439	MED23	mediator complex subunit 23										
2104	ESRRG	estrogen-related receptor gamma										
10781	ZNF266	zinc finger protein 266										
162967	ZNF320	zinc finger protein 320										
7581	ZNF33A	zinc finger protein 33A										
90594	ZNF439	zinc finger protein 439										
10919	EHMT2	euchromatic histone-lysine N-methyltransferase 2										
23760	PITPNB	phosphatidylinositol transfer protein, beta										
441024	MTHFD2L	methylenetetrahydrofolate dehydrogenase (NADP+ dependent) 2-like										
54843	SYTL2	synaptotagmin-like 2										
6560	SLC12A4	solute carrier family 12 (potassium/chloride transporters), member 4										
5091	PC	pyruvate carboxylase										
2643	GCH1	GTP cyclohydrolase 1										
54464	XRN1	5'-3' exoribonuclease 1										
27124	INPP5J	inositol polyphosphate-5-phosphatase J										
54675	CRLS1	cardiolipin synthase 1										
51144	HSD17B12	hydroxysteroid (17-beta) dehydrogenase 12										
5893	RAD52	RAD52 homolog (S. cerevisiae)										
9013	TAF1C	TATA box binding protein (TBP)-associated factor, RNA polymerase I, C, 110kDa										
2987	GUK1	guanylate kinase 1										
81539	SLC38A1	solute carrier family 38, member 1										
84912	SLC35B4	solute carrier family 35, member B4										
6565	SLC15A2	solute carrier family 15 (H+/peptide transporter), member 2										
2289	FKBP5	FK506 binding protein 5										
6602	SMARCD1	SWI/SNF related, matrix associated, actin dependent regulator of chromatin, subfamily d, member 1										
2582	GALE	UDP-galactose-4-epimerase										
51005	AMDHD2	amidohydrolase domain containing 2										
5973	RENBP	renin binding protein										
10380	BPNT1	3'(2'), 5'-bisphosphate nucleotidase 1										
6092	ROBO2	roundabout, axon guidance receptor, homolog 2 (Drosophila)										
10507	SEMA4D	sema domain, immunoglobulin domain (Ig), transmembrane domain (TM) and short cytoplasmic domain, (semaphorin) 4D										
23163	GGA3	golgi-associated, gamma adaptin ear containing, ARF binding protein 3										
8729	GBF1	golgi brefeldin A resistant guanine nucleotide exchange factor 1										
64895	PAPOLG	poly(A) polymerase gamma										
84186	ZCCHC7	zinc finger, CCHC domain containing 7										
3795	KHK	ketohexokinase (fructokinase)										
22900	CARD8	caspase recruitment domain family, member 8										
4696	NDUFA3	NADH dehydrogenase (ubiquinone) 1 alpha subcomplex, 3, 9kDa										
4726	NDUFS6	NADH dehydrogenase (ubiquinone) Fe-S protein 6, 13kDa (NADH-coenzyme Q reductase)										
54982	CLN6	ceroid-lipofuscinosis, neuronal 6, late infantile, variant										
571	BACH1	BTB and CNC homology 1, basic leucine zipper transcription factor 1										
8824	CES2	carboxylesterase 2										
79869	CPSF7	cleavage and polyadenylation specific factor 7, 59kDa										
5826	ABCD4	ATP-binding cassette, sub-family D (ALD), member 4										
90665	TBL1Y	transducin (beta)-like 1, Y-linked										
23527	ACAP2	ArfGAP with coiled-coil, ankyrin repeat and PH domains 2										
9615	GDA	guanine deaminase										
10201	NME6	non-metastatic cells 6, protein expressed in (nucleoside-diphosphate kinase)										
1997	ELF1	E74-like factor 1 (ets domain transcription factor)										
85363	TRIM5	tripartite motif containing 5										
8883	NAE1	NEDD8 activating enzyme E1 subunit 1										
10068	IL18BP	interleukin 18 binding protein										
26053	AUTS2	autism susceptibility candidate 2										
50856	CLEC4A	C-type lectin domain family 4, member A										
83734	ATG10	ATG10 autophagy related 10 homolog (S. cerevisiae)										
84869	CBR4	carbonyl reductase 4										
79050	NOC4L	nucleolar complex associated 4 homolog (S. cerevisiae)										
25977	NECAP1	NECAP endocytosis associated 1										
29121	CLEC2D	C-type lectin domain family 2, member D										
813	CALU	calumenin										
2803	GOLGA4	golgin A4										
55768	NGLY1	N-glycanase 1										
84364	ARFGAP2	ADP-ribosylation factor GTPase activating protein 2										
25992	SNED1	sushi, nidogen and EGF-like domains 1										
78987	CRELD1	cysteine-rich with EGF-like domains 1										
64225	ATL2	atlastin GTPase 2										
84173	ELMOD3	ELMO/CED-12 domain containing 3										
8372	HYAL3	hyaluronoglucosaminidase 3										
1954	MEGF8	multiple EGF-like-domains 8										
57089	ENTPD7	ectonucleoside triphosphate diphosphohydrolase 7										
11325	DDX42	DEAD (Asp-Glu-Ala-Asp) box polypeptide 42										
931	MS4A1	membrane-spanning 4-domains, subfamily A, member 1										
317749	DHRS4L2	dehydrogenase/reductase (SDR family) member 4 like 2										
6873	TAF2	TAF2 RNA polymerase II, TATA box binding protein (TBP)-associated factor, 150kDa										
83605	CCM2	cerebral cavernous malformation 2										
9942	XYLB	xylulokinase homolog (H. influenzae)										
100129482	ZNF37BP	zinc finger protein 37B, pseudogene										
10625	IVNS1ABP	influenza virus NS1A binding protein										
10799	RPP40	ribonuclease P/MRP 40kDa subunit										
1106	CHD2	chromodomain helicase DNA binding protein 2										
11257	TP53TG1	TP53 target 1 (non-protein coding)										
11272	PRR4	proline rich 4 (lacrimal)										
114793	FMNL2	formin-like 2										
1184	CLCN5	chloride channel 5										
122509	IFI27L1	interferon, alpha-inducible protein 27-like 1										
122704	MRPL52	mitochondrial ribosomal protein L52										
123096	SLC25A29	solute carrier family 25, member 29										
125488	TTC39C	tetratricopeptide repeat domain 39C										
126075	CCDC159	coiled-coil domain containing 159										
128387	TATDN3	TatD DNase domain containing 3										
128977	C22orf39	chromosome 22 open reading frame 39										
129530	LYG1	lysozyme G-like 1										
131601	TPRA1	transmembrane protein, adipocyte asscociated 1										
144132	DNHD1	dynein heavy chain domain 1										
149951	COMMD7	COMM domain containing 7										
150472	CBWD2	COBW domain containing 2										
158358	KIAA2026	KIAA2026										
165055	CCDC138	coiled-coil domain containing 138										
196074	METTL15	methyltransferase like 15										
199746	U2AF1L4	U2 small nuclear RNA auxiliary factor 1-like 4										
200030	NBPF11	neuroblastoma breakpoint family, member 11										
201931	TMEM192	transmembrane protein 192										
202309	GAPT	GRB2-binding adaptor protein, transmembrane										
202559	KHDRBS2	KH domain containing, RNA binding, signal transduction associated 2										
2186	BPTF	bromodomain PHD finger transcription factor										
219899	TBCEL	tubulin folding cofactor E-like										
219970	GLYATL2	glycine-N-acyltransferase-like 2										
221302	ZUFSP	zinc finger with UFM1-specific peptidase domain										
2218	FKTN	fukutin										
222234	FAM185A	family with sequence similarity 185, member A										
222658	KCTD20	potassium channel tetramerisation domain containing 20										
22878	TRAPPC8	trafficking protein particle complex 8										
22906	TRAK1	trafficking protein, kinesin binding 1										
23061	TBC1D9B	TBC1 domain family, member 9B (with GRAM domain)										
23094	SIPA1L3	signal-induced proliferation-associated 1 like 3										
23122	CLASP2	cytoplasmic linker associated protein 2										
23155	CLCC1	chloride channel CLIC-like 1										
23232	TBC1D12	TBC1 domain family, member 12										
23247	KIAA0556	KIAA0556										
23353	SUN1	Sad1 and UNC84 domain containing 1										
23505	TMEM131	transmembrane protein 131										
23512	SUZ12	suppressor of zeste 12 homolog (Drosophila)										
23774	BRD1	bromodomain containing 1										
23787	MTCH1	mitochondrial carrier 1										
246269	LACE1	lactation elevated 1										
256364	EML3	echinoderm microtubule associated protein like 3										
256380	SCML4	sex comb on midleg-like 4 (Drosophila)										
25934	NIPSNAP3A	nipsnap homolog 3A (C. elegans)										
26009	ZZZ3	zinc finger, ZZ-type containing 3										
26137	ZBTB20	zinc finger and BTB domain containing 20										
27067	STAU2	staufen, RNA binding protein, homolog 2 (Drosophila)										
27130	INVS	inversin										
27315	PGAP2	post-GPI attachment to proteins 2										
27352	SGSM3	small G protein signaling modulator 3										
282809	POC1B	POC1 centriolar protein homolog B (Chlamydomonas)										
283431	GAS2L3	growth arrest-specific 2 like 3										
283755	HERC2P3	hect domain and RLD 2 pseudogene 3										
284565	NBPF15	neuroblastoma breakpoint family, member 15										
285025	CCDC141	coiled-coil domain containing 141										
285386	TPRG1	tumor protein p63 regulated 1										
285636	C5orf51	chromosome 5 open reading frame 51										
286257	C9orf142	chromosome 9 open reading frame 142										
286336	FAM78A	family with sequence similarity 78, member A										
28638	TRBC2	T cell receptor beta constant 2										
29803	REPIN1	replication initiator 1										
340252	ZNF680	zinc finger protein 680										
375248	ANKRD36	ankyrin repeat domain 36										
400410	ST20	suppressor of tumorigenicity 20										
400954	EML6	echinoderm microtubule associated protein like 6										
4154	MBNL1	muscleblind-like (Drosophila)										
440590	ZYG11A	zyg-11 homolog A (C. elegans)										
440675	NBPF12	neuroblastoma breakpoint family, member 12										
440712	C1orf186	chromosome 1 open reading frame 186										
503538	A1BG-AS1	A1BG antisense RNA 1 (non-protein coding)										
51479	ANKFY1	ankyrin repeat and FYVE domain containing 1										
51501	C11orf73	chromosome 11 open reading frame 73										
51523	CXXC5	CXXC finger protein 5										
51643	TMBIM4	transmembrane BAX inhibitor motif containing 4										
5218	CDK14	cyclin-dependent kinase 14										
53346	TM6SF1	transmembrane 6 superfamily member 1										
53904	MYO3A	myosin IIIA										
54039	PCBP3	poly(rC) binding protein 3										
54093	SETD4	SET domain containing 4										
54470	ARMCX6	armadillo repeat containing, X-linked 6										
54517	PUS7	pseudouridylate synthase 7 homolog (S. cerevisiae)										
54535	CCHCR1	coiled-coil alpha-helical rod protein 1										
54542	RC3H2	ring finger and CCCH-type domains 2										
54811	ZNF562	zinc finger protein 562										
54828	BCAS3	breast carcinoma amplified sequence 3										
55083	KIF26B	kinesin family member 26B										
55102	ATG2B	ATG2 autophagy related 2 homolog B (S. cerevisiae)										
55132	LARP1B	La ribonucleoprotein domain family, member 1B										
55198	APPL2	adaptor protein, phosphotyrosine interaction, PH domain and leucine zipper containing 2										
55337	C19orf66	chromosome 19 open reading frame 66										
55486	PARL	presenilin associated, rhomboid-like										
55631	LRRC40	leucine rich repeat containing 40										
55671	SMEK1	SMEK homolog 1, suppressor of mek1 (Dictyostelium)										
55784	MCTP2	multiple C2 domains, transmembrane 2										
56834	GPR137	G protein-coupled receptor 137										
56948	SDR39U1	short chain dehydrogenase/reductase family 39U, member 1										
57409	MIF4GD	MIF4G domain containing										
57519	STARD9	StAR-related lipid transfer (START) domain containing 9										
5863	RGL2	ral guanine nucleotide dissociation stimulator-like 2										
63897	HEATR6	HEAT repeat containing 6										
64062	RBM26	RNA binding motif protein 26										
641638	SNHG6	small nucleolar RNA host gene 6 (non-protein coding)										
645784	ANKRD36BP2	ankyrin repeat domain 36B pseudogene 2										
65084	TMEM135	transmembrane protein 135										
65268	WNK2	WNK lysine deficient protein kinase 2										
65981	CAPRIN2	caprin family member 2										
6855	SYP	synaptophysin										
6871	TADA2A	transcriptional adaptor 2A										
6919	TCEA2	transcription elongation factor A (SII), 2										
7267	TTC3	tetratricopeptide repeat domain 3										
728013	CBWD7	COBW domain containing 7										
7443	VRK1	vaccinia related kinase 1										
7776	ZNF236	zinc finger protein 236										
79074	C2orf49	chromosome 2 open reading frame 49										
7918	GPANK1	G patch domain and ankyrin repeats 1										
79368	FCRL2	Fc receptor-like 2										
79573	TTC13	tetratricopeptide repeat domain 13										
79705	LRRK1	leucine-rich repeat kinase 1										
79805	VASH2	vasohibin 2										

79848	CSPP1	centrosome and spindle pole associated protein 1										
79879	CCDC134	coiled-coil domain containing 134										
79915	ATAD5	ATPase family, AAA domain containing 5										
79930	DOK3	docking protein 3										
79934	ADCK4	aarF domain containing kinase 4										
80008	TMEM156	transmembrane protein 156										
80011	FAM192A	family with sequence similarity 192, member A										
80169	CTC1	CTS telomere maintenance complex component 1										
80205	CHD9	chromodomain helicase DNA binding protein 9										
80279	CDK5RAP3	CDK5 regulatory subunit associated protein 3										
8148	TAF15	TAF15 RNA polymerase II, TATA box binding protein (TBP)-associated factor, 68kDa										
81553	FAM49A	family with sequence similarity 49, member A										
81669	CCNL2	cyclin L2										
81853	TMEM14B	transmembrane protein 14B										
81858	SHARPIN	SHANK-associated RH domain interactor										
83607	AMMECR1L	AMME chromosomal region gene 1-like										
83707	TRPT1	tRNA phosphotransferase 1										
83862	TMEM120A	transmembrane protein 120A										
83989	FAM172A	family with sequence similarity 172, member A										
84164	ASCC2	activating signal cointegrator 1 complex subunit 2										
84280	BTBD10	BTB (POZ) domain containing 10										
84287	ZDHHC16	zinc finger, DHHC-type containing 16										
8434	RECK	reversion-inducing-cysteine-rich protein with kazal motifs										
84824	FCRLA	Fc receptor-like A										
84964	ALKBH6	alkB, alkylation repair homolog 6 (E. coli)										
8847	DLEU2	deleted in lymphocytic leukemia 2 (non-protein coding)										
8904	CPNE1	copine I										
89894	TMEM116	transmembrane protein 116										
90649	ZNF486	zinc finger protein 486										
90835	C16orf93	chromosome 16 open reading frame 93										
91351	DDX60L	DEAD (Asp-Glu-Ala-Asp) box polypeptide 60-like										
91607	SLFN11	schlafen family member 11										
9205	ZMYM5	zinc finger, MYM-type 5										
92482	BBIP1	BBSome interacting protein 1										
94081	SFXN1	sideroflexin 1										
9692	KIAA0391	KIAA0391										
9703	KIAA0100	KIAA0100										
9841	ZBTB24	zinc finger and BTB domain containing 24										

Supplementary Table 12: Association of 10-day Activity Area from Eurofins Cell Line Panel with Sanger Mutations

genes	p.value	mutatedCellLines	median_WT	median_MT	dMedian
ADC	0.000220695	141	3.55	4.29	-0.74
MEGF8	0.000671984	17	2.82	3.93	-1.11
COX4I2	0.000696504	6	2.155	3.81	-1.655
CAPN1	0.001074389	46	3.425	3.97	-0.545
INPPL1	0.001625844	17	2.85	3.89	-1.04
DIS3	0.001710364	30	3.34	3.95	-0.61
USP32	0.001834347	5	1.97	3.78	-1.81
ACIN1	0.002163315	23	3.25	3.945	-0.695
CCDC30	0.002401392	12	2.75	3.91	-1.16
TP53	0.002649119	49	3.47	3.96	-0.49
ANKRD52	0.002651865	24	3.245	3.92	-0.675
C20orf186	0.002731218	12	3.135	3.91	-0.775
CSPG4	0.002862413	16	3.365	3.92	-0.555
ATP1B2	0.002898047	4	1.525	3.75	-2.225
CNGA3	0.003008393	14	3.11	3.91	-0.8
TRH	0.003042461	5	1.84	3.78	-1.94
ANKRD26	0.003214683	30	3.34	3.94	-0.6
NCOR1	0.003466409	9	4.41	3.685	0.725
CASKIN1	0.00370116	8	2.655	3.87	-1.215
ENPEP	0.003787563	13	2.36	3.84	-1.48

Supplementary Table 12: Association of 10-day gIC$_{50}$ Values from Eurofins Cell Line Panel with Sanger Mutations

genes	p.value	mutatedCellLines	median_WT	median_MT	dMedian
MEGF8	0.000230006	17	602	149.5	452.5
TP53	0.000314354	49	257	143	114
C20orf186	0.00043074	12	336	152	184
COX4I2	0.000501276	6	1626.5	154	1472.5
CSPG4	0.000926814	16	308.5	152	156.5
CNGA3	0.00100639	14	490.5	152	338.5
CAPN1	0.00107443	46	270.5	147	123.5
ACIN1	0.001297018	23	299	147	152
ABCA8	0.001312394	36	258	147	111
USP32	0.001332233	5	1170	155	1015
LRRC4	0.00158154	20	365.5	152	213.5
ATP8A2	0.001674299	34	276	152	124
CASKIN1	0.001946257	8	760	153	607
CA8	0.001978389	40	248.5	146	102.5
BAIAP3	0.002440538	15	294	152	142
EPHB1	0.002497451	16	660	153	507
TRH	0.002505527	5	1170	155	1015
ANKRD52	0.002529885	24	290	147	143
BCLAF1	0.002663209	30	298	152	146
ATP1B2	0.00289814	4	2305	156	2149

Supplementary Table 14: Positive Pearson Correlation of Expression GSEA report from Activity Area

NAME	GS<br>follow link to MSigDB	GS DETAILS	SIZE	ES	NOM p-val	NES	FDR q-val	FWER p-val	RANK AT MAX	LEADING EDGE	Splicing/mRNA Processing
REACTOME_PROCESSING_OF_CAPPED_INTRON_CONTAINING_PRE_MRNA	REACTOME_PROCESSING_OF_CAPPED_INTRON_CONTAINING_PRE_MRNA	Details ...	129	-0.6459573	0	-2.8447914	0	0	4186	tags=67%, list=22%, signal=85%	TRUE
REACTOME_MRNA_SPLICING	REACTOME_MRNA_SPLICING	Details ...	101	-0.674756	0	-2.827584	0	0	4186	tags=72%, list=22%, signal=92%	TRUE
REACTOME_MRNA_PROCESSING	REACTOME_MRNA_PROCESSING	Details ...	146	-0.62379545	0	-2.75242	0	0	4186	tags=62%, list=22%, signal=79%	TRUE
KEGG_SPLICEOSOME	KEGG_SPLICEOSOME	Details ...	120	-0.6264446	0	-2.7331548	0	0	4186	tags=68%, list=22%, signal=86%	TRUE
REACTOME_3_UTR_MEDIATED_TRANSLATIONAL_REGULATION	REACTOME_3_UTR_MEDIATED_TRANSLATIONAL_REGULATION	Details ...	52	-0.7109544	0	-2.7049658	0	0	2637	tags=65%, list=14%, signal=76%	FALSE
HALLMARK_MYC_TARGETS_V1	HALLMARK_MYC_TARGETS_V1	Details ...	178	-0.59674025	0	-2.7011209	0	0	5421	tags=67%, list=29%, signal=94%	FALSE
REACTOME_PEPTIDE_CHAIN_ELONGATION	REACTOME_PEPTIDE_CHAIN_ELONGATION	Details ...	38	-0.7630061	0	-2.6900802	0	0	1770	tags=68%, list=9%, signal=75%	FALSE
REACTOME_INFLUENZA_VIRAL_RNA_TRANSCRIPTION_AND_REPLICATION	REACTOME_INFLUENZA_VIRAL_RNA_TRANSCRIPTION_AND_REPLICATION	Details ...	54	-0.7010146	0	-2.6838734	0	0	2975	tags=61%, list=16%, signal=72%	FALSE
REACTOME_NONSENSE_MEDIATED_DECAY_ENHANCED_BY_THE_EXON_JUNCTION_COMPLEX	REACTOME_NONSENSE_MEDIATED_DECAY_ENHANCED_BY_THE_EXON_JUNCTION_COMPLEX	Details ...	58	-0.6900549	0	-2.6715739	0	0	4198	tags=74%, list=22%, signal=95%	TRUE
KEGG_RIBOSOME	KEGG_RIBOSOME	Details ...	40	-0.74578923	0	-2.6543474	0	0	2975	tags=75%, list=16%, signal=89%	FALSE
REACTOME_INFLUENZA_LIFE_CYCLE	REACTOME_INFLUENZA_LIFE_CYCLE	Details ...	86	-0.6276603	0	-2.6072252	0	0	2975	tags=51%, list=16%, signal=60%	FALSE
REACTOME_TRANSPORT_OF_MATURE_TRANSCRIPT_TO_CYTOPLASM	REACTOME_TRANSPORT_OF_MATURE_TRANSCRIPT_TO_CYTOPLASM	Details ...	51	-0.67944026	0	-2.5576494	0	0	2961	tags=65%, list=16%, signal=77%	FALSE
HALLMARK_E2F_TARGETS	HALLMARK_E2F_TARGETS	Details ...	191	-0.5493739	0	-2.5335188	0	0	4668	tags=54%, list=25%, signal=72%	FALSE
REACTOME_CLEAVAGE_OF_GROWING_TRANSCRIPT_IN_THE_TERMINATION_REGION_	REACTOME_CLEAVAGE_OF_GROWING_TRANSCRIPT_IN_THE_TERMINATION_REGION_	Details ...	40	-0.6879279	0	-2.4780562	0	0	4046	tags=80%, list=21%, signal=102%	TRUE
REACTOME_TRANSLATION	REACTOME_TRANSLATION	Details ...	90	-0.586893	0	-2.4673183	0	0	3999	tags=56%, list=21%, signal=70%	FALSE
REACTOME_METABOLISM_OF_RNA	REACTOME_METABOLISM_OF_RNA	Details ...	195	-0.5358157	0	-2.4670599	0	0	4198	tags=49%, list=22%, signal=63%	FALSE
REACTOME_MRNA_3_END_PROCESSING	REACTOME_MRNA_3_END_PROCESSING	Details ...	32	-0.7020908	0	-2.3739917	0	0	4388	tags=88%, list=23%, signal=114%	TRUE
REACTOME_METABOLISM_OF_MRNA	REACTOME_METABOLISM_OF_MRNA	Details ...	154	-0.5291658	0	-2.3704436	0	0	4198	tags=48%, list=22%, signal=61%	FALSE
REACTOME_RNA_POL_II_TRANSCRIPTION	REACTOME_RNA_POL_II_TRANSCRIPTION	Details ...	95	-0.55627656	0	-2.340773	0	0	4466	tags=59%, list=24%, signal=77%	FALSE
REACTOME_MRNA_SPLICING_MINOR_PATHWAY	REACTOME_MRNA_SPLICING_MINOR_PATHWAY	Details ...	41	-0.64123046	0	-2.3312953	4.16E-05	0.001	4154	tags=61%, list=22%, signal=78%	TRUE
REACTOME_G2_M_CHECKPOINTS	REACTOME_G2_M_CHECKPOINTS	Details ...	40	-0.6481518	0	-2.32647	3.96E-05	0.001	3243	tags=50%, list=17%, signal=60%	FALSE
HALLMARK_MYC_TARGETS_V2	HALLMARK_MYC_TARGETS_V2	Details ...	55	-0.6123377	0	-2.3241751	3.78E-05	0.001	5173	tags=75%, list=27%, signal=102%	FALSE
REACTOME_ACTIVATION_OF_ATR_IN_RESPONSE_TO_REPLICATION_STRESS	REACTOME_ACTIVATION_OF_ATR_IN_RESPONSE_TO_REPLICATION_STRESS	Details ...	35	-0.6609478	0	-2.3127935	3.61E-05	0.001	3243	tags=54%, list=17%, signal=65%	FALSE
REACTOME_SRP_DEPENDENT_COTRANSLATIONAL_PROTEIN_TARGETING_TO_MEMBRANE	REACTOME_SRP_DEPENDENT_COTRANSLATIONAL_PROTEIN_TARGETING_TO_MEMBRANE	Details ...	61	-0.5901321	0	-2.306744	3.46E-05	0.001	2975	tags=49%, list=16%, signal=58%	FALSE
KEGG_RNA_DEGRADATION	KEGG_RNA_DEGRADATION	Details ...	56	-0.5908727	0	-2.2964482	3.33E-05	0.001	4246	tags=59%, list=22%, signal=76%	FALSE
KEGG_PRIMARY_IMMUNODEFICIENCY	KEGG_PRIMARY_IMMUNODEFICIENCY	Details ...	34	-0.6530874	0	-2.2850645	3.20E-05	0.001	3837	tags=62%, list=20%, signal=77%	FALSE
REACTOME_TRANSCRIPTION	REACTOME_TRANSCRIPTION	Details ...	172	-0.500844	0	-2.275217	3.08E-05	0.001	4490	tags=53%, list=24%, signal=70%	FALSE
HALLMARK_G2M_CHECKPOINT	HALLMARK_G2M_CHECKPOINT	Details ...	196	-0.48244557	0	-2.2251072	9.08E-05	0.003	4527	tags=48%, list=24%, signal=63%	FALSE
REACTOME_ACTIVATION_OF_THE_PRE_REPLICATIVE_COMPLEX	REACTOME_ACTIVATION_OF_THE_PRE_REPLICATIVE_COMPLEX	Details ...	30	-0.67085505	0	-2.2219756	8.77E-05	0.003	3144	tags=57%, list=17%, signal=68%	FALSE
KEGG_DNA_REPLICATION	KEGG_DNA_REPLICATION	Details ...	36	-0.62915367	0	-2.216034	8.48E-05	0.003	4338	tags=64%, list=23%, signal=83%	FALSE
REACTOME_METABOLISM_OF_NON_CODING_RNA	REACTOME_METABOLISM_OF_NON_CODING_RNA	Details ...	44	-0.59580845	0	-2.18121	8.20E-05	0.003	2912	tags=50%, list=15%, signal=59%	TRUE
KEGG_BASE_EXCISION_REPAIR	KEGG_BASE_EXCISION_REPAIR	Details ...	31	-0.6308366	0	-2.152072	1.06E-04	0.004	2173	tags=45%, list=12%, signal=51%	FALSE
REACTOME_ACTIVATION_OF_THE_MRNA_UPON_BINDING_OF_THE_CAP_BINDING_COMPLEX_AND_EIFS_AND_SUBSEQUENT_BINDING_TO_43S	REACTOME_ACTIVATION_OF_THE_MRNA_UPON_BINDING_OF_THE_CAP_BINDING_COMPLEX_AND_EIFS_AND_SUBSEQUENT_BINDING_TO_43S	Details ...	30	-0.63027275	0	-2.1115923	3.09E-04	0.012	5411	tags=77%, list=29%, signal=107%	FALSE
REACTOME_DNA_REPAIR	REACTOME_DNA_REPAIR	Details ...	98	-0.49934965	0	-2.1099505	3.25E-04	0.013	6581	tags=60%, list=35%, signal=92%	FALSE
REACTOME_FORMATION_OF_THE_TERNARY_COMPLEX_AND_SUBSEQUENTLY_THE_43S_COMPLEX	REACTOME_FORMATION_OF_THE_TERNARY_COMPLEX_AND_SUBSEQUENTLY_THE_43S_COMPLEX	Details ...	26	-0.6539195	0	-2.0930018	3.65E-04	0.015	5179	tags=81%, list=27%, signal=111%	FALSE
REACTOME_DNA_STRAND_ELONGATION	REACTOME_DNA_STRAND_ELONGATION	Details ...	30	-0.6126199	0	-2.0567443	6.63E-04	0.028	4933	tags=63%, list=26%, signal=86%	FALSE
REACTOME_DEADENYLATION_DEPENDENT_MRNA_DECAY	REACTOME_DEADENYLATION_DEPENDENT_MRNA_DECAY	Details ...	40	-0.5665749	0	-2.0430028	8.76E-04	0.038	4191	tags=55%, list=22%, signal=71%	TRUE
REACTOME_DESTABILIZATION_OF_MRNA_BY_KSRP	REACTOME_DESTABILIZATION_OF_MRNA_BY_KSRP	Details ...	17	-0.67742777	0	-1.9799412	0.00228441	0.096	4191	tags=65%, list=22%, signal=83%	TRUE
PID_E2F_PATHWAY	PID_E2F_PATHWAY	Details ...	71	-0.49296692	0	-1.9762061	0.002463237	0.106	4610	tags=49%, list=24%, signal=65%	FALSE
REACTOME_RNA_POL_III_TRANSCRIPTION_INITIATION_FROM_TYPE_2_PROMOTER	REACTOME_RNA_POL_III_TRANSCRIPTION_INITIATION_FROM_TYPE_2_PROMOTER	Details ...	23	-0.6256294	0	-1.9743226	0.002550729	0.113	4490	tags=61%, list=24%, signal=80%	FALSE
REACTOME_RNA_POL_III_TRANSCRIPTION	REACTOME_RNA_POL_III_TRANSCRIPTION	Details ...	33	-0.57372385	0	-1.9662645	0.00267585	0.122	6042	tags=67%, list=32%, signal=98%	FALSE
BIOCARTA_RARRXR_PATHWAY	BIOCARTA_RARRXR_PATHWAY	Details ...	15	-0.68678546	0.001594896	-1.9656578	0.002632637	0.123	3909	tags=53%, list=21%, signal=67%	FALSE
REACTOME_MITOTIC_G1_G1_S_PHASES	REACTOME_MITOTIC_G1_G1_S_PHASES	Details ...	125	-0.4476225	0	-1.9473041	0.003105803	0.147	5547	tags=48%, list=29%, signal=67%	FALSE
PID_BARD1_PATHWAY	PID_BARD1_PATHWAY	Details ...	28	-0.582634	0.00148368	-1.9460182	0.003054731	0.148	4731	tags=61%, list=25%, signal=81%	FALSE
REACTOME_CELL_CYCLE_CHECKPOINTS	REACTOME_CELL_CYCLE_CHECKPOINTS	Details ...	105	-0.4543188	0	-1.9434242	0.003193238	0.157	4230	tags=37%, list=22%, signal=48%	FALSE
REACTOME_G0_AND_EARLY_G1	REACTOME_G0_AND_EARLY_G1	Details ...	23	-0.6122832	0	-1.943007	0.003179075	0.16	6398	tags=83%, list=34%, signal=125%	FALSE
REACTOME_CELL_CYCLE_MITOTIC	REACTOME_CELL_CYCLE_MITOTIC	Details ...	296	-0.40451768	0	-1.9408631	0.003293238	0.17	5595	tags=45%, list=30%, signal=63%	FALSE
REACTOME_PROCESSING_OF_CAPPED_INTRONLESS_PRE_MRNA	REACTOME_PROCESSING_OF_CAPPED_INTRONLESS_PRE_MRNA	Details ...	21	-0.6252946	0	-1.9383767	0.00340256	0.18	6219	tags=90%, list=33%, signal=135%	TRUE
REACTOME_NUCLEOTIDE_EXCISION_REPAIR	REACTOME_NUCLEOTIDE_EXCISION_REPAIR	Details ...	44	-0.52950406	0	-1.9374983	0.00333312	0.18	6042	tags=61%, list=32%, signal=90%	FALSE
REACTOME_GLOBAL_GENOMIC_NER_GG_NER	REACTOME_GLOBAL_GENOMIC_NER_GG_NER	Details ...	29	-0.5881858	0.001517451	-1.9330552	0.0033851	0.187	6005	tags=69%, list=32%, signal=101%	FALSE
KEGG_NUCLEOTIDE_EXCISION_REPAIR	KEGG_NUCLEOTIDE_EXCISION_REPAIR	Details ...	41	-0.5419974	0	-1.9323093	0.003335366	0.188	6005	tags=66%, list=32%, signal=96%	FALSE
REACTOME_TRANSPORT_OF_MATURE_MRNA_DERIVED_FROM_AN_INTRONLESS_TRANSCRIPT	REACTOME_TRANSPORT_OF_MATURE_MRNA_DERIVED_FROM_AN_INTRONLESS_TRANSCRIPT	Details ...	31	-0.5749481	0	-1.9279239	0.003532693	0.202	2912	tags=48%, list=15%, signal=57%	FALSE
BIOCARTA_MCM_PATHWAY	BIOCARTA_MCM_PATHWAY	Details ...	18	-0.64118826	0.001531394	-1.9233309	0.003803686	0.22	3144	tags=56%, list=17%, signal=67%	FALSE
PID_P53_REGULATION_PATHWAY	PID_P53_REGULATION_PATHWAY	Details ...	58	-0.49858072	0	-1.9229271	0.003749337	0.22	6609	tags=62%, list=35%, signal=95%	FALSE
REACTOME_RNA_POL_III_TRANSCRIPTION_INITIATION_FROM_TYPE_3_PROMOTER	REACTOME_RNA_POL_III_TRANSCRIPTION_INITIATION_FROM_TYPE_3_PROMOTER	Details ...	26	-0.57777995	0	-1.9221485	0.003727102	0.221	6737	tags=73%, list=36%, signal=113%	FALSE
PID_FANCONI_PATHWAY	PID_FANCONI_PATHWAY		47	-0.5199466	0	-1.9142041	0.004099174	0.246	4583	tags=51%, list=24%, signal=67%	FALSE
KEGG_RNA_POLYMERASE	KEGG_RNA_POLYMERASE		27	-0.5800071	0.003067485	-1.8938987	0.005039014	0.295	6930	tags=74%, list=37%, signal=117%	FALSE
REACTOME_HIV_LIFE_CYCLE	REACTOME_HIV_LIFE_CYCLE		107	-0.4393655	0	-1.8826659	0.005990841	0.338	3281	tags=38%, list=17%, signal=46%	FALSE
REACTOME_CELL_CYCLE	REACTOME_CELL_CYCLE		370	-0.38327965	0	-1.868475	0.007256683	0.389	5547	tags=45%, list=29%, signal=62%	FALSE
KEGG_MISMATCH_REPAIR	KEGG_MISMATCH_REPAIR		22	-0.59075665	0.00311042	-1.8597987	0.007959246	0.427	6005	tags=73%, list=32%, signal=106%	FALSE
REACTOME_REGULATION_OF_HYPOXIA_INDUCIBLE_FACTOR_HIF_BY_OXYGEN	REACTOME_REGULATION_OF_HYPOXIA_INDUCIBLE_FACTOR_HIF_BY_OXYGEN		22	-0.59105945	0.003149606	-1.8533489	0.00842618	0.452	2621	tags=50%, list=14%, signal=58%	FALSE
PID_MYC_ACTIV_PATHWAY	PID_MYC_ACTIV_PATHWAY		72	-0.463544	0	-1.848791	0.008659091	0.465	5308	tags=53%, list=28%, signal=73%	FALSE
REACTOME_APC_CDC20_MEDIATED_DEGRADATION_OF_NEK2A	REACTOME_APC_CDC20_MEDIATED_DEGRADATION_OF_NEK2A		19	-0.6144386	0.00462963	-1.8455536	0.008912786	0.479	3871	tags=58%, list=20%, signal=73%	FALSE
PID_ATR_PATHWAY	PID_ATR_PATHWAY		39	-0.51631737	0	-1.8398156	0.009559088	0.509	4414	tags=46%, list=23%, signal=60%	FALSE
REACTOME_GENERATION_OF_SECOND_MESSENGER_MOLECULES	REACTOME_GENERATION_OF_SECOND_MESSENGER_MOLECULES		24	-0.5714693	0	-1.8397971	0.009412025	0.509	6756	tags=71%, list=36%, signal=110%	FALSE
SIG_BCR_SIGNALING_PATHWAY	SIG_BCR_SIGNALING_PATHWAY		46	-0.50174004	0	-1.839014	0.009308457	0.51	2195	tags=35%, list=12%, signal=39%	FALSE
REACTOME_TRANSCRIPTION_COUPLED_NER_TC_NER	REACTOME_TRANSCRIPTION_COUPLED_NER_TC_NER		39	-0.51289827	0.002853067	-1.8380181	0.009322501	0.516	6042	tags=59%, list=32%, signal=87%	FALSE
REACTOME_MRNA_CAPPING	REACTOME_MRNA_CAPPING		26	-0.5544869	0.00152439	-1.8366168	0.009297967	0.522	6737	tags=62%, list=36%, signal=96%	TRUE
REACTOME_DOUBLE_STRAND_BREAK_REPAIR	REACTOME_DOUBLE_STRAND_BREAK_REPAIR		21	-0.59211785	0.001545595	-1.8360096	0.009274426	0.526	4414	tags=62%, list=23%, signal=81%	FALSE
KEGG_HOMOLOGOUS_RECOMBINATION	KEGG_HOMOLOGOUS_RECOMBINATION		28	-0.5610191	0	-1.832163	0.00948239	0.541	4414	tags=61%, list=23%, signal=79%	FALSE
REACTOME_RESPIRATORY_ELECTRON_TRANSPORT_ATP_SYNTHESIS_BY_CHEMIOSMOTIC_COUPLING_AND_HEAT_PRODUCTION_BY_UNCOUPLING_PROTEINS_	REACTOME_RESPIRATORY_ELECTRON_TRANSPORT_ATP_SYNTHESIS_BY_CHEMIOSMOTIC_COUPLING_AND_HEAT_PRODUCTION_BY_UNCOUPLING_PROTEINS_		72	-0.45674604	0	-1.8294467	0.009576488	0.553	7033	tags=61%, list=37%, signal=97%	FALSE
REACTOME_ANTIGEN_ACTIVATES_B_CELL_RECEPTOR_LEADING_TO_GENERATION_OF_SECOND_MESSENGERS	REACTOME_ANTIGEN_ACTIVATES_B_CELL_RECEPTOR_LEADING_TO_GENERATION_OF_SECOND_MESSENGERS		29	-0.5541036	0	-1.8289685	0.009525351	0.556	1913	tags=38%, list=10%, signal=42%	FALSE
KEGG_CELL_CYCLE	KEGG_CELL_CYCLE		119	-0.4221169	0	-1.8286151	0.009464556	0.558	5698	tags=50%, list=30%, signal=72%	FALSE
REACTOME_EXTENSION_OF_TELOMERES	REACTOME_EXTENSION_OF_TELOMERES		27	-0.54876375	0.001526718	-1.8224795	0.010056827	0.589	6393	tags=70%, list=34%, signal=106%	FALSE
REACTOME_APC_C_CDC20_MEDIATED_DEGRADATION_OF_CYCLIN_B	REACTOME_APC_C_CDC20_MEDIATED_DEGRADATION_OF_CYCLIN_B		17	-0.6231879	0.003144654	-1.8213794	0.009967145	0.59	3871	tags=53%, list=20%, signal=67%	FALSE
REACTOME_DEADENYLATION_OF_MRNA	REACTOME_DEADENYLATION_OF_MRNA		15	-0.63645905	0.001536098	-1.812218	0.011163083	0.635	6372	tags=87%, list=34%, signal=131%	TRUE
REACTOME_DNA_REPLICATION	REACTOME_DNA_REPLICATION		181	-0.39676428	0	-1.8099257	0.011371603	0.647	5760	tags=44%, list=30%, signal=63%	FALSE
REACTOME_S_PHASE	REACTOME_S_PHASE		102	-0.42532387	0	-1.8025489	0.01222582	0.684	5542	tags=46%, list=29%, signal=65%	FALSE
REACTOME_PKB_MEDIATED_EVENTS	REACTOME_PKB_MEDIATED_EVENTS		26	-0.55317414	0.004552352	-1.7993017	0.012513493	0.697	2321	tags=31%, list=12%, signal=35%	FALSE
REACTOME_FANCONI_ANEMIA_PATHWAY	REACTOME_FANCONI_ANEMIA_PATHWAY		21	-0.5830202	0.001508296	-1.7976738	0.012580811	0.705	4583	tags=52%, list=24%, signal=69%	FALSE
REACTOME_ASSOCIATION_OF_TRIC_CCT_WITH_TARGET_PROTEINS_DURING_BIOSYNTHESIS	REACTOME_ASSOCIATION_OF_TRIC_CCT_WITH_TARGET_PROTEINS_DURING_BIOSYNTHESIS		25	-0.55054605	0.002971768	-1.7850266	0.014514579	0.755	4876	tags=48%, list=26%, signal=65%	FALSE
REACTOME_RNA_POL_I_RNA_POL_III_AND_MITOCHONDRIAL_TRANSCRIPTION	REACTOME_RNA_POL_I_RNA_POL_III_AND_MITOCHONDRIAL_TRANSCRIPTION		90	-0.42714897	0	-1.7801294	0.015255692	0.776	6042	tags=57%, list=32%, signal=83%	FALSE
REACTOME_LATE_PHASE_OF_HIV_LIFE_CYCLE	REACTOME_LATE_PHASE_OF_HIV_LIFE_CYCLE		95	-0.42196265	0	-1.779888	0.015132493	0.777	7420	tags=71%, list=39%, signal=116%	FALSE
REACTOME_M_G1_TRANSITION	REACTOME_M_G1_TRANSITION		75	-0.4386461	0	-1.7792546	0.015083499	0.781	3144	tags=31%, list=17%, signal=37%	FALSE
PID_LPA4_PATHWAY	PID_LPA4_PATHWAY		15	-0.62056637	0.008278145	-1.7721212	0.016457064	0.818	5203	tags=60%, list=28%, signal=83%	FALSE
REACTOME_RNA_POL_III_CHAIN_ELONGATION	REACTOME_RNA_POL_III_CHAIN_ELONGATION		17	-0.6116134	0.001481482	-1.7697933	0.01663129	0.823	4490	tags=53%, list=24%, signal=69%	FALSE
REACTOME_TRANSPORT_OF_RIBONUCLEOPROTEINS_INTO_THE_HOST_NUCLEUS	REACTOME_TRANSPORT_OF_RIBONUCLEOPROTEINS_INTO_THE_HOST_NUCLEUS		26	-0.5443702	0	-1.766159	0.017028807	0.83	2912	tags=42%, list=15%, signal=50%	FALSE
REACTOME_G1_S_TRANSITION	REACTOME_G1_S_TRANSITION		101	-0.41937256	0	-1.7654934	0.01694115	0.834	5698	tags=46%, list=30%, signal=65%	FALSE
REACTOME_DESTABILIZATION_OF_MRNA_BY_BRF1	REACTOME_DESTABILIZATION_OF_MRNA_BY_BRF1		17	-0.6001382	0.009230769	-1.7641793	0.016989687	0.84	4587	tags=71%, list=24%, signal=93%	FALSE
REACTOME_RNA_POL_II_PRE_TRANSCRIPTION_EVENTS	REACTOME_RNA_POL_II_PRE_TRANSCRIPTION_EVENTS		55	-0.46070692	0.004043127	-1.7628341	0.01709122	0.846	6831	tags=67%, list=36%, signal=105%	FALSE
REACTOME_PKA_MEDIATED_PHOSPHORYLATION_OF_CREB	REACTOME_PKA_MEDIATED_PHOSPHORYLATION_OF_CREB		17	-0.5926713	0.0015625	-1.7540498	0.018713018	0.872	5203	tags=53%, list=28%, signal=73%	FALSE
REACTOME_RESPIRATORY_ELECTRON_TRANSPORT	REACTOME_RESPIRATORY_ELECTRON_TRANSPORT		58	-0.45098653	0	-1.7521731	0.018924199	0.879	6741	tags=57%, list=36%, signal=88%	FALSE
PID_HIF1A_PATHWAY	PID_HIF1A_PATHWAY		18	-0.5864772	0.007751938	-1.7511356	0.018968156	0.883	2621	tags=39%, list=14%, signal=45%	FALSE
KEGG_PARKINSONS_DISEASE	KEGG_PARKINSONS_DISEASE		102	-0.4127374	0.00127551	-1.746253	0.019766709	0.897	7001	tags=60%, list=37%, signal=94%	FALSE
BIOCARTA_HCMV_PATHWAY	BIOCARTA_HCMV_PATHWAY		17	-0.5989846	0	-1.74228	0.020309461	0.904	957	tags=29%, list=5%, signal=31%	FALSE
REACTOME_REGULATION_OF_GLUCOKINASE_BY_GLUCOKINASE_REGULATORY_PROTEIN	REACTOME_REGULATION_OF_GLUCOKINASE_BY_GLUCOKINASE_REGULATORY_PROTEIN		26	-0.5343809	0.003030303	-1.7415664	0.020203546	0.905	2912	tags=42%, list=15%, signal=50%	FALSE
REACTOME_TCR_SIGNALING	REACTOME_TCR_SIGNALING		47	-0.46680793	0.002728513	-1.7393978	0.020519182	0.909	6763	tags=60%, list=36%, signal=93%	FALSE
KEGG_UBIQUITIN_MEDIATED_PROTEOLYSIS	KEGG_UBIQUITIN_MEDIATED_PROTEOLYSIS		128	-0.3931308	0	-1.7392068	0.020361785	0.911	6414	tags=57%, list=34%, signal=86%	FALSE
REACTOME_RNA_POL_III_TRANSCRIPTION_TERMINATION	REACTOME_RNA_POL_III_TRANSCRIPTION_TERMINATION		19	-0.58189046	0.010835913	-1.7381135	0.02042347	0.911	4490	tags=47%, list=24%, signal=62%	FALSE
BIOCARTA_AKAPCENTROSOME_PATHWAY	BIOCARTA_AKAPCENTROSOME_PATHWAY		15	-0.61590874	0.004807693	-1.7371806	0.020431742	0.913	2857	tags=47%, list=15%, signal=55%	FALSE
REACTOME_SYNTHESIS_OF_DNA	REACTOME_SYNTHESIS_OF_DNA		87	-0.42258757	0.001278772	-1.7367423	0.020330084	0.915	5493	tags=44%, list=29%, signal=61%	FALSE
REACTOME_DESTABILIZATION_OF_MRNA_BY_TRISTETRAPROLIN_TTP	REACTOME_DESTABILIZATION_OF_MRNA_BY_TRISTETRAPROLIN_TTP		17	-0.5869548	0.006504065	-1.7338213	0.02088567	0.922	4587	tags=71%, list=24%, signal=93%	FALSE
REACTOME_INTERACTIONS_OF_VPR_WITH_HOST_CELLULAR_PROTEINS	REACTOME_INTERACTIONS_OF_VPR_WITH_HOST_CELLULAR_PROTEINS		31	-0.51447475	0.003016591	-1.7336277	0.020716423	0.922	7420	tags=74%, list=39%, signal=122%	FALSE
REACTOME_FORMATION_OF_RNA_POL_II_ELONGATION_COMPLEX_	REACTOME_FORMATION_OF_RNA_POL_II_ELONGATION_COMPLEX_		39	-0.47910428	0.005970149	-1.7328492	0.020672387	0.923	6737	tags=67%, list=36%, signal=103%	FALSE
BIOCARTA_PML_PATHWAY	BIOCARTA_PML_PATHWAY		17	-0.5934059	0.003159558	-1.7273479	0.021848222	0.931	4496	tags=53%, list=24%, signal=69%	FALSE
KEGG_HUNTINGTONS_DISEASE	KEGG_HUNTINGTONS_DISEASE		157	-0.38778362	0	-1.7273426	0.021642108	0.931	6890	tags=55%, list=36%, signal=86%	FALSE
BIOCARTA_IL7_PATHWAY	BIOCARTA_IL7_PATHWAY		17	-0.5912611	0.003072197	-1.726229	0.021684946	0.932	4591	tags=53%, list=24%, signal=70%	FALSE
REACTOME_PHOSPHORYLATION_OF_THE_APC_C	REACTOME_PHOSPHORYLATION_OF_THE_APC_C		15	-0.6104003	0.009615385	-1.7246544	0.021852985	0.936	3871	tags=47%, list=20%, signal=59%	FALSE
REACTOME_HIV_INFECTION	REACTOME_HIV_INFECTION		183	-0.3743151	0	-1.7243507	0.0217613	0.936	6831	tags=53%, list=36%, signal=82%	FALSE
REACTOME_NEP_NS2_INTERACTS_WITH_THE_CELLULAR_EXPORT_MACHINERY	REACTOME_NEP_NS2_INTERACTS_WITH_THE_CELLULAR_EXPORT_MACHINERY		25	-0.5252429	0.011764706	-1.709979	0.025145682	0.96	7420	tags=80%, list=39%, signal=132%	FALSE
KEGG_PYRIMIDINE_METABOLISM	KEGG_PYRIMIDINE_METABOLISM		93	-0.40352324	0.001333333	-1.7094921	0.025034828	0.96	4490	tags=38%, list=24%, signal=49%	FALSE
REACTOME_INHIBITION_OF_THE_PROTEOLYTIC_ACTIVITY_OF_APC_C_REQUIRED_FOR_THE_ONSET_OF_ANAPHASE_BY_MITOTIC_SPINDLE_CHECKPOINT_COMPONENTS	REACTOME_INHIBITION_OF_THE_PROTEOLYTIC_ACTIVITY_OF_APC_C_REQUIRED_FOR_THE_ONSET_OF_ANAPHASE_BY_MITOTIC_SPINDLE_CHECKPOINT_COMPONENTS		16	-0.5962838	0.011111111	-1.7083305	0.025084881	0.961	3871	tags=56%, list=20%, signal=71%	FALSE
PID_BCR_5PATHWAY	PID_BCR_5PATHWAY		64	-0.43996072	0	-1.7028147	0.026218884	0.969	3810	tags=38%, list=20%, signal=47%	FALSE
REACTOME_MITOTIC_M_M_G1_PHASES	REACTOME_MITOTIC_M_M_G1_PHASES		161	-0.37822896	0.001191895	-1.6994056	0.026886428	0.973	5595	tags=40%, list=30%, signal=57%	FALSE
SIG_CHEMOTAXIS	SIG_CHEMOTAXIS		44	-0.46096733	0.004329004	-1.698477	0.027014686	0.975	2069	tags=30%, list=11%, signal=33%	FALSE
REACTOME_HOMOLOGOUS_RECOMBINATION_REPAIR_OF_REPLICATION_INDEPENDENT_DOUBLE_STRAND_BREAKS	REACTOME_HOMOLOGOUS_RECOMBINATION_REPAIR_OF_REPLICATION_INDEPENDENT_DOUBLE_STRAND_BREAKS		16	-0.5894746	0.00921659	-1.696996	0.0271771	0.977	4414	tags=63%, list=23%, signal=81%	FALSE
BIOCARTA_FCER1_PATHWAY	BIOCARTA_FCER1_PATHWAY		38	-0.4776544	0.001459854	-1.6959115	0.027234972	0.979	1632	tags=29%, list=9%, signal=32%	FALSE
PID_HEDGEHOG_GLI_PATHWAY	PID_HEDGEHOG_GLI_PATHWAY		48	-0.44944715	0.006963789	-1.688697	0.0290394	0.987	5881	tags=60%, list=31%, signal=87%	FALSE
REACTOME_RNA_POL_I_TRANSCRIPTION_INITIATION	REACTOME_RNA_POL_I_TRANSCRIPTION_INITIATION		21	-0.55485076	0.01875	-1.6752243	0.03311361	0.993	3075	tags=38%, list=16%, signal=45%	FALSE
REACTOME_GENERIC_TRANSCRIPTION_PATHWAY	REACTOME_GENERIC_TRANSCRIPTION_PATHWAY		320	-0.34344044	0	-1.6704705	0.03441476	0.994	5486	tags=44%, list=29%, signal=61%	FALSE
BIOCARTA_ATRBRCA_PATHWAY	BIOCARTA_ATRBRCA_PATHWAY		20	-0.5575306	0.009360374	-1.6683583	0.034830555	0.995	4414	tags=50%, list=23%, signal=65%	FALSE
REACTOME_MITOCHONDRIAL_PROTEIN_IMPORT	REACTOME_MITOCHONDRIAL_PROTEIN_IMPORT		42	-0.45394748	0.005706134	-1.6676946	0.034789454	0.996	7647	tags=74%, list=40%, signal=124%	FALSE
REACTOME_LAGGING_STRAND_SYNTHESIS	REACTOME_LAGGING_STRAND_SYNTHESIS		19	-0.5596451	0.009244992	-1.666878	0.034775883	0.996	4338	tags=53%, list=23%, signal=68%	FALSE
REACTOME_RNA_POL_II_TRANSCRIPTION_PRE_INITIATION_AND_PROMOTER_OPENING	REACTOME_RNA_POL_II_TRANSCRIPTION_PRE_INITIATION_AND_PROMOTER_OPENING		37	-0.47256085	0.005797102	-1.6667995	0.03451581	0.996	6831	tags=62%, list=36%, signal=97%	FALSE
KEGG_BASAL_TRANSCRIPTION_FACTORS	KEGG_BASAL_TRANSCRIPTION_FACTORS		31	-0.49238983	0.009063444	-1.66531	0.034695454	0.996	3267	tags=42%, list=17%, signal=51%	FALSE
REACTOME_ANTIVIRAL_MECHANISM_BY_IFN_STIMULATED_GENES	REACTOME_ANTIVIRAL_MECHANISM_BY_IFN_STIMULATED_GENES		62	-0.42077273	0.002688172	-1.6572164	0.037236713	0.996	5503	tags=48%, list=29%, signal=68%	FALSE
HALLMARK_DNA_REPAIR	HALLMARK_DNA_REPAIR		144	-0.36911497	0	-1.655301	0.03769975	0.996	4490	tags=39%, list=24%, signal=51%	FALSE
REACTOME_OXYGEN_DEPENDENT_PROLINE_HYDROXYLATION_OF_HYPOXIA_INDUCIBLE_FACTOR_ALPHA	REACTOME_OXYGEN_DEPENDENT_PROLINE_HYDROXYLATION_OF_HYPOXIA_INDUCIBLE_FACTOR_ALPHA		15	-0.59563786	0.0112	-1.6538758	0.03789076	0.996	2621	tags=47%, list=14%, signal=54%	FALSE
PID_AR_TF_PATHWAY	PID_AR_TF_PATHWAY		52	-0.43449935	0.006738544	-1.6494391	0.039446857	0.996	5674	tags=52%, list=30%, signal=74%	FALSE
BIOCARTA_ARF_PATHWAY	BIOCARTA_ARF_PATHWAY		17	-0.5576744	0.015600624	-1.6470369	0.03998756	0.997	4591	tags=59%, list=24%, signal=78%	FALSE
PID_ERB_GENOMIC_PATHWAY	PID_ERB_GENOMIC_PATHWAY		15	-0.583657	0.022617124	-1.6427146	0.041622322	0.998	5580	tags=67%, list=30%, signal=95%	FALSE
BIOCARTA_EIF_PATHWAY	BIOCARTA_EIF_PATHWAY		15	-0.5800274	0.016819572	-1.6391567	0.042692557	0.998	1739	tags=40%, list=9%, signal=44%	FALSE
PID_ATM_PATHWAY	PID_ATM_PATHWAY		33	-0.4776773	0.01969697	-1.6332444	0.04466849	0.999	4150	tags=45%, list=22%, signal=58%	FALSE
REACTOME_REGULATORY_RNA_PATHWAYS	REACTOME_REGULATORY_RNA_PATHWAYS		24	-0.50893354	0.012195122	-1.6305248	0.045373447	0.999	3406	tags=38%, list=18%, signal=46%	FALSE
BIOCARTA_CARM_ER_PATHWAY	BIOCARTA_CARM_ER_PATHWAY		34	-0.47130957	0.010279002	-1.6243876	0.047836028	0.999	4562	tags=53%, list=24%, signal=70%	FALSE
PID_CMYB_PATHWAY	PID_CMYB_PATHWAY		83	-0.39359203	0.005174644	-1.6163135	0.0509457	0.999	3301	tags=36%, list=17%, signal=44%	FALSE
REACTOME_FORMATION_OF_INCISION_COMPLEX_IN_GG_NER	REACTOME_FORMATION_OF_INCISION_COMPLEX_IN_GG_NER		17	-0.54411674	0.022508038	-1.6162341	0.050604906	0.999	5803	tags=59%, list=31%, signal=85%	FALSE
REACTOME_TRAF6_MEDIATED_IRF7_ACTIVATION	REACTOME_TRAF6_MEDIATED_IRF7_ACTIVATION		26	-0.4958586	0.011976048	-1.6143435	0.051110145	0.999	5943	tags=62%, list=31%, signal=90%	FALSE
REACTOME_HOST_INTERACTIONS_OF_HIV_FACTORS	REACTOME_HOST_INTERACTIONS_OF_HIV_FACTORS		115	-0.37130827	0.002522068	-1.606495	0.054526873	0.999	6823	tags=49%, list=36%, signal=76%	FALSE
BIOCARTA_CTLA4_PATHWAY	BIOCARTA_CTLA4_PATHWAY		19	-0.532431	0.0152207	-1.6046537	0.055108994	0.999	7475	tags=74%, list=40%, signal=122%	FALSE
REACTOME_PROCESSIVE_SYNTHESIS_ON_THE_LAGGING_STRAND	REACTOME_PROCESSIVE_SYNTHESIS_ON_THE_LAGGING_STRAND		15	-0.55906844	0.021671826	-1.6034039	0.055428915	0.999	4338	tags=53%, list=23%, signal=69%	FALSE
REACTOME_G1_S_SPECIFIC_TRANSCRIPTION	REACTOME_G1_S_SPECIFIC_TRANSCRIPTION		16	-0.5593815	0.03069467	-1.5973135	0.057922456	0.999	6821	tags=75%, list=36%, signal=117%	FALSE
SA_PTEN_PATHWAY	SA_PTEN_PATHWAY		17	-0.5479056	0.028744327	-1.5973029	0.057523165	0.999	1999	tags=35%, list=11%, signal=39%	FALSE
REACTOME_RIG_I_MDA5_MEDIATED_INDUCTION_OF_IFN_ALPHA_BETA_PATHWAYS	REACTOME_RIG_I_MDA5_MEDIATED_INDUCTION_OF_IFN_ALPHA_BETA_PATHWAYS		66	-0.40066305	0.0067659	-1.5954138	0.05814197	0.999	5943	tags=55%, list=31%, signal=79%	FALSE
BIOCARTA_BCR_PATHWAY	BIOCARTA_BCR_PATHWAY		34	-0.4617592	0.017118402	-1.5848917	0.06344437	1	1632	tags=29%, list=9%, signal=32%	FALSE
REACTOME_TCA_CYCLE_AND_RESPIRATORY_ELECTRON_TRANSPORT	REACTOME_TCA_CYCLE_AND_RESPIRATORY_ELECTRON_TRANSPORT		106	-0.37015462	0.00655308	-1.5813459	0.065030836	1	7033	tags=54%, list=37%, signal=85%	FALSE
REACTOME_ANTIGEN_PROCESSING_UBIQUITINATION_PROTEASOME_DEGRADATION	REACTOME_ANTIGEN_PROCESSING_UBIQUITINATION_PROTEASOME_DEGRADATION		190	-0.34482875	0	-1.581298	0.06460548	1	6401	tags=45%, list=34%, signal=67%	FALSE
REACTOME_ELONGATION_ARREST_AND_RECOVERY	REACTOME_ELONGATION_ARREST_AND_RECOVERY		30	-0.47144663	0.015536723	-1.5779501	0.06605395	1	7327	tags=77%, list=39%, signal=125%	FALSE
REACTOME_G1_PHASE	REACTOME_G1_PHASE		34	-0.45321733	0.016516516	-1.5736846	0.068115614	1	5547	tags=56%, list=29%, signal=79%	FALSE
KEGG_B_CELL_RECEPTOR_SIGNALING_PATHWAY	KEGG_B_CELL_RECEPTOR_SIGNALING_PATHWAY		74	-0.39214107	0.007633588	-1.5729394	0.06814361	1	2165	tags=27%, list=11%, signal=30%	FALSE
PID_TELOMERASE_PATHWAY	PID_TELOMERASE_PATHWAY		67	-0.3983901	0.010738255	-1.5726037	0.06796732	1	6491	tags=58%, list=34%, signal=88%	FALSE
REACTOME_CHROMOSOME_MAINTENANCE	REACTOME_CHROMOSOME_MAINTENANCE		101	-0.36766347	0.00511509	-1.5694656	0.06922221	1	6393	tags=55%, list=34%, signal=83%	FALSE
PID_RB_1PATHWAY	PID_RB_1PATHWAY		64	-0.40281174	0.010840109	-1.566699	0.070454285	1	5292	tags=55%, list=28%, signal=76%	FALSE
BIOCARTA_CERAMIDE_PATHWAY	BIOCARTA_CERAMIDE_PATHWAY		22	-0.5033742	0.0311042	-1.5646292	0.071144186	1	1274	tags=27%, list=7%, signal=29%	FALSE
REACTOME_FORMATION_OF_THE_HIV1_EARLY_ELONGATION_COMPLEX	REACTOME_FORMATION_OF_THE_HIV1_EARLY_ELONGATION_COMPLEX		30	-0.45956984	0.022488756	-1.5621551	0.07226906	1	7327	tags=70%, list=39%, signal=114%	FALSE
BIOCARTA_IL3_PATHWAY	BIOCARTA_IL3_PATHWAY		15	-0.53693694	0.024509804	-1.5610015	0.07246324	1	2765	tags=33%, list=15%, signal=39%	FALSE
REACTOME_BRANCHED_CHAIN_AMINO_ACID_CATABOLISM	REACTOME_BRANCHED_CHAIN_AMINO_ACID_CATABOLISM		17	-0.5308682	0.03610675	-1.5593309	0.07314245	1	5374	tags=53%, list=28%, signal=74%	FALSE
REACTOME_UNBLOCKING_OF_NMDA_RECEPTOR_GLUTAMATE_BINDING_AND_ACTIVATION	REACTOME_UNBLOCKING_OF_NMDA_RECEPTOR_GLUTAMATE_BINDING_AND_ACTIVATION		15	-0.5500115	0.0311042	-1.5532115	0.07640072	1	3997	tags=40%, list=21%, signal=51%	FALSE
BIOCARTA_PITX2_PATHWAY	BIOCARTA_PITX2_PATHWAY		15	-0.55942047	0.033707865	-1.5478779	0.07914947	1	3132	tags=53%, list=17%, signal=64%	FALSE
PID_HDAC_CLASSI_PATHWAY	PID_HDAC_CLASSI_PATHWAY		64	-0.39088887	0.011004127	-1.5466888	0.079353906	1	4973	tags=47%, list=26%, signal=63%	FALSE
REACTOME_ACTIVATION_OF_GENES_BY_ATF4	REACTOME_ACTIVATION_OF_GENES_BY_ATF4		23	-0.49662584	0.027900146	-1.5459983	0.079346135	1	4191	tags=52%, list=22%, signal=67%	FALSE
REACTOME_BASE_EXCISION_REPAIR	REACTOME_BASE_EXCISION_REPAIR		18	-0.5254964	0.032659408	-1.5447778	0.07976394	1	2103	tags=39%, list=11%, signal=44%	FALSE
REACTOME_ABORTIVE_ELONGATION_OF_HIV1_TRANSCRIPT_IN_THE_ABSENCE_OF_TAT	REACTOME_ABORTIVE_ELONGATION_OF_HIV1_TRANSCRIPT_IN_THE_ABSENCE_OF_TAT		23	-0.48375592	0.03259259	-1.5417979	0.081355385	1	3281	tags=43%, list=17%, signal=53%	FALSE
BIOCARTA_CTCF_PATHWAY	BIOCARTA_CTCF_PATHWAY		23	-0.48486453	0.031484257	-1.5412239	0.08123603	1	2368	tags=35%, list=13%, signal=40%	FALSE
BIOCARTA_NO2IL12_PATHWAY	BIOCARTA_NO2IL12_PATHWAY		17	-0.539007	0.022047244	-1.5407641	0.08106446	1	8243	tags=82%, list=44%, signal=146%	FALSE
PID_ERA_GENOMIC_PATHWAY	PID_ERA_GENOMIC_PATHWAY		64	-0.38861215	0.008130081	-1.5304966	0.0874076	1	4562	tags=44%, list=24%, signal=57%	FALSE
REACTOME_ASSEMBLY_OF_THE_PRE_REPLICATIVE_COMPLEX	REACTOME_ASSEMBLY_OF_THE_PRE_REPLICATIVE_COMPLEX		60	-0.3950092	0.015320335	-1.5300655	0.08716332	1	3144	tags=27%, list=17%, signal=32%	FALSE
PID_IL3_PATHWAY	PID_IL3_PATHWAY		27	-0.4652481	0.038011696	-1.5291508	0.087225206	1	2926	tags=41%, list=15%, signal=48%	FALSE
REACTOME_PERK_REGULATED_GENE_EXPRESSION	REACTOME_PERK_REGULATED_GENE_EXPRESSION		26	-0.47141668	0.030927835	-1.5227274	0.091437034	1	4668	tags=54%, list=25%, signal=71%	FALSE
BIOCARTA_SHH_PATHWAY	BIOCARTA_SHH_PATHWAY		16	-0.53353196	0.037936267	-1.5225618	0.09102989	1	5881	tags=50%, list=31%, signal=73%	FALSE
BIOCARTA_CSK_PATHWAY	BIOCARTA_CSK_PATHWAY		22	-0.4921644	0.032307692	-1.5208724	0.09175884	1	8335	tags=77%, list=44%, signal=138%	FALSE
REACTOME_E2F_MEDIATED_REGULATION_OF_DNA_REPLICATION	REACTOME_E2F_MEDIATED_REGULATION_OF_DNA_REPLICATION		32	-0.45571062	0.028985508	-1.520611	0.09143752	1	5698	tags=50%, list=30%, signal=71%	FALSE
BIOCARTA_G2_PATHWAY	BIOCARTA_G2_PATHWAY		22	-0.47714716	0.03405573	-1.5139244	0.09589223	1	2797	tags=36%, list=15%, signal=43%	FALSE
PID_P38_MKK3_6PATHWAY	PID_P38_MKK3_6PATHWAY		26	-0.4628247	0.03259259	-1.5123448	0.096615836	1	2539	tags=31%, list=13%, signal=35%	FALSE
PID_TXA2PATHWAY	PID_TXA2PATHWAY		56	-0.3887677	0.016877636	-1.5109199	0.09715292	1	2859	tags=32%, list=15%, signal=38%	FALSE
PID_IL2_STAT5_PATHWAY	PID_IL2_STAT5_PATHWAY		30	-0.45010608	0.034638554	-1.5079039	0.098841086	1	5392	tags=47%, list=29%, signal=65%	FALSE
PID_REG_GR_PATHWAY	PID_REG_GR_PATHWAY		80	-0.36385876	0.014666666	-1.5062361	0.09962972	1	3788	tags=34%, list=20%, signal=42%	FALSE
BIOCARTA_ERK5_PATHWAY	BIOCARTA_ERK5_PATHWAY		17	-0.5237855	0.05148206	-1.5003994	0.10364125	1	5230	tags=53%, list=28%, signal=73%	FALSE
HALLMARK_OXIDATIVE_PHOSPHORYLATION	HALLMARK_OXIDATIVE_PHOSPHORYLATION		188	-0.32798204	0.002361275	-1.498561	0.10438202	1	7053	tags=54%, list=37%, signal=86%	FALSE
KEGG_PURINE_METABOLISM	KEGG_PURINE_METABOLISM		152	-0.33202526	0.003787879	-1.4943295	0.10722567	1	4490	tags=32%, list=24%, signal=41%	FALSE
BIOCARTA_BAD_PATHWAY	BIOCARTA_BAD_PATHWAY		26	-0.45733336	0.050925925	-1.4914998	0.10893897	1	2797	tags=31%, list=15%, signal=36%	FALSE
BIOCARTA_WNT_PATHWAY	BIOCARTA_WNT_PATHWAY		26	-0.45616817	0.04477612	-1.4903914	0.109278545	1	3393	tags=46%, list=18%, signal=56%	FALSE
REACTOME_METABOLISM_OF_NUCLEOTIDES	REACTOME_METABOLISM_OF_NUCLEOTIDES		68	-0.3769255	0.021917809	-1.4892234	0.109678425	1	4102	tags=37%, list=22%, signal=47%	FALSE
REACTOME_REGULATION_OF_MRNA_STABILITY_BY_PROTEINS_THAT_BIND_AU_RICH_ELEMENTS	REACTOME_REGULATION_OF_MRNA_STABILITY_BY_PROTEINS_THAT_BIND_AU_RICH_ELEMENTS		75	-0.3696348	0.024657534	-1.487283	0.11081855	1	3824	tags=31%, list=20%, signal=38%	FALSE
REACTOME_TRANSCRIPTIONAL_ACTIVITY_OF_SMAD2_SMAD3_SMAD4_HETEROTRIMER	REACTOME_TRANSCRIPTIONAL_ACTIVITY_OF_SMAD2_SMAD3_SMAD4_HETEROTRIMER		36	-0.42455342	0.03259259	-1.4872032	0.11027008	1	6019	tags=58%, list=32%, signal=85%	FALSE
REACTOME_CIRCADIAN_CLOCK	REACTOME_CIRCADIAN_CLOCK		52	-0.39279422	0.033195022	-1.4862255	0.11053087	1	3303	tags=40%, list=17%, signal=49%	FALSE
REACTOME_RECRUITMENT_OF_MITOTIC_CENTROSOME_PROTEINS_AND_COMPLEXES	REACTOME_RECRUITMENT_OF_MITOTIC_CENTROSOME_PROTEINS_AND_COMPLEXES		62	-0.37793863	0.019858155	-1.4820304	0.11330747	1	5249	tags=45%, list=28%, signal=62%	FALSE
REACTOME_ORC1_REMOVAL_FROM_CHROMATIN	REACTOME_ORC1_REMOVAL_FROM_CHROMATIN		62	-0.37275952	0.023904383	-1.4786557	0.11542224	1	3144	tags=26%, list=17%, signal=31%	FALSE
BIOCARTA_TEL_PATHWAY	BIOCARTA_TEL_PATHWAY		17	-0.5006306	0.05414013	-1.4744588	0.11836163	1	3799	tags=53%, list=20%, signal=66%	FALSE
REACTOME_SYNTHESIS_AND_INTERCONVERSION_OF_NUCLEOTIDE_DI_AND_TRIPHOSPHATES	REACTOME_SYNTHESIS_AND_INTERCONVERSION_OF_NUCLEOTIDE_DI_AND_TRIPHOSPHATES		17	-0.49922135	0.0576	-1.472563	0.119294174	1	6821	tags=71%, list=36%, signal=110%	FALSE
SIG_PIP3_SIGNALING_IN_B_LYMPHOCYTES	SIG_PIP3_SIGNALING_IN_B_LYMPHOCYTES		36	-0.4242128	0.033236995	-1.4713556	0.1197024	1	2797	tags=31%, list=15%, signal=36%	FALSE
PID_HES_HEY_PATHWAY	PID_HES_HEY_PATHWAY		46	-0.39509463	0.040446304	-1.4650071	0.12489057	1	3212	tags=37%, list=17%, signal=44%	FALSE
BIOCARTA_VIP_PATHWAY	BIOCARTA_VIP_PATHWAY		25	-0.45227483	0.052941177	-1.4626747	0.12643799	1	5905	tags=56%, list=31%, signal=81%	FALSE
REACTOME_FACTORS_INVOLVED_IN_MEGAKARYOCYTE_DEVELOPMENT_AND_PLATELET_PRODUCTION	REACTOME_FACTORS_INVOLVED_IN_MEGAKARYOCYTE_DEVELOPMENT_AND_PLATELET_PRODUCTION		114	-0.34181273	0.018891688	-1.4621036	0.12636814	1	3706	tags=30%, list=20%, signal=37%	FALSE
BIOCARTA_MTOR_PATHWAY	BIOCARTA_MTOR_PATHWAY		21	-0.47036204	0.072981365	-1.4616407	0.1261343	1	2137	tags=24%, list=11%, signal=27%	FALSE
KEGG_CYTOSOLIC_DNA_SENSING_PATHWAY	KEGG_CYTOSOLIC_DNA_SENSING_PATHWAY		48	-0.3929314	0.02503477	-1.4566126	0.13031235	1	3810	tags=35%, list=20%, signal=44%	FALSE
REACTOME_RNA_POL_I_TRANSCRIPTION_TERMINATION	REACTOME_RNA_POL_I_TRANSCRIPTION_TERMINATION		18	-0.49169457	0.06386555	-1.45592	0.1304136	1	6042	tags=56%, list=32%, signal=82%	FALSE
REACTOME_GLUCOSE_TRANSPORT	REACTOME_GLUCOSE_TRANSPORT		37	-0.4095489	0.04918033	-1.4523734	0.13304658	1	2912	tags=30%, list=15%, signal=35%	FALSE
REACTOME_MEIOSIS	REACTOME_MEIOSIS		86	-0.3505795	0.02356021	-1.4500114	0.13465315	1	4647	tags=43%, list=25%, signal=57%	FALSE
PID_IL8_CXCR2_PATHWAY	PID_IL8_CXCR2_PATHWAY		34	-0.41150802	0.06013986	-1.4491107	0.13486224	1	1389	tags=24%, list=7%, signal=25%	FALSE
REACTOME_CREB_PHOSPHORYLATION_THROUGH_THE_ACTIVATION_OF_CAMKII	REACTOME_CREB_PHOSPHORYLATION_THROUGH_THE_ACTIVATION_OF_CAMKII		15	-0.5066057	0.06944445	-1.4473078	0.1360767	1	3997	tags=40%, list=21%, signal=51%	FALSE
REACTOME_NUCLEOTIDE_LIKE_PURINERGIC_RECEPTORS	REACTOME_NUCLEOTIDE_LIKE_PURINERGIC_RECEPTORS		15	-0.5179431	0.060150377	-1.4472958	0.13541162	1	4784	tags=47%, list=25%, signal=62%	FALSE
REACTOME_FORMATION_OF_TRANSCRIPTION_COUPLED_NER_TC_NER_REPAIR_COMPLEX	REACTOME_FORMATION_OF_TRANSCRIPTION_COUPLED_NER_TC_NER_REPAIR_COMPLEX		24	-0.458608	0.05029586	-1.4463363	0.13573332	1	7708	tags=63%, list=41%, signal=105%	FALSE
REACTOME_MITOTIC_PROMETAPHASE	REACTOME_MITOTIC_PROMETAPHASE		82	-0.34855142	0.015665796	-1.438471	0.14284644	1	4973	tags=37%, list=26%, signal=49%	FALSE
PID_RAC1_REG_PATHWAY	PID_RAC1_REG_PATHWAY		37	-0.40381017	0.05467626	-1.4369794	0.14370884	1	1865	tags=24%, list=10%, signal=27%	FALSE
PID_RETINOIC_ACID_PATHWAY	PID_RETINOIC_ACID_PATHWAY		30	-0.42066106	0.05029586	-1.4354578	0.14462821	1	3909	tags=33%, list=21%, signal=42%	FALSE
REACTOME_DAG_AND_IP3_SIGNALING	REACTOME_DAG_AND_IP3_SIGNALING		31	-0.42788166	0.05124451	-1.4345347	0.1449054	1	5230	tags=42%, list=28%, signal=58%	FALSE
PID_NFAT_3PATHWAY	PID_NFAT_3PATHWAY		52	-0.3824954	0.028208744	-1.4294745	0.14971597	1	4205	tags=33%, list=22%, signal=42%	FALSE
REACTOME_IL_RECEPTOR_SHC_SIGNALING	REACTOME_IL_RECEPTOR_SHC_SIGNALING		27	-0.4368621	0.076323986	-1.4282554	0.15040298	1	2038	tags=22%, list=11%, signal=25%	FALSE
REACTOME_PLATELET_CALCIUM_HOMEOSTASIS	REACTOME_PLATELET_CALCIUM_HOMEOSTASIS		16	-0.49211454	0.0859375	-1.4270629	0.15094319	1	2758	tags=31%, list=15%, signal=37%	FALSE
PID_IL2_PI3K_PATHWAY	PID_IL2_PI3K_PATHWAY		34	-0.41076458	0.05671642	-1.4259982	0.15136707	1	2488	tags=32%, list=13%, signal=37%	FALSE
PID_KIT_PATHWAY	PID_KIT_PATHWAY		52	-0.37335092	0.033898305	-1.4215429	0.1553463	1	2488	tags=23%, list=13%, signal=27%	FALSE
REACTOME_THE_ROLE_OF_NEF_IN_HIV1_REPLICATION_AND_DISEASE_PATHOGENESIS	REACTOME_THE_ROLE_OF_NEF_IN_HIV1_REPLICATION_AND_DISEASE_PATHOGENESIS		27	-0.42964286	0.05865922	-1.4179285	0.15881415	1	1333	tags=22%, list=7%, signal=24%	FALSE
BIOCARTA_P53_PATHWAY	BIOCARTA_P53_PATHWAY		16	-0.49622375	0.07350689	-1.4179226	0.15807202	1	3938	tags=50%, list=21%, signal=63%	FALSE
KEGG_OLFACTORY_TRANSDUCTION	KEGG_OLFACTORY_TRANSDUCTION		107	-0.3277667	0.028350515	-1.4170938	0.15817463	1	3721	tags=28%, list=20%, signal=35%	FALSE
BIOCARTA_VEGF_PATHWAY	BIOCARTA_VEGF_PATHWAY		29	-0.42643175	0.06983655	-1.4160826	0.15851839	1	4668	tags=52%, list=25%, signal=69%	FALSE
REACTOME_OLFACTORY_SIGNALING_PATHWAY	REACTOME_OLFACTORY_SIGNALING_PATHWAY		72	-0.35023075	0.047106326	-1.4140223	0.15993677	1	3721	tags=33%, list=20%, signal=41%	FALSE
BIOCARTA_IGF1MTOR_PATHWAY	BIOCARTA_IGF1MTOR_PATHWAY		20	-0.47220394	0.075038284	-1.4125662	0.16089503	1	4668	tags=45%, list=25%, signal=60%	FALSE
BIOCARTA_TCR_PATHWAY	BIOCARTA_TCR_PATHWAY		44	-0.38549453	0.046767537	-1.4109505	0.16205154	1	1673	tags=25%, list=9%, signal=27%	FALSE
BIOCARTA_HDAC_PATHWAY	BIOCARTA_HDAC_PATHWAY		28	-0.42894098	0.07589286	-1.4107262	0.16157845	1	5905	tags=50%, list=31%, signal=73%	FALSE
REACTOME_TRAF6_MEDIATED_INDUCTION_OF_NFKB_AND_MAP_KINASES_UPON_TLR7_8_OR_9_ACTIVATION	REACTOME_TRAF6_MEDIATED_INDUCTION_OF_NFKB_AND_MAP_KINASES_UPON_TLR7_8_OR_9_ACTIVATION		72	-0.3532275	0.03601108	-1.4097326	0.16209352	1	4603	tags=36%, list=24%, signal=48%	FALSE
KEGG_OXIDATIVE_PHOSPHORYLATION	KEGG_OXIDATIVE_PHOSPHORYLATION		107	-0.32895574	0.032425422	-1.4066986	0.16495822	1	6741	tags=50%, list=36%, signal=78%	FALSE
REACTOME_NOTCH1_INTRACELLULAR_DOMAIN_REGULATES_TRANSCRIPTION	REACTOME_NOTCH1_INTRACELLULAR_DOMAIN_REGULATES_TRANSCRIPTION		44	-0.38486987	0.07573633	-1.4032189	0.16799164	1	6061	tags=59%, list=32%, signal=87%	FALSE
HALLMARK_ALLOGRAFT_REJECTION	HALLMARK_ALLOGRAFT_REJECTION		196	-0.30183727	0.010625738	-1.4015697	0.16930936	1	7858	tags=57%, list=42%, signal=96%	FALSE
REACTOME_CLASS_I_MHC_MEDIATED_ANTIGEN_PROCESSING_PRESENTATION	REACTOME_CLASS_I_MHC_MEDIATED_ANTIGEN_PROCESSING_PRESENTATION		224	-0.29576516	0.008073818	-1.3943835	0.17725515	1	4200	tags=29%, list=22%, signal=36%	FALSE
REACTOME_SMAD2_SMAD3_SMAD4_HETEROTRIMER_REGULATES_TRANSCRIPTION	REACTOME_SMAD2_SMAD3_SMAD4_HETEROTRIMER_REGULATES_TRANSCRIPTION		25	-0.44093117	0.074484944	-1.3927523	0.17849624	1	4066	tags=48%, list=22%, signal=61%	FALSE
PID_FCER1_PATHWAY	PID_FCER1_PATHWAY		61	-0.35495657	0.055478502	-1.3925941	0.17791535	1	2030	tags=25%, list=11%, signal=27%	FALSE
REACTOME_NEGATIVE_REGULATORS_OF_RIG_I_MDA5_SIGNALING	REACTOME_NEGATIVE_REGULATORS_OF_RIG_I_MDA5_SIGNALING		30	-0.41657826	0.08066971	-1.3906282	0.17958656	1	5943	tags=57%, list=31%, signal=83%	FALSE
REACTOME_MICRORNA_MIRNA_BIOGENESIS	REACTOME_MICRORNA_MIRNA_BIOGENESIS		21	-0.44472656	0.08042488	-1.3899795	0.17958836	1	2952	tags=29%, list=16%, signal=34%	FALSE
PID_MYC_PATHWAY	PID_MYC_PATHWAY		25	-0.43473706	0.08592593	-1.3894129	0.17944375	1	5874	tags=56%, list=31%, signal=81%	FALSE
KEGG_TASTE_TRANSDUCTION	KEGG_TASTE_TRANSDUCTION		42	-0.38800973	0.058739256	-1.3884704	0.17972802	1	6229	tags=43%, list=33%, signal=64%	FALSE
KEGG_AMINOACYL_TRNA_BIOSYNTHESIS	KEGG_AMINOACYL_TRNA_BIOSYNTHESIS		41	-0.38855648	0.06540698	-1.3869109	0.18078065	1	6559	tags=49%, list=35%, signal=75%	FALSE
REACTOME_MITOTIC_G2_G2_M_PHASES	REACTOME_MITOTIC_G2_G2_M_PHASES		77	-0.3434109	0.055916775	-1.3853742	0.18192248	1	5698	tags=45%, list=30%, signal=65%	FALSE
REACTOME_ACTIVATED_TLR4_SIGNALLING	REACTOME_ACTIVATED_TLR4_SIGNALLING		87	-0.33345103	0.034300793	-1.3801669	0.18769296	1	3810	tags=29%, list=20%, signal=36%	FALSE
REACTOME_IL_3_5_AND_GM_CSF_SIGNALING	REACTOME_IL_3_5_AND_GM_CSF_SIGNALING		43	-0.37865052	0.081277214	-1.3786956	0.18865167	1	4591	tags=35%, list=24%, signal=46%	FALSE
REACTOME_CA_DEPENDENT_EVENTS	REACTOME_CA_DEPENDENT_EVENTS		29	-0.4129518	0.08104197	-1.3782072	0.18849611	1	3203	tags=28%, list=17%, signal=33%	FALSE
REACTOME_MYD88_MAL_CASCADE_INITIATED_ON_PLASMA_MEMBRANE	REACTOME_MYD88_MAL_CASCADE_INITIATED_ON_PLASMA_MEMBRANE		78	-0.34513795	0.08725415	-1.3777375	0.18819875	1	3810	tags=29%, list=20%, signal=36%	FALSE
PID_CDC42_REG_PATHWAY	PID_CDC42_REG_PATHWAY		30	-0.4078636	0.07121662	-1.3767518	0.18862343	1	7182	tags=57%, list=38%, signal=91%	FALSE
SIG_IL4RECEPTOR_IN_B_LYPHOCYTES	SIG_IL4RECEPTOR_IN_B_LYPHOCYTES		27	-0.41851318	0.09379724	-1.3736827	0.19186667	1	1913	tags=22%, list=10%, signal=25%	FALSE
PID_SMAD2_3NUCLEAR_PATHWAY	PID_SMAD2_3NUCLEAR_PATHWAY		81	-0.3328342	0.04915912	-1.3698047	0.19627576	1	3337	tags=35%, list=18%, signal=42%	FALSE
REACTOME_CYTOKINE_SIGNALING_IN_IMMUNE_SYSTEM	REACTOME_CYTOKINE_SIGNALING_IN_IMMUNE_SYSTEM		248	-0.28573045	0.021468926	-1.3622074	0.20558003	1	5503	tags=43%, list=29%, signal=59%	FALSE
KEGG_ALZHEIMERS_DISEASE	KEGG_ALZHEIMERS_DISEASE		148	-0.30602047	0.025990099	-1.3604865	0.20713507	1	6756	tags=46%, list=36%, signal=71%	FALSE
REACTOME_LOSS_OF_NLP_FROM_MITOTIC_CENTROSOMES	REACTOME_LOSS_OF_NLP_FROM_MITOTIC_CENTROSOMES		55	-0.35362568	0.06910569	-1.3582418	0.20946786	1	5249	tags=40%, list=28%, signal=55%	FALSE
PID_AR_PATHWAY	PID_AR_PATHWAY		58	-0.35274938	0.0728022	-1.3549224	0.21305574	1	6269	tags=59%, list=33%, signal=88%	FALSE
ST_JNK_MAPK_PATHWAY	ST_JNK_MAPK_PATHWAY		37	-0.37118334	0.08250156	-1.3499023	0.21889056	1	5370	tags=49%, list=28%, signal=68%	FALSE
PID_PI3KCI_PATHWAY	PID_PI3KCI_PATHWAY		49	-0.3640419	0.07386363	-1.3493605	0.21870881	1	2857	tags=24%, list=15%, signal=29%	FALSE
PID_TCR_PATHWAY	PID_TCR_PATHWAY		65	-0.3432379	0.06507304	-1.3481424	0.21948352	1	7068	tags=52%, list=37%, signal=83%	FALSE
KEGG_ACUTE_MYELOID_LEUKEMIA	KEGG_ACUTE_MYELOID_LEUKEMIA		56	-0.35686358	0.0651289	-1.3454945	0.22222963	1	4753	tags=32%, list=25%, signal=43%	FALSE
REACTOME_TOLL_RECEPTOR_CASCADES	REACTOME_TOLL_RECEPTOR_CASCADES		110	-0.31345107	0.05276382	-1.3431167	0.22467405	1	4613	tags=32%, list=24%, signal=42%	FALSE
REACTOME_INTERFERON_SIGNALING	REACTOME_INTERFERON_SIGNALING		140	-0.30033112	0.043639363	-1.3404932	0.22747384	1	4584	tags=33%, list=24%, signal=43%	FALSE
REACTOME_TRNA_AMINOACYLATION	REACTOME_TRNA_AMINOACYLATION		42	-0.36923313	0.08416547	-1.3392742	0.22816157	1	3830	tags=31%, list=20%, signal=39%	FALSE
REACTOME_RESOLUTION_OF_AP_SITES_VIA_THE_MULTIPLE_NUCLEOTIDE_PATCH_REPLACEMENT_PATHWAY	REACTOME_RESOLUTION_OF_AP_SITES_VIA_THE_MULTIPLE_NUCLEOTIDE_PATCH_REPLACEMENT_PATHWAY		16	-0.45874	0.11353032	-1.3377766	0.22990177	1	1802	tags=19%, list=10%, signal=21%	FALSE
REACTOME_SIGNALING_BY_FGFR1_FUSION_MUTANTS	REACTOME_SIGNALING_BY_FGFR1_FUSION_MUTANTS		17	-0.45731482	0.13607594	-1.3362439	0.22968043	1	2870	tags=47%, list=15%, signal=55%	FALSE
BIOCARTA_IL2RB_PATHWAY	BIOCARTA_IL2RB_PATHWAY		38	-0.37960035	0.09836066	-1.3362486	0.22968558	1	2765	tags=29%, list=15%, signal=34%	FALSE

KEGG_MTOR_SIGNALING_PATHWAY	KEGG_MTOR_SIGNALING_PATHWAY		49	-0.35793945	0.08450704	-1.3356634	0.22979875	1	2137	tags=18%, list=11%, signal=21%	FALSE
REACTOME_ENERGY_DEPENDENT_REGULATION_OF_MTOR_BY_LKB1_AMPK	REACTOME_ENERGY_DEPENDENT_REGULATION_OF_MTOR_BY_LKB1_AMPK		16	-0.46048182	0.121069185	-1.3353837	0.22931252	1	1664	tags=19%, list=9%, signal=21%	FALSE
BIOCARTA_LAIR_PATHWAY	BIOCARTA_LAIR_PATHWAY		16	-0.46552163	0.13050315	-1.3304088	0.23560683	1	1589	tags=25%, list=8%, signal=27%	FALSE
REACTOME_TELOMERE_MAINTENANCE	REACTOME_TELOMERE_MAINTENANCE		59	-0.34165007	0.09090909	-1.32486	0.242864	1	6569	tags=59%, list=35%, signal=91%	FALSE
PID_AURORA_B_PATHWAY	PID_AURORA_B_PATHWAY		39	-0.37029168	0.10678211	-1.3213125	0.24721749	1	6500	tags=41%, list=34%, signal=62%	FALSE
KEGG_OOCYTE_MEIOSIS	KEGG_OOCYTE_MEIOSIS		106	-0.3079737	0.06905371	-1.3151714	0.25565127	1	5203	tags=31%, list=28%, signal=43%	FALSE
REACTOME_MITOCHONDRIAL_TRNA_AMINOACYLATION	REACTOME_MITOCHONDRIAL_TRNA_AMINOACYLATION		21	-0.42813402	0.12596007	-1.3137114	0.25703165	1	7164	tags=52%, list=38%, signal=84%	FALSE
PID_EPO_PATHWAY	PID_EPO_PATHWAY		34	-0.38045672	0.12326656	-1.308299	0.26485407	1	3423	tags=29%, list=18%, signal=36%	FALSE
PID_CXCR4_PATHWAY	PID_CXCR4_PATHWAY		99	-0.30866054	0.07105943	-1.3078206	0.26468998	1	2859	tags=26%, list=15%, signal=31%	FALSE
KEGG_HEMATOPOIETIC_CELL_LINEAGE	KEGG_HEMATOPOIETIC_CELL_LINEAGE		80	-0.31822926	0.086842105	-1.3068007	0.26535589	1	4574	tags=33%, list=24%, signal=43%	FALSE
REACTOME_DOWNREGULATION_OF_SMAD2_3_SMAD4_TRANSCRIPTIONAL_ACTIVITY	REACTOME_DOWNREGULATION_OF_SMAD2_3_SMAD4_TRANSCRIPTIONAL_ACTIVITY		19	-0.44596064	0.12698413	-1.3050313	0.26720774	1	4853	tags=47%, list=26%, signal=64%	FALSE
KEGG_LYSINE_DEGRADATION	KEGG_LYSINE_DEGRADATION		43	-0.3671009	0.1270073	-1.2992774	0.27548316	1	5211	tags=44%, list=28%, signal=61%	FALSE
REACTOME_DOWNSTREAM_TCR_SIGNALING	REACTOME_DOWNSTREAM_TCR_SIGNALING		30	-0.38312915	0.12099125	-1.2929927	0.28499815	1	7475	tags=60%, list=40%, signal=99%	FALSE
BIOCARTA_TNFR1_PATHWAY	BIOCARTA_TNFR1_PATHWAY		29	-0.3927674	0.12462908	-1.2926724	0.28446987	1	4205	tags=45%, list=22%, signal=58%	FALSE
KEGG_CYSTEINE_AND_METHIONINE_METABOLISM	KEGG_CYSTEINE_AND_METHIONINE_METABOLISM		31	-0.37549874	0.14179105	-1.2881504	0.29075524	1	5339	tags=42%, list=28%, signal=58%	FALSE
PID_DNA_PK_PATHWAY	PID_DNA_PK_PATHWAY		15	-0.46379825	0.15590551	-1.2878157	0.29027605	1	3393	tags=33%, list=18%, signal=41%	FALSE
PID_LKB1_PATHWAY	PID_LKB1_PATHWAY		45	-0.35450044	0.11500701	-1.2840633	0.29527795	1	2688	tags=24%, list=14%, signal=28%	FALSE
REACTOME_MEIOTIC_RECOMBINATION	REACTOME_MEIOTIC_RECOMBINATION		58	-0.33475193	0.14027777	-1.2800211	0.30139795	1	4647	tags=43%, list=25%, signal=57%	FALSE
BIOCARTA_MAPK_PATHWAY	BIOCARTA_MAPK_PATHWAY		86	-0.31162733	0.114583336	-1.2799242	0.3004894	1	4753	tags=37%, list=25%, signal=49%	FALSE
REACTOME_RNA_POL_I_TRANSCRIPTION	REACTOME_RNA_POL_I_TRANSCRIPTION		58	-0.3296082	0.11126006	-1.277439	0.30349627	1	6207	tags=50%, list=33%, signal=74%	FALSE
REACTOME_TRANSCRIPTIONAL_REGULATION_OF_WHITE_ADIPOCYTE_DIFFERENTIATION	REACTOME_TRANSCRIPTIONAL_REGULATION_OF_WHITE_ADIPOCYTE_DIFFERENTIATION		72	-0.3159578	0.12242091	-1.2746766	0.30717364	1	3970	tags=35%, list=21%, signal=44%	FALSE
KEGG_PROGESTERONE_MEDIATED_OOCYTE_MATURATION	KEGG_PROGESTERONE_MEDIATED_OOCYTE_MATURATION		81	-0.31198764	0.101876676	-1.2731614	0.30879676	1	5421	tags=35%, list=29%, signal=48%	FALSE
KEGG_NATURAL_KILLER_CELL_MEDIATED_CYTOTOXICITY	KEGG_NATURAL_KILLER_CELL_MEDIATED_CYTOTOXICITY		121	-0.2913788	0.09308176	-1.269773	0.3138267	1	5613	tags=38%, list=30%, signal=54%	FALSE
REACTOME_PURINE_METABOLISM	REACTOME_PURINE_METABOLISM		31	-0.37269837	0.14630468	-1.2670794	0.31766987	1	3867	tags=35%, list=20%, signal=45%	FALSE
REACTOME_CIRCADIAN_REPRESSION_OF_EXPRESSION_BY_REV_ERBA	REACTOME_CIRCADIAN_REPRESSION_OF_EXPRESSION_BY_REV_ERBA		23	-0.40083018	0.16589147	-1.2639375	0.32216224	1	3303	tags=43%, list=17%, signal=53%	FALSE
REACTOME_CYTOSOLIC_TRNA_AMINOACYLATION	REACTOME_CYTOSOLIC_TRNA_AMINOACYLATION		24	-0.39111495	0.15430267	-1.262807	0.32289046	1	2757	tags=29%, list=15%, signal=34%	FALSE
REACTOME_IL_2_SIGNALING	REACTOME_IL_2_SIGNALING		41	-0.3550521	0.15044248	-1.2627976	0.32175356	1	2765	tags=22%, list=15%, signal=26%	FALSE
ST_T_CELL_SIGNAL_TRANSDUCTION	ST_T_CELL_SIGNAL_TRANSDUCTION		45	-0.34420836	0.15064563	-1.2576776	0.33016905	1	2195	tags=29%, list=12%, signal=33%	FALSE
KEGG_FC_EPSILON_RI_SIGNALING_PATHWAY	KEGG_FC_EPSILON_RI_SIGNALING_PATHWAY		75	-0.31003156	0.12938005	-1.2574372	0.3294827	1	4826	tags=35%, list=26%, signal=46%	FALSE
KEGG_PENTOSE_PHOSPHATE_PATHWAY	KEGG_PENTOSE_PHOSPHATE_PATHWAY		26	-0.3860957	0.15499255	-1.2562749	0.33056328	1	5570	tags=38%, list=29%, signal=54%	FALSE
BIOCARTA_P38MAPK_PATHWAY	BIOCARTA_P38MAPK_PATHWAY		37	-0.35351762	0.15532544	-1.2542847	0.33307526	1	4104	tags=38%, list=22%, signal=48%	FALSE
REACTOME_PI3K_CASCADE	REACTOME_PI3K_CASCADE		66	-0.3177066	0.108815424	-1.2533112	0.33365044	1	2672	tags=18%, list=14%, signal=21%	FALSE
KEGG_NON_SMALL_CELL_LUNG_CANCER	KEGG_NON_SMALL_CELL_LUNG_CANCER		54	-0.32865202	0.13788301	-1.2526649	0.3336663	1	5547	tags=43%, list=29%, signal=60%	FALSE
PID_IL8_CXCR1_PATHWAY	PID_IL8_CXCR1_PATHWAY		28	-0.37955672	0.14454277	-1.2518873	0.33409396	1	2859	tags=32%, list=15%, signal=38%	FALSE
REACTOME_SIGNALING_BY_THE_B_CELL_RECEPTOR_BCR	REACTOME_SIGNALING_BY_THE_B_CELL_RECEPTOR_BCR		117	-0.2898247	0.097964376	-1.2488695	0.33878487	1	5483	tags=32%, list=29%, signal=44%	FALSE
BIOCARTA_IGF1R_PATHWAY	BIOCARTA_IGF1R_PATHWAY		23	-0.39441037	0.18238021	-1.2477555	0.3397897	1	4702	tags=30%, list=25%, signal=40%	FALSE
PID_HDAC_CLASSIII_PATHWAY	PID_HDAC_CLASSIII_PATHWAY		24	-0.38444194	0.17612809	-1.2447687	0.3441833	1	4562	tags=38%, list=24%, signal=49%	FALSE
REACTOME_INNATE_IMMUNE_SYSTEM	REACTOME_INNATE_IMMUNE_SYSTEM		229	-0.26141968	0.069555305	-1.2396357	0.3527644	1	6000	tags=41%, list=32%, signal=59%	FALSE
BIOCARTA_RAC1_PATHWAY	BIOCARTA_RAC1_PATHWAY		23	-0.3923249	0.17584097	-1.2390478	0.3527333	1	2137	tags=26%, list=11%, signal=29%	FALSE
KEGG_AMYOTROPHIC_LATERAL_SCLEROSIS_ALS	KEGG_AMYOTROPHIC_LATERAL_SCLEROSIS_ALS		51	-0.3299311	0.14425771	-1.2339041	0.36163297	1	5785	tags=45%, list=31%, signal=65%	FALSE
ST_B_CELL_ANTIGEN_RECEPTOR	ST_B_CELL_ANTIGEN_RECEPTOR		40	-0.3466368	0.17226891	-1.233734	0.36070684	1	2195	tags=25%, list=12%, signal=28%	FALSE
BIOCARTA_GH_PATHWAY	BIOCARTA_GH_PATHWAY		28	-0.3772671	0.1653905	-1.2331406	0.36061233	1	5472	tags=39%, list=29%, signal=55%	FALSE
BIOCARTA_HIVNEF_PATHWAY	BIOCARTA_HIVNEF_PATHWAY		56	-0.31773627	0.14824797	-1.2315992	0.3624147	1	4205	tags=36%, list=22%, signal=46%	FALSE
BIOCARTA_RELA_PATHWAY	BIOCARTA_RELA_PATHWAY		15	-0.43409896	0.19554849	-1.2315178	0.36138728	1	3810	tags=47%, list=20%, signal=58%	FALSE
REACTOME_APC_C_CDC20_MEDIATED_DEGRADATION_OF_MITOTIC_PROTEINS	REACTOME_APC_C_CDC20_MEDIATED_DEGRADATION_OF_MITOTIC_PROTEINS		60	-0.3155419	0.1536232	-1.2235597	0.37597725	1	3871	tags=25%, list=20%, signal=31%	FALSE
REACTOME_REGULATION_OF_MITOTIC_CELL_CYCLE	REACTOME_REGULATION_OF_MITOTIC_CELL_CYCLE		72	-0.30668163	0.14870396	-1.2223734	0.3772919	1	5698	tags=35%, list=30%, signal=50%	FALSE
PID_HIF2PATHWAY	PID_HIF2PATHWAY		32	-0.35690847	0.1908284	-1.2194489	0.38203153	1	2456	tags=28%, list=13%, signal=32%	FALSE
PID_CONE_PATHWAY	PID_CONE_PATHWAY		21	-0.39416462	0.19273302	-1.2127143	0.39469546	1	2606	tags=24%, list=14%, signal=28%	FALSE
REACTOME_GLUCAGON_SIGNALING_IN_METABOLIC_REGULATION	REACTOME_GLUCAGON_SIGNALING_IN_METABOLIC_REGULATION		32	-0.35489377	0.21153846	-1.2109663	0.39692357	1	5510	tags=38%, list=29%, signal=53%	FALSE
REACTOME_PRE_NOTCH_EXPRESSION_AND_PROCESSING	REACTOME_PRE_NOTCH_EXPRESSION_AND_PROCESSING		42	-0.32745335	0.18053597	-1.2056326	0.40688083	1	5874	tags=48%, list=31%, signal=69%	FALSE
PID_SMAD2_3PATHWAY	PID_SMAD2_3PATHWAY		17	-0.40999871	0.22966507	-1.2039291	0.40911505	1	6269	tags=53%, list=33%, signal=79%	FALSE
PID_GMCSF_PATHWAY	PID_GMCSF_PATHWAY		37	-0.347771	0.20857988	-1.2018658	0.4121668	1	4591	tags=35%, list=24%, signal=46%	FALSE
PID_PS1_PATHWAY	PID_PS1_PATHWAY		46	-0.32231706	0.19498608	-1.2017748	0.41104034	1	3393	tags=33%, list=18%, signal=40%	FALSE
BIOCARTA_G1_PATHWAY	BIOCARTA_G1_PATHWAY		27	-0.36715	0.21156773	-1.2013835	0.4104973	1	5698	tags=44%, list=30%, signal=64%	FALSE
REACTOME_SIGNALING_BY_ILS	REACTOME_SIGNALING_BY_ILS		104	-0.2793139	0.16645649	-1.1970339	0.41851258	1	3837	tags=26%, list=20%, signal=32%	FALSE
PID_HDAC_CLASSII_PATHWAY	PID_HDAC_CLASSII_PATHWAY		33	-0.35044086	0.24304539	-1.1967341	0.41776326	1	6135	tags=52%, list=32%, signal=76%	FALSE
PID_FOXM1_PATHWAY	PID_FOXM1_PATHWAY		38	-0.33809295	0.20199147	-1.1965082	0.41694796	1	5159	tags=39%, list=27%, signal=54%	FALSE
REACTOME_G_BETA_GAMMA_SIGNALLING_THROUGH_PI3KGAMMA	REACTOME_G_BETA_GAMMA_SIGNALLING_THROUGH_PI3KGAMMA		24	-0.38106868	0.22561863	-1.1961771	0.41636783	1	1627	tags=21%, list=9%, signal=23%	FALSE
REACTOME_G_PROTEIN_BETA_GAMMA_SIGNALLING	REACTOME_G_PROTEIN_BETA_GAMMA_SIGNALLING		27	-0.3673187	0.24054463	-1.195476	0.41655535	1	2857	tags=26%, list=15%, signal=31%	FALSE
REACTOME_PRE_NOTCH_TRANSCRIPTION_AND_TRANSLATION	REACTOME_PRE_NOTCH_TRANSCRIPTION_AND_TRANSLATION		27	-0.36031976	0.22122571	-1.1948687	0.41652665	1	5874	tags=56%, list=31%, signal=81%	FALSE
KEGG_COLORECTAL_CANCER	KEGG_COLORECTAL_CANCER		62	-0.30224526	0.18741633	-1.1943308	0.41645864	1	5142	tags=34%, list=27%, signal=46%	FALSE
REACTOME_TRIF_MEDIATED_TLR3_SIGNALING	REACTOME_TRIF_MEDIATED_TLR3_SIGNALING		70	-0.29690686	0.164	-1.1927525	0.41866776	1	4603	tags=31%, list=24%, signal=41%	FALSE
PID_MYC_REPRESS_PATHWAY	PID_MYC_REPRESS_PATHWAY		60	-0.30956858	0.18424754	-1.1912442	0.4204033	1	3263	tags=32%, list=17%, signal=38%	FALSE
BIOCARTA_PGC1A_PATHWAY	BIOCARTA_PGC1A_PATHWAY		22	-0.38240725	0.22421524	-1.1877888	0.42639142	1	5905	tags=36%, list=31%, signal=53%	FALSE
BIOCARTA_ETS_PATHWAY	BIOCARTA_ETS_PATHWAY		18	-0.39883816	0.25791138	-1.1795689	0.4431892	1	3027	tags=44%, list=16%, signal=53%	FALSE
KEGG_TYPE_II_DIABETES_MELLITUS	KEGG_TYPE_II_DIABETES_MELLITUS		46	-0.31878823	0.21022727	-1.1751395	0.45161504	1	5472	tags=33%, list=29%, signal=46%	FALSE
BIOCARTA_CREB_PATHWAY	BIOCARTA_CREB_PATHWAY		27	-0.35838994	0.23845008	-1.1727557	0.45568305	1	4702	tags=30%, list=25%, signal=39%	FALSE
BIOCARTA_NKT_PATHWAY	BIOCARTA_NKT_PATHWAY		28	-0.35226223	0.24477611	-1.1727258	0.45433417	1	7858	tags=64%, list=42%, signal=110%	FALSE
PID_MTOR_4PATHWAY	PID_MTOR_4PATHWAY		66	-0.2962807	0.22161423	-1.1706179	0.45745996	1	3810	tags=24%, list=20%, signal=30%	FALSE
KEGG_ASTHMA	KEGG_ASTHMA		26	-0.3616649	0.24585219	-1.1692369	0.45921224	1	4826	tags=38%, list=26%, signal=52%	FALSE
REACTOME_SIGNALING_BY_NOTCH	REACTOME_SIGNALING_BY_NOTCH		100	-0.28160664	0.2232258	-1.1681558	0.46031475	1	3507	tags=28%, list=19%, signal=34%	FALSE
PID_HIV_NEF_PATHWAY	PID_HIV_NEF_PATHWAY		35	-0.33593506	0.23959827	-1.1676913	0.4599275	1	5928	tags=51%, list=31%, signal=75%	FALSE
ST_P38_MAPK_PATHWAY	ST_P38_MAPK_PATHWAY		36	-0.32965767	0.24017467	-1.1675899	0.45877516	1	5370	tags=39%, list=28%, signal=54%	FALSE
BIOCARTA_CELLCYCLE_PATHWAY	BIOCARTA_CELLCYCLE_PATHWAY		23	-0.37330848	0.25714287	-1.1663564	0.460155	1	5698	tags=43%, list=30%, signal=62%	FALSE
REACTOME_PYRIMIDINE_METABOLISM	REACTOME_PYRIMIDINE_METABOLISM		24	-0.36329907	0.25075528	-1.1651275	0.46156383	1	4102	tags=38%, list=22%, signal=48%	FALSE
REACTOME_ACTIVATION_OF_BH3_ONLY_PROTEINS	REACTOME_ACTIVATION_OF_BH3_ONLY_PROTEINS		16	-0.41158634	0.27126807	-1.1626445	0.46571687	1	2071	tags=31%, list=11%, signal=35%	FALSE
REACTOME_SIGNALING_BY_TGF_BETA_RECEPTOR_COMPLEX	REACTOME_SIGNALING_BY_TGF_BETA_RECEPTOR_COMPLEX		60	-0.2993666	0.22359397	-1.160954	0.46825236	1	3413	tags=30%, list=18%, signal=36%	FALSE
REACTOME_JNK_C_JUN_KINASES_PHOSPHORYLATION_AND_ACTIVATION_MEDIATED_BY_ACTIVATED_HUMAN_TAK1	REACTOME_JNK_C_JUN_KINASES_PHOSPHORYLATION_AND_ACTIVATION_MEDIATED_BY_ACTIVATED_HUMAN_TAK1		15	-0.40982053	0.27035332	-1.160803	0.4672276	1	7318	tags=60%, list=39%, signal=98%	FALSE
PID_NFKAPPAB_ATYPICAL_PATHWAY	PID_NFKAPPAB_ATYPICAL_PATHWAY		17	-0.40472886	0.26340693	-1.1596471	0.46844915	1	4591	tags=41%, list=24%, signal=54%	FALSE
BIOCARTA_TGFB_PATHWAY	BIOCARTA_TGFB_PATHWAY		19	-0.3840921	0.25267994	-1.1581987	0.47029185	1	2969	tags=32%, list=16%, signal=37%	FALSE
KEGG_REGULATION_OF_AUTOPHAGY	KEGG_REGULATION_OF_AUTOPHAGY		29	-0.35144833	0.2590909	-1.1545972	0.4773361	1	6555	tags=48%, list=35%, signal=74%	FALSE
ST_ERK1_ERK2_MAPK_PATHWAY	ST_ERK1_ERK2_MAPK_PATHWAY		32	-0.33531538	0.26462397	-1.1528509	0.4799213	1	957	tags=16%, list=5%, signal=16%	FALSE
REACTOME_ADAPTIVE_IMMUNE_SYSTEM	REACTOME_ADAPTIVE_IMMUNE_SYSTEM		488	-0.23070903	0.1178344	-1.1525306	0.4792759	1	6882	tags=41%, list=36%, signal=63%	FALSE
REACTOME_MYOGENESIS	REACTOME_MYOGENESIS		27	-0.3539769	0.28230336	-1.1490275	0.4857625	1	5073	tags=41%, list=27%, signal=56%	FALSE
REACTOME_RORA_ACTIVATES_CIRCADIAN_EXPRESSION	REACTOME_RORA_ACTIVATES_CIRCADIAN_EXPRESSION		24	-0.36318448	0.27943486	-1.1473303	0.48819968	1	3303	tags=38%, list=17%, signal=45%	FALSE
REACTOME_METABOLISM_OF_PROTEINS	REACTOME_METABOLISM_OF_PROTEINS		352	-0.23679043	0.15	-1.1440943	0.4940536	1	3999	tags=25%, list=21%, signal=31%	FALSE
KEGG_CHEMOKINE_SIGNALING_PATHWAY	KEGG_CHEMOKINE_SIGNALING_PATHWAY		176	-0.24939147	0.18585132	-1.1415501	0.49870348	1	3019	tags=22%, list=16%, signal=25%	FALSE
BIOCARTA_TID_PATHWAY	BIOCARTA_TID_PATHWAY		18	-0.3833196	0.29438543	-1.138145	0.5052931	1	4556	tags=44%, list=24%, signal=59%	FALSE
KEGG_TOLL_LIKE_RECEPTOR_SIGNALING_PATHWAY	KEGG_TOLL_LIKE_RECEPTOR_SIGNALING_PATHWAY		94	-0.27585933	0.25064936	-1.1359447	0.50904036	1	5975	tags=41%, list=32%, signal=60%	FALSE
BIOCARTA_FMLP_PATHWAY	BIOCARTA_FMLP_PATHWAY		35	-0.32493922	0.27482015	-1.1312999	0.51855356	1	1632	tags=17%, list=9%, signal=19%	FALSE
REACTOME_BETA_DEFENSINS	REACTOME_BETA_DEFENSINS		15	-0.4013657	0.2965204	-1.1311172	0.5175235	1	7481	tags=60%, list=40%, signal=99%	FALSE
REACTOME_GPVI_MEDIATED_ACTIVATION_CASCADE	REACTOME_GPVI_MEDIATED_ACTIVATION_CASCADE		31	-0.3347369	0.28096676	-1.1308389	0.51671463	1	6756	tags=58%, list=36%, signal=90%	FALSE
KEGG_APOPTOSIS	KEGG_APOPTOSIS		86	-0.2710975	0.25562915	-1.13012	0.51690674	1	5941	tags=43%, list=31%, signal=62%	FALSE
REACTOME_PROTEIN_FOLDING	REACTOME_PROTEIN_FOLDING		48	-0.3032063	0.26988637	-1.1292254	0.51749057	1	4876	tags=31%, list=26%, signal=42%	FALSE
PID_P38_MK2_PATHWAY	PID_P38_MK2_PATHWAY		19	-0.37158355	0.28244275	-1.1282517	0.51834816	1	2444	tags=26%, list=13%, signal=30%	FALSE
KEGG_RIG_I_LIKE_RECEPTOR_SIGNALING_PATHWAY	KEGG_RIG_I_LIKE_RECEPTOR_SIGNALING_PATHWAY		65	-0.28626472	0.25989446	-1.1254933	0.5234624	1	5975	tags=42%, list=32%, signal=61%	FALSE
HALLMARK_UNFOLDED_PROTEIN_RESPONSE	HALLMARK_UNFOLDED_PROTEIN_RESPONSE		109	-0.26196435	0.26623377	-1.1247431	0.5236644	1	5746	tags=41%, list=30%, signal=59%	FALSE
REACTOME_INTRINSIC_PATHWAY_FOR_APOPTOSIS	REACTOME_INTRINSIC_PATHWAY_FOR_APOPTOSIS		28	-0.3368648	0.29239765	-1.1246403	0.52242297	1	6719	tags=61%, list=36%, signal=94%	FALSE
REACTOME_POST_NMDA_RECEPTOR_ACTIVATION_EVENTS	REACTOME_POST_NMDA_RECEPTOR_ACTIVATION_EVENTS		32	-0.3295694	0.3045977	-1.124216	0.52192426	1	4957	tags=34%, list=26%, signal=47%	FALSE
KEGG_FC_GAMMA_R_MEDIATED_PHAGOCYTOSIS	KEGG_FC_GAMMA_R_MEDIATED_PHAGOCYTOSIS		87	-0.2716431	0.25419354	-1.1237553	0.52153045	1	4591	tags=32%, list=24%, signal=42%	FALSE
REACTOME_TRAF6_MEDIATED_NFKB_ACTIVATION	REACTOME_TRAF6_MEDIATED_NFKB_ACTIVATION		19	-0.36815244	0.30838323	-1.1220944	0.52385575	1	5943	tags=53%, list=31%, signal=77%	FALSE
BIOCARTA_P53HYPOXIA_PATHWAY	BIOCARTA_P53HYPOXIA_PATHWAY		22	-0.36629814	0.30267063	-1.1199201	0.5274616	1	2456	tags=32%, list=13%, signal=37%	FALSE
REACTOME_SYNTHESIS_OF_PIPS_AT_THE_GOLGI_MEMBRANE	REACTOME_SYNTHESIS_OF_PIPS_AT_THE_GOLGI_MEMBRANE		16	-0.3951625	0.33898306	-1.1168259	0.5331907	1	1145	tags=19%, list=6%, signal=20%	FALSE
PID_CIRCADIAN_PATHWAY	PID_CIRCADIAN_PATHWAY		16	-0.3966023	0.3129139	-1.1164666	0.53255713	1	1364	tags=25%, list=7%, signal=27%	FALSE
KEGG_LONG_TERM_POTENTIATION	KEGG_LONG_TERM_POTENTIATION		67	-0.27994075	0.29598895	-1.1158093	0.5326763	1	2797	tags=19%, list=15%, signal=23%	FALSE
REACTOME_PI_METABOLISM	REACTOME_PI_METABOLISM		46	-0.2998299	0.3034483	-1.1085634	0.54846376	1	2257	tags=20%, list=12%, signal=22%	FALSE
REACTOME_DEFENSINS	REACTOME_DEFENSINS		20	-0.36647025	0.316847	-1.1080496	0.54816264	1	7481	tags=60%, list=40%, signal=99%	FALSE
REACTOME_ACTIVATION_OF_NMDA_RECEPTOR_UPON_GLUTAMATE_BINDING_AND_POSTSYNAPTIC_EVENTS	REACTOME_ACTIVATION_OF_NMDA_RECEPTOR_UPON_GLUTAMATE_BINDING_AND_POSTSYNAPTIC_EVENTS		36	-0.317905	0.32225433	-1.1072572	0.54855806	1	4957	tags=33%, list=26%, signal=45%	FALSE
KEGG_T_CELL_RECEPTOR_SIGNALING_PATHWAY	KEGG_T_CELL_RECEPTOR_SIGNALING_PATHWAY		107	-0.25915584	0.30379745	-1.1052474	0.55169874	1	5409	tags=36%, list=29%, signal=49%	FALSE
ST_INTERLEUKIN_4_PATHWAY	ST_INTERLEUKIN_4_PATHWAY		26	-0.34032178	0.32973805	-1.1050713	0.55059385	1	2137	tags=19%, list=11%, signal=22%	FALSE
KEGG_INSULIN_SIGNALING_PATHWAY	KEGG_INSULIN_SIGNALING_PATHWAY		136	-0.24814264	0.2763819	-1.1016822	0.5572064	1	5472	tags=29%, list=29%, signal=41%	FALSE
KEGG_GLYCINE_SERINE_AND_THREONINE_METABOLISM	KEGG_GLYCINE_SERINE_AND_THREONINE_METABOLISM		31	-0.3268264	0.31009957	-1.101456	0.5562735	1	6621	tags=52%, list=35%, signal=79%	FALSE
PID_NFKAPPAB_CANONICAL_PATHWAY	PID_NFKAPPAB_CANONICAL_PATHWAY		21	-0.35943434	0.32735425	-1.0985566	0.5618911	1	3810	tags=38%, list=20%, signal=48%	FALSE
REACTOME_CREB_PHOSPHORYLATION_THROUGH_THE_ACTIVATION_OF_RAS	REACTOME_CREB_PHOSPHORYLATION_THROUGH_THE_ACTIVATION_OF_RAS		26	-0.33988956	0.33781764	-1.0971922	0.56365407	1	3997	tags=31%, list=21%, signal=39%	FALSE
BIOCARTA_FAS_PATHWAY	BIOCARTA_FAS_PATHWAY		30	-0.32277882	0.35874438	-1.0953716	0.5665063	1	2990	tags=30%, list=16%, signal=36%	FALSE
REACTOME_RNA_POL_I_PROMOTER_OPENING	REACTOME_RNA_POL_I_PROMOTER_OPENING		36	-0.30915084	0.3216885	-1.0953592	0.56499803	1	4647	tags=42%, list=25%, signal=55%	FALSE
BIOCARTA_CALCINEURIN_PATHWAY	BIOCARTA_CALCINEURIN_PATHWAY		17	-0.37990832	0.36833856	-1.0953032	0.56361663	1	1632	tags=24%, list=9%, signal=26%	FALSE
REACTOME_SIGNALING_BY_NOTCH1	REACTOME_SIGNALING_BY_NOTCH1		68	-0.2781037	0.29324323	-1.0951992	0.5623437	1	6061	tags=46%, list=32%, signal=67%	FALSE
PID_P73PATHWAY	PID_P73PATHWAY		78	-0.2701282	0.3224543	-1.094557	0.56246495	1	5292	tags=36%, list=28%, signal=50%	FALSE
BIOCARTA_NOS1_PATHWAY	BIOCARTA_NOS1_PATHWAY		21	-0.35277778	0.33736762	-1.0943092	0.56155354	1	5905	tags=43%, list=31%, signal=62%	FALSE
PID_TOLL_ENDOGENOUS_PATHWAY	PID_TOLL_ENDOGENOUS_PATHWAY		21	-0.35351953	0.35276073	-1.0936936	0.5615944	1	4603	tags=33%, list=24%, signal=44%	FALSE
REACTOME_PLC_BETA_MEDIATED_EVENTS	REACTOME_PLC_BETA_MEDIATED_EVENTS		42	-0.30488572	0.3231884	-1.0920656	0.56397176	1	5905	tags=36%, list=31%, signal=52%	FALSE
REACTOME_AUTODEGRADATION_OF_CDH1_BY_CDH1_APC_C	REACTOME_AUTODEGRADATION_OF_CDH1_BY_CDH1_APC_C		51	-0.290723	0.32409972	-1.0915747	0.5638224	1	3871	tags=24%, list=20%, signal=30%	FALSE
BIOCARTA_NFAT_PATHWAY	BIOCARTA_NFAT_PATHWAY		53	-0.28608388	0.32344633	-1.0909438	0.56379473	1	5905	tags=34%, list=31%, signal=49%	FALSE
PID_CD8_TCR_PATHWAY	PID_CD8_TCR_PATHWAY		52	-0.29161376	0.30278885	-1.0907936	0.56266826	1	7856	tags=58%, list=42%, signal=98%	FALSE
KEGG_NEUROTROPHIN_SIGNALING_PATHWAY	KEGG_NEUROTROPHIN_SIGNALING_PATHWAY		122	-0.25203776	0.28607276	-1.0907179	0.56137973	1	5409	tags=33%, list=29%, signal=46%	FALSE
REACTOME_SIGNALING_BY_EGFR_IN_CANCER	REACTOME_SIGNALING_BY_EGFR_IN_CANCER		105	-0.25190645	0.3026152	-1.0850701	0.57303685	1	5409	tags=30%, list=29%, signal=41%	FALSE
REACTOME_NFKB_AND_MAP_KINASES_ACTIVATION_MEDIATED_BY_TLR4_SIGNALING_REPERTOIRE	REACTOME_NFKB_AND_MAP_KINASES_ACTIVATION_MEDIATED_BY_TLR4_SIGNALING_REPERTOIRE		67	-0.2730985	0.3237705	-1.0812117	0.5808468	1	4603	tags=30%, list=24%, signal=39%	FALSE
REACTOME_SIGNALING_BY_RHO_GTPASES	REACTOME_SIGNALING_BY_RHO_GTPASES		105	-0.25027788	0.3268734	-1.0751805	0.59398794	1	2052	tags=18%, list=11%, signal=20%	FALSE
PID_TNF_PATHWAY	PID_TNF_PATHWAY		44	-0.2910716	0.33094555	-1.0721556	0.5999693	1	1359	tags=18%, list=7%, signal=20%	FALSE
REACTOME_INFLAMMASOMES	REACTOME_INFLAMMASOMES		16	-0.37037432	0.371875	-1.0681231	0.6083576	1	2212	tags=31%, list=12%, signal=35%	FALSE
REACTOME_SIGNALING_BY_FGFR1_MUTANTS	REACTOME_SIGNALING_BY_FGFR1_MUTANTS		28	-0.3212858	0.3690476	-1.0657973	0.61263657	1	6056	tags=39%, list=32%, signal=58%	FALSE
KEGG_RENIN_ANGIOTENSIN_SYSTEM	KEGG_RENIN_ANGIOTENSIN_SYSTEM		17	-0.36482176	0.38403615	-1.0643202	0.614823	1	2980	tags=35%, list=16%, signal=42%	FALSE
KEGG_ONE_CARBON_POOL_BY_FOLATE	KEGG_ONE_CARBON_POOL_BY_FOLATE		15	-0.3694773	0.38267717	-1.0609828	0.62124836	1	6621	tags=53%, list=35%, signal=82%	FALSE
KEGG_CHRONIC_MYELOID_LEUKEMIA	KEGG_CHRONIC_MYELOID_LEUKEMIA		72	-0.2649052	0.35270807	-1.0608442	0.6200351	1	3337	tags=25%, list=18%, signal=30%	FALSE
PID_P53_DOWNSTREAM_PATHWAY	PID_P53_DOWNSTREAM_PATHWAY		133	-0.23978075	0.35507247	-1.0595671	0.6215976	1	2990	tags=23%, list=16%, signal=27%	FALSE
PID_AMB2_NEUTROPHILS_PATHWAY	PID_AMB2_NEUTROPHILS_PATHWAY		40	-0.29492518	0.3779972	-1.0593001	0.6207162	1	1589	tags=20%, list=8%, signal=22%	FALSE
ST_ADRENERGIC	ST_ADRENERGIC		34	-0.305885	0.37755102	-1.0592242	0.61928815	1	1913	tags=21%, list=10%, signal=23%	FALSE
REACTOME_NUCLEOTIDE_BINDING_DOMAIN_LEUCINE_RICH_REPEAT_CONTAINING_RECEPTOR_NLR_SIGNALING_PATHWAYS	REACTOME_NUCLEOTIDE_BINDING_DOMAIN_LEUCINE_RICH_REPEAT_CONTAINING_RECEPTOR_NLR_SIGNALING_PATHWAYS		43	-0.2928011	0.36585367	-1.0589991	0.61829615	1	5421	tags=44%, list=29%, signal=62%	FALSE
KEGG_SYSTEMIC_LUPUS_ERYTHEMATOSUS	KEGG_SYSTEMIC_LUPUS_ERYTHEMATOSUS		91	-0.2545843	0.36243385	-1.0577612	0.6197611	1	6207	tags=43%, list=33%, signal=64%	FALSE
REACTOME_INSULIN_RECEPTOR_SIGNALLING_CASCADE	REACTOME_INSULIN_RECEPTOR_SIGNALLING_CASCADE		82	-0.25766948	0.35400516	-1.0577307	0.6182719	1	2672	tags=16%, list=14%, signal=18%	FALSE
REACTOME_IL1_SIGNALING	REACTOME_IL1_SIGNALING		37	-0.30125073	0.38294798	-1.0565541	0.61967725	1	5370	tags=38%, list=28%, signal=53%	FALSE
PID_MAPK_TRK_PATHWAY	PID_MAPK_TRK_PATHWAY		33	-0.30788204	0.3762811	-1.0535457	0.6255697	1	4960	tags=36%, list=26%, signal=49%	FALSE
BIOCARTA_MEF2D_PATHWAY	BIOCARTA_MEF2D_PATHWAY		18	-0.36114967	0.40944883	-1.0532297	0.62480915	1	3847	tags=39%, list=20%, signal=49%	FALSE
BIOCARTA_PAR1_PATHWAY	BIOCARTA_PAR1_PATHWAY		35	-0.30138728	0.39244187	-1.0523094	0.6255083	1	2857	tags=26%, list=15%, signal=30%	FALSE
REACTOME_RAS_ACTIVATION_UOPN_CA2_INFUX_THROUGH_NMDA_RECEPTOR	REACTOME_RAS_ACTIVATION_UOPN_CA2_INFUX_THROUGH_NMDA_RECEPTOR		16	-0.36019683	0.38948995	-1.047338	0.6361856	1	3997	tags=31%, list=21%, signal=40%	FALSE
PID_LYMPH_ANGIOGENESIS_PATHWAY	PID_LYMPH_ANGIOGENESIS_PATHWAY		22	-0.33984435	0.3961136	-1.043543	0.6438723	1	336	tags=14%, list=2%, signal=14%	FALSE
PID_P38_ALPHA_BETA_PATHWAY	PID_P38_ALPHA_BETA_PATHWAY		31	-0.31197578	0.37904468	-1.0434096	0.6425727	1	1389	tags=19%, list=7%, signal=21%	FALSE
REACTOME_APC_C_CDH1_MEDIATED_DEGRADATION_OF_CDC20_AND_OTHER_APC_C_CDH1_TARGETED_PROTEINS_IN_LATE_MITOSIS_EARLY_G1	REACTOME_APC_C_CDH1_MEDIATED_DEGRADATION_OF_CDC20_AND_OTHER_APC_C_CDH1_TARGETED_PROTEINS_IN_LATE_MITOSIS_EARLY_G1		59	-0.26835763	0.38395417	-1.042666	0.64280844	1	3871	tags=22%, list=20%, signal=28%	FALSE
SA_G1_AND_S_PHASES	SA_G1_AND_S_PHASES		15	-0.36875415	0.39936608	-1.040867	0.6455623	1	1322	tags=20%, list=7%, signal=21%	FALSE
BIOCARTA_MITOCHONDRIA_PATHWAY	BIOCARTA_MITOCHONDRIA_PATHWAY		20	-0.33621225	0.4254317	-1.0393975	0.647563	1	5928	tags=55%, list=31%, signal=80%	FALSE
KEGG_SELENOAMINO_ACID_METABOLISM	KEGG_SELENOAMINO_ACID_METABOLISM		23	-0.32558948	0.4009009	-1.0380418	0.64933634	1	1214	tags=17%, list=6%, signal=19%	FALSE
REACTOME_REGULATION_OF_SIGNALING_BY_CBL	REACTOME_REGULATION_OF_SIGNALING_BY_CBL		18	-0.34350985	0.41167665	-1.0376492	0.6487515	1	6828	tags=61%, list=36%, signal=96%	FALSE
REACTOME_MEIOTIC_SYNAPSIS	REACTOME_MEIOTIC_SYNAPSIS		55	-0.27121812	0.40369394	-1.0310258	0.6634	1	4647	tags=40%, list=25%, signal=53%	FALSE
BIOCARTA_GLEEVEC_PATHWAY	BIOCARTA_GLEEVEC_PATHWAY		23	-0.32041815	0.4237037	-1.029602	0.6653063	1	2765	tags=26%, list=15%, signal=31%	FALSE
KEGG_JAK_STAT_SIGNALING_PATHWAY	KEGG_JAK_STAT_SIGNALING_PATHWAY		146	-0.23269689	0.40168878	-1.0292532	0.6645769	1	2875	tags=18%, list=15%, signal=22%	FALSE
REACTOME_PD1_SIGNALING	REACTOME_PD1_SIGNALING		15	-0.36569917	0.42439026	-1.0290805	0.6633963	1	7475	tags=67%, list=40%, signal=110%	FALSE
PID_CASPASE_PATHWAY	PID_CASPASE_PATHWAY		51	-0.27341133	0.4279539	-1.0285141	0.66331357	1	5928	tags=45%, list=31%, signal=66%	FALSE
BIOCARTA_AT1R_PATHWAY	BIOCARTA_AT1R_PATHWAY		31	-0.3056995	0.41826215	-1.0281433	0.6625576	1	2286	tags=23%, list=12%, signal=26%	FALSE
REACTOME_CYCLIN_E_ASSOCIATED_EVENTS_DURING_G1_S_TRANSITION_	REACTOME_CYCLIN_E_ASSOCIATED_EVENTS_DURING_G1_S_TRANSITION_		58	-0.26666525	0.41086352	-1.0242207	0.6708422	1	5698	tags=33%, list=30%, signal=47%	FALSE
KEGG_P53_SIGNALING_PATHWAY	KEGG_P53_SIGNALING_PATHWAY		67	-0.25883302	0.43427834	-1.0241858	0.66928107	1	2196	tags=19%, list=12%, signal=22%	FALSE
KEGG_CALCIUM_SIGNALING_PATHWAY	KEGG_CALCIUM_SIGNALING_PATHWAY		173	-0.22320856	0.4079254	-1.0226241	0.6715795	1	3971	tags=21%, list=21%, signal=26%	FALSE
REACTOME_NUCLEAR_EVENTS_KINASE_AND_TRANSCRIPTION_FACTOR_ACTIVATION	REACTOME_NUCLEAR_EVENTS_KINASE_AND_TRANSCRIPTION_FACTOR_ACTIVATION		24	-0.3184472	0.43712574	-1.0213145	0.673283	1	4587	tags=33%, list=24%, signal=44%	FALSE
REACTOME_SIGNALING_BY_FGFR_IN_DISEASE	REACTOME_SIGNALING_BY_FGFR_IN_DISEASE		121	-0.23311485	0.43965518	-1.0181928	0.6798954	1	5230	tags=28%, list=28%, signal=39%	FALSE
PID_IL2_1PATHWAY	PID_IL2_1PATHWAY		55	-0.267024	0.4352617	-1.0175706	0.679707	1	2857	tags=24%, list=15%, signal=28%	FALSE
REACTOME_CDT1_ASSOCIATION_WITH_THE_CDC6_ORC_ORIGIN_COMPLEX	REACTOME_CDT1_ASSOCIATION_WITH_THE_CDC6_ORC_ORIGIN_COMPLEX		51	-0.27297983	0.46534654	-1.0164672	0.68078804	1	2837	tags=18%, list=15%, signal=21%	FALSE
REACTOME_AMINO_ACID_SYNTHESIS_AND_INTERCONVERSION_TRANSAMINATION	REACTOME_AMINO_ACID_SYNTHESIS_AND_INTERCONVERSION_TRANSAMINATION		16	-0.36053297	0.44323483	-1.015361	0.681862	1	5516	tags=44%, list=29%, signal=62%	FALSE
PID_CD8_TCR_DOWNSTREAM_PATHWAY	PID_CD8_TCR_DOWNSTREAM_PATHWAY		61	-0.25695965	0.45192307	-1.0151674	0.6806866	1	3850	tags=26%, list=20%, signal=33%	FALSE
BIOCARTA_DC_PATHWAY	BIOCARTA_DC_PATHWAY		22	-0.3248047	0.45327103	-1.0128523	0.68490994	1	7792	tags=55%, list=41%, signal=93%	FALSE
PID_TCR_CALCIUM_PATHWAY	PID_TCR_CALCIUM_PATHWAY		29	-0.30535403	0.44065282	-1.0118607	0.6857349	1	2391	tags=24%, list=13%, signal=28%	FALSE
KEGG_PHOSPHATIDYLINOSITOL_SIGNALING_SYSTEM	KEGG_PHOSPHATIDYLINOSITOL_SIGNALING_SYSTEM		76	-0.24758393	0.4302949	-1.0110812	0.685976	1	3036	tags=22%, list=16%, signal=27%	FALSE
REACTOME_P75_NTR_RECEPTOR_MEDIATED_SIGNALLING	REACTOME_P75_NTR_RECEPTOR_MEDIATED_SIGNALLING		78	-0.24731797	0.4555256	-1.0079663	0.69200844	1	4930	tags=32%, list=26%, signal=43%	FALSE
BIOCARTA_IL2_PATHWAY	BIOCARTA_IL2_PATHWAY		22	-0.32192278	0.4592934	-1.0076011	0.6912929	1	3393	tags=27%, list=18%, signal=33%	FALSE
BIOCARTA_IL22BP_PATHWAY	BIOCARTA_IL22BP_PATHWAY		16	-0.3474753	0.4600639	-1.0068592	0.69143295	1	2765	tags=25%, list=15%, signal=29%	FALSE
REACTOME_GAB1_SIGNALOSOME	REACTOME_GAB1_SIGNALOSOME		36	-0.28695363	0.43235704	-1.003369	0.6984005	1	5035	tags=28%, list=27%, signal=38%	FALSE
BIOCARTA_NTHI_PATHWAY	BIOCARTA_NTHI_PATHWAY		24	-0.3110512	0.49109793	-1.0023028	0.6993912	1	3810	tags=29%, list=20%, signal=36%	FALSE
PID_INSULIN_PATHWAY	PID_INSULIN_PATHWAY		45	-0.27157214	0.4465318	-1.0014946	0.6997901	1	3423	tags=27%, list=18%, signal=32%	FALSE
KEGG_PROSTATE_CANCER	KEGG_PROSTATE_CANCER		87	-0.24076173	0.45525292	-0.9974961	0.7078845	1	4753	tags=29%, list=25%, signal=38%	FALSE
KEGG_RIBOFLAVIN_METABOLISM	KEGG_RIBOFLAVIN_METABOLISM		16	-0.34077504	0.46329528	-0.9968815	0.7077895	1	3996	tags=38%, list=21%, signal=48%	FALSE
BIOCARTA_MAL_PATHWAY	BIOCARTA_MAL_PATHWAY		19	-0.33211535	0.47230768	-0.99654204	0.70691663	1	6181	tags=42%, list=33%, signal=63%	FALSE
REACTOME_TAK1_ACTIVATES_NFKB_BY_PHOSPHORYLATION_AND_ACTIVATION_OF_IKKS_COMPLEX	REACTOME_TAK1_ACTIVATES_NFKB_BY_PHOSPHORYLATION_AND_ACTIVATION_OF_IKKS_COMPLEX		21	-0.32111806	0.47928995	-0.9929821	0.71415913	1	6000	tags=48%, list=32%, signal=70%	FALSE
REACTOME_NOD1_2_SIGNALING_PATHWAY	REACTOME_NOD1_2_SIGNALING_PATHWAY		28	-0.30057847	0.498452	-0.9916236	0.7158395	1	6000	tags=50%, list=32%, signal=73%	FALSE
PID_EPHA_FWDPATHWAY	PID_EPHA_FWDPATHWAY		34	-0.28551212	0.49853373	-0.98998266	0.718319	1	1389	tags=18%, list=7%, signal=19%	FALSE
PID_IL4_2PATHWAY	PID_IL4_2PATHWAY		63	-0.25117332	0.48233697	-0.9884391	0.7205929	1	4591	tags=24%, list=24%, signal=31%	FALSE
BIOCARTA_INSULIN_PATHWAY	BIOCARTA_INSULIN_PATHWAY		22	-0.3164762	0.46119404	-0.98742074	0.7213842	1	4960	tags=36%, list=26%, signal=49%	FALSE
KEGG_GLIOMA	KEGG_GLIOMA		65	-0.24795873	0.50334674	-0.98447925	0.7272036	1	1322	tags=12%, list=7%, signal=13%	FALSE
REACTOME_SIGNALING_BY_WNT	REACTOME_SIGNALING_BY_WNT		59	-0.2529172	0.49584487	-0.98388153	0.72702986	1	2816	tags=19%, list=15%, signal=22%	FALSE
REACTOME_SYNTHESIS_OF_PIPS_AT_THE_PLASMA_MEMBRANE	REACTOME_SYNTHESIS_OF_PIPS_AT_THE_PLASMA_MEMBRANE		30	-0.28868303	0.4760479	-0.981844	0.73033357	1	5959	tags=43%, list=32%, signal=63%	FALSE
HALLMARK_PI3K_AKT_MTOR_SIGNALING	HALLMARK_PI3K_AKT_MTOR_SIGNALING		104	-0.23295754	0.5116883	-0.98154557	0.72943556	1	3743	tags=21%, list=20%, signal=26%	FALSE
SA_B_CELL_RECEPTOR_COMPLEXES	SA_B_CELL_RECEPTOR_COMPLEXES		24	-0.30804282	0.48153618	-0.9785811	0.73517144	1	4329	tags=33%, list=23%, signal=43%	FALSE
REACTOME_SIGNALLING_BY_NGF	REACTOME_SIGNALLING_BY_NGF		211	-0.2103028	0.52878964	-0.97847337	0.73379797	1	5409	tags=29%, list=29%, signal=41%	FALSE
PID_TCPTP_PATHWAY	PID_TCPTP_PATHWAY		41	-0.26799178	0.48714286	-0.973578	0.744249	1	3027	tags=22%, list=16%, signal=26%	FALSE
PID_FOXO_PATHWAY	PID_FOXO_PATHWAY		47	-0.26241362	0.4979424	-0.9731389	0.7436163	1	5389	tags=36%, list=29%, signal=50%	FALSE
KEGG_HEDGEHOG_SIGNALING_PATHWAY	KEGG_HEDGEHOG_SIGNALING_PATHWAY		53	-0.2577365	0.4985591	-0.9730613	0.742165	1	5881	tags=38%, list=31%, signal=55%	FALSE
HALLMARK_MTORC1_SIGNALING	HALLMARK_MTORC1_SIGNALING		190	-0.21043815	0.5346651	-0.97092164	0.7456335	1	4117	tags=25%, list=22%, signal=32%	FALSE
REACTOME_HEMOSTASIS	REACTOME_HEMOSTASIS		426	-0.19482526	0.5743315	-0.9708262	0.74420714	1	2959	tags=18%, list=16%, signal=21%	FALSE
BIOCARTA_ATM_PATHWAY	BIOCARTA_ATM_PATHWAY		19	-0.32271555	0.4946401	-0.97077954	0.74265677	1	4150	tags=37%, list=22%, signal=47%	FALSE
REACTOME_EGFR_DOWNREGULATION	REACTOME_EGFR_DOWNREGULATION		24	-0.3044767	0.5	-0.9705568	0.74156195	1	1770	tags=17%, list=9%, signal=18%	FALSE
BIOCARTA_GPCR_PATHWAY	BIOCARTA_GPCR_PATHWAY		33	-0.28173694	0.4962742	-0.9694204	0.7426503	1	5905	tags=39%, list=31%, signal=57%	FALSE
BIOCARTA_TOLL_PATHWAY	BIOCARTA_TOLL_PATHWAY		36	-0.27721643	0.5123726	-0.9682742	0.7438532	1	3810	tags=28%, list=20%, signal=35%	FALSE
PID_THROMBIN_PAR1_PATHWAY	PID_THROMBIN_PAR1_PATHWAY		42	-0.26489812	0.52578795	-0.9669386	0.7455377	1	4996	tags=31%, list=26%, signal=42%	FALSE
REACTOME_CELL_DEATH_SIGNALLING_VIA_NRAGE_NRIF_AND_NADE	REACTOME_CELL_DEATH_SIGNALLING_VIA_NRAGE_NRIF_AND_NADE		57	-0.2523149	0.53185594	-0.9666569	0.744618	1	1853	tags=18%, list=10%, signal=19%	FALSE
BIOCARTA_RACCYCD_PATHWAY	BIOCARTA_RACCYCD_PATHWAY		25	-0.3006032	0.52444446	-0.9600176	0.7591899	1	5547	tags=44%, list=29%, signal=62%	FALSE
KEGG_NEUROACTIVE_LIGAND_RECEPTOR_INTERACTION	KEGG_NEUROACTIVE_LIGAND_RECEPTOR_INTERACTION		267	-0.20183478	0.5825688	-0.95585483	0.76803833	1	5289	tags=27%, list=28%, signal=36%	FALSE
REACTOME_PRE_NOTCH_PROCESSING_IN_GOLGI	REACTOME_PRE_NOTCH_PROCESSING_IN_GOLGI		16	-0.3345135	0.5159332	-0.9549745	0.76848227	1	3930	tags=25%, list=21%, signal=32%	FALSE
REACTOME_OPIOID_SIGNALLING	REACTOME_OPIOID_SIGNALLING		76	-0.23605634	0.5545335	-0.9544356	0.7681483	1	5557	tags=30%, list=29%, signal=43%	FALSE
BIOCARTA_GATA3_PATHWAY	BIOCARTA_GATA3_PATHWAY		16	-0.32646653	0.5194805	-0.9533372	0.76913273	1	2789	tags=19%, list=15%, signal=22%	FALSE
BIOCARTA_SPRY_PATHWAY	BIOCARTA_SPRY_PATHWAY		18	-0.32030883	0.51969695	-0.94708484	0.78239244	1	2099	tags=17%, list=11%, signal=19%	FALSE
BIOCARTA_STRESS_PATHWAY	BIOCARTA_STRESS_PATHWAY		24	-0.28998968	0.54615384	-0.9453652	0.7849042	1	4357	tags=33%, list=23%, signal=43%	FALSE
REACTOME_MAP_KINASE_ACTIVATION_IN_TLR_CASCADE	REACTOME_MAP_KINASE_ACTIVATION_IN_TLR_CASCADE		48	-0.25481826	0.5423729	-0.94529694	0.7833702	1	4603	tags=29%, list=24%, signal=38%	FALSE
PID_IL1_PATHWAY	PID_IL1_PATHWAY		31	-0.27990618	0.5432277	-0.9449069	0.7826174	1	5370	tags=39%, list=28%, signal=54%	FALSE
BIOCARTA_BARRESTIN_SRC_PATHWAY	BIOCARTA_BARRESTIN_SRC_PATHWAY		15	-0.33571896	0.5318471	-0.94144446	0.7895877	1	1274	tags=13%, list=7%, signal=14%	FALSE
KEGG_ALANINE_ASPARTATE_AND_GLUTAMATE_METABOLISM	KEGG_ALANINE_ASPARTATE_AND_GLUTAMATE_METABOLISM		32	-0.27363735	0.54761904	-0.9410791	0.7887806	1	5435	tags=38%, list=29%, signal=53%	FALSE
REACTOME_RAP1_SIGNALLING	REACTOME_RAP1_SIGNALLING		15	-0.32952115	0.5416667	-0.94080776	0.7877656	1	2566	tags=27%, list=14%, signal=31%	FALSE
KEGG_INTESTINAL_IMMUNE_NETWORK_FOR_IGA_PRODUCTION	KEGG_INTESTINAL_IMMUNE_NETWORK_FOR_IGA_PRODUCTION		43	-0.2576469	0.58129495	-0.9397425	0.7886436	1	8124	tags=58%, list=43%, signal=102%	FALSE
KEGG_WNT_SIGNALING_PATHWAY	KEGG_WNT_SIGNALING_PATHWAY		146	-0.21178064	0.60692215	-0.9395182	0.78748786	1	3503	tags=22%, list=19%, signal=27%	FALSE
KEGG_ANTIGEN_PROCESSING_AND_PRESENTATION	KEGG_ANTIGEN_PROCESSING_AND_PRESENTATION		70	-0.23668228	0.58077437	-0.9391173	0.7867481	1	6189	tags=40%, list=33%, signal=59%	FALSE
BIOCARTA_KERATINOCYTE_PATHWAY	BIOCARTA_KERATINOCYTE_PATHWAY		46	-0.25325873	0.5832168	-0.9382403	0.78713816	1	3810	tags=26%, list=20%, signal=33%	FALSE
PID_AR_NONGENOMIC_PATHWAY	PID_AR_NONGENOMIC_PATHWAY		31	-0.28209895	0.5557185	-0.93791467	0.7863053	1	6649	tags=48%, list=35%, signal=75%	FALSE
REACTOME_REGULATION_OF_WATER_BALANCE_BY_RENAL_AQUAPORINS	REACTOME_REGULATION_OF_WATER_BALANCE_BY_RENAL_AQUAPORINS		42	-0.258678	0.55604076	-0.937599	0.7853896	1	6908	tags=40%, list=37%, signal=64%	FALSE
REACTOME_PHOSPHOLIPASE_C_MEDIATED_CASCADE	REACTOME_PHOSPHOLIPASE_C_MEDIATED_CASCADE		53	-0.24816589	0.57466066	-0.93449616	0.7909671	1	7831	tags=45%, list=41%, signal=77%	FALSE
REACTOME_PROSTACYCLIN_SIGNALLING_THROUGH_PROSTACYCLIN_RECEPTOR	REACTOME_PROSTACYCLIN_SIGNALLING_THROUGH_PROSTACYCLIN_RECEPTOR		18	-0.30919978	0.55365473	-0.93408585	0.79026014	1	1627	tags=17%, list=9%, signal=18%	FALSE
REACTOME_SHC_RELATED_EVENTS	REACTOME_SHC_RELATED_EVENTS		17	-0.32118142	0.56288344	-0.93001777	0.79837346	1	1274	tags=18%, list=7%, signal=19%	FALSE
REACTOME_ACTIVATION_OF_NF_KAPPAB_IN_B_CELLS	REACTOME_ACTIVATION_OF_NF_KAPPAB_IN_B_CELLS		57	-0.2424833	0.5882353	-0.92713934	0.8033941	1	3810	tags=21%, list=20%, signal=26%	FALSE
REACTOME_VIF_MEDIATED_DEGRADATION_OF_APOBEC3G	REACTOME_VIF_MEDIATED_DEGRADATION_OF_APOBEC3G		46	-0.25153813	0.57567185	-0.9270343	0.8019521	1	864	tags=11%, list=5%, signal=11%	FALSE
KEGG_AUTOIMMUNE_THYROID_DISEASE	KEGG_AUTOIMMUNE_THYROID_DISEASE		43	-0.25339794	0.5858156	-0.9256999	0.8033831	1	6072	tags=37%, list=32%, signal=55%	FALSE
REACTOME_SIGNALING_BY_ERBB2	REACTOME_SIGNALING_BY_ERBB2		97	-0.22019507	0.61243385	-0.92567885	0.8017498	1	5409	tags=30%, list=29%, signal=42%	FALSE
BIOCARTA_CHEMICAL_PATHWAY	BIOCARTA_CHEMICAL_PATHWAY		22	-0.29899853	0.59431136	-0.9213765	0.81027	1	5613	tags=50%, list=30%, signal=71%	FALSE
REACTOME_BMAL1_CLOCK_NPAS2_ACTIVATES_CIRCADIAN_EXPRESSION	REACTOME_BMAL1_CLOCK_NPAS2_ACTIVATES_CIRCADIAN_EXPRESSION		36	-0.26185504	0.5816024	-0.9209153	0.80970603	1	3303	tags=31%, list=17%, signal=37%	FALSE
PID_PTP1B_PATHWAY	PID_PTP1B_PATHWAY		50	-0.24579754	0.60928965	-0.9199388	0.8102816	1	3424	tags=26%, list=18%, signal=32%	FALSE
REACTOME_GLUCOSE_METABOLISM	REACTOME_GLUCOSE_METABOLISM		63	-0.23932117	0.58998644	-0.9192636	0.810173	1	5905	tags=37%, list=31%, signal=53%	FALSE
KEGG_GNRH_SIGNALING_PATHWAY	KEGG_GNRH_SIGNALING_PATHWAY		95	-0.21719304	0.6176084	-0.91863203	0.81008446	1	5905	tags=34%, list=31%, signal=49%	FALSE
PID_ANGIOPOIETIN_RECEPTOR_PATHWAY	PID_ANGIOPOIETIN_RECEPTOR_PATHWAY		49	-0.24391514	0.5982659	-0.91861796	0.8084562	1	4591	tags=29%, list=24%, signal=38%	FALSE
REACTOME_DEPOSITION_OF_NEW_CENPA_CONTAINING_NUCLEOSOMES_AT_THE_CENTROMERE	REACTOME_DEPOSITION_OF_NEW_CENPA_CONTAINING_NUCLEOSOMES_AT_THE_CENTROMERE		45	-0.25084388	0.5828652	-0.91582006	0.8131082	1	4909	tags=38%, list=26%, signal=51%	FALSE
PID_P38_ALPHA_BETA_DOWNSTREAM_PATHWAY	PID_P38_ALPHA_BETA_DOWNSTREAM_PATHWAY		36	-0.25920385	0.60178304	-0.9153036	0.81263524	1	4104	tags=31%, list=22%, signal=39%	FALSE
REACTOME_SIGNALING_BY_CONSTITUTIVELY_ACTIVE_EGFR	REACTOME_SIGNALING_BY_CONSTITUTIVELY_ACTIVE_EGFR		17	-0.31758556	0.5786164	-0.91278	0.8167951	1	5230	tags=35%, list=28%, signal=49%	FALSE
HALLMARK_WNT_BETA_CATENIN_SIGNALING	HALLMARK_WNT_BETA_CATENIN_SIGNALING		42	-0.2500916	0.59615386	-0.9116637	0.8177116	1	2510	tags=21%, list=13%, signal=25%	FALSE
REACTOME_DARPP_32_EVENTS	REACTOME_DARPP_32_EVENTS		24	-0.28555596	0.5904335	-0.9112825	0.81694835	1	7605	tags=58%, list=40%, signal=98%	FALSE
REACTOME_SIGNALING_BY_ERBB4	REACTOME_SIGNALING_BY_ERBB4		87	-0.21978237	0.6586667	-0.91098744	0.8159569	1	5035	tags=28%, list=27%, signal=37%	FALSE
REACTOME_ACTIVATED_TAK1_MEDIATES_P38_MAPK_ACTIVATION	REACTOME_ACTIVATED_TAK1_MEDIATES_P38_MAPK_ACTIVATION		16	-0.31765378	0.5996835	-0.909656	0.81752735	1	7318	tags=50%, list=39%, signal=82%	FALSE
BIOCARTA_HER2_PATHWAY	BIOCARTA_HER2_PATHWAY		22	-0.28954908	0.6050671	-0.9071156	0.8215413	1	2542	tags=23%, list=13%, signal=26%	FALSE
REACTOME_SIGNALING_BY_FGFR	REACTOME_SIGNALING_BY_FGFR		108	-0.21185338	0.6650062	-0.90624243	0.8218475	1	5409	tags=28%, list=29%, signal=39%	FALSE
REACTOME_ADP_SIGNALLING_THROUGH_P2RY12	REACTOME_ADP_SIGNALLING_THROUGH_P2RY12		20	-0.29284295	0.58044165	-0.9053637	0.82215214	1	3969	tags=25%, list=21%, signal=32%	FALSE
REACTOME_NEUROTRANSMITTER_RECEPTOR_BINDING_AND_DOWNSTREAM_TRANSMISSION_IN_THE_POSTSYNAPTIC_CELL	REACTOME_NEUROTRANSMITTER_RECEPTOR_BINDING_AND_DOWNSTREAM_TRANSMISSION_IN_THE_POSTSYNAPTIC_CELL		131	-0.20456395	0.66142684	-0.9048824	0.8215457	1	3997	tags=21%, list=21%, signal=26%	FALSE
PID_IL12_2PATHWAY	PID_IL12_2PATHWAY		62	-0.23027296	0.6282225	-0.9042662	0.82128686	1	6926	tags=40%, list=37%, signal=63%	FALSE
REACTOME_GABA_RECEPTOR_ACTIVATION	REACTOME_GABA_RECEPTOR_ACTIVATION		51	-0.24004398	0.62274617	-0.9042253	0.819763	1	5510	tags=31%, list=29%, signal=44%	FALSE
REACTOME_ACTIVATED_AMPK_STIMULATES_FATTY_ACID_OXIDATION_IN_MUSCLE	REACTOME_ACTIVATED_AMPK_STIMULATES_FATTY_ACID_OXIDATION_IN_MUSCLE		16	-0.31680378	0.58747995	-0.9027598	0.82135934	1	1664	tags=13%, list=9%, signal=14%	FALSE
BIOCARTA_MPR_PATHWAY	BIOCARTA_MPR_PATHWAY		33	-0.26067406	0.6304985	-0.8988312	0.82856095	1	3812	tags=24%, list=20%, signal=30%	FALSE
BIOCARTA_CDC42RAC_PATHWAY	BIOCARTA_CDC42RAC_PATHWAY		15	-0.32031083	0.6137026	-0.8978645	0.82912296	1	5073	tags=47%, list=27%, signal=64%	FALSE
REACTOME_G_ALPHA_Z_SIGNALLING_EVENTS	REACTOME_G_ALPHA_Z_SIGNALLING_EVENTS		43	-0.24913071	0.6179775	-0.8973861	0.8286283	1	5510	tags=30%, list=29%, signal=43%	FALSE
REACTOME_P53_DEPENDENT_G1_DNA_DAMAGE_RESPONSE	REACTOME_P53_DEPENDENT_G1_DNA_DAMAGE_RESPONSE		50	-0.23793209	0.6407355	-0.89666516	0.828638	1	2139	tags=14%, list=11%, signal=16%	FALSE
REACTOME_KINESINS	REACTOME_KINESINS		23	-0.2852782	0.6132353	-0.8950137	0.83067954	1	3294	tags=22%, list=17%, signal=26%	FALSE
REACTOME_G_ALPHA_I_SIGNALLING_EVENTS	REACTOME_G_ALPHA_I_SIGNALLING_EVENTS		182	-0.1962625	0.7117438	-0.89254534	0.83453256	1	5581	tags=29%, list=30%, signal=41%	FALSE
REACTOME_THROMBIN_SIGNALLING_THROUGH_PROTEINASE_ACTIVATED_RECEPTORS_PARS	REACTOME_THROMBIN_SIGNALLING_THROUGH_PROTEINASE_ACTIVATED_RECEPTORS_PARS		29	-0.2672388	0.6393939	-0.8912743	0.835754	1	2302	tags=17%, list=12%, signal=20%	FALSE
BIOCARTA_ACH_PATHWAY	BIOCARTA_ACH_PATHWAY		16	-0.31026673	0.60787404	-0.8912534	0.8341321	1	4591	tags=38%, list=24%, signal=49%	FALSE
REACTOME_AMINE_LIGAND_BINDING_RECEPTORS	REACTOME_AMINE_LIGAND_BINDING_RECEPTORS		36	-0.25283828	0.6454948	-0.8873881	0.84094083	1	5571	tags=31%, list=29%, signal=43%	FALSE
REACTOME_NGF_SIGNALLING_VIA_TRKA_FROM_THE_PLASMA_MEMBRANE	REACTOME_NGF_SIGNALLING_VIA_TRKA_FROM_THE_PLASMA_MEMBRANE		134	-0.20052831	0.710269	-0.88494444	0.84475094	1	5230	tags=27%, list=28%, signal=37%	FALSE
BIOCARTA_NFKB_PATHWAY	BIOCARTA_NFKB_PATHWAY		22	-0.28377628	0.64878047	-0.8848212	0.8433724	1	3810	tags=32%, list=20%, signal=40%	FALSE

KEGG_VALINE_LEUCINE_AND_ISOLEUCINE_DEGRADATION	KEGG_VALINE_LEUCINE_AND_ISOLEUCINE_DEGRADATION		43	-0.24424697	0.6325967	-0.8843481	0.8427839	1	4499	tags=28%, list=24%, signal=37%	FALSE
REACTOME_NEF_MEDIATES_DOWN_MODULATION_OF_CELL_SURFACE_RECEPTORS_BY_RECRUITING_THEM_TO_CLATHRIN_ADAPTERS	REACTOME_NEF_MEDIATES_DOWN_MODULATION_OF_CELL_SURFACE_RECEPTORS_BY_RECRUITING_THEM_TO_CLATHRIN_ADAPTERS		20	-0.2925269	0.6343402	-0.88421065	0.8414161	1	8510	tags=60%, list=45%, signal=109%	FALSE
REACTOME_NUCLEAR_SIGNALING_BY_ERBB4	REACTOME_NUCLEAR_SIGNALING_BY_ERBB4		38	-0.24371724	0.66369045	-0.88192016	0.8447866	1	5022	tags=32%, list=27%, signal=43%	FALSE
REACTOME_SIGNAL_AMPLIFICATION	REACTOME_SIGNAL_AMPLIFICATION		29	-0.26755795	0.6237337	-0.8814054	0.84427196	1	1627	tags=14%, list=9%, signal=15%	FALSE
ST_FAS_SIGNALING_PATHWAY	ST_FAS_SIGNALING_PATHWAY		62	-0.22213031	0.66574967	-0.8811851	0.8431164	1	4205	tags=26%, list=22%, signal=33%	FALSE
KEGG_VASOPRESSIN_REGULATED_WATER_REABSORPTION	KEGG_VASOPRESSIN_REGULATED_WATER_REABSORPTION		43	-0.24223456	0.66429585	-0.8804439	0.8430985	1	4192	tags=23%, list=22%, signal=30%	FALSE
REACTOME_PREFOLDIN_MEDIATED_TRANSFER_OF_SUBSTRATE_TO_CCT_TRIC	REACTOME_PREFOLDIN_MEDIATED_TRANSFER_OF_SUBSTRATE_TO_CCT_TRIC		26	-0.26994726	0.642527	-0.8798413	0.8426904	1	4864	tags=35%, list=26%, signal=47%	FALSE
REACTOME_AUTODEGRADATION_OF_THE_E3_UBIQUITIN_LIGASE_COP1	REACTOME_AUTODEGRADATION_OF_THE_E3_UBIQUITIN_LIGASE_COP1		44	-0.24523494	0.66519827	-0.87977767	0.84119093	1	2816	tags=16%, list=15%, signal=19%	FALSE
REACTOME_REGULATION_OF_IFNA_SIGNALING	REACTOME_REGULATION_OF_IFNA_SIGNALING		19	-0.29240066	0.63722396	-0.87738764	0.844629	1	3706	tags=32%, list=20%, signal=39%	FALSE
REACTOME_GLUCONEOGENESIS	REACTOME_GLUCONEOGENESIS		30	-0.26262906	0.6433878	-0.8768861	0.8440948	1	5293	tags=33%, list=28%, signal=46%	FALSE
PID_CERAMIDE_PATHWAY	PID_CERAMIDE_PATHWAY		48	-0.24072854	0.6693989	-0.87675804	0.8427623	1	3520	tags=25%, list=19%, signal=31%	FALSE
REACTOME_INTEGRIN_ALPHAIIB_BETA3_SIGNALING	REACTOME_INTEGRIN_ALPHAIIB_BETA3_SIGNALING		26	-0.26741257	0.652439	-0.87635714	0.84198743	1	334	tags=12%, list=2%, signal=12%	FALSE
KEGG_INOSITOL_PHOSPHATE_METABOLISM	KEGG_INOSITOL_PHOSPHATE_METABOLISM		54	-0.2306838	0.6790634	-0.87438464	0.84451014	1	3036	tags=22%, list=16%, signal=26%	FALSE
REACTOME_DOWNSTREAM_SIGNALING_OF_ACTIVATED_FGFR	REACTOME_DOWNSTREAM_SIGNALING_OF_ACTIVATED_FGFR		97	-0.20511422	0.6978233	-0.8733436	0.84506375	1	5409	tags=27%, list=29%, signal=37%	FALSE
KEGG_TGF_BETA_SIGNALING_PATHWAY	KEGG_TGF_BETA_SIGNALING_PATHWAY		83	-0.21435508	0.7003891	-0.87262964	0.8449057	1	3788	tags=25%, list=20%, signal=32%	FALSE
BIOCARTA_AKT_PATHWAY	BIOCARTA_AKT_PATHWAY		21	-0.2829313	0.64102566	-0.87010705	0.84866744	1	5022	tags=38%, list=27%, signal=52%	FALSE
REACTOME_CTNNB1_PHOSPHORYLATION_CASCADE	REACTOME_CTNNB1_PHOSPHORYLATION_CASCADE		15	-0.30914256	0.64759034	-0.869321	0.84871644	1	2509	tags=27%, list=13%, signal=31%	FALSE
KEGG_PRION_DISEASES	KEGG_PRION_DISEASES		35	-0.2471262	0.6596343	-0.86842525	0.8490649	1	2566	tags=17%, list=14%, signal=20%	FALSE
REACTOME_EFFECTS_OF_PIP2_HYDROLYSIS	REACTOME_EFFECTS_OF_PIP2_HYDROLYSIS		22	-0.27300045	0.67434716	-0.86728925	0.8497629	1	2461	tags=23%, list=13%, signal=26%	FALSE
REACTOME_MAPK_TARGETS_NUCLEAR_EVENTS_MEDIATED_BY_MAP_KINASES	REACTOME_MAPK_TARGETS_NUCLEAR_EVENTS_MEDIATED_BY_MAP_KINASES		30	-0.25695738	0.65966386	-0.8666166	0.8495677	1	4587	tags=33%, list=24%, signal=44%	FALSE
KEGG_CARDIAC_MUSCLE_CONTRACTION	KEGG_CARDIAC_MUSCLE_CONTRACTION		68	-0.21780321	0.7008197	-0.8653261	0.8507184	1	6070	tags=35%, list=32%, signal=52%	FALSE
REACTOME_P53_INDEPENDENT_G1_S_DNA_DAMAGE_CHECKPOINT	REACTOME_P53_INDEPENDENT_G1_S_DNA_DAMAGE_CHECKPOINT		44	-0.23614612	0.6747851	-0.86511207	0.8495433	1	2816	tags=16%, list=15%, signal=19%	FALSE
REACTOME_REGULATION_OF_KIT_SIGNALING	REACTOME_REGULATION_OF_KIT_SIGNALING		16	-0.3026478	0.6615147	-0.8636322	0.8510715	1	334	tags=13%, list=2%, signal=13%	FALSE
REACTOME_GASTRIN_CREB_SIGNALLING_PATHWAY_VIA_PKC_AND_MAPK	REACTOME_GASTRIN_CREB_SIGNALLING_PATHWAY_VIA_PKC_AND_MAPK		187	-0.18576922	0.76615745	-0.86151475	0.85370976	1	3103	tags=18%, list=16%, signal=22%	FALSE
REACTOME_GLYCOGEN_BREAKDOWN_GLYCOGENOLYSIS	REACTOME_GLYCOGEN_BREAKDOWN_GLYCOGENOLYSIS		16	-0.3002284	0.6575758	-0.86103505	0.85306525	1	5905	tags=44%, list=31%, signal=64%	FALSE
KEGG_GLYOXYLATE_AND_DICARBOXYLATE_METABOLISM	KEGG_GLYOXYLATE_AND_DICARBOXYLATE_METABOLISM		15	-0.30784723	0.6312595	-0.8592614	0.8552212	1	6077	tags=47%, list=32%, signal=69%	FALSE
KEGG_PROTEIN_EXPORT	KEGG_PROTEIN_EXPORT		23	-0.27614826	0.67275494	-0.8575787	0.8570059	1	2718	tags=22%, list=14%, signal=25%	FALSE
REACTOME_SCF_BETA_TRCP_MEDIATED_DEGRADATION_OF_EMI1	REACTOME_SCF_BETA_TRCP_MEDIATED_DEGRADATION_OF_EMI1		46	-0.23434357	0.7053206	-0.85587865	0.8588111	1	5483	tags=28%, list=29%, signal=40%	FALSE
HALLMARK_REACTIVE_OXIGEN_SPECIES_PATHWAY	HALLMARK_REACTIVE_OXIGEN_SPECIES_PATHWAY		48	-0.23044136	0.7216783	-0.85345495	0.86201257	1	3806	tags=21%, list=20%, signal=26%	FALSE
SA_CASPASE_CASCADE	SA_CASPASE_CASCADE		19	-0.28553092	0.68944097	-0.8485658	0.8701489	1	5928	tags=47%, list=31%, signal=69%	FALSE
BIOCARTA_CASPASE_PATHWAY	BIOCARTA_CASPASE_PATHWAY		23	-0.2651332	0.6994048	-0.84796387	0.86977607	1	1226	tags=17%, list=6%, signal=19%	FALSE
KEGG_RENAL_CELL_CARCINOMA	KEGG_RENAL_CELL_CARCINOMA		67	-0.21504883	0.737931	-0.8476772	0.86874145	1	2488	tags=18%, list=13%, signal=21%	FALSE
REACTOME_DOWNSTREAM_SIGNALING_EVENTS_OF_B_CELL_RECEPTOR_BCR	REACTOME_DOWNSTREAM_SIGNALING_EVENTS_OF_B_CELL_RECEPTOR_BCR		88	-0.20212057	0.75195825	-0.8461079	0.8702388	1	5483	tags=26%, list=29%, signal=37%	FALSE
KEGG_GRAFT_VERSUS_HOST_DISEASE	KEGG_GRAFT_VERSUS_HOST_DISEASE		34	-0.24462202	0.71040726	-0.8449782	0.8708193	1	8568	tags=68%, list=45%, signal=124%	FALSE
REACTOME_CLASS_A1_RHODOPSIN_LIKE_RECEPTORS	REACTOME_CLASS_A1_RHODOPSIN_LIKE_RECEPTORS		276	-0.1767564	0.8654708	-0.8434971	0.87214905	1	5289	tags=27%, list=28%, signal=37%	FALSE
PID_ALPHA_SYNUCLEIN_PATHWAY	PID_ALPHA_SYNUCLEIN_PATHWAY		33	-0.24696948	0.71556884	-0.84199214	0.8734849	1	3520	tags=24%, list=19%, signal=30%	FALSE
BIOCARTA_EIF4_PATHWAY	BIOCARTA_EIF4_PATHWAY		22	-0.27319488	0.69349843	-0.8417819	0.87231266	1	9180	tags=59%, list=49%, signal=115%	FALSE
BIOCARTA_STATHMIN_PATHWAY	BIOCARTA_STATHMIN_PATHWAY		19	-0.2840034	0.69110763	-0.83911985	0.87590516	1	7505	tags=42%, list=40%, signal=70%	FALSE
ST_DIFFERENTIATION_PATHWAY_IN_PC12_CELLS	ST_DIFFERENTIATION_PATHWAY_IN_PC12_CELLS		42	-0.22954987	0.71327686	-0.8380422	0.87639654	1	3366	tags=21%, list=18%, signal=26%	FALSE
BIOCARTA_41BB_PATHWAY	BIOCARTA_41BB_PATHWAY		17	-0.2881861	0.6698413	-0.83597064	0.87883955	1	3810	tags=35%, list=20%, signal=44%	FALSE
REACTOME_G_ALPHA_Q_SIGNALLING_EVENTS	REACTOME_G_ALPHA_Q_SIGNALLING_EVENTS		166	-0.18331827	0.8035714	-0.8359637	0.8772624	1	3103	tags=18%, list=16%, signal=21%	FALSE
PID_INSULIN_GLUCOSE_PATHWAY	PID_INSULIN_GLUCOSE_PATHWAY		25	-0.25598058	0.6858476	-0.8344774	0.8786155	1	3911	tags=28%, list=21%, signal=35%	FALSE
REACTOME_PLATELET_HOMEOSTASIS	REACTOME_PLATELET_HOMEOSTASIS		73	-0.20748533	0.76666665	-0.8341874	0.8776396	1	2758	tags=15%, list=15%, signal=18%	FALSE
KEGG_VASCULAR_SMOOTH_MUSCLE_CONTRACTION	KEGG_VASCULAR_SMOOTH_MUSCLE_CONTRACTION		108	-0.1939477	0.7992126	-0.83273226	0.87865674	1	5203	tags=29%, list=28%, signal=39%	FALSE
KEGG_VEGF_SIGNALING_PATHWAY	KEGG_VEGF_SIGNALING_PATHWAY		71	-0.20783983	0.7671602	-0.8303099	0.88160044	1	6105	tags=39%, list=32%, signal=58%	FALSE
BIOCARTA_IGF1_PATHWAY	BIOCARTA_IGF1_PATHWAY		21	-0.26818046	0.6971609	-0.8302246	0.88015753	1	4960	tags=33%, list=26%, signal=45%	FALSE
REACTOME_CITRIC_ACID_CYCLE_TCA_CYCLE	REACTOME_CITRIC_ACID_CYCLE_TCA_CYCLE		17	-0.28383565	0.69952303	-0.82998997	0.87905884	1	8867	tags=65%, list=47%, signal=122%	FALSE
REACTOME_ERK_MAPK_TARGETS	REACTOME_ERK_MAPK_TARGETS		21	-0.26736447	0.73071104	-0.8290394	0.879263	1	4182	tags=29%, list=22%, signal=37%	FALSE
REACTOME_G_ALPHA_S_SIGNALLING_EVENTS	REACTOME_G_ALPHA_S_SIGNALLING_EVENTS		115	-0.19190554	0.8164557	-0.8281908	0.8792246	1	5571	tags=29%, list=29%, signal=40%	FALSE
ST_PHOSPHOINOSITIDE_3_KINASE_PATHWAY	ST_PHOSPHOINOSITIDE_3_KINASE_PATHWAY		36	-0.23784903	0.7341954	-0.82755995	0.8788401	1	4202	tags=25%, list=22%, signal=32%	FALSE
KEGG_PRIMARY_BILE_ACID_BIOSYNTHESIS	KEGG_PRIMARY_BILE_ACID_BIOSYNTHESIS		15	-0.29515168	0.6921875	-0.8205595	0.89039475	1	2771	tags=20%, list=15%, signal=23%	FALSE
KEGG_ALLOGRAFT_REJECTION	KEGG_ALLOGRAFT_REJECTION		33	-0.23968318	0.72713864	-0.82025373	0.8893893	1	8568	tags=64%, list=45%, signal=116%	FALSE
PID_HEDGEHOG_2PATHWAY	PID_HEDGEHOG_2PATHWAY		22	-0.2656686	0.74681526	-0.8172353	0.8931498	1	7018	tags=45%, list=37%, signal=72%	FALSE
REACTOME_DOWNSTREAM_SIGNAL_TRANSDUCTION	REACTOME_DOWNSTREAM_SIGNAL_TRANSDUCTION		91	-0.19729309	0.7939317	-0.8163536	0.8931739	1	5409	tags=29%, list=29%, signal=40%	FALSE
BIOCARTA_TOB1_PATHWAY	BIOCARTA_TOB1_PATHWAY		19	-0.27132517	0.72424245	-0.8140681	0.8956002	1	7839	tags=58%, list=41%, signal=99%	FALSE
SA_TRKA_RECEPTOR	SA_TRKA_RECEPTOR		17	-0.27992564	0.74254316	-0.8112191	0.8991093	1	7815	tags=53%, list=41%, signal=90%	FALSE
REACTOME_REGULATION_OF_APOPTOSIS	REACTOME_REGULATION_OF_APOPTOSIS		53	-0.21375962	0.8088033	-0.80373687	0.9106839	1	1770	tags=11%, list=9%, signal=12%	FALSE
BIOCARTA_STEM_PATHWAY	BIOCARTA_STEM_PATHWAY		15	-0.2877447	0.73619634	-0.80336833	0.90966713	1	1566	tags=13%, list=8%, signal=15%	FALSE
KEGG_LEISHMANIA_INFECTION	KEGG_LEISHMANIA_INFECTION		62	-0.20344634	0.8170564	-0.8020536	0.91026	1	4757	tags=26%, list=25%, signal=34%	FALSE
REACTOME_AQUAPORIN_MEDIATED_TRANSPORT	REACTOME_AQUAPORIN_MEDIATED_TRANSPORT		49	-0.21473528	0.79886687	-0.7999062	0.91240203	1	7113	tags=37%, list=38%, signal=59%	FALSE
PID_AURORA_A_PATHWAY	PID_AURORA_A_PATHWAY		28	-0.24067686	0.79062957	-0.7987034	0.9128533	1	5231	tags=39%, list=28%, signal=54%	FALSE
REACTOME_DESTABILIZATION_OF_MRNA_BY_AUF1_HNRNP_D0	REACTOME_DESTABILIZATION_OF_MRNA_BY_AUF1_HNRNP_D0		45	-0.22077493	0.8051948	-0.797337	0.9136177	1	2816	tags=16%, list=15%, signal=18%	FALSE
PID_FAS_PATHWAY	PID_FAS_PATHWAY		37	-0.22657456	0.8148148	-0.78891444	0.9259038	1	4591	tags=27%, list=24%, signal=36%	FALSE
KEGG_FATTY_ACID_METABOLISM	KEGG_FATTY_ACID_METABOLISM		39	-0.22146629	0.7837838	-0.7883877	0.925184	1	4109	tags=28%, list=22%, signal=36%	FALSE
REACTOME_PIP3_ACTIVATES_AKT_SIGNALING	REACTOME_PIP3_ACTIVATES_AKT_SIGNALING		27	-0.24036852	0.79911375	-0.7871169	0.92565584	1	5035	tags=26%, list=27%, signal=35%	FALSE
BIOCARTA_RAS_PATHWAY	BIOCARTA_RAS_PATHWAY		23	-0.24871111	0.79204893	-0.7862107	0.92558867	1	5073	tags=35%, list=27%, signal=47%	FALSE
KEGG_PANCREATIC_CANCER	KEGG_PANCREATIC_CANCER		68	-0.19750653	0.83741933	-0.78569937	0.9248399	1	5688	tags=34%, list=30%, signal=48%	FALSE
REACTOME_LIGAND_GATED_ION_CHANNEL_TRANSPORT	REACTOME_LIGAND_GATED_ION_CHANNEL_TRANSPORT		21	-0.2529353	0.7741935	-0.7847227	0.9248818	1	7300	tags=48%, list=39%, signal=78%	FALSE
REACTOME_PLATELET_AGGREGATION_PLUG_FORMATION	REACTOME_PLATELET_AGGREGATION_PLUG_FORMATION		34	-0.22707671	0.78008914	-0.7846734	0.9233925	1	334	tags=9%, list=2%, signal=9%	FALSE
HALLMARK_SPERMATOGENESIS	HALLMARK_SPERMATOGENESIS		131	-0.17991278	0.89762795	-0.7842888	0.92241275	1	5698	tags=27%, list=30%, signal=38%	FALSE
BIOCARTA_TNFR2_PATHWAY	BIOCARTA_TNFR2_PATHWAY		17	-0.2721282	0.7893891	-0.78027546	0.92708445	1	5891	tags=47%, list=31%, signal=68%	FALSE
BIOCARTA_PDGF_PATHWAY	BIOCARTA_PDGF_PATHWAY		32	-0.22833781	0.8067227	-0.77921534	0.927112	1	1274	tags=13%, list=7%, signal=13%	FALSE
REACTOME_RIP_MEDIATED_NFKB_ACTIVATION_VIA_DAI	REACTOME_RIP_MEDIATED_NFKB_ACTIVATION_VIA_DAI		16	-0.2665708	0.7856025	-0.7698993	0.93979675	1	3810	tags=25%, list=20%, signal=31%	FALSE
REACTOME_SIGNALING_BY_FGFR_MUTANTS	REACTOME_SIGNALING_BY_FGFR_MUTANTS		42	-0.21186031	0.8375706	-0.7657232	0.94425577	1	2870	tags=17%, list=15%, signal=20%	FALSE
REACTOME_SIGNALING_BY_INSULIN_RECEPTOR	REACTOME_SIGNALING_BY_INSULIN_RECEPTOR		103	-0.17859669	0.91108245	-0.75934637	0.9519287	1	2321	tags=12%, list=12%, signal=13%	FALSE
PID_IL12_STAT4_PATHWAY	PID_IL12_STAT4_PATHWAY		33	-0.22060226	0.83920366	-0.759273	0.95038646	1	5817	tags=36%, list=31%, signal=52%	FALSE
BIOCARTA_TFF_PATHWAY	BIOCARTA_TFF_PATHWAY		20	-0.25420415	0.8133748	-0.7590872	0.9490702	1	7274	tags=55%, list=38%, signal=89%	FALSE
KEGG_GALACTOSE_METABOLISM	KEGG_GALACTOSE_METABOLISM		26	-0.2333775	0.8081571	-0.75843775	0.9483651	1	7351	tags=50%, list=39%, signal=82%	FALSE
REACTOME_METABOLISM_OF_AMINO_ACIDS_AND_DERIVATIVES	REACTOME_METABOLISM_OF_AMINO_ACIDS_AND_DERIVATIVES		185	-0.16244347	0.9521531	-0.7541529	0.95257986	1	5523	tags=26%, list=29%, signal=36%	FALSE
REACTOME_THROMBOXANE_SIGNALLING_THROUGH_TP_RECEPTOR	REACTOME_THROMBOXANE_SIGNALLING_THROUGH_TP_RECEPTOR		21	-0.24296094	0.7939394	-0.75106585	0.9551339	1	1627	tags=14%, list=9%, signal=16%	FALSE
PID_PI3KCI_AKT_PATHWAY	PID_PI3KCI_AKT_PATHWAY		34	-0.21819654	0.84302324	-0.7501679	0.954734	1	5035	tags=29%, list=27%, signal=40%	FALSE
REACTOME_ACTIVATED_NOTCH1_TRANSMITS_SIGNAL_TO_THE_NUCLEUS	REACTOME_ACTIVATED_NOTCH1_TRANSMITS_SIGNAL_TO_THE_NUCLEUS		26	-0.22937714	0.8220588	-0.7488684	0.95497066	1	1770	tags=15%, list=9%, signal=17%	FALSE
REACTOME_SCFSKP2_MEDIATED_DEGRADATION_OF_P27_P21	REACTOME_SCFSKP2_MEDIATED_DEGRADATION_OF_P27_P21		49	-0.20298703	0.87096775	-0.74710214	0.955795	1	5698	tags=29%, list=30%, signal=41%	FALSE
REACTOME_NRAGE_SIGNALS_DEATH_THROUGH_JNK	REACTOME_NRAGE_SIGNALS_DEATH_THROUGH_JNK		42	-0.20578776	0.8814286	-0.7449176	0.9570509	1	1853	tags=14%, list=10%, signal=16%	FALSE
PID_ERBB2_ERBB3_PATHWAY	PID_ERBB2_ERBB3_PATHWAY		44	-0.20434396	0.8654391	-0.74468035	0.9557388	1	5409	tags=32%, list=29%, signal=44%	FALSE
PID_NOTCH_PATHWAY	PID_NOTCH_PATHWAY		58	-0.19320591	0.87967914	-0.74414206	0.9548575	1	5476	tags=33%, list=29%, signal=46%	FALSE
REACTOME_GABA_B_RECEPTOR_ACTIVATION	REACTOME_GABA_B_RECEPTOR_ACTIVATION		37	-0.20933627	0.8653001	-0.7400506	0.95835245	1	5510	tags=27%, list=29%, signal=38%	FALSE
REACTOME_DOWNREGULATION_OF_TGF_BETA_RECEPTOR_SIGNALING	REACTOME_DOWNREGULATION_OF_TGF_BETA_RECEPTOR_SIGNALING		21	-0.23867223	0.8395245	-0.7369042	0.96062595	1	2969	tags=24%, list=16%, signal=28%	FALSE
REACTOME_GPCR_LIGAND_BINDING	REACTOME_GPCR_LIGAND_BINDING		373	-0.14796726	0.99	-0.72909606	0.96857876	1	5289	tags=24%, list=28%, signal=33%	FALSE
BIOCARTA_LONGEVITY_PATHWAY	BIOCARTA_LONGEVITY_PATHWAY		15	-0.26267064	0.8215434	-0.7290897	0.9669727	1	1160	tags=13%, list=6%, signal=14%	FALSE
KEGG_GLYCOLYSIS_GLUCONEOGENESIS	KEGG_GLYCOLYSIS_GLUCONEOGENESIS		59	-0.18826821	0.90796703	-0.72697556	0.96785086	1	5570	tags=29%, list=29%, signal=41%	FALSE
PID_ARF_3PATHWAY	PID_ARF_3PATHWAY		19	-0.24537012	0.8519108	-0.725832	0.9675788	1	5129	tags=32%, list=27%, signal=43%	FALSE
REACTOME_SHC_MEDIATED_SIGNALLING	REACTOME_SHC_MEDIATED_SIGNALLING		15	-0.25542054	0.8388158	-0.7196048	0.97293097	1	1274	tags=13%, list=7%, signal=14%	FALSE
PID_RHODOPSIN_PATHWAY	PID_RHODOPSIN_PATHWAY		23	-0.22839153	0.8549849	-0.7194253	0.97150356	1	2606	tags=17%, list=14%, signal=20%	FALSE
BIOCARTA_NKCELLS_PATHWAY	BIOCARTA_NKCELLS_PATHWAY		17	-0.24793993	0.859375	-0.71881473	0.9706054	1	7583	tags=53%, list=40%, signal=88%	FALSE
REACTOME_FORMATION_OF_TUBULIN_FOLDING_INTERMEDIATES_BY_CCT_TRIC	REACTOME_FORMATION_OF_TUBULIN_FOLDING_INTERMEDIATES_BY_CCT_TRIC		20	-0.23437022	0.8547401	-0.714211	0.97385776	1	4864	tags=40%, list=26%, signal=54%	FALSE
HALLMARK_KRAS_SIGNALING_DN	HALLMARK_KRAS_SIGNALING_DN		196	-0.15487099	0.98194945	-0.7131014	0.97337407	1	5178	tags=25%, list=27%, signal=34%	FALSE
BIOCARTA_TH1TH2_PATHWAY	BIOCARTA_TH1TH2_PATHWAY		19	-0.23721126	0.86752826	-0.7116113	0.97333735	1	8243	tags=58%, list=44%, signal=103%	FALSE
HALLMARK_ADIPOGENESIS	HALLMARK_ADIPOGENESIS		194	-0.15364629	0.97563803	-0.71142226	0.9719274	1	5783	tags=29%, list=31%, signal=42%	FALSE
REACTOME_SIGNALING_BY_SCF_KIT	REACTOME_SIGNALING_BY_SCF_KIT		75	-0.17480324	0.94133335	-0.70828754	0.97355926	1	3019	tags=15%, list=16%, signal=17%	FALSE
PID_TRAIL_PATHWAY	PID_TRAIL_PATHWAY		27	-0.21508546	0.88112926	-0.7057926	0.974418	1	5941	tags=33%, list=31%, signal=49%	FALSE
PID_RXR_VDR_PATHWAY	PID_RXR_VDR_PATHWAY		26	-0.2155241	0.88670695	-0.7001106	0.9781863	1	5534	tags=38%, list=29%, signal=54%	FALSE
REACTOME_IMMUNOREGULATORY_INTERACTIONS_BETWEEN_A_LYMPHOID_AND_A_NON_LYMPHOID_CELL	REACTOME_IMMUNOREGULATORY_INTERACTIONS_BETWEEN_A_LYMPHOID_AND_A_NON_LYMPHOID_CELL		57	-0.18215814	0.93475175	-0.698034	0.9785779	1	6711	tags=42%, list=36%, signal=65%	FALSE
REACTOME_INWARDLY_RECTIFYING_K_CHANNELS	REACTOME_INWARDLY_RECTIFYING_K_CHANNELS		30	-0.21006078	0.8937409	-0.6962815	0.9785744	1	1352	tags=10%, list=7%, signal=11%	FALSE
REACTOME_PI3K_AKT_ACTIVATION	REACTOME_PI3K_AKT_ACTIVATION		36	-0.1994024	0.9314456	-0.6899891	0.98263925	1	5035	tags=25%, list=27%, signal=34%	FALSE
REACTOME_PEROXISOMAL_LIPID_METABOLISM	REACTOME_PEROXISOMAL_LIPID_METABOLISM		19	-0.23128206	0.8880484	-0.68881565	0.98204106	1	4109	tags=21%, list=22%, signal=27%	FALSE
BIOCARTA_CK1_PATHWAY	BIOCARTA_CK1_PATHWAY		17	-0.22965139	0.9049844	-0.6825887	0.9857929	1	8081	tags=59%, list=43%, signal=103%	FALSE
PID_REELIN_PATHWAY	PID_REELIN_PATHWAY		28	-0.20716451	0.90404797	-0.6821677	0.98451626	1	7081	tags=46%, list=37%, signal=74%	FALSE
KEGG_PANTOTHENATE_AND_COA_BIOSYNTHESIS	KEGG_PANTOTHENATE_AND_COA_BIOSYNTHESIS		16	-0.23484251	0.8776435	-0.6719194	0.99091476	1	6307	tags=38%, list=33%, signal=56%	FALSE
REACTOME_TRANSMISSION_ACROSS_CHEMICAL_SYNAPSES	REACTOME_TRANSMISSION_ACROSS_CHEMICAL_SYNAPSES		180	-0.1448748	0.9976825	-0.6705797	0.99034315	1	3997	tags=17%, list=21%, signal=22%	FALSE
REACTOME_COSTIMULATION_BY_THE_CD28_FAMILY	REACTOME_COSTIMULATION_BY_THE_CD28_FAMILY		58	-0.17246535	0.9624478	-0.6687998	0.99010897	1	7706	tags=47%, list=41%, signal=78%	FALSE
REACTOME_ANTIGEN_PROCESSING_CROSS_PRESENTATION	REACTOME_ANTIGEN_PROCESSING_CROSS_PRESENTATION		66	-0.16974427	0.97483444	-0.6666898	0.98998314	1	2238	tags=12%, list=12%, signal=14%	FALSE
SIG_CD40PATHWAYMAP	SIG_CD40PATHWAYMAP		33	-0.19064507	0.938326	-0.660553	0.992735	1	5719	tags=33%, list=30%, signal=48%	FALSE
KEGG_NITROGEN_METABOLISM	KEGG_NITROGEN_METABOLISM		23	-0.20675789	0.93026704	-0.65510964	0.9947062	1	7388	tags=48%, list=39%, signal=78%	FALSE
KEGG_STARCH_AND_SUCROSE_METABOLISM	KEGG_STARCH_AND_SUCROSE_METABOLISM		36	-0.18656583	0.9451852	-0.6501806	0.99629956	1	3483	tags=19%, list=18%, signal=24%	FALSE
REACTOME_G_PROTEIN_ACTIVATION	REACTOME_G_PROTEIN_ACTIVATION		26	-0.20191824	0.9307229	-0.64668524	0.9968165	1	6904	tags=38%, list=37%, signal=61%	FALSE
REACTOME_PI_3K_CASCADE	REACTOME_PI_3K_CASCADE		54	-0.16841368	0.9747899	-0.6418292	0.99809235	1	5149	tags=22%, list=27%, signal=30%	FALSE
REACTOME_G_BETA_GAMMA_SIGNALLING_THROUGH_PLC_BETA	REACTOME_G_BETA_GAMMA_SIGNALLING_THROUGH_PLC_BETA		19	-0.2112052	0.93977815	-0.6268888	1	1	3969	tags=21%, list=21%, signal=27%	FALSE
BIOCARTA_IL12_PATHWAY	BIOCARTA_IL12_PATHWAY		21	-0.20087962	0.94392526	-0.6266723	1	1	7810	tags=52%, list=41%, signal=89%	FALSE
REACTOME_CDK_MEDIATED_PHOSPHORYLATION_AND_REMOVAL_OF_CDC6	REACTOME_CDK_MEDIATED_PHOSPHORYLATION_AND_REMOVAL_OF_CDC6		43	-0.17199299	0.982906	-0.6239908	1	1	1770	tags=9%, list=9%, signal=10%	FALSE
BIOCARTA_GCR_PATHWAY	BIOCARTA_GCR_PATHWAY		19	-0.20536818	0.9356288	-0.6215242	1	1	957	tags=11%, list=5%, signal=11%	FALSE
REACTOME_PYRUVATE_METABOLISM_AND_CITRIC_ACID_TCA_CYCLE	REACTOME_PYRUVATE_METABOLISM_AND_CITRIC_ACID_TCA_CYCLE		37	-0.17630447	0.9717262	-0.6179188	1	1	7905	tags=46%, list=42%, signal=79%	FALSE
REACTOME_GLYCOLYSIS	REACTOME_GLYCOLYSIS		26	-0.18707362	0.9623734	-0.6118752	1	1	5572	tags=35%, list=29%, signal=49%	FALSE
PID_ANTHRAX_PATHWAY	PID_ANTHRAX_PATHWAY		17	-0.20802774	0.943662	-0.6075629	1	1	303	tags=6%, list=2%, signal=6%	FALSE
PID_CXCR3_PATHWAY	PID_CXCR3_PATHWAY		43	-0.16664319	0.9722222	-0.5996122	1	1	6649	tags=33%, list=35%, signal=50%	FALSE
REACTOME_NEGATIVE_REGULATION_OF_FGFR_SIGNALING	REACTOME_NEGATIVE_REGULATION_OF_FGFR_SIGNALING		36	-0.17179526	0.9792285	-0.59310704	1	1	7831	tags=44%, list=41%, signal=76%	FALSE
REACTOME_INHIBITION_OF_VOLTAGE_GATED_CA2_CHANNELS_VIA_GBETA_GAMMA_SUBUNITS	REACTOME_INHIBITION_OF_VOLTAGE_GATED_CA2_CHANNELS_VIA_GBETA_GAMMA_SUBUNITS		24	-0.18600449	0.9470499	-0.59199864	1	1	1352	tags=8%, list=7%, signal=9%	FALSE
REACTOME_CYCLIN_A_B1_ASSOCIATED_EVENTS_DURING_G2_M_TRANSITION	REACTOME_CYCLIN_A_B1_ASSOCIATED_EVENTS_DURING_G2_M_TRANSITION		15	-0.21204087	0.96036583	-0.590003	1	1	5698	tags=33%, list=30%, signal=48%	FALSE
KEGG_CITRATE_CYCLE_TCA_CYCLE	KEGG_CITRATE_CYCLE_TCA_CYCLE		28	-0.1801025	0.97910446	-0.58690304	1	1	7905	tags=46%, list=42%, signal=80%	FALSE
REACTOME_PYRUVATE_METABOLISM	REACTOME_PYRUVATE_METABOLISM		17	-0.20187876	0.9486781	-0.58480066	1	1	6095	tags=35%, list=32%, signal=52%	FALSE
KEGG_PROPANOATE_METABOLISM	KEGG_PROPANOATE_METABOLISM		31	-0.17144854	0.97193503	-0.5836798	1	1	7421	tags=42%, list=39%, signal=69%	FALSE
BIOCARTA_IL17_PATHWAY	BIOCARTA_IL17_PATHWAY		15	-0.20637038	0.95886075	-0.57957643	1	1	1566	tags=13%, list=8%, signal=15%	FALSE
REACTOME_TIE2_SIGNALING	REACTOME_TIE2_SIGNALING		17	-0.19276193	0.9706337	-0.5628761	1	1	4591	tags=24%, list=24%, signal=31%	FALSE
KEGG_SNARE_INTERACTIONS_IN_VESICULAR_TRANSPORT	KEGG_SNARE_INTERACTIONS_IN_VESICULAR_TRANSPORT		38	-0.15515758	0.99425286	-0.54619426	1	1	4575	tags=24%, list=24%, signal=31%	FALSE
PID_IL27_PATHWAY	PID_IL27_PATHWAY		26	-0.16786689	0.9878971	-0.54453117	1	1	5975	tags=35%, list=32%, signal=51%	FALSE
REACTOME_REGULATION_OF_INSULIN_SECRETION_BY_GLUCAGON_LIKE_PEPTIDE1	REACTOME_REGULATION_OF_INSULIN_SECRETION_BY_GLUCAGON_LIKE_PEPTIDE1		40	-0.14918557	0.9970458	-0.53807396	1	1	2789	tags=13%, list=15%, signal=15%	FALSE
REACTOME_GLUCAGON_TYPE_LIGAND_RECEPTORS	REACTOME_GLUCAGON_TYPE_LIGAND_RECEPTORS		32	-0.1592477	0.99402094	-0.53745496	1	1	3969	tags=19%, list=21%, signal=24%	FALSE
KEGG_FRUCTOSE_AND_MANNOSE_METABOLISM	KEGG_FRUCTOSE_AND_MANNOSE_METABOLISM		33	-0.15537132	0.9928775	-0.53196514	1	1	5775	tags=27%, list=31%, signal=39%	FALSE
REACTOME_INHIBITION_OF_INSULIN_SECRETION_BY_ADRENALINE_NORADRENALINE	REACTOME_INHIBITION_OF_INSULIN_SECRETION_BY_ADRENALINE_NORADRENALINE		24	-0.16214503	0.99242425	-0.51521707	1	1	6729	tags=33%, list=36%, signal=52%	FALSE
REACTOME_LATENT_INFECTION_OF_HOMO_SAPIENS_WITH_MYCOBACTERIUM_TUBERCULOSIS	REACTOME_LATENT_INFECTION_OF_HOMO_SAPIENS_WITH_MYCOBACTERIUM_TUBERCULOSIS		31	-0.14892049	0.99415207	-0.5018681	1	1	7964	tags=48%, list=42%, signal=84%	FALSE
REACTOME_REGULATION_OF_ORNITHINE_DECARBOXYLASE_ODC	REACTOME_REGULATION_OF_ORNITHINE_DECARBOXYLASE_ODC		45	-0.1348995	0.9986053	-0.501519	1	1	2816	tags=11%, list=15%, signal=13%	FALSE
KEGG_PROTEASOME	KEGG_PROTEASOME		40	-0.1340145	1	-0.4778764	1	1	6189	tags=25%, list=33%, signal=37%	FALSE
REACTOME_TRAFFICKING_OF_GLUR2_CONTAINING_AMPA_RECEPTORS	REACTOME_TRAFFICKING_OF_GLUR2_CONTAINING_AMPA_RECEPTORS		15	-0.15684807	0.9921384	-0.44237995	1	1	7856	tags=47%, list=42%, signal=80%	FALSE
REACTOME_CROSS_PRESENTATION_OF_SOLUBLE_EXOGENOUS_ANTIGENS_ENDOSOMES	REACTOME_CROSS_PRESENTATION_OF_SOLUBLE_EXOGENOUS_ANTIGENS_ENDOSOMES		41	-0.1070661	1	-0.38908672	0.9998237	1	864	tags=5%, list=5%, signal=5%	FALSE

Supplementary Table 15: Negative Pearson Correlation of Expression GSEA report from Activity Area

NAME	GS<br>follow link to MSigDB	GS DETAILS	SIZE	ES	NOM p-val	NES	FDR q-val	FWER p-val	RANK AT MAX	LEADING EDGE	Splicing/mRNA Processing
REACTOME_PROCESSING_OF_CAPPED_INTRON_CONTAINING_PRE_MRNA	REACTOME_PROCESSING_OF_CAPPED_INTRON_CONTAINING_PRE_MRNA	Details ...	129	-0.6459573	0	-2.8447914	0	0	4186	tags=67%, list=22%, signal=85%	TRUE
REACTOME_MRNA_SPLICING	REACTOME_MRNA_SPLICING	Details ...	101	-0.674756	0	-2.827584	0	0	4186	tags=72%, list=22%, signal=92%	TRUE
REACTOME_MRNA_PROCESSING	REACTOME_MRNA_PROCESSING	Details ...	146	-0.62379545	0	-2.75242	0	0	4186	tags=62%, list=22%, signal=79%	TRUE
KEGG_SPLICEOSOME	KEGG_SPLICEOSOME	Details ...	120	-0.6264446	0	-2.7331548	0	0	4186	tags=68%, list=22%, signal=86%	TRUE
REACTOME_3_UTR_MEDIATED_TRANSLATIONAL_REGULATION	REACTOME_3_UTR_MEDIATED_TRANSLATIONAL_REGULATION	Details ...	52	-0.7109544	0	-2.7049658	0	0	2637	tags=65%, list=14%, signal=76%	FALSE
HALLMARK_MYC_TARGETS_V1	HALLMARK_MYC_TARGETS_V1	Details ...	178	-0.59674025	0	-2.7011209	0	0	5421	tags=67%, list=29%, signal=94%	FALSE
REACTOME_PEPTIDE_CHAIN_ELONGATION	REACTOME_PEPTIDE_CHAIN_ELONGATION	Details ...	38	-0.7630061	0	-2.6900802	0	0	1770	tags=68%, list=9%, signal=75%	FALSE
REACTOME_INFLUENZA_VIRAL_RNA_TRANSCRIPTION_AND_REPLICATION	REACTOME_INFLUENZA_VIRAL_RNA_TRANSCRIPTION_AND_REPLICATION	Details ...	54	-0.7010146	0	-2.6838734	0	0	2975	tags=61%, list=16%, signal=72%	FALSE
REACTOME_NONSENSE_MEDIATED_DECAY_ENHANCED_BY_THE_EXON_JUNCTION_COMPLEX	REACTOME_NONSENSE_MEDIATED_DECAY_ENHANCED_BY_THE_EXON_JUNCTION_COMPLEX	Details ...	58	-0.6900549	0	-2.6715739	0	0	4198	tags=74%, list=22%, signal=95%	TRUE
KEGG_RIBOSOME	KEGG_RIBOSOME	Details ...	40	-0.74578923	0	-2.6543474	0	0	2975	tags=75%, list=16%, signal=89%	FALSE
REACTOME_INFLUENZA_LIFE_CYCLE	REACTOME_INFLUENZA_LIFE_CYCLE	Details ...	86	-0.6276603	0	-2.6072252	0	0	2975	tags=51%, list=16%, signal=60%	FALSE
REACTOME_TRANSPORT_OF_MATURE_TRANSCRIPT_TO_CYTOPLASM	REACTOME_TRANSPORT_OF_MATURE_TRANSCRIPT_TO_CYTOPLASM	Details ...	51	-0.67944026	0	-2.5576494	0	0	2961	tags=65%, list=16%, signal=77%	FALSE
HALLMARK_E2F_TARGETS	HALLMARK_E2F_TARGETS	Details ...	191	-0.5493739	0	-2.5335188	0	0	4668	tags=54%, list=25%, signal=72%	FALSE
REACTOME_CLEAVAGE_OF_GROWING_TRANSCRIPT_IN_THE_TERMINATION_REGION_	REACTOME_CLEAVAGE_OF_GROWING_TRANSCRIPT_IN_THE_TERMINATION_REGION_	Details ...	40	-0.6879279	0	-2.4780562	0	0	4046	tags=80%, list=21%, signal=102%	TRUE
REACTOME_TRANSLATION	REACTOME_TRANSLATION	Details ...	90	-0.586893	0	-2.4673183	0	0	3999	tags=56%, list=21%, signal=70%	FALSE
REACTOME_METABOLISM_OF_RNA	REACTOME_METABOLISM_OF_RNA	Details ...	195	-0.5358157	0	-2.4670599	0	0	4198	tags=49%, list=22%, signal=63%	FALSE
REACTOME_MRNA_3_END_PROCESSING	REACTOME_MRNA_3_END_PROCESSING	Details ...	32	-0.7020908	0	-2.3739917	0	0	4388	tags=88%, list=23%, signal=114%	TRUE
REACTOME_METABOLISM_OF_MRNA	REACTOME_METABOLISM_OF_MRNA	Details ...	154	-0.5291658	0	-2.3704436	0	0	4198	tags=48%, list=22%, signal=61%	FALSE
REACTOME_RNA_POL_II_TRANSCRIPTION	REACTOME_RNA_POL_II_TRANSCRIPTION	Details ...	95	-0.55627656	0	-2.340773	0	0	4466	tags=59%, list=24%, signal=77%	FALSE
REACTOME_MRNA_SPLICING_MINOR_PATHWAY	REACTOME_MRNA_SPLICING_MINOR_PATHWAY	Details ...	41	-0.64123046	0	-2.3312953	4.16E-05	0.001	4154	tags=61%, list=22%, signal=78%	TRUE
REACTOME_G2_M_CHECKPOINTS	REACTOME_G2_M_CHECKPOINTS	Details ...	40	-0.6481518	0	-2.32647	3.96E-05	0.001	3243	tags=50%, list=17%, signal=60%	FALSE
HALLMARK_MYC_TARGETS_V2	HALLMARK_MYC_TARGETS_V2	Details ...	55	-0.6123377	0	-2.3241751	3.78E-05	0.001	5173	tags=75%, list=27%, signal=102%	FALSE
REACTOME_ACTIVATION_OF_ATR_IN_RESPONSE_TO_REPLICATION_STRESS	REACTOME_ACTIVATION_OF_ATR_IN_RESPONSE_TO_REPLICATION_STRESS	Details ...	35	-0.6609478	0	-2.3127935	3.61E-05	0.001	3243	tags=54%, list=17%, signal=65%	FALSE
REACTOME_SRP_DEPENDENT_COTRANSLATIONAL_PROTEIN_TARGETING_TO_MEMBRANE	REACTOME_SRP_DEPENDENT_COTRANSLATIONAL_PROTEIN_TARGETING_TO_MEMBRANE	Details ...	61	-0.5901321	0	-2.306744	3.46E-05	0.001	2975	tags=49%, list=16%, signal=58%	FALSE
KEGG_RNA_DEGRADATION	KEGG_RNA_DEGRADATION	Details ...	56	-0.5908727	0	-2.2964482	3.33E-05	0.001	4246	tags=59%, list=22%, signal=76%	FALSE
KEGG_PRIMARY_IMMUNODEFICIENCY	KEGG_PRIMARY_IMMUNODEFICIENCY	Details ...	34	-0.6530874	0	-2.2850645	3.20E-05	0.001	3837	tags=62%, list=20%, signal=77%	FALSE
REACTOME_TRANSCRIPTION	REACTOME_TRANSCRIPTION	Details ...	172	-0.500844	0	-2.275217	3.08E-05	0.001	4490	tags=53%, list=24%, signal=70%	FALSE
HALLMARK_G2M_CHECKPOINT	HALLMARK_G2M_CHECKPOINT	Details ...	196	-0.48244557	0	-2.2251072	9.08E-05	0.003	4527	tags=48%, list=24%, signal=63%	FALSE
REACTOME_ACTIVATION_OF_THE_PRE_REPLICATIVE_COMPLEX	REACTOME_ACTIVATION_OF_THE_PRE_REPLICATIVE_COMPLEX	Details ...	30	-0.67085505	0	-2.2219756	8.77E-05	0.003	3144	tags=57%, list=17%, signal=68%	FALSE
KEGG_DNA_REPLICATION	KEGG_DNA_REPLICATION	Details ...	36	-0.62915367	0	-2.216034	8.48E-05	0.003	4338	tags=64%, list=23%, signal=83%	FALSE
REACTOME_METABOLISM_OF_NON_CODING_RNA	REACTOME_METABOLISM_OF_NON_CODING_RNA	Details ...	44	-0.59580845	0	-2.18121	8.20E-05	0.003	2912	tags=50%, list=15%, signal=59%	TRUE
KEGG_BASE_EXCISION_REPAIR	KEGG_BASE_EXCISION_REPAIR	Details ...	31	-0.6308366	0	-2.152072	1.06E-04	0.004	2173	tags=45%, list=12%, signal=51%	FALSE
REACTOME_ACTIVATION_OF_THE_MRNA_UPON_BINDING_OF_THE_CAP_BINDING_COMPLEX_AND_EIFS_AND_SUBSEQUENT_BINDING_TO_43S	REACTOME_ACTIVATION_OF_THE_MRNA_UPON_BINDING_OF_THE_CAP_BINDING_COMPLEX_AND_EIFS_AND_SUBSEQUENT_BINDING_TO_43S	Details ...	30	-0.63027275	0	-2.1115923	3.09E-04	0.012	5411	tags=77%, list=29%, signal=107%	FALSE
REACTOME_DNA_REPAIR	REACTOME_DNA_REPAIR	Details ...	98	-0.49934965	0	-2.1099505	3.25E-04	0.013	6581	tags=60%, list=35%, signal=92%	FALSE
REACTOME_FORMATION_OF_THE_TERNARY_COMPLEX_AND_SUBSEQUENTLY_THE_43S_COMPLEX	REACTOME_FORMATION_OF_THE_TERNARY_COMPLEX_AND_SUBSEQUENTLY_THE_43S_COMPLEX	Details ...	26	-0.6539195	0	-2.0930018	3.65E-04	0.015	5179	tags=81%, list=27%, signal=111%	FALSE
REACTOME_DNA_STRAND_ELONGATION	REACTOME_DNA_STRAND_ELONGATION	Details ...	30	-0.6126199	0	-2.0567443	6.63E-04	0.028	4933	tags=63%, list=26%, signal=86%	FALSE
REACTOME_DEADENYLATION_DEPENDENT_MRNA_DECAY	REACTOME_DEADENYLATION_DEPENDENT_MRNA_DECAY	Details ...	40	-0.5665749	0	-2.0430028	8.76E-04	0.038	4191	tags=55%, list=22%, signal=71%	TRUE
REACTOME_DESTABILIZATION_OF_MRNA_BY_KSRP	REACTOME_DESTABILIZATION_OF_MRNA_BY_KSRP	Details ...	17	-0.67742777	0	-1.9799412	0.00228441	0.096	4191	tags=65%, list=22%, signal=83%	TRUE
PID_E2F_PATHWAY	PID_E2F_PATHWAY	Details ...	71	-0.49296692	0	-1.9762061	0.002463237	0.106	4610	tags=49%, list=24%, signal=65%	FALSE
REACTOME_RNA_POL_III_TRANSCRIPTION_INITIATION_FROM_TYPE_2_PROMOTER	REACTOME_RNA_POL_III_TRANSCRIPTION_INITIATION_FROM_TYPE_2_PROMOTER	Details ...	23	-0.6256294	0	-1.9743226	0.002550729	0.113	4490	tags=61%, list=24%, signal=80%	FALSE
REACTOME_RNA_POL_III_TRANSCRIPTION	REACTOME_RNA_POL_III_TRANSCRIPTION	Details ...	33	-0.57372385	0	-1.9662645	0.00267585	0.122	6042	tags=67%, list=32%, signal=98%	FALSE
BIOCARTA_RARRXR_PATHWAY	BIOCARTA_RARRXR_PATHWAY	Details ...	15	-0.68678546	0.001594896	-1.9656578	0.002632637	0.123	3909	tags=53%, list=21%, signal=67%	FALSE
REACTOME_MITOTIC_G1_G1_S_PHASES	REACTOME_MITOTIC_G1_G1_S_PHASES	Details ...	125	-0.4476225	0	-1.9473041	0.003105803	0.147	5547	tags=48%, list=29%, signal=67%	FALSE
PID_BARD1_PATHWAY	PID_BARD1_PATHWAY	Details ...	28	-0.582634	0.00148368	-1.9460182	0.003054731	0.148	4731	tags=61%, list=25%, signal=81%	FALSE
REACTOME_CELL_CYCLE_CHECKPOINTS	REACTOME_CELL_CYCLE_CHECKPOINTS	Details ...	105	-0.4543188	0	-1.9434242	0.003193238	0.157	4230	tags=37%, list=22%, signal=48%	FALSE
REACTOME_G0_AND_EARLY_G1	REACTOME_G0_AND_EARLY_G1	Details ...	23	-0.6122832	0	-1.943007	0.003179075	0.16	6398	tags=83%, list=34%, signal=125%	FALSE
REACTOME_CELL_CYCLE_MITOTIC	REACTOME_CELL_CYCLE_MITOTIC	Details ...	296	-0.40451768	0	-1.9408631	0.003293238	0.17	5595	tags=45%, list=30%, signal=63%	FALSE
REACTOME_PROCESSING_OF_CAPPED_INTRONLESS_PRE_MRNA	REACTOME_PROCESSING_OF_CAPPED_INTRONLESS_PRE_MRNA	Details ...	21	-0.6252946	0	-1.9383767	0.00340256	0.18	6219	tags=90%, list=33%, signal=135%	TRUE
REACTOME_NUCLEOTIDE_EXCISION_REPAIR	REACTOME_NUCLEOTIDE_EXCISION_REPAIR	Details ...	44	-0.52950406	0	-1.9374983	0.00333312	0.18	6042	tags=61%, list=32%, signal=90%	FALSE
REACTOME_GLOBAL_GENOMIC_NER_GG_NER	REACTOME_GLOBAL_GENOMIC_NER_GG_NER	Details ...	29	-0.5881858	0.001517451	-1.9330552	0.0033851	0.187	6005	tags=69%, list=32%, signal=101%	FALSE
KEGG_NUCLEOTIDE_EXCISION_REPAIR	KEGG_NUCLEOTIDE_EXCISION_REPAIR	Details ...	41	-0.5419974	0	-1.9323093	0.003335366	0.188	6005	tags=66%, list=32%, signal=96%	FALSE
REACTOME_TRANSPORT_OF_MATURE_MRNA_DERIVED_FROM_AN_INTRONLESS_TRANSCRIPT	REACTOME_TRANSPORT_OF_MATURE_MRNA_DERIVED_FROM_AN_INTRONLESS_TRANSCRIPT	Details ...	31	-0.5749481	0	-1.9279239	0.003532693	0.202	2912	tags=48%, list=15%, signal=57%	FALSE
BIOCARTA_MCM_PATHWAY	BIOCARTA_MCM_PATHWAY	Details ...	18	-0.64118826	0.001531394	-1.9233309	0.003803686	0.22	3144	tags=56%, list=17%, signal=67%	FALSE
PID_P53_REGULATION_PATHWAY	PID_P53_REGULATION_PATHWAY	Details ...	58	-0.49858072	0	-1.9229271	0.003749337	0.22	6609	tags=62%, list=35%, signal=95%	FALSE
REACTOME_RNA_POL_III_TRANSCRIPTION_INITIATION_FROM_TYPE_3_PROMOTER	REACTOME_RNA_POL_III_TRANSCRIPTION_INITIATION_FROM_TYPE_3_PROMOTER	Details ...	26	-0.57777995	0	-1.9221485	0.003727102	0.221	6737	tags=73%, list=36%, signal=113%	FALSE
PID_FANCONI_PATHWAY	PID_FANCONI_PATHWAY		47	-0.5199466	0	-1.9142041	0.004099174	0.246	4583	tags=51%, list=24%, signal=67%	FALSE
KEGG_RNA_POLYMERASE	KEGG_RNA_POLYMERASE		27	-0.5800071	0.003067485	-1.8938987	0.005039014	0.295	6930	tags=74%, list=37%, signal=117%	FALSE
REACTOME_HIV_LIFE_CYCLE	REACTOME_HIV_LIFE_CYCLE		107	-0.4393655	0	-1.8826659	0.005990841	0.338	3281	tags=38%, list=17%, signal=46%	FALSE
REACTOME_CELL_CYCLE	REACTOME_CELL_CYCLE		370	-0.38327965	0	-1.868475	0.007256683	0.389	5547	tags=45%, list=29%, signal=62%	FALSE
KEGG_MISMATCH_REPAIR	KEGG_MISMATCH_REPAIR		22	-0.59075665	0.00311042	-1.8597987	0.007959246	0.427	6005	tags=73%, list=32%, signal=106%	FALSE
REACTOME_REGULATION_OF_HYPOXIA_INDUCIBLE_FACTOR_HIF_BY_OXYGEN	REACTOME_REGULATION_OF_HYPOXIA_INDUCIBLE_FACTOR_HIF_BY_OXYGEN		22	-0.59105945	0.003149606	-1.8533489	0.00842618	0.452	2621	tags=50%, list=14%, signal=58%	FALSE
PID_MYC_ACTIV_PATHWAY	PID_MYC_ACTIV_PATHWAY		72	-0.463544	0	-1.848791	0.008659091	0.465	5308	tags=53%, list=28%, signal=73%	FALSE
REACTOME_APC_CDC20_MEDIATED_DEGRADATION_OF_NEK2A	REACTOME_APC_CDC20_MEDIATED_DEGRADATION_OF_NEK2A		19	-0.6144386	0.00462963	-1.8455536	0.008912786	0.479	3871	tags=58%, list=20%, signal=73%	FALSE
PID_ATR_PATHWAY	PID_ATR_PATHWAY		39	-0.51631737	0	-1.8398156	0.009559088	0.509	4414	tags=46%, list=23%, signal=60%	FALSE
REACTOME_GENERATION_OF_SECOND_MESSENGER_MOLECULES	REACTOME_GENERATION_OF_SECOND_MESSENGER_MOLECULES		24	-0.5714693	0	-1.8397971	0.009412025	0.509	6756	tags=71%, list=36%, signal=110%	FALSE
SIG_BCR_SIGNALING_PATHWAY	SIG_BCR_SIGNALING_PATHWAY		46	-0.50174004	0	-1.839014	0.009308457	0.51	2195	tags=35%, list=12%, signal=39%	FALSE
REACTOME_TRANSCRIPTION_COUPLED_NER_TC_NER	REACTOME_TRANSCRIPTION_COUPLED_NER_TC_NER		39	-0.51289827	0.002853067	-1.8380181	0.009322501	0.516	6042	tags=59%, list=32%, signal=87%	FALSE
REACTOME_MRNA_CAPPING	REACTOME_MRNA_CAPPING		26	-0.5544869	0.00152439	-1.8366168	0.009297967	0.522	6737	tags=62%, list=36%, signal=96%	TRUE
REACTOME_DOUBLE_STRAND_BREAK_REPAIR	REACTOME_DOUBLE_STRAND_BREAK_REPAIR		21	-0.59211785	0.001545595	-1.8360096	0.009274426	0.526	4414	tags=62%, list=23%, signal=81%	FALSE
KEGG_HOMOLOGOUS_RECOMBINATION	KEGG_HOMOLOGOUS_RECOMBINATION		28	-0.5610191	0	-1.832163	0.00948239	0.541	4414	tags=61%, list=23%, signal=79%	FALSE
REACTOME_RESPIRATORY_ELECTRON_TRANSPORT_ATP_SYNTHESIS_BY_CHEMIOSMOTIC_COUPLING_AND_HEAT_PRODUCTION_BY_UNCOUPLING_PROTEINS_	REACTOME_RESPIRATORY_ELECTRON_TRANSPORT_ATP_SYNTHESIS_BY_CHEMIOSMOTIC_COUPLING_AND_HEAT_PRODUCTION_BY_UNCOUPLING_PROTEINS_		72	-0.45674604	0	-1.8294467	0.009576488	0.553	7033	tags=61%, list=37%, signal=97%	FALSE
REACTOME_ANTIGEN_ACTIVATES_B_CELL_RECEPTOR_LEADING_TO_GENERATION_OF_SECOND_MESSENGERS	REACTOME_ANTIGEN_ACTIVATES_B_CELL_RECEPTOR_LEADING_TO_GENERATION_OF_SECOND_MESSENGERS		29	-0.5541036	0	-1.8289685	0.009525351	0.556	1913	tags=38%, list=10%, signal=42%	FALSE
KEGG_CELL_CYCLE	KEGG_CELL_CYCLE		119	-0.4221169	0	-1.8286151	0.009464556	0.558	5698	tags=50%, list=30%, signal=72%	FALSE
REACTOME_EXTENSION_OF_TELOMERES	REACTOME_EXTENSION_OF_TELOMERES		27	-0.54876375	0.001526718	-1.8224795	0.010056827	0.589	6393	tags=70%, list=34%, signal=106%	FALSE
REACTOME_APC_C_CDC20_MEDIATED_DEGRADATION_OF_CYCLIN_B	REACTOME_APC_C_CDC20_MEDIATED_DEGRADATION_OF_CYCLIN_B		17	-0.6231879	0.003144654	-1.8213794	0.009967145	0.59	3871	tags=53%, list=20%, signal=67%	FALSE
REACTOME_DEADENYLATION_OF_MRNA	REACTOME_DEADENYLATION_OF_MRNA		15	-0.63645905	0.001536098	-1.812218	0.011163083	0.635	6372	tags=87%, list=34%, signal=131%	TRUE
REACTOME_DNA_REPLICATION	REACTOME_DNA_REPLICATION		181	-0.39676428	0	-1.8099257	0.011371603	0.647	5760	tags=44%, list=30%, signal=63%	FALSE
REACTOME_S_PHASE	REACTOME_S_PHASE		102	-0.42532387	0	-1.8025489	0.01222582	0.684	5542	tags=46%, list=29%, signal=65%	FALSE
REACTOME_PKB_MEDIATED_EVENTS	REACTOME_PKB_MEDIATED_EVENTS		26	-0.55317414	0.004552352	-1.7993017	0.012513493	0.697	2321	tags=31%, list=12%, signal=35%	FALSE
REACTOME_FANCONI_ANEMIA_PATHWAY	REACTOME_FANCONI_ANEMIA_PATHWAY		21	-0.5830202	0.001508296	-1.7976738	0.012580811	0.705	4583	tags=52%, list=24%, signal=69%	FALSE
REACTOME_ASSOCIATION_OF_TRIC_CCT_WITH_TARGET_PROTEINS_DURING_BIOSYNTHESIS	REACTOME_ASSOCIATION_OF_TRIC_CCT_WITH_TARGET_PROTEINS_DURING_BIOSYNTHESIS		25	-0.55054605	0.002971768	-1.7850266	0.014514579	0.755	4876	tags=48%, list=26%, signal=65%	FALSE
REACTOME_RNA_POL_I_RNA_POL_III_AND_MITOCHONDRIAL_TRANSCRIPTION	REACTOME_RNA_POL_I_RNA_POL_III_AND_MITOCHONDRIAL_TRANSCRIPTION		90	-0.42714897	0	-1.7801294	0.015255692	0.776	6042	tags=57%, list=32%, signal=83%	FALSE
REACTOME_LATE_PHASE_OF_HIV_LIFE_CYCLE	REACTOME_LATE_PHASE_OF_HIV_LIFE_CYCLE		95	-0.42196265	0	-1.779888	0.015132493	0.777	7420	tags=71%, list=39%, signal=116%	FALSE
REACTOME_M_G1_TRANSITION	REACTOME_M_G1_TRANSITION		75	-0.4386461	0	-1.7792546	0.015083499	0.781	3144	tags=31%, list=17%, signal=37%	FALSE
PID_LPA4_PATHWAY	PID_LPA4_PATHWAY		15	-0.62056637	0.008278145	-1.7721212	0.016457064	0.818	5203	tags=60%, list=28%, signal=83%	FALSE
REACTOME_RNA_POL_III_CHAIN_ELONGATION	REACTOME_RNA_POL_III_CHAIN_ELONGATION		17	-0.6116134	0.001481482	-1.7697933	0.01663129	0.823	4490	tags=53%, list=24%, signal=69%	FALSE
REACTOME_TRANSPORT_OF_RIBONUCLEOPROTEINS_INTO_THE_HOST_NUCLEUS	REACTOME_TRANSPORT_OF_RIBONUCLEOPROTEINS_INTO_THE_HOST_NUCLEUS		26	-0.5443702	0	-1.766159	0.017028807	0.83	2912	tags=42%, list=15%, signal=50%	FALSE
REACTOME_G1_S_TRANSITION	REACTOME_G1_S_TRANSITION		101	-0.41937256	0	-1.7654934	0.01694115	0.834	5698	tags=46%, list=30%, signal=65%	FALSE
REACTOME_DESTABILIZATION_OF_MRNA_BY_BRF1	REACTOME_DESTABILIZATION_OF_MRNA_BY_BRF1		17	-0.6001382	0.009230769	-1.7641793	0.016989687	0.84	4587	tags=71%, list=24%, signal=93%	FALSE
REACTOME_RNA_POL_II_PRE_TRANSCRIPTION_EVENTS	REACTOME_RNA_POL_II_PRE_TRANSCRIPTION_EVENTS		55	-0.46070692	0.004043127	-1.7628341	0.01709122	0.846	6831	tags=67%, list=36%, signal=105%	FALSE
REACTOME_PKA_MEDIATED_PHOSPHORYLATION_OF_CREB	REACTOME_PKA_MEDIATED_PHOSPHORYLATION_OF_CREB		17	-0.5926713	0.0015625	-1.7540498	0.018713018	0.872	5203	tags=53%, list=28%, signal=73%	FALSE
REACTOME_RESPIRATORY_ELECTRON_TRANSPORT	REACTOME_RESPIRATORY_ELECTRON_TRANSPORT		58	-0.45098653	0	-1.7521731	0.018924199	0.879	6741	tags=57%, list=36%, signal=88%	FALSE
PID_HIF1A_PATHWAY	PID_HIF1A_PATHWAY		18	-0.5864772	0.007751938	-1.7511356	0.018968156	0.883	2621	tags=39%, list=14%, signal=45%	FALSE
KEGG_PARKINSONS_DISEASE	KEGG_PARKINSONS_DISEASE		102	-0.4127374	0.00127551	-1.746253	0.019766709	0.897	7001	tags=60%, list=37%, signal=94%	FALSE
BIOCARTA_HCMV_PATHWAY	BIOCARTA_HCMV_PATHWAY		17	-0.5989846	0	-1.74228	0.020309461	0.904	957	tags=29%, list=5%, signal=31%	FALSE
REACTOME_REGULATION_OF_GLUCOKINASE_BY_GLUCOKINASE_REGULATORY_PROTEIN	REACTOME_REGULATION_OF_GLUCOKINASE_BY_GLUCOKINASE_REGULATORY_PROTEIN		26	-0.5343809	0.003030303	-1.7415664	0.020203546	0.905	2912	tags=42%, list=15%, signal=50%	FALSE
REACTOME_TCR_SIGNALING	REACTOME_TCR_SIGNALING		47	-0.46680793	0.002728513	-1.7393978	0.020519182	0.909	6763	tags=60%, list=36%, signal=93%	FALSE
KEGG_UBIQUITIN_MEDIATED_PROTEOLYSIS	KEGG_UBIQUITIN_MEDIATED_PROTEOLYSIS		128	-0.3931308	0	-1.7392068	0.020361785	0.911	6414	tags=57%, list=34%, signal=86%	FALSE
REACTOME_RNA_POL_III_TRANSCRIPTION_TERMINATION	REACTOME_RNA_POL_III_TRANSCRIPTION_TERMINATION		19	-0.58189046	0.010835913	-1.7381135	0.02042347	0.911	4490	tags=47%, list=24%, signal=62%	FALSE
BIOCARTA_AKAPCENTROSOME_PATHWAY	BIOCARTA_AKAPCENTROSOME_PATHWAY		15	-0.61590874	0.004807693	-1.7371806	0.020431742	0.913	2857	tags=47%, list=15%, signal=55%	FALSE
REACTOME_SYNTHESIS_OF_DNA	REACTOME_SYNTHESIS_OF_DNA		87	-0.42258757	0.001278772	-1.7367423	0.020330084	0.915	5493	tags=44%, list=29%, signal=61%	FALSE
REACTOME_DESTABILIZATION_OF_MRNA_BY_TRISTETRAPROLIN_TTP	REACTOME_DESTABILIZATION_OF_MRNA_BY_TRISTETRAPROLIN_TTP		17	-0.5869548	0.006504065	-1.7338213	0.02088567	0.922	4587	tags=71%, list=24%, signal=93%	FALSE
REACTOME_INTERACTIONS_OF_VPR_WITH_HOST_CELLULAR_PROTEINS	REACTOME_INTERACTIONS_OF_VPR_WITH_HOST_CELLULAR_PROTEINS		31	-0.51447475	0.003016591	-1.7336277	0.020716423	0.922	7420	tags=74%, list=39%, signal=122%	FALSE
REACTOME_FORMATION_OF_RNA_POL_II_ELONGATION_COMPLEX_	REACTOME_FORMATION_OF_RNA_POL_II_ELONGATION_COMPLEX_		39	-0.47910428	0.005970149	-1.7328492	0.020672387	0.923	6737	tags=67%, list=36%, signal=103%	FALSE
BIOCARTA_PML_PATHWAY	BIOCARTA_PML_PATHWAY		17	-0.5934059	0.003159558	-1.7273479	0.021848222	0.931	4496	tags=53%, list=24%, signal=69%	FALSE
KEGG_HUNTINGTONS_DISEASE	KEGG_HUNTINGTONS_DISEASE		157	-0.38778362	0	-1.7273426	0.021642108	0.931	6890	tags=55%, list=36%, signal=86%	FALSE
BIOCARTA_IL7_PATHWAY	BIOCARTA_IL7_PATHWAY		17	-0.5912611	0.003072197	-1.726229	0.021684946	0.932	4591	tags=53%, list=24%, signal=70%	FALSE
REACTOME_PHOSPHORYLATION_OF_THE_APC_C	REACTOME_PHOSPHORYLATION_OF_THE_APC_C		15	-0.6104003	0.009615385	-1.7246544	0.021852985	0.936	3871	tags=47%, list=20%, signal=59%	FALSE
REACTOME_HIV_INFECTION	REACTOME_HIV_INFECTION		183	-0.3743151	0	-1.7243507	0.0217613	0.936	6831	tags=53%, list=36%, signal=82%	FALSE
REACTOME_NEP_NS2_INTERACTS_WITH_THE_CELLULAR_EXPORT_MACHINERY	REACTOME_NEP_NS2_INTERACTS_WITH_THE_CELLULAR_EXPORT_MACHINERY		25	-0.5252429	0.011764706	-1.709979	0.025145682	0.96	7420	tags=80%, list=39%, signal=132%	FALSE
KEGG_PYRIMIDINE_METABOLISM	KEGG_PYRIMIDINE_METABOLISM		93	-0.40352324	0.001333333	-1.7094921	0.025034828	0.96	4490	tags=38%, list=24%, signal=49%	FALSE
REACTOME_INHIBITION_OF_THE_PROTEOLYTIC_ACTIVITY_OF_APC_C_REQUIRED_FOR_THE_ONSET_OF_ANAPHASE_BY_MITOTIC_SPINDLE_CHECKPOINT_COMPONENTS	REACTOME_INHIBITION_OF_THE_PROTEOLYTIC_ACTIVITY_OF_APC_C_REQUIRED_FOR_THE_ONSET_OF_ANAPHASE_BY_MITOTIC_SPINDLE_CHECKPOINT_COMPONENTS		16	-0.5962838	0.011111111	-1.7083305	0.025084881	0.961	3871	tags=56%, list=20%, signal=71%	FALSE
PID_BCR_5PATHWAY	PID_BCR_5PATHWAY		64	-0.43996072	0	-1.7028147	0.026218884	0.969	3810	tags=38%, list=20%, signal=47%	FALSE
REACTOME_MITOTIC_M_M_G1_PHASES	REACTOME_MITOTIC_M_M_G1_PHASES		161	-0.37822896	0.001191895	-1.6994056	0.026886428	0.973	5595	tags=40%, list=30%, signal=57%	FALSE
SIG_CHEMOTAXIS	SIG_CHEMOTAXIS		44	-0.46096733	0.004329004	-1.698477	0.027014686	0.975	2069	tags=30%, list=11%, signal=33%	FALSE
REACTOME_HOMOLOGOUS_RECOMBINATION_REPAIR_OF_REPLICATION_INDEPENDENT_DOUBLE_STRAND_BREAKS	REACTOME_HOMOLOGOUS_RECOMBINATION_REPAIR_OF_REPLICATION_INDEPENDENT_DOUBLE_STRAND_BREAKS		16	-0.5894746	0.00921659	-1.696996	0.0271771	0.977	4414	tags=63%, list=23%, signal=81%	FALSE
BIOCARTA_FCER1_PATHWAY	BIOCARTA_FCER1_PATHWAY		38	-0.4776544	0.001459854	-1.6959115	0.027234972	0.979	1632	tags=29%, list=9%, signal=32%	FALSE
PID_HEDGEHOG_GLI_PATHWAY	PID_HEDGEHOG_GLI_PATHWAY		48	-0.44944715	0.006963789	-1.688697	0.0290394	0.987	5881	tags=60%, list=31%, signal=87%	FALSE
REACTOME_RNA_POL_I_TRANSCRIPTION_INITIATION	REACTOME_RNA_POL_I_TRANSCRIPTION_INITIATION		21	-0.55485076	0.01875	-1.6752243	0.03311361	0.993	3075	tags=38%, list=16%, signal=45%	FALSE
REACTOME_GENERIC_TRANSCRIPTION_PATHWAY	REACTOME_GENERIC_TRANSCRIPTION_PATHWAY		320	-0.34344044	0	-1.6704705	0.03441476	0.994	5486	tags=44%, list=29%, signal=61%	FALSE
BIOCARTA_ATRBRCA_PATHWAY	BIOCARTA_ATRBRCA_PATHWAY		20	-0.5575306	0.009360374	-1.6683583	0.034830555	0.995	4414	tags=50%, list=23%, signal=65%	FALSE
REACTOME_MITOCHONDRIAL_PROTEIN_IMPORT	REACTOME_MITOCHONDRIAL_PROTEIN_IMPORT		42	-0.45394748	0.005706134	-1.6676946	0.034789454	0.996	7647	tags=74%, list=40%, signal=124%	FALSE
REACTOME_LAGGING_STRAND_SYNTHESIS	REACTOME_LAGGING_STRAND_SYNTHESIS		19	-0.5596451	0.009244992	-1.666878	0.034775883	0.996	4338	tags=53%, list=23%, signal=68%	FALSE
REACTOME_RNA_POL_II_TRANSCRIPTION_PRE_INITIATION_AND_PROMOTER_OPENING	REACTOME_RNA_POL_II_TRANSCRIPTION_PRE_INITIATION_AND_PROMOTER_OPENING		37	-0.47256085	0.005797102	-1.6667995	0.03451581	0.996	6831	tags=62%, list=36%, signal=97%	FALSE
KEGG_BASAL_TRANSCRIPTION_FACTORS	KEGG_BASAL_TRANSCRIPTION_FACTORS		31	-0.49238983	0.009063444	-1.66531	0.034695454	0.996	3267	tags=42%, list=17%, signal=51%	FALSE
REACTOME_ANTIVIRAL_MECHANISM_BY_IFN_STIMULATED_GENES	REACTOME_ANTIVIRAL_MECHANISM_BY_IFN_STIMULATED_GENES		62	-0.42077273	0.002688172	-1.6572164	0.037236713	0.996	5503	tags=48%, list=29%, signal=68%	FALSE
HALLMARK_DNA_REPAIR	HALLMARK_DNA_REPAIR		144	-0.36911497	0	-1.655301	0.03769975	0.996	4490	tags=39%, list=24%, signal=51%	FALSE
REACTOME_OXYGEN_DEPENDENT_PROLINE_HYDROXYLATION_OF_HYPOXIA_INDUCIBLE_FACTOR_ALPHA	REACTOME_OXYGEN_DEPENDENT_PROLINE_HYDROXYLATION_OF_HYPOXIA_INDUCIBLE_FACTOR_ALPHA		15	-0.59563786	0.0112	-1.6538758	0.03789076	0.996	2621	tags=47%, list=14%, signal=54%	FALSE
PID_AR_TF_PATHWAY	PID_AR_TF_PATHWAY		52	-0.43449935	0.006738544	-1.6494391	0.039446857	0.996	5674	tags=52%, list=30%, signal=74%	FALSE
BIOCARTA_ARF_PATHWAY	BIOCARTA_ARF_PATHWAY		17	-0.5576744	0.015600624	-1.6470369	0.03998756	0.997	4591	tags=59%, list=24%, signal=78%	FALSE
PID_ERB_GENOMIC_PATHWAY	PID_ERB_GENOMIC_PATHWAY		15	-0.583657	0.022617124	-1.6427146	0.041622322	0.998	5580	tags=67%, list=30%, signal=95%	FALSE
BIOCARTA_EIF_PATHWAY	BIOCARTA_EIF_PATHWAY		15	-0.5800274	0.016819572	-1.6391567	0.042692557	0.998	1739	tags=40%, list=9%, signal=44%	FALSE
PID_ATM_PATHWAY	PID_ATM_PATHWAY		33	-0.4776773	0.01969697	-1.6332444	0.04466849	0.999	4150	tags=45%, list=22%, signal=58%	FALSE
REACTOME_REGULATORY_RNA_PATHWAYS	REACTOME_REGULATORY_RNA_PATHWAYS		24	-0.50893354	0.012195122	-1.6305248	0.045373447	0.999	3406	tags=38%, list=18%, signal=46%	FALSE
BIOCARTA_CARM_ER_PATHWAY	BIOCARTA_CARM_ER_PATHWAY		34	-0.47130957	0.010279002	-1.6243876	0.047836028	0.999	4562	tags=53%, list=24%, signal=70%	FALSE
PID_CMYB_PATHWAY	PID_CMYB_PATHWAY		83	-0.39359203	0.005174644	-1.6163135	0.0509457	0.999	3301	tags=36%, list=17%, signal=44%	FALSE
REACTOME_FORMATION_OF_INCISION_COMPLEX_IN_GG_NER	REACTOME_FORMATION_OF_INCISION_COMPLEX_IN_GG_NER		17	-0.54411674	0.022508038	-1.6162341	0.050604906	0.999	5803	tags=59%, list=31%, signal=85%	FALSE
REACTOME_TRAF6_MEDIATED_IRF7_ACTIVATION	REACTOME_TRAF6_MEDIATED_IRF7_ACTIVATION		26	-0.4958586	0.011976048	-1.6143435	0.051110145	0.999	5943	tags=62%, list=31%, signal=90%	FALSE
REACTOME_HOST_INTERACTIONS_OF_HIV_FACTORS	REACTOME_HOST_INTERACTIONS_OF_HIV_FACTORS		115	-0.37130827	0.002522068	-1.606495	0.054526873	0.999	6823	tags=49%, list=36%, signal=76%	FALSE
BIOCARTA_CTLA4_PATHWAY	BIOCARTA_CTLA4_PATHWAY		19	-0.532431	0.0152207	-1.6046537	0.055108994	0.999	7475	tags=74%, list=40%, signal=122%	FALSE
REACTOME_PROCESSIVE_SYNTHESIS_ON_THE_LAGGING_STRAND	REACTOME_PROCESSIVE_SYNTHESIS_ON_THE_LAGGING_STRAND		15	-0.55906844	0.021671826	-1.6034039	0.055428915	0.999	4338	tags=53%, list=23%, signal=69%	FALSE
REACTOME_G1_S_SPECIFIC_TRANSCRIPTION	REACTOME_G1_S_SPECIFIC_TRANSCRIPTION		16	-0.5593815	0.03069467	-1.5973135	0.057922456	0.999	6821	tags=75%, list=36%, signal=117%	FALSE
SA_PTEN_PATHWAY	SA_PTEN_PATHWAY		17	-0.5479056	0.028744327	-1.5973029	0.057523165	0.999	1999	tags=35%, list=11%, signal=39%	FALSE
REACTOME_RIG_I_MDA5_MEDIATED_INDUCTION_OF_IFN_ALPHA_BETA_PATHWAYS	REACTOME_RIG_I_MDA5_MEDIATED_INDUCTION_OF_IFN_ALPHA_BETA_PATHWAYS		66	-0.40066305	0.0067659	-1.5954138	0.05814197	0.999	5943	tags=55%, list=31%, signal=79%	FALSE
BIOCARTA_BCR_PATHWAY	BIOCARTA_BCR_PATHWAY		34	-0.4617592	0.017118402	-1.5848917	0.06344437	1	1632	tags=29%, list=9%, signal=32%	FALSE
REACTOME_TCA_CYCLE_AND_RESPIRATORY_ELECTRON_TRANSPORT	REACTOME_TCA_CYCLE_AND_RESPIRATORY_ELECTRON_TRANSPORT		106	-0.37015462	0.00655308	-1.5813459	0.065030836	1	7033	tags=54%, list=37%, signal=85%	FALSE
REACTOME_ANTIGEN_PROCESSING_UBIQUITINATION_PROTEASOME_DEGRADATION	REACTOME_ANTIGEN_PROCESSING_UBIQUITINATION_PROTEASOME_DEGRADATION		190	-0.34482875	0	-1.581298	0.06460548	1	6401	tags=45%, list=34%, signal=67%	FALSE
REACTOME_ELONGATION_ARREST_AND_RECOVERY	REACTOME_ELONGATION_ARREST_AND_RECOVERY		30	-0.47144663	0.015536723	-1.5779501	0.06605395	1	7327	tags=77%, list=39%, signal=125%	FALSE
REACTOME_G1_PHASE	REACTOME_G1_PHASE		34	-0.45321733	0.016516516	-1.5736846	0.068115614	1	5547	tags=56%, list=29%, signal=79%	FALSE
KEGG_B_CELL_RECEPTOR_SIGNALING_PATHWAY	KEGG_B_CELL_RECEPTOR_SIGNALING_PATHWAY		74	-0.39214107	0.007633588	-1.5729394	0.06814361	1	2165	tags=27%, list=11%, signal=30%	FALSE
PID_TELOMERASE_PATHWAY	PID_TELOMERASE_PATHWAY		67	-0.3983901	0.010738255	-1.5726037	0.06796732	1	6491	tags=58%, list=34%, signal=88%	FALSE
REACTOME_CHROMOSOME_MAINTENANCE	REACTOME_CHROMOSOME_MAINTENANCE		101	-0.36766347	0.00511509	-1.5694656	0.06922221	1	6393	tags=55%, list=34%, signal=83%	FALSE
PID_RB_1PATHWAY	PID_RB_1PATHWAY		64	-0.40281174	0.010840109	-1.566699	0.070454285	1	5292	tags=55%, list=28%, signal=76%	FALSE
BIOCARTA_CERAMIDE_PATHWAY	BIOCARTA_CERAMIDE_PATHWAY		22	-0.5033742	0.0311042	-1.5646292	0.071144186	1	1274	tags=27%, list=7%, signal=29%	FALSE
REACTOME_FORMATION_OF_THE_HIV1_EARLY_ELONGATION_COMPLEX	REACTOME_FORMATION_OF_THE_HIV1_EARLY_ELONGATION_COMPLEX		30	-0.45956984	0.022488756	-1.5621551	0.07226906	1	7327	tags=70%, list=39%, signal=114%	FALSE
BIOCARTA_IL3_PATHWAY	BIOCARTA_IL3_PATHWAY		15	-0.53693694	0.024509804	-1.5610015	0.07246324	1	2765	tags=33%, list=15%, signal=39%	FALSE
REACTOME_BRANCHED_CHAIN_AMINO_ACID_CATABOLISM	REACTOME_BRANCHED_CHAIN_AMINO_ACID_CATABOLISM		17	-0.5308682	0.03610675	-1.5593309	0.07314245	1	5374	tags=53%, list=28%, signal=74%	FALSE
REACTOME_UNBLOCKING_OF_NMDA_RECEPTOR_GLUTAMATE_BINDING_AND_ACTIVATION	REACTOME_UNBLOCKING_OF_NMDA_RECEPTOR_GLUTAMATE_BINDING_AND_ACTIVATION		15	-0.5500115	0.0311042	-1.5532115	0.07640072	1	3997	tags=40%, list=21%, signal=51%	FALSE
BIOCARTA_PITX2_PATHWAY	BIOCARTA_PITX2_PATHWAY		15	-0.55942047	0.033707865	-1.5478779	0.07914947	1	3132	tags=53%, list=17%, signal=64%	FALSE
PID_HDAC_CLASSI_PATHWAY	PID_HDAC_CLASSI_PATHWAY		64	-0.39088887	0.011004127	-1.5466888	0.079353906	1	4973	tags=47%, list=26%, signal=63%	FALSE
REACTOME_ACTIVATION_OF_GENES_BY_ATF4	REACTOME_ACTIVATION_OF_GENES_BY_ATF4		23	-0.49662584	0.027900146	-1.5459983	0.079346135	1	4191	tags=52%, list=22%, signal=67%	FALSE
REACTOME_BASE_EXCISION_REPAIR	REACTOME_BASE_EXCISION_REPAIR		18	-0.5254964	0.032659408	-1.5447778	0.07976394	1	2103	tags=39%, list=11%, signal=44%	FALSE
REACTOME_ABORTIVE_ELONGATION_OF_HIV1_TRANSCRIPT_IN_THE_ABSENCE_OF_TAT	REACTOME_ABORTIVE_ELONGATION_OF_HIV1_TRANSCRIPT_IN_THE_ABSENCE_OF_TAT		23	-0.48375592	0.03259259	-1.5417979	0.081355385	1	3281	tags=43%, list=17%, signal=53%	FALSE
BIOCARTA_CTCF_PATHWAY	BIOCARTA_CTCF_PATHWAY		23	-0.48486453	0.031484257	-1.5412239	0.08123603	1	2368	tags=35%, list=13%, signal=40%	FALSE
BIOCARTA_NO2IL12_PATHWAY	BIOCARTA_NO2IL12_PATHWAY		17	-0.539007	0.022047244	-1.5407641	0.08106446	1	8243	tags=82%, list=44%, signal=146%	FALSE
PID_ERA_GENOMIC_PATHWAY	PID_ERA_GENOMIC_PATHWAY		64	-0.38861215	0.008130081	-1.5304966	0.0874076	1	4562	tags=44%, list=24%, signal=57%	FALSE
REACTOME_ASSEMBLY_OF_THE_PRE_REPLICATIVE_COMPLEX	REACTOME_ASSEMBLY_OF_THE_PRE_REPLICATIVE_COMPLEX		60	-0.3950092	0.015320335	-1.5300655	0.08716332	1	3144	tags=27%, list=17%, signal=32%	FALSE
PID_IL3_PATHWAY	PID_IL3_PATHWAY		27	-0.4652481	0.038011696	-1.5291508	0.087225206	1	2926	tags=41%, list=15%, signal=48%	FALSE
REACTOME_PERK_REGULATED_GENE_EXPRESSION	REACTOME_PERK_REGULATED_GENE_EXPRESSION		26	-0.47141668	0.030927835	-1.5227274	0.091437034	1	4668	tags=54%, list=25%, signal=71%	FALSE
BIOCARTA_SHH_PATHWAY	BIOCARTA_SHH_PATHWAY		16	-0.53353196	0.037936267	-1.5225618	0.09102989	1	5881	tags=50%, list=31%, signal=73%	FALSE
BIOCARTA_CSK_PATHWAY	BIOCARTA_CSK_PATHWAY		22	-0.4921644	0.032307692	-1.5208724	0.09175884	1	8335	tags=77%, list=44%, signal=138%	FALSE
REACTOME_E2F_MEDIATED_REGULATION_OF_DNA_REPLICATION	REACTOME_E2F_MEDIATED_REGULATION_OF_DNA_REPLICATION		32	-0.45571062	0.028985508	-1.520611	0.09143752	1	5698	tags=50%, list=30%, signal=71%	FALSE
BIOCARTA_G2_PATHWAY	BIOCARTA_G2_PATHWAY		22	-0.47714716	0.03405573	-1.5139244	0.09589223	1	2797	tags=36%, list=15%, signal=43%	FALSE
PID_P38_MKK3_6PATHWAY	PID_P38_MKK3_6PATHWAY		26	-0.4628247	0.03259259	-1.5123448	0.096615836	1	2539	tags=31%, list=13%, signal=35%	FALSE
PID_TXA2PATHWAY	PID_TXA2PATHWAY		56	-0.3887677	0.016877636	-1.5109199	0.09715292	1	2859	tags=32%, list=15%, signal=38%	FALSE
PID_IL2_STAT5_PATHWAY	PID_IL2_STAT5_PATHWAY		30	-0.45010608	0.034638554	-1.5079039	0.098841086	1	5392	tags=47%, list=29%, signal=65%	FALSE
PID_REG_GR_PATHWAY	PID_REG_GR_PATHWAY		80	-0.36385876	0.014666666	-1.5062361	0.09962972	1	3788	tags=34%, list=20%, signal=42%	FALSE
BIOCARTA_ERK5_PATHWAY	BIOCARTA_ERK5_PATHWAY		17	-0.5237855	0.05148206	-1.5003994	0.10364125	1	5230	tags=53%, list=28%, signal=73%	FALSE
HALLMARK_OXIDATIVE_PHOSPHORYLATION	HALLMARK_OXIDATIVE_PHOSPHORYLATION		188	-0.32798204	0.002361275	-1.498561	0.10438202	1	7053	tags=54%, list=37%, signal=86%	FALSE
KEGG_PURINE_METABOLISM	KEGG_PURINE_METABOLISM		152	-0.33202526	0.003787879	-1.4943295	0.10722567	1	4490	tags=32%, list=24%, signal=41%	FALSE
BIOCARTA_BAD_PATHWAY	BIOCARTA_BAD_PATHWAY		26	-0.45733336	0.050925925	-1.4914998	0.10893897	1	2797	tags=31%, list=15%, signal=36%	FALSE
BIOCARTA_WNT_PATHWAY	BIOCARTA_WNT_PATHWAY		26	-0.45616817	0.04477612	-1.4903914	0.109278545	1	3393	tags=46%, list=18%, signal=56%	FALSE
REACTOME_METABOLISM_OF_NUCLEOTIDES	REACTOME_METABOLISM_OF_NUCLEOTIDES		68	-0.3769255	0.021917809	-1.4892234	0.109678425	1	4102	tags=37%, list=22%, signal=47%	FALSE
REACTOME_REGULATION_OF_MRNA_STABILITY_BY_PROTEINS_THAT_BIND_AU_RICH_ELEMENTS	REACTOME_REGULATION_OF_MRNA_STABILITY_BY_PROTEINS_THAT_BIND_AU_RICH_ELEMENTS		75	-0.3696348	0.024657534	-1.487283	0.11081855	1	3824	tags=31%, list=20%, signal=38%	FALSE
REACTOME_TRANSCRIPTIONAL_ACTIVITY_OF_SMAD2_SMAD3_SMAD4_HETEROTRIMER	REACTOME_TRANSCRIPTIONAL_ACTIVITY_OF_SMAD2_SMAD3_SMAD4_HETEROTRIMER		36	-0.42455342	0.03259259	-1.4872032	0.11027008	1	6019	tags=58%, list=32%, signal=85%	FALSE
REACTOME_CIRCADIAN_CLOCK	REACTOME_CIRCADIAN_CLOCK		52	-0.39279422	0.033195022	-1.4862255	0.11053087	1	3303	tags=40%, list=17%, signal=49%	FALSE
REACTOME_RECRUITMENT_OF_MITOTIC_CENTROSOME_PROTEINS_AND_COMPLEXES	REACTOME_RECRUITMENT_OF_MITOTIC_CENTROSOME_PROTEINS_AND_COMPLEXES		62	-0.37793863	0.019858155	-1.4820304	0.11330747	1	5249	tags=45%, list=28%, signal=62%	FALSE
REACTOME_ORC1_REMOVAL_FROM_CHROMATIN	REACTOME_ORC1_REMOVAL_FROM_CHROMATIN		62	-0.37275952	0.023904383	-1.4786557	0.11542224	1	3144	tags=26%, list=17%, signal=31%	FALSE
BIOCARTA_TEL_PATHWAY	BIOCARTA_TEL_PATHWAY		17	-0.5006306	0.05414013	-1.4744588	0.11836163	1	3799	tags=53%, list=20%, signal=66%	FALSE
REACTOME_SYNTHESIS_AND_INTERCONVERSION_OF_NUCLEOTIDE_DI_AND_TRIPHOSPHATES	REACTOME_SYNTHESIS_AND_INTERCONVERSION_OF_NUCLEOTIDE_DI_AND_TRIPHOSPHATES		17	-0.49922135	0.0576	-1.472563	0.119294174	1	6821	tags=71%, list=36%, signal=110%	FALSE
SIG_PIP3_SIGNALING_IN_B_LYMPHOCYTES	SIG_PIP3_SIGNALING_IN_B_LYMPHOCYTES		36	-0.4242128	0.033236995	-1.4713556	0.1197024	1	2797	tags=31%, list=15%, signal=36%	FALSE
PID_HES_HEY_PATHWAY	PID_HES_HEY_PATHWAY		46	-0.39509463	0.040446304	-1.4650071	0.12489057	1	3212	tags=37%, list=17%, signal=44%	FALSE
BIOCARTA_VIP_PATHWAY	BIOCARTA_VIP_PATHWAY		25	-0.45227483	0.052941177	-1.4626747	0.12643799	1	5905	tags=56%, list=31%, signal=81%	FALSE
REACTOME_FACTORS_INVOLVED_IN_MEGAKARYOCYTE_DEVELOPMENT_AND_PLATELET_PRODUCTION	REACTOME_FACTORS_INVOLVED_IN_MEGAKARYOCYTE_DEVELOPMENT_AND_PLATELET_PRODUCTION		114	-0.34181273	0.018891688	-1.4621036	0.12636814	1	3706	tags=30%, list=20%, signal=37%	FALSE
BIOCARTA_MTOR_PATHWAY	BIOCARTA_MTOR_PATHWAY		21	-0.47036204	0.072981365	-1.4616407	0.1261343	1	2137	tags=24%, list=11%, signal=27%	FALSE
KEGG_CYTOSOLIC_DNA_SENSING_PATHWAY	KEGG_CYTOSOLIC_DNA_SENSING_PATHWAY		48	-0.3929314	0.02503477	-1.4566126	0.13031235	1	3810	tags=35%, list=20%, signal=44%	FALSE
REACTOME_RNA_POL_I_TRANSCRIPTION_TERMINATION	REACTOME_RNA_POL_I_TRANSCRIPTION_TERMINATION		18	-0.49169457	0.06386555	-1.45592	0.1304136	1	6042	tags=56%, list=32%, signal=82%	FALSE
REACTOME_GLUCOSE_TRANSPORT	REACTOME_GLUCOSE_TRANSPORT		37	-0.4095489	0.04918033	-1.4523734	0.13304658	1	2912	tags=30%, list=15%, signal=35%	FALSE
REACTOME_MEIOSIS	REACTOME_MEIOSIS		86	-0.3505795	0.02356021	-1.4500114	0.13465315	1	4647	tags=43%, list=25%, signal=57%	FALSE
PID_IL8_CXCR2_PATHWAY	PID_IL8_CXCR2_PATHWAY		34	-0.41150802	0.06013986	-1.4491107	0.13486224	1	1389	tags=24%, list=7%, signal=25%	FALSE
REACTOME_CREB_PHOSPHORYLATION_THROUGH_THE_ACTIVATION_OF_CAMKII	REACTOME_CREB_PHOSPHORYLATION_THROUGH_THE_ACTIVATION_OF_CAMKII		15	-0.5066057	0.06944445	-1.4473078	0.1360767	1	3997	tags=40%, list=21%, signal=51%	FALSE
REACTOME_NUCLEOTIDE_LIKE_PURINERGIC_RECEPTORS	REACTOME_NUCLEOTIDE_LIKE_PURINERGIC_RECEPTORS		15	-0.5179431	0.060150377	-1.4472958	0.13541162	1	4784	tags=47%, list=25%, signal=62%	FALSE
REACTOME_FORMATION_OF_TRANSCRIPTION_COUPLED_NER_TC_NER_REPAIR_COMPLEX	REACTOME_FORMATION_OF_TRANSCRIPTION_COUPLED_NER_TC_NER_REPAIR_COMPLEX		24	-0.458608	0.05029586	-1.4463363	0.13573332	1	7708	tags=63%, list=41%, signal=105%	FALSE
REACTOME_MITOTIC_PROMETAPHASE	REACTOME_MITOTIC_PROMETAPHASE		82	-0.34855142	0.015665796	-1.438471	0.14284644	1	4973	tags=37%, list=26%, signal=49%	FALSE
PID_RAC1_REG_PATHWAY	PID_RAC1_REG_PATHWAY		37	-0.40381017	0.05467626	-1.4369794	0.14370884	1	1865	tags=24%, list=10%, signal=27%	FALSE
PID_RETINOIC_ACID_PATHWAY	PID_RETINOIC_ACID_PATHWAY		30	-0.42066106	0.05029586	-1.4354578	0.14462821	1	3909	tags=33%, list=21%, signal=42%	FALSE
REACTOME_DAG_AND_IP3_SIGNALING	REACTOME_DAG_AND_IP3_SIGNALING		31	-0.42788166	0.05124451	-1.4345347	0.1449054	1	5230	tags=42%, list=28%, signal=58%	FALSE
PID_NFAT_3PATHWAY	PID_NFAT_3PATHWAY		52	-0.3824954	0.028208744	-1.4294745	0.14971597	1	4205	tags=33%, list=22%, signal=42%	FALSE
REACTOME_IL_RECEPTOR_SHC_SIGNALING	REACTOME_IL_RECEPTOR_SHC_SIGNALING		27	-0.4368621	0.076323986	-1.4282554	0.15040298	1	2038	tags=22%, list=11%, signal=25%	FALSE
REACTOME_PLATELET_CALCIUM_HOMEOSTASIS	REACTOME_PLATELET_CALCIUM_HOMEOSTASIS		16	-0.49211454	0.0859375	-1.4270629	0.15094319	1	2758	tags=31%, list=15%, signal=37%	FALSE
PID_IL2_PI3K_PATHWAY	PID_IL2_PI3K_PATHWAY		34	-0.41076458	0.05671642	-1.4259982	0.15136707	1	2488	tags=32%, list=13%, signal=37%	FALSE
PID_KIT_PATHWAY	PID_KIT_PATHWAY		52	-0.37335092	0.033898305	-1.4215429	0.1553463	1	2488	tags=23%, list=13%, signal=27%	FALSE
REACTOME_THE_ROLE_OF_NEF_IN_HIV1_REPLICATION_AND_DISEASE_PATHOGENESIS	REACTOME_THE_ROLE_OF_NEF_IN_HIV1_REPLICATION_AND_DISEASE_PATHOGENESIS		27	-0.42964286	0.05865922	-1.4179285	0.15881415	1	1333	tags=22%, list=7%, signal=24%	FALSE
BIOCARTA_P53_PATHWAY	BIOCARTA_P53_PATHWAY		16	-0.49622375	0.07350689	-1.4179226	0.15807202	1	3938	tags=50%, list=21%, signal=63%	FALSE
KEGG_OLFACTORY_TRANSDUCTION	KEGG_OLFACTORY_TRANSDUCTION		107	-0.3277667	0.028350515	-1.4170938	0.15817463	1	3721	tags=28%, list=20%, signal=35%	FALSE
BIOCARTA_VEGF_PATHWAY	BIOCARTA_VEGF_PATHWAY		29	-0.42643175	0.06983655	-1.4160826	0.15851839	1	4668	tags=52%, list=25%, signal=69%	FALSE
REACTOME_OLFACTORY_SIGNALING_PATHWAY	REACTOME_OLFACTORY_SIGNALING_PATHWAY		72	-0.35023075	0.047106326	-1.4140223	0.15993677	1	3721	tags=33%, list=20%, signal=41%	FALSE
BIOCARTA_IGF1MTOR_PATHWAY	BIOCARTA_IGF1MTOR_PATHWAY		20	-0.47220394	0.075038284	-1.4125662	0.16089503	1	4668	tags=45%, list=25%, signal=60%	FALSE
BIOCARTA_TCR_PATHWAY	BIOCARTA_TCR_PATHWAY		44	-0.38549453	0.046767537	-1.4109505	0.16205154	1	1673	tags=25%, list=9%, signal=27%	FALSE
BIOCARTA_HDAC_PATHWAY	BIOCARTA_HDAC_PATHWAY		28	-0.42894098	0.07589286	-1.4107262	0.16157845	1	5905	tags=50%, list=31%, signal=73%	FALSE
REACTOME_TRAF6_MEDIATED_INDUCTION_OF_NFKB_AND_MAP_KINASES_UPON_TLR7_8_OR_9_ACTIVATION	REACTOME_TRAF6_MEDIATED_INDUCTION_OF_NFKB_AND_MAP_KINASES_UPON_TLR7_8_OR_9_ACTIVATION		73	-0.3532275	0.03601108	-1.4097326	0.16209253	1	4601	tags=36%, list=24%, signal=48%	FALSE
KEGG_OXIDATIVE_PHOSPHORYLATION	KEGG_OXIDATIVE_PHOSPHORYLATION		107	-0.32895574	0.032425422	-1.4066986	0.16495822	1	6741	tags=56%, list=36%, signal=86%	FALSE
REACTOME_NOTCH1_INTRACELLULAR_DOMAIN_REGULATES_TRANSCRIPTION	REACTOME_NOTCH1_INTRACELLULAR_DOMAIN_REGULATES_TRANSCRIPTION		44	-0.38486987	0.07573633	-1.4032189	0.16799164	1	6061	tags=59%, list=32%, signal=87%	FALSE
HALLMARK_ALLOGRAFT_REJECTION	HALLMARK_ALLOGRAFT_REJECTION		198	-0.30183727	0.016625738	-1.4015697	0.16930936	1	7858	tags=59%, list=42%, signal=96%	FALSE
REACTOME_CLASS_I_MHC_MEDIATED_ANTIGEN_PROCESSING_PRESENTATION	REACTOME_CLASS_I_MHC_MEDIATED_ANTIGEN_PROCESSING_PRESENTATION		224	-0.29576516	0.008073818	-1.3943835	0.17255515	1	4200	tags=34%, list=22%, signal=41%	FALSE
REACTOME_SMAD2_SMAD3_SMAD4_HETEROTRIMER_REGULATES_TRANSCRIPTION	REACTOME_SMAD2_SMAD3_SMAD4_HETEROTRIMER_REGULATES_TRANSCRIPTION		25	-0.45047317	0.074484984	-1.3927523	0.17849624	1	6066	tags=72%, list=32%, signal=106%	FALSE
PID_FCER1_PATHWAY	PID_FCER1_PATHWAY		61	-0.35495657	0.055478502	-1.3925941	0.17791535	1	2030	tags=25%, list=11%, signal=27%	FALSE
REACTOME_NEGATIVE_REGULATORS_OF_RIG_I_MDA5_SIGNALING	REACTOME_NEGATIVE_REGULATORS_OF_RIG_I_MDA5_SIGNALING		30	-0.41657826	0.08066971	-1.3906282	0.17958656	1	5940	tags=50%, list=32%, signal=73%	FALSE
REACTOME_MICRORNA_MIRNA_BIOGENESIS	REACTOME_MICRORNA_MIRNA_BIOGENESIS		15	-0.44726056	0.08042488	-1.3899795	0.17958836	1	2952	tags=40%, list=16%, signal=48%	FALSE
PID_MYC_PATHWAY	PID_MYC_PATHWAY		25	-0.43437706	0.08592593	-1.3894129	0.17944375	1	5874	tags=56%, list=31%, signal=81%	FALSE
KEGG_TASTE_TRANSDUCTION	KEGG_TASTE_TRANSDUCTION		42	-0.38800973	0.058739256	-1.3884704	0.17972802	1	6229	tags=45%, list=33%, signal=67%	FALSE
KEGG_AMINOACYL_TRNA_BIOSYNTHESIS	KEGG_AMINOACYL_TRNA_BIOSYNTHESIS		41	-0.38851648	0.06540698	-1.3869109	0.18078065	1	6559	tags=49%, list=35%, signal=75%	FALSE
REACTOME_MITOTIC_G2_G2_M_PHASES	REACTOME_MITOTIC_G2_G2_M_PHASES		77	-0.3434109	0.059916775	-1.3853742	0.18192248	1	5698	tags=43%, list=30%, signal=61%	FALSE

REACTOME_ACTIVATED_TLR4_SIGNALLING	REACTOME_ACTIVATED_TLR4_SIGNALLING		87	-0.33345103	0.034300793	-1.3801669	0.18769296	1	3810	tags=29%, list=20%, signal=36%	FALSE
REACTOME_IL_3_5_AND_GM_CSF_SIGNALING	REACTOME_IL_3_5_AND_GM_CSF_SIGNALING		43	-0.37865052	0.081277214	-1.3786956	0.18865167	1	4591	tags=35%, list=24%, signal=46%	FALSE
REACTOME_CA_DEPENDENT_EVENTS	REACTOME_CA_DEPENDENT_EVENTS		29	-0.4129518	0.08104197	-1.3782072	0.18849611	1	5203	tags=38%, list=28%, signal=52%	FALSE
REACTOME_MYD88_MAL_CASCADE_INITIATED_ON_PLASMA_MEMBRANE	REACTOME_MYD88_MAL_CASCADE_INITIATED_ON_PLASMA_MEMBRANE		78	-0.33866933	0.04725415	-1.3777375	0.18819875	1	3810	tags=29%, list=20%, signal=37%	FALSE
PID_CDC42_REG_PATHWAY	PID_CDC42_REG_PATHWAY		30	-0.40786436	0.07121662	-1.3767518	0.18862343	1	7182	tags=57%, list=38%, signal=91%	FALSE
SIG_IL4RECEPTOR_IN_B_LYPHOCYTES	SIG_IL4RECEPTOR_IN_B_LYPHOCYTES		27	-0.41851318	0.093797274	-1.3736827	0.19186667	1	1913	tags=22%, list=10%, signal=25%	FALSE
PID_SMAD2_3NUCLEAR_PATHWAY	PID_SMAD2_3NUCLEAR_PATHWAY		81	-0.3328342	0.04915912	-1.3698047	0.19627576	1	3337	tags=35%, list=18%, signal=42%	FALSE
REACTOME_CYTOKINE_SIGNALING_IN_IMMUNE_SYSTEM	REACTOME_CYTOKINE_SIGNALING_IN_IMMUNE_SYSTEM		248	-0.28573045	0.021468926	-1.3622074	0.20558003	1	5503	tags=35%, list=29%, signal=49%	FALSE
KEGG_ALZHEIMERS_DISEASE	KEGG_ALZHEIMERS_DISEASE		148	-0.30602047	0.025990099	-1.3604865	0.20713507	1	6376	tags=45%, list=34%, signal=67%	FALSE
REACTOME_LOSS_OF_NLP_FROM_MITOTIC_CENTROSOMES	REACTOME_LOSS_OF_NLP_FROM_MITOTIC_CENTROSOMES		55	-0.35362568	0.06910569	-1.3582418	0.20946786	1	5249	tags=45%, list=28%, signal=63%	FALSE
PID_AR_PATHWAY	PID_AR_PATHWAY		58	-0.35274938	0.0728022	-1.3549224	0.21305574	1	6269	tags=59%, list=33%, signal=87%	FALSE
ST_JNK_MAPK_PATHWAY	ST_JNK_MAPK_PATHWAY		40	-0.37118334	0.08250356	-1.3499023	0.21889056	1	5370	tags=45%, list=28%, signal=63%	FALSE
PID_PI3KCI_PATHWAY	PID_PI3KCI_PATHWAY		49	-0.3640419	0.07386363	-1.3493605	0.21870881	1	2857	tags=31%, list=15%, signal=36%	FALSE
PID_TCR_PATHWAY	PID_TCR_PATHWAY		65	-0.3432379	0.06507304	-1.3481424	0.21948352	1	7068	tags=52%, list=37%, signal=83%	FALSE
KEGG_ACUTE_MYELOID_LEUKEMIA	KEGG_ACUTE_MYELOID_LEUKEMIA		56	-0.35486358	0.0651289	-1.3454945	0.22222963	1	4753	tags=36%, list=25%, signal=48%	FALSE
REACTOME_TOLL_RECEPTOR_CASCADES	REACTOME_TOLL_RECEPTOR_CASCADES		110	-0.3143517	0.05276382	-1.3431762	0.22467405	1	4613	tags=34%, list=24%, signal=44%	FALSE
REACTOME_INTERFERON_SIGNALING	REACTOME_INTERFERON_SIGNALING		140	-0.30033112	0.043636363	-1.3404932	0.22747384	1	3822	tags=28%, list=20%, signal=35%	FALSE
REACTOME_TRNA_AMINOACYLATION	REACTOME_TRNA_AMINOACYLATION		42	-0.36923313	0.084165476	-1.3392742	0.22816157	1	3830	tags=31%, list=20%, signal=39%	FALSE
REACTOME_RESOLUTION_OF_AP_SITES_VIA_THE_MULTIPLE_NUCLEOTIDE_PATCH_REPLACEMENT_PATHWAY	REACTOME_RESOLUTION_OF_AP_SITES_VIA_THE_MULTIPLE_NUCLEOTIDE_PATCH_REPLACEMENT_PATHWAY		16	-0.45874	0.11353032	-1.3373766	0.22996177	1	1802	tags=31%, list=10%, signal=35%	FALSE
REACTOME_SIGNALING_BY_FGFR1_FUSION_MUTANTS	REACTOME_SIGNALING_BY_FGFR1_FUSION_MUTANTS		17	-0.45731482	0.13607594	-1.3369632	0.22968043	1	2870	tags=35%, list=15%, signal=42%	FALSE
BIOCARTA_IL2RB_PATHWAY	BIOCARTA_IL2RB_PATHWAY		38	-0.37960035	0.09836066	-1.3362486	0.22984558	1	2765	tags=29%, list=15%, signal=34%	FALSE
KEGG_MTOR_SIGNALING_PATHWAY	KEGG_MTOR_SIGNALING_PATHWAY		49	-0.35793945	0.08450704	-1.3356634	0.22979875	1	2137	tags=18%, list=11%, signal=21%	FALSE
REACTOME_ENERGY_DEPENDENT_REGULATION_OF_MTOR_BY_LKB1_AMPK	REACTOME_ENERGY_DEPENDENT_REGULATION_OF_MTOR_BY_LKB1_AMPK		16	-0.46048182	0.121069185	-1.3353837	0.22931252	1	1664	tags=19%, list=9%, signal=21%	FALSE
BIOCARTA_LAIR_PATHWAY	BIOCARTA_LAIR_PATHWAY		16	-0.46552163	0.13050315	-1.3304088	0.23560683	1	1589	tags=25%, list=8%, signal=27%	FALSE
REACTOME_TELOMERE_MAINTENANCE	REACTOME_TELOMERE_MAINTENANCE		59	-0.34165007	0.09090909	-1.32486	0.242864	1	6569	tags=59%, list=35%, signal=91%	FALSE
PID_AURORA_B_PATHWAY	PID_AURORA_B_PATHWAY		39	-0.37029168	0.10678211	-1.3213125	0.24721749	1	6500	tags=41%, list=34%, signal=62%	FALSE
KEGG_OOCYTE_MEIOSIS	KEGG_OOCYTE_MEIOSIS		106	-0.3079737	0.06905371	-1.3151714	0.25565127	1	5203	tags=31%, list=28%, signal=43%	FALSE
REACTOME_MITOCHONDRIAL_TRNA_AMINOACYLATION	REACTOME_MITOCHONDRIAL_TRNA_AMINOACYLATION		21	-0.42813402	0.12596007	-1.3137114	0.25703165	1	7164	tags=52%, list=38%, signal=84%	FALSE
PID_EPO_PATHWAY	PID_EPO_PATHWAY		34	-0.38045672	0.12326656	-1.308299	0.26485407	1	3423	tags=29%, list=18%, signal=36%	FALSE
PID_CXCR4_PATHWAY	PID_CXCR4_PATHWAY		99	-0.30866054	0.07105943	-1.3078206	0.26468998	1	2859	tags=26%, list=15%, signal=31%	FALSE
KEGG_HEMATOPOIETIC_CELL_LINEAGE	KEGG_HEMATOPOIETIC_CELL_LINEAGE		80	-0.31822926	0.086842105	-1.3068007	0.26535589	1	4574	tags=33%, list=24%, signal=43%	FALSE
REACTOME_DOWNREGULATION_OF_SMAD2_3_SMAD4_TRANSCRIPTIONAL_ACTIVITY	REACTOME_DOWNREGULATION_OF_SMAD2_3_SMAD4_TRANSCRIPTIONAL_ACTIVITY		19	-0.44596064	0.12698413	-1.3050313	0.26720774	1	4853	tags=47%, list=26%, signal=64%	FALSE
KEGG_LYSINE_DEGRADATION	KEGG_LYSINE_DEGRADATION		43	-0.3671009	0.1270073	-1.2992774	0.27548316	1	5211	tags=44%, list=28%, signal=61%	FALSE
REACTOME_DOWNSTREAM_TCR_SIGNALING	REACTOME_DOWNSTREAM_TCR_SIGNALING		30	-0.38312915	0.12099125	-1.2929927	0.28499815	1	7475	tags=60%, list=40%, signal=99%	FALSE
BIOCARTA_TNFR1_PATHWAY	BIOCARTA_TNFR1_PATHWAY		29	-0.3927674	0.12462908	-1.2926724	0.28446987	1	4205	tags=45%, list=22%, signal=58%	FALSE
KEGG_CYSTEINE_AND_METHIONINE_METABOLISM	KEGG_CYSTEINE_AND_METHIONINE_METABOLISM		31	-0.37549874	0.14179105	-1.2881504	0.29075524	1	5339	tags=42%, list=28%, signal=58%	FALSE
PID_DNA_PK_PATHWAY	PID_DNA_PK_PATHWAY		15	-0.46379825	0.15590551	-1.2878157	0.29027605	1	3393	tags=33%, list=18%, signal=41%	FALSE
PID_LKB1_PATHWAY	PID_LKB1_PATHWAY		45	-0.35450044	0.11500701	-1.2840633	0.29527795	1	2688	tags=24%, list=14%, signal=28%	FALSE
REACTOME_MEIOTIC_RECOMBINATION	REACTOME_MEIOTIC_RECOMBINATION		58	-0.33475193	0.14027777	-1.2800211	0.30139795	1	4647	tags=43%, list=25%, signal=57%	FALSE
BIOCARTA_MAPK_PATHWAY	BIOCARTA_MAPK_PATHWAY		86	-0.31162733	0.114583336	-1.2799242	0.3004894	1	4753	tags=37%, list=25%, signal=49%	FALSE
REACTOME_RNA_POL_I_TRANSCRIPTION	REACTOME_RNA_POL_I_TRANSCRIPTION		58	-0.3296082	0.11126006	-1.277439	0.30349627	1	6207	tags=50%, list=33%, signal=74%	FALSE
REACTOME_TRANSCRIPTIONAL_REGULATION_OF_WHITE_ADIPOCYTE_DIFFERENTIATION	REACTOME_TRANSCRIPTIONAL_REGULATION_OF_WHITE_ADIPOCYTE_DIFFERENTIATION		72	-0.3159578	0.12242091	-1.2746766	0.30717364	1	3970	tags=35%, list=21%, signal=44%	FALSE
KEGG_PROGESTERONE_MEDIATED_OOCYTE_MATURATION	KEGG_PROGESTERONE_MEDIATED_OOCYTE_MATURATION		81	-0.31198764	0.101876676	-1.2731614	0.30879676	1	5421	tags=35%, list=29%, signal=48%	FALSE
KEGG_NATURAL_KILLER_CELL_MEDIATED_CYTOTOXICITY	KEGG_NATURAL_KILLER_CELL_MEDIATED_CYTOTOXICITY		121	-0.2913788	0.09308176	-1.269773	0.3138267	1	5613	tags=38%, list=30%, signal=54%	FALSE
REACTOME_PURINE_METABOLISM	REACTOME_PURINE_METABOLISM		31	-0.37269837	0.14630468	-1.2670794	0.31766987	1	3867	tags=35%, list=20%, signal=45%	FALSE
REACTOME_CIRCADIAN_REPRESSION_OF_EXPRESSION_BY_REV_ERBA	REACTOME_CIRCADIAN_REPRESSION_OF_EXPRESSION_BY_REV_ERBA		23	-0.40083018	0.16589147	-1.2639375	0.32216224	1	3303	tags=43%, list=17%, signal=53%	FALSE
REACTOME_CYTOSOLIC_TRNA_AMINOACYLATION	REACTOME_CYTOSOLIC_TRNA_AMINOACYLATION		24	-0.39111495	0.15430267	-1.262807	0.32289046	1	2757	tags=29%, list=15%, signal=34%	FALSE
REACTOME_IL_2_SIGNALING	REACTOME_IL_2_SIGNALING		41	-0.3550521	0.15044248	-1.2627976	0.32175356	1	2765	tags=22%, list=15%, signal=26%	FALSE
ST_T_CELL_SIGNAL_TRANSDUCTION	ST_T_CELL_SIGNAL_TRANSDUCTION		45	-0.34420836	0.15064563	-1.2576776	0.33016905	1	2195	tags=29%, list=12%, signal=33%	FALSE
KEGG_FC_EPSILON_RI_SIGNALING_PATHWAY	KEGG_FC_EPSILON_RI_SIGNALING_PATHWAY		75	-0.31003156	0.12938005	-1.2574372	0.3294827	1	4826	tags=35%, list=26%, signal=46%	FALSE
KEGG_PENTOSE_PHOSPHATE_PATHWAY	KEGG_PENTOSE_PHOSPHATE_PATHWAY		26	-0.3860957	0.15499255	-1.2562749	0.33056328	1	5570	tags=38%, list=29%, signal=54%	FALSE
BIOCARTA_P38MAPK_PATHWAY	BIOCARTA_P38MAPK_PATHWAY		37	-0.35351762	0.15532544	-1.2542847	0.33307526	1	4104	tags=38%, list=22%, signal=48%	FALSE
REACTOME_PI3K_CASCADE	REACTOME_PI3K_CASCADE		66	-0.3177066	0.108815424	-1.2533112	0.33365044	1	2672	tags=18%, list=14%, signal=21%	FALSE
KEGG_NON_SMALL_CELL_LUNG_CANCER	KEGG_NON_SMALL_CELL_LUNG_CANCER		54	-0.32865202	0.13788301	-1.2526649	0.3336663	1	5547	tags=43%, list=29%, signal=60%	FALSE
PID_IL8_CXCR1_PATHWAY	PID_IL8_CXCR1_PATHWAY		28	-0.37955672	0.14454277	-1.2518873	0.33409396	1	2859	tags=32%, list=15%, signal=38%	FALSE
REACTOME_SIGNALING_BY_THE_B_CELL_RECEPTOR_BCR	REACTOME_SIGNALING_BY_THE_B_CELL_RECEPTOR_BCR		117	-0.2898247	0.097964376	-1.2488695	0.33878487	1	5483	tags=32%, list=29%, signal=44%	FALSE
BIOCARTA_IGF1R_PATHWAY	BIOCARTA_IGF1R_PATHWAY		23	-0.39441037	0.18238021	-1.2477555	0.3397897	1	4702	tags=30%, list=25%, signal=40%	FALSE
PID_HDAC_CLASSIII_PATHWAY	PID_HDAC_CLASSIII_PATHWAY		24	-0.38444194	0.17612809	-1.2447687	0.3441833	1	4562	tags=38%, list=24%, signal=49%	FALSE
REACTOME_INNATE_IMMUNE_SYSTEM	REACTOME_INNATE_IMMUNE_SYSTEM		229	-0.26141968	0.069555305	-1.2396357	0.3527644	1	6000	tags=41%, list=32%, signal=59%	FALSE
BIOCARTA_RAC1_PATHWAY	BIOCARTA_RAC1_PATHWAY		23	-0.3923249	0.17584097	-1.2390478	0.3527333	1	2137	tags=26%, list=11%, signal=29%	FALSE
KEGG_AMYOTROPHIC_LATERAL_SCLEROSIS_ALS	KEGG_AMYOTROPHIC_LATERAL_SCLEROSIS_ALS		51	-0.3299311	0.14425771	-1.2339041	0.36163297	1	5785	tags=45%, list=31%, signal=65%	FALSE
ST_B_CELL_ANTIGEN_RECEPTOR	ST_B_CELL_ANTIGEN_RECEPTOR		40	-0.3466368	0.17226891	-1.233734	0.36070684	1	2195	tags=25%, list=12%, signal=28%	FALSE
BIOCARTA_GH_PATHWAY	BIOCARTA_GH_PATHWAY		28	-0.3772671	0.1653905	-1.2331406	0.36061233	1	5472	tags=39%, list=29%, signal=55%	FALSE
BIOCARTA_HIVNEF_PATHWAY	BIOCARTA_HIVNEF_PATHWAY		56	-0.31773627	0.14824797	-1.2315992	0.3624147	1	4205	tags=36%, list=22%, signal=46%	FALSE
BIOCARTA_RELA_PATHWAY	BIOCARTA_RELA_PATHWAY		15	-0.43409896	0.19554849	-1.2315178	0.36138728	1	3810	tags=47%, list=20%, signal=58%	FALSE
REACTOME_APC_C_CDC20_MEDIATED_DEGRADATION_OF_MITOTIC_PROTEINS	REACTOME_APC_C_CDC20_MEDIATED_DEGRADATION_OF_MITOTIC_PROTEINS		60	-0.3155419	0.1536232	-1.2235597	0.37597725	1	3871	tags=25%, list=20%, signal=31%	FALSE
REACTOME_REGULATION_OF_MITOTIC_CELL_CYCLE	REACTOME_REGULATION_OF_MITOTIC_CELL_CYCLE		72	-0.30668163	0.14870396	-1.2223734	0.3772919	1	5698	tags=35%, list=30%, signal=50%	FALSE
PID_HIF2PATHWAY	PID_HIF2PATHWAY		32	-0.35690847	0.1908284	-1.2194489	0.38203153	1	2456	tags=28%, list=13%, signal=32%	FALSE
PID_CONE_PATHWAY	PID_CONE_PATHWAY		21	-0.39416462	0.19273302	-1.2127143	0.39469546	1	2606	tags=24%, list=14%, signal=28%	FALSE
REACTOME_GLUCAGON_SIGNALING_IN_METABOLIC_REGULATION	REACTOME_GLUCAGON_SIGNALING_IN_METABOLIC_REGULATION		32	-0.35489377	0.21153846	-1.2109663	0.39692357	1	5510	tags=38%, list=29%, signal=53%	FALSE
REACTOME_PRE_NOTCH_EXPRESSION_AND_PROCESSING	REACTOME_PRE_NOTCH_EXPRESSION_AND_PROCESSING		42	-0.32745335	0.18053597	-1.2056326	0.40688083	1	5874	tags=48%, list=31%, signal=69%	FALSE
PID_SMAD2_3PATHWAY	PID_SMAD2_3PATHWAY		17	-0.40999871	0.22966507	-1.2039291	0.40911505	1	6269	tags=53%, list=33%, signal=79%	FALSE
PID_GMCSF_PATHWAY	PID_GMCSF_PATHWAY		37	-0.347771	0.20857988	-1.2018658	0.4121668	1	4591	tags=35%, list=24%, signal=46%	FALSE
PID_PS1_PATHWAY	PID_PS1_PATHWAY		46	-0.32231706	0.19498608	-1.2017748	0.41104034	1	3393	tags=33%, list=18%, signal=40%	FALSE
BIOCARTA_G1_PATHWAY	BIOCARTA_G1_PATHWAY		27	-0.36715	0.21156773	-1.2013835	0.4104973	1	5698	tags=44%, list=30%, signal=64%	FALSE
REACTOME_SIGNALING_BY_ILS	REACTOME_SIGNALING_BY_ILS		104	-0.2793139	0.16645649	-1.1970339	0.41851258	1	3837	tags=26%, list=20%, signal=32%	FALSE
PID_HDAC_CLASSII_PATHWAY	PID_HDAC_CLASSII_PATHWAY		33	-0.35044086	0.24304539	-1.1967341	0.41776326	1	6135	tags=52%, list=32%, signal=76%	FALSE
PID_FOXM1_PATHWAY	PID_FOXM1_PATHWAY		38	-0.33809295	0.20199147	-1.1965082	0.41694796	1	5159	tags=39%, list=27%, signal=54%	FALSE
REACTOME_G_BETA_GAMMA_SIGNALLING_THROUGH_PI3KGAMMA	REACTOME_G_BETA_GAMMA_SIGNALLING_THROUGH_PI3KGAMMA		24	-0.38106868	0.22561863	-1.1961771	0.41636783	1	1627	tags=21%, list=9%, signal=23%	FALSE
REACTOME_G_PROTEIN_BETA_GAMMA_SIGNALLING	REACTOME_G_PROTEIN_BETA_GAMMA_SIGNALLING		27	-0.3673187	0.24054463	-1.195476	0.41655535	1	2857	tags=26%, list=15%, signal=31%	FALSE
REACTOME_PRE_NOTCH_TRANSCRIPTION_AND_TRANSLATION	REACTOME_PRE_NOTCH_TRANSCRIPTION_AND_TRANSLATION		27	-0.36031976	0.22122571	-1.1948687	0.41652665	1	5874	tags=56%, list=31%, signal=81%	FALSE
KEGG_COLORECTAL_CANCER	KEGG_COLORECTAL_CANCER		62	-0.30224526	0.18741633	-1.1943308	0.41645864	1	5142	tags=34%, list=27%, signal=46%	FALSE
REACTOME_TRIF_MEDIATED_TLR3_SIGNALING	REACTOME_TRIF_MEDIATED_TLR3_SIGNALING		70	-0.29690686	0.164	-1.1927525	0.41866776	1	4603	tags=31%, list=24%, signal=41%	FALSE
PID_MYC_REPRESS_PATHWAY	PID_MYC_REPRESS_PATHWAY		60	-0.30956858	0.18424754	-1.1912442	0.4204033	1	3263	tags=32%, list=17%, signal=38%	FALSE
BIOCARTA_PGC1A_PATHWAY	BIOCARTA_PGC1A_PATHWAY		22	-0.38240725	0.22421524	-1.1877888	0.42639142	1	5905	tags=36%, list=31%, signal=53%	FALSE
BIOCARTA_ETS_PATHWAY	BIOCARTA_ETS_PATHWAY		18	-0.39883816	0.25791138	-1.1795689	0.4431892	1	3027	tags=44%, list=16%, signal=53%	FALSE
KEGG_TYPE_II_DIABETES_MELLITUS	KEGG_TYPE_II_DIABETES_MELLITUS		46	-0.31878823	0.21022727	-1.1751395	0.45161504	1	5472	tags=33%, list=29%, signal=46%	FALSE
BIOCARTA_CREB_PATHWAY	BIOCARTA_CREB_PATHWAY		27	-0.35838994	0.23845008	-1.1727557	0.45568305	1	4702	tags=30%, list=25%, signal=39%	FALSE
BIOCARTA_NKT_PATHWAY	BIOCARTA_NKT_PATHWAY		28	-0.35226223	0.24477611	-1.1727258	0.45433417	1	7858	tags=64%, list=42%, signal=110%	FALSE
PID_MTOR_4PATHWAY	PID_MTOR_4PATHWAY		66	-0.2962807	0.22161423	-1.1706179	0.45745996	1	3810	tags=24%, list=20%, signal=30%	FALSE
KEGG_ASTHMA	KEGG_ASTHMA		26	-0.3616649	0.24585219	-1.1692369	0.45921224	1	4826	tags=38%, list=26%, signal=52%	FALSE
REACTOME_SIGNALING_BY_NOTCH	REACTOME_SIGNALING_BY_NOTCH		100	-0.28160664	0.2232258	-1.1681558	0.46031475	1	3507	tags=28%, list=19%, signal=34%	FALSE
PID_HIV_NEF_PATHWAY	PID_HIV_NEF_PATHWAY		35	-0.33593506	0.23959827	-1.1676913	0.4599275	1	5928	tags=51%, list=31%, signal=75%	FALSE
ST_P38_MAPK_PATHWAY	ST_P38_MAPK_PATHWAY		36	-0.32965767	0.24017467	-1.1675899	0.45877516	1	5370	tags=39%, list=28%, signal=54%	FALSE
BIOCARTA_CELLCYCLE_PATHWAY	BIOCARTA_CELLCYCLE_PATHWAY		23	-0.37330848	0.25714287	-1.1663564	0.460155	1	5698	tags=43%, list=30%, signal=62%	FALSE
REACTOME_PYRIMIDINE_METABOLISM	REACTOME_PYRIMIDINE_METABOLISM		24	-0.36329907	0.25075528	-1.1651275	0.46156383	1	4102	tags=38%, list=22%, signal=48%	FALSE
REACTOME_ACTIVATION_OF_BH3_ONLY_PROTEINS	REACTOME_ACTIVATION_OF_BH3_ONLY_PROTEINS		16	-0.41158634	0.27126807	-1.1626445	0.46571687	1	2071	tags=31%, list=11%, signal=35%	FALSE
REACTOME_SIGNALING_BY_TGF_BETA_RECEPTOR_COMPLEX	REACTOME_SIGNALING_BY_TGF_BETA_RECEPTOR_COMPLEX		60	-0.2993666	0.22359397	-1.160954	0.46825236	1	3413	tags=30%, list=18%, signal=36%	FALSE
REACTOME_JNK_C_JUN_KINASES_PHOSPHORYLATION_AND_ACTIVATION_MEDIATED_BY_ACTIVATED_HUMAN_TAK1	REACTOME_JNK_C_JUN_KINASES_PHOSPHORYLATION_AND_ACTIVATION_MEDIATED_BY_ACTIVATED_HUMAN_TAK1		15	-0.40982053	0.27035332	-1.160803	0.4672276	1	7318	tags=60%, list=39%, signal=98%	FALSE
PID_NFKAPPAB_ATYPICAL_PATHWAY	PID_NFKAPPAB_ATYPICAL_PATHWAY		17	-0.40472886	0.26340693	-1.1596471	0.46844915	1	4591	tags=41%, list=24%, signal=54%	FALSE
BIOCARTA_TGFB_PATHWAY	BIOCARTA_TGFB_PATHWAY		19	-0.3840921	0.25267994	-1.1581987	0.47029185	1	2969	tags=32%, list=16%, signal=37%	FALSE
KEGG_REGULATION_OF_AUTOPHAGY	KEGG_REGULATION_OF_AUTOPHAGY		29	-0.35144833	0.2590909	-1.1545972	0.4773361	1	6555	tags=48%, list=35%, signal=74%	FALSE
ST_ERK1_ERK2_MAPK_PATHWAY	ST_ERK1_ERK2_MAPK_PATHWAY		32	-0.33531538	0.26462397	-1.1528509	0.4799213	1	957	tags=16%, list=5%, signal=16%	FALSE
REACTOME_ADAPTIVE_IMMUNE_SYSTEM	REACTOME_ADAPTIVE_IMMUNE_SYSTEM		488	-0.23070903	0.1178344	-1.1525306	0.4792759	1	6882	tags=41%, list=36%, signal=63%	FALSE
REACTOME_MYOGENESIS	REACTOME_MYOGENESIS		27	-0.3539769	0.28230336	-1.1490275	0.4857625	1	5073	tags=41%, list=27%, signal=56%	FALSE
REACTOME_RORA_ACTIVATES_CIRCADIAN_EXPRESSION	REACTOME_RORA_ACTIVATES_CIRCADIAN_EXPRESSION		24	-0.36318448	0.27943486	-1.1473303	0.48819968	1	3303	tags=38%, list=17%, signal=45%	FALSE
REACTOME_METABOLISM_OF_PROTEINS	REACTOME_METABOLISM_OF_PROTEINS		352	-0.23679043	0.15	-1.1440943	0.4940536	1	3999	tags=25%, list=21%, signal=31%	FALSE
KEGG_CHEMOKINE_SIGNALING_PATHWAY	KEGG_CHEMOKINE_SIGNALING_PATHWAY		176	-0.24939147	0.18585132	-1.1415501	0.49870348	1	3019	tags=22%, list=16%, signal=25%	FALSE
BIOCARTA_TID_PATHWAY	BIOCARTA_TID_PATHWAY		18	-0.3833196	0.29438543	-1.138145	0.5052931	1	4556	tags=44%, list=24%, signal=59%	FALSE
KEGG_TOLL_LIKE_RECEPTOR_SIGNALING_PATHWAY	KEGG_TOLL_LIKE_RECEPTOR_SIGNALING_PATHWAY		94	-0.27585933	0.25064936	-1.1359447	0.50904036	1	5975	tags=41%, list=32%, signal=60%	FALSE
BIOCARTA_FMLP_PATHWAY	BIOCARTA_FMLP_PATHWAY		35	-0.32493922	0.27482015	-1.1312999	0.51855356	1	1632	tags=17%, list=9%, signal=19%	FALSE
REACTOME_BETA_DEFENSINS	REACTOME_BETA_DEFENSINS		15	-0.4013657	0.2965204	-1.1311172	0.5175235	1	7481	tags=60%, list=40%, signal=99%	FALSE
REACTOME_GPVI_MEDIATED_ACTIVATION_CASCADE	REACTOME_GPVI_MEDIATED_ACTIVATION_CASCADE		31	-0.3347369	0.28096676	-1.1308389	0.51671463	1	6756	tags=58%, list=36%, signal=90%	FALSE
KEGG_APOPTOSIS	KEGG_APOPTOSIS		86	-0.2710975	0.25562915	-1.13012	0.51690674	1	5941	tags=43%, list=31%, signal=62%	FALSE
REACTOME_PROTEIN_FOLDING	REACTOME_PROTEIN_FOLDING		48	-0.3032063	0.26988637	-1.1292254	0.51749057	1	4876	tags=31%, list=26%, signal=42%	FALSE
PID_P38_MK2_PATHWAY	PID_P38_MK2_PATHWAY		19	-0.37158355	0.28244275	-1.1282517	0.51834816	1	2444	tags=26%, list=13%, signal=30%	FALSE
KEGG_RIG_I_LIKE_RECEPTOR_SIGNALING_PATHWAY	KEGG_RIG_I_LIKE_RECEPTOR_SIGNALING_PATHWAY		65	-0.28626472	0.25989446	-1.1254933	0.5234624	1	5975	tags=42%, list=32%, signal=61%	FALSE
HALLMARK_UNFOLDED_PROTEIN_RESPONSE	HALLMARK_UNFOLDED_PROTEIN_RESPONSE		109	-0.26196435	0.26623377	-1.1247431	0.5236644	1	5746	tags=41%, list=30%, signal=59%	FALSE
REACTOME_INTRINSIC_PATHWAY_FOR_APOPTOSIS	REACTOME_INTRINSIC_PATHWAY_FOR_APOPTOSIS		28	-0.3368648	0.29239765	-1.1246403	0.52242297	1	6719	tags=61%, list=36%, signal=94%	FALSE
REACTOME_POST_NMDA_RECEPTOR_ACTIVATION_EVENTS	REACTOME_POST_NMDA_RECEPTOR_ACTIVATION_EVENTS		32	-0.3295694	0.3045977	-1.124216	0.52192426	1	4957	tags=34%, list=26%, signal=47%	FALSE
KEGG_FC_GAMMA_R_MEDIATED_PHAGOCYTOSIS	KEGG_FC_GAMMA_R_MEDIATED_PHAGOCYTOSIS		87	-0.2716431	0.25419354	-1.1237553	0.52153045	1	4591	tags=32%, list=24%, signal=42%	FALSE
REACTOME_TRAF6_MEDIATED_NFKB_ACTIVATION	REACTOME_TRAF6_MEDIATED_NFKB_ACTIVATION		19	-0.36815244	0.30838323	-1.1220944	0.52385575	1	5943	tags=53%, list=31%, signal=77%	FALSE
BIOCARTA_P53HYPOXIA_PATHWAY	BIOCARTA_P53HYPOXIA_PATHWAY		22	-0.36629814	0.30267063	-1.1199201	0.5274616	1	2456	tags=32%, list=13%, signal=37%	FALSE
REACTOME_SYNTHESIS_OF_PIPS_AT_THE_GOLGI_MEMBRANE	REACTOME_SYNTHESIS_OF_PIPS_AT_THE_GOLGI_MEMBRANE		16	-0.3951625	0.33898306	-1.1168259	0.5331907	1	1145	tags=19%, list=6%, signal=20%	FALSE
PID_CIRCADIAN_PATHWAY	PID_CIRCADIAN_PATHWAY		16	-0.3966023	0.3129139	-1.1164666	0.53255713	1	1364	tags=25%, list=7%, signal=27%	FALSE
KEGG_LONG_TERM_POTENTIATION	KEGG_LONG_TERM_POTENTIATION		67	-0.27994075	0.29598895	-1.1158093	0.5326763	1	2797	tags=19%, list=15%, signal=23%	FALSE
REACTOME_PI_METABOLISM	REACTOME_PI_METABOLISM		46	-0.2998299	0.3034483	-1.1085634	0.54846376	1	2257	tags=20%, list=12%, signal=22%	FALSE
REACTOME_DEFENSINS	REACTOME_DEFENSINS		20	-0.36647025	0.316847	-1.1080496	0.54816264	1	7481	tags=60%, list=40%, signal=99%	FALSE
REACTOME_ACTIVATION_OF_NMDA_RECEPTOR_UPON_GLUTAMATE_BINDING_AND_POSTSYNAPTIC_EVENTS	REACTOME_ACTIVATION_OF_NMDA_RECEPTOR_UPON_GLUTAMATE_BINDING_AND_POSTSYNAPTIC_EVENTS		36	-0.317905	0.32225433	-1.1072572	0.54855806	1	4957	tags=33%, list=26%, signal=45%	FALSE
KEGG_T_CELL_RECEPTOR_SIGNALING_PATHWAY	KEGG_T_CELL_RECEPTOR_SIGNALING_PATHWAY		107	-0.25915584	0.30379745	-1.1052474	0.55169874	1	5409	tags=36%, list=29%, signal=49%	FALSE
ST_INTERLEUKIN_4_PATHWAY	ST_INTERLEUKIN_4_PATHWAY		26	-0.34032178	0.32973805	-1.1050713	0.55059385	1	2137	tags=19%, list=11%, signal=22%	FALSE
KEGG_INSULIN_SIGNALING_PATHWAY	KEGG_INSULIN_SIGNALING_PATHWAY		136	-0.24814264	0.2763819	-1.1016822	0.5572064	1	5472	tags=29%, list=29%, signal=41%	FALSE
KEGG_GLYCINE_SERINE_AND_THREONINE_METABOLISM	KEGG_GLYCINE_SERINE_AND_THREONINE_METABOLISM		31	-0.3268264	0.31009957	-1.101456	0.5562735	1	6621	tags=52%, list=35%, signal=79%	FALSE
PID_NFKAPPAB_CANONICAL_PATHWAY	PID_NFKAPPAB_CANONICAL_PATHWAY		21	-0.35943434	0.32735425	-1.0985566	0.5618911	1	3810	tags=38%, list=20%, signal=48%	FALSE
REACTOME_CREB_PHOSPHORYLATION_THROUGH_THE_ACTIVATION_OF_RAS	REACTOME_CREB_PHOSPHORYLATION_THROUGH_THE_ACTIVATION_OF_RAS		26	-0.33988956	0.33781764	-1.0971922	0.56365407	1	3997	tags=31%, list=21%, signal=39%	FALSE
BIOCARTA_FAS_PATHWAY	BIOCARTA_FAS_PATHWAY		30	-0.32277882	0.35874438	-1.0953716	0.5665063	1	2990	tags=30%, list=16%, signal=36%	FALSE
REACTOME_RNA_POL_I_PROMOTER_OPENING	REACTOME_RNA_POL_I_PROMOTER_OPENING		36	-0.30915084	0.3216885	-1.0953592	0.56499803	1	4647	tags=42%, list=25%, signal=55%	FALSE
BIOCARTA_CALCINEURIN_PATHWAY	BIOCARTA_CALCINEURIN_PATHWAY		17	-0.37990832	0.36833856	-1.0953032	0.56361663	1	1632	tags=24%, list=9%, signal=26%	FALSE
REACTOME_SIGNALING_BY_NOTCH1	REACTOME_SIGNALING_BY_NOTCH1		68	-0.2781037	0.29324323	-1.0951992	0.5623437	1	6061	tags=46%, list=32%, signal=67%	FALSE
PID_P73PATHWAY	PID_P73PATHWAY		78	-0.2701282	0.3224543	-1.094557	0.56246495	1	5292	tags=36%, list=28%, signal=50%	FALSE
BIOCARTA_NOS1_PATHWAY	BIOCARTA_NOS1_PATHWAY		21	-0.35277778	0.33736762	-1.0943092	0.56155354	1	5905	tags=43%, list=31%, signal=62%	FALSE
PID_TOLL_ENDOGENOUS_PATHWAY	PID_TOLL_ENDOGENOUS_PATHWAY		21	-0.35351953	0.35276073	-1.0936936	0.5615944	1	4603	tags=33%, list=24%, signal=44%	FALSE
REACTOME_PLC_BETA_MEDIATED_EVENTS	REACTOME_PLC_BETA_MEDIATED_EVENTS		42	-0.30488572	0.3231884	-1.0920656	0.56397176	1	5905	tags=36%, list=31%, signal=52%	FALSE
REACTOME_AUTODEGRADATION_OF_CDH1_BY_CDH1_APC_C	REACTOME_AUTODEGRADATION_OF_CDH1_BY_CDH1_APC_C		51	-0.290723	0.32409972	-1.0915747	0.5638224	1	3871	tags=24%, list=20%, signal=30%	FALSE
BIOCARTA_NFAT_PATHWAY	BIOCARTA_NFAT_PATHWAY		53	-0.28608388	0.32344633	-1.0909438	0.56379473	1	5905	tags=34%, list=31%, signal=49%	FALSE
PID_CD8_TCR_PATHWAY	PID_CD8_TCR_PATHWAY		52	-0.29161376	0.30278885	-1.0907936	0.56266826	1	7856	tags=58%, list=42%, signal=98%	FALSE
KEGG_NEUROTROPHIN_SIGNALING_PATHWAY	KEGG_NEUROTROPHIN_SIGNALING_PATHWAY		122	-0.25203776	0.28607276	-1.0907179	0.56137973	1	5409	tags=33%, list=29%, signal=46%	FALSE
REACTOME_SIGNALING_BY_EGFR_IN_CANCER	REACTOME_SIGNALING_BY_EGFR_IN_CANCER		105	-0.25190645	0.3026152	-1.0850701	0.57303685	1	5409	tags=30%, list=29%, signal=41%	FALSE
REACTOME_NFKB_AND_MAP_KINASES_ACTIVATION_MEDIATED_BY_TLR4_SIGNALING_REPERTOIRE	REACTOME_NFKB_AND_MAP_KINASES_ACTIVATION_MEDIATED_BY_TLR4_SIGNALING_REPERTOIRE		67	-0.2730985	0.3237705	-1.0812117	0.5808468	1	4603	tags=30%, list=24%, signal=39%	FALSE
REACTOME_SIGNALING_BY_RHO_GTPASES	REACTOME_SIGNALING_BY_RHO_GTPASES		105	-0.25027788	0.3268734	-1.0751805	0.59398794	1	2052	tags=18%, list=11%, signal=20%	FALSE
PID_TNF_PATHWAY	PID_TNF_PATHWAY		44	-0.2910716	0.33094555	-1.0721556	0.5999693	1	1359	tags=18%, list=7%, signal=20%	FALSE
REACTOME_INFLAMMASOMES	REACTOME_INFLAMMASOMES		16	-0.37037432	0.371875	-1.0681231	0.6083576	1	2212	tags=31%, list=12%, signal=35%	FALSE
REACTOME_SIGNALING_BY_FGFR1_MUTANTS	REACTOME_SIGNALING_BY_FGFR1_MUTANTS		28	-0.3212858	0.3690476	-1.0657973	0.61263657	1	6056	tags=39%, list=32%, signal=58%	FALSE
KEGG_RENIN_ANGIOTENSIN_SYSTEM	KEGG_RENIN_ANGIOTENSIN_SYSTEM		17	-0.36482176	0.38403615	-1.0643202	0.614823	1	2980	tags=35%, list=16%, signal=42%	FALSE
KEGG_ONE_CARBON_POOL_BY_FOLATE	KEGG_ONE_CARBON_POOL_BY_FOLATE		15	-0.3694773	0.38267717	-1.0609828	0.62124836	1	6621	tags=53%, list=35%, signal=82%	FALSE
KEGG_CHRONIC_MYELOID_LEUKEMIA	KEGG_CHRONIC_MYELOID_LEUKEMIA		72	-0.2649052	0.35270807	-1.0608442	0.6200351	1	3337	tags=25%, list=18%, signal=30%	FALSE
PID_P53_DOWNSTREAM_PATHWAY	PID_P53_DOWNSTREAM_PATHWAY		133	-0.23978075	0.35507247	-1.0595671	0.6215976	1	2990	tags=23%, list=16%, signal=27%	FALSE
PID_AMB2_NEUTROPHILS_PATHWAY	PID_AMB2_NEUTROPHILS_PATHWAY		40	-0.29492518	0.3779972	-1.0593001	0.6207162	1	1589	tags=20%, list=8%, signal=22%	FALSE
ST_ADRENERGIC	ST_ADRENERGIC		34	-0.305885	0.37755102	-1.0592242	0.61928815	1	1913	tags=21%, list=10%, signal=23%	FALSE
REACTOME_NUCLEOTIDE_BINDING_DOMAIN_LEUCINE_RICH_REPEAT_CONTAINING_RECEPTOR_NLR_SIGNALING_PATHWAYS	REACTOME_NUCLEOTIDE_BINDING_DOMAIN_LEUCINE_RICH_REPEAT_CONTAINING_RECEPTOR_NLR_SIGNALING_PATHWAYS		43	-0.2928011	0.36585367	-1.0589991	0.61829615	1	5421	tags=44%, list=29%, signal=62%	FALSE
KEGG_SYSTEMIC_LUPUS_ERYTHEMATOSUS	KEGG_SYSTEMIC_LUPUS_ERYTHEMATOSUS		91	-0.2545843	0.36243385	-1.0577612	0.6197611	1	6207	tags=43%, list=33%, signal=64%	FALSE
REACTOME_INSULIN_RECEPTOR_SIGNALLING_CASCADE	REACTOME_INSULIN_RECEPTOR_SIGNALLING_CASCADE		82	-0.25766948	0.35400516	-1.0577307	0.6182719	1	2672	tags=16%, list=14%, signal=18%	FALSE
REACTOME_IL1_SIGNALING	REACTOME_IL1_SIGNALING		37	-0.30125073	0.38294798	-1.0565541	0.61967725	1	5370	tags=38%, list=28%, signal=53%	FALSE
PID_MAPK_TRK_PATHWAY	PID_MAPK_TRK_PATHWAY		33	-0.30788204	0.3762811	-1.0535457	0.6255697	1	4960	tags=36%, list=26%, signal=49%	FALSE
BIOCARTA_MEF2D_PATHWAY	BIOCARTA_MEF2D_PATHWAY		18	-0.36114967	0.40944883	-1.0532297	0.62480915	1	3847	tags=39%, list=20%, signal=49%	FALSE
BIOCARTA_PAR1_PATHWAY	BIOCARTA_PAR1_PATHWAY		35	-0.30138728	0.39244187	-1.0523094	0.6255083	1	2857	tags=26%, list=15%, signal=30%	FALSE
REACTOME_RAS_ACTIVATION_UOPN_CA2_INFUX_THROUGH_NMDA_RECEPTOR	REACTOME_RAS_ACTIVATION_UOPN_CA2_INFUX_THROUGH_NMDA_RECEPTOR		16	-0.36019683	0.38948995	-1.047338	0.6361856	1	3997	tags=31%, list=21%, signal=40%	FALSE
PID_LYMPH_ANGIOGENESIS_PATHWAY	PID_LYMPH_ANGIOGENESIS_PATHWAY		22	-0.33984435	0.3961136	-1.043543	0.6438723	1	336	tags=14%, list=2%, signal=14%	FALSE
PID_P38_ALPHA_BETA_PATHWAY	PID_P38_ALPHA_BETA_PATHWAY		31	-0.31197578	0.37904468	-1.0434096	0.6425727	1	1389	tags=19%, list=7%, signal=21%	FALSE
REACTOME_APC_C_CDH1_MEDIATED_DEGRADATION_OF_CDC20_AND_OTHER_APC_C_CDH1_TARGETED_PROTEINS_IN_LATE_MITOSIS_EARLY_G1	REACTOME_APC_C_CDH1_MEDIATED_DEGRADATION_OF_CDC20_AND_OTHER_APC_C_CDH1_TARGETED_PROTEINS_IN_LATE_MITOSIS_EARLY_G1		59	-0.26835763	0.38395417	-1.042666	0.64280844	1	3871	tags=22%, list=20%, signal=28%	FALSE
SA_G1_AND_S_PHASES	SA_G1_AND_S_PHASES		15	-0.36875415	0.39936608	-1.040867	0.6455623	1	1322	tags=20%, list=7%, signal=21%	FALSE
BIOCARTA_MITOCHONDRIA_PATHWAY	BIOCARTA_MITOCHONDRIA_PATHWAY		20	-0.33621225	0.4254317	-1.0393975	0.647563	1	5928	tags=55%, list=31%, signal=80%	FALSE
KEGG_SELENOAMINO_ACID_METABOLISM	KEGG_SELENOAMINO_ACID_METABOLISM		23	-0.32558948	0.4009009	-1.0380418	0.64933634	1	1214	tags=17%, list=6%, signal=19%	FALSE
REACTOME_REGULATION_OF_SIGNALING_BY_CBL	REACTOME_REGULATION_OF_SIGNALING_BY_CBL		18	-0.34350985	0.41167665	-1.0376492	0.6487515	1	6828	tags=61%, list=36%, signal=96%	FALSE
REACTOME_MEIOTIC_SYNAPSIS	REACTOME_MEIOTIC_SYNAPSIS		55	-0.27121812	0.40369394	-1.0310258	0.6634	1	4647	tags=40%, list=25%, signal=53%	FALSE
BIOCARTA_GLEEVEC_PATHWAY	BIOCARTA_GLEEVEC_PATHWAY		23	-0.32041815	0.4237037	-1.029602	0.6653063	1	2765	tags=26%, list=15%, signal=31%	FALSE
KEGG_JAK_STAT_SIGNALING_PATHWAY	KEGG_JAK_STAT_SIGNALING_PATHWAY		146	-0.23269689	0.40168878	-1.0292532	0.6645769	1	2875	tags=18%, list=15%, signal=22%	FALSE
REACTOME_PD1_SIGNALING	REACTOME_PD1_SIGNALING		15	-0.36569917	0.42439026	-1.0290805	0.6633963	1	7475	tags=67%, list=40%, signal=110%	FALSE
PID_CASPASE_PATHWAY	PID_CASPASE_PATHWAY		51	-0.27341133	0.4279539	-1.0285141	0.66331357	1	5928	tags=45%, list=31%, signal=66%	FALSE
BIOCARTA_AT1R_PATHWAY	BIOCARTA_AT1R_PATHWAY		31	-0.3056995	0.41826215	-1.0281433	0.6625576	1	2286	tags=23%, list=12%, signal=26%	FALSE
REACTOME_CYCLIN_E_ASSOCIATED_EVENTS_DURING_G1_S_TRANSITION_	REACTOME_CYCLIN_E_ASSOCIATED_EVENTS_DURING_G1_S_TRANSITION_		58	-0.26666525	0.41086352	-1.0242207	0.6708422	1	5698	tags=33%, list=30%, signal=47%	FALSE
KEGG_P53_SIGNALING_PATHWAY	KEGG_P53_SIGNALING_PATHWAY		67	-0.25883302	0.43427834	-1.0241858	0.66928107	1	2196	tags=19%, list=12%, signal=22%	FALSE
KEGG_CALCIUM_SIGNALING_PATHWAY	KEGG_CALCIUM_SIGNALING_PATHWAY		173	-0.22320856	0.4079254	-1.0226241	0.6715795	1	3971	tags=21%, list=21%, signal=26%	FALSE
REACTOME_NUCLEAR_EVENTS_KINASE_AND_TRANSCRIPTION_FACTOR_ACTIVATION	REACTOME_NUCLEAR_EVENTS_KINASE_AND_TRANSCRIPTION_FACTOR_ACTIVATION		24	-0.3184472	0.43712574	-1.0213145	0.673283	1	4587	tags=33%, list=24%, signal=44%	FALSE
REACTOME_SIGNALING_BY_FGFR_IN_DISEASE	REACTOME_SIGNALING_BY_FGFR_IN_DISEASE		121	-0.23311485	0.43965518	-1.0181928	0.6798954	1	5230	tags=28%, list=28%, signal=39%	FALSE
PID_IL2_1PATHWAY	PID_IL2_1PATHWAY		55	-0.267024	0.4352617	-1.0175706	0.679707	1	2857	tags=24%, list=15%, signal=28%	FALSE
REACTOME_CDT1_ASSOCIATION_WITH_THE_CDC6_ORC_ORIGIN_COMPLEX	REACTOME_CDT1_ASSOCIATION_WITH_THE_CDC6_ORC_ORIGIN_COMPLEX		51	-0.27297983	0.46534654	-1.0164672	0.68078804	1	2837	tags=18%, list=15%, signal=21%	FALSE
REACTOME_AMINO_ACID_SYNTHESIS_AND_INTERCONVERSION_TRANSAMINATION	REACTOME_AMINO_ACID_SYNTHESIS_AND_INTERCONVERSION_TRANSAMINATION		16	-0.36053297	0.44323483	-1.015361	0.681862	1	5516	tags=44%, list=29%, signal=62%	FALSE
PID_CD8_TCR_DOWNSTREAM_PATHWAY	PID_CD8_TCR_DOWNSTREAM_PATHWAY		61	-0.25695965	0.45192307	-1.0151674	0.6806866	1	3850	tags=26%, list=20%, signal=33%	FALSE
BIOCARTA_DC_PATHWAY	BIOCARTA_DC_PATHWAY		22	-0.3248047	0.45327103	-1.0128523	0.68490994	1	7792	tags=55%, list=41%, signal=93%	FALSE
PID_TCR_CALCIUM_PATHWAY	PID_TCR_CALCIUM_PATHWAY		29	-0.30535403	0.44065282	-1.0118607	0.6857349	1	2391	tags=24%, list=13%, signal=28%	FALSE
KEGG_PHOSPHATIDYLINOSITOL_SIGNALING_SYSTEM	KEGG_PHOSPHATIDYLINOSITOL_SIGNALING_SYSTEM		76	-0.24758393	0.4302949	-1.0110812	0.685976	1	3036	tags=22%, list=16%, signal=27%	FALSE
REACTOME_P75_NTR_RECEPTOR_MEDIATED_SIGNALLING	REACTOME_P75_NTR_RECEPTOR_MEDIATED_SIGNALLING		78	-0.24731797	0.4555256	-1.0079663	0.69200844	1	4930	tags=32%, list=26%, signal=43%	FALSE
BIOCARTA_IL2_PATHWAY	BIOCARTA_IL2_PATHWAY		22	-0.32192278	0.4592934	-1.0076011	0.6912929	1	3393	tags=27%, list=18%, signal=33%	FALSE
BIOCARTA_IL22BP_PATHWAY	BIOCARTA_IL22BP_PATHWAY		16	-0.3474753	0.4600639	-1.0068592	0.69143295	1	2765	tags=25%, list=15%, signal=29%	FALSE
REACTOME_GAB1_SIGNALOSOME	REACTOME_GAB1_SIGNALOSOME		36	-0.28695363	0.43235704	-1.003369	0.6984005	1	5035	tags=28%, list=27%, signal=38%	FALSE
BIOCARTA_NTHI_PATHWAY	BIOCARTA_NTHI_PATHWAY		24	-0.3110512	0.49109793	-1.0023028	0.6993912	1	3810	tags=29%, list=20%, signal=36%	FALSE
PID_INSULIN_PATHWAY	PID_INSULIN_PATHWAY		45	-0.27157214	0.4465318	-1.0014946	0.6997901	1	3423	tags=27%, list=18%, signal=32%	FALSE
KEGG_PROSTATE_CANCER	KEGG_PROSTATE_CANCER		87	-0.24076173	0.45525292	-0.9974961	0.7078845	1	4753	tags=29%, list=25%, signal=38%	FALSE
KEGG_RIBOFLAVIN_METABOLISM	KEGG_RIBOFLAVIN_METABOLISM		16	-0.34077504	0.46329528	-0.9968815	0.7077895	1	3996	tags=38%, list=21%, signal=48%	FALSE
BIOCARTA_MAL_PATHWAY	BIOCARTA_MAL_PATHWAY		19	-0.33211535	0.47230768	-0.99654204	0.70691663	1	6181	tags=42%, list=33%, signal=63%	FALSE
REACTOME_TAK1_ACTIVATES_NFKB_BY_PHOSPHORYLATION_AND_ACTIVATION_OF_IKKS_COMPLEX	REACTOME_TAK1_ACTIVATES_NFKB_BY_PHOSPHORYLATION_AND_ACTIVATION_OF_IKKS_COMPLEX		21	-0.32111806	0.47928995	-0.9929821	0.71415913	1	6000	tags=48%, list=32%, signal=70%	FALSE
REACTOME_NOD1_2_SIGNALING_PATHWAY	REACTOME_NOD1_2_SIGNALING_PATHWAY		28	-0.30057847	0.498452	-0.9916236	0.7158395	1	6000	tags=50%, list=32%, signal=73%	FALSE
PID_EPHA_FWDPATHWAY	PID_EPHA_FWDPATHWAY		34	-0.28551212	0.49853373	-0.98998266	0.718319	1	1389	tags=18%, list=7%, signal=19%	FALSE
PID_IL4_2PATHWAY	PID_IL4_2PATHWAY		63	-0.25117332	0.48233697	-0.9884391	0.7205929	1	4591	tags=24%, list=24%, signal=31%	FALSE
BIOCARTA_INSULIN_PATHWAY	BIOCARTA_INSULIN_PATHWAY		22	-0.3164762	0.46119404	-0.98742074	0.7213842	1	4960	tags=36%, list=26%, signal=49%	FALSE
KEGG_GLIOMA	KEGG_GLIOMA		65	-0.24795873	0.50334674	-0.98447925	0.7272036	1	1322	tags=12%, list=7%, signal=13%	FALSE
REACTOME_SIGNALING_BY_WNT	REACTOME_SIGNALING_BY_WNT		59	-0.2529172	0.49584487	-0.98388153	0.72702986	1	2816	tags=19%, list=15%, signal=22%	FALSE
REACTOME_SYNTHESIS_OF_PIPS_AT_THE_PLASMA_MEMBRANE	REACTOME_SYNTHESIS_OF_PIPS_AT_THE_PLASMA_MEMBRANE		30	-0.28868303	0.4760479	-0.981844	0.73033357	1	5959	tags=43%, list=32%, signal=63%	FALSE
HALLMARK_PI3K_AKT_MTOR_SIGNALING	HALLMARK_PI3K_AKT_MTOR_SIGNALING		104	-0.23295754	0.5116883	-0.98154557	0.72943556	1	3743	tags=21%, list=20%, signal=26%	FALSE
SA_B_CELL_RECEPTOR_COMPLEXES	SA_B_CELL_RECEPTOR_COMPLEXES		24	-0.30804282	0.48153618	-0.9785811	0.73517144	1	4329	tags=33%, list=23%, signal=43%	FALSE
REACTOME_SIGNALLING_BY_NGF	REACTOME_SIGNALLING_BY_NGF		211	-0.2103028	0.52878964	-0.97847337	0.73379797	1	5409	tags=29%, list=29%, signal=41%	FALSE
PID_TCPTP_PATHWAY	PID_TCPTP_PATHWAY		41	-0.26799178	0.48714286	-0.973578	0.744249	1	3027	tags=22%, list=16%, signal=26%	FALSE
PID_FOXO_PATHWAY	PID_FOXO_PATHWAY		47	-0.26241362	0.4979424	-0.9731389	0.7436163	1	5389	tags=36%, list=29%, signal=50%	FALSE
KEGG_HEDGEHOG_SIGNALING_PATHWAY	KEGG_HEDGEHOG_SIGNALING_PATHWAY		53	-0.2577365	0.4985591	-0.9730613	0.742165	1	5881	tags=38%, list=31%, signal=55%	FALSE
HALLMARK_MTORC1_SIGNALING	HALLMARK_MTORC1_SIGNALING		190	-0.21043815	0.5346651	-0.97092164	0.7456335	1	4117	tags=25%, list=22%, signal=32%	FALSE
REACTOME_HEMOSTASIS	REACTOME_HEMOSTASIS		426	-0.19482526	0.5743315	-0.9708262	0.74420714	1	2959	tags=18%, list=16%, signal=21%	FALSE
BIOCARTA_ATM_PATHWAY	BIOCARTA_ATM_PATHWAY		19	-0.32271555	0.4946401	-0.97077954	0.74265677	1	4150	tags=37%, list=22%, signal=47%	FALSE
REACTOME_EGFR_DOWNREGULATION	REACTOME_EGFR_DOWNREGULATION		24	-0.3044767	0.5	-0.9705568	0.74156195	1	1770	tags=17%, list=9%, signal=18%	FALSE
BIOCARTA_GPCR_PATHWAY	BIOCARTA_GPCR_PATHWAY		33	-0.28173694	0.4962742	-0.9694204	0.7426503	1	5905	tags=39%, list=31%, signal=57%	FALSE
BIOCARTA_TOLL_PATHWAY	BIOCARTA_TOLL_PATHWAY		36	-0.27721643	0.5123726	-0.9682742	0.7438532	1	3810	tags=28%, list=20%, signal=35%	FALSE
PID_THROMBIN_PAR1_PATHWAY	PID_THROMBIN_PAR1_PATHWAY		42	-0.26489812	0.52578795	-0.9669386	0.7455377	1	4996	tags=31%, list=26%, signal=42%	FALSE
REACTOME_CELL_DEATH_SIGNALLING_VIA_NRAGE_NRIF_AND_NADE	REACTOME_CELL_DEATH_SIGNALLING_VIA_NRAGE_NRIF_AND_NADE		57	-0.2523149	0.53185594	-0.9666569	0.744618	1	1853	tags=18%, list=10%, signal=19%	FALSE
BIOCARTA_RACCYCD_PATHWAY	BIOCARTA_RACCYCD_PATHWAY		25	-0.3006032	0.52444446	-0.9600176	0.7591899	1	5547	tags=44%, list=29%, signal=62%	FALSE
KEGG_NEUROACTIVE_LIGAND_RECEPTOR_INTERACTION	KEGG_NEUROACTIVE_LIGAND_RECEPTOR_INTERACTION		267	-0.20183478	0.5825688	-0.95585483	0.76803833	1	5289	tags=27%, list=28%, signal=36%	FALSE
REACTOME_PRE_NOTCH_PROCESSING_IN_GOLGI	REACTOME_PRE_NOTCH_PROCESSING_IN_GOLGI		16	-0.3345135	0.5159332	-0.9549745	0.76848227	1	3930	tags=25%, list=21%, signal=32%	FALSE
REACTOME_OPIOID_SIGNALLING	REACTOME_OPIOID_SIGNALLING		76	-0.23605634	0.5545335	-0.9544356	0.7681483	1	5557	tags=30%, list=29%, signal=43%	FALSE
BIOCARTA_GATA3_PATHWAY	BIOCARTA_GATA3_PATHWAY		16	-0.32646653	0.5194805	-0.9533372	0.76913273	1	2789	tags=19%, list=15%, signal=22%	FALSE
BIOCARTA_SPRY_PATHWAY	BIOCARTA_SPRY_PATHWAY		18	-0.32030883	0.51969695	-0.94708484	0.78239244	1	2099	tags=17%, list=11%, signal=19%	FALSE
BIOCARTA_STRESS_PATHWAY	BIOCARTA_STRESS_PATHWAY		24	-0.28998968	0.54615384	-0.9453652	0.7849042	1	4357	tags=33%, list=23%, signal=43%	FALSE
REACTOME_MAP_KINASE_ACTIVATION_IN_TLR_CASCADE	REACTOME_MAP_KINASE_ACTIVATION_IN_TLR_CASCADE		48	-0.25481826	0.5423729	-0.94529694	0.7833702	1	4603	tags=29%, list=24%, signal=38%	FALSE
PID_IL1_PATHWAY	PID_IL1_PATHWAY		31	-0.27990618	0.5432277	-0.9449069	0.7826174	1	5370	tags=39%, list=28%, signal=54%	FALSE
BIOCARTA_BARRESTIN_SRC_PATHWAY	BIOCARTA_BARRESTIN_SRC_PATHWAY		15	-0.33571896	0.5318471	-0.94144446	0.7895877	1	1274	tags=13%, list=7%, signal=14%	FALSE
KEGG_ALANINE_ASPARTATE_AND_GLUTAMATE_METABOLISM	KEGG_ALANINE_ASPARTATE_AND_GLUTAMATE_METABOLISM		32	-0.27363735	0.54761904	-0.9410791	0.7887806	1	5435	tags=38%, list=29%, signal=53%	FALSE
REACTOME_RAP1_SIGNALLING	REACTOME_RAP1_SIGNALLING		15	-0.32952115	0.5416667	-0.94080776	0.7877656	1	2566	tags=27%, list=14%, signal=31%	FALSE
KEGG_INTESTINAL_IMMUNE_NETWORK_FOR_IGA_PRODUCTION	KEGG_INTESTINAL_IMMUNE_NETWORK_FOR_IGA_PRODUCTION		43	-0.2576469	0.58129495	-0.9397425	0.7886436	1	8124	tags=58%, list=43%, signal=102%	FALSE
KEGG_WNT_SIGNALING_PATHWAY	KEGG_WNT_SIGNALING_PATHWAY		146	-0.21178064	0.60692215	-0.9395182	0.78748786	1	3503	tags=22%, list=19%, signal=27%	FALSE

KEGG_ANTIGEN_PROCESSING_AND_PRESENTATION	KEGG_ANTIGEN_PROCESSING_AND_PRESENTATION		70	-0.23668228	0.58077437	-0.9391173	0.7867481	1	6189	tags=40%, list=33%, signal=59%	FALSE
BIOCARTA_KERATINOCYTE_PATHWAY	BIOCARTA_KERATINOCYTE_PATHWAY		46	-0.25325873	0.5832168	-0.9382403	0.78713816	1	3810	tags=26%, list=20%, signal=33%	FALSE
PID_AR_NONGENOMIC_PATHWAY	PID_AR_NONGENOMIC_PATHWAY		31	-0.28209895	0.5557185	-0.93791467	0.7863053	1	6649	tags=48%, list=35%, signal=75%	FALSE
REACTOME_REGULATION_OF_WATER_BALANCE_BY_RENAL_AQUAPORINS	REACTOME_REGULATION_OF_WATER_BALANCE_BY_RENAL_AQUAPORINS		42	-0.258678	0.55604076	-0.937599	0.7853896	1	6908	tags=40%, list=37%, signal=64%	FALSE
REACTOME_PHOSPHOLIPASE_C_MEDIATED_CASCADE	REACTOME_PHOSPHOLIPASE_C_MEDIATED_CASCADE		53	-0.24816589	0.57466066	-0.93449616	0.7909671	1	7831	tags=45%, list=41%, signal=77%	FALSE
REACTOME_PROSTACYCLIN_SIGNALLING_THROUGH_PROSTACYCLIN_RECEPTOR	REACTOME_PROSTACYCLIN_SIGNALLING_THROUGH_PROSTACYCLIN_RECEPTOR		18	-0.30919978	0.55365473	-0.93408585	0.79026014	1	1627	tags=17%, list=9%, signal=18%	FALSE
REACTOME_SHC_RELATED_EVENTS	REACTOME_SHC_RELATED_EVENTS		17	-0.32118142	0.56288344	-0.93001777	0.79837346	1	1274	tags=18%, list=7%, signal=19%	FALSE
REACTOME_ACTIVATION_OF_NF_KAPPAB_IN_B_CELLS	REACTOME_ACTIVATION_OF_NF_KAPPAB_IN_B_CELLS		57	-0.2424833	0.5882353	-0.92713934	0.8033941	1	3810	tags=21%, list=20%, signal=26%	FALSE
REACTOME_VIF_MEDIATED_DEGRADATION_OF_APOBEC3G	REACTOME_VIF_MEDIATED_DEGRADATION_OF_APOBEC3G		46	-0.25153813	0.57567185	-0.9270343	0.8019521	1	864	tags=11%, list=5%, signal=11%	FALSE
KEGG_AUTOIMMUNE_THYROID_DISEASE	KEGG_AUTOIMMUNE_THYROID_DISEASE		43	-0.25339794	0.5858156	-0.9256999	0.8033831	1	6072	tags=37%, list=32%, signal=55%	FALSE
REACTOME_SIGNALING_BY_ERBB2	REACTOME_SIGNALING_BY_ERBB2		97	-0.22019507	0.61243385	-0.92567885	0.8017498	1	5409	tags=30%, list=29%, signal=42%	FALSE
BIOCARTA_CHEMICAL_PATHWAY	BIOCARTA_CHEMICAL_PATHWAY		22	-0.29899853	0.59431136	-0.9213765	0.81027	1	5613	tags=50%, list=30%, signal=71%	FALSE
REACTOME_BMAL1_CLOCK_NPAS2_ACTIVATES_CIRCADIAN_EXPRESSION	REACTOME_BMAL1_CLOCK_NPAS2_ACTIVATES_CIRCADIAN_EXPRESSION		36	-0.26185504	0.5816024	-0.9209153	0.80970603	1	3303	tags=31%, list=17%, signal=37%	FALSE
PID_PTP1B_PATHWAY	PID_PTP1B_PATHWAY		50	-0.24579754	0.60928965	-0.9199388	0.8102816	1	3424	tags=26%, list=18%, signal=32%	FALSE
REACTOME_GLUCOSE_METABOLISM	REACTOME_GLUCOSE_METABOLISM		63	-0.23932117	0.58998644	-0.9192636	0.810173	1	5905	tags=37%, list=31%, signal=53%	FALSE
KEGG_GNRH_SIGNALING_PATHWAY	KEGG_GNRH_SIGNALING_PATHWAY		95	-0.21719304	0.6176084	-0.91863203	0.81008446	1	5905	tags=34%, list=31%, signal=49%	FALSE
PID_ANGIOPOIETIN_RECEPTOR_PATHWAY	PID_ANGIOPOIETIN_RECEPTOR_PATHWAY		49	-0.24391514	0.5982659	-0.91861796	0.8084562	1	4591	tags=29%, list=24%, signal=38%	FALSE
REACTOME_DEPOSITION_OF_NEW_CENPA_CONTAINING_NUCLEOSOMES_AT_THE_CENTROMERE	REACTOME_DEPOSITION_OF_NEW_CENPA_CONTAINING_NUCLEOSOMES_AT_THE_CENTROMERE		45	-0.25084388	0.5828652	-0.91582006	0.8131082	1	4909	tags=38%, list=26%, signal=51%	FALSE
PID_P38_ALPHA_BETA_DOWNSTREAM_PATHWAY	PID_P38_ALPHA_BETA_DOWNSTREAM_PATHWAY		36	-0.25920385	0.60178304	-0.9153036	0.81263524	1	4104	tags=31%, list=22%, signal=39%	FALSE
REACTOME_SIGNALING_BY_CONSTITUTIVELY_ACTIVE_EGFR	REACTOME_SIGNALING_BY_CONSTITUTIVELY_ACTIVE_EGFR		17	-0.31758556	0.5786164	-0.91278	0.8167951	1	5230	tags=35%, list=28%, signal=49%	FALSE
HALLMARK_WNT_BETA_CATENIN_SIGNALING	HALLMARK_WNT_BETA_CATENIN_SIGNALING		42	-0.2500916	0.59615386	-0.9116637	0.8177116	1	2510	tags=21%, list=13%, signal=25%	FALSE
REACTOME_DARPP_32_EVENTS	REACTOME_DARPP_32_EVENTS		24	-0.28555596	0.5904335	-0.9112825	0.81694835	1	7605	tags=58%, list=40%, signal=98%	FALSE
REACTOME_SIGNALING_BY_ERBB4	REACTOME_SIGNALING_BY_ERBB4		87	-0.21978237	0.6586667	-0.91098744	0.8159569	1	5035	tags=28%, list=27%, signal=37%	FALSE
REACTOME_ACTIVATED_TAK1_MEDIATES_P38_MAPK_ACTIVATION	REACTOME_ACTIVATED_TAK1_MEDIATES_P38_MAPK_ACTIVATION		16	-0.31765378	0.5996835	-0.909656	0.81752735	1	7318	tags=50%, list=39%, signal=82%	FALSE
BIOCARTA_HER2_PATHWAY	BIOCARTA_HER2_PATHWAY		22	-0.28954908	0.6050671	-0.9071156	0.8215413	1	2542	tags=23%, list=13%, signal=26%	FALSE
REACTOME_SIGNALING_BY_FGFR	REACTOME_SIGNALING_BY_FGFR		108	-0.21185338	0.6650062	-0.90624243	0.8218475	1	5409	tags=28%, list=29%, signal=39%	FALSE
REACTOME_ADP_SIGNALLING_THROUGH_P2RY12	REACTOME_ADP_SIGNALLING_THROUGH_P2RY12		20	-0.29284295	0.58044165	-0.9053637	0.82215214	1	3969	tags=25%, list=21%, signal=32%	FALSE
REACTOME_NEUROTRANSMITTER_RECEPTOR_BINDING_AND_DOWNSTREAM_TRANSMISSION_IN_THE_POSTSYNAPTIC_CELL	REACTOME_NEUROTRANSMITTER_RECEPTOR_BINDING_AND_DOWNSTREAM_TRANSMISSION_IN_THE_POSTSYNAPTIC_CELL		131	-0.20456395	0.66142684	-0.9048824	0.8215457	1	3997	tags=21%, list=21%, signal=26%	FALSE
PID_IL12_2PATHWAY	PID_IL12_2PATHWAY		62	-0.23027296	0.6282225	-0.9042662	0.82128686	1	6926	tags=40%, list=37%, signal=63%	FALSE
REACTOME_GABA_RECEPTOR_ACTIVATION	REACTOME_GABA_RECEPTOR_ACTIVATION		51	-0.24004398	0.62274617	-0.9042253	0.819763	1	5510	tags=31%, list=29%, signal=44%	FALSE
REACTOME_ACTIVATED_AMPK_STIMULATES_FATTY_ACID_OXIDATION_IN_MUSCLE	REACTOME_ACTIVATED_AMPK_STIMULATES_FATTY_ACID_OXIDATION_IN_MUSCLE		16	-0.31680378	0.58747995	-0.9027598	0.82135934	1	1664	tags=13%, list=9%, signal=14%	FALSE
BIOCARTA_MPR_PATHWAY	BIOCARTA_MPR_PATHWAY		33	-0.26067406	0.6304985	-0.8988312	0.82856095	1	3812	tags=24%, list=20%, signal=30%	FALSE
BIOCARTA_CDC42RAC_PATHWAY	BIOCARTA_CDC42RAC_PATHWAY		15	-0.32031083	0.6137026	-0.8978645	0.82912296	1	5073	tags=47%, list=27%, signal=64%	FALSE
REACTOME_G_ALPHA_Z_SIGNALLING_EVENTS	REACTOME_G_ALPHA_Z_SIGNALLING_EVENTS		43	-0.24913071	0.6179775	-0.8973861	0.8286283	1	5510	tags=30%, list=29%, signal=43%	FALSE
REACTOME_P53_DEPENDENT_G1_DNA_DAMAGE_RESPONSE	REACTOME_P53_DEPENDENT_G1_DNA_DAMAGE_RESPONSE		50	-0.23793209	0.6407355	-0.89666516	0.828638	1	2139	tags=14%, list=11%, signal=16%	FALSE
REACTOME_KINESINS	REACTOME_KINESINS		23	-0.2852782	0.6132353	-0.8950137	0.83067954	1	3294	tags=22%, list=17%, signal=26%	FALSE
REACTOME_G_ALPHA_I_SIGNALLING_EVENTS	REACTOME_G_ALPHA_I_SIGNALLING_EVENTS		182	-0.1962625	0.7117438	-0.89254534	0.83453256	1	5581	tags=29%, list=30%, signal=41%	FALSE
REACTOME_THROMBIN_SIGNALLING_THROUGH_PROTEINASE_ACTIVATED_RECEPTORS_PARS	REACTOME_THROMBIN_SIGNALLING_THROUGH_PROTEINASE_ACTIVATED_RECEPTORS_PARS		29	-0.2672388	0.6393939	-0.8912743	0.835754	1	2302	tags=17%, list=12%, signal=20%	FALSE
BIOCARTA_ACH_PATHWAY	BIOCARTA_ACH_PATHWAY		16	-0.31026673	0.60787404	-0.8912534	0.8341321	1	4591	tags=38%, list=24%, signal=49%	FALSE
REACTOME_AMINE_LIGAND_BINDING_RECEPTORS	REACTOME_AMINE_LIGAND_BINDING_RECEPTORS		36	-0.25283828	0.6454948	-0.8873881	0.84094083	1	5571	tags=31%, list=29%, signal=43%	FALSE
REACTOME_NGF_SIGNALLING_VIA_TRKA_FROM_THE_PLASMA_MEMBRANE	REACTOME_NGF_SIGNALLING_VIA_TRKA_FROM_THE_PLASMA_MEMBRANE		134	-0.20052831	0.710269	-0.88494444	0.84475094	1	5230	tags=27%, list=28%, signal=37%	FALSE
BIOCARTA_NFKB_PATHWAY	BIOCARTA_NFKB_PATHWAY		22	-0.28377628	0.64878047	-0.8848212	0.8433724	1	3810	tags=32%, list=20%, signal=40%	FALSE
KEGG_VALINE_LEUCINE_AND_ISOLEUCINE_DEGRADATION	KEGG_VALINE_LEUCINE_AND_ISOLEUCINE_DEGRADATION		43	-0.24424697	0.6325967	-0.8843481	0.8427839	1	4499	tags=28%, list=24%, signal=37%	FALSE
REACTOME_NEF_MEDIATES_DOWN_MODULATION_OF_CELL_SURFACE_RECEPTORS_BY_RECRUITING_THEM_TO_CLATHRIN_ADAPTERS	REACTOME_NEF_MEDIATES_DOWN_MODULATION_OF_CELL_SURFACE_RECEPTORS_BY_RECRUITING_THEM_TO_CLATHRIN_ADAPTERS		20	-0.2925269	0.6343402	-0.88421065	0.8414161	1	8510	tags=60%, list=45%, signal=109%	FALSE
REACTOME_NUCLEAR_SIGNALING_BY_ERBB4	REACTOME_NUCLEAR_SIGNALING_BY_ERBB4		38	-0.24371724	0.66369045	-0.88192016	0.8447866	1	5022	tags=32%, list=27%, signal=43%	FALSE
REACTOME_SIGNAL_AMPLIFICATION	REACTOME_SIGNAL_AMPLIFICATION		29	-0.26755795	0.6237337	-0.8814054	0.84427196	1	1627	tags=14%, list=9%, signal=15%	FALSE
ST_FAS_SIGNALING_PATHWAY	ST_FAS_SIGNALING_PATHWAY		62	-0.22213031	0.66574967	-0.8811851	0.8431164	1	4205	tags=26%, list=22%, signal=33%	FALSE
KEGG_VASOPRESSIN_REGULATED_WATER_REABSORPTION	KEGG_VASOPRESSIN_REGULATED_WATER_REABSORPTION		43	-0.24223456	0.66429585	-0.8804439	0.8430985	1	4192	tags=23%, list=22%, signal=30%	FALSE
REACTOME_PREFOLDIN_MEDIATED_TRANSFER_OF_SUBSTRATE_TO_CCT_TRIC	REACTOME_PREFOLDIN_MEDIATED_TRANSFER_OF_SUBSTRATE_TO_CCT_TRIC		26	-0.26994726	0.642527	-0.8798413	0.8426904	1	4864	tags=35%, list=26%, signal=47%	FALSE
REACTOME_AUTODEGRADATION_OF_THE_E3_UBIQUITIN_LIGASE_COP1	REACTOME_AUTODEGRADATION_OF_THE_E3_UBIQUITIN_LIGASE_COP1		44	-0.24523494	0.66519827	-0.87977767	0.84119093	1	2816	tags=16%, list=15%, signal=19%	FALSE
REACTOME_REGULATION_OF_IFNA_SIGNALING	REACTOME_REGULATION_OF_IFNA_SIGNALING		19	-0.29240066	0.63722396	-0.87738764	0.844629	1	3706	tags=32%, list=20%, signal=39%	FALSE
REACTOME_GLUCONEOGENESIS	REACTOME_GLUCONEOGENESIS		30	-0.26262906	0.6433878	-0.8768861	0.8440948	1	5293	tags=33%, list=28%, signal=46%	FALSE
PID_CERAMIDE_PATHWAY	PID_CERAMIDE_PATHWAY		48	-0.24072854	0.6693989	-0.87675804	0.8427623	1	3520	tags=25%, list=19%, signal=31%	FALSE
REACTOME_INTEGRIN_ALPHAIIB_BETA3_SIGNALING	REACTOME_INTEGRIN_ALPHAIIB_BETA3_SIGNALING		26	-0.26741257	0.652439	-0.87635714	0.84198743	1	334	tags=12%, list=2%, signal=12%	FALSE
KEGG_INOSITOL_PHOSPHATE_METABOLISM	KEGG_INOSITOL_PHOSPHATE_METABOLISM		54	-0.2306838	0.6790634	-0.87438464	0.84451014	1	3036	tags=22%, list=16%, signal=26%	FALSE
REACTOME_DOWNSTREAM_SIGNALING_OF_ACTIVATED_FGFR	REACTOME_DOWNSTREAM_SIGNALING_OF_ACTIVATED_FGFR		97	-0.20511422	0.6978233	-0.8733436	0.84506375	1	5409	tags=27%, list=29%, signal=37%	FALSE
KEGG_TGF_BETA_SIGNALING_PATHWAY	KEGG_TGF_BETA_SIGNALING_PATHWAY		83	-0.21435508	0.7003891	-0.87262964	0.8449057	1	3788	tags=25%, list=20%, signal=32%	FALSE
BIOCARTA_AKT_PATHWAY	BIOCARTA_AKT_PATHWAY		21	-0.2829313	0.64102566	-0.87010705	0.84866744	1	5022	tags=38%, list=27%, signal=52%	FALSE
REACTOME_CTNNB1_PHOSPHORYLATION_CASCADE	REACTOME_CTNNB1_PHOSPHORYLATION_CASCADE		15	-0.30914256	0.64759034	-0.869321	0.84871644	1	2509	tags=27%, list=13%, signal=31%	FALSE
KEGG_PRION_DISEASES	KEGG_PRION_DISEASES		35	-0.2471262	0.6596343	-0.86842525	0.8490649	1	2566	tags=17%, list=14%, signal=20%	FALSE
REACTOME_EFFECTS_OF_PIP2_HYDROLYSIS	REACTOME_EFFECTS_OF_PIP2_HYDROLYSIS		22	-0.27300045	0.67434716	-0.86728925	0.8497629	1	2461	tags=23%, list=13%, signal=26%	FALSE
REACTOME_MAPK_TARGETS_NUCLEAR_EVENTS_MEDIATED_BY_MAP_KINASES	REACTOME_MAPK_TARGETS_NUCLEAR_EVENTS_MEDIATED_BY_MAP_KINASES		30	-0.25695738	0.65966386	-0.8666166	0.8495677	1	4587	tags=33%, list=24%, signal=44%	FALSE
KEGG_CARDIAC_MUSCLE_CONTRACTION	KEGG_CARDIAC_MUSCLE_CONTRACTION		68	-0.21780321	0.7008197	-0.8653261	0.8507184	1	6070	tags=35%, list=32%, signal=52%	FALSE
REACTOME_P53_INDEPENDENT_G1_S_DNA_DAMAGE_CHECKPOINT	REACTOME_P53_INDEPENDENT_G1_S_DNA_DAMAGE_CHECKPOINT		44	-0.23614612	0.6747851	-0.86511207	0.8495433	1	2816	tags=16%, list=15%, signal=19%	FALSE
REACTOME_REGULATION_OF_KIT_SIGNALING	REACTOME_REGULATION_OF_KIT_SIGNALING		16	-0.3026478	0.6615147	-0.8636322	0.8510715	1	334	tags=13%, list=2%, signal=13%	FALSE
REACTOME_GASTRIN_CREB_SIGNALLING_PATHWAY_VIA_PKC_AND_MAPK	REACTOME_GASTRIN_CREB_SIGNALLING_PATHWAY_VIA_PKC_AND_MAPK		187	-0.18576922	0.76615745	-0.86151475	0.85370976	1	3103	tags=18%, list=16%, signal=22%	FALSE
REACTOME_GLYCOGEN_BREAKDOWN_GLYCOGENOLYSIS	REACTOME_GLYCOGEN_BREAKDOWN_GLYCOGENOLYSIS		16	-0.3002284	0.6575758	-0.86103505	0.85306525	1	5905	tags=44%, list=31%, signal=64%	FALSE
KEGG_GLYOXYLATE_AND_DICARBOXYLATE_METABOLISM	KEGG_GLYOXYLATE_AND_DICARBOXYLATE_METABOLISM		15	-0.30784723	0.6312595	-0.8592614	0.8552212	1	6077	tags=47%, list=32%, signal=69%	FALSE
KEGG_PROTEIN_EXPORT	KEGG_PROTEIN_EXPORT		23	-0.27614826	0.67275494	-0.8575787	0.8570059	1	2718	tags=22%, list=14%, signal=25%	FALSE
REACTOME_SCF_BETA_TRCP_MEDIATED_DEGRADATION_OF_EMI1	REACTOME_SCF_BETA_TRCP_MEDIATED_DEGRADATION_OF_EMI1		46	-0.23434357	0.7053206	-0.85587865	0.8588111	1	5483	tags=28%, list=29%, signal=40%	FALSE
HALLMARK_REACTIVE_OXIGEN_SPECIES_PATHWAY	HALLMARK_REACTIVE_OXIGEN_SPECIES_PATHWAY		48	-0.23044136	0.7216783	-0.85345495	0.86201257	1	3806	tags=21%, list=20%, signal=26%	FALSE
SA_CASPASE_CASCADE	SA_CASPASE_CASCADE		19	-0.28553092	0.68944097	-0.8485658	0.8701489	1	5928	tags=47%, list=31%, signal=69%	FALSE
BIOCARTA_CASPASE_PATHWAY	BIOCARTA_CASPASE_PATHWAY		23	-0.2651332	0.6994048	-0.84796387	0.86977607	1	1226	tags=17%, list=6%, signal=19%	FALSE
KEGG_RENAL_CELL_CARCINOMA	KEGG_RENAL_CELL_CARCINOMA		67	-0.21504883	0.737931	-0.8476772	0.86874145	1	2488	tags=18%, list=13%, signal=21%	FALSE
REACTOME_DOWNSTREAM_SIGNALING_EVENTS_OF_B_CELL_RECEPTOR_BCR	REACTOME_DOWNSTREAM_SIGNALING_EVENTS_OF_B_CELL_RECEPTOR_BCR		88	-0.20212057	0.75195825	-0.8461079	0.8702388	1	5483	tags=26%, list=29%, signal=37%	FALSE
KEGG_GRAFT_VERSUS_HOST_DISEASE	KEGG_GRAFT_VERSUS_HOST_DISEASE		34	-0.24462202	0.71040726	-0.8449782	0.8708193	1	8568	tags=68%, list=45%, signal=124%	FALSE
REACTOME_CLASS_A1_RHODOPSIN_LIKE_RECEPTORS	REACTOME_CLASS_A1_RHODOPSIN_LIKE_RECEPTORS		276	-0.1767564	0.8654708	-0.8434971	0.87214905	1	5289	tags=27%, list=28%, signal=37%	FALSE
PID_ALPHA_SYNUCLEIN_PATHWAY	PID_ALPHA_SYNUCLEIN_PATHWAY		33	-0.24696948	0.71556884	-0.84199214	0.8734849	1	3520	tags=24%, list=19%, signal=30%	FALSE
BIOCARTA_EIF4_PATHWAY	BIOCARTA_EIF4_PATHWAY		22	-0.27319488	0.69349843	-0.8417819	0.87231266	1	9180	tags=59%, list=49%, signal=115%	FALSE
BIOCARTA_STATHMIN_PATHWAY	BIOCARTA_STATHMIN_PATHWAY		19	-0.2840034	0.69110763	-0.83911985	0.87590516	1	7505	tags=42%, list=40%, signal=70%	FALSE
ST_DIFFERENTIATION_PATHWAY_IN_PC12_CELLS	ST_DIFFERENTIATION_PATHWAY_IN_PC12_CELLS		42	-0.22954987	0.71327686	-0.8380422	0.87639654	1	3366	tags=21%, list=18%, signal=26%	FALSE
BIOCARTA_41BB_PATHWAY	BIOCARTA_41BB_PATHWAY		17	-0.2881861	0.6698413	-0.83597064	0.87883955	1	3810	tags=35%, list=20%, signal=44%	FALSE
REACTOME_G_ALPHA_Q_SIGNALLING_EVENTS	REACTOME_G_ALPHA_Q_SIGNALLING_EVENTS		166	-0.18331827	0.8035714	-0.8359637	0.8772624	1	3103	tags=18%, list=16%, signal=21%	FALSE
PID_INSULIN_GLUCOSE_PATHWAY	PID_INSULIN_GLUCOSE_PATHWAY		25	-0.25598058	0.6858476	-0.8344774	0.8786155	1	3911	tags=28%, list=21%, signal=35%	FALSE
REACTOME_PLATELET_HOMEOSTASIS	REACTOME_PLATELET_HOMEOSTASIS		73	-0.20748533	0.76666665	-0.8341874	0.8776396	1	2758	tags=15%, list=15%, signal=18%	FALSE
KEGG_VASCULAR_SMOOTH_MUSCLE_CONTRACTION	KEGG_VASCULAR_SMOOTH_MUSCLE_CONTRACTION		108	-0.1939477	0.7992126	-0.83273226	0.87865674	1	5203	tags=29%, list=28%, signal=39%	FALSE
KEGG_VEGF_SIGNALING_PATHWAY	KEGG_VEGF_SIGNALING_PATHWAY		71	-0.20783983	0.7671602	-0.8303099	0.88160044	1	6105	tags=39%, list=32%, signal=58%	FALSE
BIOCARTA_IGF1_PATHWAY	BIOCARTA_IGF1_PATHWAY		21	-0.26818046	0.6971609	-0.8302246	0.88015753	1	4960	tags=33%, list=26%, signal=45%	FALSE
REACTOME_CITRIC_ACID_CYCLE_TCA_CYCLE	REACTOME_CITRIC_ACID_CYCLE_TCA_CYCLE		17	-0.28383565	0.69952303	-0.82998997	0.87905884	1	8867	tags=65%, list=47%, signal=122%	FALSE
REACTOME_ERK_MAPK_TARGETS	REACTOME_ERK_MAPK_TARGETS		21	-0.26736447	0.73071104	-0.8290394	0.879263	1	4182	tags=29%, list=22%, signal=37%	FALSE
REACTOME_G_ALPHA_S_SIGNALLING_EVENTS	REACTOME_G_ALPHA_S_SIGNALLING_EVENTS		115	-0.19190554	0.8164557	-0.8281908	0.8792246	1	5571	tags=29%, list=29%, signal=40%	FALSE
ST_PHOSPHOINOSITIDE_3_KINASE_PATHWAY	ST_PHOSPHOINOSITIDE_3_KINASE_PATHWAY		36	-0.23784903	0.7341954	-0.82755995	0.8788401	1	4202	tags=25%, list=22%, signal=32%	FALSE
KEGG_PRIMARY_BILE_ACID_BIOSYNTHESIS	KEGG_PRIMARY_BILE_ACID_BIOSYNTHESIS		15	-0.29515168	0.6921875	-0.8205595	0.89039475	1	2771	tags=20%, list=15%, signal=23%	FALSE
KEGG_ALLOGRAFT_REJECTION	KEGG_ALLOGRAFT_REJECTION		33	-0.23968318	0.72713864	-0.82025373	0.8893893	1	8568	tags=64%, list=45%, signal=116%	FALSE
PID_HEDGEHOG_2PATHWAY	PID_HEDGEHOG_2PATHWAY		22	-0.2656686	0.74681526	-0.8172353	0.8931498	1	7018	tags=45%, list=37%, signal=72%	FALSE
REACTOME_DOWNSTREAM_SIGNAL_TRANSDUCTION	REACTOME_DOWNSTREAM_SIGNAL_TRANSDUCTION		91	-0.19729309	0.7939317	-0.8163536	0.8931739	1	5409	tags=29%, list=29%, signal=40%	FALSE
BIOCARTA_TOB1_PATHWAY	BIOCARTA_TOB1_PATHWAY		19	-0.27132517	0.72424245	-0.8140681	0.8956002	1	7839	tags=58%, list=41%, signal=99%	FALSE
SA_TRKA_RECEPTOR	SA_TRKA_RECEPTOR		17	-0.27992564	0.74254316	-0.8112191	0.8991093	1	7815	tags=53%, list=41%, signal=90%	FALSE
REACTOME_REGULATION_OF_APOPTOSIS	REACTOME_REGULATION_OF_APOPTOSIS		53	-0.21375962	0.8088033	-0.80373687	0.9106839	1	1770	tags=11%, list=9%, signal=12%	FALSE
BIOCARTA_STEM_PATHWAY	BIOCARTA_STEM_PATHWAY		15	-0.2877447	0.73619634	-0.80336833	0.90966713	1	1566	tags=13%, list=8%, signal=15%	FALSE
KEGG_LEISHMANIA_INFECTION	KEGG_LEISHMANIA_INFECTION		62	-0.20344634	0.8170564	-0.8020536	0.91026	1	4757	tags=26%, list=25%, signal=34%	FALSE
REACTOME_AQUAPORIN_MEDIATED_TRANSPORT	REACTOME_AQUAPORIN_MEDIATED_TRANSPORT		49	-0.21473528	0.79886687	-0.7999062	0.91240203	1	7113	tags=37%, list=38%, signal=59%	FALSE
PID_AURORA_A_PATHWAY	PID_AURORA_A_PATHWAY		28	-0.24067686	0.79062957	-0.7987034	0.9128533	1	5231	tags=39%, list=28%, signal=54%	FALSE
REACTOME_DESTABILIZATION_OF_MRNA_BY_AUF1_HNRNP_D0	REACTOME_DESTABILIZATION_OF_MRNA_BY_AUF1_HNRNP_D0		45	-0.22077493	0.8051948	-0.797337	0.9136177	1	2816	tags=16%, list=15%, signal=18%	FALSE
PID_FAS_PATHWAY	PID_FAS_PATHWAY		37	-0.22657456	0.8148148	-0.78891444	0.9259038	1	4591	tags=27%, list=24%, signal=36%	FALSE
KEGG_FATTY_ACID_METABOLISM	KEGG_FATTY_ACID_METABOLISM		39	-0.22146629	0.7837838	-0.7883877	0.925184	1	4109	tags=28%, list=22%, signal=36%	FALSE
REACTOME_PIP3_ACTIVATES_AKT_SIGNALING	REACTOME_PIP3_ACTIVATES_AKT_SIGNALING		27	-0.24036852	0.79911375	-0.7871169	0.92565584	1	5035	tags=26%, list=27%, signal=35%	FALSE
BIOCARTA_RAS_PATHWAY	BIOCARTA_RAS_PATHWAY		23	-0.24871111	0.79204893	-0.7862107	0.92558867	1	5073	tags=35%, list=27%, signal=47%	FALSE
KEGG_PANCREATIC_CANCER	KEGG_PANCREATIC_CANCER		68	-0.19750653	0.83741933	-0.78569937	0.9248399	1	5688	tags=34%, list=30%, signal=48%	FALSE
REACTOME_LIGAND_GATED_ION_CHANNEL_TRANSPORT	REACTOME_LIGAND_GATED_ION_CHANNEL_TRANSPORT		21	-0.2529353	0.7741935	-0.7847227	0.9248818	1	7300	tags=48%, list=39%, signal=78%	FALSE
REACTOME_PLATELET_AGGREGATION_PLUG_FORMATION	REACTOME_PLATELET_AGGREGATION_PLUG_FORMATION		34	-0.22707671	0.78008914	-0.7846734	0.9233925	1	334	tags=9%, list=2%, signal=9%	FALSE
HALLMARK_SPERMATOGENESIS	HALLMARK_SPERMATOGENESIS		131	-0.17991278	0.89762795	-0.7842888	0.92241275	1	5698	tags=27%, list=30%, signal=38%	FALSE
BIOCARTA_TNFR2_PATHWAY	BIOCARTA_TNFR2_PATHWAY		17	-0.2721282	0.7893891	-0.78027546	0.92708445	1	5891	tags=47%, list=31%, signal=68%	FALSE
BIOCARTA_PDGF_PATHWAY	BIOCARTA_PDGF_PATHWAY		32	-0.22833781	0.8067227	-0.77921534	0.927112	1	1274	tags=13%, list=7%, signal=13%	FALSE
REACTOME_RIP_MEDIATED_NFKB_ACTIVATION_VIA_DAI	REACTOME_RIP_MEDIATED_NFKB_ACTIVATION_VIA_DAI		16	-0.2665708	0.7856025	-0.7698993	0.93979675	1	3810	tags=25%, list=20%, signal=31%	FALSE
REACTOME_SIGNALING_BY_FGFR_MUTANTS	REACTOME_SIGNALING_BY_FGFR_MUTANTS		42	-0.21186031	0.8375706	-0.7657232	0.94425577	1	2870	tags=17%, list=15%, signal=20%	FALSE
REACTOME_SIGNALING_BY_INSULIN_RECEPTOR	REACTOME_SIGNALING_BY_INSULIN_RECEPTOR		103	-0.17859669	0.91108245	-0.75934637	0.9519287	1	2321	tags=12%, list=12%, signal=13%	FALSE
PID_IL12_STAT4_PATHWAY	PID_IL12_STAT4_PATHWAY		33	-0.22060226	0.83920366	-0.759273	0.95038646	1	5817	tags=36%, list=31%, signal=52%	FALSE
BIOCARTA_TFF_PATHWAY	BIOCARTA_TFF_PATHWAY		20	-0.25420415	0.8133748	-0.7590872	0.9490702	1	7274	tags=55%, list=38%, signal=89%	FALSE
KEGG_GALACTOSE_METABOLISM	KEGG_GALACTOSE_METABOLISM		26	-0.2333775	0.8081571	-0.75843775	0.9483651	1	7351	tags=50%, list=39%, signal=82%	FALSE
REACTOME_METABOLISM_OF_AMINO_ACIDS_AND_DERIVATIVES	REACTOME_METABOLISM_OF_AMINO_ACIDS_AND_DERIVATIVES		185	-0.16244347	0.9521531	-0.7541529	0.95257986	1	5523	tags=26%, list=29%, signal=36%	FALSE
REACTOME_THROMBOXANE_SIGNALLING_THROUGH_TP_RECEPTOR	REACTOME_THROMBOXANE_SIGNALLING_THROUGH_TP_RECEPTOR		21	-0.24296094	0.7939394	-0.75106585	0.9551339	1	1627	tags=14%, list=9%, signal=16%	FALSE
PID_PI3KCI_AKT_PATHWAY	PID_PI3KCI_AKT_PATHWAY		34	-0.21819654	0.84302324	-0.7501679	0.954734	1	5035	tags=29%, list=27%, signal=40%	FALSE
REACTOME_ACTIVATED_NOTCH1_TRANSMITS_SIGNAL_TO_THE_NUCLEUS	REACTOME_ACTIVATED_NOTCH1_TRANSMITS_SIGNAL_TO_THE_NUCLEUS		26	-0.22937714	0.8220588	-0.7488684	0.95497066	1	1770	tags=15%, list=9%, signal=17%	FALSE
REACTOME_SCFSKP2_MEDIATED_DEGRADATION_OF_P27_P21	REACTOME_SCFSKP2_MEDIATED_DEGRADATION_OF_P27_P21		49	-0.20298703	0.87096775	-0.74710214	0.955795	1	5698	tags=29%, list=30%, signal=41%	FALSE
REACTOME_NRAGE_SIGNALS_DEATH_THROUGH_JNK	REACTOME_NRAGE_SIGNALS_DEATH_THROUGH_JNK		42	-0.20578776	0.8814286	-0.7449176	0.9570509	1	1853	tags=14%, list=10%, signal=16%	FALSE
PID_ERBB2_ERBB3_PATHWAY	PID_ERBB2_ERBB3_PATHWAY		44	-0.20434396	0.8654391	-0.74468035	0.9557388	1	5409	tags=32%, list=29%, signal=44%	FALSE
PID_NOTCH_PATHWAY	PID_NOTCH_PATHWAY		58	-0.19320591	0.87967914	-0.74414206	0.9548575	1	5476	tags=33%, list=29%, signal=46%	FALSE
REACTOME_GABA_B_RECEPTOR_ACTIVATION	REACTOME_GABA_B_RECEPTOR_ACTIVATION		37	-0.20933627	0.8653001	-0.7400506	0.95835245	1	5510	tags=27%, list=29%, signal=38%	FALSE
REACTOME_DOWNREGULATION_OF_TGF_BETA_RECEPTOR_SIGNALING	REACTOME_DOWNREGULATION_OF_TGF_BETA_RECEPTOR_SIGNALING		21	-0.23867223	0.8395245	-0.7369042	0.96062595	1	2969	tags=24%, list=16%, signal=28%	FALSE
REACTOME_GPCR_LIGAND_BINDING	REACTOME_GPCR_LIGAND_BINDING		373	-0.14796726	0.99	-0.72909606	0.96857876	1	5289	tags=24%, list=28%, signal=33%	FALSE
BIOCARTA_LONGEVITY_PATHWAY	BIOCARTA_LONGEVITY_PATHWAY		15	-0.26267064	0.8215434	-0.7290897	0.9669727	1	1160	tags=13%, list=6%, signal=14%	FALSE
KEGG_GLYCOLYSIS_GLUCONEOGENESIS	KEGG_GLYCOLYSIS_GLUCONEOGENESIS		59	-0.18826821	0.90796703	-0.72697556	0.96785086	1	5570	tags=29%, list=29%, signal=41%	FALSE
PID_ARF_3PATHWAY	PID_ARF_3PATHWAY		19	-0.24537012	0.8519108	-0.725832	0.9675788	1	5129	tags=32%, list=27%, signal=43%	FALSE
REACTOME_SHC_MEDIATED_SIGNALLING	REACTOME_SHC_MEDIATED_SIGNALLING		15	-0.25542054	0.8388158	-0.7196048	0.97293097	1	1274	tags=13%, list=7%, signal=14%	FALSE
PID_RHODOPSIN_PATHWAY	PID_RHODOPSIN_PATHWAY		23	-0.22839153	0.8549849	-0.7194253	0.97150356	1	2606	tags=17%, list=14%, signal=20%	FALSE
BIOCARTA_NKCELLS_PATHWAY	BIOCARTA_NKCELLS_PATHWAY		17	-0.24793993	0.859375	-0.71881473	0.9706054	1	7583	tags=53%, list=40%, signal=88%	FALSE
REACTOME_FORMATION_OF_TUBULIN_FOLDING_INTERMEDIATES_BY_CCT_TRIC	REACTOME_FORMATION_OF_TUBULIN_FOLDING_INTERMEDIATES_BY_CCT_TRIC		20	-0.23437022	0.8547401	-0.714211	0.97385776	1	4864	tags=40%, list=26%, signal=54%	FALSE
HALLMARK_KRAS_SIGNALING_DN	HALLMARK_KRAS_SIGNALING_DN		196	-0.15487099	0.98194945	-0.7131014	0.97337407	1	5178	tags=25%, list=27%, signal=34%	FALSE
BIOCARTA_TH1TH2_PATHWAY	BIOCARTA_TH1TH2_PATHWAY		19	-0.23721126	0.86752826	-0.7116113	0.97333735	1	8243	tags=58%, list=44%, signal=103%	FALSE
HALLMARK_ADIPOGENESIS	HALLMARK_ADIPOGENESIS		194	-0.15364629	0.97563803	-0.71142226	0.9719274	1	5783	tags=29%, list=31%, signal=42%	FALSE
REACTOME_SIGNALING_BY_SCF_KIT	REACTOME_SIGNALING_BY_SCF_KIT		75	-0.17480324	0.94133335	-0.70828754	0.97355926	1	3019	tags=15%, list=16%, signal=17%	FALSE
PID_TRAIL_PATHWAY	PID_TRAIL_PATHWAY		27	-0.21508546	0.88112926	-0.7057926	0.974418	1	5941	tags=33%, list=31%, signal=49%	FALSE
PID_RXR_VDR_PATHWAY	PID_RXR_VDR_PATHWAY		26	-0.2155241	0.88670695	-0.7001106	0.9781863	1	5534	tags=38%, list=29%, signal=54%	FALSE
REACTOME_IMMUNOREGULATORY_INTERACTIONS_BETWEEN_A_LYMPHOID_AND_A_NON_LYMPHOID_CELL	REACTOME_IMMUNOREGULATORY_INTERACTIONS_BETWEEN_A_LYMPHOID_AND_A_NON_LYMPHOID_CELL		57	-0.18215814	0.93475175	-0.698034	0.9785779	1	6711	tags=42%, list=36%, signal=65%	FALSE
REACTOME_INWARDLY_RECTIFYING_K_CHANNELS	REACTOME_INWARDLY_RECTIFYING_K_CHANNELS		30	-0.21006078	0.8937409	-0.6962815	0.9785744	1	1352	tags=10%, list=7%, signal=11%	FALSE
REACTOME_PI3K_AKT_ACTIVATION	REACTOME_PI3K_AKT_ACTIVATION		36	-0.1994024	0.9314456	-0.6899891	0.98263925	1	5035	tags=25%, list=27%, signal=34%	FALSE
REACTOME_PEROXISOMAL_LIPID_METABOLISM	REACTOME_PEROXISOMAL_LIPID_METABOLISM		19	-0.23128206	0.8880484	-0.68881565	0.98204106	1	4109	tags=21%, list=22%, signal=27%	FALSE
BIOCARTA_CK1_PATHWAY	BIOCARTA_CK1_PATHWAY		17	-0.22965139	0.9049844	-0.6825887	0.9857929	1	8081	tags=59%, list=43%, signal=103%	FALSE
PID_REELIN_PATHWAY	PID_REELIN_PATHWAY		28	-0.20716451	0.90404797	-0.6821677	0.98451626	1	7081	tags=46%, list=37%, signal=74%	FALSE
KEGG_PANTOTHENATE_AND_COA_BIOSYNTHESIS	KEGG_PANTOTHENATE_AND_COA_BIOSYNTHESIS		16	-0.23484251	0.8776435	-0.6719194	0.99091476	1	6307	tags=38%, list=33%, signal=56%	FALSE
REACTOME_TRANSMISSION_ACROSS_CHEMICAL_SYNAPSES	REACTOME_TRANSMISSION_ACROSS_CHEMICAL_SYNAPSES		180	-0.1448748	0.9976825	-0.6705797	0.99034315	1	3997	tags=17%, list=21%, signal=22%	FALSE
REACTOME_COSTIMULATION_BY_THE_CD28_FAMILY	REACTOME_COSTIMULATION_BY_THE_CD28_FAMILY		58	-0.17246535	0.9624478	-0.6687998	0.99010897	1	7706	tags=47%, list=41%, signal=78%	FALSE
REACTOME_ANTIGEN_PROCESSING_CROSS_PRESENTATION	REACTOME_ANTIGEN_PROCESSING_CROSS_PRESENTATION		66	-0.16974427	0.97483444	-0.6666898	0.98998314	1	2238	tags=12%, list=12%, signal=14%	FALSE
SIG_CD40PATHWAYMAP	SIG_CD40PATHWAYMAP		33	-0.19064507	0.938326	-0.660553	0.992735	1	5719	tags=33%, list=30%, signal=48%	FALSE
KEGG_NITROGEN_METABOLISM	KEGG_NITROGEN_METABOLISM		23	-0.20675789	0.93026704	-0.65510964	0.9947062	1	7388	tags=48%, list=39%, signal=78%	FALSE
KEGG_STARCH_AND_SUCROSE_METABOLISM	KEGG_STARCH_AND_SUCROSE_METABOLISM		36	-0.18656583	0.9451852	-0.6501806	0.99629956	1	3483	tags=19%, list=18%, signal=24%	FALSE
REACTOME_G_PROTEIN_ACTIVATION	REACTOME_G_PROTEIN_ACTIVATION		26	-0.20191824	0.9307229	-0.64668524	0.9968165	1	6904	tags=38%, list=37%, signal=61%	FALSE
REACTOME_PI_3K_CASCADE	REACTOME_PI_3K_CASCADE		54	-0.16841368	0.9747899	-0.6418292	0.99809235	1	5149	tags=22%, list=27%, signal=30%	FALSE
REACTOME_G_BETA_GAMMA_SIGNALLING_THROUGH_PLC_BETA	REACTOME_G_BETA_GAMMA_SIGNALLING_THROUGH_PLC_BETA		19	-0.2112052	0.93977815	-0.6268888	1	1	3969	tags=21%, list=21%, signal=27%	FALSE
BIOCARTA_IL12_PATHWAY	BIOCARTA_IL12_PATHWAY		21	-0.20087962	0.94392526	-0.6266723	1	1	7810	tags=52%, list=41%, signal=89%	FALSE
REACTOME_CDK_MEDIATED_PHOSPHORYLATION_AND_REMOVAL_OF_CDC6	REACTOME_CDK_MEDIATED_PHOSPHORYLATION_AND_REMOVAL_OF_CDC6		43	-0.17199299	0.982906	-0.6239908	1	1	1770	tags=9%, list=9%, signal=10%	FALSE
BIOCARTA_GCR_PATHWAY	BIOCARTA_GCR_PATHWAY		19	-0.20536818	0.9356288	-0.6215242	1	1	957	tags=11%, list=5%, signal=11%	FALSE
REACTOME_PYRUVATE_METABOLISM_AND_CITRIC_ACID_TCA_CYCLE	REACTOME_PYRUVATE_METABOLISM_AND_CITRIC_ACID_TCA_CYCLE		37	-0.17630447	0.9717262	-0.6179188	1	1	7905	tags=46%, list=42%, signal=79%	FALSE
REACTOME_GLYCOLYSIS	REACTOME_GLYCOLYSIS		26	-0.18707362	0.9623734	-0.6118752	1	1	5572	tags=35%, list=29%, signal=49%	FALSE
PID_ANTHRAX_PATHWAY	PID_ANTHRAX_PATHWAY		17	-0.20802774	0.943662	-0.6075629	1	1	303	tags=6%, list=2%, signal=6%	FALSE
PID_CXCR3_PATHWAY	PID_CXCR3_PATHWAY		43	-0.16664319	0.9722222	-0.5996122	1	1	6649	tags=33%, list=35%, signal=50%	FALSE
REACTOME_NEGATIVE_REGULATION_OF_FGFR_SIGNALING	REACTOME_NEGATIVE_REGULATION_OF_FGFR_SIGNALING		36	-0.17179526	0.9792285	-0.59310704	1	1	7831	tags=44%, list=41%, signal=76%	FALSE
REACTOME_INHIBITION_OF_VOLTAGE_GATED_CA2_CHANNELS_VIA_GBETA_GAMMA_SUBUNITS	REACTOME_INHIBITION_OF_VOLTAGE_GATED_CA2_CHANNELS_VIA_GBETA_GAMMA_SUBUNITS		24	-0.18600449	0.9470499	-0.59199864	1	1	1352	tags=8%, list=7%, signal=9%	FALSE
REACTOME_CYCLIN_A_B1_ASSOCIATED_EVENTS_DURING_G2_M_TRANSITION	REACTOME_CYCLIN_A_B1_ASSOCIATED_EVENTS_DURING_G2_M_TRANSITION		15	-0.21204087	0.96036583	-0.590003	1	1	5698	tags=33%, list=30%, signal=48%	FALSE
KEGG_CITRATE_CYCLE_TCA_CYCLE	KEGG_CITRATE_CYCLE_TCA_CYCLE		28	-0.1801025	0.97910446	-0.58690304	1	1	7905	tags=46%, list=42%, signal=80%	FALSE
REACTOME_PYRUVATE_METABOLISM	REACTOME_PYRUVATE_METABOLISM		17	-0.20187876	0.9486781	-0.58480066	1	1	6095	tags=35%, list=32%, signal=52%	FALSE
KEGG_PROPANOATE_METABOLISM	KEGG_PROPANOATE_METABOLISM		31	-0.17144854	0.97193503	-0.5836798	1	1	7421	tags=42%, list=39%, signal=69%	FALSE
BIOCARTA_IL17_PATHWAY	BIOCARTA_IL17_PATHWAY		15	-0.20637038	0.95886075	-0.57957643	1	1	1566	tags=13%, list=8%, signal=15%	FALSE
REACTOME_TIE2_SIGNALING	REACTOME_TIE2_SIGNALING		17	-0.19276193	0.9706337	-0.5628761	1	1	4591	tags=24%, list=24%, signal=31%	FALSE
KEGG_SNARE_INTERACTIONS_IN_VESICULAR_TRANSPORT	KEGG_SNARE_INTERACTIONS_IN_VESICULAR_TRANSPORT		38	-0.15515758	0.99425286	-0.54619426	1	1	4575	tags=24%, list=24%, signal=31%	FALSE
PID_IL27_PATHWAY	PID_IL27_PATHWAY		26	-0.16786689	0.9878971	-0.54453117	1	1	5975	tags=35%, list=32%, signal=51%	FALSE
REACTOME_REGULATION_OF_INSULIN_SECRETION_BY_GLUCAGON_LIKE_PEPTIDE1	REACTOME_REGULATION_OF_INSULIN_SECRETION_BY_GLUCAGON_LIKE_PEPTIDE1		40	-0.14918557	0.9970458	-0.53807396	1	1	2789	tags=13%, list=15%, signal=15%	FALSE
REACTOME_GLUCAGON_TYPE_LIGAND_RECEPTORS	REACTOME_GLUCAGON_TYPE_LIGAND_RECEPTORS		32	-0.1592477	0.99402094	-0.53745496	1	1	3969	tags=19%, list=21%, signal=24%	FALSE
KEGG_FRUCTOSE_AND_MANNOSE_METABOLISM	KEGG_FRUCTOSE_AND_MANNOSE_METABOLISM		33	-0.15537132	0.9928775	-0.53196514	1	1	5775	tags=27%, list=31%, signal=39%	FALSE
REACTOME_INHIBITION_OF_INSULIN_SECRETION_BY_ADRENALINE_NORADRENALINE	REACTOME_INHIBITION_OF_INSULIN_SECRETION_BY_ADRENALINE_NORADRENALINE		24	-0.16214503	0.99242425	-0.51521707	1	1	6729	tags=33%, list=36%, signal=52%	FALSE
REACTOME_LATENT_INFECTION_OF_HOMO_SAPIENS_WITH_MYCOBACTERIUM_TUBERCULOSIS	REACTOME_LATENT_INFECTION_OF_HOMO_SAPIENS_WITH_MYCOBACTERIUM_TUBERCULOSIS		31	-0.14892049	0.99415207	-0.5018681	1	1	7964	tags=48%, list=42%, signal=84%	FALSE
REACTOME_REGULATION_OF_ORNITHINE_DECARBOXYLASE_ODC	REACTOME_REGULATION_OF_ORNITHINE_DECARBOXYLASE_ODC		45	-0.1348995	0.9986053	-0.501519	1	1	2816	tags=11%, list=15%, signal=13%	FALSE
KEGG_PROTEASOME	KEGG_PROTEASOME		40	-0.1340145	1	-0.4778764	1	1	6189	tags=25%, list=33%, signal=37%	FALSE
REACTOME_TRAFFICKING_OF_GLUR2_CONTAINING_AMPA_RECEPTORS	REACTOME_TRAFFICKING_OF_GLUR2_CONTAINING_AMPA_RECEPTORS		15	-0.15684807	0.9921384	-0.44237995	1	1	7856	tags=47%, list=42%, signal=80%	FALSE
REACTOME_CROSS_PRESENTATION_OF_SOLUBLE_EXOGENOUS_ANTIGENS_ENDOSOMES	REACTOME_CROSS_PRESENTATION_OF_SOLUBLE_EXOGENOUS_ANTIGENS_ENDOSOMES		41	-0.1070661	1	-0.38908672	0.9998237	1	864	tags=5%, list=5%, signal=5%	FALSE

Supplementary Table 16: Materials for Western Blotting and Gene Expression Studies

Primary Antibodies

Target	Application	Supplier	Catalog #
SDMA	Western, ELISA, Immunofluorescence	Cell Signaling Technology	13222S
SmD3	ELISA, Immunofluorescence	Abgent	AP12451A
MDM4	Western	Abcam	ab49993
p53	Western	Santa Cruz Biotechnology	sc-126
p21	Western	Sigma	P1484
p21	Western	Cell Signaling Technology	2946
H3	Western	Cell Signaling Technology	9715
Tubulin	Western	Sigma	T9026
Actin	Western	Thermo	PA1-46296
p53	Immunohistochemistry	Thermo	MA5-16387

Gene Expression Reagents

Target	Application	Supplier	Catalog #
HDMX, forward	PCR	Biosearch	SS294168-01
HDMX, reverse	PCR	Biosearch	SS294169-01
GAPDH	PCR	Qiagen	PPH00150F
CCL4	Taqman Assay	Thermo	Hs00605740_g1
CCL3	Taqman Assay	Thermo	Hs04194942_s1
GADD45A	Taqman Assay	Thermo	Hs00169255_m1
SESN1	Taqman Assay	Thermo	Hs00902787_m1
ACTA2	Taqman Assay	Thermo	Hs00426835_g1
MDM2	Taqman Assay	Thermo	Hs01066930_m1
PHLDA3	Taqman Assay	Thermo	Hs00385313_m1
TRIM22	Taqman Assay	Thermo	Hs01001179_m1
EGR2	Taqman Assay	Thermo	Hs00166165_m1
CDKN1A	Taqman Assay	Thermo	Hs00355782_m1
BAX	Taqman Assay	Thermo	Hs00180269_m1
Actin	Taqman Assay	Thermo	401846
GAPDH	Taqman Assay	Thermo	402869

Supplemental Materials and Methods

Compounds

Two PRMT5 inhibitors, GSK3203591 (tool compound, MW = 380.483) and GSK3326595 (clinical compound, MW = 452.549), were synthesized by GSK Medicinal Chemistry. These compounds are used interchangeably due to their similar profiles.

Biochemistry

Biochemical Studies

PRMT5/MEP50 was purified as described previously (1). In summary, Flag-PRMT5 (2-end) was coexpressed with his-MEP50 (2-end) in a baculovirus expression system and purified via NiNTA affinity. Flag-hu-PRMT9-2-845 (NP_612373) prep 3 was expressed in a baculovirus expression system and purified via anti-flag M2 chromatography and Superdex 75 gel filtration chromatography. The following reagents and consumables were purchased from commercial vendors: (1) [3H]-SAM, Adenosyl-L-Methionine, S-[methyl-3H]-, 1mCi (37MBq) PerkinElmer NET155H001MC, (2) SAM, Adenosyl-L-Methionine, New England BioLabs #B9003S, (3) Histone H4 1-21 peptide (SGRGKGGKGLGKGGAKRHRKV) AnaSpec, Inc. USA. Catalog #62499, (4) Histone H2A 1-20 peptide (SGRGKQGGKARAKAKTRSSRGG-K(Biotin)) AnaSpec, Inc. USA. Catalog #64842-1, (5) 96 well reaction plate, Costar #3884, (6) MSPH filter plate, Millipore MSPHNXB50, (7) MicroScint-20, PerkinElmer #6013621, (8) Arginine Binding Ysi SPA beads, PerkinElmer RPNQ0101. The following peptide substrates were custom ordered from 21st Century Biochemicals, USA.: (1) FUBP1 349-369 peptide (NPGGPGPGGRGRGRGQGNWNM-amide), (2) HNRNPH1 211-231 peptide (DRPGAGRGYNSIGRGAGFERM-amide), (3) SmD3 88-106 peptide (MKNKNQGSGAGRGKAAILKAQ-amide), and (4) SAP145 496-516 peptide (NSVPVPRHWCFKRKYLQGKRG –amide). All other reagents were of commercial grade.

Data analysis converted cpm output to % inhibition. IC_{50} values were determined using GraFit software by fitting the experimental % inhibition data to a 3-parameter model, where the upper limit was fixed at 100. To determine K_i^{app}, IC_{50} data was fit to the Cheng-Prusoff equation for competitive or uncompetitive inhibition.

Formula to yield % inhibition:

$$\%\, inhibition = (1 - \left(\frac{cpm_{cmpd} - cpm_{min}}{cpm_{max} - cpm_{min}}\right)) \times 100$$

Where cpm = counts per minute, $cmpd$ = signal in treated well, max = the positive signal control and min = the negative signal control

Formula to yield IC_{50} values:

$$\% \, inhibition = Bottom + \frac{Top - Bottom}{(1 + (IC_{50}/[I])^n)}$$

Where Top = inhibited value fixed to 100%, $Bottom$ = uninhibited value, $[I]$ = concentration of inhibitor, IC_{50} = half maximal inhibitory concentration and n = Hill Slope. The equation assumes that y (% $inhibition$) increases with increasing x ($[I]$).

Formula for Cheng-Prusoff:

$$If \; Competitive; \; K_i^{app} = \frac{IC_{50}}{1 + ([S]/K_m^{app})}$$

$$If \; Uncompetitive; \; K_i^{app} = \frac{IC_{50}}{1 + (K_m^{app}/[S])}$$

Where K_i^{app} is the binding affinity of the inhibitor, IC_{50} is half maximal inhibitory concentration, [S] is the substrate concentration and K_m^{app} is the concentrationof the substrate at which the enzyme activity is half maximal

Mode of Inhibition Studies

GSK3326595 (typically 11-point, 3-fold serial dilution spanning 0.09-5102 nM) was prestamped at 500 nL per well in the reaction plate. Titrating concentrations of SAM comprising of a mixture of ^{3}H-SAM and unlabeled SAM including a constant concentration of peptide (12 µL per well, at 2X in assay buffer) was added to the compound plate. The product inhibitor SAH (24 µL per well of 1 mM stock) was added to select wells for use as the minimum signal control. Enzyme mix (12 µL per well, 2X in assay buffer) was added to initiate the reaction. Final assay conditions consisted of 6 nM PRMT5/MEP50, 40 nM peptide, 50-8000 nM SAM in 50 mM Tris (pH 8.5), 0.002% Tween-20, 4 mM $MgCl_2$ and 1 mM DTT. Reactions were incubated for 30 min at room temperature and quenched with 24 µL per well of 1 mM SAH. Arginine Binding Ysi SPA beads at 3 mg/mL in 0.2M NH4CO3 (24 µL per well) were added, plates were sealed and equilibrated for ≥ 30 min, centrifuged and then read on a MicroBeta (PerkinElmer) following a ≥ 200 min delay to measure the amount of tritium incorporated into the peptide substrate, reported as counts per minute (CPM). The peptide substrate (positive charge) is captured on the bead (negative charge) through charge/charge interaction. Once in proximity to the radiolabel, the bead scintillant is stimulated and emits light. Above methods were used for the peptide mode of inhibition studies expect final assay conditions were 6 nM PRMT5/MEP50, 1.6-1000 nM peptide, 1000 nM SAM (250 nM ^{3}H-SAM and 750 nM unlabeled SAM) in 50 mM Tris (pH 8.5), 0.002% Tween-20, 4 mM $MgCl_2$ and 1 mM DTT.

Analysis of Slow Binding Inhibition

GSK3326595 (typically 11-point, 3-fold serial dilution spanning 0.09-5102 nM) was prestamped at 500 nL per well in the reaction plate. SAM comprising of a mixture of ^{3}H-SAM and unlabeled SAM (6 µL per well, 4X in assay buffer) followed by

PRMT5/MEP50 (12 µL per well, 2X in assay buffer) were added to the compound plate and incubated for 3-60 min. The product inhibitor SAH (24 µL per well of 1 mM stock) was added to select wells for use as the minimum signal control. Peptide mix (6 µL per well, 4X in assay buffer) was added to initiate the reaction. For 0 min preincubation, SAM and peptide were added to the compound plate followed by enzyme addition to initiate the reaction. Final assay conditions consisted of 0.8 or 4 nM PRMT5/MEP50, 50 nM peptide (K_m^{app}), 1000 nM SAM (260 nM ^{3}H-SAM and 740 nM unlabeled SAM, K_m^{app}) in 50 mM Tris (pH 8.5), 0.002% Tween-20, 4 mM $MgCl_2$ and 1 mM DTT. Reactions were incubated for 30 min at room temperature and quenched with 24 µL per well of 1 mM SAH. Arginine Binding Ysi SPA beads were added to the plate and processed as listed above.

IC_{50} determinations with Enzyme:SAM:Inhibitor preincubation

GSK3326595 (typically 11-point, 3-fold serial dilution spanning 0.09-5102 nM) was prestamped at 500 nL per well in the reaction plate. SAM comprising of a mixture of ^{3}H-SAM and unlabeled SAM (6 µL per well, 4X in assay buffer) followed by PRMT5/MEP50 (12 µL per well, 2X in assay buffer) were added to the compound plate and incubated for 60 min. The product inhibitor SAH (24 µL per well of 1 mM stock) was added to select wells for use as the minimum signal control. Peptide mix (6 µL per well, 4X in assay buffer) was added to initiate the reaction. Reactions were incubated for 30 min at room temperature and quenched with 24 µL per well of 1 mM SAH. Arginine Binding Ysi SPA beads were added to the plate and processed as listed above. Final assay conditions consisted of 2-6 nM PRMT5/MEP50, 1000 nM SAM (260 nM ^{3}H-SAM and 740 nM unlabeled SAM, K_m^{app}) in 50 mM Tris (pH 8.5), 0.002% Tween-20, 4 mM $MgCl_2$ and 1 mM DTT plus peptide. Assay was substituted with desired peptide substrate tested at respective K_m^{app} value, H4 1-21 (50 nM), H2A 1-20 (150 nM), SmD3 88-106 (230 nM), FUBPH1 349-369 (230 nM) or HNRNPH1 211-231(230 nM).

Above methods were used for the PRMT9 inhibition studies expect final assay conditions were 3 nM PRMT9, 150 nM SAP145 peptide (K_m^{app}), 3000 nM SAM (510 nM ^{3}H-SAM and 3000 nM unlabeled SAM, K_m^{app}) in 25 mM Tris (pH 8), 0.002% Tween-20, 4 mM $MgCl_2$, 100 mM NaCl and 1 mM DTT. Following quench, a 30 µL volume of the reaction was transferred to a premoistened MSPH filter plate followed by the addition of 150 µL of 0.2M NH4HCO3. The peptide substrate (positive charge) is captured on the filter plate (negative charge) through charge/charge interaction. After an incubation time of 15 min, the plate was washed 4 times with 150 µL per well of 0.2M NH4HCO3 and dried in an oven at 50°C. MicroScint-20 (35 µL per well) was added, plates were sealed and equilibrated for ≥ 30 min and then read on a TopCount (PerkinElmer) to measure the amount of tritium incorporated into the peptide substrate, reported as counts per minute (CPM).

Methyltransferase cross-screening panel

Cross-screening against a panel of 19 protein methyltransferase enzymes (shown in Figure 1D) was done according to general procedures as previously described (2).

Final assay conditions varied slightly depending on the enzyme tested. In summary, GSK3326595 (spanning 2-40,000 nM) was incubated for 30 min with enzyme in assay buffer. Substrate mix comprising of assay buffer with either nucleosome or biotin labeled peptide (10-330 nM) and between 8-2500 nM SAM (mixture of ^{3}H-SAM and unlabeled) was added to initiate the reaction. The final substrate concentration was fixed at the K_m^{app} for each substrate (SAM, peptide or nucleosome) and was specific to the enzyme tested. Reactions were incubated for between 90-120 min at room temperature and quenched with unlabeled SAM. The minimum signal control contained the product inhibitor SAH (50-2000 μM). After an incubation time of 1 hour to overnight, plates were washed with 0.1% Tween-20. Incorporation of radioactivity into substrate was measured by capturing substrate on a PerkinElmer Flashplate, and detecting on a PerkinElmer TopCount.

MethylScan®

MethylScan technology (Cell Signaling Technology) was used to evaluate the cellular targets of PRMT5 in the Z-138 cell line. Methylated peptide was immuno-affinity purified (IAP) with a symmetric dimethyl arginine antibody (Cell Signaling, # 13222S clone D2C3D6). Tandem mass spectrometry (LC-MS/MS) was used to analyze enriched symmetrically dimethylated peptides for qualitative sequence and site identification. Finally, quantitative analysis of methylated peptide fold-change allowed for comparison between DMSO and GSK3203591-treated samples. More negative fold change values correspond to the greatest effect.

Cellular proliferation assays

6-day, 384-well single agent proliferation assays (GSK)

Cells were plated at an optimized seeding density in 384-well format using the Hamilton STARlet automated liquid handling platform and were incubated for 24 hours. On assay Day 0, cells were treated in duplicate with a 20-point, two-fold dilution series of GSK3203591 or GSK3326595 (≤29μM top dose) and ≤0.15% DMSO, and incubated for 6 days. Cell growth was evaluated on Day 0 and Day 6 by measuring luminescence signal generated by addition of CellTiter-Glo reagent (Promega G8462).

Data were fit with a sigmoidal 4-point, 4 parameter, one-site dose response model using Assay Client software, where the following equation was used to generate a concentration response curve:

$$y\,(fit) = A + \frac{(B - A)}{1 + (C/x)^D}$$

Individual curves were QC checked for outlier data points, cell growth (population doubling ≥ 1.5), and proper curve fitting. Growth IC50 (gIC50) values correspond to the concentration intersecting the mid-point of the growth window (between DMSO and T0 values) and represent the concentration of compound required to inhibit the total cell growth observed during the assay by half. Growth IC100 (gIC100) values correspond to

the concentration at which 100% growth inhibition is achieved, based on the growth window. Net cell growth/death values were calculated by subtracting the T0 value (100%) from the Ymin value on the curve, and are a measure of net population cell growth or death. This value reflects the difference between the number of cells seeded at Day 0 (100%) and the number of cells remaining at the endpoint of the assay, such that negative values correspond to net cell death while positive values correspond to net cell growth. dEC50 values correspond to the concentration at which 50% net cell death is observed.

10-day, 384-well single agent proliferation assays (Eurofins panel)

Cells were grown in RPMI-1640, 10% FBS, 2 mM L-alanyl-L-Glutamine, 1mM Na Pyruvate or a special medium in a humidified atmosphere of 5% CO_2 at 37°C. Cells were seeded into 384-well plates and incubated in a humidified atmosphere of 5% CO_2 at 37°C. Compounds were added 24 hours post cell seeding. At the same time, a time zero untreated cell plate was generated. At 168 hours post cell seeding, the cell plates were briefly spun, and media was removed and replaced with fresh growth media. The plates were then re-dosed with the test compound. After a 240 hour incubation period, cells were fixed and stained with fluorescent nuclear dye to allow visualization of nuclei. Compounds were serially diluted 3.16-fold and assayed over 10 concentrations in a final assay concentration of 0.1% DMSO from the highest test concentration specified in the sample information chapter. Automated fluorescence microscopy was carried out using a Molecular Devices ImageXpress Micro XL high-content imager, and images were collected with a 4X objective.

16-bit TIFF images were acquired and analyzed with MetaXpress 5.1.0.41 software. EC50 and IC50 values were calculated using nonlinear regression to fit data to a sigmoidal 4-point, 4 parameter, one-site dose response model, where:

$$y\,(fit) = A + \frac{(B - A)}{1 + (C/x)^D}$$

Curve-fitting, EC_{50} / IC_{50} calculations and report generation was performed using a custom data reduction engine and MathIQ based software (AIM). The cell proliferation assay uses a cell image based analysis technique where cells are fixed and stained with fluorescent nuclear dye to visualize nuclei.

Cell proliferation is measured by the signal intensity of the incorporated nuclear dye. The cell proliferation assay output is referred to as the relative cell count. To determine the cell proliferation end point, the cell proliferation data output is transformed to percent of control (POC) using the following formula:

$$POC = \left(\frac{relative\ cell\ count\ (compound\ wells)}{relative\ cell\ count\ (vehicle\ wells)}\right) * 100$$

Relative cell count IC_{50} is the test compound concentration at 50% of maximal possible response. A relative cell count EC_{50} is the test compound concentration at the curve inflection point or half the effective response (parameter C of the fitted curve solution). GI_{50} is the concentration needed to reduce the observed growth by half. This is the concentration that inhibits the growth midway between untreated cells and the number of cells seeded in the well (Time zero value), and will be reported in this manuscript as gIC_{50} for consistency with GSK data. Activity area in an estimate of the integrated area above the curve. Activity area values generally range from 0-10, where a value of zero indicates no inhibition of proliferation at all concentrations, and a value of 10 indicates complete inhibition of proliferation at all concentrations. In rare instances, values <0 or >10 may be observed. In these instances, values <0 should be considered as equivalent to 0, whereas values >10 should be considered equivalent to 10.

Time zero non-treated plate is used to determine number of doublings in 72 hour assay period: Number of doublings in 72 hours = Log2[Cell number (72 hrs end point) / Cell number (time zero)].

10-day, 96-well single agent proliferation assays (GSK)

10-day growth-death assays were also performed on SW48 (Sigma CLL1008-1VL) and SW48 $p53^{-/-}$ (Sigma CLL1013-1VL) cell lines to evaluate the effect of p53 knockout on proliferation. An extended time course was used in these experiments because Western blotting for SDMA suggested effective target engagement would require longer than 6-days (data not shown). Optimal cell seeding was determined by monitoring proliferation over a range of seeding densities in 96-well format and identifying the seeding density at which cells grow logarithmically at 3, 6, and 10-day time points. The PerkinElmer Zephyr workstation was used to seed cells in 96-well plates at the optimal seeding density in 100µL of culture media supplemented with 10% FBS. Seven plates were prepared per cell line (duplicate plates for Day 0, 3, and 6 reads) and incubated overnight at 37°C in 5% CO2. The following day, cells were treated in duplicate on the Zephyr workstation with a 20-point, two-fold dilution series of GSK3326595 (26µM top dose) and ≤0.15% DMSO. Plates were incubated for the indicated number of days at the conditions described above. Cell growth was measured using CellTiter-Glo reagent (Promega G8462) and luminescence signal was detected using an Envision microplate reader. A plate of untreated cells was read at the time of compound addition to determine the T=0 value representing the starting number of cells. Data were analyzed using XLfit at Graphpad Prism software.

SDMA Analysis

SDMA ELISA

In vitro treated lymphoma cells and frozen tumors powder samples were resuspended in RIPA buffer containing protease/phosphatase inhibitor cocktail (Cell Signaling

#5872S). Tumor powder was homogenized with a hand-held polytron (IKA T10 basic Ultra Turrax) on ice, followed by sonication with a hand-held polytron (IKA T10 basic Ultra Turrax) at setting 6, on ice (20 seconds/twice). Cell lysates were spun at 12,000 rpm for 10 minutes at 4ºC and cleared lysate was transferred to a new tube.

Cell lysates were diluted with PBS Carbonate-Bicarbonate buffer pH 9.6 (Sigma, #C3041) and added to black high binding plates (Nunc Maxisorp #437111). A titration (serial 2-fold dilution from 960 to 2 ng/well) of vehicle treated control lysate was used to evaluate linear range. Lysate was incubated for 2 hours at room temperature. Lysate was removed and plates were washed 4 times with 200uL wash solution (PBST, Cell Signaling, #9809S), followed by a 2 hour block step with PBST with 1% bovine serum albumen (BSA; Alfa Aesar, #J61089). Prior to addition of the primary antibody, the plates are washed as previously described.

The primary antibodies, SDMA (Cell Signaling, # 13222S clone D2C3D6, lot #2) and SMD3 (Abgent, #AP12451A), were diluted with 1% BSA buffer, 100uL added/well. Plates were incubated overnight with rocking at 4ºC. Primary antibody was removed, plates were washed as previously described, and secondary HRP-conjugated anti-rabbit antibody (Cell Signaling Technology #7074) was added and incubated at room temperature for one hour. Following the final wash step, plates were tapped briefly on paper towels to remove any residual wash buffer and 100uL of Luminata Forte substrate (Millipore #WBKUF0500) was added to each well. Plates were incubated for 15 minutes with rocking and read on an Envision plate reader using a 96 well luminescence protocol. SDMA levels were normalized to SMD3 values and IC_{50} values were determined using a 4 parameter curve fit.

SDMA Imaging Assay

The SDMA imaging assay was used to evaluate the potency of GSK3326595 in breast cancer cell lines. Cells were plated at 2,000 cells per well in an optical bottom 96-well cell culture plate (Nunc 1256670) and allowed to adhere overnight. Cells were treated for 72 hours with a 10-point 5-fold dilution series (dose range = 0.8 pM – 1500 nM), fixed with 4% formaldehyde (ThemoScientific 28908), permeabilized, and blocked with 10% goat serum (Gibco 16210) in immunofluorescence (IF) buffer (130mM NaCl, 7mM Na2HPO4, 3.5 mM NaH2PO4, 7.7mM NaN3, 0.1% BSA, 0.2% Triton X, 0.05% Tween 20) prior to overnight incubation with antibodies specific for SDMA (Cell Signaling Technology # 13138BF, lot D2C3D6) and SMD3 (Abgent # AP12451A, lot 150508HF). Following washes with IF buffer, cells were further incubated with fluorescent secondary antibodies (Invitrogen Oregon Green Anti-rabbit #11038) and DAPI nuclear staining (Invitrogen #D357) prior to quantification of nuclear SDMA and SMD3 levels via Molecular Devices MetaExpress high-content fluorescent imager using a 10x objective to image a minimum of 4 sites per well. Image analysis was performed with ImageExpress software using Multiwavelength scoring to quantitate each fluorescent signal (FITC, and DAPI) in each well. SDMA levels were then normalized to SMD3 levels and IC50, IC90, EC50 and EC90 values were determined using a 4 parameter curve fit.

Cell Cycle and Cell Death Analysis

Cell cycle analysis

Cell cycle phase distribution was examined by flow cytometry of propidium iodide stained nuclei. Cells were treated with PRMT5 inhibitor or DMSO for the indicated period of time, depending on experiment. Pellets were processed using the BD Biosciences CycleTest kit according to the manufacturer's instructions and were evaluated using a FACSCalibur flow cytometer. FlowJo software was used to determine the cell cycle distribution.

Annexin V and 7AAD Staining

Early apoptosis and cell death were evaluated using Annexin V staining (APC Annexin V, BD Biosciences, #550475) and 7-AAD (BD Bioscience #559925) and performed as recommended by the manufacturer. Cells were treated with PRMT5 inhibitor for 3 or 6 days or Camptothecan, a positive control, for 12 hours. Compound treated cells were washed with cold PBS, and cell pellets were resuspended in 1X binding buffer (10X binding buffer, BD Bioscience #556454) to a concentration of a million cells per ml. Stain was added to 100 ul cells in duplicate, and incubated for 15 minutes at room temperature followed by further 5 fold dilution with 1 X binding buffer. Fluorescent events were collected on a FACSCalibur Becton Dickonson (BD Biosciences) and data were analyzed using FlowJo (Treestar, Ashland) to discriminate live cells (Annexin V (-) 7-AAD (-)) from apoptotic cells (AnnexinV (+)7-AAD (-)) from dead cells Annexin V (+/-) 7-AAD (+)). Individual control samples unstained, and stained with each fluorochrome independently are used to establish spectral overlap. Compensation was calculated post data collection within the software.

RNA-Seq

RNA-Seq sample preparation and differential expression

Z-138, SU-DHL-6, JVM-2, SU-DHL-5, MAVER-1, MINO, SU-DHL-4, and REC-1 cell lines were treated with either DMSO (control) or 200 nM GSK3326595. Cells were collected for RNA-seq analysis post 3 and 6 days. Z-138, GRANTA-519, and DOHH-2 cell lines were treated with either DMSO (control) or 1000 nM GSK3203591. Cells were collected for RNA-seq analysis post 2 and 4 days.

RNA was extracted from cell pellets with Trizol using Qiagen's RNeasy Mini Kit. Libraries were prepared using Illumina TruSeq Stranded Total RNA with Ribo-Zero Gold Library Prep and sequenced on Illumina HiSeq2500 to 60-80 million PE 2 x 101bp. RNA-seq fastq files were aligned to human hg38 with the gencode GRCh38.v23 annotation using a 2-pass STAR-2.5.2b (3) and then ribosomal and mitochondrial alignments were removed. Differential gene expression was calculated using Cufflinks2 suite considering genes with q-value less than 0.05, and a minimum expression >1

FPKM, and a minimum 2-fold change in expression as significant (4). Individual gene expressions in FPKM values were taken from the normalized differential values from the cuffdiff output files.

Gene pathway analysis
Gene symbols were submitted for MsigDB comparing against the CP, CP:BIOCARTA, CP:KEGG, CP:REACTOME, and Hallmark gene sets (Broad), as well as using the R package clusterProfiler and ReactomePA (5,6), reporting only the most significant gene set.

Alternative splicing analysis
To calculate global splicing events, alignment files from STAR were used as input for rMATS and run using default parameters with the –novelSS flag to identify novel splicing events. Events were consifered significant with FDR < 0.01, SD < 0.2, junction coverage > 10 for both the spliced in and excluded junctions, and -0.2 < IncLevelDifference > 0.2 for alternative splicing events from “ JunctionCountOnly” output files (6).

MDM4 splicing and p53 pathway analysis

MDM4 splicing and p53 pathway analysis

RNA isolation and RT-PCR for MDM4 splicing analysis
RNA isolated from cell pellets was converted to cDNA using ABI High Capacity Reverse Transcription Reagents (4274966). Reverse transcription reactions were incubated in a thermal cycler for 10 minutes at 25°C, 2 hours at 37°C, and 5 minutes at 85°C. Master mix was prepared using Thermo DreamTaq Master Mix (K1081), 20uM HDMX forward primer (Biosearch Technologies, catalog # SS294168-01), 20uM HDMX reverse primer (Biosearch Technologies, catalog #SS294169-01), and nuclease-free water. A control master mix was prepared similarly using GAPDH primers (Qiagen PPH00150F). PCR reactions were prepared by combining 10uL of 9-fold diluted cDNA with 40uL of master mix and were incubated on a thermal cycler as follows: 1 cycle of 95°C for 5 minutes; 26 cycles of 95°C for 40 seconds, 58°C for 30 seconds, and 72°C for 40 seconds; then 1 cycle of 72°C for 4 minutes. Reaction products were separated using agarose ethidium bromide gel electrophoresis and visualized using a Bio-Rad VersaDoc Model 5000 imaging system. Images were captured using Bio-Rad Quantity One software at various exposure times to optimize signal strength and avoid saturation. Images are shown using an inverted color scheme for easier visualization.

Western Blotting for p53 pathway analysis
Gel samples were prepared by combining whole cell lysates with NuPAGE LDS Sample Buffer (NP007), NuPAGE Sample Reducing Agent (NP009), and RIPA buffer (Sigma R0278). Samples were loaded onto NuPAGE Novex 4-12% Bis-Tris protein gels and run in MES SDS (NP002) buffer at 150V until bands were well resolved. Proteins were transferred onto nitrocellulose with an iBlot and probed membranes were developed

using a LiCOR Odyssey scanner. Blots were scanned at various exposures to optimize signal strength and avoid saturation. Antibodies against p53 (Santa Cruz sc-126), p21 (Sigma P1484 or Cell Signaling 2946), SDMA (Cell Signaling 13138BF, clone #D2C3D6), MDM4 (Abcam ab49993), H3 (Cell Signaling 9715), tubulin (Sigma T9026), and actin (Thermo PA1-46296) were used to probe protein expression levels in Western blotting experiments.

Sensitivity Correlations

A 10-point dose response growth assay was performed 10 days post GSK3203591 treatment in a panel of 240 cell lines representing various tumor types. Activity area was calculated for each cell line and used as the metric for sensitivity.

Whole exome-seq mutation data for 1000 cell lines was downloaded from the Genomics of Drug Sensitivity in Cancer (Sanger Institute's **http://www.cancerrxgene.org**) and used to calculate each gene's mutant versus wild type activity area using a Wilcoxon rank sum test from the 185 common cell lines. Waterfall plots of cell lines sorted by increasing activity area with mutant cell lines indicated in green were generated and p-value calculated using R.

CCLE microarray RMA normalized expression values across 1019 cell lines were used to correlate each gene's expression profile using a Pearson correlation with activity area in the 178 common cell lines. Genes were sorted by Pearson correlation and the sorted gene list submitted to GSEA pre-ranked analysis interrogating hallmark gene sets, canonical pathways, BioCarta, Kegg, and Reactome gene sets (Broad Institute). Increasing false discovery rates (FDR) are plotted against normalized enrichment scores of the gene sets. An example gene of the lowest p-value from each of "Reactome_mRNA_Splicing", "Reactome_nonsense_mediated_decay_by_the_exon_junction_complex", and "PID_p53_regulation_pathway" gene sets is plotted comparing cell lines sorted by activity area. Each of the CLNS1A, RIOK1, and the ratio of the two's gene expression, is plotted comparing cell lines sorted by activity area.

A heatmap of all genes within the Reactome_mRNA_splicing gene set (the lowest p-value) in the 20 most resistant and sensitive cell lines hierarchical clustered by euclidean distance.

MTAP gene level copy data was downloaded from the Genomics of Drug Sensitivity in Cancer for 1000 cell lines and the 185 cell lines in common had a Wilcoxon rank sum test performed to test the difference in the mean of sensitivities between MTAP homozygous deleted cell lines and those with at least one functional copy.

Lentiviruses

Lentiviruses were engineered to express MDM4-FL (generated in-house), EGFP (generated in-house), p53-targeting shRNA (Santa Cruz, sc-29435-V), or a scrambled non-targeting shRNA (Santa Cruz, sc-108080). Lentiviral particles were added at a multiplicity of transduction (MOT) of 20 to Z-138 cells that had been seeded into 6-well dishes, then polybrene was added to a final concentration of 50ug/mL. The dishes were centrifuged at 1,000xg for 90 minutes at 37ºC. Cell pellets were collected, supernatant discarded, and pellets were resuspended in culture media. Each pellet was re-seeded into a fresh 6-well dish and then incubated at 37ºC and 5% CO_2 for 48h. Puromycin selection was used to generate and maintain stable cell lines. An anti-MDM4 antibody (Abcam ab49993) was used to evaluate the success of the overexpression via Western blot. Proliferation assays were conducted in 384-well format at 3- and 6-day time points with these cell lines as described earlier.

Gene expression

Gene expression analysis in Z-138 cell line

Z-138 cells were obtained from ATCC, (CRL-3001) and maintained in RPMI-1640 supplemented with 10% horse serum. Cells were incubated at 37°C with 5% CO_2 and split once a week. Z-138 cells were treated with increasing doses of GSK3326595 in 96-well cell culture plates for 2 and 4 days, RNA was isolated using TurboCapture 96 Kit (Qiagen # 72251). Reverse transcription was performed with High Capacity Reverse Transcription reagents (ABI #4374966), followed by qPCR (ABI Taqman Fast Advanced Master mix, cat # 4444557) analysis on ABI ViiA7 real-time PCR machine using the Fast TaqMan Comparative Ct ($2^{-\Delta\Delta Ct}$) method (7). Taqman assays targeted the genes CCL4 (Hs00605740_g1), CCL3 (Hs04194942_s1), GADD45A (Hs00169255_m1), SESN1 (Hs00902787_m1), ACTA2 (Hs00426835_g1), MDM2 (Hs01066930_m1), PHLDA3 (Hs00385313_m1), TRIM22 (Hs01001179_m1), EGR2 (Hs00166165_m1), CDKN1A (Hs00355782_m1), BAX (Hs00180269_m1), actin (Thermo 401846), and GAPDH (Thermo 402869).

Xenograft studies

Z-138 efficacy and PD study

The Z-138 (human B Cell Non-Hodgkin's Lymphoma) cells from ATCC (ATCC No: CRL-3001, Lot No: 57634322) were maintained *in vitro* as a suspension culture in IMDM medium supplemented with 10% heat inactivated horse serum, 100 U/ml penicillin and 100 µg/ml streptomycin at 37 ℃ in atmosphere of 5% CO_2 in air. The tumor cells were routinely subcultured twice weekly with fresh medium. Cells growing in an exponential growth phase were harvested and counted for tumor inoculation.
Each mouse was inoculated subcutaneously at the right flank with Z-138 tumor cells (5 x 10^6 cells/mouse, 50% Matrigel) in 0.2 ml mixture of base media and Matrigel (IMDM:

Matrigel = 100:100) for tumor development. The treatments were started when the tumor size reached 152.98 mm^3 for the tumor efficacy study (day 14 post inoculation). Tumor bearing mice were block randomized into 13 groups and each group has 10 mice. Mice were dosed with either Vehicle [0.5% methylcellulose (Sigma #M0430, St. Louis, MO) or GSK3326595D in vehicle at 0.2, 0.5, 1.4, 4.2, 12.5, 25, 50, 100 mg/kg twice daily, and 50, 100, 200 mg/kg once daily. Twice daily doses were administered 12 hour between doses.

All the procedures related to animal handling, care and the treatment in this study were performed according to the guidelines approved by the Institutional Animal Care and Use Committee (IACUC) of Shanghai Chempartner following the guidance of the Association for Assessment and Accreditation of Laboratory Animal Care (AAALAC). At the time of routine monitoring, the animals were checked for any effects of tumor growth and treatments on normal behavior such as mobility, food and water consumption (by looking only), and body weight gain/loss (body weights were measured twice weekly), corneaopacity, matted fur and any other abnormal effect. Death and observed clinical signs were recorded on the basis of the numbers of animals within each subset. Animals that were observed to be in a continuing deteriorating condition or their tumor size exceeding 3000 mm^3 were euthanized prior to death or before reaching a comatose state.

Tumor size was measured twice weekly in two dimensions using a caliper, and the volume was expressed in mm^3 using the formula: $V = 0.5\ a \times b^2$ where a and b were the long and short diameters of the tumor, respectively. The tumor size was then used for calculations of TGI values. Tumor growth inhibition (TGI) was calculated according to the following equation:

$$TGI\ (\%) = \left(1 - \frac{(TV_{Treatment,Day\ 22} - TV_{Treatment,Day\ 0})}{(TV_{Control,Day\ 22} - TV_{Control,Day\ 0})}\right) * 100\%$$

Treatment groups with limited efficacy were euthanized on treatment day 22, and sampling was 2 hours post the last dose. Subsequently tumors were flash frozen and pulverized.

SDMA ELISA in Z-138 xenograft

SDMA inhibition in frozen tumor powder samples was analyzed by ELISA, as described earlier.

p53 IHC in Z-138 xenograft

Performed IHC detection by using HRP/DAB IHC detection kit (ABC kit, #ab64261) and following the manufacturer's protocol. Anti-p53 rabbit monoclonal, clone SP5 from Thermo Scientific # MA5-16387), used as primary antibody for IHC. Images were taken

with AxioVision software, and cell staining intensity was quantified using the Meta Imaging Series software from Molecular Devices.

Gene expression analysis in Z-138 xenograft

Z-138 xenograft tumors were treated with various doses of GSK3326595 QD (50, 100 and 200 mg/kg) and BID (3, 6, 12.5, 25, 50 and 100 mg/kg) and collected at 2 hours post last dose. Mouse tissue samples were resuspended in QIAzol (Qiagen #79306) lysis reagent and vortexed to completely homogenize lysate. Samples were pre-cleared by MaXtract High Density tubes (Qiagen #129056) and RNA was isolated by RNeasy Mini kit **(**Qiagen #74106). Then, RT-PCR was performed with High Capacity Reverse Transcription reagents (ABI #4374966), followed by qPCR (ABI Taqman Fast Advanced Master mix, cat # 4444557) analysis on ABI ViiA7 real-time PCR machine using the Fast TaqMan $\Delta\Delta C_t$ method. A panel of 11 GSK3326595-responsive genes (see "Gene Expression in Z-138 cell line" for gene list and catalog information) was analyzed and normalized to two housekeeping genes, GAPDH and ACTIN. The average fold change in the treatment group vs. the vehicle group as well as the number of genes with higher than 2-fold change in gene expression for each dose were calculated.

REC-1 efficacy study

We tested GSK3326595 anti-tumor activity in a xenograft model of REC-1, an MCL cell line that is not sensitive at 6 days of exposure to PRMT5 inhibition *in vitro*.

The study consisted of six groups (n = 10) of mice bearing subcutaneous REC-1 tumors on Day 1. Group 1 served as the primary control group and received vehicle 1 (0.5% methylcellulose), orally (p.o.) twice daily to the end of the study (bid to end), first day one dose. Group 2 was given GSK3326595 p.o., at 126.1 mg/kg (79.3% active compound), BID to end, first day one dose. Tumors were measured twice per week. The study endpoint was a mean tumor volume of 2000 mm^3 in the control group or 25 days, whichever came first. The study reached the 2000 mm^3 endpoint on Day 25. The control tumor growth in this study was in line with our historical data although tumors in this group reached endpoint with measurements taken by an alternate technician. For this reason TGI analysis was performed on Day 22.

Treatment outcome was based on percent tumor growth inhibition (%TGI), defined as the percent difference between Day 22 median tumor volumes (MTVs) of treated and control mice. MTV values for groups were compared with the non-parametric Mann-Whitney U test. The results were analyzed and were deemed statistically significant at $P \leq 0.05$. A treatment that produced at least 60% TGI was considered to have potential therapeutic activity. Animals were also monitored for partial regression (PR) and complete regression (CR) responses. Treatment tolerability was assessed by body Weight (BW) measurements and frequent observation for clinical signs of treatment-related (TR) side effects.

1. Chan-Penebre E, Kuplast KG, Majer CR, Boriack-Sjodin PA, Wigle TJ, Johnston LD*, et al.* A selective inhibitor of PRMT5 with in vivo and in vitro potency in MCL models. Nat Chem Biol **2015**;11(6):432-7 doi 10.1038/nchembio.1810.
2. Knutson SK, Wigle TJ, Warholic NM, Sneeringer CJ, Allain CJ, Klaus CR*, et al.* A selective inhibitor of EZH2 blocks H3K27 methylation and kills mutant lymphoma cells. Nat Chem Biol **2012**;8(11):890-6 doi 10.1038/nchembio.1084.
3. Dobin A, Davis CA, Schlesinger F, Drenkow J, Zaleski C, Jha S*, et al.* STAR: ultrafast universal RNA-seq aligner. Bioinformatics **2013**;29(1):15-21 doi 10.1093/bioinformatics/bts635.
4. Trapnell C, Hendrickson DG, Sauvageau M, Goff L, Rinn JL, Pachter L. Differential analysis of gene regulation at transcript resolution with RNA-seq. Nat Biotechnol **2013**;31(1):46-53 doi 10.1038/nbt.2450.
5. Yu G, Wang LG, Han Y, He QY. clusterProfiler: an R package for comparing biological themes among gene clusters. OMICS **2012**;16(5):284-7 doi 10.1089/omi.2011.0118.
6. Shen S, Park JW, Lu ZX, Lin L, Henry MD, Wu YN*, et al.* rMATS: robust and flexible detection of differential alternative splicing from replicate RNA-Seq data. Proc Natl Acad Sci U S A **2014**;111(51):E5593-601 doi 10.1073/pnas.1419161111.
7. Livak KJ, Schmittgen TD. Analysis of relative gene expression data using real-time quantitative PCR and the 2(-Delta Delta C(T)) Method. Methods **2001**;25(4):402-8 doi 10.1006/meth.2001.1262.
